# Reverse Anomeric Effects in Pyranose and Furanose Isomers in Schiff Bases of d‑Galactosamine

**DOI:** 10.1021/acs.joc.5c00796

**Published:** 2025-09-17

**Authors:** Esther Matamoros, Esther M. S. Pérez, Pedro Cintas, Mark E. Light, Juan C. Palacios

**Affiliations:** † Departamento de Química Orgánica e Inorgánica, Facultad de Ciencias, and Instituto del Agua, Cambio Climático y Sostenibilidad (IACYS), 16759 Universidad de Extremadura, 06006 Badajoz, Spain; ‡ Departamento de Química Orgánica, Universidad de Málaga, Campus Teatinos s/n, 29071 Málaga, Spain; § Instituto de Investigación Biomédica de Málaga y Plataforma en Nanomedicina-IBIMA, Plataforma Bionand, Parque Tecnológico de Andalucía, 29590 Málaga, Spain; ∥ Department of Chemistry, Faculty of Natural and Environmental Sciences, 7423 University of Southampton, Southampton SO17 1BJ, U.K.

## Abstract

The present study discloses for the first time furanose structures in imines derived from 2-amino-2-deoxyaldoses, thus assessing the anomeric equilibria. In DMSO solution, imines derived from d-galactosamine, [(2*R*,3*R*,4*R*,5*R*,6*R*)-3-amino-6-hydroxymethyltetrahydropyran-2,4,5-triol], exist in equilibrium between α and β anomers of the corresponding pyranose and furanose forms. In parallel analogy to glycoimines existing exclusively in pyranoid structures, β-anomers are extensively favored, a bias that can now be ascribed with confidence to a genuine reverse anomeric effect. Specifically, this effect describes a conformational preference opposite to the anomeric effect, thereby implying a destabilization of the axial anomer (α-anomer) together with pure steric effects. As extensively detailed throughout this paper by experimental and computational methods, the core argument is the existence, in both α-pyranose and α-furanose imines, of an intramolecular hydrogen bond between the anomeric hydroxyl and the nitrogen atom that inhibits the *exo*-anomeric effect. Moreover, solvation may synergistically reinforce this inhibition of the *exo*-anomeric effect, thus favoring the predominance of the β-anomer.

## Graphical abstract

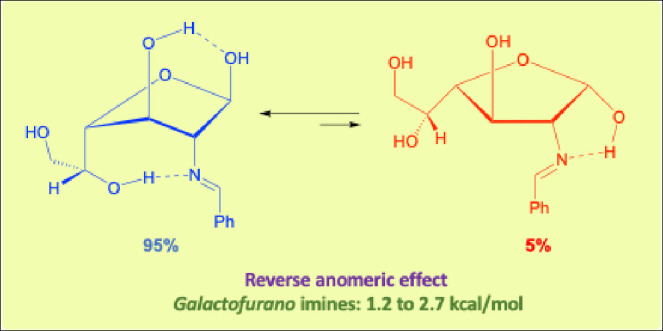

## Introduction and Background

Among aldohexoses, galactose exhibits a unique and distinctive behavior, as its five-membered furanose ring may be a dominant structural motif in numerous living organisms, especially bacteria, fungi, protozoa and plants.^1^ Gram-negative bacteria possess an outer lipopolysaccharide (LPS) membrane that lies outside the peptidoglycan wall. The LPS layer has antigens containing sugars in the furanose form. d-Galactofuranose is arguably the most abundant furanose-sugar present in the cell envelopes of *Escherichia coli*, *Mycobacterium tuberculosis*, *Klebsiella pneumoniae*, *Salmonella typhimurium*, or *Shigella dysenteriae*.^2,3^ Furanose residues have likewise been identified in plant cells and contribute to maintain the rigidity and impermeability of the cell wall.^4^ Overall, such a furanose array constitutes a protective arsenal, which is crucial for bacteria growth and survival. In fact deletion of the *glfA* gene, corresponding to an enzyme required for galactofuranose biosynthesis in the opportunistic pathogen *Aspergillus fumigatus*, rendered the fungus cells less virulent and sensitive to therapeutic agents.^5^ Accordingly, while mammalian glycans incorporate only the pyranose form of galactose, methods to distinguish between galactopyranose and galactofuranose isomers of different anomers (either α or β) are relevant for detecting and identifying the fingerprints present in bacterial glycans.^6^ Unlike unsubstituted d-galactose, the presence of other functional groups, particularly at C-2, should significantly modify the mutarotational and/or anomerization equilibria and, in context, the corresponding 2-amino-2-deoxy derivatives are suitable for modulating stereoelectronic effects capable of altering isomer populations.

Although the first imines derived from 2-amino-2-deoxyaldoses were reported at the dawn of the 20th century (Scheme 1),^7^ imines (**3**, **4**) derived from d-glucosamine (**1**) and d-galactosamine (**2**) were isolated later from hydrolyzed polysaccharides of egg albumin,^8^ cultures of *Bacteria dysenteriae*,^9^ β-heparin,^10^ as well as from *Streptomyces* strains.^11^ Such transformations represent suitable methods for isolating compounds **1** and **2** from complex reaction mixtures of either synthetic or natural origin.

**Scheme 1:**
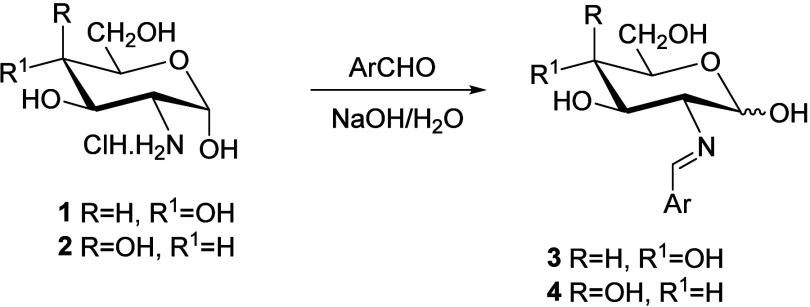
Short Syntheses of Imines Derived from Unprotected 2-Amino-2-deoxyaldoses

Other authors,^9^ while investigating the formation of Schiff bases, were able to isolate small amounts of d-glucosamine and d-galactosamine, and concluded that imines **5–** **9**, derived from *p*-nitrobenzaldehyde, *p*-nitrocinnamaldehyde, 2-hydroxy-1-naphthaldehyde, and 4-hydroxy-3-methoxy-benzaldehyde, respectively, were the most appropriate derivatives for the isolation and characterization of d-galactosamine from complex mixtures without interfering with the presence of other carbohydrates or amino acids. 2-Hydroxy-1-naphthaldehyde derivatives were the most insoluble compounds and enabled the isolation of up to 30 mg of free d-galactosamine.

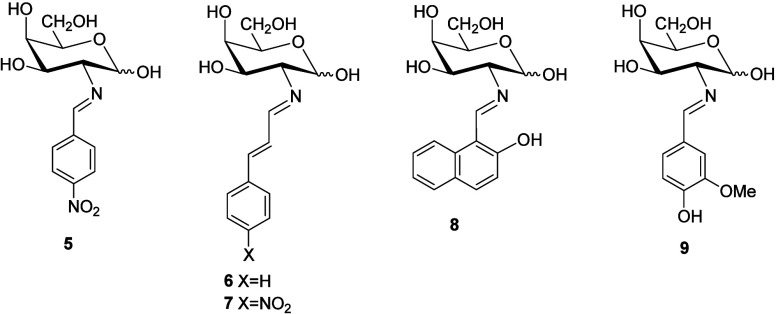



The anisal derivative of d-galactosamine (**10**) was synthesized and characterized during studies aimed at elucidating the structure of β-heparin,^10^ which differs from standard heparin, also called α-heparin, by the presence of d-galactosamine instead of d-glucosamine. Between 1952 and 1961, other groups^12,13^ used Schiff bases from 2-hydroxy-1-naphthaldehyde to characterize various mono-, di-, and trimethyl derivatives of d-galactosamine (**11–** **17**), albeit the only valuable structural data reported were their optical rotation and the existence of mutarotation.

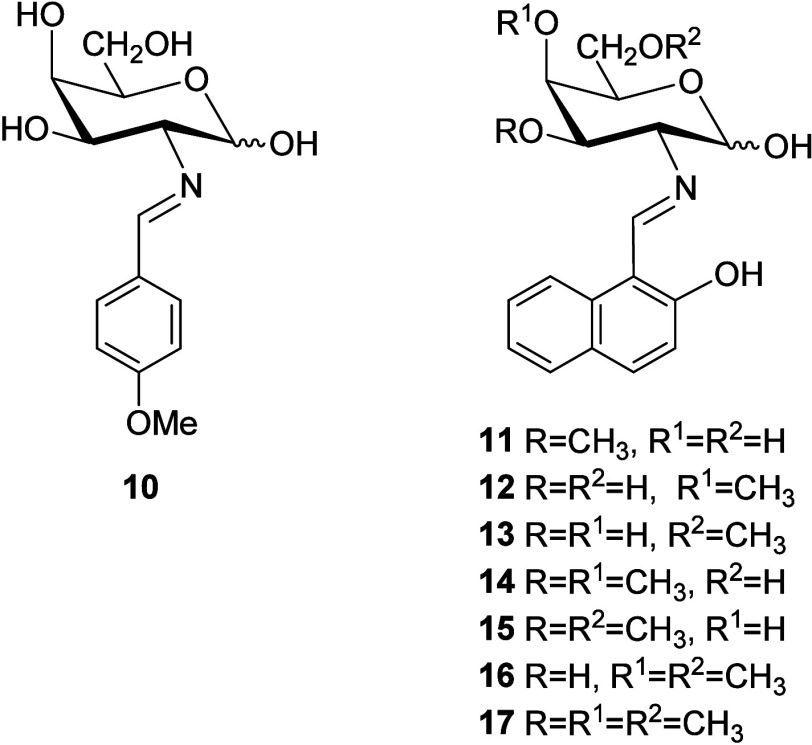



In contrast, the reaction of 2-amino-2-deoxy-d-galactose (**2**) with acetylacetone leads to a pyrrole derivative (**19**) through the intermediacy of enamine **18**.^14^ However, it was found^15^ that compound **2** condensed with two molecules of acetylacetone. The first reaction takes place with the amino group and then, the resulting product undergoes aldehydic condensation with a second molecule of acetylacetone. The product in question, 1-*C*-(1-acetylacetonyl)-2-deoxy-2-(1-methyl-3-oxo-but-1-enyl)­amino-d-galactitol (**20**) shows the properties of both enamine and diketone side chains.

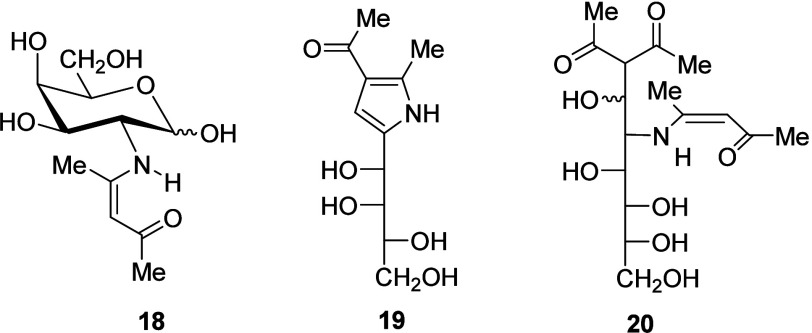



More recently, compound **21** (**6** crystallizes as its β-anomer) has been employed to synthesize 1,3,4,6-tetra-*O*-acetyl-2-amino-2-deoxy-β-d-galactopyranose (**23**) through its per-*O*-acetyl imine **22**.^16^ Likewise, the α-anomer **26** has been obtained via the enamine derivative **24**, generated by reaction of **2** with diethyl ethoxymethylene malonate, the latter being a synthetic equivalent of an aldehyde, i.e., diethyl formylmalonate (Scheme 2).^16^

**Scheme 2:**
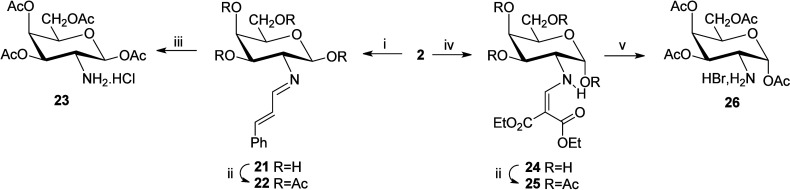
Complementary Routes to *O*-Acyl Anomers of Protected Galactosamine^a^

a Reagents: (i) PhCH = CHCHO, NaHCO_3_; (ii) Ac_2_O, C_5_H_5_N; (iii) 5 M HCl­(aq), CH_3_COCH_3_; (iv) EtOCH = C­(CO_2_Et)_2_, Et_3_N; (v) Br_2_, H_2_O, CHCl_3_.

Although the chemistry of **1** has been extensively explored,^17^ this does not apply to the imines of d-galactosamine (**2**), for which neither a comprehensive study nor the origin of mutarotational equilibria (Scheme 3) have been reported so far. We have recently shown that arylimines derived from **1** do actually exhibit a true reverse anomeric effect (RAE), despite the controversy surrounding this concept (vide infra), whose magnitude can be as intense as that of the *endo*-anomeric effect.^18a^ The existence of this RAE can now be disconnected from purely steric arguments, and involves the combined inhibition of the *exo*-anomeric effect in the α-anomer along with solvent effects.

**Scheme 3:**
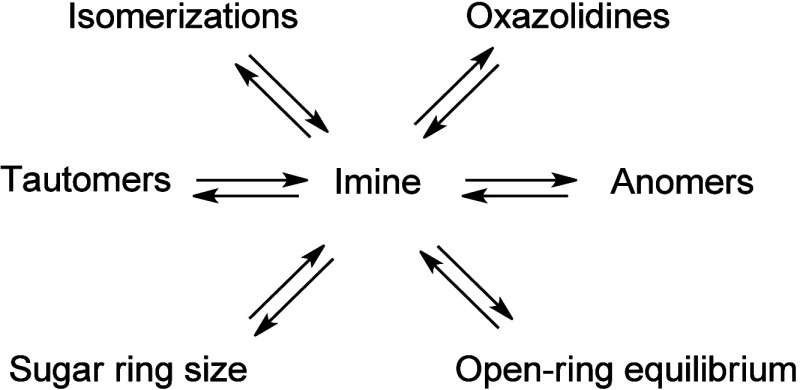
Structural Conjectures Accounting for Mutarotation in Aminosugar Imines

The present study sheds light into the mutarotation equilibria of imines derived from d-galactosamine (**2**) and the RAE shown by pyranoside and furanoside structures, which is quantified for the first time. As noted in the introductory paragraph, the thermodynamically less stable galactofuranose form is essential for the viability of some pathogens and their glycans are therefore potential targets for drug action.^19^

## Results and Discussion

### Syntheses and Structural Elucidation

The direct condensation of 2-amino-2-deoxy-α-d-galactopyranose hydrochloride (**2**) with cinnamaldehyde and other substituted benzaldehydes in aqueous solution under alkaline conditions, proceeds quickly at room temperature to afford the corresponding Schiff bases as solid products. Thus, compound **21** and a series of 2-(arylmethylidene)­amino-2-deoxy-β-d-galactopyranoses (**27–** **31**) could easily be obtained. As we shall see later, and like in the case of **6**, the crystalline material isolated for imines **9** and **10** correspond to their β-anomers **27** and **28**, respectively.

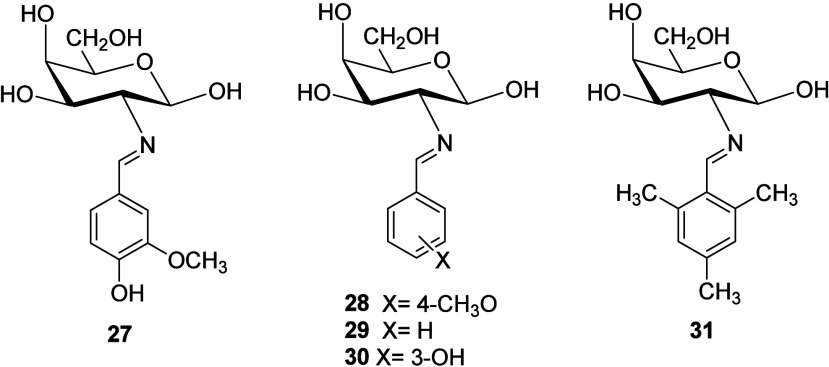



Likewise, condensations of **2** with some polyaromatic aldehydes, namely 1-naphthaldehyde, 4-methoxy-1-naphthaldehyde, 2-naphthaldehyde, 9-anthracenealdehyde and 9-phenanthrene aldehyde, gave rise to the corresponding imines **32–36**, all having β-configuration at the anomeric position.

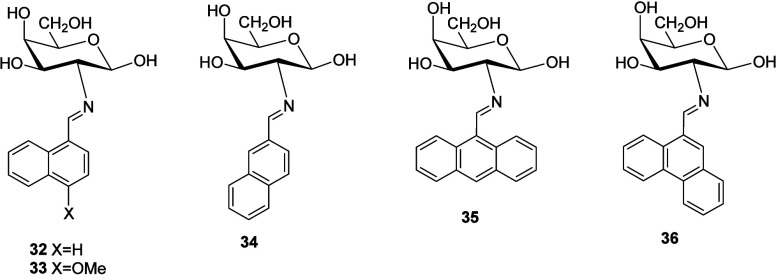



Such bulky derivatives will allow us to evaluate the influence of steric or hydrophobic effects, if any, caused by the iminic substituent on the anomeric equilibrium. In contrast, these effects would be reduced in the cinnamylidene derivative **21**, where the aromatic ring and the sugar moiety are interconnected by a vinylene spacer, the latter enabling however the propagation of substituent electronic effects.

The structures attributed to the above-mentioned Schiff bases are consistent with their spectroscopic data and elemental analyses (Tables S1–S3). That compounds **21** and **27–36** do actually exist as β-anomers can be inferred from their *J*_1,2_ coupling constants having a value of 7–8 Hz, consistent with an antiperiplanar relationship between the H-1 and H-2 protons, i.e. the OH group adopts an equatorial disposition at the anomeric carbon. Moreover, the constant between the anomeric hydrogen and carbon atoms (^1^ *J*), as measured in the coupled ^13^C NMR spectra, is ∼160 Hz. Early studies by Bock and Pedersen^20,21^ have shown the diagnostic value of that anomeric constant in pyranose rings as axial protons (β-anomers) have consistently lower values (by ∼10 Hz) than those found for α-anomers (i.e., equatorial proton). For d-galactosamine derivatives, the α-anomers show ^1^ *J*_C1–H1_ values close to 170 Hz, which decrease at ∼160 Hz in their β-counterparts. In all cases, imines **21** and **27–36** exhibit constants indicative of β-configurations at the anomeric position.^20,22^

The H-2 proton is usually the most shielded signal appearing at ∼3.1–3.3 ppm with the sole exception of the anthracenyl derivative **35**, deshielded by ∼0.3 ppm. Moreover, the iminic proton is shifted downfield by ∼0.6–1 ppm. Such variations are not observed in phenanthrene **36** which behaves like the naphthalene derivatives **32–34**. These variations should be ascribed to conformational orientations of the anthracene ring causing a significant deshielding on the H-2 and CH=N protons. The phenanthrene nucleus adopts however an analogous disposition to the one found for naphthalene rings (Figure 1).

**Figure 1:**
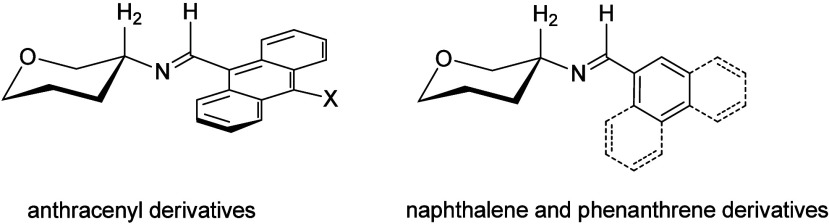
Spatial arrangements of aromatic rings in sugar imines.

The low values found for *J*_3,4_ (∼3.5 Hz) and *J*_4,5_ (0 Hz) coupling constants also agree with a d-galacto configuration plus ^4^ *C*_1_ conformation of the pyranose ring. In addition, the galactopyranose structure attributed to imines **27**, **28**, and **29** could further be confirmed by preparing the corresponding per-*O*-acetylderivatives (**37**–**** **39**), isolated in good yields, by reaction with acetic anhydride and pyridine at room temperature. NMR spectra showed the existence of another side product during the acetylation of compounds **27** and **29**. Accordingly, pure samples of **37** and **38** were obtained after fractional crystallization.

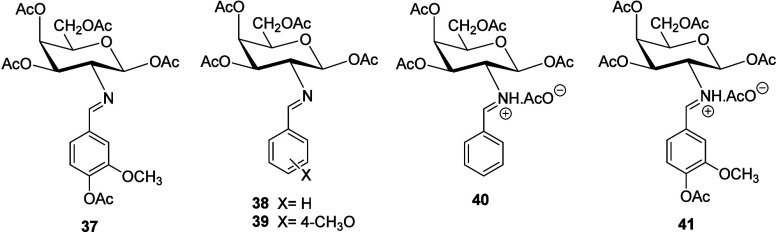



The structures attributed to **37–39** are fully supported by their elemental analyses, polarimetric and spectroscopic data. Again, for comparative purposes, Tables S4–S6 include the spectroscopic data of compound **22**.^16^ IR spectra show no absorptions above 3100 cm^–1^, which confirms the acetylation of the phenolic OH group in **27**. Significant peaks correspond to stretching bands of the carbonyl group (∼1750 cm^–1^), the C–O–C moiety of acetate groups (∼1250 cm^–1^), and the iminic C=N bond (∼1650 cm^–1^). The high values measured for *J*_1,2_ coupling constants are also consistent with β-configurations at the anomeric center, identical to that of parent imines. Moreover, the solid-state structure of **38** could be unambiguously determined by single-crystal X-ray diffraction (Figure 2 and Table S7).^23^

**Figure 2:**
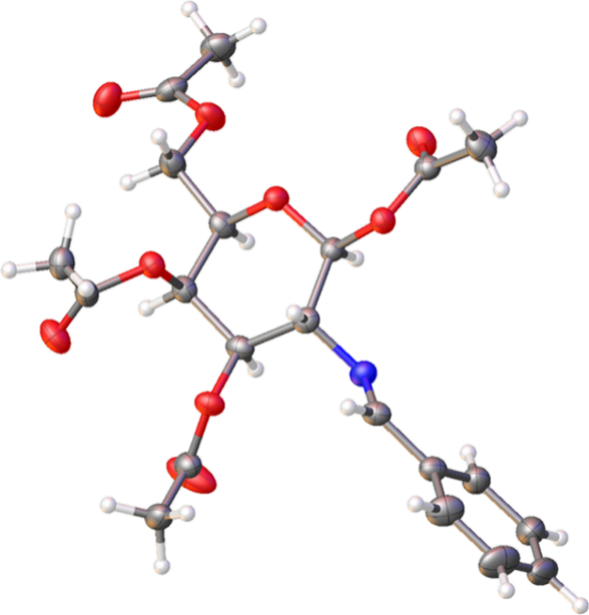
ORTEP diagram generated by X-ray diffraction analysis of benzaldehyde derivative **38**. Ellipsoids are drawn at 50% probability.

Although the synthesis of **38** was accompanied by little amounts (<5%) of its acetic acid salt **40**, the latter could be removed by crystallization. However, compound **37** was isolated together with significant amounts of its acetate **41** (65:35, respectively). However, the structures of **40** and **41** are based on their spectroscopic data. The IR spectrum of a sample containing **37** and **41** shows absorptions at ∼3260 cm^–1^, characteristic of the NH group. The corresponding ^1^H NMR spectrum (Figure 3) shows the H-1, H-2, and H-3 protons of **41** shifted more upfield than those of **37** (Δδ ∼ 0.25, 0.1, and 0.2 ppm, respectively). In stark contrast, as a result of protonation, the H-2 signal appears shifted downfield (Δδ ∼ 0.8 ppm) as quadruplet, being coupled with the H-1 and H-3 protons, and the NH signal (*J*_1,2_ ∼ *J*_2,3_ ∼ *J*_2,NH_). The latter appears as doublet at ∼5.43 ppm, whose identification is confirmed by D_2_O exchange; the shift being also concentration-dependent. Furthermore, ^13^C NMR data agree with the proposed structures. The C-1 signal resonating at 93.0 ppm in compound **41** is almost coincidental with that of **37** (93.3 ppm). The methyl signal of the acetate group resonates at ∼23.3 ppm.

**Figure 3:**
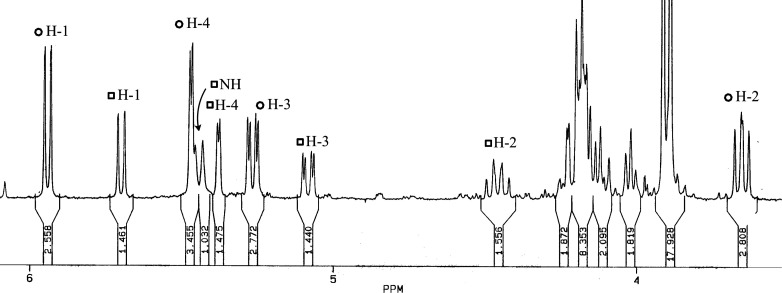
^1^H NMR spectrum of a mixture containing **37** (○) and **41** (□) (magnification of the zone between 3.5 and 6 ppm as recorded in CDCl_3_).

The coupling constants measured for **41** are also similar to those found for **37**, thereby proving the existence of a β-glycopyranose ring (*J*_1,2_ ∼ 8.7 Hz for **41**) with d-galacto configuration and ^4^ *C*_1_ conformation. In addition, the high values measured for *J*_2,NH_ (∼10 Hz) demonstrate that such hydrogen atoms exhibit an antiperiplanar relationship, which is consistent with a preferential conformation in solution similar to the one found for d-glucosamine-based imines^18^ and that of compound **38** in the solid state (Figure 2). In other words, the planar iminic functionality is nearly orthogonal (∼90°) to the mean plane of the pyranoid ring (Figure 4).

**Figure 4:**
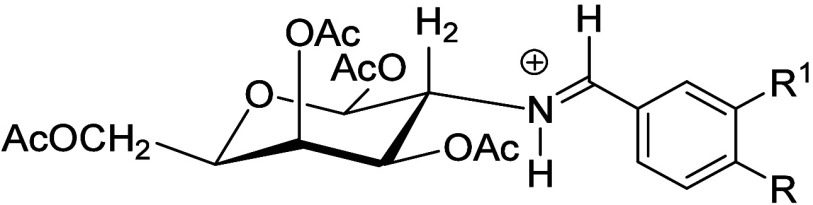
Preferential conformation adopted by compounds **40** and **41** in solution.

The per-*O*-acetylimines **42–44** could easily be obtained by conventional acetylation of compounds **33–35**. Once again, their structures are corroborated by elemental analyses and spectroscopic data (Tables S4–S6), which also confirm the structures of the starting imines. Compounds **42**–**** **44** show large *J*_1,2_ values (∼8 Hz), consistent with an equatorial arrangement of the acetate group. The anthracenyl derivative **44** exhibits rather downfield shifts for the iminic (Δδ_CH=N_ ∼ 0.8 ppm) and H-2 (Δδ_H‑2_ ∼ 0.3 ppm) protons, relative to those found in per-*O*-acetylimines **37**–**** **39**, **42**, and **43**.

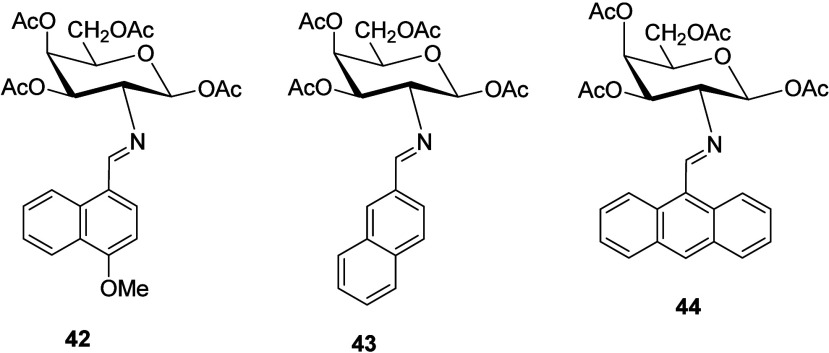



### Equilibria Involved in Mutarotation

Like other reducing sugars, the above unprotected imines derived from 2-amino-2-deoxy-d-galactose exhibit mutarotation; i.e., their optical rotation in solution changes temporarily to reach ultimately a constant equilibrium value. As outlined in Scheme 3, the causes of mutarotation are manifold and may often overlap or mutually reinforce each other. Regarding the sugar moiety, the origin of mutarotation can be associated with configurational changes at the anomeric center and/or variations in ring size. To shed light into the mutarotational phenomenon, we have performed a detailed study on the putative equilibria undergone by the Schiff bases synthesized herein with identification, whenever possible, of the intermediates involved.

Despite the fact that d-galactosamine-based imines are structurally related to those of d-glucosamine (differing by their configurations at the C-4 atom), their behavior in solution is markedly different.^18^ Thus, NMR monitoring of imines **27**–**** **36** in DMSO-*d*_6_ solution at room temperature show their equilibration with three additional products as evidenced by the presence of four signals between 7.8 and 8.5 ppm, characteristic of the corresponding iminic protons. Likewise, the region between 6.0 and 6.5 ppm shows four resonances generated by the anomeric hydroxyl groups. Peak integration indicates that two products are formed in low yield, although the other amounts up to *ca*. 25% after equilibration. As a typical example, Figure 5 shows the evolution of proton signals in the case of imine **30** on zooming the upfield resonances between 3.0 and 5.4 ppm. Compounds **a**–**** **d** are labeled in decreasing order of relative abundance.

**Figure 5:**
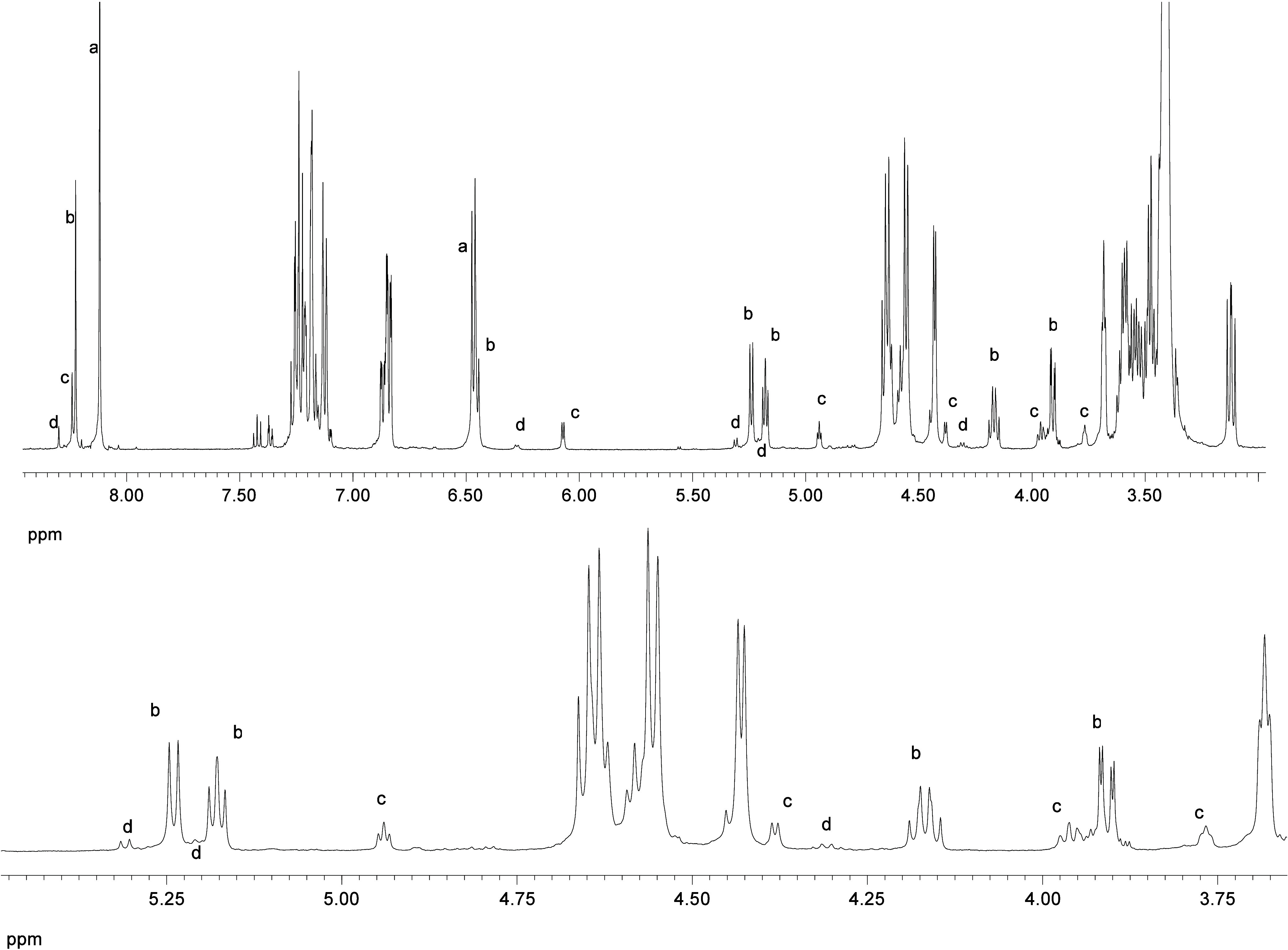
Top: ^1^H NMR spectrum of the product mixture generated from compound **30** after equilibration (signals corresponding to individual substances are labeled **a**–**** **d**). Bottom: magnification of the upfield proton zone.

Also, four signal sets can easily be identified in the corresponding ^13^C NMR spectra, as shown in Figure 6 for **29**. It is particularly noteworthy the region between 90 and 105 ppm showing the four anomeric carbon atoms. Like in Figure 5, the carbon resonances of individual substances are labeled **a**–**** **d** showing their relative abundance. As we will see later, these isomers correspond to the structures of β-pyranose, β-furanose, α-pyranose and α-furanose isomers, respectively. Table 1 gathers some selected spectroscopic data for such products arising from Schiff base **29**, as well as their relative populations determined by peak integrations of different proton signals, according to eq 1:

$$\%{\mathrm{Sb}}_{X}=100\cdot I_{X}/(I_{\beta -\mathrm{pyr}}+I_{\beta -\mathrm{fur}}+I_{\alpha -\mathrm{pyr}}+I_{\alpha -\mathrm{fur}})$$ 1

where the *I*_X_ terms correspond to the integration values of the signals corresponding to the different isomers of the Schiff base (Sb_X_). In addition, Table S8 lists all carbon signals shown in Figure 6 and their assignment.

**Table 1:** Selected NMR Data (δ, ppm; *J*, Hz), Composition and Relative Stabilities (kcal/mol) in Solution of Products Generated from 29 after Equilibration^a^

	^1^H NMR^b^			^13^C NMR^c^			
compound	CH=N	C1**–**OH	*J*_C1H–OH_	CH=N	C-1	population^d^ (%)	Δ*G*°_exp_.^e^
**a**	8.21 s	6.52	7.0	162.27	96.28	70.4	0.00
**b**	8.32 s	6.49	6.5	162.27	100.58	24.7	0.63
**c**	8.33 s	6.12	4.3	162.42	93.50	3.4	1.82
**d**	8.39 s	6.32	3.5	162.54	97.27	1.5	2.31

a In DMSO-*d*_6_.

b At 400 MHz.

c At 100 MHz.

d Proton integration.

e From eq 2.

**Figure 6:**
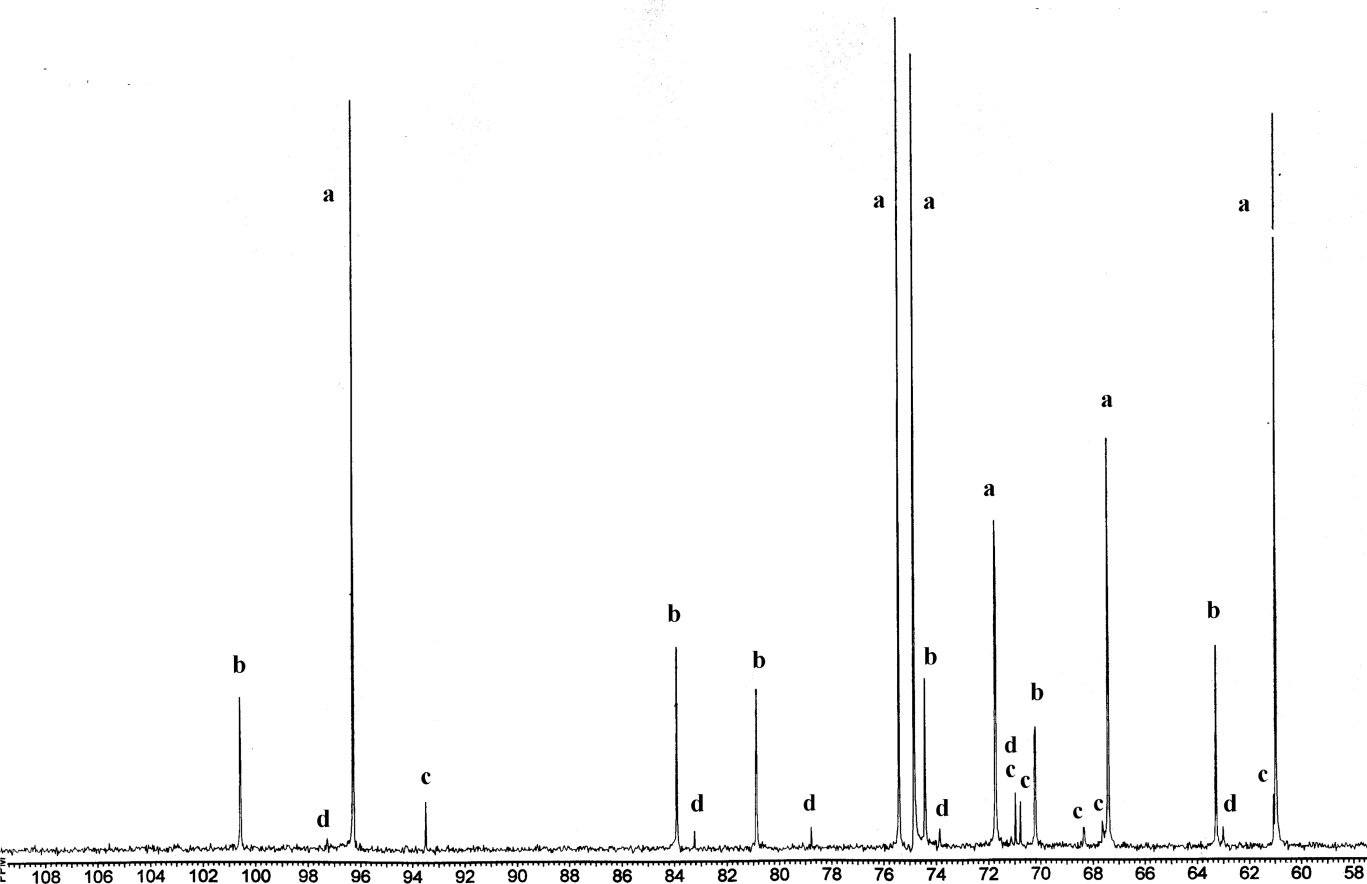
Partial view of the ^13^C resonances obtained from imine **29** at equilibrium. Products **a**–**** **d** are labeled in decreasing order of relative abundance.

As an example, the relative stability of the four pyranose/furanose and α/β isomers of **29** is given in Table 1 (last column), calculated using eq 2, where *K*_eq_ is the equilibrium constant between two given isomers and in which the values of the gas constant, *R*, and the temperature, 27 °C (300 K), have been introduced. The relative stabilities are referred to the most abundant isomer (β-pyranose).

$$\Delta G^{{}^{\circ}}\;(\mathrm{kcal}/\mathrm{mol})=-RT\ln{K_{\mathrm{eq}}}^{X}=-0.6\;\ln{K_{\mathrm{eq}}}^{X}=-0.6\;\ln([{\mathrm{Sb}}_{X}]/[\beta \text{‐}\mathrm{pyr}])$$ 2

All the imines derived from d-galactosamine exhibit a pattern similar to the one described for **29**, as exemplified by the data obtained for imine **31** after equilibration (Table S8). Likewise, the cinnamylidene derivative **21** shows similar trends, thus evidencing that this behavior does not depend on the iminic substituent, as the insertion of the vinylene linker between the imine functionality and the aromatic ring has little or no effect on the equilibrium, a fact already identified in imines derived from **1**.^18b^

As outlined in Table 2 the relative distribution of products **a**–**** **d** follows an identical order with small variations ([a] > [b] > [c] > [d]). However, less reliable data can be inferred from the peak integration in compounds **c** and **d** due to their low-yielding formation. Overall, those ratios are accurate enough and show the consistent formation of the same substances.

**Table 2:** Populations (%) Measured by Proton NMR Integration in the Equilibrium Mixtures from **21** and **28–36** ^a^

compound	21	28	29	30	31	32	33	34	35	36
**a**	70.1	58.8	70.4	63.7	73.8	63.1	65.6	63.8	81.6	79.2
**b**	25.2	32.9	24.7	27.5	21.9	27.5	25.6	28.3	12.0	18.6
**c**	3.5	5.9	3.4	6.9	3.9	7.6	6.8	6.2	4.8	2.0
**d**	1.2	2.4	1.5	1.9	0.4	1.9	2.0	1.6	1.6	0.2

a In DMSO-*d*_6_ at room temperature.

A careful spectroscopic analysis allows to identify such secondary substances formed after equilibration. Thus, compounds **a** and **c** most likely correspond to pyranose imines **45** and **46** having either β or α anomeric configurations, respectively; whereas structures **b** and **d** should be furanose rings (**47** and **48**) possessing β and α configurations, respectively, at their anomeric centers (Scheme 4).

**Scheme 4:**
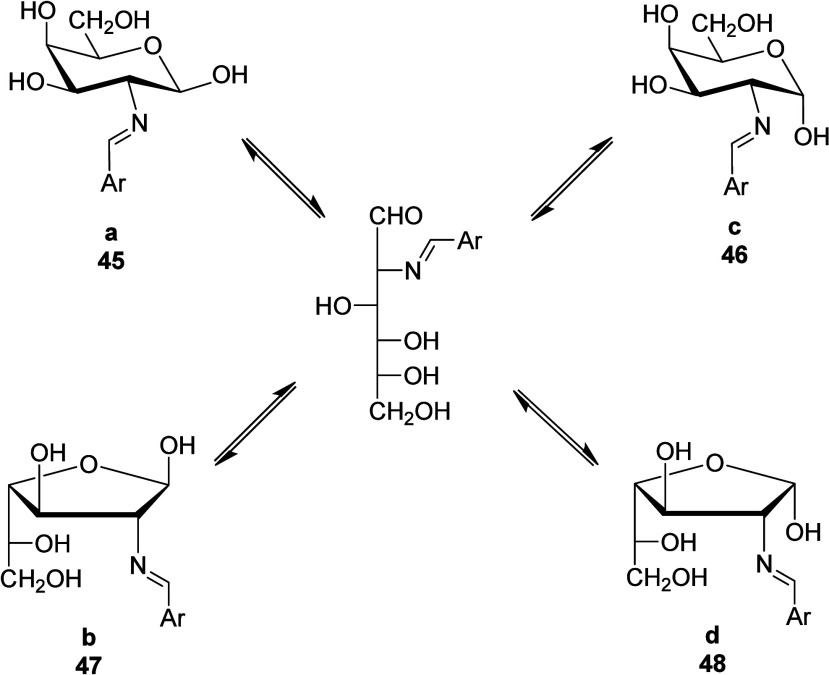
Equilibria Involving the Formation of Pyranose and Furanose Structures from d-Galactosamine-Based Imines

The structural elucidation of the major product (**a**) as 2-(arylmethylene)­amino-2-deoxy-β-d-galactopyranose (**45**) could be easily accomplished by its clear-cut spectroscopic analogy to the starting product. Compound **b** exhibits a different shift pattern, which enables the identification of such signals by both D_2_O exchange and double resonance experiments. NMR data are collected in Tables S9–S11. The treatment with D_2_O shows that doublet signals at 6.50 and 5.27 ppm correspond to OH groups, while the triplet centered at 5.21 ppm and the quadruplet at 4.19 ppm convert into doublet and triplet signals, respectively. Selective irradiation of the two OH groups evidence their couplings with signals centered at 5.21 and 4.19 ppm, which correspond to the H-1 and H-3 protons, respectively. Further DEPT and 2D ^1^H–^13^C correlation spectra enable the unambiguous assignment of all the signals.

The above structural assignment rules out the alternative formation of products generated by intramolecular cyclization of the anomeric hydroxyl to the iminic bond. This type of tautomerism in imines derived from 1,2- and 1,3-aminoalcohols is well established and has been extensively studied. Thus, imines derived from 1,2-aminoalcohols lead to five-membered rings (1,3-oxazolidines),^24^ whereas those of 1,3-aminoalcohols generate the corresponding six-membered rings (1,3-oxazines).^24f,24i,25^ In the present case, one could conjecture the formation of a bicyclic 1,3-oxazolidine, either *trans*- or *cis*-fused to the pyranoid ring (**49** and **50**, respectively), or alternatively *cis*-fused to the furanoid ring (**52**). A *trans*-fused furanoid bicycle (**51**) would be significantly strained and less stable than the aforementioned structures (Scheme 5).^18b^ Nevertheless, the presence of typical NMR resonances for the iminic bond (CH=N) at ∼8.3 and ∼162 ppm, together with the absence of signals at ∼5.30–6.00 ^24–27^ and ∼90–93 ppm,^24,26a^ which are to be expected for the H-2 and C-2 atoms of the 1,3-oxazolidine ring, rule out the formation of structures **49–52**.

**Scheme 5:**
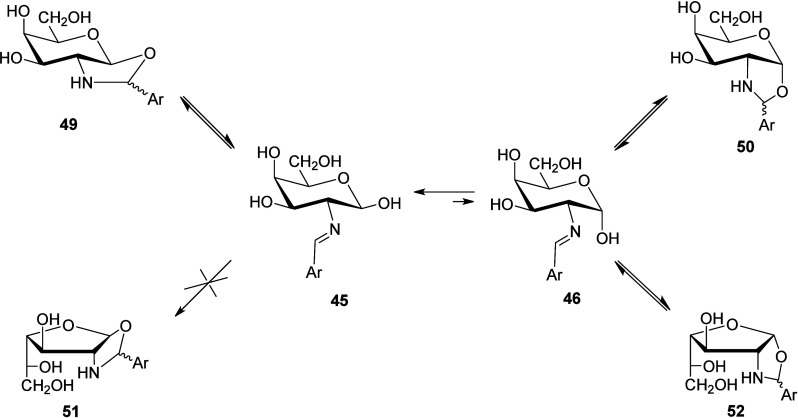
Plausible and Rejected Structures Generated by Intramolecular Cyclizations in d-Galactosamine-Based Imines

Product **b** would also be inconsistent with the pyranoid α-anomer (**46**) because the anomeric proton resonates at ∼5.2 ppm,^26a,28^ which deviates from the shift expected for α-configurations, more shielded than that of the β-anomer (∼4.7 ppm). Moreover, the coupling constant *J*_1,2_ shows values at ∼5–6 Hz, which also disagrees with that of α-anomers (∼3.5 Hz). Lastly, the large value of *J*_3,4_ (≥8 Hz) is not compatible with a *gauche* relationship between the H-3 and H-4 protons in pyranose structures of d-galacto configuration and ^4^ *C*_1_ conformation.

For compound **28**, the anomeric carbon of its byproduct **a**, resonating at ∼96.5 ppm, shows a coupling ^1^ *J*_C1,H_ of ∼157 Hz, consistent with a β-configuration.^20–22^ The anomeric signal of product **b** shows instead a value for ^1^ *J*_C1,H_ of 165 Hz. The latter would agree with an α-configuration at the anomeric center,^20,21^ albeit the chemical shift at ∼101 ppm is rather unusual. It is well-known that the anomeric carbons in both d-hexopyranoses and d-pentopyranoses, regardless of their absolute configuration, do not exceed the standard shift of 98.2 ppm.^20,29^ On the contrary, the anomeric carbons of the corresponding furanose rings may resonate beyond 100 ppm.^20^ Accordingly, the new products (**53**–**** **63**), called formerly compounds **b**, should all have the structure of 2-(arylmethylene)­amino-2-deoxy-β-d-galactofuranose (**47**) and their spectroscopic data are collected in Tables S9–S11.

A similar behavior has been previously reported for arabinose. This pentose shows the same relative configurations at the furanose ring as those of d-galactose or d-galactosamine. Arabinose does exist preferentially in a pyranose structure, equilibrated with its furanoid ring nevertheless. The latter amounts to *ca*. 3% in aqueous solution and up to 33% in DMSO.^26a,30^

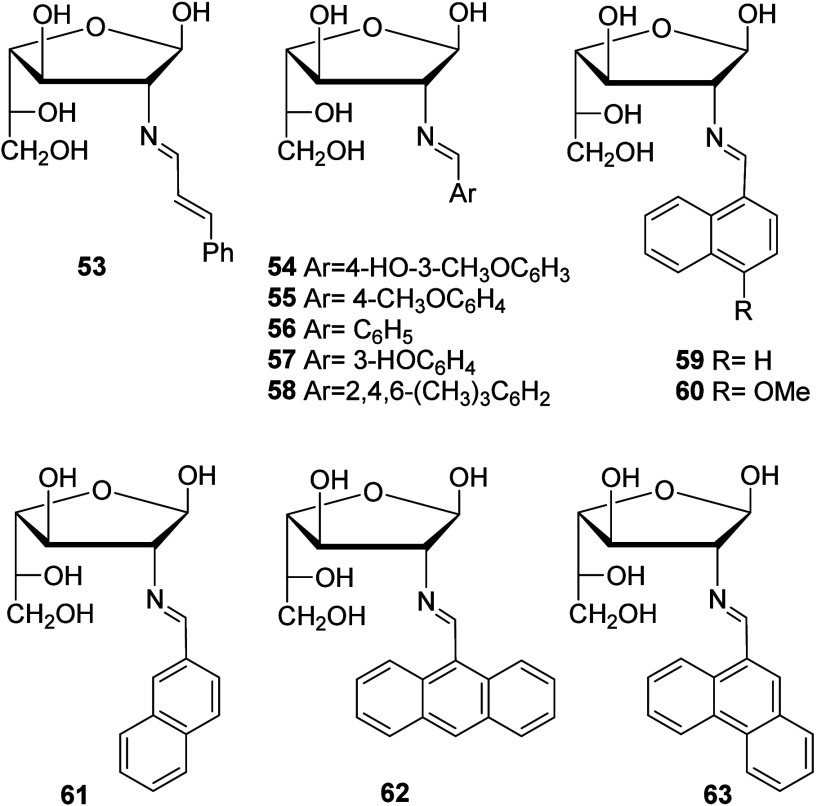



In conclusion, the spectroscopic data supporting a β-configured furanose structure for **53–63** are:

1. All carbon shifts in **53–63** are nearly coincidental with those of β-d-galactofuranose (**64**).^20^ For comparative purposes Table S11 includes ^13^C chemical shifts for compounds **64** and its α-anomer **65**.
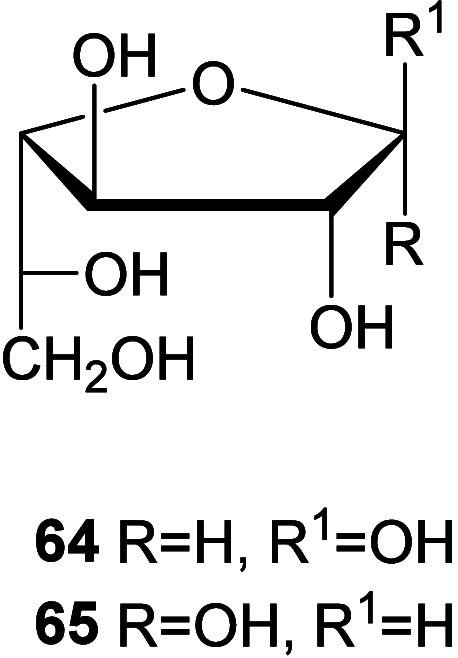

2. Multiplicity of the H-3 proton: a quadruplet that results from its couplings with H-2, H-4, and the OH signal at C-3. The magnitude of *J*_2,3_ ∼ *J*_3,4_ ∼ *J*_3,OH_ ∼ 7.0–8.5 Hz can only be compatible with a *trans* arrangement among H-2, H-3, and H-4 in furanoses of d-galacto configuration (l-arabino configuration for ring carbons).
3. The chemical shifts of the H-1 and C-1 signals in **53–63**, more deshielded than the corresponding signals in pyranoid isomers **21**, **27–36**, also agree with the rule of thumb stating that the anomeric signals of glycopyranose structures resonate invariably more upfield than those of the corresponding glycofuranose ones.^20,26a^

Only a few proton signals could be identified in the case of products **c**, the third in relative abundance, albeit comparisons of ^13^C chemical shifts with those of model compounds suggest structures of 2-(arylmethylene)­amino-2-deoxy-α-d-galactopyranoses (**66–76**). Table S12 collects the carbon resonances for such model compounds, namely both α- and β-anomers of 2-acetamido-2-deoxy-d-galactopyranose (**77** and **78**, respectively).^20^

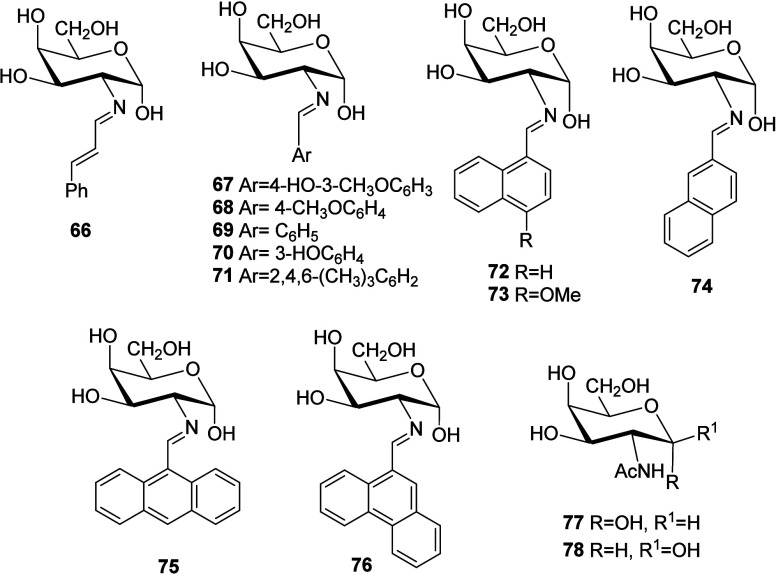



The minor products **d** have been identified as 2-(arylmethylene)­amino-2-deoxy-α-d-galactofuranoses (**79–89**). Again, Table S12 shows, for comparative purposes, the carbon resonances of some compounds along with those of the α-anomer of d-galactofuranose (**65**).

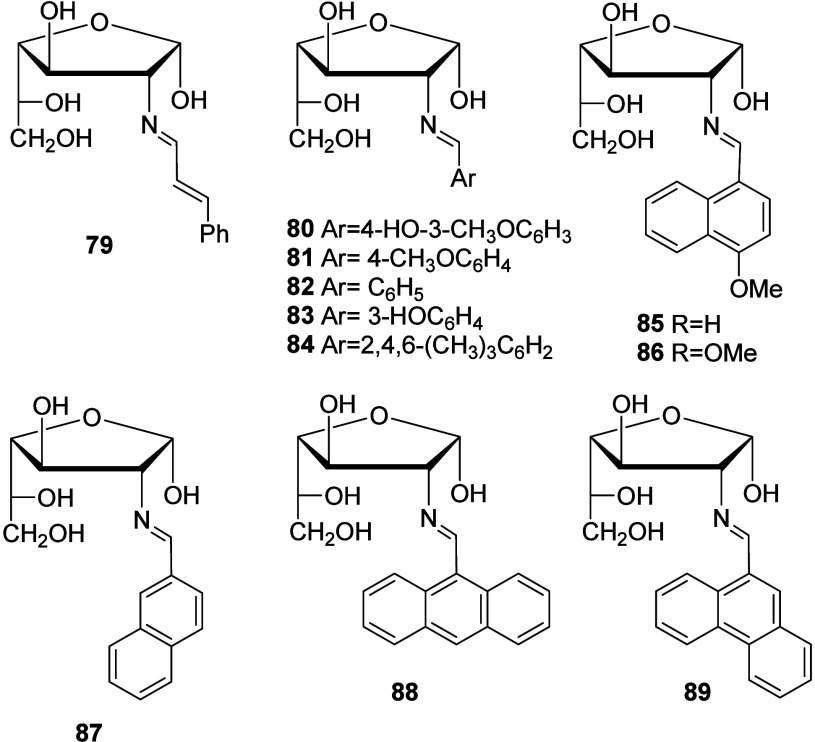



Overall, the behavior shown by imines derived from d-galactosamine contrasts with the one found in their d-glucosamine counterparts,^18^ which are stable enough in solution and only equilibrate between α- and β-anomers of pyranoid structures.

### Anomeric Equilibria in Imines and Enamines Derived from d-Galactosamine

Due to the anomeric effect,^31,32^ both d-glucose and d-galactose exhibit a greater population (by three times higher) than expected for the α-anomer in aqueous solution,^33^ which increases further in less polar solvents such as DMSO.^34^ This trend is also observed for the corresponding hydrochlorides of α-d-glucosamine (**1**), α-d-galactosamine (**2**), and 2-amino-2-deoxy-α-d-glycero-l-gluco-heptopyranose (**90**),^35^ although apparently the anomeric effect is almost negligible in their imines derived from aromatic aldehydes,^18^ because the population of α-anomers in DMSO decreases to ≤12% in both pyranose and furanose structures (Table 3).

**Table 3:** Equilibrium Populations (%) of Pyranose Anomers for d-Galactosamine Imines^a^

	compound												
anomer	21	28	29	30	31	32	33	34	35	36	91	92	95
α	3.5	5.9	3.4	6.9	3.9	7.6	6.8	6.2	4.8	2.0	35.7	31.8	82.0
β	70.1	58.8	70.4	63.7	73.8	63.1	65.6	63.8	81.6	79.2	41.8	60.5	18.0
α^b^	4.8	9.1	4.6	9.8	5.0	10.7	9.4	8.9	5.6	2.5	46.1	34.5	82.0
β^b^	95.2	90.9	95.4	90.2	95.0	89.3	90.6	91.1	94.4	97.5	53.9	65.5	18.0
Δ*G*°_an_^pyr^ ^c^	–1.79	–1.38	–1.82	–1.33	–1.77	–1.27	–1.36	–1.40	–1.69	–2.20	–0.09	–0.38	+0.91
*E*_an_^pyr^ ^d^ ^,^ ^e^	–0.54	–0.13	–0.57	–0.08	–0.52	–0.02	–0.11	–0.15	–0.44	–0.95	+1.15	+0.87	+2.16
Δ*G*°_rae_^pyr^ ^f^ ^,^ ^g^	1.73	1.32	1.76	1.27	1.71	1.21	1.30	1.34	1.63	2.14	–0.04	–0.32	–0.97

a In DMSO-*d*_6_.

b α-Pyranose + β-pyranose = 100%.

c From eq 3, in kcal/mol.

d From eq 8, in kcal/mol.

e Anomeric stabilization referred to cyclohexanol.

f From eq 9, in kcal/mol.

g Reverse anomeric stabilization referred to **91**.

The preferential stabilization^36^ of pyranose sugar rings when they contain an axial electronegative substituent at C1 is contrary to expectations based on considerations of steric or solvation factors.^37^ Three hypotheses have been frequently invoked,^32,38,39^ even if not mutually exclusive, accounting for the anomeric effect: (a) dipole interaction,^31a^ i.e., the α-anomeric preference would be due to the repulsion between the dipoles of the anomeric hydroxyl and those of the endocyclic oxygen; (b) hyperconjugation or antiperiplanar lone pair hypothesis model (ALPH), based on the n → σ* interaction, i.e., stabilizing interaction of the axial electron pair on the endocyclic oxygen (nO, HOMO) with the empty orbital σ* of the C–OH bond of the α-anomer (LUMO),^40^ and (c) electron pair repulsion model (n → n interaction): strong destabilization generated by the interaction between orbitals filled of two pairs of solitary electrons, spatially very close.^41^

However, Mo and associates reported some computational evidence ruling out hyperconjugative interactions as key factors responsible for the anomeric effect,^42^ which is better interpreted in terms of electrostatic interactions. The underlying physical origin(s) of this phenomenon remains unclear^43,44^ and the anomeric effect can be attributed to a combination of steric, resonance, hyperconjugation, inductive, hydrogen bonding, electrostatic interaction, and solvation effects, all influenced in addition by the theoretical level of calculations.^45,46^

On the other hand, it has been suggested that amino and alkylamino substituents in anomeric positions, either neutral or protonated, can show a reverse anomeric effect.^47,48^ The existence of the reverse anomeric effect has been questioned and debated. The greater equatorial preference has been attributed to an accentuation of the steric effects, as illustrated by the protonation of alkylamino or imidazole substituents.^49–51^ NMR and X-ray crystallography studies of a 1,3-dioxolane derivative, which carries a bulky quaternary quinuclidine substituent on C-2, has revealed the absence of reverse anomeric stabilization.^52^

Most of the work carried out on the anomeric effect has been limited to investigate the influence of different substitution patterns on the anomeric configuration. Enamines formed by the condensation of β-dicarbonyl compounds and their synthetic equivalents with **1** and **2** always adopt the α-anomeric configuration; that is, the anomeric hydroxyl is arranged axially.^53^ In stark contrast, Schiff bases resulting from the condensation of these amino sugars with simple aromatic aldehydes adopt the opposite configuration.^16,18,54–58^

In the conformation adopted by the imine group with respect to the pyranose ring, the pair of unshared electrons of the nitrogen is arranged approximately parallel to the axial bonds of the ring. In this particular arrangement the electron pair of the iminic nitrogen could then exert a stereoelectronic effect that disfavors the axial anomer (α). Actually, for this anomer one can envisage a conformation showing a possible repulsive interaction between the electron pair of the nitrogen and one of the lone pairs on the oxygen at the anomeric hydroxyl (Figure 7a). However, theoretical calculations indicate that this effect, if any, should be negligible or does not exist at all.^18a^

**Figure 7:**
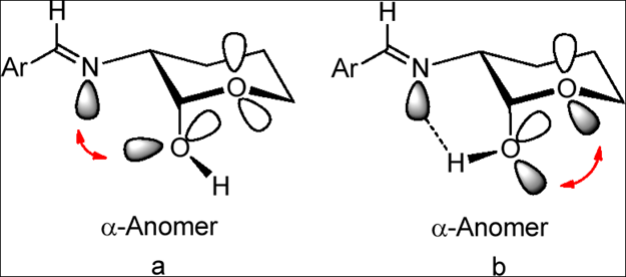
Stereoelectronic interactions and hydrogen bonding in the axial anomer of sugar imines.

In the absence of any other factor, the *exo*-anomeric interaction is the most dominant, even in the α-anomer. Nevertheless, intramolecular hydrogen bonding can take place between the imine nitrogen at C2 and the anomeric hydroxyl, which changes the orientation of the lone pairs of the latter, thereby decreasing or eliminating the *exo*-anomeric effect on the α-anomer (Figure 7b). Formation of such a bonding in the β-anomer is hampered by the conformational rigidity of the arylimino group. The destabilization that these interactions cause in the axial anomer translates into a significant increase in the population of the equatorial anomer. Globally considered, this fact could be interpreted as an inversion of the anomeric population, that is, a genuine reverse anomeric effect (RAE).^18^

### Reverse Anomeric Effect in Pyranose Structures

In line with the preceding structural discussion, it is now quite obvious that the structural complexity of imines derived from d-galactosamine in solution stems from a superposition of anomeric and ring (pyranose versus furanose) equilibria (Table 2). Despite this fact, our experimental results point to a prevalence of the β-pyranose structure over the α-form, owing to the above-mentioned RAE that destabilizes the α-anomer, which could be roughly estimated. Table 3 collects the percentages of pyranoses (both α and β), in equilibrium with the corresponding α- or β-furanoses, for d-galactosamine Schiff bases. This Table also includes the percentages referred to pyranoid forms only (i.e., α-pyranose + β-pyranose = 100%), Δ*G*°_an_^pyr^, the anomeric stabilization *E*_an_^pyr^, and the RAE Δ*G*°_rae_^pyr^.

Δ*G*°_an_^pyr^ is the observed free energy change for the balance between the axial and equatorial disposition (eq 3) in pyranoid imines: α-anomer ⇄ β-anomer

$$\Delta {{G^{o}}_{\mathrm{an}}}^{\mathrm{pyr}}=-RT\;\ln([\beta \text{‐}\mathrm{pyranose}]/[\alpha \text{‐}\mathrm{pyranose}])=-RT\;\ln{K_{\mathrm{an}}}^{\mathrm{pyr}}$$ 3

The anomeric stabilization *E*_an_^pyr^, defined as the nonsteric stabilization of the axial conformer, can be quantified by correcting the axial preference of a substituent (Δ*G*°_an_^pyr^) with the steric effects that favor an equatorial arrangement (Δ*G* ^o^_steric_):

$${E_{\mathrm{an}}}^{\mathrm{pyr}}=\Delta {{G^{o}}_{\mathrm{an}}}^{\mathrm{pyr}}-\Delta {G^{o}}_{\mathrm{steric}}$$ 4

The steric component is measured on model compounds in nonanomeric systems. For pyranose sugar derivatives, the *A*_X_ values of cyclohexane are generally used. The parameter *A* for a substituent X is given by *A*_X_ = −Δ*G*°_steric_, where Δ*G*°_steric_ measures the variation of free energy in the axial ⇄ equatorial equilibrium for cyclohexane carrying the substituent X; that is, it measures the steric preference for the equatorial arrangement of a given substituent (eq 5).

$$\Delta {G^{o}}_{\mathrm{steric}}=-RT\;\ln([\mathrm{equatorial}]/[\mathrm{axial}])=-A_{X}$$ 5

When one varies the substituents at nonanomeric positions *A*_X_ = *A*_OH_ and the quantitative relationship for the anomeric hydroxyl group can be expressed by eq 6:

$${E_{\mathrm{an}}}^{\mathrm{pyr}}=-RT\;\ln{K_{\mathrm{an}}}^{\mathrm{pyr}}+A_{\mathrm{OH}}$$ 6

Thus, the A_OH_ value for the hydroxyl group in aqueous solution is 1.25 kcal/mol and corresponds to 89%-predominance of cyclohexanol with the equatorial hydroxyl^59,60^ (eq 7).

$$A_{\mathrm{OH}}=-\Delta {G^{o}}_{\mathrm{steric}}=0.002\times 298\times \ln(89/11)=1.25\;\mathrm{kcal}/\mathrm{mol}$$ 7

The resulting eq 8 can then be employed for determining *E*_an_^pyr^:

$${E_{\mathrm{an}}}^{\mathrm{pyr}}\;(\mathrm{kcal}/\mathrm{mol})=-0.6\;\ln{K_{\mathrm{an}}}^{\mathrm{pyr}}+1.25$$ 8

Data in Table 3 show that the anomeric stabilization values lie between −0.02 and −0.95 kcal/mol. They have a negative value, indicating that the β-anomer is widely favored in the anomeric equilibrium.

The difference between the anomeric effect (Δ*G*°_an_^pyr^) in absence of the imine group and the anomeric stabilization in imines (Δ*G*°_im_^pyr^) allow us to estimate the reverse anomeric effect (Δ*G*°_rae_^pyr^).^18^ If we accept as quantitative estimation of the anomeric effect the value of *E*_an_^pyr^ shown by 2-deoxy-d-galactose (2-deoxy-d-*lyxo*-hexopyranose, **91**) in D_2_O for the ratio 40% α-anomer: 44% β-anomer at 31 °C (*E*_an_^**91**^ = −0.6 × ln­[44/40]) = 1.19 kcal/mol),^61^ then

$$\Delta {{G^{o}}_{\mathrm{rae}}}^{\mathrm{pyr}}=\Delta {{G^{o}}_{\mathrm{an}}}^{91}-\Delta {{G^{o}}_{\mathrm{an}}}^{\mathrm{pyr}}={E_{\mathrm{an}}}^{91}-{E_{\mathrm{an}}}^{\mathrm{pyr}}=1.19-{E_{\mathrm{an}}}^{\mathrm{pyr}}$$ 9

Clearly the extent of the reverse anomeric effect is high enough, between 1.21 and 2.14 kcal/mol, which exceeds that of the *endo*-anomeric effect (0.96–1.91 kcal/mol).^38c^ For comparative purposes, Table 3 includes the data for d-galactopyranose (**92**).

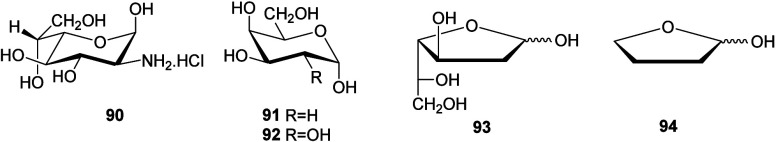



### Reverse Anomeric Effect in Furanose Structures

A similar analysis can be conducted on the corresponding galactofuranose structures, with data collected in Table 4, which also shows the percentages relative solely to the equilibrium between α- and β-furanoid anomers (i.e., α-furanose + β-furanose = 100%) and Δ*G*°_an_^fur^ values (eq 10):

$$\Delta {{G^{o}}_{\mathrm{an}}}^{\mathrm{fur}}=-RT\;\ln([\beta \text{‐}\mathrm{furanose}]/[\alpha \text{‐}\mathrm{furanose}])=-RT\;\ln{K_{\mathrm{an}}}^{\mathrm{fur}}$$ 10

**Table 4:** Equilibrium Populations (%) of Furanose Anomers for d-Galactosamine Imines^a^

	compound										
anomer	53	55	56	57	58	59	60	61	62	63	93
α	1.2	2.4	1.5	1.9	0.4	1.9	2.0	1.6	1.6	0.2	3.1
β	25.2	32.9	24.7	27.5	21.9	27.5	25.6	28.3	12.0	18.6	4.6
α^b^	4.5	6.8	5.7	6.5	1.8	6.5	7.2	5.4	11.8	1.1	40.3
β^b^	95.5	93.2	94.3	93.5	98.2	93.5	92.8	94.6	88.2	98.9	59.7
Δ*G*°_an_^fur^ ^c^	–1.83	–1.57	–1.68	–1.60	–2.40	–1.60	–1.53	–1.72	–1.21	–2.70	–0.24
*A*_OH_^fur^ ^d^	1.97	1.87	1.98	1.86	1.96	1.84	1.87	1.88	1.95	2.07	1.63
*E*_an_^fur^ ^e^ ^,^ ^f^	–0.28	–0.02	–0.13	–0.05	–0.85	–0.05	0.02	–0.17	0.34	–1.20	1.79
Δ*G*°_rae_^fur^ ^g^ ^,^ ^h^	1.83	1.57	1.68	1.60	2.40	1.60	1.53	1.72	1.21	2.70	0.24

a In DMSO-*d*_6_.

b α-Furanose + β-furanose = 100%.

c From eq 10, in kcal/mol.

d From eq 16, in kcal/mol.

e From eq 17, in kcal/mol.

f Anomeric stabilization referred to cyclopentanol.

g From eq 18, in kcal/mol.

h Reverse anomeric stabilization referred to **93**.

Such data indicate again that β-furanose structures are more abundant than their α-furanose counterparts, thus suggesting the same stabilizing steric and/or stereoelectronic effects as those present in pyranoid rings. On the other hand, the steric effect in β-anomers arising from eclipsing *cis*-vicinal groups in five-membered rings cannot be neglected.

In this case we were unable to calculate the anomeric stabilization parameter *E*_an_ because the *A*_OH_ value for cyclopentanol is not available. However, recently, Gaweda and Plazinski have shown by calculations that the *endo*-anomeric effect in furanose derivatives exhibit a mean increase of the *endo*-anomeric effect of 0.64 kcal/mol (2.7 kJ/mol) compared to structurally analogous pyranosides. In the specific case of the hydroxyl group, this value is 0.36 kcal/mol (1.5 kJ/mol)^62^ and accordingly

$${E_{\mathrm{an}}}^{\mathrm{fur}}={E_{\mathrm{an}}}^{\mathrm{pyr}}+0.36$$ 11

By applying this result to furanose imines in Table 4 (from data collected in Table 3), *E*_an_^fur^ will take values between approximately 0.34 and ***–*** 0.59 kcal/mol.

A plot of the percentages of furanose structures as a function of the corresponding percentages of pyranose structures for a given anomer (Table 3) leads to an acceptable correlation. This representation for the β-anomers (eq 12 and Figure S1) indicates that similar effects occur in both β-pyranose and β-furanose anomers.

[β‐fur(%)]=0.72[β‐pyr(%)]+28.53(r=0.915) 12

Likewise, when the values of Δ*G*°_an_ for the furanose imines (Δ*G*°_an_^fur^) are plotted as a function of those of the corresponding pyranose imines (Δ*G*°_an_^pyr^), a good linear correlation could be obtained as well (eq 13 and Figure 8). The value larger than 1 of the slope (1.24) demonstrates that furanose imines show a RAE greater than that shown by their pyranose analogs.

$$\Delta {{G^{o}}_{\mathrm{an}}}^{\mathrm{fur}}=1.24\Delta {{G^{o}}_{\mathrm{an}}}^{\mathrm{pyr}}+0.07(r=0.921)$$ 13

**Figure 8:**
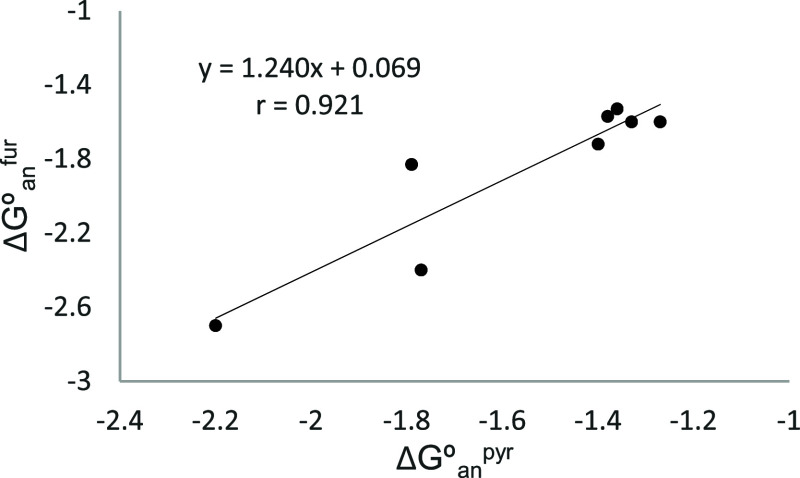
Representation of Δ*G*°_an_^fur^ versus Δ*G*°_an_^pyr^ for β-anomers of **21**, **28**, **30–** **34**, and **36**.

In a similar way to the procedure performed with pyranose imines, the anomeric stabilization *E*_an_^fur^ can be formulated according to eq 14:

$${E_{\mathrm{an}}}^{\mathrm{fur}}=\Delta {{G^{o}}_{\mathrm{an}}}^{\mathrm{fur}}-\Delta {{G^{o}}_{\mathrm{steric}}}^{\mathrm{cyclp}}=\Delta {{G^{o}}_{\mathrm{an}}}^{\mathrm{fur}}+{A_{\mathrm{OH}}}^{\mathrm{cyclp}}$$ 14

where *A*_OH_^cyclp^ measures the steric preference for the pseudo-equatorial arrangement of the hydroxyl group in hydroxycyclopentane (eq 15):

$${A_{\mathrm{OH}}}^{\mathrm{cyclp}}=-\Delta {{G^{o}}_{\mathrm{steric}}}^{\mathrm{cyclp}}=RT\;\ln([\mathrm{pseudo}\text{‐}\mathrm{equatorial}]/[\mathrm{pseudo}\text{‐}\mathrm{axial}])$$ 15

It is not possible to measure *A*_OH_^fur^ experimentally, because the barriers to interconversion between the different conformations of hydroxycyclopentane derivatives are very low (<1 kcal/mol).^62^ However, a good approach can be inferred from eqs 6, 11, 13, and 14:

$${E_{\mathrm{an}}}^{\mathrm{fur}}-{A_{\mathrm{OH}}}^{\mathrm{cyclp}}={E_{\mathrm{an}}}^{\mathrm{pyr}}+0.36-{A_{\mathrm{OH}}}^{\mathrm{cyclp}}=1.24({E_{\mathrm{an}}}^{\mathrm{pyr}}-{A_{\mathrm{OH}}}^{\mathrm{cyclh}})+0.07$$ 16

and accordingly

$${A_{\mathrm{OH}}}^{\mathrm{cyclp}}=-0.24{E_{\mathrm{an}}}^{\mathrm{pyr}}+1.84=0.14\;\ln{K_{\mathrm{an}}}^{\mathrm{pyr}}+1.54$$ 17

The apparent value of *A*_OH_^cyclp^ depends on the value of *E*_an_^pyr^, namely, the substituent attached to C-2. Table 4 shows such results averaging to 1.93 kcal/mol. However, these values may be influenced by both electronic and steric effects produced by arylimino groups located at C-2. To avoid this, we used the published data for the equilibrium composition of 2-deoxy-d-lyxo-hexopyranose (**91**) (α-pyranose:β-pyranose 40:44) with *A*_OH_^cyclp^ taking the value 1.55 kcal/mol. From eq 14 the following expression can be obtained:

$${E_{\mathrm{an}}}^{\mathrm{fur}}=\Delta {{G^{o}}_{\mathrm{an}}}^{\mathrm{fur}}+1.55=-0.6\;\ln{K_{\mathrm{an}}}^{\mathrm{fur}}+1.55$$ 18

The calculated values, using eq 18, of *E*_an_^fur^ lie between 0.34 and −1.20 kcal/mol, similar to those obtained by means of eq 11. The value of the anomeric effect for the equilibrium of 2-deoxy-d-lyxo-hexofuranose (**93**) (α-furanose:β-furanose ratio: 8:8) is 1.55 kcal/mol.^61^ This value is very close to that calculated for the anomeric effect in 2-hydroxytetrahydrofuran (**94**), which is 1.43 kcal/mol (6.0 kJ/mol).^62^ The magnitude of RAE will be given by eq 19:

$$\Delta {{G^{o}}_{\mathrm{rae}}}^{\mathrm{fur}}=\Delta {{G^{o}}_{\mathrm{an}}}^{93}-\Delta {{G^{o}}_{\mathrm{an}}}^{\mathrm{fur}}={E_{\mathrm{an}}}^{93}-{E_{\mathrm{an}}}^{\mathrm{fur}}=1.55-{E_{\mathrm{an}}}^{\mathrm{fur}}=-\Delta {{G^{o}}_{\mathrm{an}}}^{\mathrm{fur}}$$ 19

In general, the calculated values of Δ*G*°_rae_^fur^ are somewhat higher than those of Δ*G*°_rae_^pyr^, ranging between 1.21 and 2.70 kcal/mol. These results indicate that the RAE is greater for furanoid imines than for their pyranoid isomers, and overcomes the extent of the *endo*-anomeric effect. In furanoses, this effect is typically larger than in pyranoses.^62^

Therefore, the experimental data support the existence of a RAE in imines derived from 2-amino-2-deoxyaldoses, capable of counterbalancing largely the anomeric effect. The opposite effect is observed when the Schiff base shows an enamine structure, as evidenced by compounds **24** and **95** ^9b^ (α-anomer >80%); their lone pair is heavily involved in electron delocalization with the double bond, thus inhibiting completely or partially the formation of the hydrogen bond between the anomeric hydroxyl and the nitrogen atom. Again, *endo*- and *exo*-anomeric effects make the α-anomer more stable and hence the most populated isomer (for comparative assessments, the anomeric populations of **95** have been included in Table 3). Moreover, protonation of the imine nitrogen restores the presence of the *exo*-anomeric effect, as evidenced by hydrochloride **96** in which the α-anomer dominates again in the equilibrium mixture (α-anomer: 84.8%).^18^

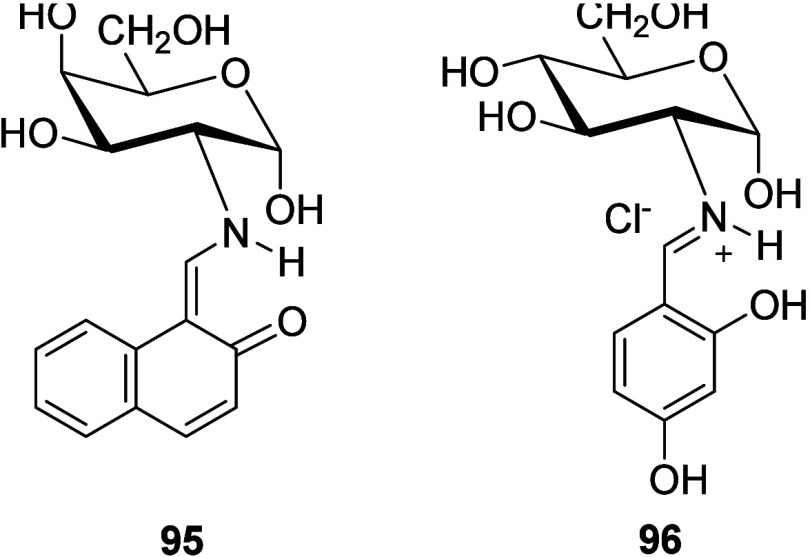



### Theoretical Calculations

In the search for a rationale accounting for the above-detailed facts, we performed additional computational studies on the relative stability of all species involved in mutarotational equilibria. DFT calculations with inclusion of solvation effects (SMD method),^63^ as implemented in the Gaussian09 package,^64^ with the 6-31G­(d,p), 6-311G­(d,p),^65^ and def2-TZVP valence-triple-ζ basis^66^ sets, optimizing the geometries in gas phase with the B3LYP^67^ and M06-2X^68^ density functional methods^69^ without any geometric restriction (see Experimental Section), were carried out and will be described herein. In addition, electronic structures were analyzed by the natural bond orbital (NBO) method.^70^ In order to alleviate the computational cost (3^6^ = 729 possible conformations for each anomer), these calculations have been conducted on a structurally simple imine (**29**) and Scheme 6 depicts the computed species.

**Scheme 6:**
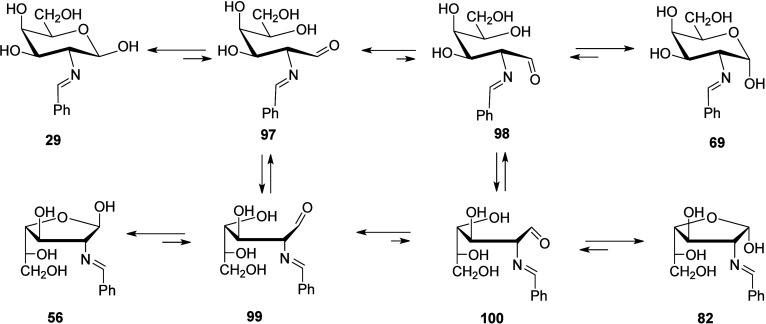
Generation of Pyranose and Furanose Structures in Mutarotational Equilibria of d-Galactosamine Imines

Because of hydroxyl groups may participate in both inter- and intramolecular hydrogen bonding with the endocylic oxygen and the iminic nitrogen, these possibilities have been taken into account. Thus, we have considered the hydroxyl arrangements **a–** **c** for compounds **29** and **69**, being the most stable structures those forming intramolecular hydrogen bonds (Figure 9). Table 5 collect some computed data on relative stability (for extended data, see Supporting Information and Table S13).

**Figure 9:**
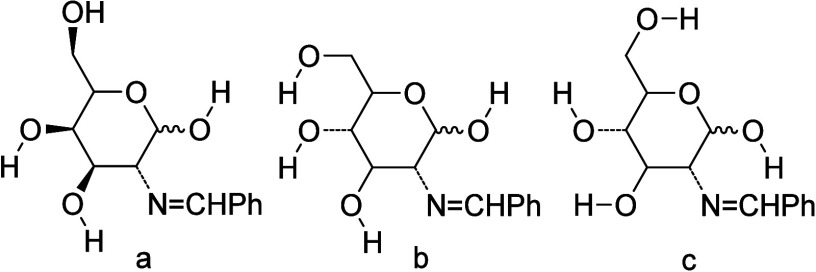
H-bonded structural arrangements (**a**–**** **c**) for phenylimino derivatives at C-2.

**Table 5:** Relative Stabilities (kcal/mol) of All Species Involved in the Mutarotation of 29

			gas phase^b^		DMSO^b^	
	entry	conformer^a^	Δ*E*	Δ*G*_r_	Δ*E*	Δ*G*_r_
**29**	b	^4^ *C*_1_	2.26	1.92	0.67	0.31
	c	^4^ *C*_1_	2.42	1.85	0.07	0.34
**69**	b	^4^ *C*_1_	1.32	1.31	0.40	0.68
	c	^4^ *C*_1_	0.00	0.00	0.00	0.00
**56**	c	^2^ *T*_1_	6.65	4.89	5.89	3.14
	d	^1^ *T*_2_	1.71	0.63	1.16	1.26
	e	^1^ *T*_2_	8.15	7.07	7.07	6.01
**82**	c	^2^ *T*_1_	2.20	1.12	1.94	1.36
	d	^1^ *T*_2_	4.96	3.72	3.09	3.10

a Ring conformation.

b M06-2X/def2-TZVP.

All the conformational dispositions adopted by hydroxyl groups in aldehyde structures **97–** **100** leading to the cyclic arrangements of **29**, **56**, **69**, and **82** showed higher energies than those of acyclic ones (Tables 6 and S14), presumably due to the enhanced stability of single C–O bonds relative to the π bond of carbonyl groups.

**Table 6:** Relative Stabilities (kcal/mol) of Acyclic Species Involved in the Mutarotation of 29^a^ ^,^ ^b^

	gas phase		DMSO	
	Δ*E*	Δ*G*_r_	Δ*E*	Δ*G*_r_
**97**	17.08	13.44	16.13	12.43
**98**	10.71	8.22	11.16	8.24
**99**	14.53	10.21	15.33	11.96
**100**	14.59	10.47	12.77	9.09

a Energies refer to those of **69c** (Table 5).

b M06-2X/def2-TZVP.

Entries **b** and **c** in cyclic structures **29**, **56**, **69**, and **82** refer to staggered conformations of the hydroxymethylene OH group at C-6. In pyranose species **b**, that OH group is involved in hydrogen bonding with the OH group at C-4. On the contrary, hydrogen bonding takes place with the endocyclic oxygen in species **c** (Figure 9). The latter appears to be the most stable form in DMSO solution for both anomers. Figure 10 shows the most stable conformations encountered for the four anomers through computational analyses.

**Figure 10:**
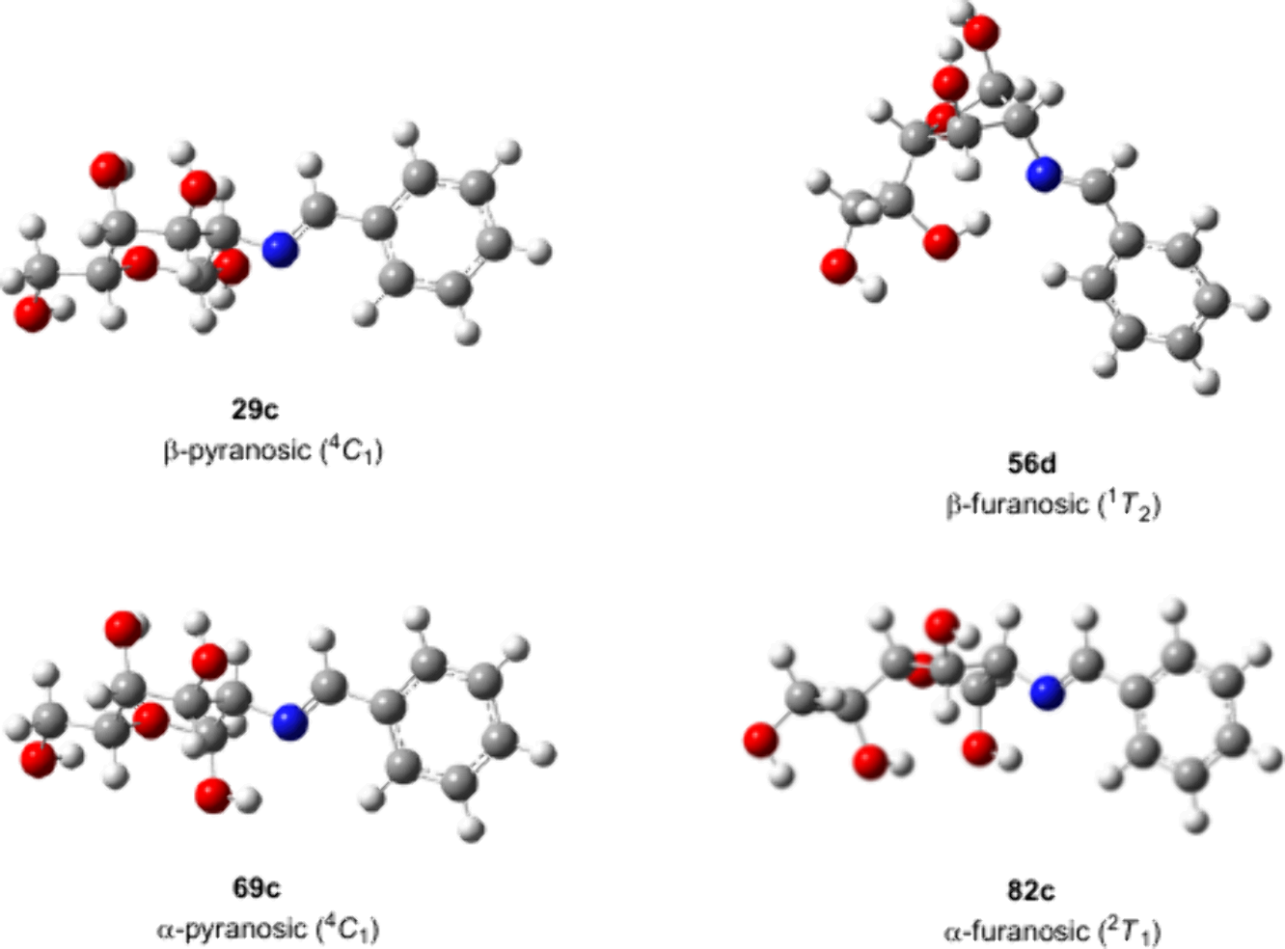
Preferential conformations adopted by **29c**, **56d**, **69c**, and **82c** in DMSO solution.

Calculations in Table 5 show a greater stability of the pyranoid α-anomer over the β-configuration (ΔΔ*G*° ∼ 0.6 kcal/mol at the 6-311G­(d,p) level or ∼0.3 kcal/mol using def2-TZVP), thus accounting for a prevalence of the former (*ca*. 70 or 60%) at room temperature. However, this disagrees with the experiment that shows a preferential formation of pyranoid β-anomers. This mismatch should clearly be attributed to the SMD method’s inaccuracy, which does not consider explicitly solvent molecules competing with intramolecular hydrogen bonds (OH groups) in structures **29**, **56**, **69**, and **82**.

It is particularly noteworthy the stability of the furanoid β-anomer (**56**), which amounts up to 28% in the equilibrium mixture. Calculations point to the greater stability of the β-anomer (**56**), which adopts the ^1^ *T*_2_ conformation and is capable of forming two hydrogen bonds: one involving the OH group at C-5 and the iminic nitrogen and the other between the anomeric OH and the OH group at C-3, thus leading to the most stable furanose structure.^71^ In contrast, the α-anomer (**82**), due to a stabilizing hydrogen bond between the OH at C-5 and the anomeric OH, leads to a furanoid ring in ^2^ *T*_1_ conformation.

The theoretical analysis shows that the stability of **56d** decreases by ∼5 kcal/mol on removing the hydrogen bond with the iminic nitrogen by 180°-rotation of the angle C5–O (Table 5 entry e, Figure 11). This value is coincidental to the H-bond energy calculated from geometrical data involving the OH groups at C-1 and C-3 (Table S15), according to the empirical relationship of eq 20.^72^ The parameter *d*_D···A_ denotes the calculated distance (in Å) between donor and acceptor atoms of the hydrogen bonding. As inferred from data gathered in Table S15 (last column), both hydrogen bonds do increase the stability by ∼8–10 kcal/mol.

$$E_{\mathrm{HB}}\;(\mathrm{kcal}/\mathrm{mol})=-5.554\times 10^{5}e^{-4.12d_{D\cdot \cdot \cdot A}}$$ 20

**Figure 11:**
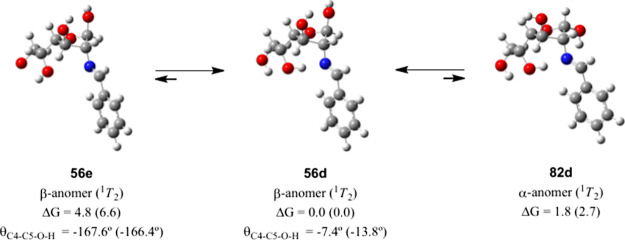
Relative stabilities of α- and β-anomers in furanose structures (kcal/mol) at M06-2X/def2-TZVP in DMSO. Data in parentheses at the M06-2X/6-311G­(d,p) level.

Moreover, the α-anomer (**82**) adopting the ^1^ *T*_2_ conformation could still form an intramolecular hydrogen bond between the OH group at C-5 and the iminic nitrogen, but not the one involving the anomeric OH and the OH group at C-3, thus decreasing its global stability to ∼2 kcal/mol (Figure 11).

It is interesting to point out that the aryliminic group at C-2 adopts invariably the same orientation, regardless of the cyclic structure, either pyranose or furanose. The plane containing that group is approximately orthogonal to the sugar plane, with the lone pair on the iminic nitrogen adopting and antiperiplanar arrangement with respect to the H-2 proton. This general observation suggests the existence of a stereoelectronic effect accounting for that spatial orientation. Such effects involve usually the interaction with heteroatom lone pairs or donor–acceptor orbital interactions that satisfy a stereochemical requisite.

Application of the NBO method^73^ to the most stable conformations of **29**, **56**, **69**, and **82** indicates that the strongest interactions involve heteroatom lone pairs and antibonding orbitals C–C, C–O, and C–H in antiperiplanar dispositions (Tables S16 and S17). Both the anomeric and *exo*-anomeric effects belong to this kind of stereoelectronic effects.^32^ In particular, the interactions between the lone pair on the nitrogen atom and antibonding σ orbitals of the C2–H bond (n_N_ → σ*_C2–H2_ ∼ 5.6–7 kcal/mol) and the CH (iminic) bond (n_N_ → σ*_=C–H_ ∼ 12–13 kcal/mol) manifest themselves in pyranoid and furanoid imines, and justify the spatial arrangement of the arylimino group. Such interactions have been previously reported by other authors.^74,75^ In addition, compound **69** exhibits a significant *endo*-anomeric effect (n_O2_ → σ*_C1–O1_ ∼ 12 kcal/mol), while a stronger *exo*-anomeric effect (n_O1_ → σ*_O2–C1_ ∼ 18 kcal/mol) is present in **29** (Figure 12).

**Figure 12:**
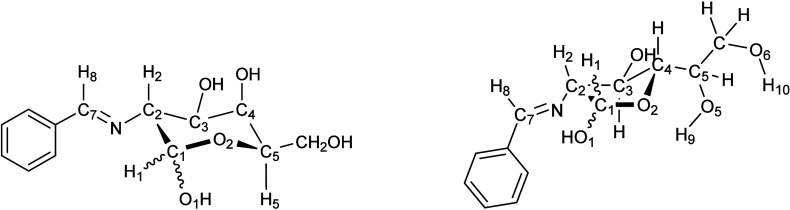
Atom numbering for the NBO analysis of pyranose (**29c**/**69c**) and furanose structures (**56d**/**82c**).

In ^1^ *T*_2_ and ^2^ *T*_1_ conformations, adopted by the furanoid anomers **56d** and **82c**, respectively, the anomeric hydroxy groups prefer pseudoaxial orientations and both show *endo*-anomeric (n_O2_ → σ*_C1–O1_ ∼ 17–18 kcal/mol) and *exo*-anomeric effects (n_O1_ → σ*_C1–O2_ ∼ 12 kcal/mol for β-anomer and ∼8 kcal/mol for its α-counterpart). Furthermore, compound **56d** exhibits a notable interaction between the lone pair on the nitrogen and the antibonding orbital of the OH at C-5 (n_N_ → σ*_O5–H9_ ∼ 14 kcal/mol in DMSO), thus explaining the hydrogen bonding linking those groups and contributing to the stability of the ^2^ *T*_1_ conformer.

A particular advantage of the acetylated derivatives is the possibility of evaluating the relative stability shown by α- and β-anomers in the gas phase and the existence, if any, of a RAE caused by the arylimino function and not disturbed by intramolecular hydrogen bonding. Bearing this goal in mind, gas-phase and CHCl_3_-based computations have been performed on the tetraacetyl derivative **38** and its α-anomer (**101**), which reproduce well the solid-state parameters (bond lengths and dihedral angles, Table S18) obtained by single-crystal X-ray analysis.

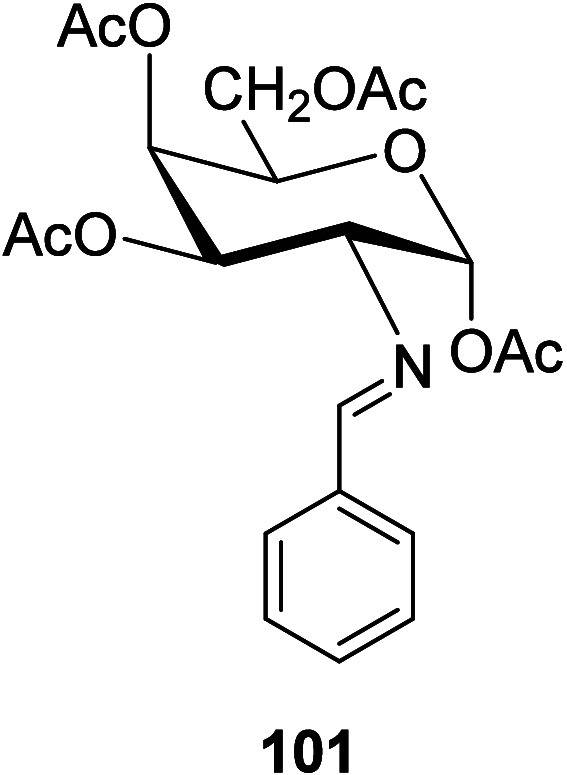



In order to determine the relative stabilities of both anomers, the three possible orientations around the acetoxymethyl group at C-5 have been taken into account (Figure 13). However, only the disposition obtained by X-ray diffractometry has been considered for the remaining acetate groups of **38**, which adopt a *Z* stereochemistry around the CO–O bond; in other words, the C=O bond lies approximately in a parallel arrangement to the contiguous C–H bond. The NBO method^73^ shows a strong interaction n_O_ → π*_C=O_ (∼51 kcal/mol), arising from the electron delocalization of one lone pair on the oxygen atom with the C=O bond, plus another interaction n_O_ → σ*_C–H_ (∼3–5 kcal/mol), which largely account for the orientation adopted by acetate groups (Table S19). Moreover, this conformation significantly minimizes steric repulsions. The orientation adopted by the arylimino group, ranging from the solid state to gas phase and solution, similar to the one adopted by nonacetylated imines, results from a couple of moderate interactions: n_N_ → σ*_C2–H2_ (∼7 kcal/mol) and n_N_ → σ*_=C–H_ (∼13 kcal/mol).

**Figure 13:**
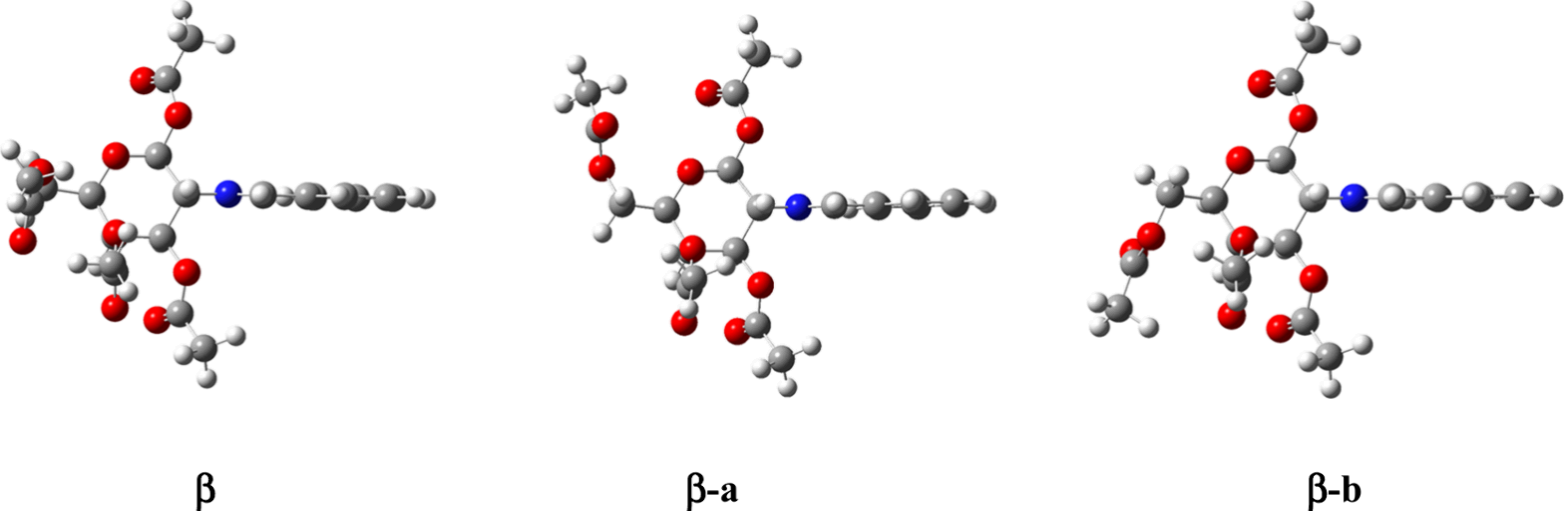
Different conformational orientations of the acetoxymethyl group in **38**.

Irrespective of the level of theory, gas-phase calculations predict a greater stability of the α-anomer over the β-isomer, most likely due to the anomeric effect (Table 7). This result suggests the absence of a RAE, at least for the acetylated derivatives. The difference in stability decreases by incorporating the solvent effect, albeit a completely reverse trend is not observed at all.

**Table 7:** Relative Energies for Different Molecular Arrangements of 38 and 101 (kcal/mol)^a^

		gas phase^a^		CHCl_3_ ^a^		gas phase^b^		CHCl_3_ ^b^	
		Δ*E*	Δ*G*	Δ*E*	Δ*G*	Δ*E*	Δ*G*	Δ*E*	Δ*G*
**38**	β	3.56	4.86	2.11	2.40	3.38	3.92	1.20	1.17
	β-a	1.31	2.05	0.28	0.60	2.90	1.29	1.54	0.94
	β-b	1.03	1.03	0.44	–0.81	1.59	1.95	0.72	1.04
**101**	α	2.66	3.98	1.91	3.60	1.59	2.41	0.36	0.70
	α-a	0.92	0.25	0.24	–0.18	2.75	0.94	1.33	–1.39
	α-b	0.00	0.00	0.00	0.00	0.00	0.00	0.00	0.00

a At the B3LYP/6-31G­(d,p) level.

b At the M06-2X/6-311G­(d,p) level.

The preceding data, however, do not explain why the β-anomer of nonacetylated imines **21**, **27–** **36** becomes the most stable isomer in DMSO solution. Had any stereoelectronic effect counterbalancing the anomeric effect, it would have likely been unveiled by theoretical calculations. It is evident that in the imines of d-galactosamine, like in those derived from d-glucosamine, the presence of the free anomeric hydroxyl is compulsory to establish the crucial hydrogen bonding with the iminic nitrogen, thus generating an RAE.

We then turned our attention to the role played by the solvent on nonacetylated imines. Thus, and regardless of the hybrid functional employed, the difference in stability between α- and β-anomers invariably decreases as the solvent polarity increases. While in the gas phase, the α-pyranose structures are more stable than their β-homologues, the energy difference decreases and even inverts on increasing the polarity (Table 7).

Unfortunately, the SMD method is not fully satisfactory to account for the experimental evidence, because β-anomers are preferentially formed according to NMR data recorded in DMSO-*d*_6_, which disagrees with the relative stabilities determined by computation in the same solvent. The solvation effect could be better reproduced by discrete methods that explicitly consider solvent molecules. In a preliminary analysis, only the first layer of water molecules surrounding the imine molecule has been taken into account. It is well-known that DFT methods computing explicitly the interaction of one molecule of water with the hydroxyl groups of a carbohydrate fragment provide more structural details than those currently employed (e.g., the SMD method), which ignore hydrogen bonding and other conformational aspects affected by solvation. Momany and co-workers^76^ have shown that the use of such discrete models do justify the abundance of β-anomers in D_2_O solutions of d-glucose and d-galactose, despite the existence of anomeric effects. Their conformational analysis performed at the B3LYP/6-311++G­(d,p) level on the different gas-phase and hydrated structures (mono- and pentahydrates), reveal equilibrium α/β populations of d-glucose (32/68) close to those detected experimentally (36/64) and far from the one obtained by computation in the gas phase (63/37).

In the present case, to assess the influence of water on the relative stability of α- and β-anomers, the analysis has been conducted on the monohydrate derivative. Since the imine functionality provides a highly basic center, one could conjecture a strong interaction between the iminic nitrogen and one molecule of water. The latter was deliberately placed near the anomeric OH group, thus enabling hydrogen bonding involving water, the OH group, and the nitrogen atom. This computational analysis was optimized without any geometrical restriction, at the aforementioned M06-2X/6-311G­(d,p)^65^ and M06-2X/def2-TZVP^66^ levels. Data obtained with both basis sets are almost coincidental and are gathered in Table 8 with further visualization in Figure 14.

**Table 8:** Relative Energies of 29 and 69 (Anhydrous and Hydrated Pyranose Forms) in Media of Varied Polarity^a^

		gas phase		DMSO		water	
		Δ*E*°	Δ*G*°	Δ*E*°	Δ*G*°	Δ*E*°	Δ*G*°
**29** ^b^	β	3.33	2.59	1.03	0.59	–0.24	–3.00
**69** ^b^	α	0.00	0.00	0.00	0.00	0.00	0.00
**29·1H**_**2**_ **O** ^b^	β	4.46	4.01	1.92	1.74	0.11	–0.37
**69·1H**_**2**_ **O** ^b^	α	0.00	0.00	0.00	0.00	0.00	0.00
**29·5H**_**2**_ **O** ^b^	β	3.47	3.97	–0.52	–3.43	–0.38	–2.40
**69·5H**_**2**_ **O** ^b^	α	0.00	0.00	0.00	0.00	0.00	0.00
**29·5H**_**2**_ **O** ^c^	β	2.61	2.47	–1.21	–3.78	–0.86	–1.94
**69·5H**_**2**_ **O** ^c^	α	0.00	0.00	0.00	0.00	0.00	0.00

a In kcal/mol.

b M06-2X/6-311G­(d,p).

c M06-2X/def2-TZVP.

**Figure 14:**
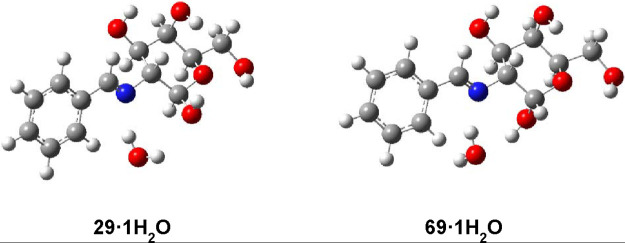
Gas phase-calculated monohydrated structures of anomers **29** and **69**.

Table S20 summarizes the geometrical parameters for hydrogen bonding involving the nitrogen atoms together with a quantitative estimation of their strength (*E*_HB_).^72^ For the β-anomer (**29·1H**_**2**_ **O**), one hydrogen bond is generated between the iminic nitrogen and water (*E*_HB_ ∼ −3.5 kcal/mol). However, the α-anomer (**69·1H**_**2**_ **O**) may establish a second hydrogen bond between the molecule of water and the anomeric OH in axial orientation. The bond length in these intermolecular interactions is shorter than the one found for the hydrogen bond in **29·1H**_**2**_ **O**, thereby indicating stronger interactions (*E*_HB_ ∼ −5.5 kcal/mol), which should most likely account for the greater stability of the α-anomer monohydrate, relative to the situation in the absence of water (Table 8). By applying the SMD method to the monohydrate species, the energy difference between both anomers decreases to a significant extent in DMSO and favors the formation of the β-isomer in water. However, the strength of the hydrogen bond is hardly altered.

In view of these results showing the benefits of computing explicitly discrete molecules of water around the polyhydroxylated sugar chain, further conclusions can be extracted from the interaction with five water molecules. Their orientation is somewhat random, although facilitating the formation of intermolecular hydrogen bonding with the OH groups and the iminic nitrogen. It is clear that intermolecular coordination with water molecules exerts a crucial influence on the anomer stabilization. Such dispositions for pyranose species are depicted in Figure 15. In the case of the α-anomer, the formation of intramolecular hydrogen bonding between the anomeric OH and the nitrogen atom does actually take place. Both the stability and parameters of this bond are compiled in Tables 8 and S21 (*E*_HB_ ∼ −7 to 8 kcal/mol).

**Figure 15:**
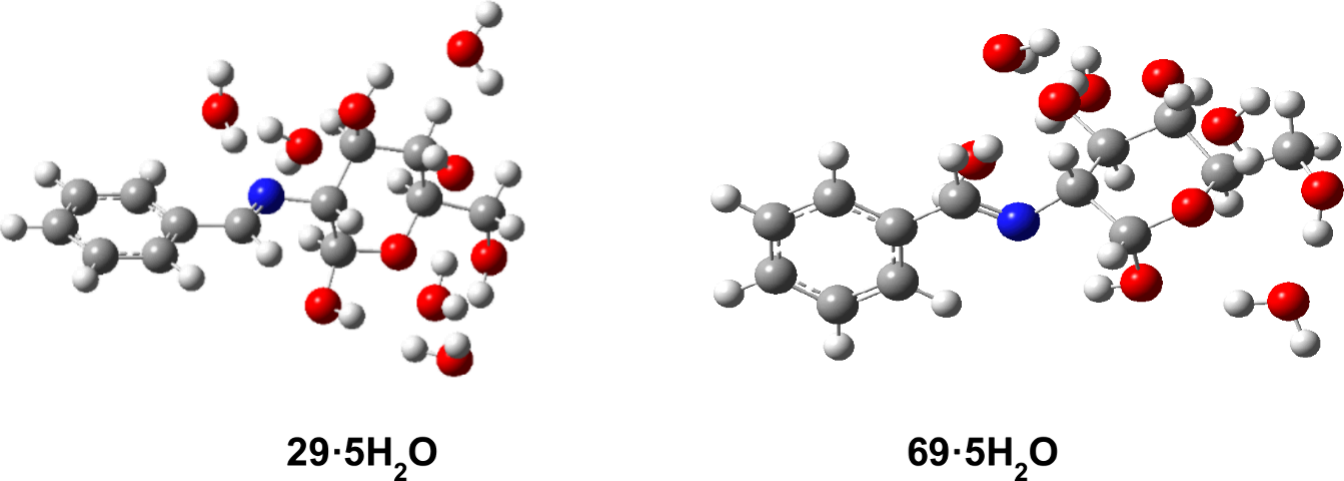
Gas phase-calculated pentahydrated structures of pyranose anomers **29** and **69**.

The energy difference between pentahydrated anomers decreases, although the α-anomer still represents the favored isomer in gas phase. Incorporation of additional water molecules could decrease the energy difference still further, but the computational cost does substantially increase. Since the five-molecule sphere constitutes the first solvation layer, it would be more appropriate the application of the SMD model to pentahydrated species and consider the effects of solvents other than water. Accordingly, the relative stabilities show the opposite trend favoring the β-anomer, both in water and DMSO.

We also calculated the relative energies of **29** and **69** solvated with discrete DMSO molecules (i.e., the solvent in which the RAE was determined). In this case, only three discrete DMSO molecules were considered to generate the first solvation shell, interacting via hydrogen bonds with the OH groups at C-3, C-4, and C-6 of the pyranose ring. The anomeric hydroxyl was left free to form the hydrogen bond with the imine nitrogen. Furthermore, we studied how the presence of DMSO and water as solvent (SMD method) affects the structures obtained in the gas-phase calculations. These results, gathered in Table 9 and Figure 16, were similar to those found for hydrated species: the β-anomer is more stable than the α-anomer.

**Table 9:** Relative Energies of 29 and 69 (Trisolvated Pyranose Forms) in DMSO^a^ ^,^ ^b^

		gas phase		DMSO^c^		water^c^	
comp.	anomer	Δ*E*°	Δ*G*°	Δ*E*°	Δ*G*°	Δ*E*°	Δ*G*°
**29**	β	0.26	–0.78	–0.65	–1.49	–2.29	–3.67
**69**	α	0.00	0.00	0.00	0.00	0.00	0.00

a In kcal/mol.

b M06-2X/def2-TZVP.

c SMD method.

**Figure 16:**
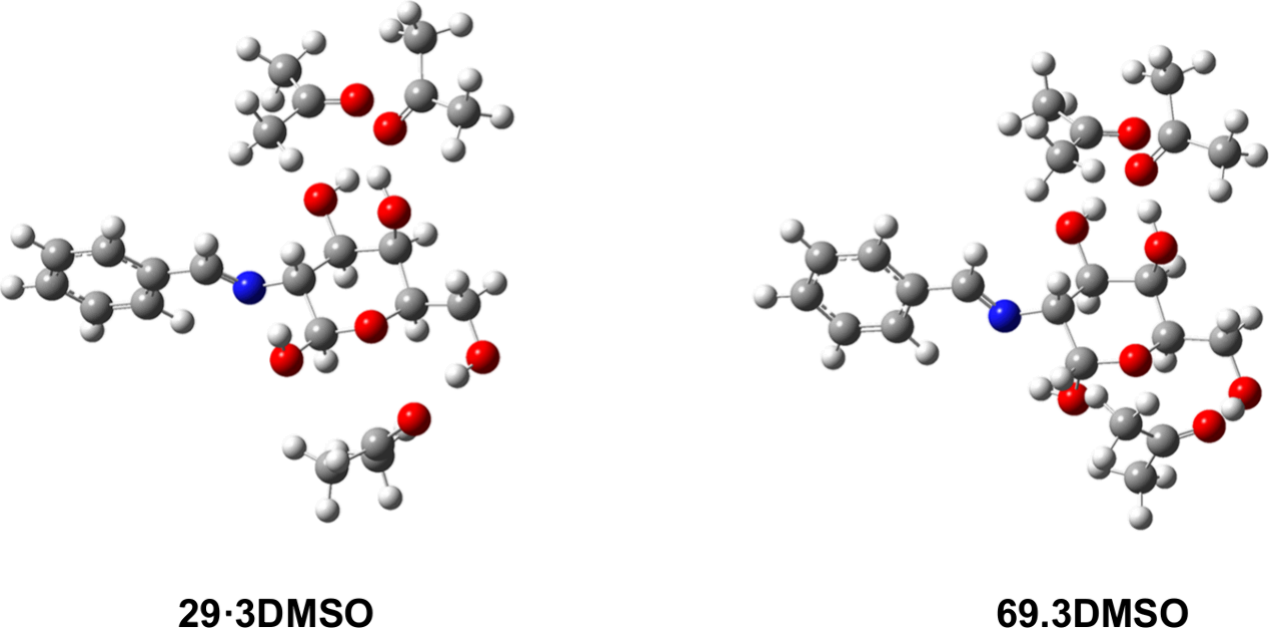
Optimized trisolvated structures (DMSO as solvent), from gas-phase calculations, for pyranose anomers **29** and **69.**

We have applied the same methodology to the β- and α-anomers with furanose structures **56** and **82**, respectively. Five water molecules were arranged next to the hydroxyl groups (Figure 17) with optimization at the M06-2X/6-311G­(d,p) and M06-2X/def2-TZVP levels as well,^65,66^ and devoid of any geometrical restriction. Then, the influence played by solvent effects on the relative stability of both anomers has been examined with the SMD method (Figure 18, Table 10).

**Figure 17:**
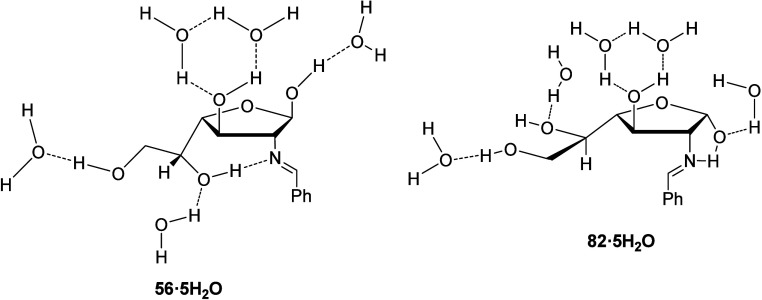
Conformational arrangements of water molecules employed for calculations.

**Table 10:** Relative Energies of 56 and 82 (Pentahydrated Furanose Forms) in Media of Varied Polarity^a^

		gas phase^b^		DMSO^b^		water^b^	
		Δ*E*	Δ*G*	Δ*E*	Δ*G*	Δ*E*	Δ*G*
**56·5H**_**2**_ **O** ^b^	β	0.00	0.00	0.00	0.00	0.00	0.00
**82·5H**_**2**_ **O** ^b^	α	5.09	3.81	4.71	3.15	1.81	2.88
**56·5H**_**2**_ **O** ^c^	β	0.00	0.00	0.00	0.00	0.00	0.00
**82·5H**_**2**_ **O** ^c^	α	3.36	1.22	3.71	1.71	1.29	–0.54

a In kcal/mol.

b M06-2X/6-311G­(d,p).

c M06-2X/def2-TZVP.

**Figure 18:**
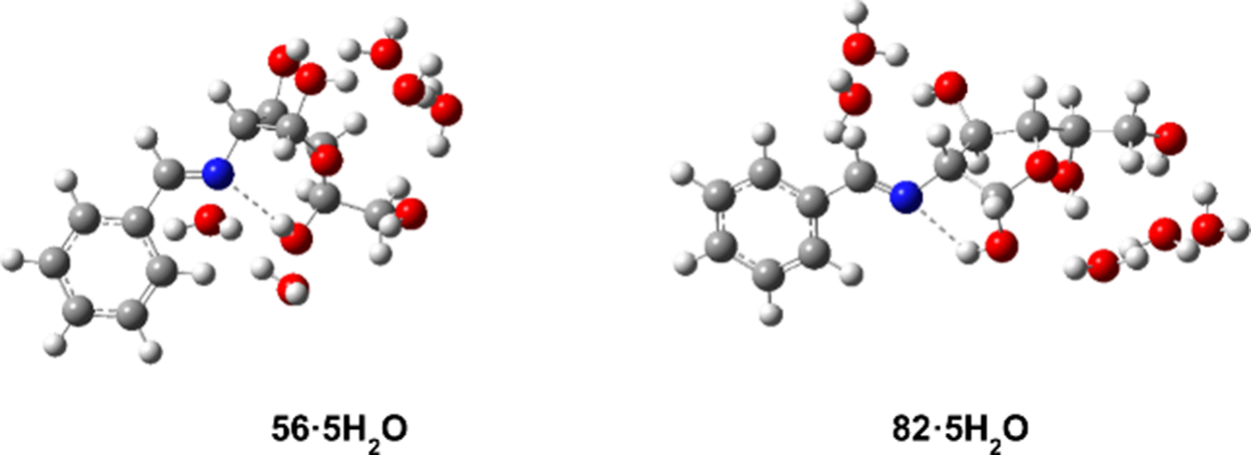
Gas phase-calculated pentahydrated structures of furanose anomers **56** and **82**.

In general, it is observed that the β-furanoid anomer is more stable than the α-furanoid counterpart; especially in DMSO, as observed experimentally. The β-anomer shows the hydrogen bond between the hydroxyl at C-5 and the iminic nitrogen, responsible for its stability and high proportion in solution in DMSO (∼28%). The calculated strength of this bond is ∼5–8 kcal/mol (Table S22).

On the other hand, the α-anomer shows the hydrogen bond involving the nitrogen atom with the anomeric hydroxyl, like the α-pyranose isomer (∼8–9 kcal/mol, Table S23). In this case, the disposition adopted by the anomeric hydroxyl inhibits totally or partially the existence of a possible *exo*-anomeric effect, as occurs with its pyranose counterpart. Therefore, the furanose imines derived from d-galactosamine also present a RAE, responsible, at least in part, for the predominance of the β-anomer in the mutarotational equilibrium. Obviously, further analyses concerning all the possible arrangements of solvated imines, involving either five water molecules or three DMSO molecules, could afford more conclusive results. However, such an in-depth computational study is beyond the scope of the present work.

Finally, we have already indicated that tautomers with enamine structure such as **24** and **95** do not exhibit the RAE, as the nitrogen atom cannot establish a hydrogen bond with the anomeric hydroxyl. Neither could it form with the hydroxyl at C-5 (as in **56d**) to stabilize the furanose structure. As a result, in the anomeric equilibrium of enamine **24**, only the α- and β-configured pyranose structures are present, while the corresponding furanose forms could not be detected in appreciable quantities.^16^

## Conclusions

Schiff bases derived from d-galactosamine are obtained in crystalline form as β-pyranose anomers, which however exhibit a complex equilibrium in solution with the α-anomer as well as with α- and β-furanose structures. Furanose structures in imines derived from 2-amino-deoxyaldoses are unprecedented in the literature. DFT calculations show that the significant presence of the furanose β-anomer (∼28%) is due to the formation of a hydrogen bond between the iminic nitrogen and the hydroxyl at C-5. When the Schiff base adopts an enamine structure, the formation of this H-bond is not possible and, in fact the occurrence of furanose structures is rare. In both types of imines, pyranoses and furanoses, the β-configured anomers are largely favored. An estimation of the RAE in furanoid imines is proposed, which generally turns out to be higher in magnitude than that shown by the corresponding pyranoid imines.

Through experimental measurements and theoretical support from calculations, the configurational bias results from the interplay between a RAE and solvation effects. The former, even if controversial, appears to be genuine enough to overcome purely steric considerations. The prevalence of β-anomers in solution in both pyranose and furanose structures can be ascribed to inhibition of the *exo*-anomeric effect, resulting from an intramolecular hydrogen bond established between the anomeric hydroxyl and the nitrogen atom. This bonding that alters substantially other associated stereoelectronic effects, provides a valuable rationale, apparently overlooked so far, which should stimulate the investigation of this sort of cooperative effects in synthesis and catalysis.

## Experimental Section

### General Information

All reagents and solvents were obtained from commercial suppliers and used without further purification. Compounds **21** and **24** were synthesized according to methods described previously.^16^ Melting points were determined on Gallenkamp and Electrothermal apparatuses and are uncorrected. Optical rotations were measured on a PerkinElmer 241 polarimeter at 20 ± 4 °C, with sodium (D line, λ = 589 nm) and mercury beams (λ = 578, 546, 436 nm). IR spectra were recorded in the range of 4000–600 cm^–1^ on an FT-IR Thermo spectrophotometer. Solid samples were recorded on KBr pellets. ^1^H NMR spectra, at 400 or 500 MHz and ^13^C NMR spectra at 100 or 125 MHz, respectively, were measured on Bruker 400 and 500 AC/PC instruments in DMSO-*d*_6_ or CDCl_3_. Structural elucidation was facilitated through (a) distortionless enhancement by polarization transfer (DEPT), (b) 2D correlation spectroscopy (COSY), (c) heteronuclear multiple-quantum correlation (HMQC), (d) heteronuclear multiple bond correlation (HMBC) and (e) isotope exchange with deuterium oxide. The resonance signals of different deuterated solvents were used as internal standards: CDCl_3_ (δ_H_ = 7.26, δ_C_ = 77.16 ppm), DMSO-*d*_6_ (δ_H_ = 2.50, δ_C_ = 39.5 ppm). The multiplicities of the resonances are abbreviated as followed: s (singlet), bs (broad singlet), d (doublet), dd (doublet of doublets), ddd (doublet of doublets of doublets), t (triplet), m (multiplet). All *J* values are given in Hertz. Microanalyses were determined on a Leco CHNS-932 analyzer. Analytical and preparative TLC was performed on silica gel with monitoring by means of UV light at 254 and 360 nm and iodine vapors. High-resolution mass spectra (HRMS) were obtained using electrospray ionization (ESI) techniques with a 6520 Accurate-Mass Q-TOF LC/MS system from Agilent Technologies at the Servicio de Apoyo a la Investigación (SAIUEX) in the University of Extremadura.

### Computational Details

The computational DFT study, as implemented in the Gaussian09 package,^64^ was carried out using the M06-2X^68^ hybrid density functionals in conjunction with 6-31G­(d,p), 6-311G­(d,p)^65^ and the def2-TZVP valence-triple-ζ basis sets.^66^ The latter has proven to be reliable enough for estimating structure and binding in other carbohydrate derivatives.^77–79^ The M06-2X method was chosen on the basis of previous studies in estimating conformational energies related to noncovalent interactions. In all cases, frequency analyses were carried out to confirm the existence of true stationary points on the potential energy surface. All thermal corrections were calculated at the standard values of 1 atm at 298.15 K. Solvent effects were modeled through density-based, self-consistent reaction field (SCRF) theory of bulk electrostatics, i.e., the solvation model based on density (SMD),^63^ as implemented in the Gaussian09 suite of programs as well. This solvation method accounts for long-range electrostatic polarization (bulk solvent) together with short-range effects due to cavitation, dispersion, and solvent structural effects. We assessed mutarotational equilibria and solvent effects in 2-iminoaldose derivatives using four approaches: (a) gas-phase, as the absence of solvent allows determining the intrinsic stability of each species; (b) continuum solvation: anomerization was studied in solution with a description of the solvent as a continuum dielectric medium, using specifically the SMD model; (c) microsolvation: calculations were conducted in the gas phase, but several water molecules were added to the resulting structures of the stationary points in order to determine the stabilization induced by hydrogen bonding, and (d) microsolvation and continuum solvation, which represents the hybrid between methods (b) and (c). Here, the assembly of the imine with one or several water molecules was studied in a continuum and polarizable dielectric medium. Electronic structures were analyzed by the natural bond orbital (NBO) method.^70,73^

### Crystal Acquisition Data and Structural Refinement

A single crystal of **38** (0.30 × 0.30 × 0.20 mm^3^), suitable for diffraction, was prepared by slow crystallization in 96% ethanol (Table S7). Cell dimensions and intensity data for **38** were recorded at 120 K, using a Bruker Nonius Kappa CCD area detector diffractometer mounted on the window of a rotating Mo anode (λ (Mo Kα) = 0.71073 Å). Data collection and processing were carried out using the programs COLLECT^80^ and DENZO,^81^ and an empirical absorption correction was applied using SADABS.^82^ The structures were solved via direct methods^83^ and refined by full matrix least-squares on *F*2. The hydrogen atoms were placed in calculated positions and included in the refinement using a riding model approximation. Crystallographic illustrations were prepared using the CAMERON programs.^84^

### Synthesis of Schiff Bases from d-Galactosamine

Procedure 1: to a solution of d-galactosamine hydrochloride (0.5 g, 2.3 mmol) in 1*M* NaOH (4.5 mL) was added the appropriate aromatic aldehyde (2.5 mmol), and the mixture was stirred at room temperature for 1 h. After cooling at 4–5 °C overnight, the resulting solid was collected by filtration and washed successively with cold water, ethanol, and diethyl ether, and dried over silicagel.

Procedure 2: to a solution of d-galactosamine hydrochloride (1.01g, 4.7 mmol) in water (6 mL) was added NaHCO_3_ (0.50 g, 4.7 mmol). Then, a methanolic solution of the aromatic aldehyde (4.7 mmol) was added and the resulting mixture was stirred until precipitation of a white solid that was stored at 4–5 °C. As above, it was collected by filtration, washed successively with cold water, ethanol, and diethyl ether, and dried over silica gel.

#### 2-Deoxy-2-[(*E*)-(4-hydroxy-3-methoxybenzylidene)­amino]-β-d-galactopyranose (27)

White microcrystalline solid. Procedure 1 (0.72 g, 100%); Mp 166–168 °C; [Lit.^9b^ Mp 153–155 °C]; [α]_D_ +44.4°; [α]_578_ + 47.6°; [α]_546_ + 55.2°; [α]_436_ + 42.6° (*c* 0.5, pyridine); IR (KBr) ν_máx_ 3370 (OH), 1643 (C=N), 1595, 1518 (arom), 1289 (C–O–C, ether), 1132, 1028 cm^–1^ (C–O), 831 (arom). ^1^H NMR (400 MHz, DMSO-*d*_6_) δ 8.05 (1H, s, CH=N), 7.36 (1H, d, arom), 7.10 (1H, dd, arom), 6.81­(1H, d, arom), 6.44 (1H, bs, C1-OH), 4.63 (1H, d, *J*_1,2_ 7.5, H-1), 3.66 (1H, d, *J*_3,4_ 3.1 Hz, *J*_4,5_ 0 Hz, H-4), 3.59 (2H, m, H-3, H-6), 3.52 (1H, dd, *J*_5,6’_ 6.0 Hz, *J*_6,6′_ 10.7 Hz, H-6′), 3.46 (1H, t, *J*_4,5_ 0 Hz, *J*_5,6_ = *J*_5,6′_ 6.2 Hz, H-5), 3.06 (1H, dd, *J*_1,2_ 7.8 Hz *J*_2,3_ 9.4 Hz, H-2). ^13^C­{^1^H} NMR (100 MHz, DMSO-*d*_6_) δ 162.1 (C=N), 148.1, 128.3, 123.2, 115.9, 115.5, 110.1 (C arom), 96.4 (C-1), 75.4 (C-5), 74.7 (C-2), 71.8 (C-3), 67.5 (C-4), 61.0 (C-6). Anal. calcd for C_14_H_19_NO_7_: C, 53.67, H, 6.11, N, 4.47. Found: C, 53.52, H, 6.11, N, 4.48.

#### 2-Deoxy-2-[(*E*)-(4-methoxybenzylidene)­amino]-β-d-galactopyranose (28)

White microcrystalline solid. Procedure 1 (0.43 g, 63%); Mp 184–186 °C; [Lit.^11^ Mp 150 °C]; [α]_D_ + 30.0°; [α]_578_ + 32.2°; [α]_546_ + 39.0°; [α]_436_ + 62.0° (*c* 0.5, pyridine); IR (KBr) ν_máx_ 3494, 3294, 3183 (OH), 1641 (C=N), 1607, 1516 (arom), 1264 (C–O–C, ether), 1157, 1027 (C–O), 833 cm^–1^ (arom). ^1^H NMR (500 MHz, DMSO-*d*_6_) δ 8.13 (1H, s, CH=N), 7.67 (2H, d, arom), 6.98 (2H, d, arom), 6.43 (1H, d, *J*_1,OH_ 6.5 Hz, C1-OH), 4.63 (1H, t, *J*_1,2_ ≈ *J*_C1,OH_ 7.0 Hz, H-1), 4.60 (1H, bs, C6-OH), 4.51 (1H, d, *J*_C4,OH_ 5.5 Hz, C4-OH), 4.40 (1H, d, *J*_C3,OH_ 3.0 Hz, C3-OH), 3.80 (3H, s, CH_3_), 3.67 (1H, s, H-4), 3.58 (2H, m, H-3, H-6), 3.53 (1H, m, *J*_5,6′_ 6.0 Hz, *J*_6,6′_ 10 Hz, H-6′), 3.46 (1H, t, *J*_4,5_ 0 Hz, *J*_5,6′_ = *J*_5,6′_ 6.0 Hz, H-5), 3.09 (1H, dd, *J*_1,2_ 7.5 Hz, *J*_2,3_ 9.5 Hz, H-2). ^13^C­{^1^H} NMR (125 MHz, DMSO-*d*_6_) δ 161.1 (CH=N), 160.9 (C arom), 129.4 (2 C arom), 129.1 (C arom), 113.8 (2C-arom), 96.0 (C-1), 75.0 (C-5), 74.7 (C-2), 71.5 (C-3), 67.1 (C-4), 60.6 (C-6), 55.1 (OCH_3_). Anal. calcd for C_14_H_19_NO_6_: C, 56.56, H, 6.44, N, 4.71. Found: C, 56.53, H, 6.18, N, 4.31.

#### 2-[(*E*)-Benzylidenamino]-2-deoxy-β-d-galactopyranose (29)

White microcrystalline solid. Procedure 1 (0.45 g, 73%); Mp 190–191 °C; [α]_D_ + 32.6°; [α]_578_ + 35.6°; [α]_546_ + 42.0°; [α]_436_ + 73.0° (*c* 0.5, pyridine); IR (KBr) ν_máx_ 3536, 3243 (OH), 2957, 2895 (C–H), 1640 (C=N), 1580 (arom), 1152, 1078, 1030 (C–O), 881, 762, 692 cm^–1^ (arom). ^1^H NMR (400 MHz, DMSO-*d*_6_) δ 8.21 (1H, s, CH=N), 7.73 (2H, m, arom), 7.44 (3H, m, arom), 6.49 (1H, d, *J*_C1,OH_ 6.8 Hz, C1–OH), 4.68 (1H, t, *J*_1,2_ ≈ *J*_C1,OH_ 7.3 Hz, H-1), 4.63 (1H, t, *J*_C6,OH_ 6.2 Hz, C6–OH), 4.58 (1H, d, *J*_C3,OH_ 6.9 Hz, C3–OH), 4.45 (1H, d, *J*_C4,OH_ 4.4 Hz, C4–OH), 3.67 (1H, t, *J*_3,4_ 3.6 Hz, *J*_4,5_ 0 Hz, H-4), 3.59 (2H, m, H-3, H-6), 3.52 (1H, dd, *J*_5,6′_ 5.3 Hz, *J*_6,6__′_ 10.7 Hz, H-6′), 3.49 (1H, t, *J*_5,6_ = *J*_5,6__′_ 6.1 Hz, H-5), 3.15 (1H, t, *J*_1,2_ ≈ *J*_2,3_ 8.6 Hz, H-2). ^13^C­{^1^H} NMR (100 MHz, DMSO-*d*_6_) δ 162.4 (C=N), 136.5 (C arom), 130.7 (C arom), 128.8 (2C arom), 128.2 (C arom), 96.3 (C-1), 75.4 (C-5), 74.9 (C-2), 71.8 (C-3), 67.4 (C-4), 61.0 (C-6). Anal. calcd for C_13_H_17_NO_5_: C, 58.42, H, 6.06, N, 5.26. Found: C, 58.21, H, 6.33, N, 5.33.

#### 2-Deoxy-2-[(*E*)-(3-hydroxybenzylidene)­amino]-β-d-galactopyranose (30)

White microcrystalline solid. Procedure 1 and recrystallized from methanol (0.53 g, 82%); Mp 191–193 °C; [α]_D_ + 53.0°; [α]_578_ + 57.0°; [α]_546_ + 67.4°; [α]_436_ + 147.4° (*c* 0.5, pyridine); IR (KBr) ν_máx_ 3454, 3090 (OH), 1643 (C=N), 1587 (arom), 1169, 1111, 1018 (C–O), 878, 794 cm^–1^ (arom). ^1^H NMR (500 MHz, DMSO-*d*_6_) δ 9.52 (1H, s, OH-arom), 8.11 (1H, s, CH=N), 7.23 (1H, t, arom), 7.18 (1H, t, arom), 7.11 (1H, d, arom), 6.82 (1H, dd, arom), 6.45 (1H, d, *J*_C1,OH_ 7.0 Hz, C1–OH), 4.63 (2H, m, H-1, C6–OH), 4.54 (1H, d, *J*_C3,OH_ 6.0 Hz, C3–OH), 4.42 (1H, s, C4–OH), 3.67 (1H, s, H-4), 3.58 (3H, m, H-3, H-6, H-6′), 3.46 (1H, t, *J*_4,5_ 0 Hz, *J*_5,6_ = *J*_5,6′_ 6.5 Hz, H-5), 3.11 (1H, dd, *J*_1,2_ 8.0 Hz, *J*_2,3_ 9.5 Hz, H-2). ^13^C­{^1^H} NMR (125 MHz, DMSO-*d*_6_) δ 162.4 (C=N), 157.9, 138.1, 129.9, 119.9, 118.0, 115.0 (C arom), 96.5 (C-1), 75.6 (C-5), 74.9 (C-2), 71.9 (C-3), 67.6 (C-4), 61.2 (C-6). Anal. calcd for C_13_H_17_NO_6_: C, 55.12, H, 6.05, N, 4.94. Found: C, 55.31, H, 6.15, N, 4.98.

#### 2-Deoxy-2-[(*E*)-(2,4,6-trimethylbenzylidene)­amino]-β-d-galactopyranose (31)

White microcrystalline solid. Procedure 1 (0.40 g, 56%); Mp 185–186 °C; [α]_D_ +2.0°; [α]_578_ + 2.0°; [α]_546_ + 2.8°; [α]_436_ + 9.4° (*c* 0.5, pyridine); IR (KBr) ν_máx_ 3500–3000 (OH), 1650 (C=N), 1612 (arom), 1282 (C–O–C, ether), 1156, 1068, 1013 (C–O), 876, 778 cm^–1^ (arom). ^1^H NMR (400 MHz, DMSO-*d*_6_) δ 8.41 (1H, s, CH=N), 6.84 (2H, s, arom), 6.47 (1H, d, *J*_C1,OH_ 6.8 Hz, C1–OH), 4.65 (2H, m, *J*_1,2_ 7.4 Hz, *J*_C6,OH_ 6.0 Hz, C6–OH, H-1), 4.58 (1H, d, *J*_C3,OH_ 7.6 Hz, C3–OH), 4.46 (1H, d, *J*_C4,OH_ 4.8 Hz, C4–OH), 3.67 (1H, t, *J*_3,4_ ≈ *J*_4,OH_ 3.6 Hz, *J*_4,5_ 0 Hz, H-4), 3.59 (1H, dd, *J*_6,6′_ 11.2 Hz, *J*_5,6_ 5.6 Hz, H-6), 3.56 (1H, m, H-3), 3.51 (1H, dd, *J*_6,6’_ 11.2 Hz, *J*_5,6′_ 5.6 Hz, H-6′), 3.45 (1H, t, *J*_5.6_ ≈ *J*_5.6′_ 6.0 Hz, *J*_4,5_ 0 Hz, H-5), 3.11 (1H, t, *J*_1,2_ ≈ *J*_2,3_ 8.6 Hz, H-2). ^13^C­{^1^H} NMR (100 MHz, DMSO-*d*_6_) δ 162.1 (C=N), 137.2 (3C arom), 131.7 (C arom), 129.1 (2C arom), 96.4 (C-1), 75.9 (C-5), 75.4 (C-2), 71.7 (C-3), 67.5 (C-4), 61.0 (C-6), 21.0 (CH_3_), 20.5 (2 CH_3_). Anal. calcd for C_16_H_23_NO_5_: C, 62.12, H, 7.49, N, 4.53. Found: C, 62.20; H, 7.33; N, 4.60.

#### 2-Deoxy-2-[(*E*)-(1-naphthylmethylene)­amino]-β-d-galactopyranose (32)

White microcrystalline solid. Procedure 2 (0.46 g, 31%); Mp 134–135 °C; [α]_D_ + 14.2°; [α]_578_ + 12.6°; [α]_546_ + 14.6°; [α]_436_ + 100.2° (*c* 0.5, pyridine); IR (KBr) ν_máx_ 3265 (OH), 1636 (C=N), 1576, 1511 (arom), 1236 (C–O–C, ether), 1151, 1082, 1028 (C–O), 803, 775 cm^–1^ (arom). ^1^H NMR (500 MHz, DMSO-*d*_6_) δ 9.05 (1H, d, arom), 8.84 (1H, s, CH=N), 8.02 (1H, d, arom), 7.98 (1H, d, arom), 7.91 (1H, d, arom), 7.58 (3H, m, arom), 6.54 (1H, d, *J*_C1–OH_ 7.0 Hz, C1-OH), 4.77 (1H, t, *J*_1,2_ ≈ *J*_C1,OH_ 7.5 Hz, H-1), 4.64 (2H, m, *J*_C3–OH_ 7.5 Hz, *J*_C6–OH_ 4.0 Hz, C3-OH, C6-OH), 4.51 (1H, d, *J*_C4–OH_ 4.0 Hz, C4-OH), 3.73 (1H, m, *J*_3,4_ 3.5 Hz, *J*_4,5_ 0 Hz, H-4), 3.67 (1H, m, *J*_5,6_ 5.0 Hz, *J*_6,6′_ 10 Hz, H-6), 3.62 (1H, m, H-3), 3.57 (1H, m, *J*_5,6′_ 6 Hz, *J*_6,6′_ 11 Hz, H-6′), 3.53 (1H, m, *J*_5,6_ 6.0 Hz, H-5), 3.27 (1H, dd, *J*_1,2_ 8.0 Hz, *J*_2,3_ 9.5 Hz, H-2). ^13^C­{^1^H} NMR (125 MHz, DMSO-*d*_6_) δ 161.9 (C=N), 133.3, 131.4, 130.6, 130.5, 128.8, 128.3, 126.9, 126.0, 125.2, 124.7 (C arom), 96.1 (C-1), 75.4 (C-5), 75.1 (C-2), 71.6 (C-3), 67.2 (C-4), 60.7 (C-6). Anal. calcd for C_17_H_19_NO_5_: C, 64.34, H, 6.03, N, 4.41. Found: C, 64.52, H, 5.88, N, 4.46.

#### 2-Deoxy-2-[(*E*)-(4-methoxy-1-naphthyl)­methylene]­amino-β-d-galactopyranose (33)

White microcrystalline solid. Procedure 2 (0.28 g, 18%); Mp 165–167 °C; [α]_578_ + 13.0; [α]_546_ + 14.8 (*c* 0.5, pyridine); IR (KBr) ν_máx_ 3296 (OH), 1678 (C=N), 1578, 1514 (arom), 1229 (C–O–C, ether), 1086, 1015 (C–O), 808 cm^–1^ (arom). ^1^H NMR (400 MHz, DMSO-*d*_6_) δ 9.21 (1H, d, arom), 8.67 (1H, s, CH=N), 8.22 (1H, d, arom), 7.82 (1H, d, arom), 7.62 (1H, t, arom), 7.55 (1H, t, arom), 7.06 (1H, d, arom), 6.50 (1H, d, *J*_C1‑OH_ 7.0 Hz, C1-OH), 4.74 (1H, t, *J*_1,2_ ≈ *J*_C1,OH_ 7.3 Hz, H-1), 4.65 (1H, t, *J*_C6‑OH_ 5.0 Hz, C6-OH), 4.60 (1H, d, *J*_C3‑OH_ 7.0 Hz, C3-OH), 4.49 (1H, d, *J*_C4‑OH_ 4.2 Hz, C4-OH), 4.02 (3H, s, OCH_3_), 3.72–3.56 (4H, m, H-3, H-4, H-6, H-6′), 3.52 (1H, m, *J*_5,6_ 5.7 Hz, H-5), 3.19 (1H, t, *J*_1,2_ = *J*_2,3_ 8.6 Hz, H-2). ^13^C­{^1^H} NMR (100 MHz, DMSO-*d*_6_) δ 162.5 (C=N), 156.8, 132.0, 131.6, 127.7, 125.8, 125.4, 125.2, 124.4, 122.0, 104.2 (C arom), 96.6 (C-1), 75.7 (C-5), 75.4 (C-2), 72.1 (C-3), 67.6 (C-4), 61.0 (C-6), 56.1 (OCH_3_). Anal. calcd for C_18_H_21_NO_6_·H_2_O: C, 59.17, H, 6.35, N, 3.83. Found: C, 59.35, H, 6.57, N, 3.83.

#### 2-Deoxy-2-[(*E*)-(2-naphthylmethylene)­amino]-β-d-galactopyranose (34)

White microcrystalline solid. Procedure 2 (0.42 g, 28%); Mp 178–180 °C; [α]_D_ + 44.0°; [α]_578_ + 46.6°; [α]_546_ + 56.2°; [α]_436_ + 133.2° (*c* 0.5, pyridine); IR (KBr) ν_máx_ 3545, 3246 (OH), 1634 (C=N), 1433 (arom), 1152, 1082, 1034 (C–O), 746 cm^–1^ (arom). ^1^H NMR (400 MHz, DMSO-*d*_6_) δ 8.39 (1H, s, CH=N), 8.19 (1H, s, arom), 8.01 (1H, m, arom), 7.95 (3H, m, arom), 7.56 (2H, m, arom), 6.56 (1H, d, *J*_C1‑OH_ 6.9 Hz, C1-OH), 4.73 (1H, t, *J*_1,2_ ≈ *J*_C1,OH_ 7.3 Hz, H-1), 4.67 (2H, m, *J*_C3‑OH_ 7.1 Hz, *J*_C6‑OH_ 5.4 Hz, C3-OH, C6-OH), 4.51 (1H, d, *J*_C4‑OH_ 4.4 Hz, C4-OH), 3.72 (1H, m, H-4), 3.60 (4H, m, H-3, H-5, H-6, H-6’), 3.24 (1H, t, *J*_1,2_ = *J*_2,3_ 8.6 Hz, H-2). ^13^C­{^1^H} NMR (100 MHz, DMSO-*d*_6_) δ 162.8 (C=N), 134.5, 134.3, 133.2, 130.2, 129.0, 128.6, 128.2, 127.7, 127.1, 124.2 (C arom), 96.5 (C-1), 75.6 (C-5), 75.1 (C-2), 72.0 (C-3), 67.7 (C-4), 61.2 (C-6). Anal. calcd for C_17_H_19_NO_5_: C, 64.34, H, 6.04, N, 4.41. Found: C, 64.21, H, 5.83, N, 4.39.

#### 2-[(*E*)­(9-Anthrylmethylene)­amino]-2-deoxy-β-d-galactopyranose (35)

Yellowish powder. Procedure 2 (1.21 g, 67%); Mp 162–164 °C; [α]_578_ + 4.0°; [α]_546_ + 5.0° (*c* 0.5, pyridine); IR (KBr) ν_máx_ 3391 (OH), 1645 (C=N), 1643, 1519 (arom), 1158, 1073, 1017 (C–O), 875, 798 cm^–1^ (arom); ^1^H NMR (500 MHz, DMSO-*d*_6_) δ 9.29 (1H, s, CH=N), 8.64 (3H, m, arom), 8.12 (2H, m, arom), 7.54 (4H, m, arom), 6.77 (1H, d, *J*_C1,OH_ 7.5 Hz, C1-OH), 4.89 (1H, d, *J*_C3,OH_ 7.5 Hz, C3-OH), 4.84 (1H, t, *J*_1,2_ ≈ *J*_C1,OH_ 7.5, H-1), 4.67 (1H, t, *J*_C6,OH_ 5.5 Hz, C6-OH), 4.64 (1H, d, *J*_C4,OH_ 4.5 Hz, C4-OH), 3.80 (1H, m, H-4), 3.76 (1H, m, H-3), 3.66 (1H, m, H-6), 3.62 (1H, m, H-6′), 3.56 (2H, m, H-2, H-5). ^13^C­{^1^H} NMR (125 MHz, DMSO-*d*_6_) δ 161.3 (C=N), 130.7 (2C arom), 129.4 (C arom), 129.0 (2C arom), 128.4 (2C arom), 128.2 (C arom), 126.6 (2C arom), 125.5 (2C arom), 125.3 (2C arom), 96.1 (C-1), 75.9 (C-2), 75.3 (C-5), 71.5 (C-3), 67.3 (C-4), 60.7 (C-6). Anal. calcd for C_21_H_21_NO_5_·H_2_O: C, 65.44, H, 6.02, N, 3.63. Found: C, 65.61, H, 6.14, N, 3.43.

#### 2-Deoxy-2-[(*E*)-(9-phenanthrylmethylene)­amino]-β-d-galactopyranose (36)

Yellowish powder. Procedure 2 (0.73 g, 42%); Mp 118–120 °C; [α]_D_ 18.2°; [α]_578_ 18.6°; [α]_546_ 14.6° (*c* 0.5, pyridine); IR (KBr) ν_máx_ 3335 (OH), 1637 (C=N, arom), 1446 (arom), 1149 (C–O–C, ether), 1084 (C–O), 745 cm^–1^ (arom). ^1^H NMR (400 MHz, DMSO-*d*_6_) δ 9.25 (1H, d, arom), 8.91 (1H, d, arom), 8.86 (1H, s, arom), 8.84 (1H, d, arom), 8.23 (1H, s, CH=N), 8.10 (1H, d, arom), 7.73 (4H, m, arom), 6.61 (1H, d, *J*_C1‑OH_ 7.0 Hz, C1-OH), 4.82 (1H, t, *J*_1,2_ ≈ *J*_C1,OH_ 7.1 Hz, H-1), 4.72 (2H, m, C3-OH, C6-OH), 4.64 (1H, m, C4-OH), 3.76 (1H, m, H-4), 3.58 (4H, m, H-3, H-5, H-6, H-6′), 3.32 (1H, t, *J*_1,2_ = *J*_2,3_ 8.5 Hz, H-2). ^13^C­{^1^H} NMR (100 MHz, DMSO-*d*_6_) δ 162.8 (C=N), 131.2, 130.8, 130.4, 129.5, 128.0, 127.5, 127.4, 127.2, 126.1, 123.4, 123.1 (C arom), 96.4 (C-1), 75.9 (C-5), 75.5 (C-2), 71.9 (C-3), 67.5 (C-4), 61.0 (C-6). Anal. calcd for C_21_H_21_NO_5_: C, 68.65, H, 5.76, N, 3.81. Found: C, 68.76; H, 5.57; N 3.68.

### General Procedure for the Synthesis of Per-*O*-acetyl-2-(arylmethylene)­amino-2-deoxy-d-galactopyranoses

To a suspension of the corresponding 2-(arylmethylene)­amino-2-deoxy-β-d-galactopyranose (1.8 mmol) in pyridine (2.4 mL) was added acetic anhydride (2.2 mL) under stirring and cooling at 0 °C with ice. After 24 h, it was poured into ice–water and a solid was subsequently obtained after agitation, which was collected by filtration and washed with cold water, and dried over SiO_2_.

#### 1,3,4,6-Tetra-*O*-acetyl-2-deoxy-2-[(*E*)-(3-methoxy-4-acetoxybenzylidene)­amino]-β-d-galactopyranose (37) and 1,3,4,6-Tetra-*O*-acetyl-2-deoxy-2-[(*E*)-(3-methoxy-4-acetoxy-benzylidene)­amino]-β-d-galactopyranose Acetate (41)

(a) Using the general method a mixture of **37** and **41** (proportion 65:35, respectively) (0.21 g) was obtained from **27** (0.56 g). (b) Following the general method from **27** (0.56 g, 1.8 mmol) and pouring the reaction mixture into ice–water with added sodium hydrogen carbonate, solid **37** (0.39 g, 41%, white crystal) precipitated. Crystallized from ethanol–water, it had Mp. 219–221 °C; [α]_D_ + 13.6°; [α]_578_ + 15.0°; [α]_546_ + 17.6°; [α]_436_ + 25.8° (*c* 0.5, CHCl_3_); IR (KBr) ν_máx_ 3262 (NH), 1753 (C=O), 1647 (C=N), 1568 (arom), 1217 (C–O–C, ether), 1078 (C–O), 901, 750 cm^–1^ (arom). ^1^H NMR (400 MHz, CDCl_3_) δ 9.95 (1H, s, CH=N), 7.51–7.07 (3H, arom), 5.94 (1H, d, *J*_1,2_ 8.2 Hz, H-1), 5.46 (1H, d, *J*_3,4_ 3.3 Hz, H-4), 5.26 (1H, dd, *J*_2,3_ 10.5 Hz, H-3), 4.23–4.12 (3H, m, H-5, H-6, H-6′), 3.91 (3H, s, OCH_3_), 3.65 (1H, t, *J*_1,2_ ≈ *J*_2,3_ 9.2 Hz, H-2), 2.35, 2.18, 2.06, 2.05, 1.91 (5 × 3H, s, CH_3_). ^13^C­{^1^H} NMR (100 MHz, CDCl_3_) δ 170.3, 170.1, 169.5, 168.8 (C=O), 164.3 (C=N), 151.5, 135.2, 124.7, 123.4, 122.7, 110.8 (C arom), 93.3 (C-1), 71.8 (C-2), 71.4 (C-5), 68.8 (C-3), 65.8 (C-4), 61.3 (C-6), 56.1 (OCH_3_), 20.8 (1 × CH_3_, acetate), 20.6 (3 × CH_3_, acetate). Anal. calcd for C_24_H_29_NO_12_: C, 55.07, H, 5.58, N, 2.68. Found: C, 54.87; H, 5.43; N 2.58.

### Spectral Data of Compound 41

^1^H NMR (400 MHz, CDCl_3_) δ 8.23 (1H, s, CH=N), 7.51–7.07 (3H, arom), 5.70 (1H, d, *J*_1,2_ 8.7 Hz, H-1), 5.54 (1H, d, *J*_NH,2_ 9.5 Hz, NH), 5.37 (1H, d, *J*_3,4_ 3.1 Hz, H-4), 5.08 (1H, dd, *J*_2,3_ 11.2 Hz, H-3), 4.48 (1H, c, *J*_1,2_ ≈ *J*_2,3_ ≈ *J*_2,NH_ 10.5 Hz, H-2), 4.16 (3H, m, H-5, H-6, H-6′), 3.89 (3H, s, OCH_3_), 2.33, 2.13, 2.04, 1.94 (4 × 3H, s, CH_3_). ^13^C­{^1^H} NMR (100 MHz, CDCl_3_) δ 170.7, 170.4, 169.7, 168.3 (C=O), 164.3 (C=N), 152.0, 134.3, 124.7, 123.4, 122.8, 110.5 (C arom), 93.0 (C-1), 71.8 (C-2), 71.4 (C-5), 70.3 (C-3), 66.3 (C-4), 61.3 (C-6), 56.0 (OCH_3_), 23.3, 20.8 (2 CH_3_), 20.6 (2 CH_3_).

#### 1,3,4,6-Tetra-*O*-acetyl-2-[(*E*)-benzylideneamino]-2-deoxy-β-d-galactopyranose (38) and 1,3,4,6-Tetra-*O*-acetyl-2-[(*E*)-benzylideneamino]-2-deoxy-β-d-galactopyranose Acetate (40)

A mixture of **38** and **40** (proportion ∼ 95:5, respectively) (0.76 g) was obtained from **29** (0.48 g). Compound **38** could be isolated by fractional crystallization in ethanol–water (0.24 g, 31%, white crystal). Mp 134–135 °C; [α]_D_ + 43.6°, [α]_578_ + 46.4°, [α]_546_ + 53.6°, [α]_436_ + 111.4°, [α]_365_ + 232.0° (*c* 0.5, CHCl_3_); IR (KBr) ν_máx_ 1750 (C=O), 1647 (C=N), 1582 (arom), 1215 (C–O–C, ether), 1074 (C–O), 931, 756, 692 cm^–1^ (arom). ^1^H NMR (400 MHz, CDCl_3_) δ 8.30 (1H, s, CH=N), 7.72 (2H, d, arom), 7.43 (3H, m, arom), 5.95 (1H, d, *J*_1,2_ 8.2 Hz, H-1), 5.47 (1H, d, *J*_3,4_ 3.3 Hz, H-4), 5.27 (1H, dd, *J*_2,3_ 10.4 Hz, H-3), 4.19 (3H, m, H-5, H-6, H-6′), 3.65 (1H, t, *J*_1,2_ 8.2 Hz, H-2), 2.18, 2.11, 2.03, 1.89 (4 × 3H, s, CH_3_). ^13^C­{^1^H} NMR (100 MHz, CDCl_3_) δ 170.9, 170.4, 169.6, 168.7 (C=O), 165.3 (C=N), 135.4, 131.4, 128.7, 128.5 (C arom), 93.4 (C-1), 71.8 (C-2), 71.4 (C-5), 68.8 (C-3), 65.9 (C-4), 61.3 (C-6), 20.7 (2 CH_3_), 20.5 (CH_3_). Anal. calcd for C_21_H_25_NO_9_: C, 57.93, H, 5.79, N, 3.22. Found: C, 57.43, H, 5.94, N, 3.45.

### Spectral Data of Compound 40

^1^H NMR (400 MHz, CDCl_3_) δ 8.30 (1H, s, CH=), 7.72 (2H, d, arom), 7.43 (3H, m, arom), 5.71 (1H, d, *J*_1,2_ 9.9 Hz, H-1), 5.50 (1H, d, *J*_NH,2_ 10.1 Hz, NH), 5.38 (1H, d, *J*_3,4_ 2.8 Hz, H-4), 5.09 (1H, dd, *J*_2,3_ 11.3 Hz_,_ *J*_3,4_ 3.3 Hz, H-3), 4.46 (1H, c, *J*_2,3_ = *J*_3,4_ = *J*_2,NH_ 10.1 Hz, H-2), 4.16 (2H, m, H-6, H-6′), 4.03 (1H, c, *J*_4,5_ 0 Hz, *J*_5,6_ 5.5 Hz, H-5), 2.17, 2.13, 2.05, 2.03, 1.94 (5 × 3H, s, CH_3_, acetate groups). ^13^C­{^1^H} NMR (100 MHz, CDCl_3_) δ 170.9, 170.4, 169.6, 168.7 (C=O), 165.3 (N=CH), 135.4, 131.4, 128.7, 128.5 (C arom), 93.0 (C-1), 71.8 (C-2), 71.4 (C-5), 70.3 (C-3), 66.3 (C-4), 61.3 (C-6), 20.7 (2 CH_3_), 20.5 (CH_3_).

#### 1,3,4,6-Tetra-*O*-acetyl-2-deoxy-2-[(*E*)-(4-methoxybenzylidene)­amino]-β-d-galactopyranose (39)

White microcrystalline solid. The title compound (0.47 g, 56%) was obtained from **28**. Mp 175–176 °C; [α]_D_ + 56.4°; [α]_578_ + 47.6°; [α]_546_ + 55.2°; [α]_436_ + 42.6° (*c* 0.5, CHCl_3_); IR (KBr) ν_máx_ 1748 (C=O), 1643 (C=N), 1607, 1514 (arom), 1254 (C–O–C, ether), 1067 (C–O), 837, 712 cm^–1^ (arom). ^1^H NMR (400 MHz, CDCl_3_) δ 8.21 (1H, s, CH=N), 7.66 (2H, d, arom), 6.93 (2H, d, arom), 5.93 (1H, d, *J*_1,2_ 8.2 Hz, H-1), 5.47 (1H, d, *J*_3,4_ 3.3 Hz, *J*_4,5_ 0 Hz, H-4), 5.25 (1H, dd, *J*_3,4_ 3.3 Hz, *J*_2,3_ 10.4 Hz H-3), 4.19 (3H, m, H-5, H-6, H-6′), 3.61 (1H, t, *J*_1,2_ 8.3 Hz, *J*_2,3_ 10.2 Hz, H-2), 2.18, 2.05, 2.03, 1.89 (4 × CH_3_). ^13^C­{^1^H} NMR (100 MHz, CDCl_3_) δ 170.5, 170.1, 169.7, 168.8 (C=O), 164.4 (C=N), 162.2, 130.2, 128.4 (C arom), 114.0 (2C arom), 93.5 (C-1), 71.7 (C-2), 71.5 (C-5), 68.8 (C-3), 65.9 (C-4), 61.3 (C-6), 55.4 (OCH_3_), 20.8 (CH_3_), 20.7 (2 CH_3_), 20.5 (CH_3_). Anal. calcd for C_22_H_27_NO_10_: C, 56.77, H, 5.85, N, 3.01. Found: C, 57.67, H, 5.51, N, 3.19.

#### 1,3,4,6-Tetra-*O*-acetyl-2-deoxy-2-[(*E*)-(4-methoxy-1-naphthyl)­methylene]­amino-β-d-galactopyranose (42)

White microcrystalline solid. The title compound (0.46 g, 50%) was obtained from **33**. Mp 128–130 °C; [α]_D_ + 39.0°; [α]_578_ + 40.4°; [α]_546_ + 49.0°; [α]_436_ + 117.2° (*c* 0.5, CHCl_3_); IR (KBr) ν_máx_ 1746 (C=O), 1645 (C=N), 1577 (arom), 1232 (C–O–C, ether), 1091, 1035 cm^–1^ (C–O). ^1^H NMR (400 MHz, CDCl_3_) δ 8.91 (1H, d, arom), 8.79 (1H, s, CH=N), 8.32 (1H, d, arom), 7.78 (1H, d, arom), 7.61 (1H, m, arom), 7.53 (1H, m, arom), 6.85 (1H, d, arom), 6.04 (1H, d, *J*_1,2_ 8.4 Hz, H-1), 5.51 (1H, d, *J*_3,4_ 2.8 *J*_4,5_ 0 Hz, H-4), 5.36 (1H, dd, *J*_3,4_ 3.2 *J*_2,3_ 10.4, H-3), 4.22 (3H, m, H-5, H-6, H-6′), 3.70 (1H, dd, *J*_1,2_ 8.4 Hz, *J*_2,3_ 10.0 Hz, H-2), 2.21, 2.07, 2.02, 1.89 (4 × 3H, s, CH_3_, acetate groups). ^13^C­{^1^H} NMR (100 MHz, CDCl_3_) δ 170.4, 170.1, 169.7, 168.8 (C=O), 165.1 (C=N), 132.2, 131.5, 129.5, 128.01, 125.6, 125.5, 124.2, 122.4, 122.3, 103.2 (C arom), 93.6 (C-1), 71.8 (C-2), 71.7 (C-5), 69.7 (C-3), 66.0 (C-4), 61.3 (C-6), 55.7 (OCH_3_), 20.7, 20.5 (4 × CH_3_). Anal. calcd for C_26_H_29_NO_10_: C, 60.58, H, 5.67, N, 2.72. Found: C, 60.34; H, 5.72: N, 2.88.

#### 1,3,4,6-Tetra-*O*-acetyl-2-deoxy-2-[(*E*)-(2-naphthylmethylene)­amino]-β-d-galactopyranose (43)

White microcrystalline solid. The title compound (0.50 g, 57%) was obtained from **34**. Mp 85–86 °C; [α]_D_ + 33.4°; [α]_578_ + 34.6°; [α]_546_ + 39.8°; [α]_436_ + 83.8° (*c* 0.5, CHCl_3_); IR (KBr) ν_máx_ 1751 (C=O), 1643 (C=N, arom), 1217 (C–O–C, ether), 1042 (C–O), 752 cm^–1^ (arom). ^1^H NMR (400 MHz, CDCl_3_) δ 8.35 (1H, s, CH=N), 8.05 (1H, s, arom), 7.90 (4H, m, arom), 6.00 (1H, d, *J*_1,2_ 8.2 Hz, H-1), 5.49 (1H, d, *J*_3,4_ 3.3 *J*_4,5_ 0 Hz, H-4), 5.32 (1H, dd, *J*_3,4_ 3.4 *J*_2,3_ 10.5, H-3), 4.19 (3H, m, H-5, H-6, H-6′), 3.73 (1H, dd, *J*_1,2_ 8.2 Hz, *J*_2,3_ 10.3 Hz, H-2), 2.20, 2.07, 2.05, 1.91 (4 × 3H, s, CH_3_, acetate groups). ^13^C­{^1^H} NMR (100 MHz, CDCl_3_) δ 170.4, 170.1, 169.7, 168.7 (C=O), 165.4 (C=N), 135.0, 134.6, 133.1, 132.8, 129.5, 128.9, 128.6, 128.0, 123.8, 122.7 (C arom), 93.4 (C-1), 71.8 (C-2), 71.5 (C-5), 69.0 (C-3), 65.9 (C-4), 61.3 (C-6), 20.7, 20.7, 20.5 (4 CH_3_). Anal. calcd for C_25_H_27_NO_9_: C, 61.85, H, 5.61, N, 2.89. Found: C, 61.93; H, 5.84; N, 2.76.

#### 1,3,4,6-Tetra-*O*-acetyl-2-deoxy-2-[(*E*)-(9-anthrylmethylene)­amino]-β-d-galactopyranose (44)

Yellowish powder. The title compound (0.79 g, 83%) was obtained from **35**. Mp 225–228 °C; [α]_578_ + 31.0°; [α]_546_ + 38.4° (*c* 0.5, CHCl_3_). ^1^H NMR (400 MHz, CDCl_3_) δ 9.49 (1H, s, CH=N), 8.54 (1H, s, arom), 8.34 (2H, m, arom), 8.04 (2H, m, arom), 7.52 (4H, m, arom), 6.13 (1H, d, *J*_1,2_ 8.3 Hz, H-1), 5.59 (1H, d, *J*_3,4_ 3.2 Hz, *J*_4,5_ 0 Hz, H-4), 5.47 (1H, dd, *J*_3,4_ 3.2 Hz, *J*_2,3_ 10.5 Hz, H-3), 4.26 (3H, m, H-5, H-6, H-6’), 4.05 (1H, dd, *J*_1,2_ 8.4 Hz, *J*_2,3_ 10.4 Hz, H-2). ^13^C­{^1^H} NMR (100 MHz, CDCl_3_) δ170.5, 170.2, 169.7, 168.6 (4 C=O), 165.9 (C=N), 131.2, 130.0, 129.1, 127.4, 127.0, 125.4, 124.0 (C arom), 93.4 (C-1), 71.9 (C-2), 71.7 (C-5), 70.5 (C-3), 65.9 (C-4), 61.3 (C-6), 20.8 (2 OCH_3_), 20.7 (OCH_3_), 20.6 (OCH_3_). Anal. calcd for C_29_H_29_NO_9_·H_2_O: C, 62.92, H, 5.64, N, 2.53. Found: C, 63.18, H, 5.58, N, 2.67.

### Mutarotation of d-Galactosamine Imines

The corresponding imine was dissolved in DMSO-*d*_6_ and NMR spectra were recorded at different intervals until they remained unchanged.

#### 2-[(*E*,*E*)-Cinnamylideneamino]-2-deoxy-β-d-galactofuranose (53)

^1^H NMR (400 MHz, DMSO-*d*_6_) δ 8.05 (1H, d, *J*_=C*H*–CH_ 8.8 Hz, N=C*H*–CH), 7.59 (2H, m, arom), 7.36 (3H, m, arom), 7.17 (1H, d, *J*_CH=C*H*_ 16.0 Hz, CH=C*H*–Ar), 6.94 (1H, dd, *J*_C*H*=CH_ 18.5 Hz, *J*_C*H*–CH_ 8.8 Hz, CH–C*H*=CH), 6.47 (1H, d, *J*_C1‑OH_ 6.4 Hz, C1-OH), 5.25 (1H, d, *J*_C3‑OH_ 6.3 Hz, C3-OH), 5.13 (1H, t, *J*_1,2_ ≈ *J*_C1‑OH_ 5.8 Hz, H-1), 4.13 (1H, c, *J*_2,3_ ≈ *J*_3,4_ 7.5 Hz, H-3), 3.88 (1H, dd, *J*_3,4_ 8.4 Hz, *J*_4,5_ 1.8 Hz, H-4), 3.67–3.36 (4H, m, H-2, H-5, H-6, H-6’). ^13^C­{^1^H} NMR (100 MHz, DMSO-*d*_6_) δ 164.0 (C=N), 142.0 (2C, CH=CH), 135.9, 129.5, 129.2, 128.7, 127.6 (C arom), 100.6 (C-1), 85.0 (C-2), 80.9 (C-4), 74.9 (C-3), 70.3 (C-5), 63.3 (C-6).

#### 2-Deoxy-2-[(*E*)-(4-methoxybenzylidene)­amino]-β-d-galactofuranose (55)

^1^H NMR (500 MHz, DMSO-*d*_6_) δ 8.23 (1H, s, CH=N), 7.71 (2H, d, arom), 7.00 (2H, d, arom), 6.40 (1H, d, *J*_C1,OH_ 6.5 Hz, C1-OH), 5.20 (1H, d, *J*_C3,OH_ 6.0 Hz, C3-OH), 5.16 (1H, t, *J*_1,2_ ≈ *J*_C1,OH_ 5.5 Hz, H-1), 4.16 (1H, c, *J*_2,3_ = *J*_3,4_ 8.0, H-3), 3.90 (1H, dd, *J*_3,4_ 8.5 Hz, *J*_4,5_ 2.0 Hz, H-4), 3.80 (3H, s, CH_3_), 3.60–3.30 (4H, m, H-2, H-5, H-6, H-6′). ^13^C­{^1^H} NMR (125 MHz, DMSO-*d*_6_) δ 161.1 (C=N), 129.5, 129.1, 114.0 (C arom), 100.6 (C-1), 83.8 (C-2), 80.9 (C-4), 74.5 (C-3), 70.3 (C-5), 63.3 (C-6), 55.2 (OCH_3_).

#### 2-[(*E*)-Benzylideneamino]-2-deoxy-β-d-galactofuranose (56)

^1^H NMR (400 MHz, DMSO-*d*_6_) δ 8.32 (1H, s, CH=N), 7.77 (2H, d, arom), 7.47 (2H, d, arom), 6.49 (1H, d, *J*_C1,OH_ 7.6 Hz, C1-OH), 5.27 (1H, d, *J*_C3,OH_ 6.4 Hz, C3-OH), 5.20 (1H, d, *J*_1,2_ ≈ *J*_C1,OH_ 5.8 Hz, H-1), 4.42 (1H, c, *J*_2,3_ = *J*_3,4_ 6.8 Hz, H-3), 3.95 (1H, dd, *J*_3,4_ 6.9 Hz, H-4), 3.60–3.50 (4H, m, H-2, H-5, H-6, H-6^’^). ^13^C­{^1^H} NMR (100 MHz, DMSO-*d*_6_) δ 162.4 (C=N), 136.2 (C arom), 131.1 (C arom), 129.0 (2C arom), 128.4 (C arom), 100.6 (C-1), 83.9 (C-2), 81.0 (C-4), 74.5 (C-3), 70.3 (C-5), 63.3 (C-6).

#### 2-Deoxy-2-[(*E*)-(3-hydroxybenzylidene)­amino]-β-d-galactofuranose (57)

^1^H NMR (500 MHz, DMSO-*d*_6_) δ 9.54 (1H, bs, OH-arom), 8.22 (1H, s, CH=N), 7.24 (1H, d, arom), 7.17 (2H, m, arom), 6.86 (1H, m, arom), 6.44 (1H, d, *J*_C1,OH_ 6.5 Hz, C1-OH), 5.23 (1H, d, *J*_C3,OH_ 6.0 Hz, C3-OH), 5.17 (1H, t, *J*_1,2_ ≈ *J*_C1,OH_ 5.5 Hz, H-1), 4.16 (1H, c, *J*_2,3_ ≈ *J*_3,4_ 8.0 Hz, H-3), 3.90 (1H, dd, *J*_3,4_ 8.0 Hz, *J*_4,5_ 2.0 Hz, H-4), 3.62–3.37 (4H, m, H-2, H-5, H-6, H-6′). ^13^C­{^1^H} NMR (125 MHz, DMSO-*d*_6_) δ 162.5 (C=N), 158.0, 137.8, 130.2, 118.5, 114.3 (C arom), 100.9 (C-1), 84.0 (C-2), 81.3 (C-4), 74.6 (C-3), 70.6 (C-5), 63.5 (C-6).

#### 2-Deoxy-2-[(*E*)-(2,4,6-trimethylbenzylidene)­amino]-β-d-galactofuranose (58)

^1^H NMR (400 MHz, DMSO-*d*_6_) δ 8.52 (1H, s, CH=N), 6.88 (2H, s, H-arom), 6.51 (1H, d, *J*_C1‑OH_ 7.2 Hz, C1-OH), 5.30 (1H, d, *J*_C3‑OH_ 6.4 Hz, C3-OH), 5.20 (1H, t, *J*_1,2_ ≈ *J*_C1‑OH_ 5.4 Hz, H-1), 4.58 (1H, d, *J* 6.4 Hz, OH), 4.18 (1H, c, *J*_2,3_ ≈ *J*_3,4_ 7.4 Hz, H-3), 3.90 (1H, dd, *J*_3,4_ 8.4 Hz, *J*_4,5_ 1.4 Hz, H-4), 3.69–3.41 (4H, m, H-2, H-5, H-6, H-6′). ^13^C­{^1^H} NMR (100 MHz, DMSO-*d*_6_) δ 162.1 (C=N), 138.8, 129.1 (C arom), 100.8 (C-1), 85.2 (C-2), 81.1 (C-4), 74.7 (C-3), 70.4 (C-5), 63.5 (C-6).

#### 2-Deoxy-2-[(*E*)-(1-naphthylmethylene)­amino]-β-d-galactofuranose (59)

^1^H NMR (500 MHz, DMSO-*d*_6_) δ 9.15 (1H, d, H-arom), 8.91 (1H, s, CH=N), 8.04 (1H, d, arom), 7.95 (1H, d, arom), 7.58 (3H, m, arom), 6.53 (1H, d, *J*_C1‑OH_ 6.5 Hz, C1-OH), 5.32 (1H, d, *J*_C3,OH_ 6.0 Hz, C3-OH), 5.31 (1H, t, *J*_1,2_ ≈ *J*_C1,OH_ 5.5 Hz, H-1), 4.29 (1H, c, *J*_2,3_ ≈ *J*_3,4_ 8.0 Hz, H-3), 3.96 (1H, dd, *J*_3,4_ 8.0 Hz, *J*_4,5_ 2.0 Hz, H-4), 3.75–3.30 (4H, m, H-2, H-5, H-6, H-6’). ^13^C­{^1^H} NMR (125 MHz, DMSO-*d*_6_) δ 162.2 (C=N), 133.4, 131.0, 130.9, 129.7, 128.5, 127.2, 126.1, 125.3, 124.6 (C arom), 100.4 (C-1), 84.5 (C-2), 80.7 (C-4), 74.4 (C-3), 70.0 (C-5), 63.0 (C-6).

#### 2-Deoxy-2-[(*E*)-(4-methoxy-1-naphthylmethylene)­amino]-β-d-galactofuranose (60)

^1^H NMR (400 MHz, DMSO-*d*_6_) δ 9.31 (1H, d, *J* 8.5 Hz H-arom), 8.73 (1H, s, CH=N), 8.22 (1H, d, arom), 7.82 (1H, d, arom), 7.62 (1H, t, arom), 7.55 (1H, t, arom), 7.06 (1H, d, arom), 6.48 (1H, d, *J*_C1‑OH_ 5.4 Hz, C1-OH), 5.27 (1H, m, H-1, C3-OH), 4.25 (1H, c, H-3), 3.93 (1H, dd, *J*_3,4_ 8.4 Hz, *J*_4,5_ 1.8 Hz, H-4), 3.74–3.42 (4H, m, H-2, H-5, H-6, H-6′). ^13^C­{^1^H} NMR (100 MHz, DMSO-*d*_6_) δ 162.8 (C=N), 156.8, 132.0, 131.6, 127.7, 125.8, 125.4, 125.2, 124.4, 122.0, 104.2 (C arom), 100.8 (C-1), 84.9 (C-2), 80.9 (C-4), 74.7 (C-3), 70.2 (C-5), 63.4 (C-6), 56.2 (OCH_3_).

#### 2-Deoxy-2-[(*E*)-(2-naphthyl)­methylene]­amino-β-d-galactofuranose (61)

^1^H NMR (400 MHz, DMSO-*d*_6_) δ 8.48 (1H, s, CH = N), 8.25 (1H, s, arom), 8.02 (1H, m, arom), 7.95 (3H, m, arom), 7.56 (2H, m, arom), 6.52 (1H, d, *J*_C1‑OH_ 6.6 Hz, C1-OH), 5.31 (1H, d, *J*_C3‑OH_ 6.3 Hz, C3-OH), 5.25 (1H, t, *J*_1,2_ ≈ *J*_C1,OH_ 5.8 Hz, H-1), 4.23 (1H, c, *J*_2,3_ ≈ *J*_3,4_ ≈ *J*_C3‑OH_ 7.5 Hz, H-3), 3.93 (1H, dd, *J*_3,4_ 7.5 Hz, *J*_4,5_ 1.1 Hz, H-4), 3.70–3.22 (4H, m, H-2, H-5, H-6, H-6′). ^13^C­{^1^H} NMR (100 MHz, DMSO-*d*_6_) δ 162.5 (C=N), 134.4, 133.8, 132.5, 130.3, 129.0, 128.6, 127.7, 127.0, 123.7, 122.6 (C arom), 100.7 (C-1), 84.0 (C-2), 81.0 (C-4), 74.6 (C-3), 70.3 (C-5), 63.3 (C-6).

#### 2-[(*E*)-(9-Anthrylmethylene)­amino]-2-deoxy-β-d-galactofuranose (62)

^1^H NMR (500 MHz, DMSO-*d*_6_) δ 9.45 (1H, s, CH=N), 8.71–8.59 (3H, m, arom), 8.14 (2H, m, arom), 7.59 (4H, m, arom), 6.67 (1H, d, *J*_C1‑OH_ 6.5 Hz, C1-OH), 5.51 (1H, d, *J*_C3,OH_ 6.5 Hz, C3-OH), 5.41 (1H, t, *J*_1,2_ ≈ *J*_C1,OH_ 6.0 Hz, H-1), 4.41 (1H, c, *J*_3,4_ ≈ *J*_2,3_ 8.0 Hz, H-3), 4.04 (1H, dd, *J*_3,4_ 8.0 Hz, *J*_4,5_ 2.0 Hz, H-4), 3.90–3.35 (4H, m, H-2, H-5, H-6, H-6′). ^13^C­{^1^H} NMR (125 MHz, DMSO-*d*_6_) δ 161.2 (C=N), 129.2, 129.2, 128.7, 127.9, 126.8, 125.4 (C arom), 100.2 (C-1), 84.9 (C-2), 80.8 (C-4), 74.2 (C-3), 70.0 (C-5), 63.0 (C-6).

#### 2-Deoxy-2-[(*E*)-(9-phenanthrylmethylene)­amino]-β-d-galactofuranose (63)

^1^H NMR (400 MHz, DMSO-*d*_6_) δ 9.35 (1H, m, arom), 8.92 (1H, m, arom), 8.86 (1H, s, arom), 8.84 (1H, d, arom), 8.31 (1H, s, CH=N), 8.10 (1H, d, arom), 7.73 (4H, m, arom), 6.60 (1H, d, *J*_C1‑OH_ 6.5 Hz, C1-OH), 5.35 (1H, t, *J*_1,2_ ≈ *J*_C1,OH_ 5.8 Hz, H-1), 4.34 (1H, c, *J*_3,4_ ≈ *J*_2,3_ 7.6 Hz, H-3), 3.98 (1H, dd, *J*_3,4_ 7.5 Hz, *J*_4,5_ 1.4 Hz, H-4), 3.75–3.45 (4H, m, H-2, H-5, H-6, H-6′). ^13^C­{^1^H} NMR (100 MHz, DMSO-*d*_6_) δ 163.0 (C=N), 1312, 130.8, 130.4, 129.5, 128.0, 127.5, 127.4, 127.2, 126.1, 123.4, 123.1 (C arom), 100.6 (C-1), 85.0 (C-2), 80.9 (C-4), 74.6 (C-3), 70.3 (C-5), 63.4 (C-6).

## Supplementary Material



## Data Availability

The data underlying this study are available in the published article, in its Supporting Information, and openly available in the institutional repositories: https://dehesa.unex.es/handle/10662/5152 and https://dialnet.unirioja.es/servlet/tesis?codigo=565, respectively.

## Notes

Full spectroscopic and analytical characterization for all compounds reported, crystal data and geometric parameters for compound 38, FT-IR and NMR spectra, and Cartesian coordinates and electronic energies for every stationary point optimized at a given level of theory (PDF)

## Notes

This manuscript is dedicated to the memory of María Dolores Méndez-Cordero.

## References

[1] Peltier P., Euzen R., Daniellou R., Nugier-Chauvin C., Ferrières V. (2008). Recent knowledge and innovations related to hexofuranosides:
structure, synthesis and applications. Carbohydr.
Res..

[2] Nassau P. M., Martin S. L., Brown R. E., Weston A., Monsey D., McNeil M. R., Duncan K. (1996). Galactofuranose biosynthesis
in *Escherichia coli* K-12: identification and cloning
of UDP-galactopyranose mutase. J. Bacteriol..

[3] Brennan P. J., Nikaido H. (1995). The envelope of mycobacteria. Annu. Rev. Biochem..

[4] Ridley B. L., O’Neill M. A., Mohnen D. (2001). Pectins: structure, biosynthesis,
and oligogalacturonide-related signaling. Phytochemistry.

[5] Schmalhorst P. S., Krappmann S., Vervecken W., Rohde M., Müller M., Braus G. H., Contreras R., Braun A., Bakker H., Routier F. H. (2008). Contribution of galactofuranose to the virulence of
the opportunistic pathogen Aspergillus fumigatus. Eukaryot. Cell.

[6] Ho J. S., Gharbi A., Schindler B., Yeni O., Brédy R., Legentil L., Ferrières V., Kiessling L. L., Compagnon I. (2021). Distinguishing galactoside isomers with mass spectrometry
and gas-phase infrared spectroscopy. J. Am.
Chem. Soc..

[7] Irvine J. C., Hynd A. (1913). VIII.-Synthetical aminoglucosides
derived from d-glucosamine. J. Chem. Soc..

[8] Neuberger A. (1938). Carbohydrates
in protein: the carbohydrate component of crystalline egg albumin. Biochem, J..

[9] Morgan W. T. J. (1938). Isolierung von D-Galaktose und L-Rhamnose aus dem Hydrolysat
des spezifischen Polysaccharids von *Bact. dysenteriae* (Shiga). Helv. Chim. Acta.

[10] Marbet R., Winterstein A. (1951). Probleme der
Blutgerinnung. 4. Mitteilung. β-Heparin,
ein neuer, blutgerinnungshemmender Mucoitinschwefelsäureester. Helv. Chim. Acta.

[11] Arcamone F., Bizioli F. (1957). Isolation and constitution of trehalosamine, a new
amino-sugar from a streptomyces. Gazz. Chim.
Ital..

[12] Jeanloz R. W. (1952). 3,4-Dimethyl-D-glucosamine
hydrochloride
and derivatives. J. Am. Chem. Soc..

[13] Fostes A. B., Stacey M., Vardheim S. V. (1959). Aminosugars and related compounds.
Part VI. The action of alkali on some benzyloxycarbonylamino derivatives. Acta Chem. Scand..

[14] Ludowieg J. J., Benmaman J. D. (1968). A method for analysis
of amino sugars: specificity
and mechanism of the reaction. Carbohyd. Res..

[15] Serafini-Cessi F., Cessi C. (1970). Isolation of some precursors
of 2-methylpyrrole in the Elson-Morgan
reaction. Biochem. J..

[16] Ávalos, M. ; Babiano, R. ; Cintas, P. ; Hursthouse, M. B. ; Jiménez, J. L. ; Light, M. E. ; Palacios, J. C. ; Pérez, E. M. S. Synthesis of sugar isocyanates and their application to the formation of ureido-linked disaccharides. Eur. J. Org. Chem. 2006, 2006 657–671. 10.1002/ejoc.200500601

[17] Horton, D. ; Wander, J. D. Amino Sugars. In The Carbohydrates. Chemistry and Biochemistry, Vol. 1B; Pigman, W. ; Horton, D. ; Wander, J. D. , Eds.; Academic Press: New York, 1980; pp 643–760.

[18] Matamoros E., Perez E. M. S., Light M. E., Cintas P., Martinez R. F., Palacios J. C. (2024). A true reverse anomeric
effect does exist after all: A hydrogen bonding stereocontroling effect
in 2-iminoaldoses. J. Org. Chem..

[19] Richards M. R., Lowary T. L. (2009). Chemistry and biology
of galactofuranose-containing
polysaccharides. ChemBioChem..

[20] Bock K., Pedersen C. (1983). Carbon-13 nuclear magnetic
resonance spectroscopy of monosaccharides. Adv.
Carbohydr. Chem. Biochem..

[21] Bock K., Pedersen C. (1974). A study of ^13^C-H coupling
constants in hexopyranoses. J.
Chem. Soc., Perkin Trans..

[22] Tvaroska I., Taravel F. R. (1995). Carbon-proton coupling
constants in the conformational
análisis of sugar molecules. Adv. Carbohydr.
Chem. Biochem..

[23] Crystal data for compound **38** have been deposited with the Cambridge Crystallographic Data Centre (CCDC-1845143) and can be obtained, upon request, from the authors or the Director, Cambridge Crystallographic Data Centre, 12 Union Road, Cambridge CBL 1EZ, UK.

[24] Fülöp F., Bernáth G., Mattinen J., Pihlaja K. (1989). Ring-chain tautomerism
of 1,3-oxazolidines
prepared from norephedrine and norpseudoephedrine. Tetrahedron.

[25] Fülöp F., Lázár L., Bernáth G., Sillanpää R., Pihlaja K. (1993). Substituent
effects on the ring-chain tautomerism of 1,3-oxazines. Tetrahedron.

[26] Angyal S. J. (1969). The composition and conformation
of sugars in solution. Angew. Chem., Int. Ed.
Engl..

[27] Maireanu C., Darabantu M., Plé G., Berghian C., Condamine E., Ramondenc Y., Silaghi-Dumitrescu I., Mager S. (2002). Ring-chain tautomerism
and other versatile behaviour of 1,4-diimino- and 1,2-phenylene derivatives
of some *C*-substituted serinols. Tetrahedron.

[28] Stoddart, J. F. Stereochemistry of Carbohydrates; John Wiley & Sons, Inc.: New York, 1971; ch 4.

[29] King-Morris M. J., Serianni A. S. (1987). Carbon-13 NMR studies
of [1-^13^C]­aldoses:
empirical rules correlating pyranose ring configuration and conformation
with carbon-13 chemical shifts and carbon-13/carbon-13 spin couplings. J. Am. Chem. Soc..

[30] Mackie W., Perlin A. S. (1966). Pyranose-furanose and anomeric equilibria: influence
of solvent and of partial methylation. Can.
J. Chem..

[31] Edward J. T. (1955). Stability
of glycosides to acid hydrolysis. Chem. Ind..

[32] a Kirby, A. J. The Anomeric Effect and Related Stereoelectronic Effects at Oxygen; Springer-Verlag: Berlin, 1983.

[33] Ryan G., Utley H. P., Jones H. F. (1988). Electro-organic
reactions. Part 33.
Reduction of sugar oximes. Tetrahedron Lett..

[34] Eliel E. L., Giza C. A. (1968). Conformational analysis.
XVII. 2-Alkoxy- and 2-alkylthiotetrahydropyrans
and 2-alkoxy-1,3-dioxanes. Anomeric effect. J. Org. Chem..

[35] Horton D., Jewell J. S., Philips K. D. (1966). Anomeric
Equilibria in Derivatives of Amino Sugars. Some 2-Amino-2-deoxy-D-hexose
Derivatives. J. Org. Chem..

[36] Jungius C. L. (1905). Isomeric
changes of some dextrose derivatives and the mutarotation of sugars. Z. Phys. Chem..

[37] Eliel, E. L. ; Wilen, S. H. Stereochemistry of Organic Compounds; Wiley-Interscience: New York, NY, 1994; pp 749–753.

[38] a Deslongchamps, P. Stereoelectronic Effects in Organic Chemistry; Pergamon Press: Oxford, 1983.

[39] Tvaroska I., Bleha T. (1989). Anomeric and exo-anomeric
effects in carbohydrate chemistry. Adv. Carbohydr.
Chem. Biochem..

[40] d Romers, C. ; Altona, C. ; Buys, H. R. ; Havinga, E. Geometry and conformational properties of some five- and six-membered heterocyclic compounds containing oxygen and sulfur. In Topics in Stereochemistry, Vol. 4; Eliel, E. L. ; Allinger, N. L. , Eds.; John Wiley & Sons, Inc.: New York, 1969; pp 39–97.

[41] Box V. G. S. (1990). The role of lone pair interactions
in the chemistry of the monosaccharides anomeric effect. Heterocycles.

[42] Mo Y. (2010). Computational evidence that hyperconjugative
interactions are not responsible for the anomeric effect. Nat. Chem..

[43] Cocinero E. J., Carçabal P., Vaden T. D., Simons J. P., Davis B. G. (2011). Sensing
the anomeric effect in a solvent-free environment. Nature.

[44] Lemieux R. U. (1971). Effects of unshared pairs of electrons
and their solvation on conformational equilibria. Pure Appl. Chem..

[45] Freitas M.
P. (2013). The anomeric effect
on the basis
of natural bond orbital analysis. Org. Biomol.
Chem..

[46] Alabugin I.
V., Kuhn L., Krivoshchapov N. V., Mehaffy P., Medvedev M. G. (2021). Anomeric effect,
hyperconjugation
and electrostatics: lessons from complexity in a classic stereoelectronic
phenomenon. Chem. Soc. Rev..

[47] Lemieux R. U., Morgan A. R. (1965). The abnormal conformations of pyridinium α-glycopyranosides. Can. J. Chem..

[48] Wolfe S., Whangbo M.-H., Mitchell D. J. (1979). On the
magnitudes and origins of the “anomeric effects”, “exo-anomeric
effects”, “reverse anomeric effects”, and the
C-X and C-Y bond-lengths in XCH_2_YH molecules. Carbohydr. Res..

[49] Perrin C. L., Fabian M. A., Brunckova J., Ohta B. K. (1999). Absence of reverse anomeric effect in glycosylimidazoles. J. Am. Chem. Soc..

[50] Finch P., Nagpurkar A. G. (1976). The reverse anomeric effect: further observations on *N*-glycosylimidazoles. Carbohydr. Res..

[51] Chan S. S.
C., Szarek W. A., Thatcher G. R. J. (1995). The
reverse anomeric effect in *N*-pyranosylimidazolides:
a molecular orbital study. J. Chem. Soc., Perkin
Trans..

[52] Jones, P. G. ; Komarov, I. V. ; Wothers, P. D. A test for the reverse anomeric effect. Chem. Commun. 1998, 1695–1696. 10.1039/a804354j

[53] Gómez-Sánchez A., Borrachero Moya P., Bellanato J. (1984). Protection of the amino group of
amino sugars by the acylvinyl group: Part I, glycoside formation by
the Fischer reaction. Carbohydr. Res..

[54] Bergmann M., Zervas L. (1931). Synthesen mit Glucosamin. Ber.
Deuts. Chem. Ges..

[55] Morel C. J. (1958). Über die Darstellung und Eigenschaften
von 2,6-Didesoxy-2-amino-D-glucose (6-Desoxy-D-glucosamin). Helv. Chim. Acta.

[56] Wacker O., Fritz H. (1967). Zur Synthese von D-Glucopyrano-(*cis*-2’,1’-*c*)-1,2,3,4-tetrahydro-isochinolinen. Helv. Chim. Acta.

[57] Kuhn R., Kirschenlohr W. (1956). 2-Amino-2-desoxy-zucker durch katalytische
Halbhydrierung
von Amino-, Arylamino- und Benzylamino-nitrilen; D- und L-Glucosamin. Aminozucker-synthesen II. Liebigs Ann. Chem..

[58] Ávalos M., Babiano R., Cintas P., Jiménez J. L., Palacios J. C., Fuentes J. (1990). Synthesis of acylated
thioureylenedisaccharides. J. Chem. Soc., Perkin
Trans..

[59] Durette P. L., Horton D. (1971). Conformational analysis of sugars and their derivatives. Adv. Carbohydr. Chem..

[60] Anet F. A. L. (1962). The
use of remote deuteration for the determination of coupling constants
and conformational equilibria in cyclohexane derivatives. J. Am. Chem. Soc..

[61] Angyal S. J. (1984). The composition
of reducing sugars in solution. Adv. Carbohydr.
Chem. Biochem..

[62] Gaweda K., Plazinski W. (2020). The *endo*- and *exo*-anomeric effects in furanosides. A computational study. Eur. J. Org. Chem..

[63] Marenich A. V., Cramer C. J., Truhlar D. G. (2009). Universal solvation model based on
solute electron density and on a continuum model of the solvent defined
by the bulk dielectric constant and atomic surface tensions. J. Phys. Chem. B.

[64] Frisch, M. J. ; Trucks, G. W. ; Schlegel, H. B. ; Scuseria, G. E. ; Robb, M. A. ; Cheeseman, J. R. ; Scalmani, G. ; Barone, V. ; Mennucci, B. ; Petersson, G. A. ; Nakatsuji, H. ; Caricato, M. ; Li, X. ; Hratchian, H. P. ; Izmaylov, A. F. ; Bloino, J. ; Zheng, G. ; Sonnenberg, J. L. ; Hada, M. ; Ehara, M. ; Toyota, K. ; Fukuda, R. ; Hasegawa, J. ; Ishida, M. ; Nakajima, T. ; Honda, Y. ; Kitao, O. ; Nakai, H. ; Vreven, T. ; Montgomery, J. A. Jr. ; Peralta, J. E. ; Ogliaro, F. ; Bearpark, M. ; Heyd, J. J. ; Brothers, E. ; Kudin, K. N. ; Staroverov, V. N. ; Kobayashi, R. ; Normand, J. ; Raghavachari, K. ; Rendell, A. ; Burant, J. C. ; Iyengar, S. S. ; Tomasi, J. ; Cossi, M. ; Rega, N. ; Millam, J. M. ; Klene, M. ; Knox, J. E. ; Cross, J. B. ; Bakken, V. ; Adamo, C. ; Jaramillo, J. ; Gomperts, R. ; Stratmann, R. E. ; Yazyev, O. ; Austin, A. J. ; Cammi, R. ; Pomelli, C. ; Ochterski, J. W. ; Martin, R. L. ; Morokuma, K. ; Zakrzewski, V. G. ; Voth, G. A. ; Salvador, P. ; Dannenberg, J. J. ; Dapprich, S. ; Daniels, A. D. ; Farkas, Ö ; Foresman, J. B. ; Ortiz, J. V. ; Cioslowski, J. ; Fox, D. J. Gaussian 09, Revision A.1; Gaussian, Inc.: Wallingford, CT, 2009.

[65] McLean A. D., Chandler G. S. (1980). Contracted Gaussian
basis sets for molecular calculations. I. Second row atoms, Z = 11–18. J. Chem. Phys..

[66] Weigend F., Ahlrichs R. (2005). Balanced basis sets of split valence, triple zeta valence
and quadruple zeta valence quality for H to Rn: design and assessment
of accuracy. Phys. Chem. Chem. Phys..

[67] Becke A. D. (1993). Density-functional thermochemistry.
III. The role of exact exchange. J. Chem. Phys..

[68] Zhao Y., Truhlar D. G. (2008). The M06 suite of
density functionals for main group
thermochemistry, thermochemical kinetics, noncovalent interactions,
excited states, and transition elements: two new functionals and systematic
testing of four M06-class functionals and 12 other functionals. Theor. Chem. Acc..

[69] a Parr, R. G. ; Yang, W. Density-Functional Theory of Atoms and Molecules; Oxford University Press: Oxford, 1989.

[70] Glendening, E. D. ; Reed, A. E. ; Carpenter, J. A. ; Weinhold, F. NBO 3.1; University of Wisconsin: Madison, 2001.

[71] Stoddart, J. F. Stereochemistry of Carbohydrates; John Wiley & Sons, Inc.: New York, 1971; pp 66–98.

[72] Musin R. N., Mariam Y. H. (2006). An integrated approach to the study of intramolecular
hydrogen bonds in malonaldehyde enol derivatives and anphthazarin:
trend in energetic versus geometrical consequences. J. Phys. Org. Chem..

[73] Reed A. E., Weinstock R. B., Weinhold F. (1985). Natural population
analysis. J. Chem. Phys..

[74] Bohlmann F. (1957). Zur Konfigurationsbestimmung von
Chinolizin-Derivaten. Angew. Chem..

[75] Perlin A. S., Casu B. (1969). Carbon-13 and proton
magnetic resonance spectra of D-glucose-^13^C. Tetrahedron Lett..

[76] Appell M., Strati G., Willett J. L., Momamy F. A. (2004). B3LYP/6–311++G** study of α- and β-D-glucopyranose
and 1,5-anhydro-D-glucitol: ^4^C_1_ and ^1^C_4_ chairs, ^3,O^B and B_3,O_ boats,
and skew-boat conformations. Carbohydr. Res..

[77] Csonka G. I., French A. D., Johnson G. P., Stortz C. A. (2009). Evaluation of density
functionals and basis sets for carbohydrates. J. Chem. Theory Comput..

[78] St.
John P. C., Kim Y., Kim S., Paton R. S. (2020). Prediction
of organic homolytic bond dissociation enthalpies and near chemical
accuracy with sub-second computational cost. Nat. Commun..

[79] Turner J. A., Adrianov T., Zakaria M. A., Taylor M. S. (2022). Effects of configuration
and substitution on C–H bond dissociation enthalpies in carbohydrate
derivatives: A systematic computational study. J. Org. Chem..

[80] Hooft, R. W. W. COLLECT: Data Collection Software; Nonius BV: Delft, The Netherlands, 1998.

[81] Otwinowski, Z. ; Minor, W. Methods in Enzymology, Vol. 276: Macromolecular Crystallography, Part A; Carter, C. W., Jr. ; Sweet, R. M. , Eds.; Academic Press: New York, NY, 1997; pp 307–326. 10.1016/S0076-6879(97)76066-X 27754618

[82] Sheldrick, G. M. SADABS-Bruker Nonius Area Detector Scaling and Absorption Correction-v2.10; SADABS, 1993.

[83] Sheldrick, G. M. SHELX97: Programs for Crystal Structure Analysis (Release 97–2); Göttingen Universität: Göttingen, Germany, 1998.

[84] Watkin, D. M. ; Pearce, L. ; Prout, C. K. CAMERON. A Molecular Graphics Package; University of Oxford: Oxford, UK, 1993.

