# Supplementary material for: Reverse Anomeric Effects in Pyranose and Furanose Isomers in Schiff Bases of d‑Galactosamine
Source: J Org Chem. 2025 Sep 17;90(38):13374–98. doi: 10.1021/acs.joc.5c00796 (PMC12481579; doi:10.1021/acs.joc.5c00796)
Supplement: Supplementary file 1 [file jo5c00796_si_001.pdf]

Supporting Information

Reverse Anomeric Effects in Pyranose and Furanose Isomers in Schiff Bases of D-Galactosamine

Esther Matamoros,[a,b,c,*] Esther M. S. Pérez,[a] Pedro Cintas,[a] Mark E. Light,[d] and Juan C. Palacios[a,*]

[a]*Departamento de Química Orgánica e Inorgánica, Facultad de Ciencias, and Instituto del Agua, Cambio Climático y Sostenibilidad (IACYS), Universidad de Extremadura, 06006 Badajoz, Spain*
[b]*Departamento de Química Orgánica, Universidad de Málaga, Campus Teatinos s/n, 29071 Málaga, Spain*
[c]*Instituto de Investigación Biomédica de Málaga y Plataforma en Nanomedicina – IBIMA, Plataforma Bionand, Parque Tecnológico de Andalucía, 29590 Málaga, Spain*
[d]*Department of Chemistry, Faculty of Natural and Environmental Sciences, University of Southampton, Southampton SO17 1BJ, UK*

Table of Contents

Tables S1-S23 and Figure S1 Pages S2-S13
IR Spectra (Figures S2-S17) Pages S14-S21
NMR Spectra (Figures S18-S82) Pages S22-S54
Cartesian Coordinates and Calculated Energies at the B3LYP/6-31G(d,p), M06-2X/6-311G(d,p) and M06-2X/def2-TZVP Level in Gas Phase, $CHCl_3$, DMSO and water (SMD Model) Pages S55-S173

Table S1. ^{1}H NMR Data (δ, ppm) for Compounds 21 and 27-36[a]

Compound	ArCH	C1OH	H-1	H-2	H-3	H-4	H-5	H-6	H-6′
**21**[b]	7.95 d	6.49 d	4.58 m	3.02 dd	3.58 m	3.66 t	3.43 t	3.58 m	3.52 dd
**27**[b]	8.05 s	6.44 bs	4.63 d	3.06 dd	3.59 m	3.66 d	3.46 t	3.59 m	3.52 dd
**28**[c]	8.13 s	6.43 d	4.63 t	3.09 dd	3.58 m	3.67 s	3.46 t	3.58 m	3.53 m
**29**[b]	8.21 s	6.49 d	4.68 d	3.15 t	3.59 m	3.68 t	3.49 t	3.59 m	3.52 dd
**30**[c]	8.11 s	6.45 d	4.63 m	3.11 dd	3.58 m	3.67 s	3.46 t	3.58 m	3.58 m
**31**[b]	8.41 s	6.47 d	4.65 m	3.11 t	3.56 m	3.67 t	3.45 t	3.59 dd	3.51 dd
**32**[c]	8.84 s	6.54 d	4.77 t	3.27 dd	3.62 m	3.73 t	3.53m	3.67 m	3.57 m
**33**[b]	8.67 s	6.50 d	4.74 t	3.19 t	3.64 m	3.64	3.52 m	3.64 m	3.64 m
**34**[b]	8.39 s	6.56 d	4.73 t	3.24 t	3.60 m	3.72	3.60 m	3.60 m	3.60 m
**35**[c]	9.29 s	6.77 d	4.84 t	3.56 m	3.76 m	3.80	3.56 m	3.66 d	3.62 m
**36**[b]	8.23 s	6.61 d	4.82 t	3.32 t	3.58 m	3.76	3.58 m	3.58 m	3.58 m

[a]In DMSO-d_6. [b]At 400 MHz. [c]At 500 MHz.

Table S2. Coupling Constants (Hz) for Compounds 21 and 27-36[a]

Compound	$J_{1,2}$	$J_{2,3}$	$J_{3,4}$	$J_{4,5}$	$J_{5,6}$	$J_{5,6'}$	$J_{6,6'}$	$J_{C1,OH}$
**21**[b]	7.7	9.6	3.8	0	6.1	6.1	10.7	6.9
**27**[b]	7.5	9.4	3.1	0	6.0	6.2	10.7	--
**28**[c]	7.0	9.5	--	0	6.0	6.0	10.0	6.5
**29**[b]	7.3	8.6	3.6	0	6.1	6.1	10.7	6.8
**30**[c]	8.0	9.5	--	0	6.5	6.5	--	7.0
**31**[b]	7.4	8.6	3.6	0	5.6	6.0	11.2	6.8
**32**[c]	7.5	9.5	3.5	0	5.0	6.0	11.0	7.0
**33**[b]	7.3	8.6	--	0	5.7	--	--	7.0
**34**[b]	7.3	8.6	--	0	--	--	--	6.9
**35**[c]	7.5	--	--	0	--	--	--	7.5
**36**[b]	7.1	8.5	--	0	--	--	--	7.0

[a]In DMSO-d_6. [b]At 400 MHz. [c]At 500 MHz.

Table S3. ^{13}C NMR Data (δ, ppm) for Compounds 21 and 27-36[a]

Compound	ArCHN	C-1	C-2	C-3	C-4	C-5	C-6
**21**[b]	163.93	96.32	74.87	71.87	67.40	75.44	60.98
**27**[b]	162.10	96.39	74.72	71.79	67.53	75.38	61.01
**28**[c]	161.10	96.03	74.67	71.50	67.12	75.03	60.63
**29**[b]	162.35	96.31	74.86	71.76	67.44	75.44	61.01
**30**[c]	162.44	96.49	74.87	71.94	67.62	75.57	61.17
**31**[b]	162.07	96.41	75.43	71.73	67.48	75.88	60.99
**32**[c]	161.91	96.10	75.12	71.58	67.17	75.36	60.68
**33**[b]	162.50	96.56	75.44	72.07	67.55	75.74	61.01
**34**[b]	162.84	96.46	75.08	71.97	67.66	75.61	61.20
**35**[c]	161.29	96.07	75.25	71.49	67.29	75.92	60.73
**36**[b]	162.76	96.43	75.48	71.93	67.49	75.87	61.00

[a]In DMSO-d_6. [b]At 100 MHz. [c]At 125 MHz.

Table S4. ^{1}H NMR Data (δ, ppm) for Compounds 22 and 37-44[a]

Compd.	Ar	H-1	H-2	H-3	H-4	H-5	H-6	H-6′	NH
**22**	8.06	5.88 d	3.55 dd	5.20 dd	5.46 d	4.19 m	4.19 m	4.19 m	---
**37**	9.95	5.94 d	3.65 t	5.26 dd	5.46 d	4.17 m	4.17 m	4.17 m	---
**38**	8.30	5.95 d	3.65 t	5.27 dd	5.47 d	4.19 m	4.19 m	4.19 m	---
**39**	8.21	5.93 d	3.61 t	5.25 dd	5.47 d	4.19 m	4.19 m	4.19 m	---
**40**	8.30	5.71 d	4.46 c	5.09dd	5.38 d	4.03 c	4.16 m	4.16 m	5.50
**41**	8.23	5.70 d	4.48 c	5.08 dd	5.37 d	4.16 m	4.16 m	4.16 m	5.54
**42**	8.79	6.04 d	3.70 dd	5.36 dd	5.51 d	4.22 m	4.22 m	4.22 m	---
**43**	8.35	6.00 d	3.73 dd	5.32 dd	5.49 d	4.19 m	4.19 m	4.19 m	---
**44**	9.49	6.13 d	4.05 dd	5.47 dd	5.59 d	4.26 m	4.26 m	4.26 m	---

[a]In $CDCl_3$ at 400 MHz.

Table S5. Coupling Constants (Hz) for Compounds 22 and 37-44[a]

Compd.	$J_{1,2}$	$J_{2,3}$	$J_{3,4}$	$J_{4,}$	$J_{5,6}$	$J_{NH,2}$
**22**	8.2	10.3	3.2	0	--	--
**37**	8.2	10.5	3.3	0	--	--
**38**	8.3	10.4	3.3	0	--	--
**39**	8.2	10.3	3.3	0	--	--
**40**	9.9	11.3	3.0	0	5.5	10.1
**41**	8.7	11.2	3.1	0	--	9.5
**42**	8.4	10.2	3.2	0	---	---
**43**	8.2	10.3	3.3	0	---	---
**44**	8.3	10.4	3.2	0	--	--

[a]In $CDCl_3$ at 400 MHz.

Table S6. ^{13}C NMR Data (δ, ppm) for Compounds 22 and 37-44[a]

Compd.	ArCH	C-1	C-2	C-3	C-4	C-5	C-6
**22**	167.05	93.32	71.69	68.81	65.79	71.48	61.22
**37**	164.26	93.29	71.75	68.78	65.81	71.42	61.26
**38**	165.27	93.38	71.77	68.84	65.86	71.43	61.28
**39**	164.44	93.50	71.73	68.78	65.93	71.54	61.30
**40**	165.27	93.00	71.77	70.26	66.30	71.43	61.28
**41**	164.26	93.02	71.83	70.30	66.29	71.41	61.26
**42**	165.10	93.59	71.78	69.72	65.99	71.69	61.32
**43**	165.41	93.41	71.78	68.99	65.90	71.48	61.32
**44**	165.90	93.35	71.93	70.54	65.88	71.69	61.26

[a]In $CDCl_3$ at 100 MHz.

Table S7. Crystal data and structure refinement of compound 38

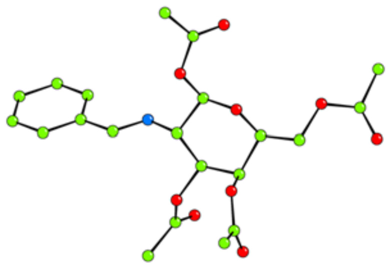

Identification code	**03ESP0201**
Empirical formula	$C_{21}H_{25}NO_9$
Formula weight	435.42
Temperature	120(2) K
Wavelength	0.71073 Å
Crystal system	Monoclinic
Space group	$P2_1$
Unit cell dimensions	a = 5.3534(3) Å
	b = 17.7575(8) Å β = 94.967(2)°
	c = 11.4880(6) Å
Volume	1087.98(10) Å^3
Z	2
Density (calculated)	1.329 Mg / m^3
Absorption coefficient	0.105 mm^{-1}
F(000)	460
Crystal	Colourless block
Crystal size	0.30 × 0.30 × 0.20 mm^3
θ range for data collection	3.56 – 25.03°
Index ranges	$-6 \le h \le 6$, $-21 \le k \le 20$, $-13 \le l \le 13$
Reflections collected	10575
Independent reflections	3837 [R_{int} = 0.0507]
Completeness to θ = 25.03°	99.8 %
Absorption correction	Semi–empirical from equivalents
Max. and min. transmission	0.9794 and 0.9693
Refinement method	Full-matrix least-squares on F^2
Data / restraints / parameters	3837 / 1 / 285
Goodness-of-fit on F^2	1.030
Final R indices [$F^2 > 2\sigma(F^2)$]	$R1$ = 0.0334, $wR2$ = 0.0785
R indices (all data)	$R1$ = 0.0406, $wR2$ = 0.0818
Absolute structure parameter	0.0(7)
Extinction coefficient	0.019(3)
Largest diff. peak and hole	0.149 and –0.171 e Å^{-3}

Diffractometer: *Nonius KappaCCD* area detector (ϕ scans and ω scans to fill *asymmetric unit* sphere). **Cell determination:** DirAx (Duisenberg, A.J.M.(1992). J. Appl. Cryst. 25, 92-96.) **Data collection:** Collect (Collect: Data collection software, R. Hooft, Nonius B.V., 1998). **Data reduction and cell refinement**: *Denzo* (Z. Otwinowski & W. Minor, *Methods in Enzymology* (1997) Vol. **276**: *Macromolecular Crystallography*, part A, pp. 307–326; C. W. Carter, Jr. & R. M. Sweet, Eds., Academic Press). **Absorption correction**: *SORTAV* (R. H. Blessing, Acta Cryst. A**51** (1995) 33–37; R. H. Blessing, J. Appl. Cryst. **30** (1997) 421–426). **Structure solution**: *SHELXS97* (G. M. Sheldrick, Acta Cryst. (1990) A**46** 467–473). **Structure refinement**: *SHELXL97* (G. M. Sheldrick (1997), University of Göttingen, Germany). **Graphics:** Cameron - A Molecular Graphics Package. (D. M. Watkin, L. Pearce and C. K. Prout, Chemical Crystallography Laboratory, University of Oxford, 1993).

Special details: All hydrogen atoms were placed in idealised positions and refined using a riding model.

Table S8. ^{13}C NMR Data (δ, ppm) for Compounds a-d Detected after Equilibration in Solution from imines 21, 29 and 31[a]

	Compound	ArCHN	C-1	C-2	C-3	C-4	C-5	C-6
**21**	**a**	163.97	96.35	74.91	71.88	67.41	75.46	61.01
	**b**	163.97	100.61	84.04	74.55	80.92	70.25	63.30
	**c**	---	93.54	70.95	67.74	68.35	70.95	61.01
	**d**	164.14	97.25	79.10	73.59	83.23	71.10	63.03
**29**	**a**	162.27	96.28	74.83	71.73	67.41	75.41	60.96
	**b**	162.27	100.58	83.90	74.41	80.87	70.21	63.27
	**c**	162.42	93.50	70.97	67.62	68.34	70.78	61.04
	**d**	162.54	97.27	78.77	73.86	83.24	70.97	63.00
**31**	**a**	162.44	96.53	75.97	71.88	67.66	75.58	61.17
	**b**	162.13	100.81	85.16	74.67	80.10	70.36	63.48
	**c**	162.44	93.99	72.03	67.78	68.54	71.09	61.17
	**d**	162.44	97.51	80.01	74.12	83.40	71.27	63.20

[a]In DMSO-d_6 at 100 MHz.

Table S9. ^{1}H NMR Data (δ, ppm) for Compounds 53-63[a]

Compd.	ArCHN	C1OH	H-1	H-2	H-3	H-4	H-5	H-6	H-6′
**53**[b]	8.05 d	6.47 d	5.13 t	3.52 m	4.13 c	3.88 dd	3.52	3.52 m	3.52 m
**54**[b]	8.16 s	--	5.17 d	3.65 m	4.16 t	3.89 dd	3.65	3.65 m	3.65 m
**55**[c]	8.23 s	6.40 d	5.16 t	3.50 m	4.16 c	3.90 dd	3.50	3.50 m	3.50 m
**56**[b]	8.32 s	6.49 d	5.20 t	3.65 m	4.19 c	3.92 dd	3.60	3.65 m	3.65 m
**57**[c]	8.22 s	6.44 d	5.17 t	3.50 m	4.16 c	3.90 dd	3.50	3.50 m	3.50 m
**58**[b]	8.52 s	6.51 d	5.20 t	3.55 m	4.18 c	3.90 dd	3.55	3.55 m	3.55 m
**59**[c]	8.91 s	6.53 d	5.31 t	3.52 m	4.29 c	3.96 dd	3.52	3.52 m	3.52 m
**60**[b]	8.73 s	6.48 d	5.27	3.55 m	4.25 c	3.93 dd	3.55	3.55 m	3.55 m
**61**[b]	8.48 s	6.52 d	5.25 t	3.46 m	4.23 c	3.93 dd	3.46	3.46 m	3.46 m
**62**[c]	9.45 s	6.67 d	5.41 t	3.63 m	4.41 c	4.04 dd	3.63	3.63 m	3.63 m
**63**[b]	8.31 s	6.60 d	5.35 t	3.55 m	4.34 c	3.98 dd	3.55	3.55 m	3.55 m

[a]In DMSO-d_6. [b]At 400 MHz. [c]At 500 MHz.

Table S10. Coupling Constants (Hz) for Compounds 53-63[a]

Compound	$J_{1,2}$	$J_{2,3}$	$J_{3,4}$	$J_{4,5}$	$J_{C1,OH}$
**53**[b]	5.8	7.5	8.4	1.8	6.1
**54**[b]	5.6	8.0	8.0	1.6	--
**55**[c]	5.5	8.0	8.5	2.0	6.5
**56**[b]	5.8	6.8	6.9	--	7.6
**57**[c]	5.5	8.0	8.0	2.0	6.5
**58**[b]	5.4	7.4	8.4	1.4	7.2
**59**[c]	5.5	8.0	8.0	2.0	6.5
**60**[b]	--	--	8.4	1.8	5.4
**61**[b]	5.8	7.5	7.5	1.1	6.6
**62**[c]	6.0	8.0	8.0	2.0	6.5
**63**[b]	5.8	7.6	7.5	1.4	6.5

[a]In DMSO-d_6. [b]At 400 MHz. [c]At 500 MHz.

Table S11. ^{13}C **NMR Data (δ, ppm) for Compounds 53-65**[a]

Compound	ArCHN	C-1	C-2	C-3	C-4	C-5	C-6
**53**[b]	163.97	100.61	84.95	74.90	80.92	70.25	63.30
**54**[b]	162.10	100.69	83.75	74.52	80.95	71.00	63.33
**55**[c]	161.10	100.36	83.43	74.26	80.71	70.04	62.98
**56**[b]	162.35	100.61	83.89	74.47	80.95	70.28	63.30
**57**[c]	162.49	100.89	83.97	74.15	81.28	70.59	63.54
**58**[b]	162.13	100.81	85.16	74.67	81.10	70.36	63.48
**59**[c]	162.20	100.42	84.48	74.38	80.70	69.98	63.04
**60**[b]	162.79	100.82	84.90	74.69	80.91	70.24	63.36
**61**[b]	162.47	100.66	83.98	74.55	80.98	70.27	63.33
**62**[c]	161.16	100.22	84.85	74.17	80.79	69.98	63.02
**63**[b]	163.04	100.64	84.97	74.64	80.94	70.25	63.35
**64**[b]	--	101.8	82.2	76.6	82.8	71.5	63.6
**65**[b]	--	95.8	77.1	75.1	81.6	--	63.3

[a]In DMSO-d_6. [b]At 400 MHz. [c]At 500 MHz.

Table S12. ^{13}C **NMR Data (δ, ppm) for Compounds 65, 67, 68-70, 72, 75, 77, 78, and 81-84**[a]

Compound	C-1	C-2	C-3	C-4	C-5	C-6
**65**[b]	95.8	77.1	75.1	81.6	--	63.3
**67**[b]	93.99	72.03	67.78	68.54	71.09	61.17
**68**[c]	93.26	70.60	67.37	68.04	70.27	60.72
**69**[b]	93.50	70.47	67.62	68.34	70.78	61.04
**70**[c]	93.76	71.16	67.88	68.62	70.59	61.31
**72**[c]	93.37	71.42	67.55	68.12	70.67	60.80
**75**[c]	93.42	71.77	67.51	68.24	70.68	60.81
**77**[b,d]	92.2	51.4	68.6	69.7	71.6	62.4
**78**[b,d]	96.5	54.9	72.3	69.0	76.3	62.2
**81**[c]	97.00	78.27	70.87	82.97	69.96	62.72
**82**[b]	92.27	78.77	73.86	83.24	72.00	63.00
**83**[c]	97.52	78.90	74.15	83.54	71.40	63.29
**84**[b]	97.51	80.01	74.12	83.40	71.27	63.20
**85**[c]	97.05	79.48	73.61	82.99	70.84	62.77
**88**[c]	97.06	--	73.49	83.07	--	62.79

[a]In DMSO-d_6. [b]At 100 MHz. [c]At 125 MHz. [d]Reference 22.

Table S13. Relative Stabilities (kcal/mol) of All Species Involved in the Mutarotation of 29

	Entry	Conformer[a]	Gas phase[b]		DMSO[b]		Gas phase[c]		DMSO[c]	
			ΔE	ΔG_r	ΔE	ΔG_r	ΔE	ΔG_r	ΔE	ΔG_r
**29**	a	$^{4}C_{1}$	3.01	2.00	2.81	2.76	2.20	1.69	2.14	1.63
	b	$^{4}C_{1}$	3.14	2.06	1.76	1.07	3.16	2.40	1.82	0.74
	c	$^{4}C_{1}$	2.93	2.03	0.88	1.07	3.33	2.59	1.03	0.59
**69**	a	$^{4}C_{1}$	1.88	1.85	2.11	2.28	0.89	0.14	1.32	0.35
	b	$^{4}C_{1}$	1.93	1.83	0.87	0.99	1.77	1.47	0.74	0.32
	c	$^{4}C_{1}$	0.00	0.00	0.00	0.00	0.00	0.00	0.00	0.00
**56**	a	$^{2}T_{1}$	9.46	6.51	7.38	5.06	11.45	8.41	9.42	7.18
	b	$^{2}T_{1}$	6.11	3.77	5.66	4.42	7.45	5.30	7.36	6.00
	c	$^{2}T_{1}$	5.63	3.21	4.97	2.66	7.90	5.74	7.17	5.00
	d	$^{1}T_{2}$	0.08	-0.68	-0.45	0.39	1.37	0.32	1.06	0.58
	e	$^{1}T_{2}$	7.69	5.89	6.73	5.03	8.54	6.59	7.81	7.17
	f	^{4}E	6.71	4.70	5.51	4.04	9.24	7.30	8.12	6.12
**82**	a	$^{2}T_{1}$	4.41	2.87	3.10	2.57	6.07	3.81	4.85	2.96
	a*	$^{2}T_{1}$	7.99	4.68	6.60	4.09	9.71	6.37	8.51	6.10
	b	$^{2}T_{1}$	5.50	3.50	5.28	4.40	7.32	5.37	7.37	6.18
	c	$^{2}T_{1}$	0.50	-0.61	-0.07	-0.48	2.69	1.10	2.34	1.02
	d	$^{1}T_{2}$	4.42	2.61	2.60	2.27	5.66	4.11	4.14	3.32
	e	E_{2}	7.16	5.27	5.24	2.95	8.93	6.56	7.19	5.45

[a]Ring conformation. [b]B3LYP/6-31G(d,p). [c]M06-2X/6-311G(d,p).

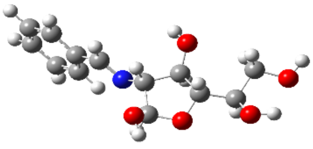
56a

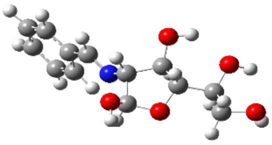
56b

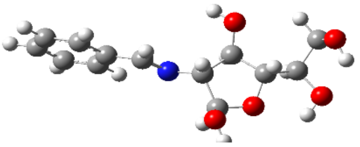
56c

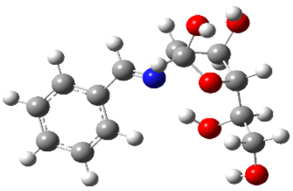
56d

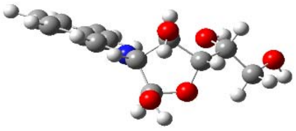
56e

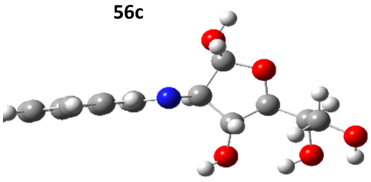
56f

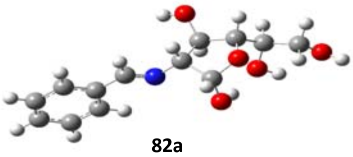
82a

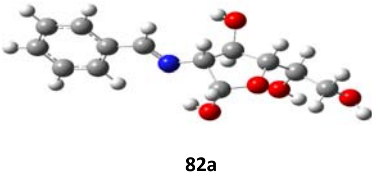
82a

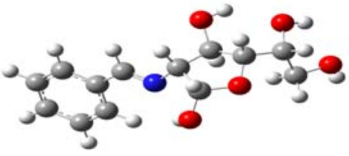
82b

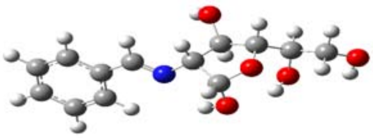
82c

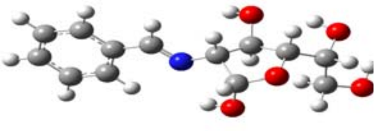
82d

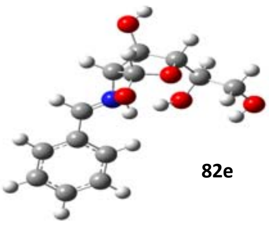
82e

Entries **a**, **b**, and **c** in cyclic structures **29**, **56**, **69**, and **82** refer to staggered conformations of the hydroxymethylene OH group at C-6. In pyranoid species **a** and **b** that OH group is involved in hydrogen bonding with the OH group at C-4. On the contrary, that hydrogen bonding takes place with the endocyclic oxygen in species **c**.

The β-anomer (**56**) adopting the $^{1}T_{2}$ conformation is capable of forming two hydrogen bonds: one involving the OH group at C-5 and the iminic nitrogen and the other between the anomeric OH and the OH group at C-3, thus being the most stable furanoid structure (entry **d**). Removal of the H-bond with the iminic nitrogen and forming it with either the OH at C-6 (entry **e**) or the OH at C-3 (entry **f**) appreciably decreases stability.

Entry **a** point to the greater stability of the α-anomer (**82**), presumably due to a stabilizing H-bondinf between the anomeric OH and the OH at C-5, leading to a furanoid ring in $^{2}T_{1}$ conformation. However, H-bonding to the iminic nitrogen (entries **a*** and **b**) strongly reduces stability (~3 kcal/mol). H-Bonding of the OH at C-5 to the anomeric OH oxygen of the OH at C-3 (entry **d**), and the same happens when H-boinding involves the OH at C-5 and iminic nitrogen (entry **e**)

Table S14. Relative Stabilities (kcal/mol) of 97-100 Involved in the Mutarotation of 29[a]

	Entry	Gas phase[a]		DMSO[a]		Gas phase[b]		DMSO[b]	
		ΔE	ΔG_r	ΔE	ΔG_r	ΔE	ΔG_r	ΔE	ΔG_r
**97**	a	14.08	9.95	13.64	10.24	16.17	12.38	15.74	12.38
	c	13.65	10.19	12.70	10.08	17.09	13.03	16.01	12.72
**98**	a	7.46	4.44	7.92	4.81	9.40	6.52	10.01	6.92
	c	12.55	7.65	11.19	7.43	14.78	10.95	12.77	9.16
**99**	a	11.69	6.66	12.58	7.67	14.26	9.50	15.32	10.89
	c	13.59	9.53	12.27	8.85	17.40	13.17	16.07	12.48
**100**	a	11.71	6.95	9.99	5.00	14.64	9.93	13.07	7.85
	c	15.07	9.96	10.94	6.69	17.36	11.46	14.30	9.75

[a] B3LYP/6-31G(d,p). [b] M06-2X/6-311G(d,p).

Table S15. Geometrical (Å and °) and Energy (kcal/mol) Data of Hydrogen Bonding for 56d

		D–H···*A*	*d*(*D*–H)	*d*(H···*A*)	*d*(*D*···*A*)	∠(*DHA*)	E_{HB}[e]
B3LYP	Gas phase[a]	O-H···N	0.98	1.91	2.81	151.24	-5.21
	DMSO[a]	O-H···N	0.98	1.87	2.80	156.75	-5.43
	Gas phase[a]	O-H···O[d]	0.97	2.09	2.86	135.36	-4.24
	DMSO[a]	O-H···O[d]	0.97	2.05	2.83	136.30	-4.80
M06-2X	Gas phase[b]	O-H···N	0.97	1.99	2.84	144.49	-4.60
	DMSO[b]	O-H···N	0.97	1.91	2.83	154.60	-4.80
	Gas phase[b]	O-H···O[d]	0.96	2.12	2.86	132.70	-4.24
	DMSO[b]	O-H···O[d]	0.97	2.08	2.83	133.38	-4.80
	Gas phase[c]	O-H···N	0.97	1.97	2.87	152.54	-4.07
	DMSO[c]	O-H···N	0.98	1.90	2.84	160.57	-4.60
	Gas phase[c]	O-H···O[d]	0.97	2.17	2.90	131.15	-3.60
	DMSO[c]	O-H···O[d]	0.97	2.12	2.86	132.10	-4.24

[a]At B3LYP/6-31G(d,p) level. [b] At M06-2X/6-311G(d,p) level. [c] At M06-2X-def2-TZVP level [d] Hydrogen bond between the OH groups at C-1 and C-3. [e] Calculated from equation [20], Reference 72.

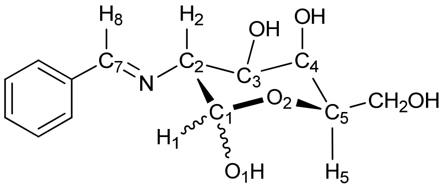

Table S16. NBO Data for 29c and 69c

Donor	Acceptor	29c		69c	
		Gas phase	DMSO	Gas phase	DMSO
LP(1) N	BD*(1) C7 – H8	13.38	12.67	12.69	12.05
LP(1) N	BD*(1) C2 - H2	6.96	6.92	5.59	5.64
LP (1) O2	BD*(1) C1 - O1	4.38	4.29	1.06	1.04
LP (1) O2	BD*(1) C1 - H1			3.49	3.51
LP (2) O2	BD*(1) C2 - C1	6.14	6.54	5.73	5.64
LP (2) O2	BD*(1) C4 – C5	6.83	7.00	5.78	5.78
LP (2) O2	BD*(1) C5 – H5	5.68	5.23	6.06	6.3
LP (2) O2	BD*(1) C1 - H1	6.49	5.95		
LP (2) O2	BD*(1) C1 - O1			11.71	12.32
LP (1) O1	BD*(1) C1 - H1	2.43	2.29		
LP (1) O1	BD*(1) C2 - C1			4.35	4.36
LP (2) O1	BD*(1) O2 - C1	17.95	17.83	4.55	5.25
LP (2) O1	BD*(1) C1 - H1	5.89	5.46	10.64	9.96

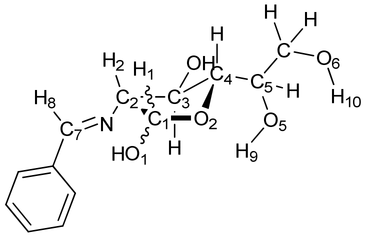

Table S17. NBO Data of 56d and 82c

Donor	Acceptor	56d		82c	
		Gas phase	DMSO	Gas phase	DMSO
LP (1) N	BD*(1) C2 – H2	6.9	6.31	6.42	6.34
LP (1) N	BD*(1) C7 – H8	12.96	11.86	12.83	11.95
LP (1) N	BD*(1) O5 – H9	9.54	13.89		
LP (1) O2	BD*(1) C4 – C3	3.19	3.23	5.58	3.49
LP (2) O2	BD*(1) C4 – H4	8.14	8.06	5.86	5.49
LP (2) O2	BD*(1) C4- C5	2.74	2.82	5.58	5.62
LP (2) O2	BD*(1) C1 – C2	3.41	3.39	1.98	1.94
LP (2) O2	BD*(1) C1 – O1	18.08	17.52	17.18	17.21
LP (1) O1	BD*(1) C1 – C2	1.04	1.06	4.64	4.53
LP (2) O1	BD*(1) O2 – C1	11.72	12.83	7.92	8.22
LP (2) O1	BD*(1) C1 – H1	7.36	6.67	7.21	6.14
LP (2) O1	BD*(1) O5 – H9			4.44	6.55

Table S18. Crystallographic and DFT-Calculated Bond Lengths (Å) and Dihedral Angles (degrees) for compound 38[a]

Bond length	Crystal	Gas phase	$CHCl_3$
C1-Oendo	1.41	1.40	1.40
C6-Oendo	1.44	1.41	1.42
C1-Oexo	1.42	1.40	1.41
C2-N	1.45	1.44	1.45
N-CH	1.27	1.27	1.27
Dihedral angle			
H1 C1 C2 H2	165.94	175.71	176.21
H2 C2 C3 H3	-162.67	-174.76	-174.36
H3 C3 C4 H4	49.11	54.01	53.07
H4 C4 C5 H5	-62.55	-57.34	-57.52
O C1 C2 C3	50.71	56.83	57.39
C1 C2 C3 C4	-45.24	-52.24	-52.28
C2 C3 C4 C5	50.08	52.44	52.17
C3 C4 C5 O	-59.76	-55.92	-55.83
C4 C5 O C1	67.60	63.91	63.96
C5 O C1 C2	-62.98	-64.75	-65.11
H2 C2 N CH	16.36	7.64	7.64
C2 N CH H	0.44	-0.74	-0.74
H2 C2 (=CH) H	15.90	6.40	6.42

[a]At the M06-2X/6-311G(d,p) level.

Table S19. NBO Data of 38[a]

Donor	Acceptor	Gas phase	$CHCl_3$
LP (1) N19	BD*(1) C2 - H10	7.38	7.08
LP (1) N19	BD*(1) C30 - H31	13.35	12.67
LP (1) O11	BD*(1) C1 - O18	5.53	5.36
LP (2) O11	BD*(1) C1 - C2	7.91	7.91
LP (2) O11	BD*(1) C1 - H6	6.77	6.44
LP (2) O11	BD*(1) C4 - C5	8.06	7.99
LP (2) O11	BD*(1) C5 - H9	6.16	5.77
LP (1) O18	BD*(1) C34 - O36	7.1	7.13
LP (2) O18	BD*(1) C1 - H6	4.3	4.03
LP (2) O18	BD*(1) C1 - O11	12.51	12.66
LP (2) O18	BD*(1) C34 - O36	50.81	51.68
LP (1) O17	BD*(1) C35 - O39	6.98	7.26
LP (2) O17	BD*(1) C3 - C4	7.29	7.08
LP (2) O17	BD*(1) C3 - H7	3.51	3.4
LP (2) O17	BD*(1) C35 - O39	49.55	51.99
LP (1) O16	BD*(1) C4 - H8	2.68	2.73
LP (1) O16	BD*(1) C33 - O38	7.73	7.71
LP (2) O16	BD*(1) C3 - C4	6.59	6.53
LP (2) O16	BD*(1) C33 - O38	54.65	55.03
LP (1) O15	BD*(1) C12 - H14	3.22	3.11
LP (1) O15	BD*(1) C32 - O37	7.38	7.7
LP (2) O15	BD*(1) C5 - C12	5.95	5.68
LP (2) O15	BD*(1) C32 - O37	48.98	52.18

[a]In kcal/mol.

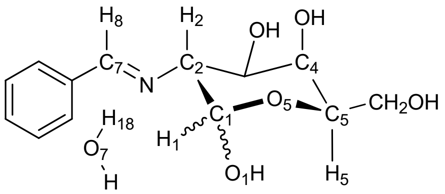

Table S20. H-Bonding Data for Monohydrated species of 29 and 69[a]

	Anomer	Medium	Angle[b]	*D*-H···*A*	*d*(*D*-H)	*d*(H···*A*)	*d*(*D*···*A*)	Ð(*DHA*)	E_{HB}[c]
**29·1H2O**	**β**	Gas phase	30.17	O7-H18···N	0.968	1.993	2.913	157.86	-3.41
		DMSO	26.13	O7-H18···N	0.971	1.982	2.901	157.03	-3.59
		Water	25.76	O7-H18···N	0.975	1.945	2.889	162.37	-3.75
**69·1H2O**	**α**	Gas phase	33.07	O7-H18···N	0.980	1.856	2.793	158.68	-5.60
		DMSO	8.43	O7-H18···N	0.978	1.880	2.790	153.59	-5.65
		Water	27.71	O7-H18···N	0.981	1.872	2.828	163.76	-4.84

[a]At the M06-2X/6-311G(d,p). [b]Dihedral angle H8-C7-C2-H2. [c]In kcal/mol.

Table S21. Calculated Geometric Parameters for Intramolecular Hydrogen Bonds

		***D*-H···A**	***d*(*D*-H)[c]**	***d*(H···*A*)[c]**	***d*(*D*···*A*)[c]**	**<(*DHA*)[d]**	***E*HB[e]**
**69.5H₂O**	Gas phase[a]	O_1-H···N	0.9662	2.1792	2.7371	115.44	-7.04
	DMSO[a]	O_1-H···N	0.9682	2.1554	2.7176	115.58	-7.62
	Water[a]	O_1-H···N	0.9677	2.2283	2.7719	114.40	-6.10
	Gas phase[b]	O_1-H···N	0.9683	2.1614	2.7300	116.14	-7.24
	DMSO[b]	O_1-H···N	0.9696	2.1424	2.7094	115.84	-7.89
	Water[b]	O_1-H···N	0.9689	2.2077	2.7553	114.57	-6.53

[a]M06-2X/6-311G(d,p). [b]M06-2X/def2- TZVP. [c]In Å. [d]In degrees. [e]From equation [20].

Table S22. Calculated Geometric Parameters of Intramolecular Hydrogen Bonds in β-Furanose Imine

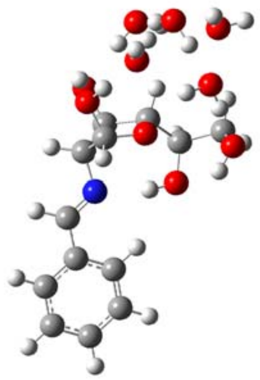

		***D*-H···A**	***d*(*D*-H)[c]**	***d*(H···*A*)[c]**	***d*(*D*···*A*)[c]**	**<(*DHA*)[d]**	***E*HB[e]**
**56·5H2O b**	Gas phase[a]	O-H···N	0.9759	1.8586	2.8125	164.97	-5.16
	DMSO[a]	O-H···N	0.9793	1.8249	2.7890	167.45	-5.68
	Water[a]	O-H···N	0.9810	1.8154	2.7859	169.59	-5.75
	Gas phase[b]	O-H···N	0.9772	1.8811	2.8442	167.95	-4.53
	DMSO[b]	O-H···N	0.9806	1.8539	2.8202	167.88	-5.00
	Water[b]	O-H···N	0.9836	1.8294	2.8015	169.10	-5.40

[a] M06-2X/6-311G(d,p). [b]M06-2X/def2- TZVP. [c]In Å. [d]In degrees. [d]From equation [20].

Table S23. Calculated Geometric Parameters of Intramolecular Hydrogen Bonds in α-Furanose Imine

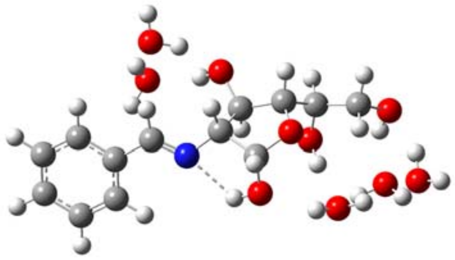

		D-H···A	*d*(*D*-H)[c]	*d*(H···*A*)[c]	*d*(*D*···*A*)[c]	<(*DHA*)[d]	E_{HB}[e]
**82·5H2O**	Gas phase[a]	O-H···N	0.9675	2.0984	2.7107	119.65	-7.84
	DMSO[a]	O-H···N	0.9695	2.0759	2.6916	119.70	-8.49
	Water[a]	O-H···N	0.9677	2.9588	2.8698	75.28	-4.07
	Gas phase[b]	O-H···N	0.9686	2.1117	2.7163	118.96	-7.67
	DMSO[b]	O-H···N	0.9707	2.0857	2.6953	119.14	-8.36
	Water[b]	O-H···N	0.9676	2.2990	2.7702	109.06	-6.14

[a] M06-2X/6-311G(d,p); [b]M06-2X/def2- TZVP; [c] in Å; [d] in °, [e] from equation [20]

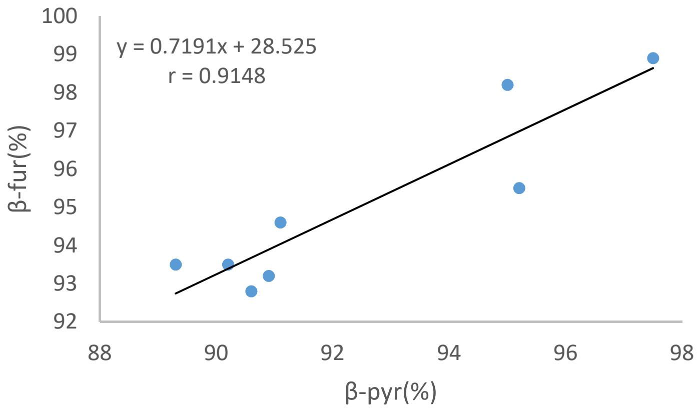

Figure S1. Plot of furanoside versus pyranoside percentages for β-anomers derived from compounds **21**, **28**, **30-34**, and **36**.

IR spectra

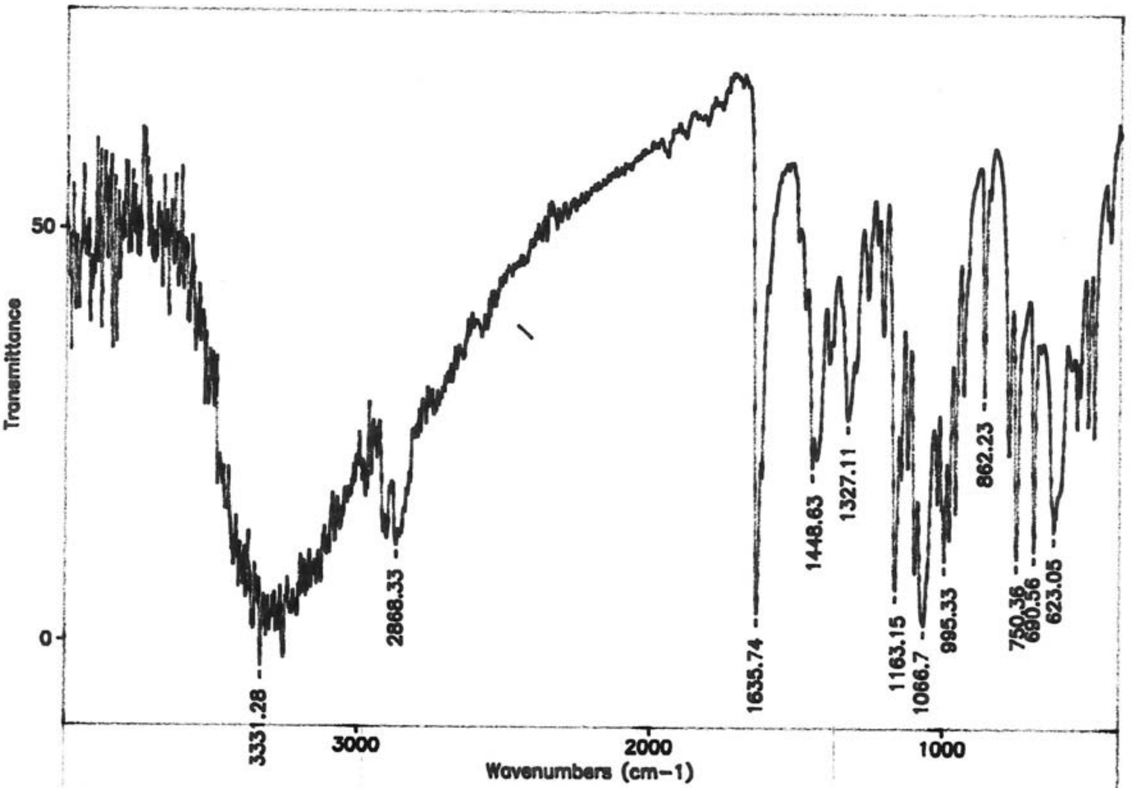

Figure S2. IR spectrum of **21**.

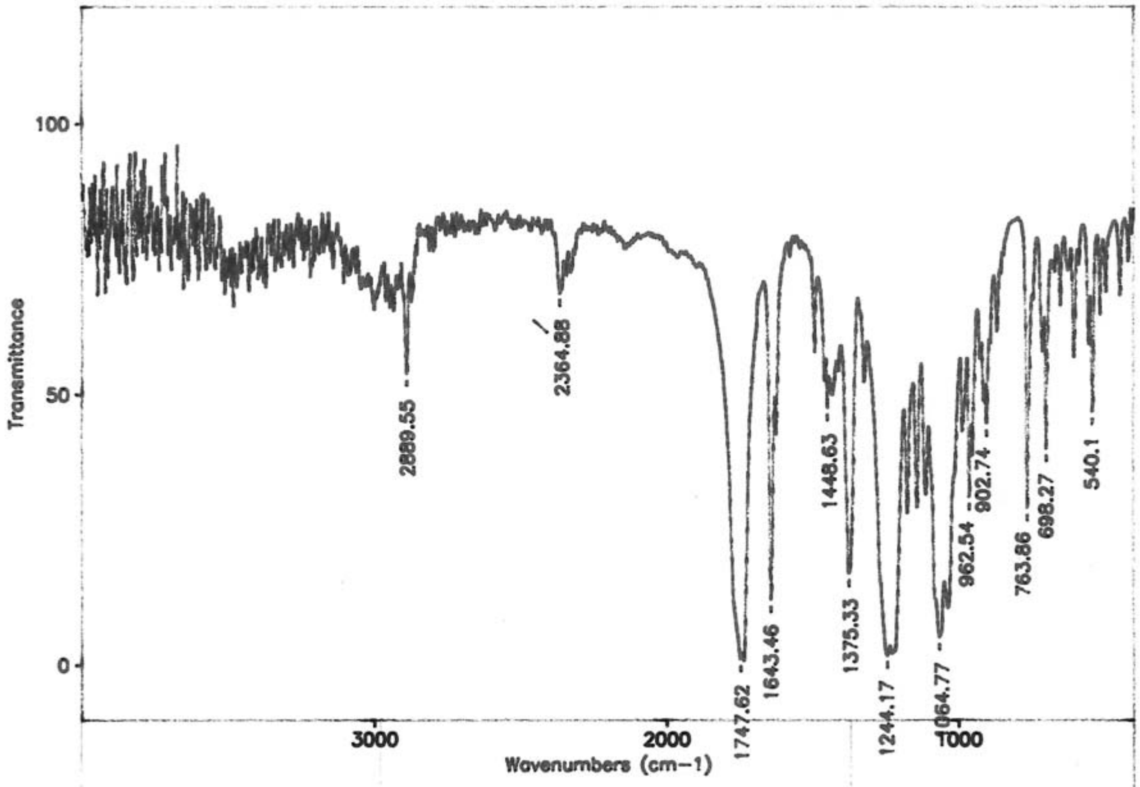

Figure S3. IR spectrum of **22**.

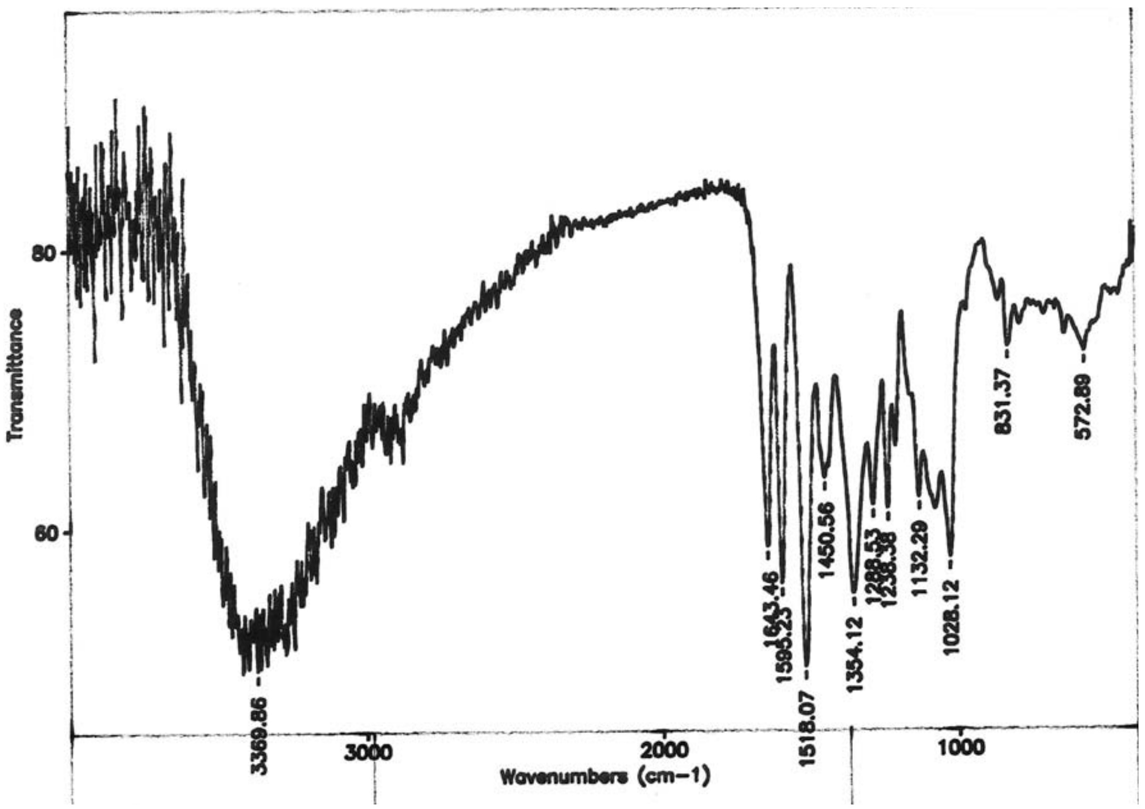

Figure S4. IR spectrum of **27**.

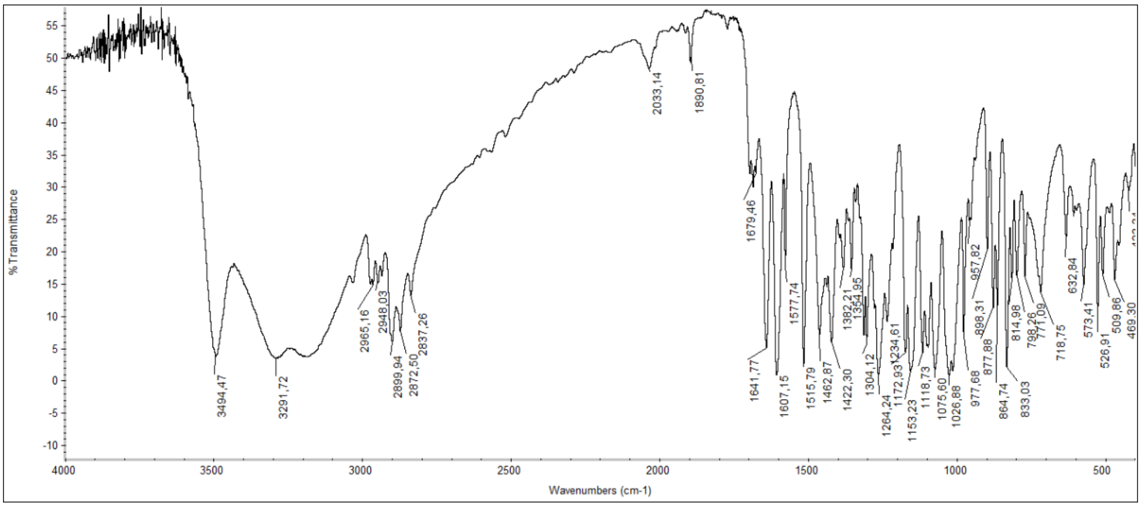

Figure S5. IR spectrum of **28**.

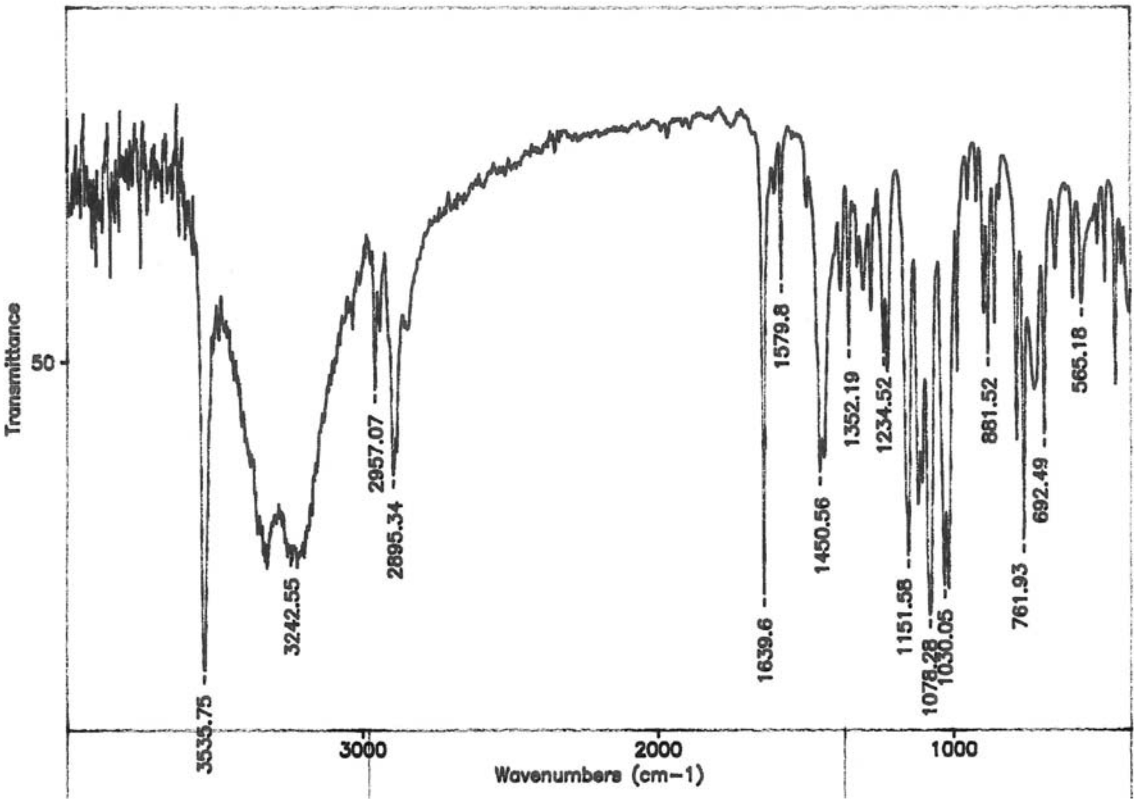

Figure S6. IR spectrum of **29**.

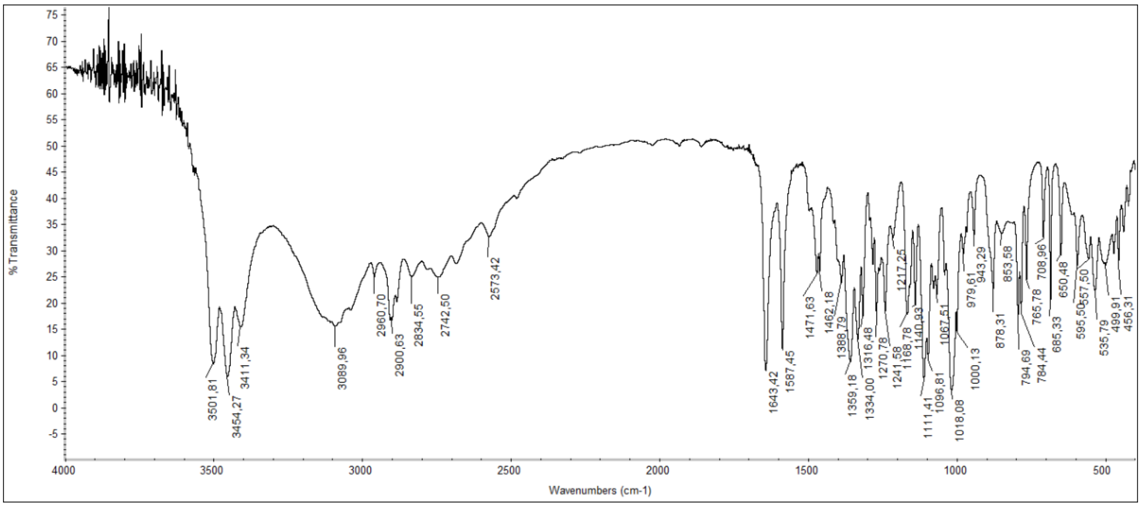

Figure S7. IR spectrum of **30**.

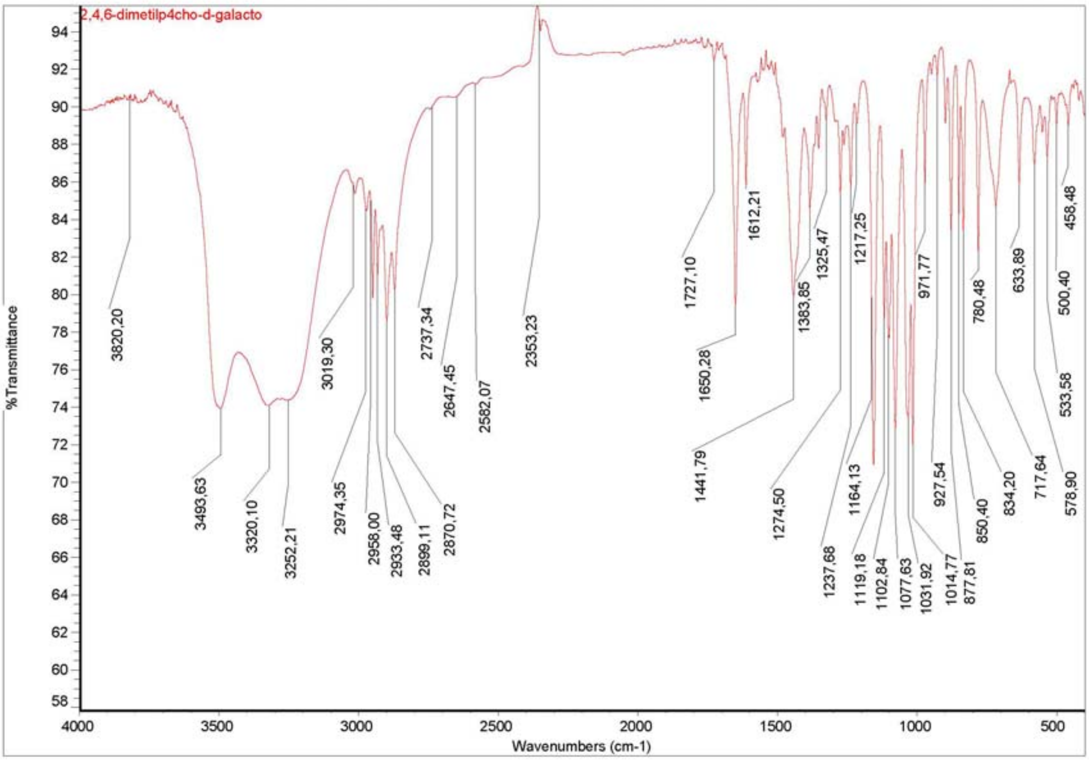

Figure S8. IR spectrum of **31**.

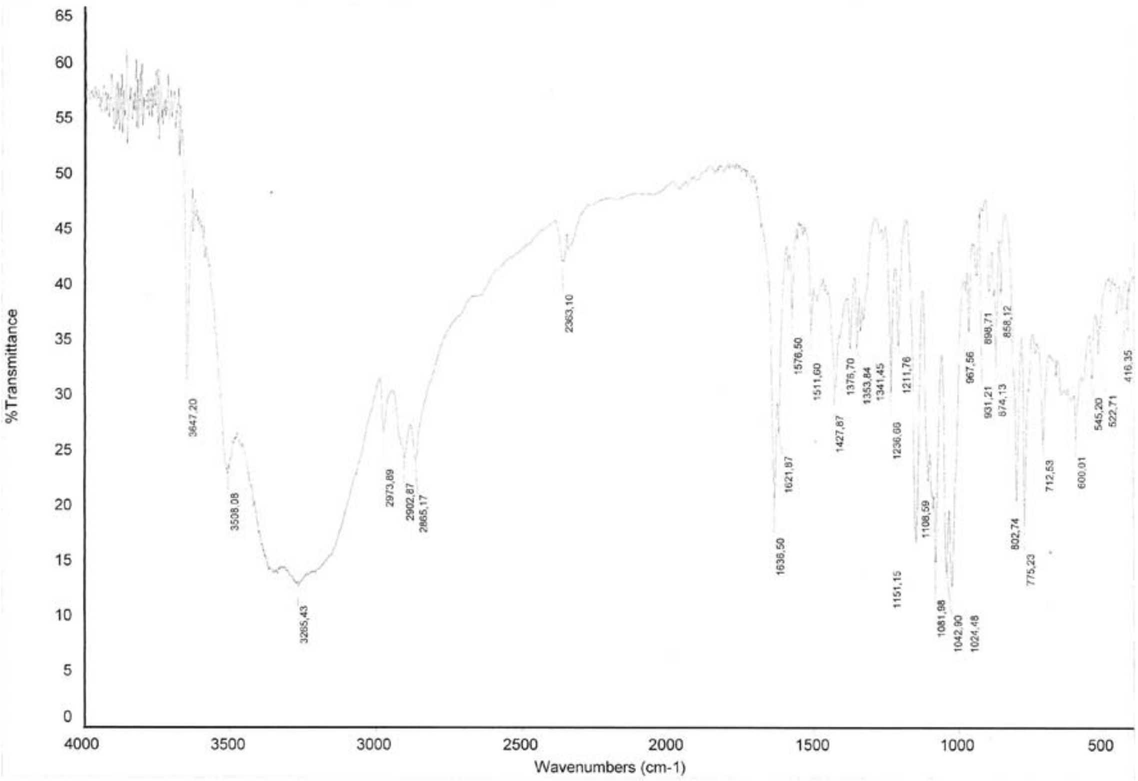

Figure S9. IR spectrum of **32**.

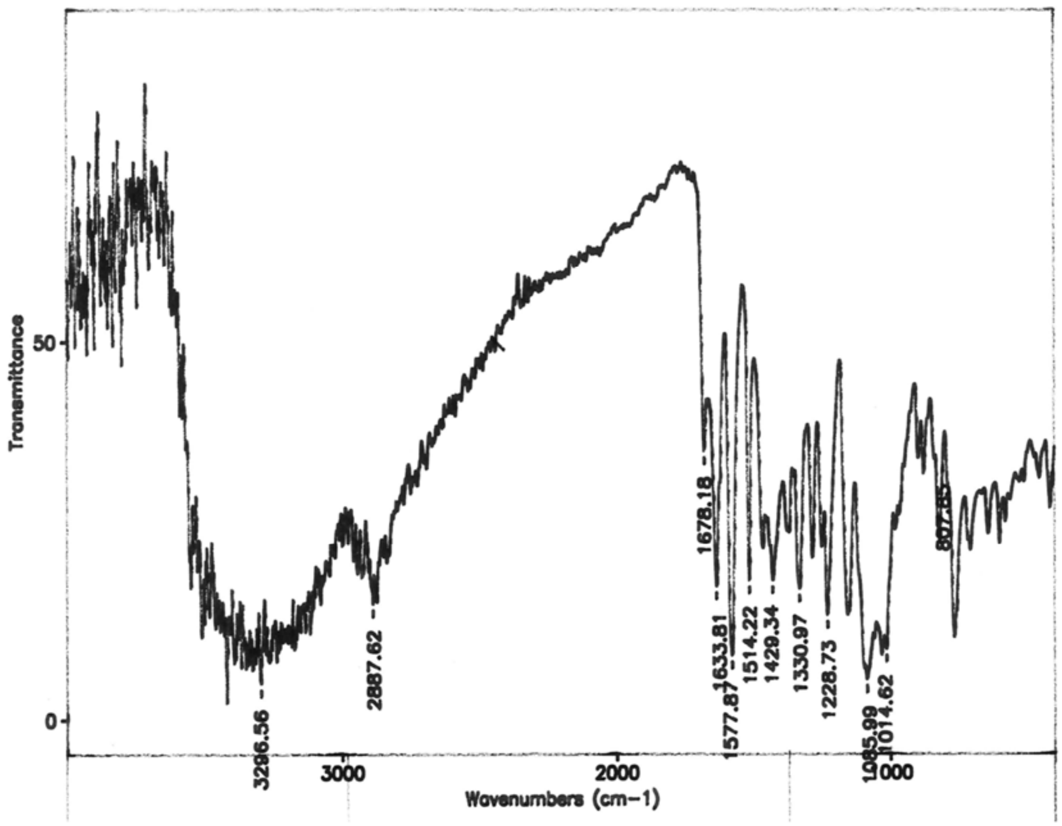

Figure S10. IR spectrum of **33**.

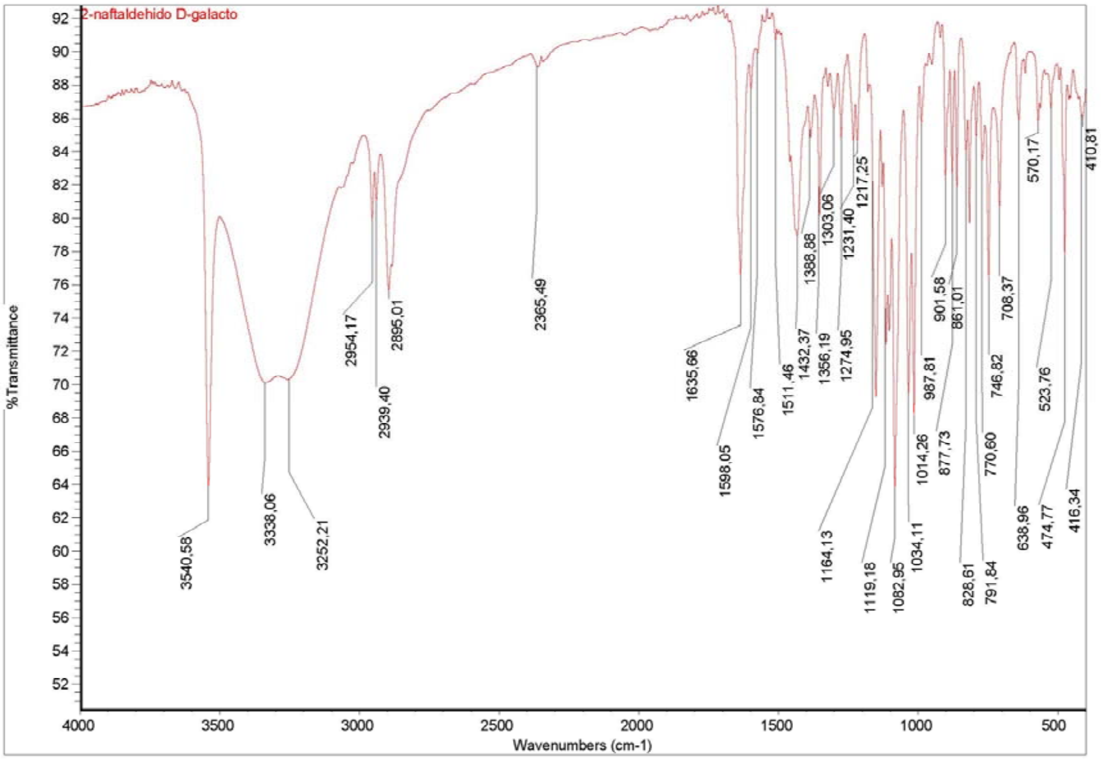

Figure S11. IR spectrum of **34**.

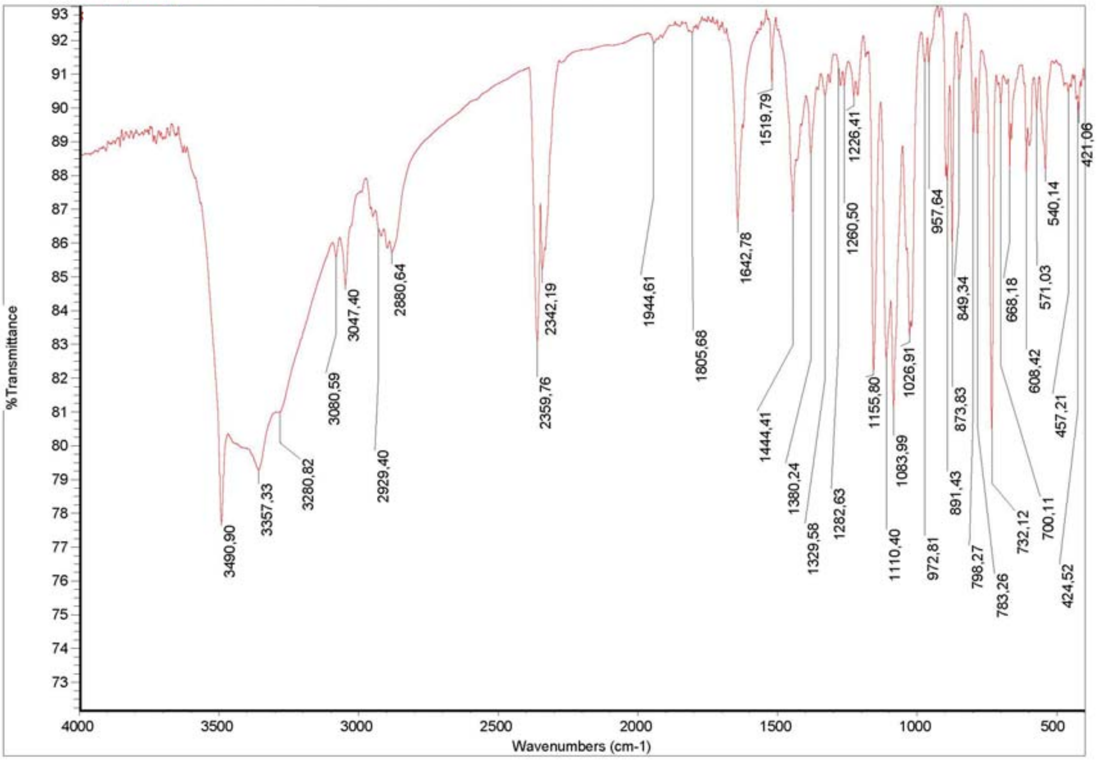

Figure S12. IR spectrum of **35**.

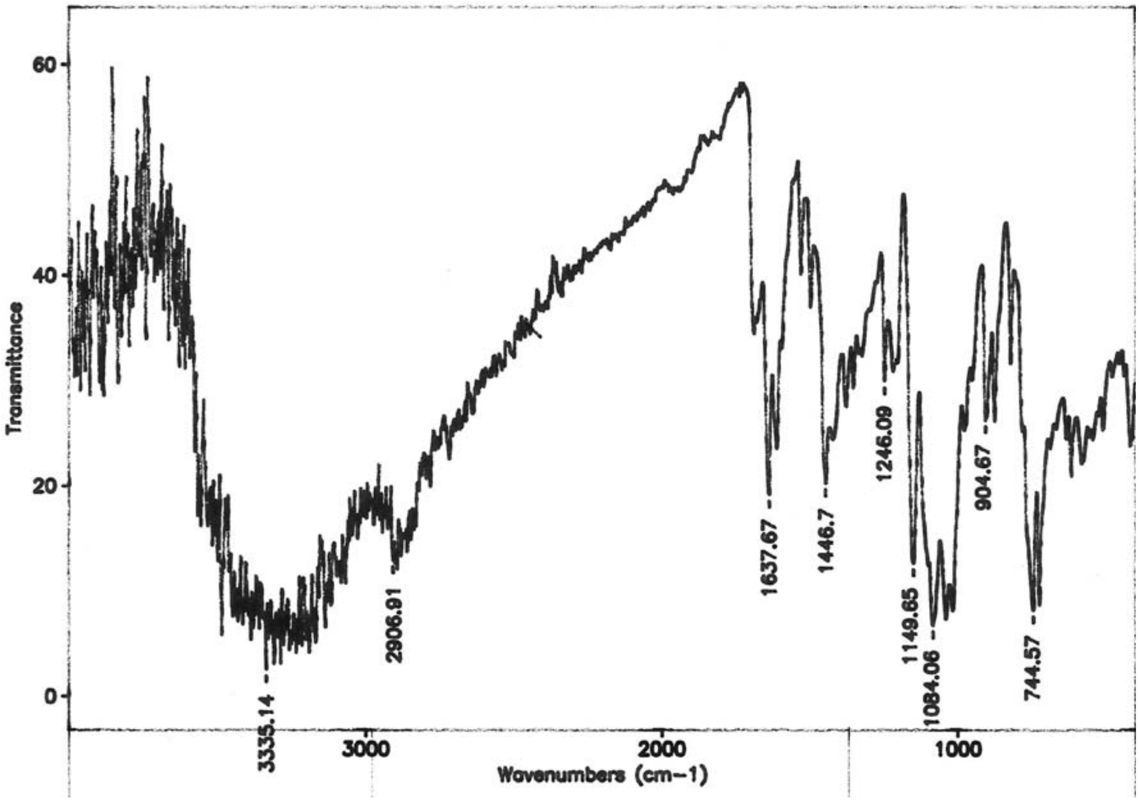

Figure S13. IR spectrum of **36**.

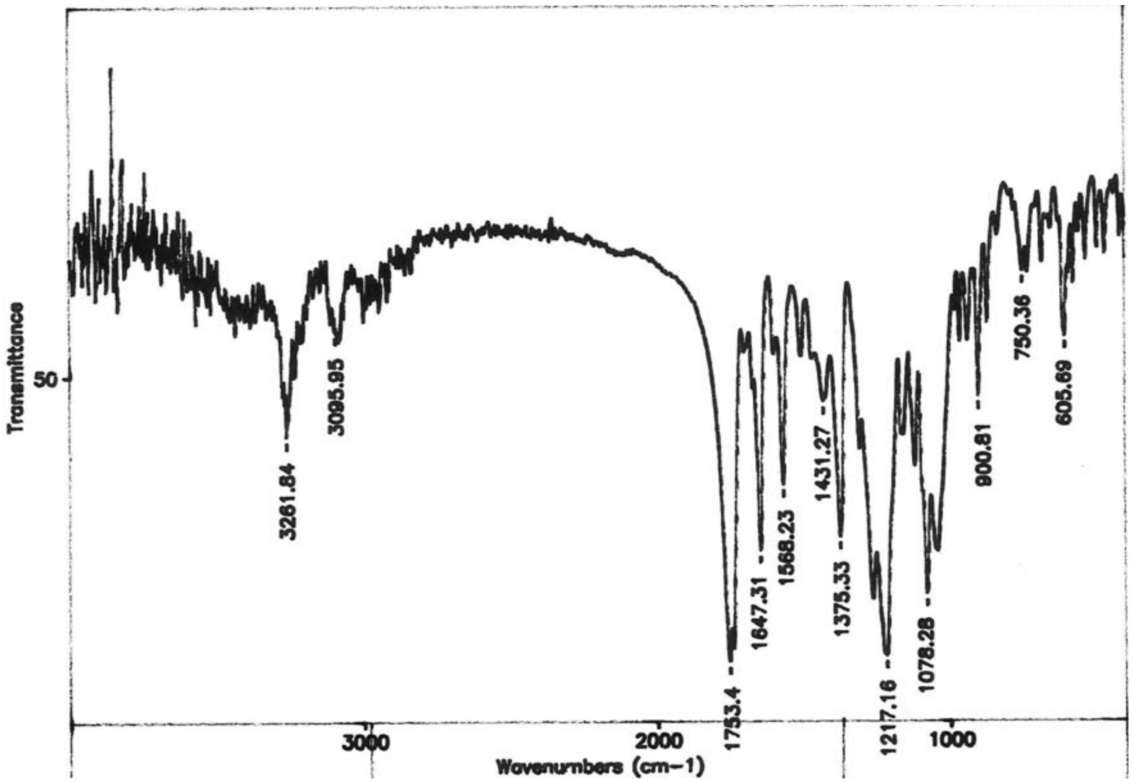

Figure S14. IR spectrum of **37** and **41**.

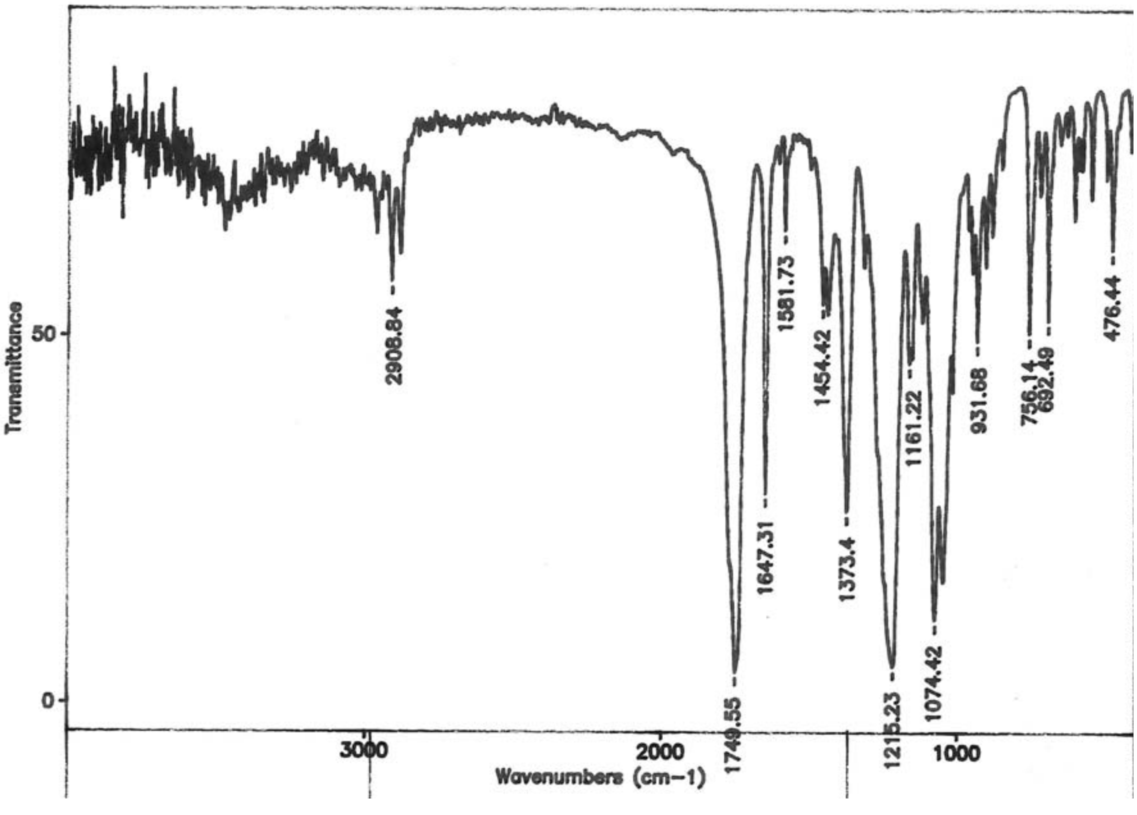

Figure S15. IR spectrum of **38**.

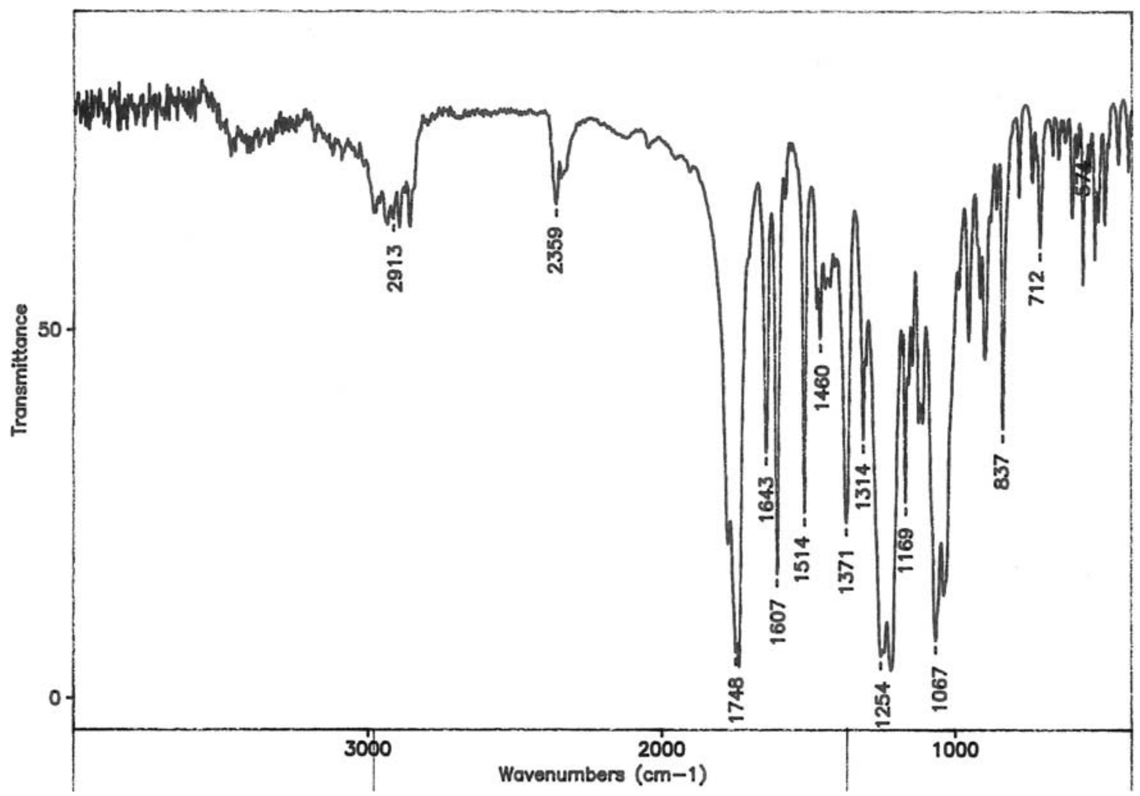

Figure S16. IR spectrum of **39**.

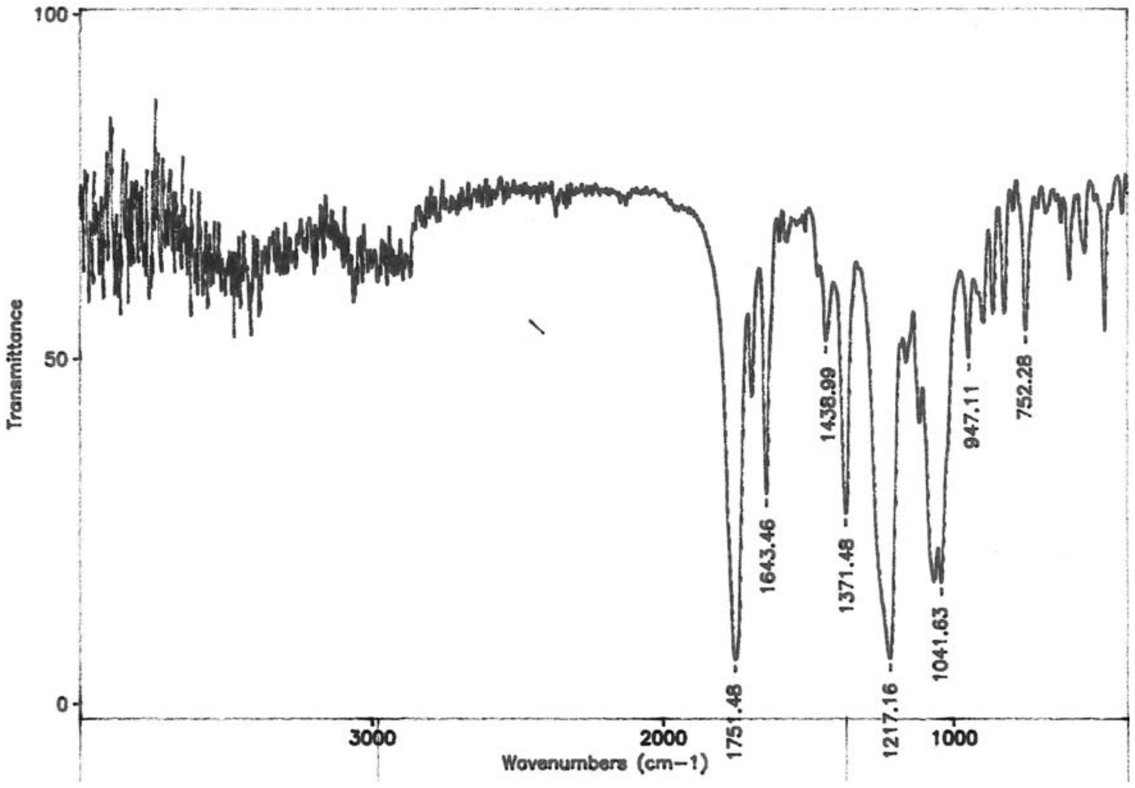

Figure S17. IR spectrum of **43**.

NMR spectra

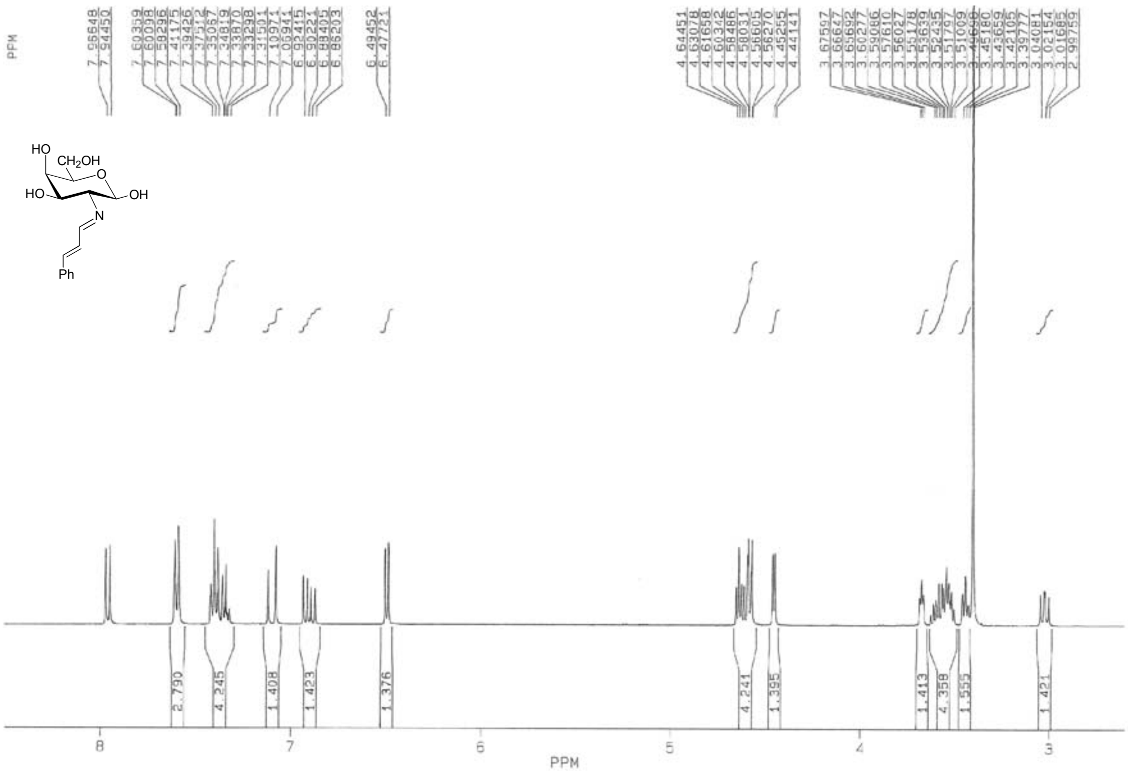

Figure S18. ^{1}H NMR spectrum of **21** (400 MHz, DMSO-d_6).

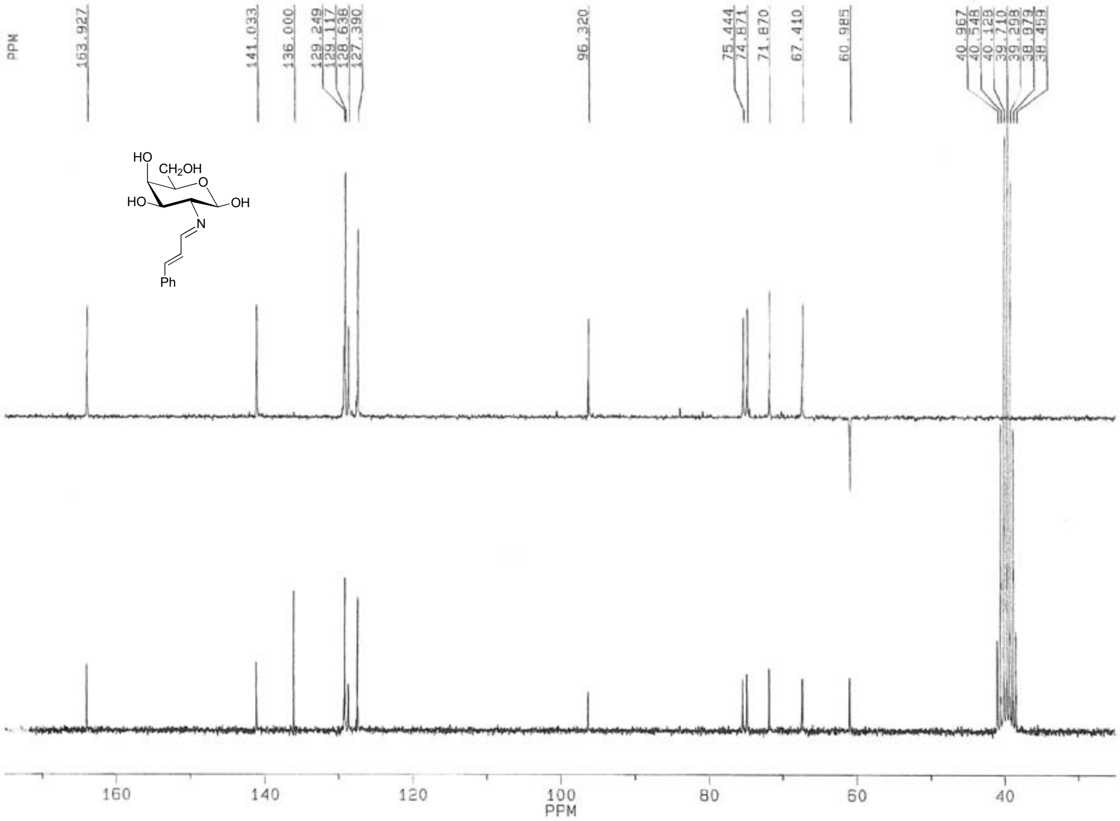

Figure S19. $^{13}C\{^{1}H\}$ NMR spectrum of **21** (100 MHz, DMSO-d_6).

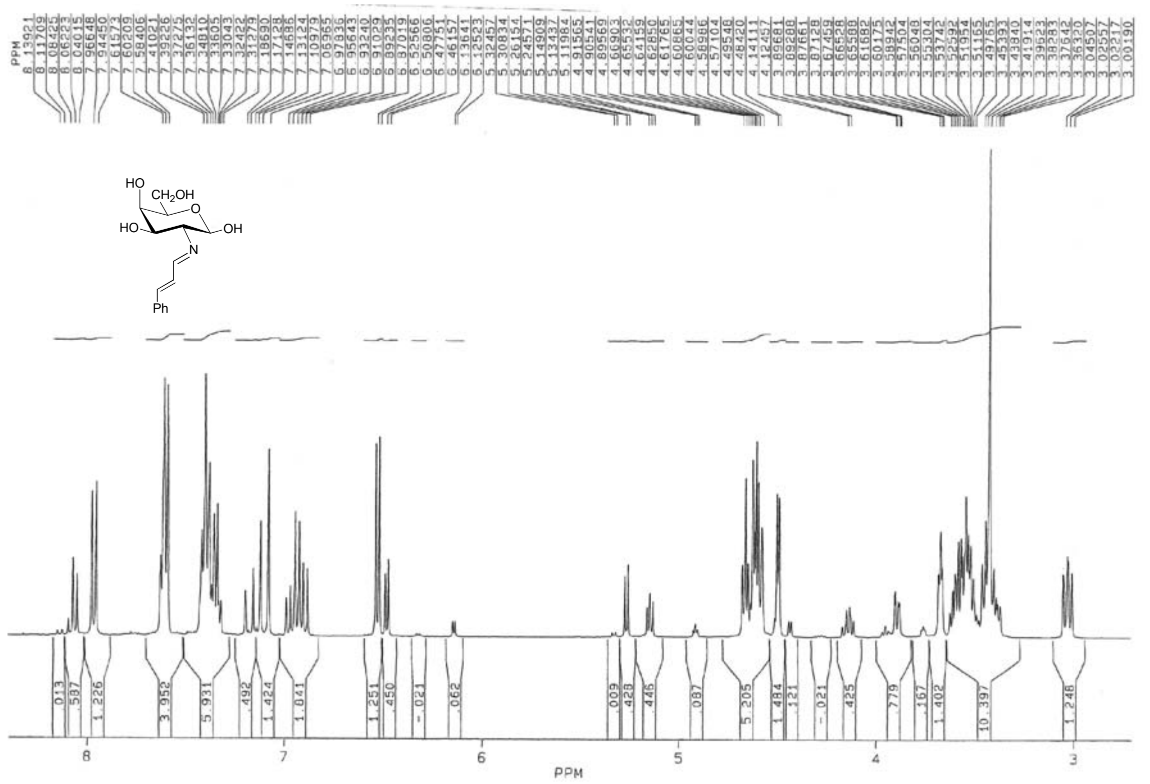

Figure S20. ^{1}H NMR spectrum of **21** in equilibrium (400 MHz, DMSO-d_6).

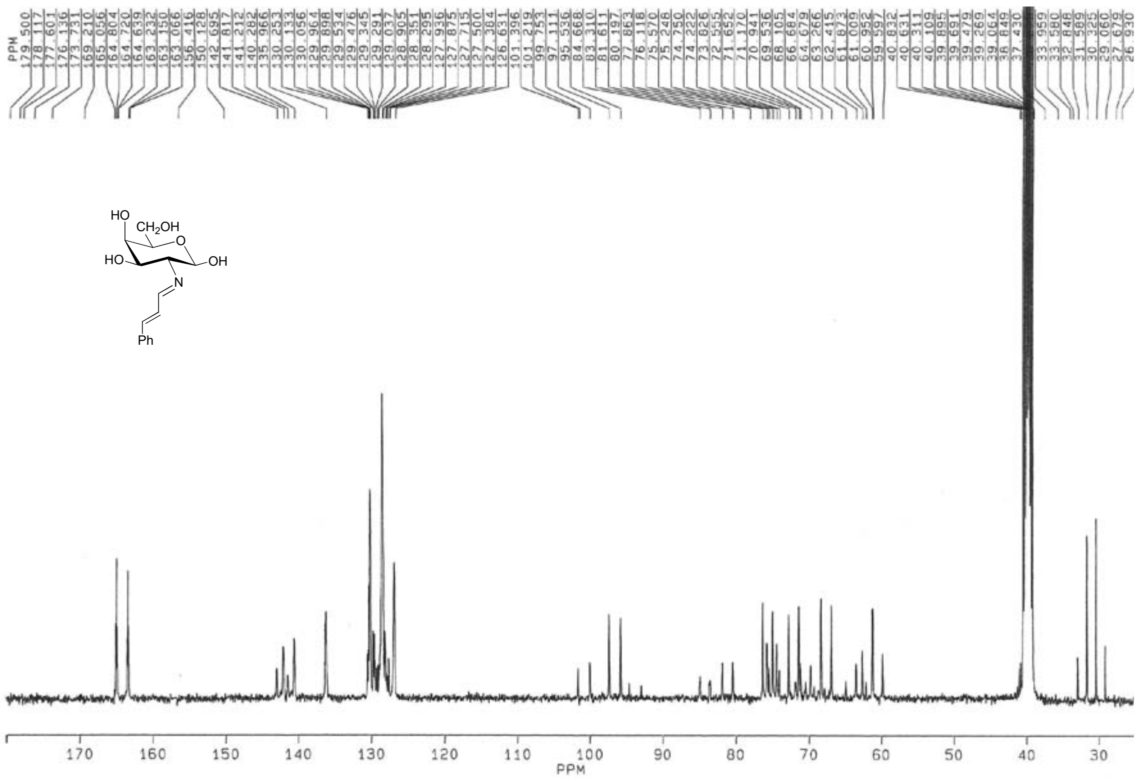

Figure S21. Coupled ^{13}C NMR spectrum of **21** in equilibrium (100 MHz, DMSO-d_6).

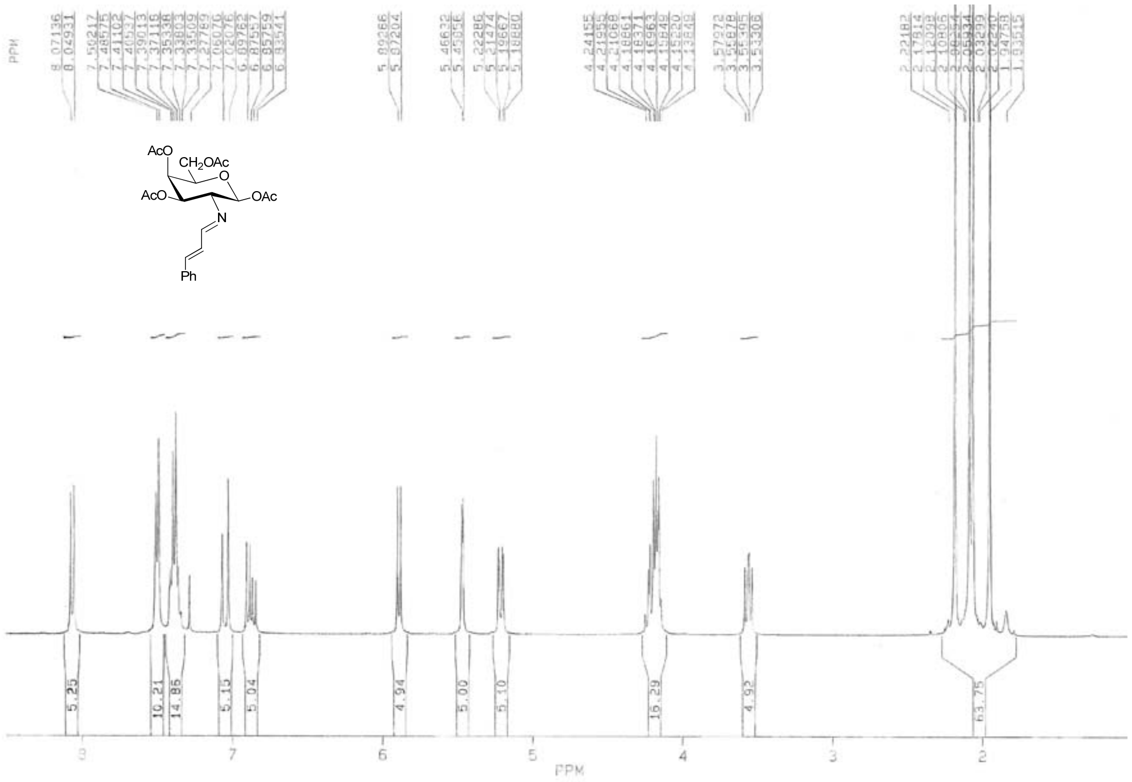

Figure S22. ^{1}H NMR spectrum of **22** (400 MHz, DMSO-d_6).

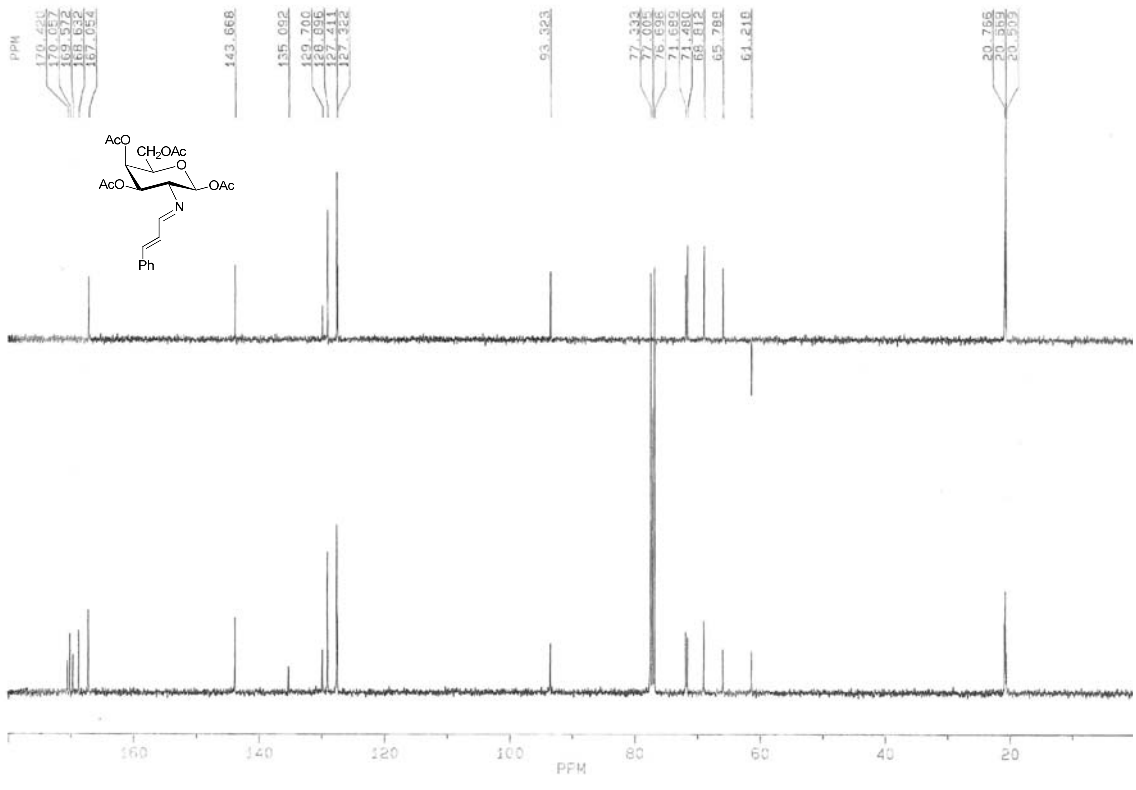

Figure S23. $^{13}C\{^{1}H\}$ NMR spectrum of **22** (100 MHz, DMSO-d_6).

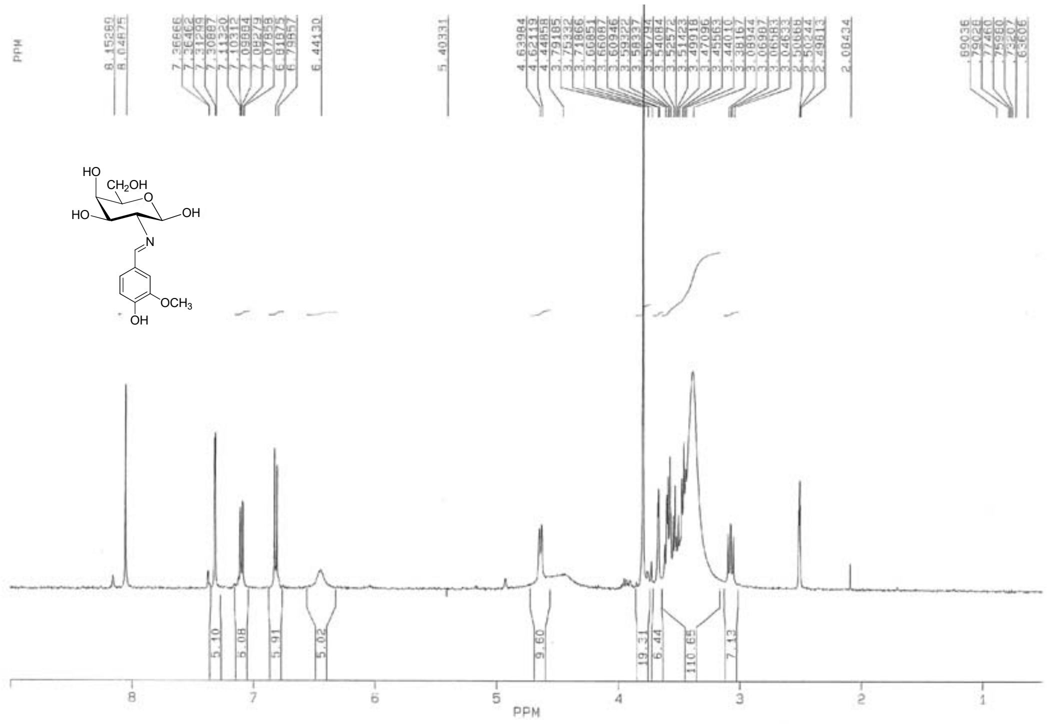

Figure S24. ^{1}H NMR spectrum of **27** (400 MHz, DMSO-d_6).

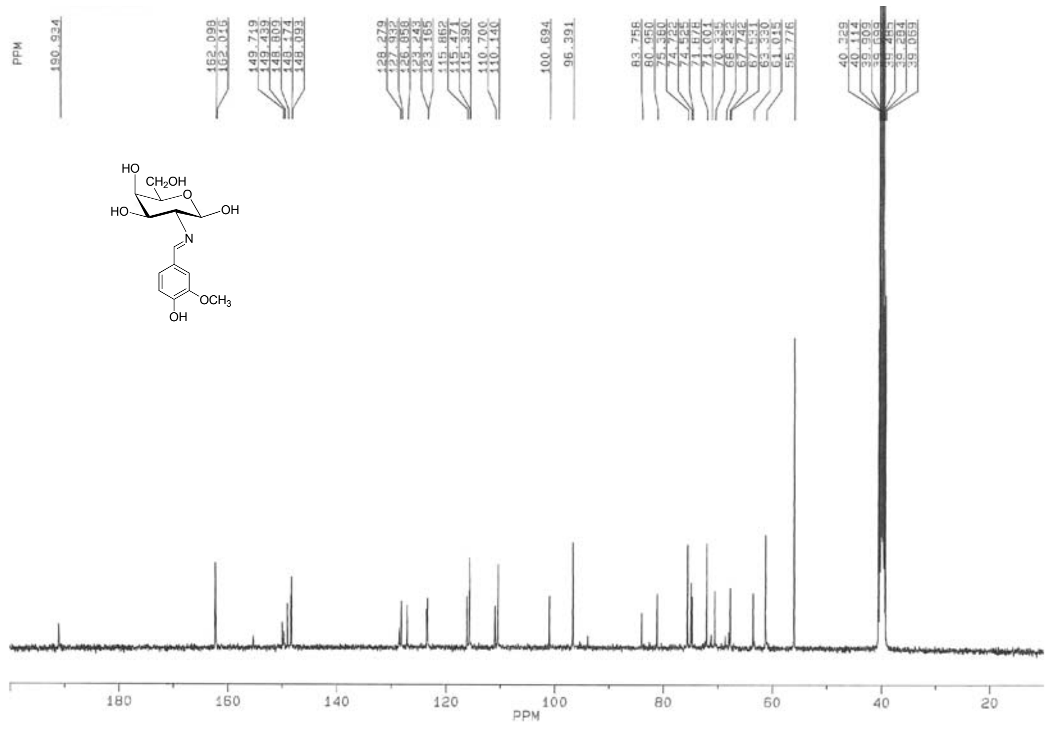

Figure S25. $^{13}C\{^{1}H\}$ NMR spectrum of **27**(100 MHz, DMSO-d_6).

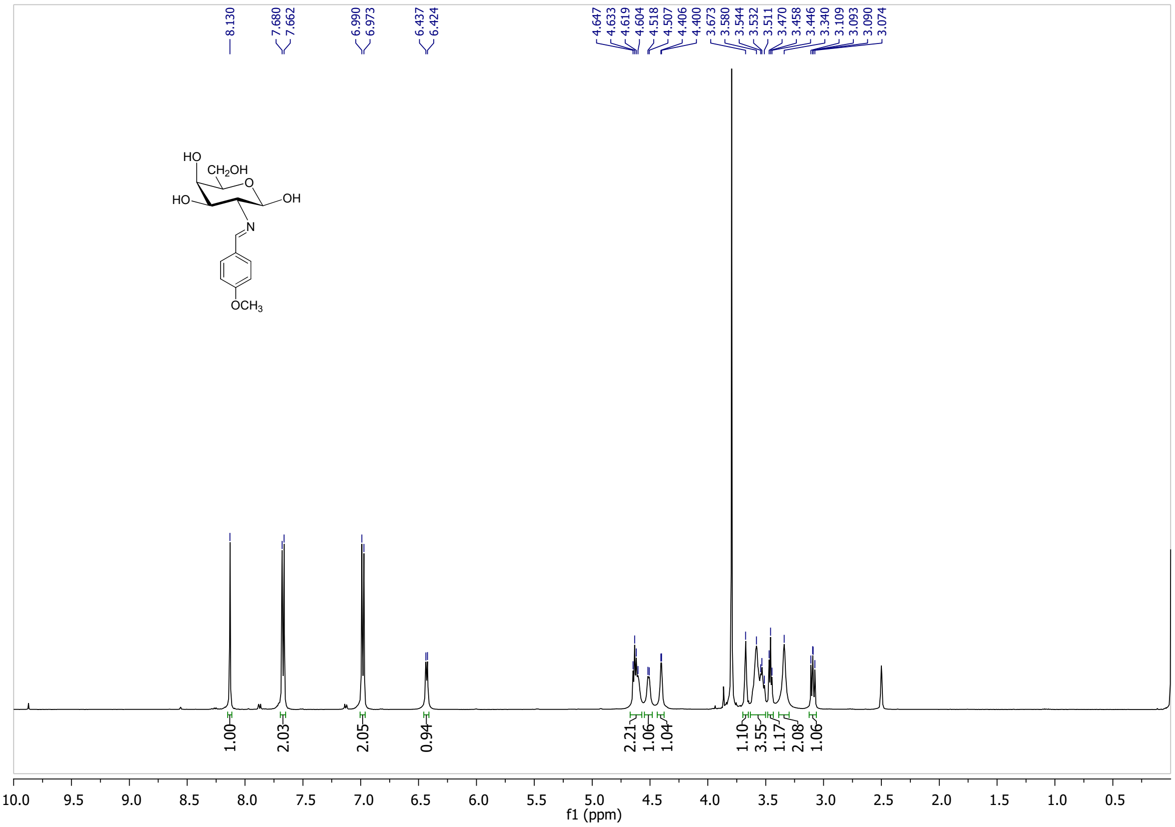

Figure S26. ^{1}H NMR spectrum of **28** (500 MHz, DMSO-d_6).

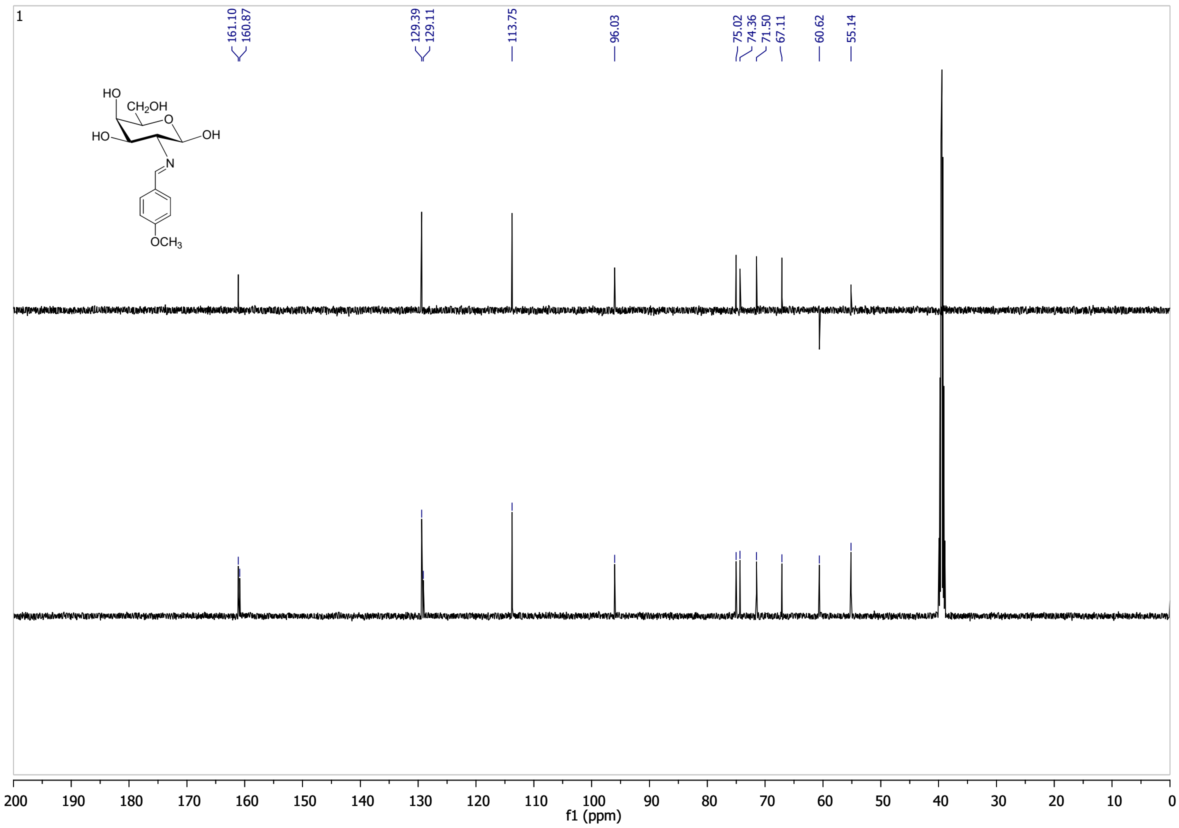

Figure S27. ^{13}C{^{1}H} NMR and DEPT spectra of **28** (125 MHz, DMSO-d_6).

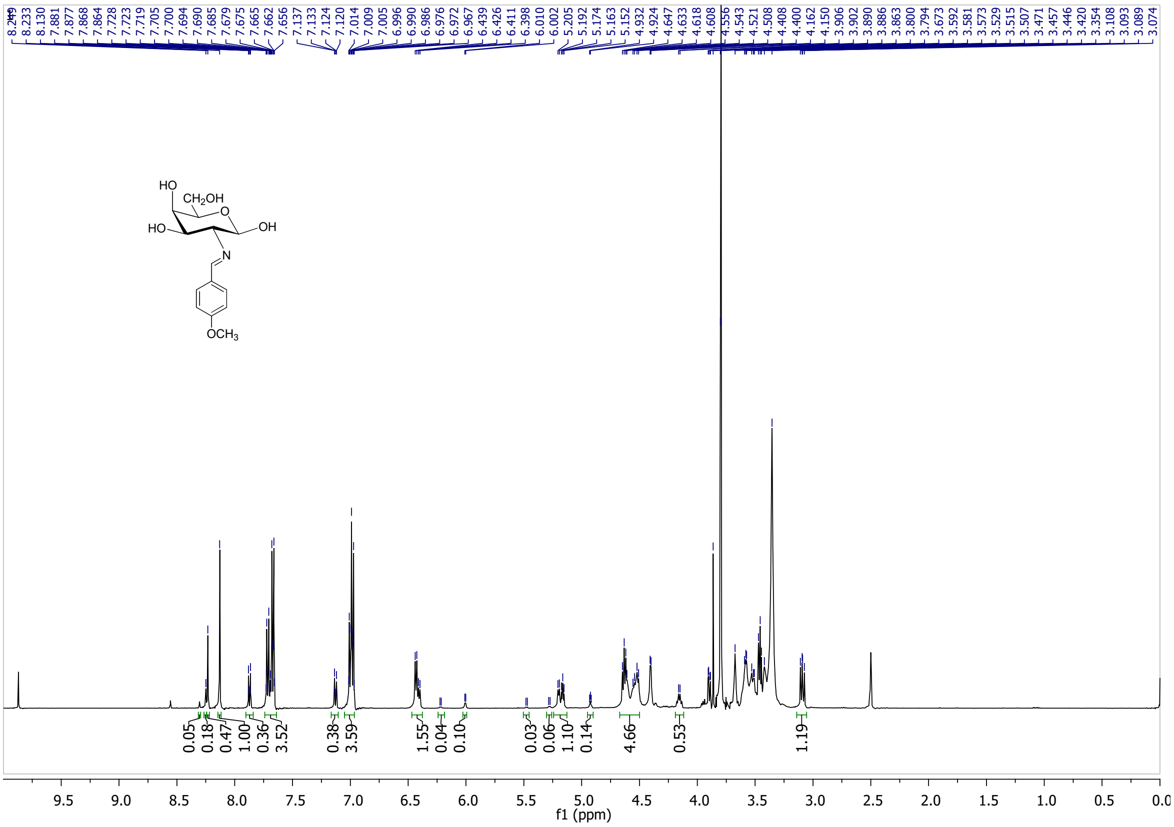

Figure S28. ^{1}H NMR spectrum of **28** in equilibrium (500 MHz, DMSO-d_6).

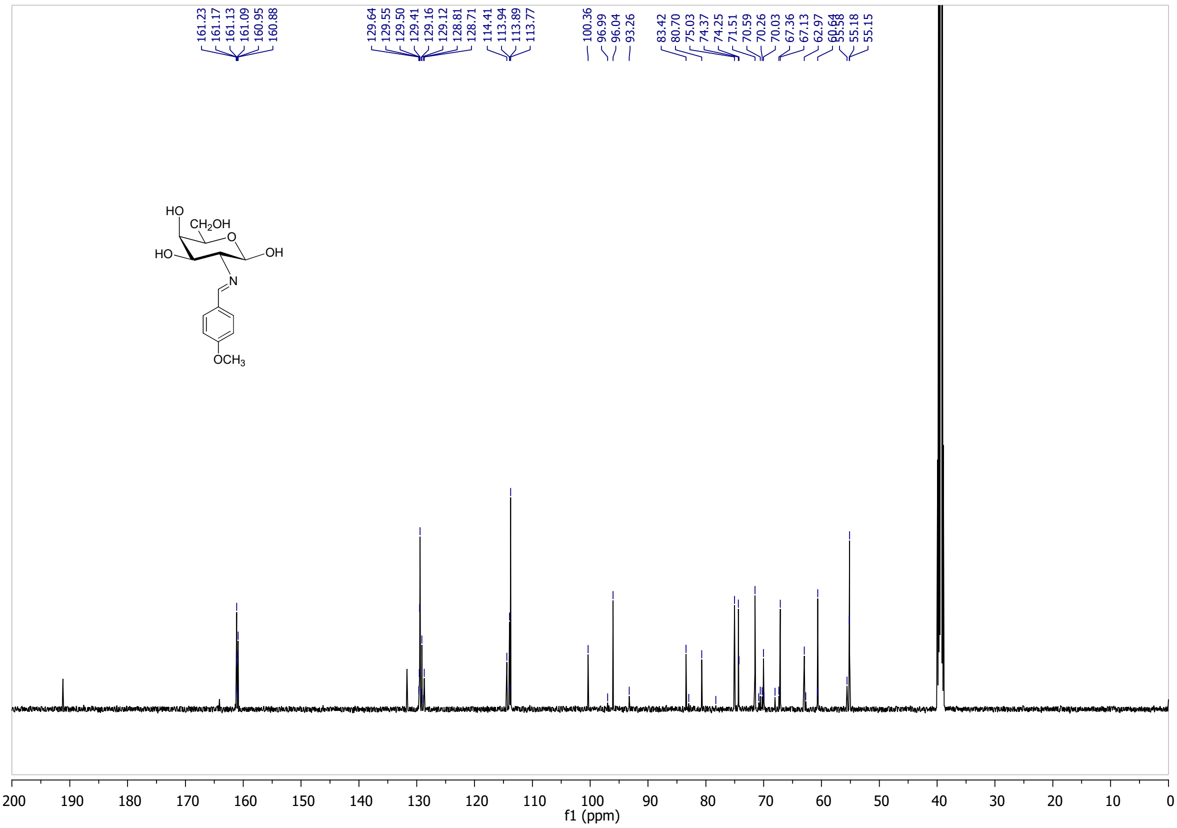

Figure S29. $^{13}C\{^{1}H\}$ NMR spectrum of **28** in equilibrium (125 MHz, DMSO-d_6).

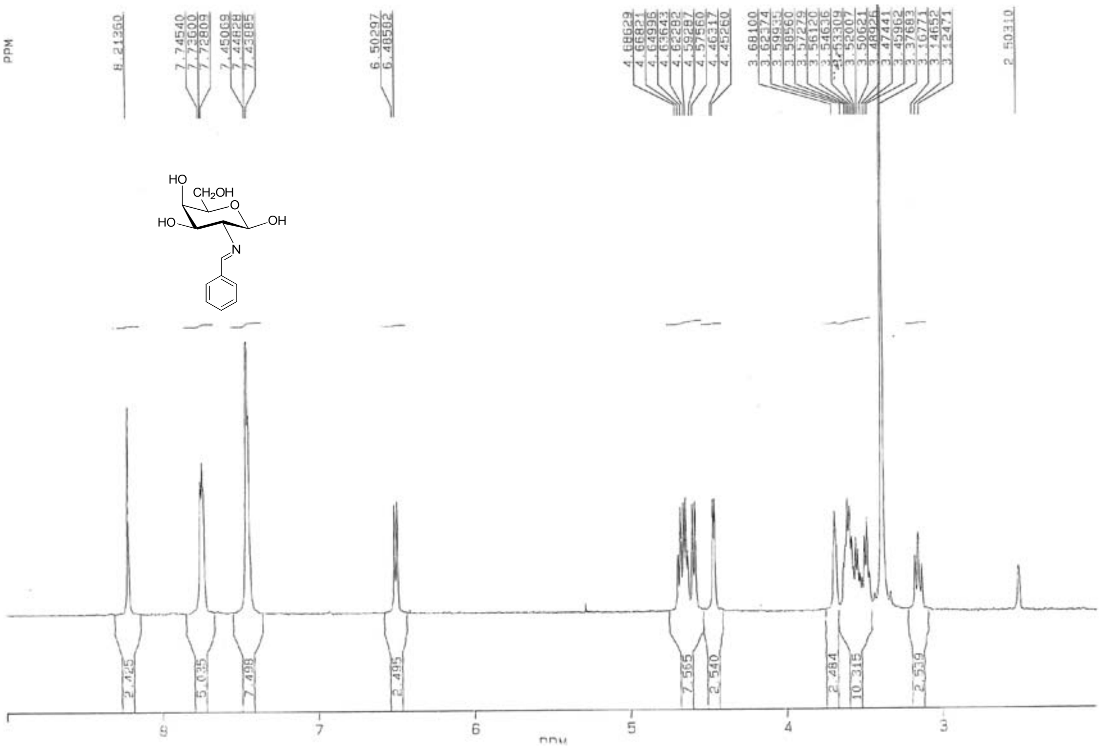

Figure S30. ^{1}H NMR spectrum of **29** (400 MHz, DMSO-d_6).

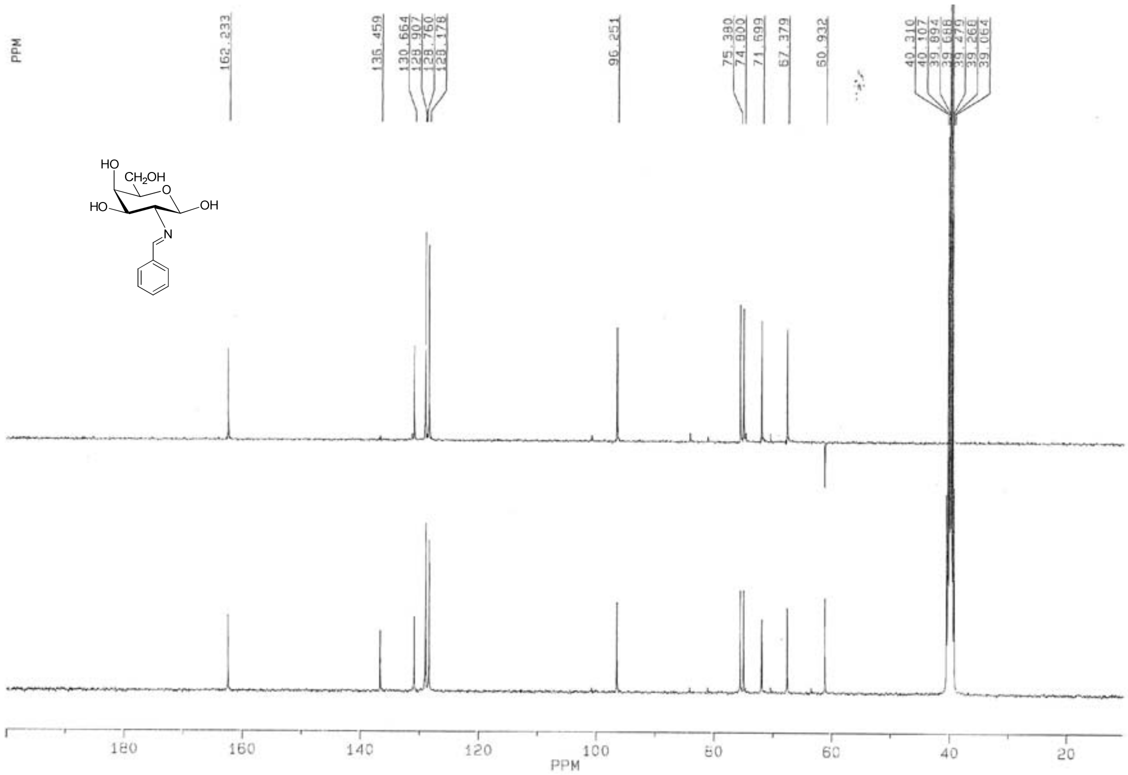

Figure S31. $^{13}C\{^{1}H\}$ NMR and DEPT spectra of **29** (100 MHz, DMSO-d_6).

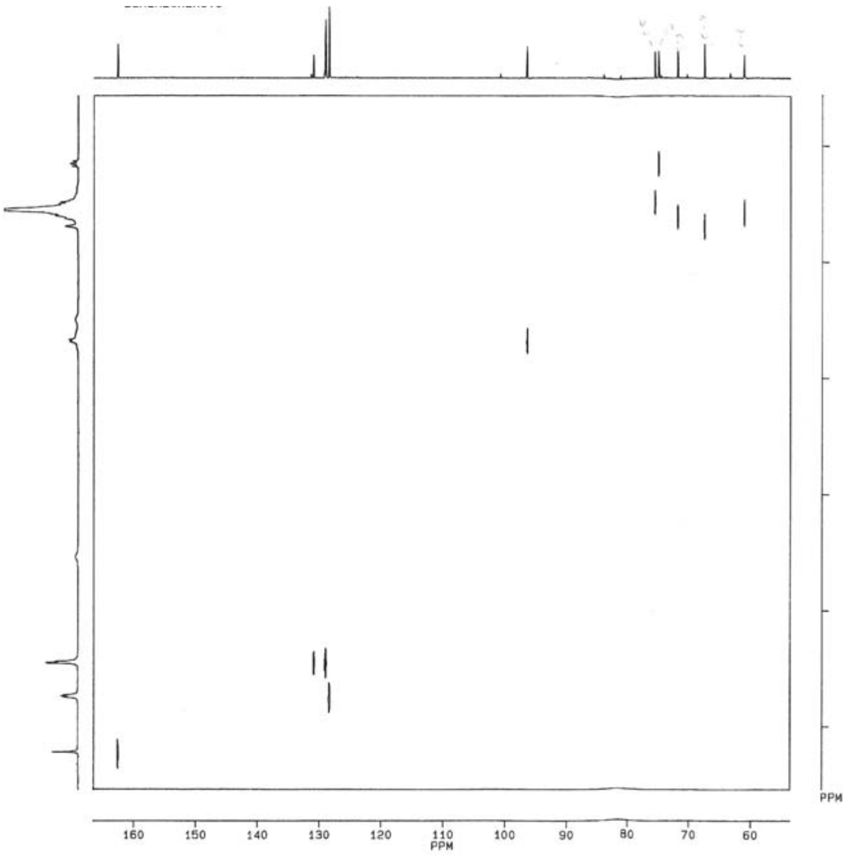

Figure S32. HMQC spectrum of **29** (400 MHz, DMSO-d_6).

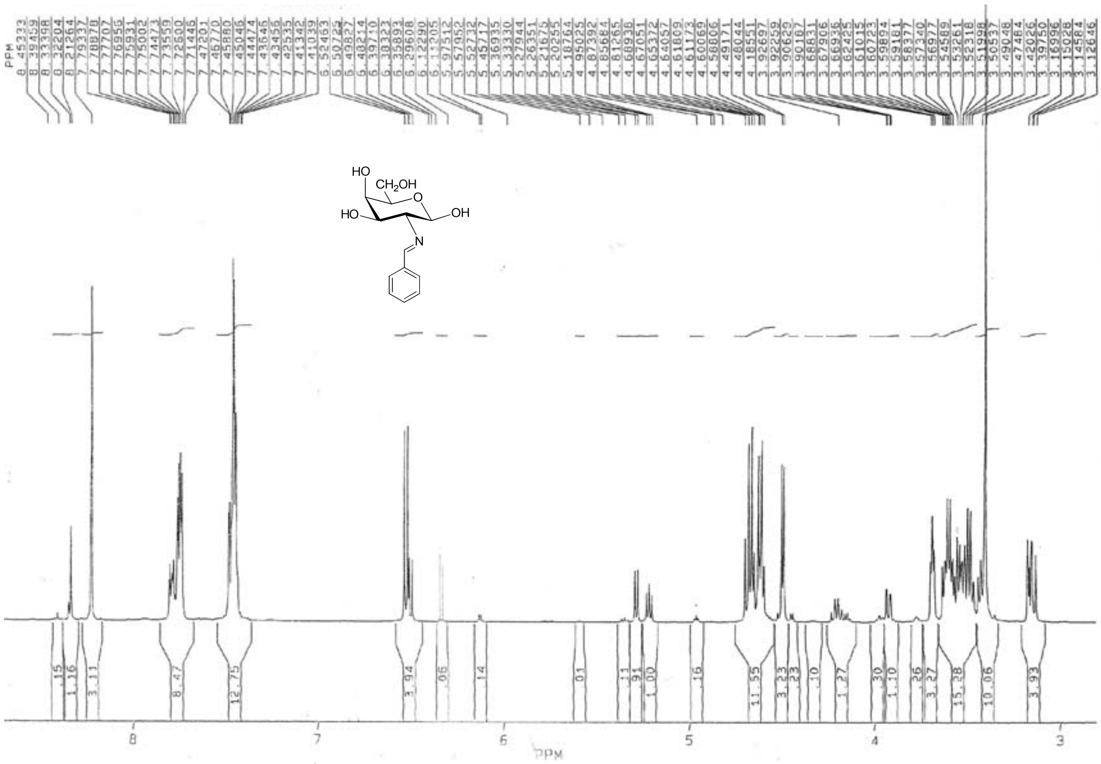

Figure S33. ^{1}H NMR spectrum of **29** in equilibrium (400 MHz, DMSO-d_6).

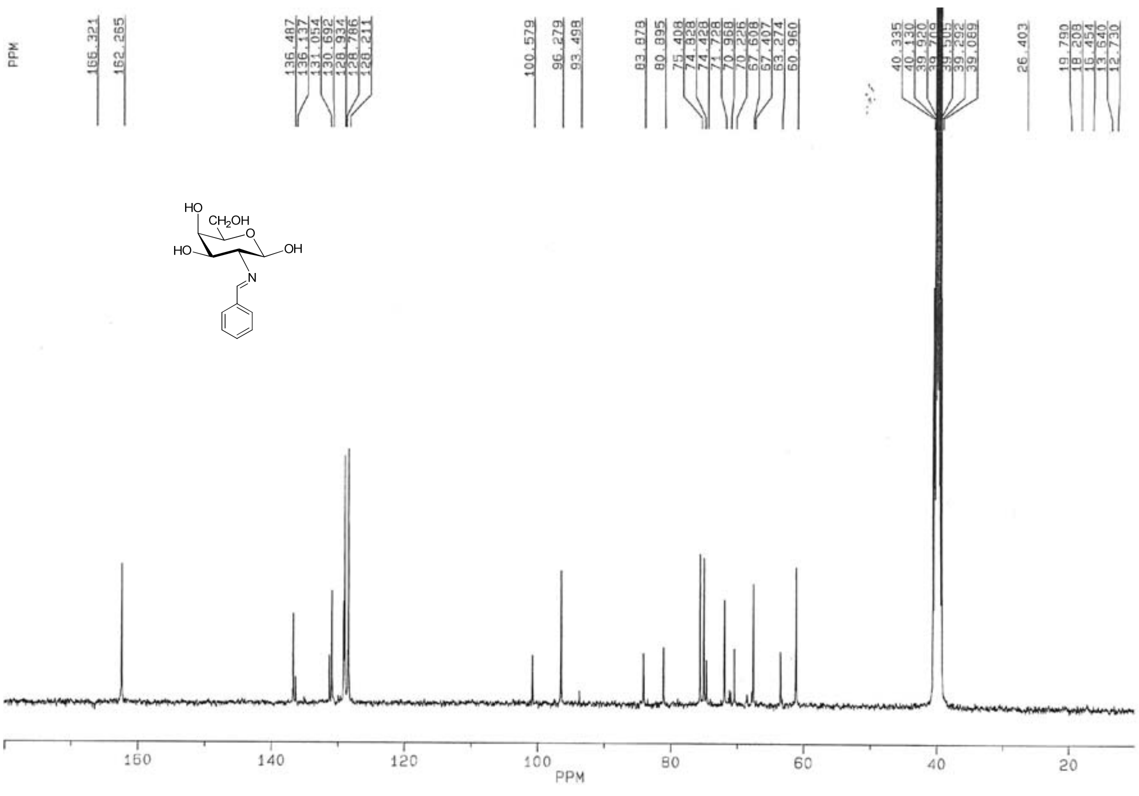

Figure S34. ^{13}C{^{1}H} NMR and DEPT spectra of **29** in equilibrium (100 MHz, DMSO-d_6).

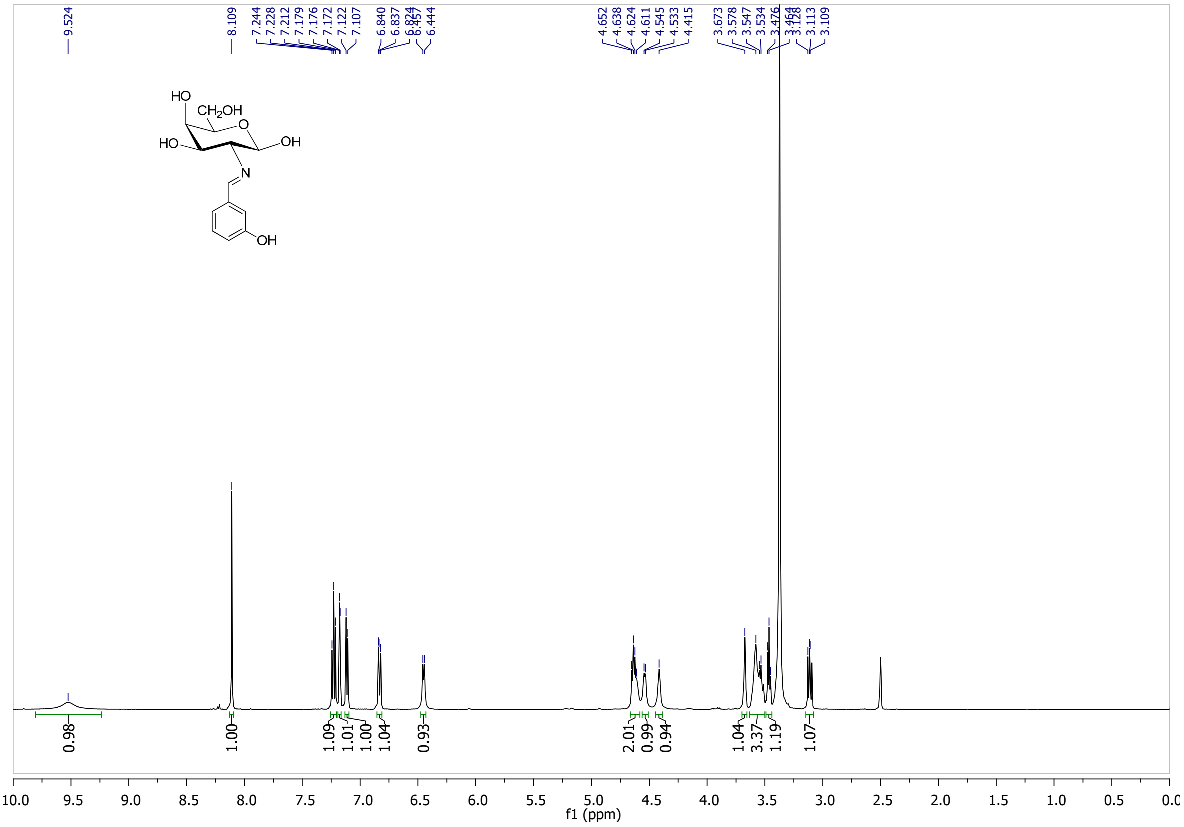

Figure S35. ^{1}H NMR spectrum of **30** (500 MHz, DMSO-d_6).

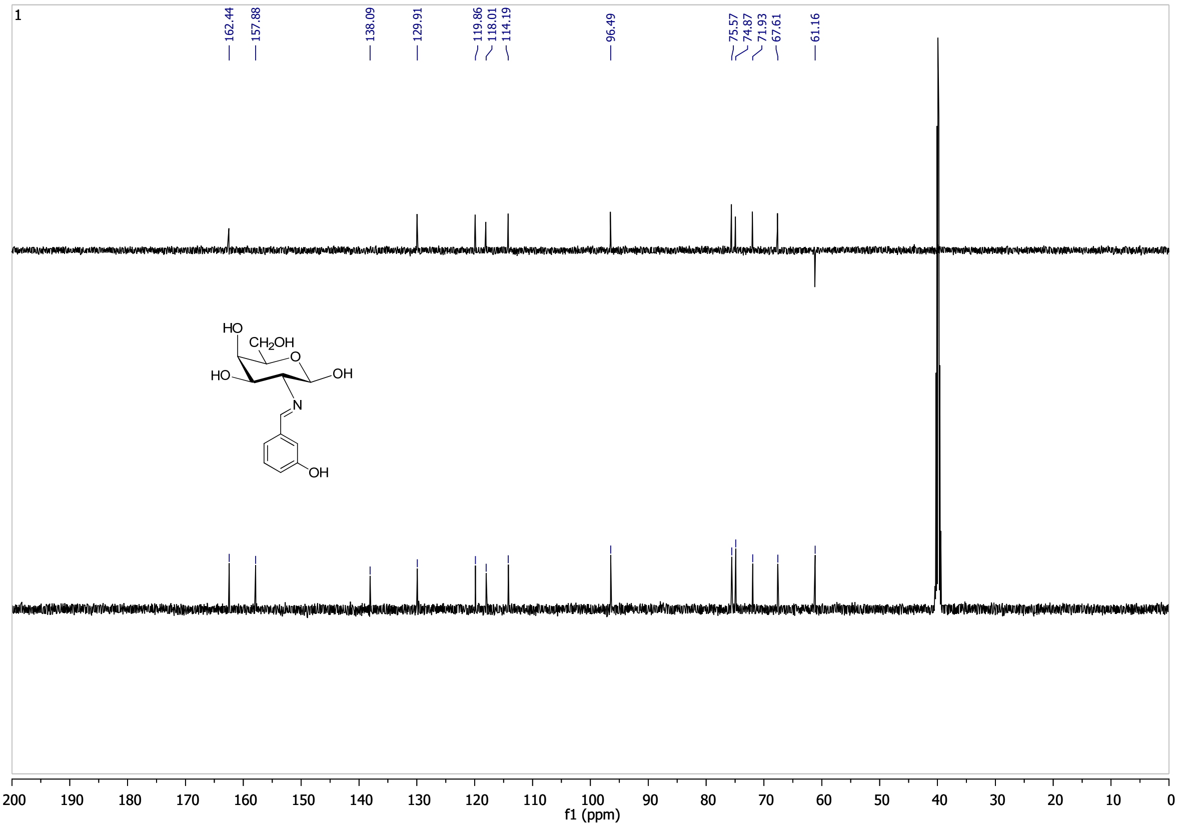

Figure S36. $^{13}C\{^{1}H\}$ NMR and DEPT spectra of **30** (125 MHz, DMSO-d_6).

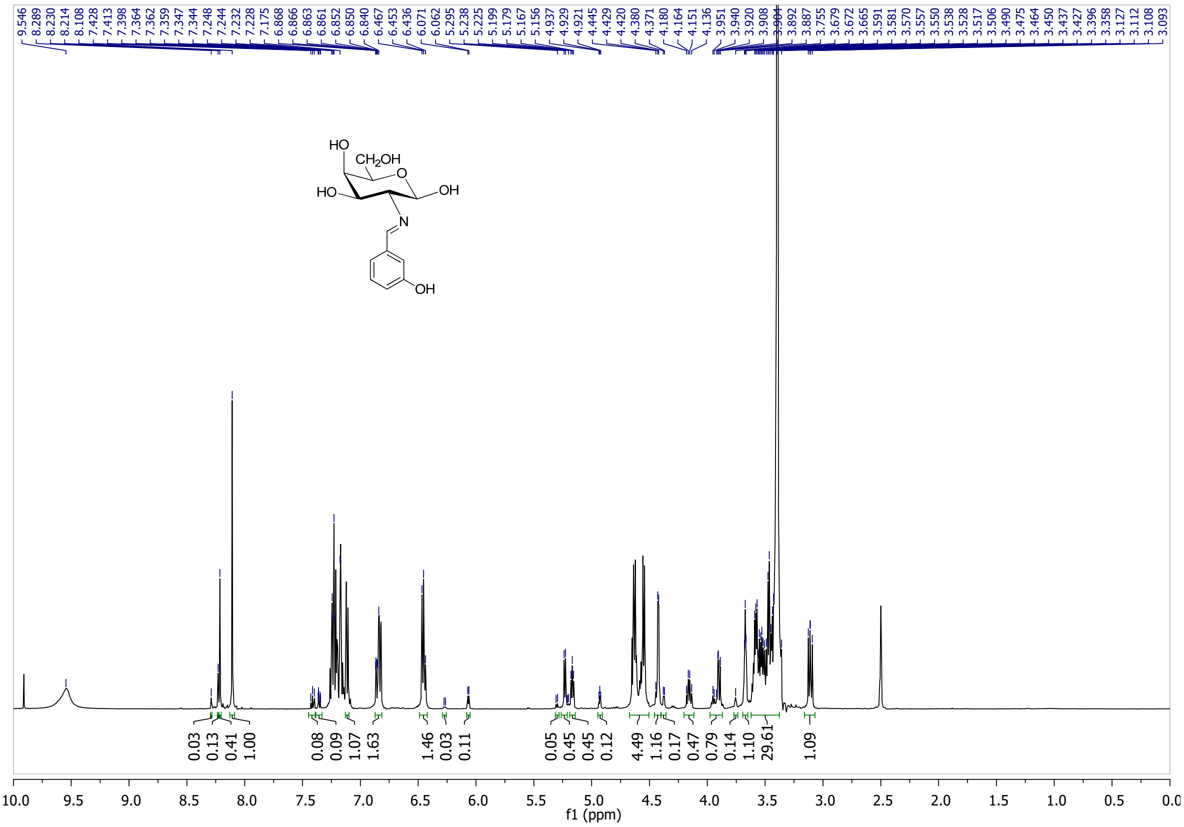

Figure S37. ^{1}H NMR spectrum of **30** in equilibrium (500 MHz, DMSO-d_6).

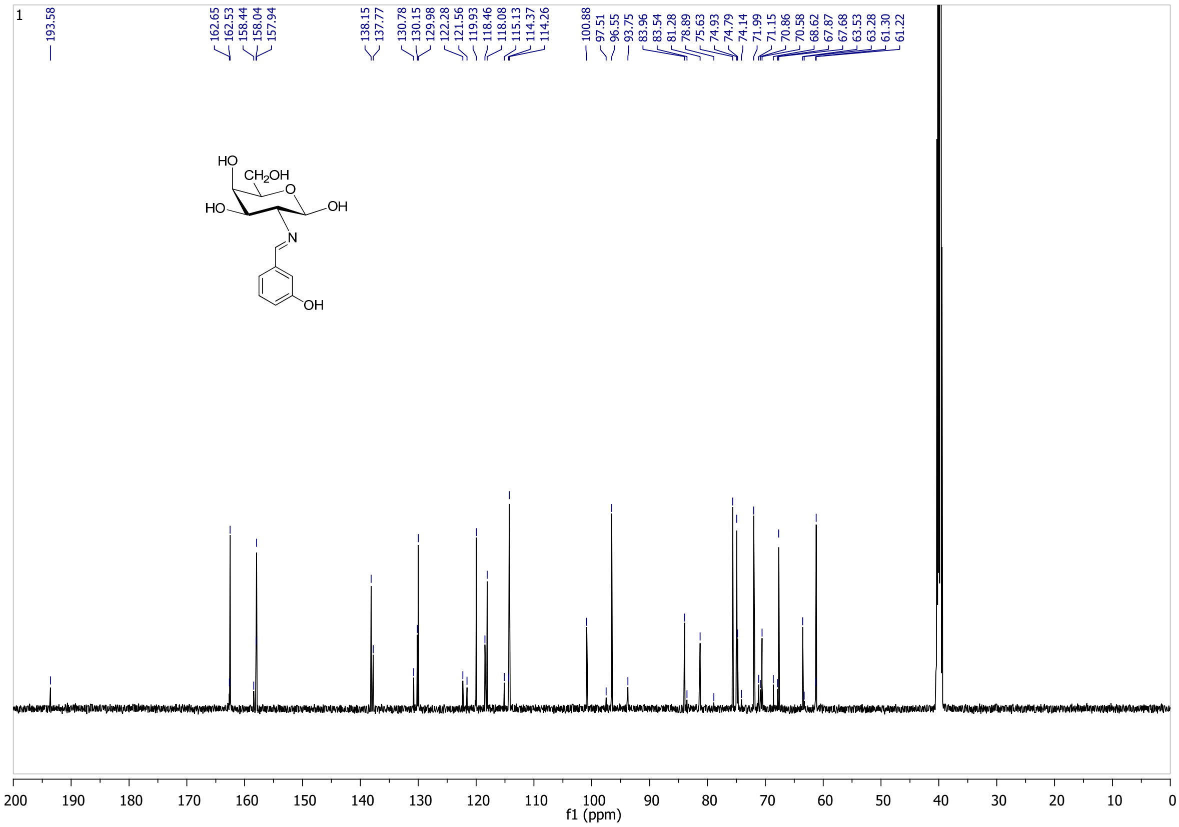

Figure S38. $^{13}C\{^{1}H\}$ NMR spectrum of **30** in equilibrium (125 MHz, DMSO-d_6).

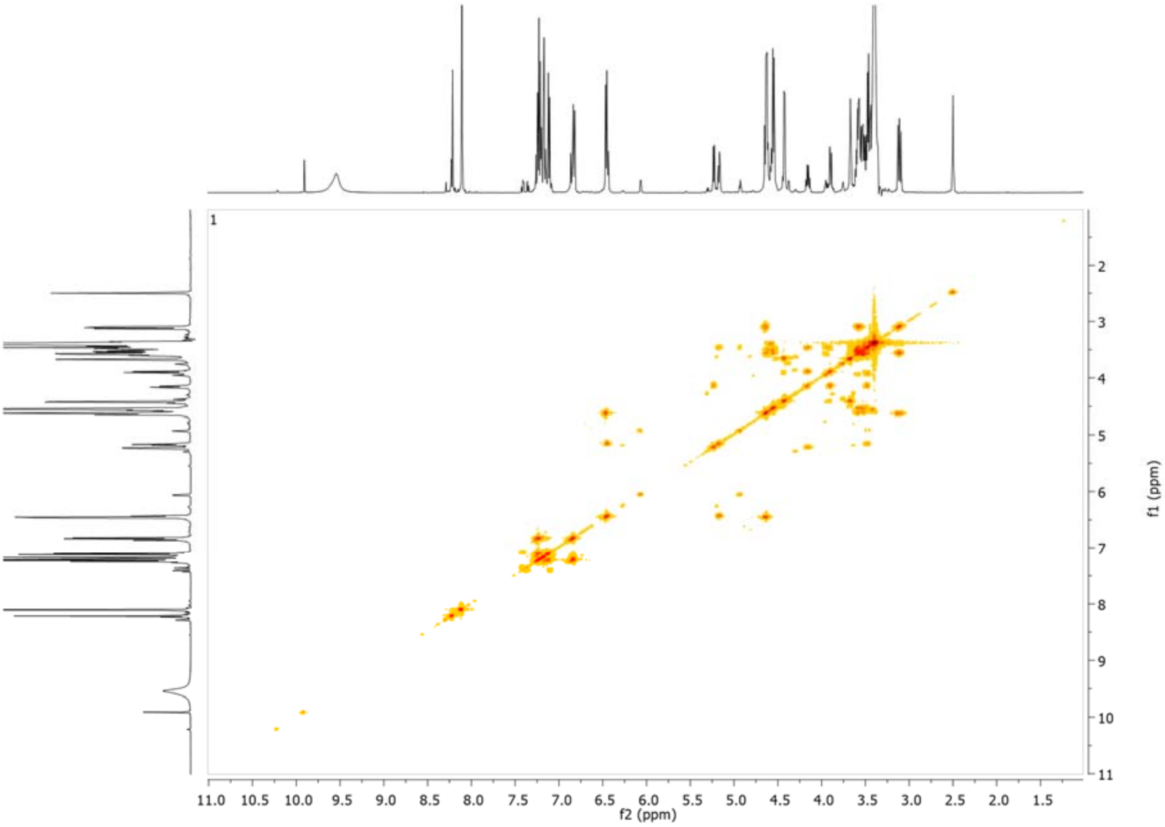

Figure S39. COSY spectrum of **30** in equilibrium (500 MHz, DMSO-d_6).

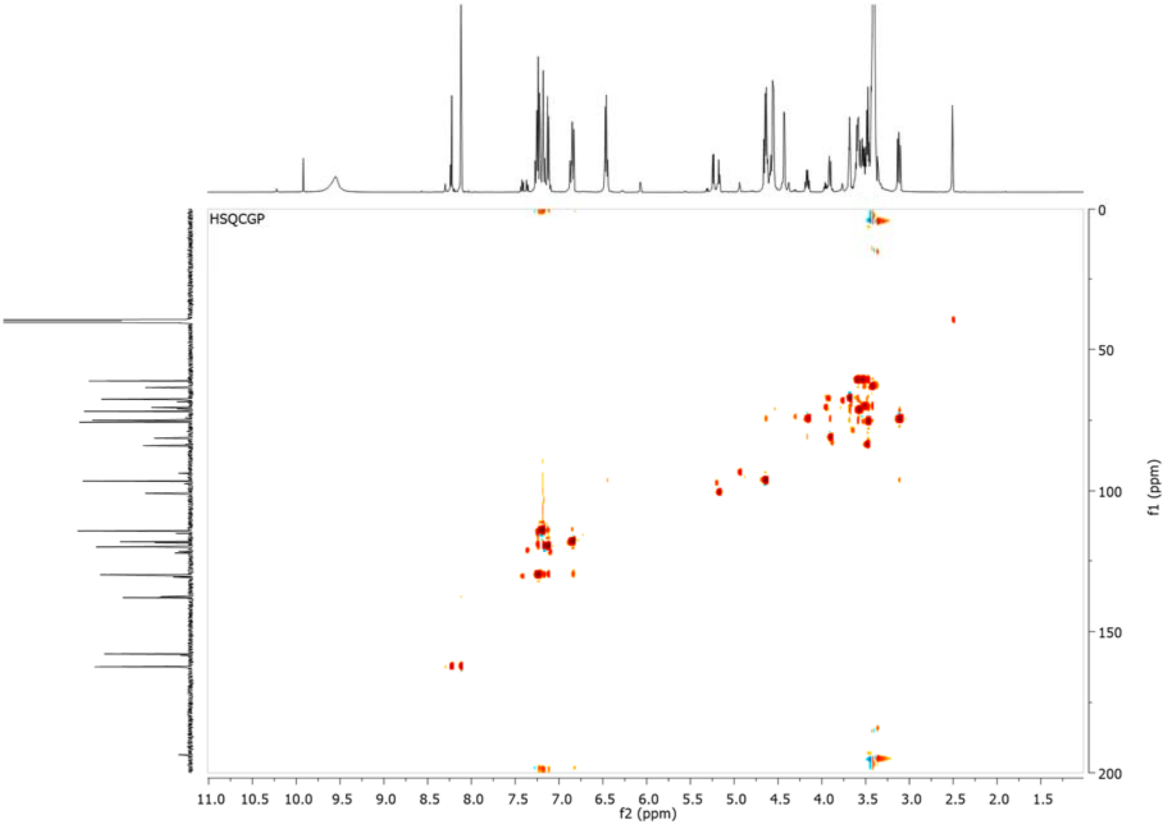

Figure S40. HSQC spectrum of **30** in equilibrium (500 MHz, DMSO-d_6).

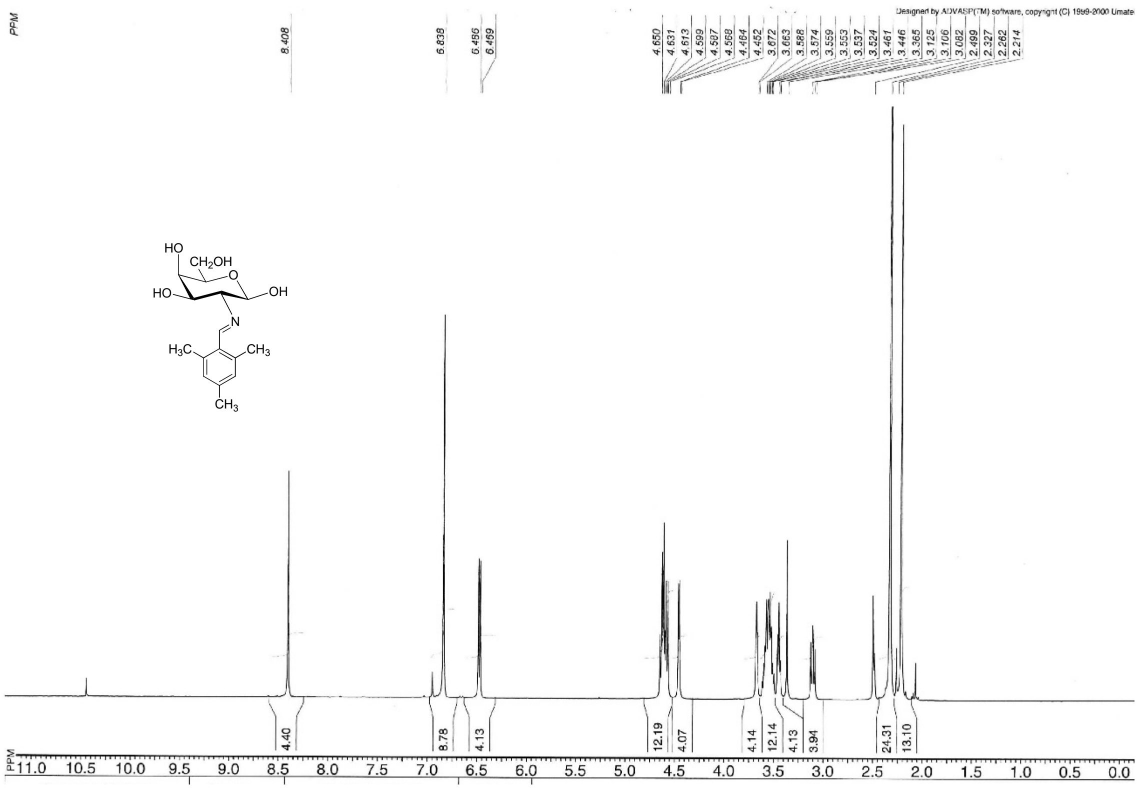

Figure S41. ^{1}H NMR spectrum of **31** (400 MHz, DMSO-d_6).

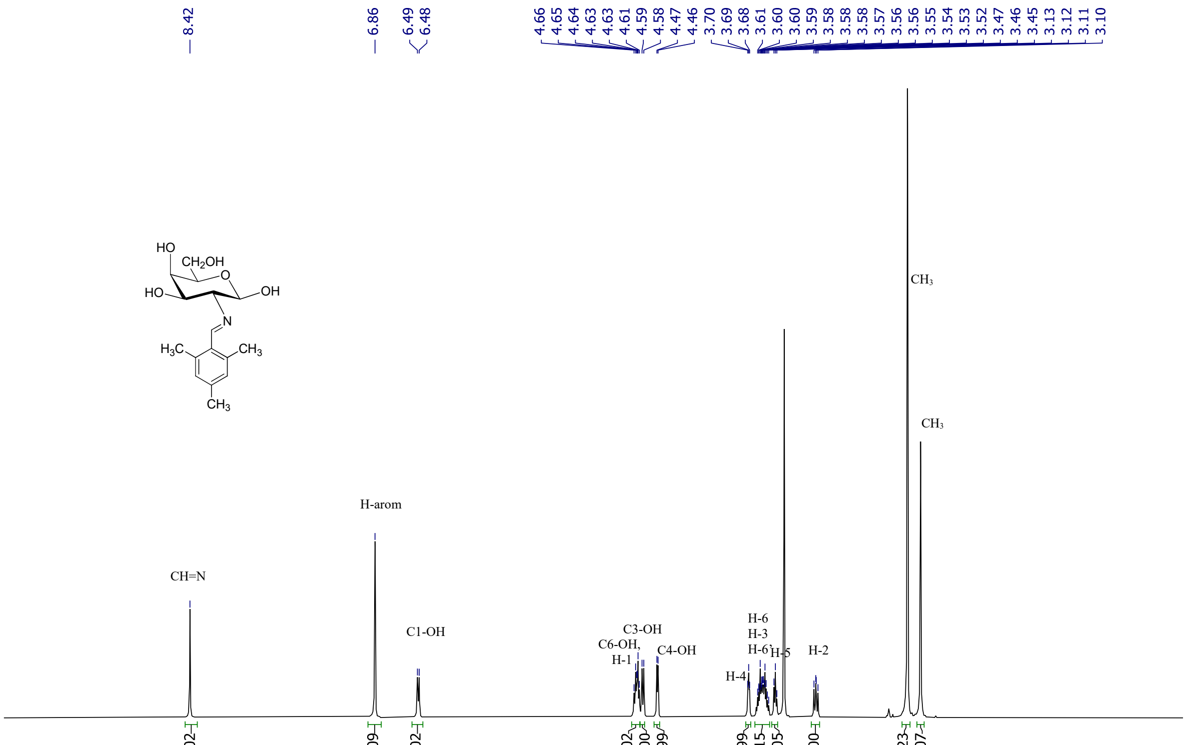

Figure S42. ^{1}H NMR spectrum of **31** (500 MHz, DMSO-d_6).

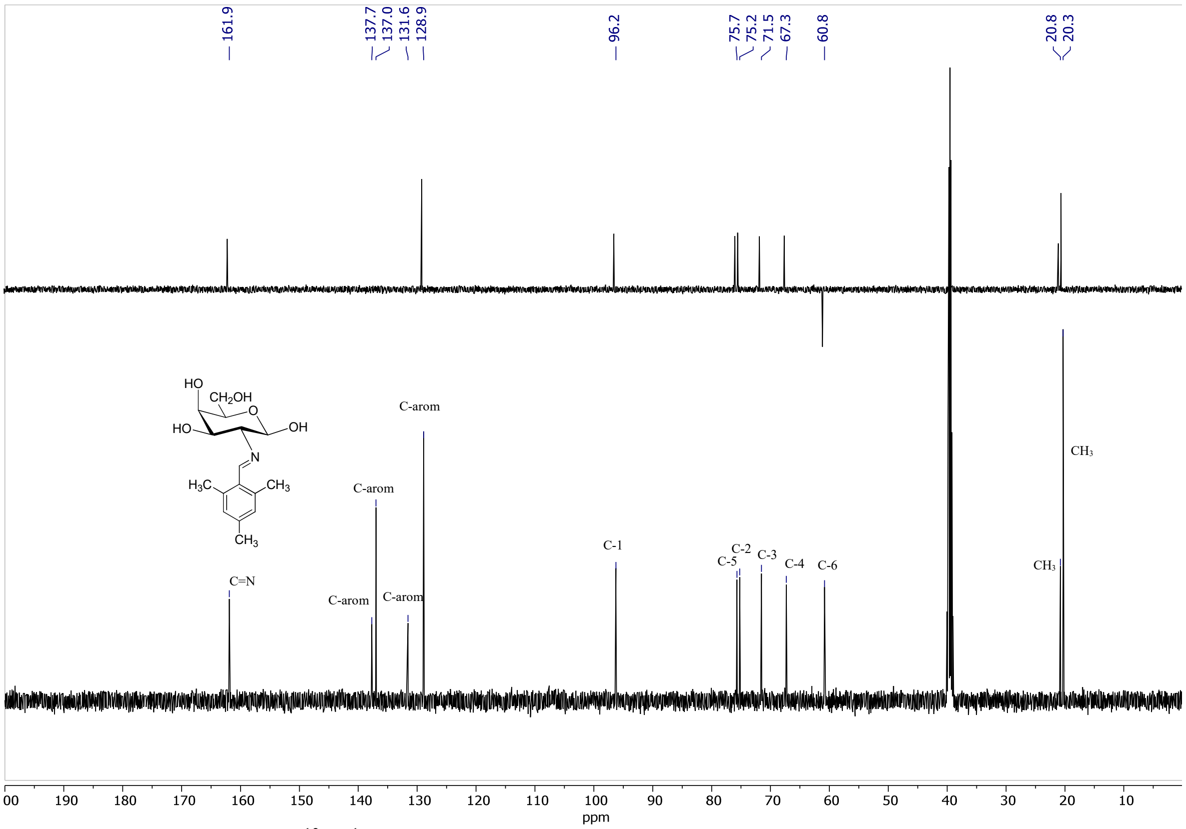

Figure S43. $^{13}C\{^1H\}$ NMR and DEPT spectra of **31** (125 MHz, DMSO-d_6).

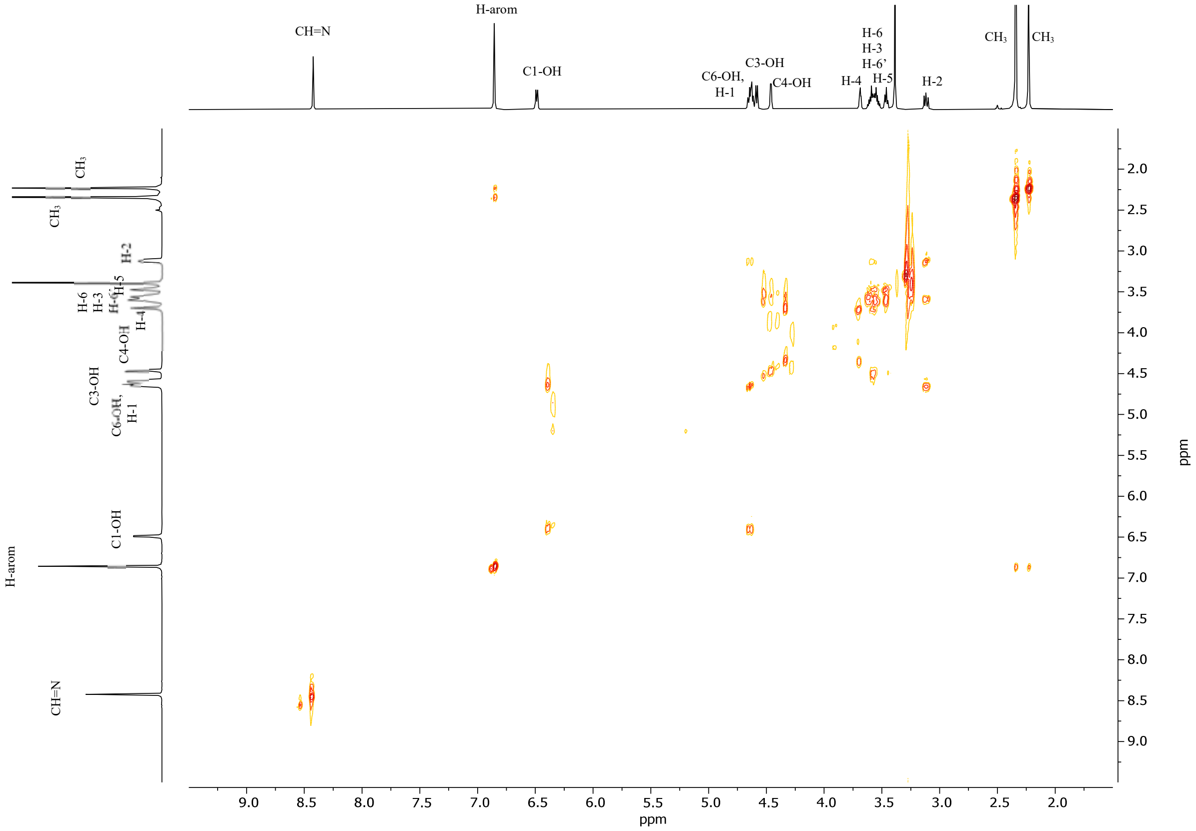

Figure S44. COSY spectrum of **31** in equilibrium (500 MHz, DMSO-d_6).

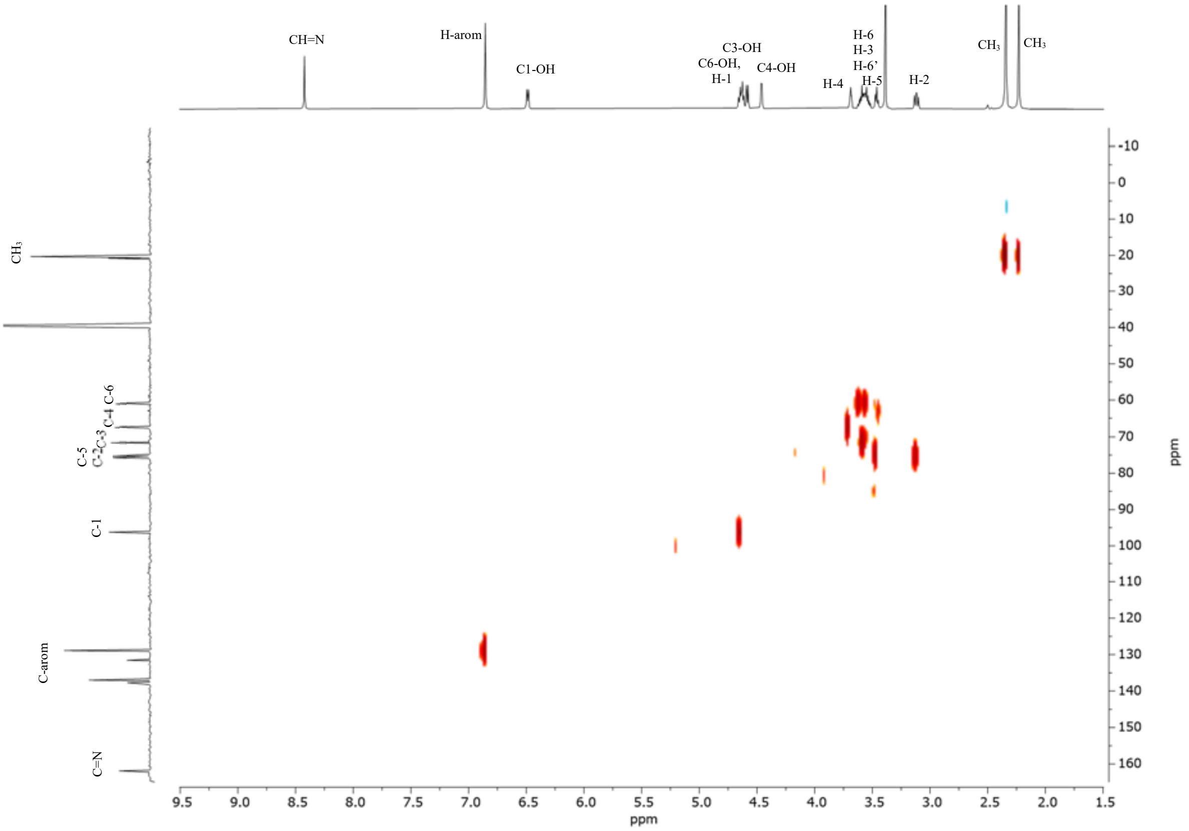

Figure S45. HSQC spectrum of **31** in equilibrium (500 MHz, DMSO-d_6).

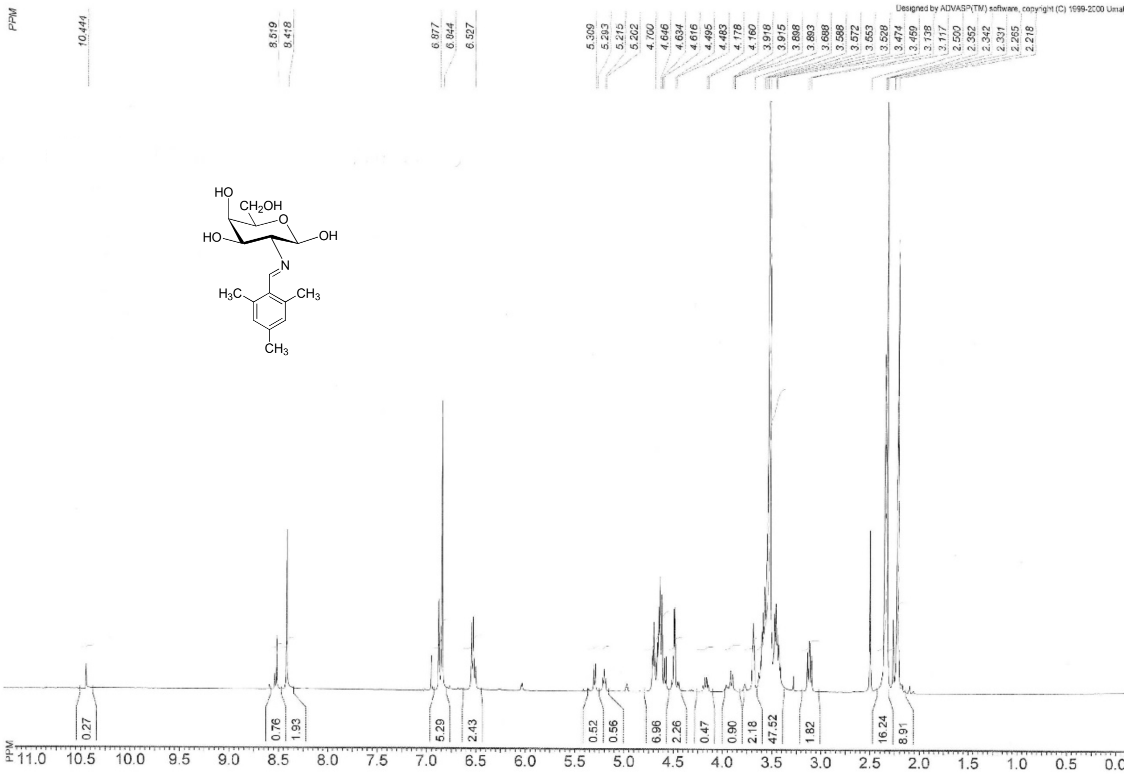

Figure S46. ^{1}H NMR spectrum of **31** in equilibrium (400 MHz, DMSO-d_6).

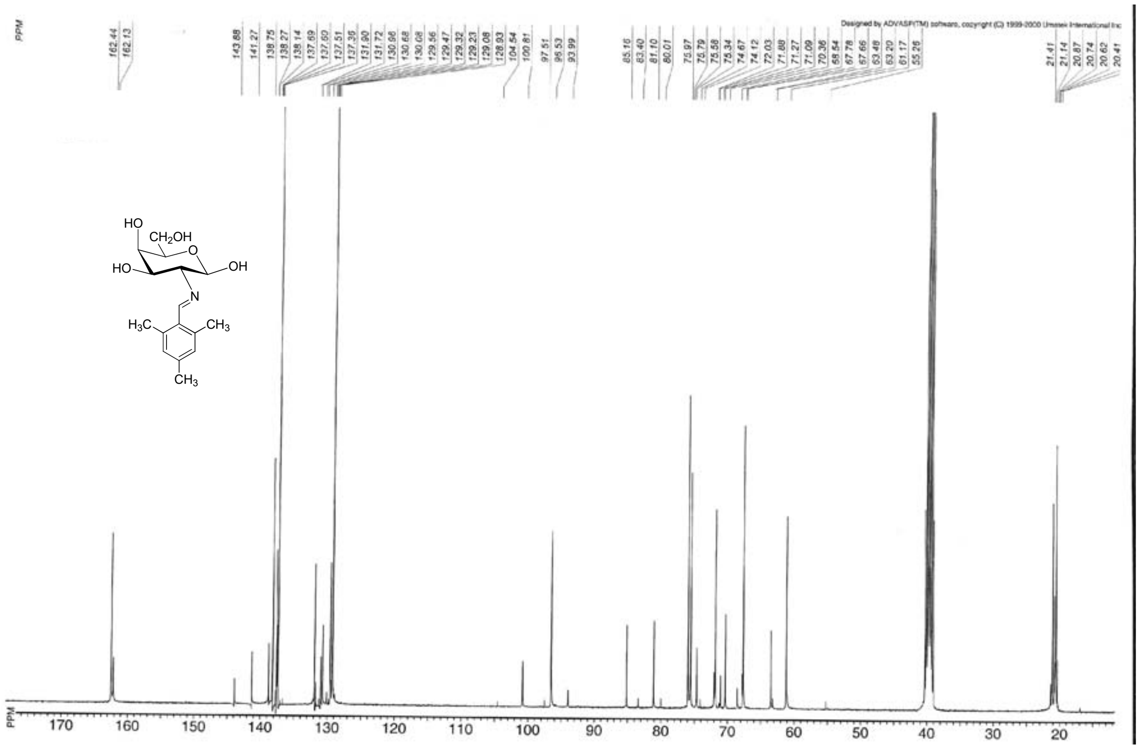

Figure S47. $^{13}C\{^1H\}$ NMR spectrum of **31** in equilibrium (100 MHz, DMSO-d_6).

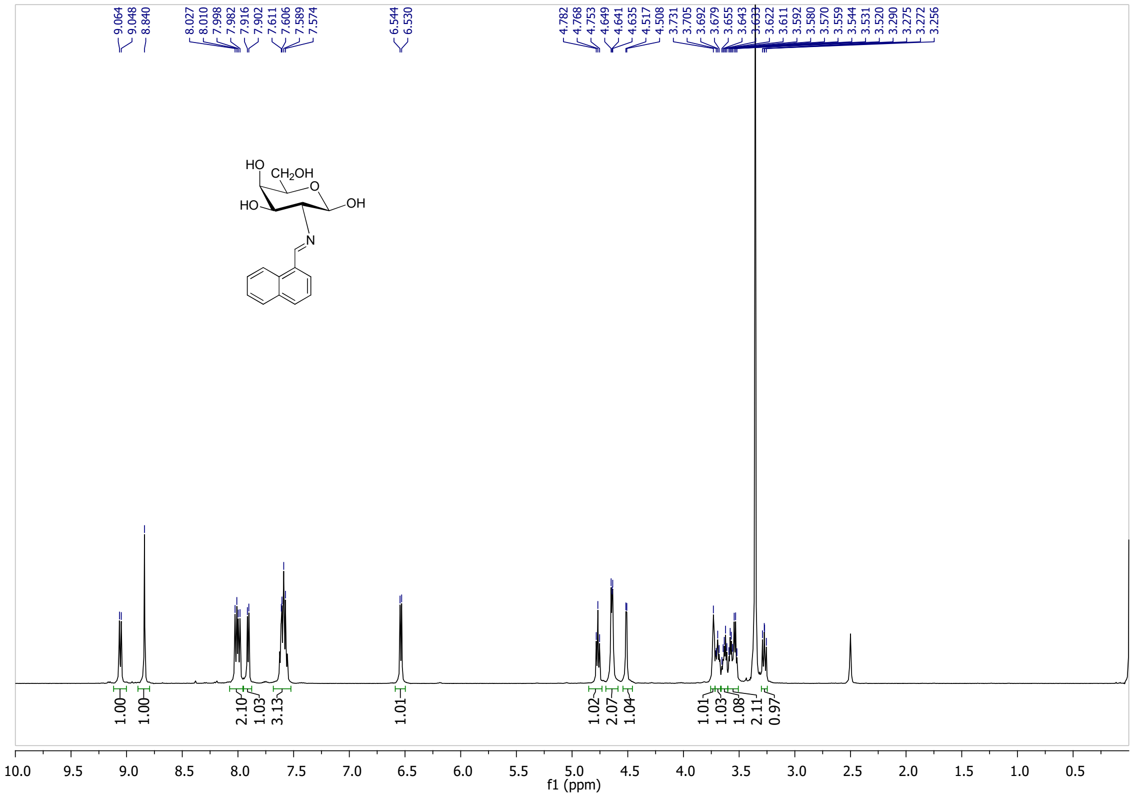

Figure S48. ^1H NMR spectrum of **32** (500 MHz, DMSO-d_6).

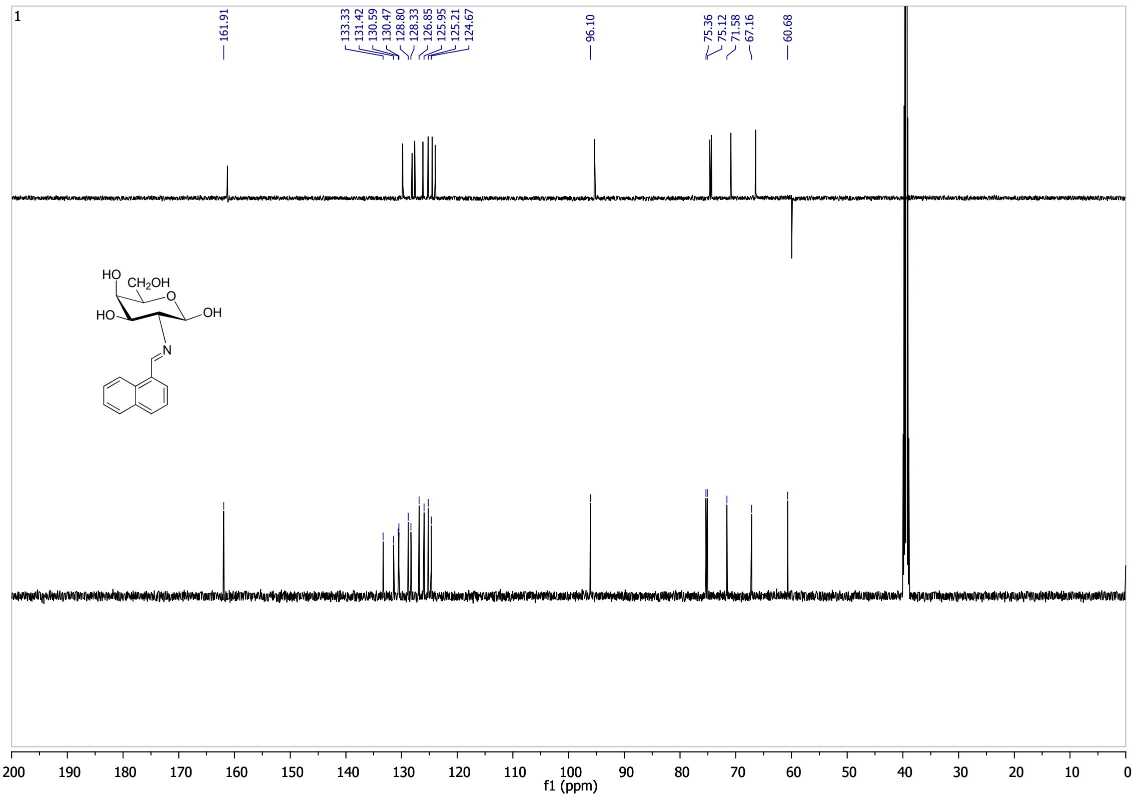

Figure S49. $^{13}C\{^{1}H\}$ NMR and DEPT spectra of **32** (125 MHz, DMSO-d_6).

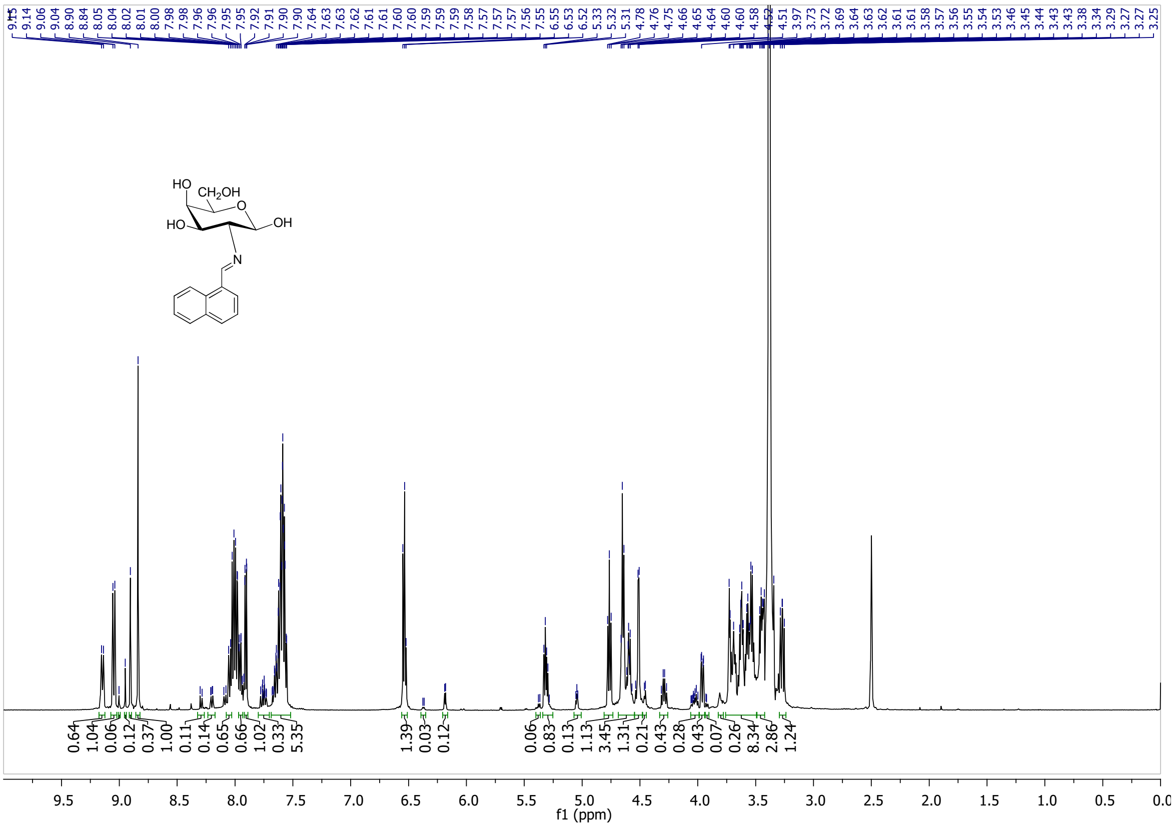

Figure S50. ^{1}H NMR spectrum of **32** in equilibrium (500 MHz, DMSO-d_6).

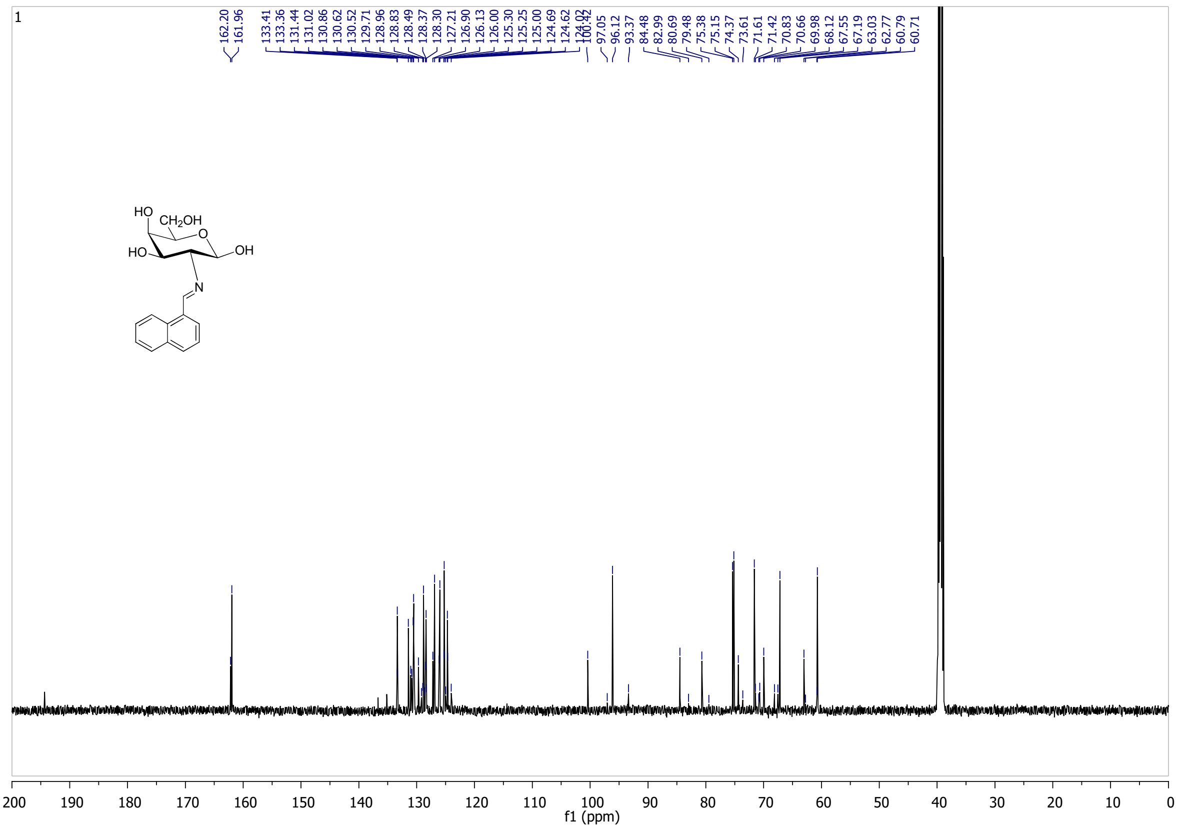

Figure S51. $^{13}C\{^1H\}$ NMR spectrum of **32** in equilibrium (125 MHz, DMSO-d_6).

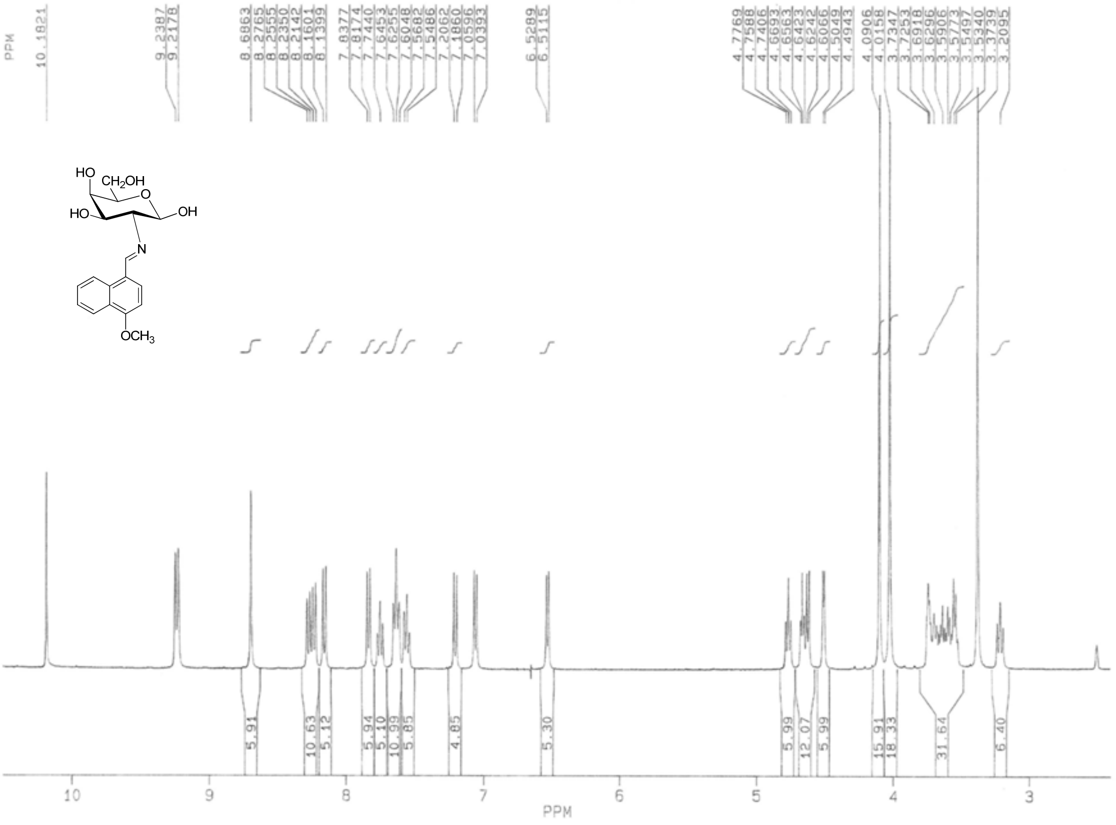

Figure S52. ^1H NMR spectrum of **33** (400 MHz, DMSO-d_6).

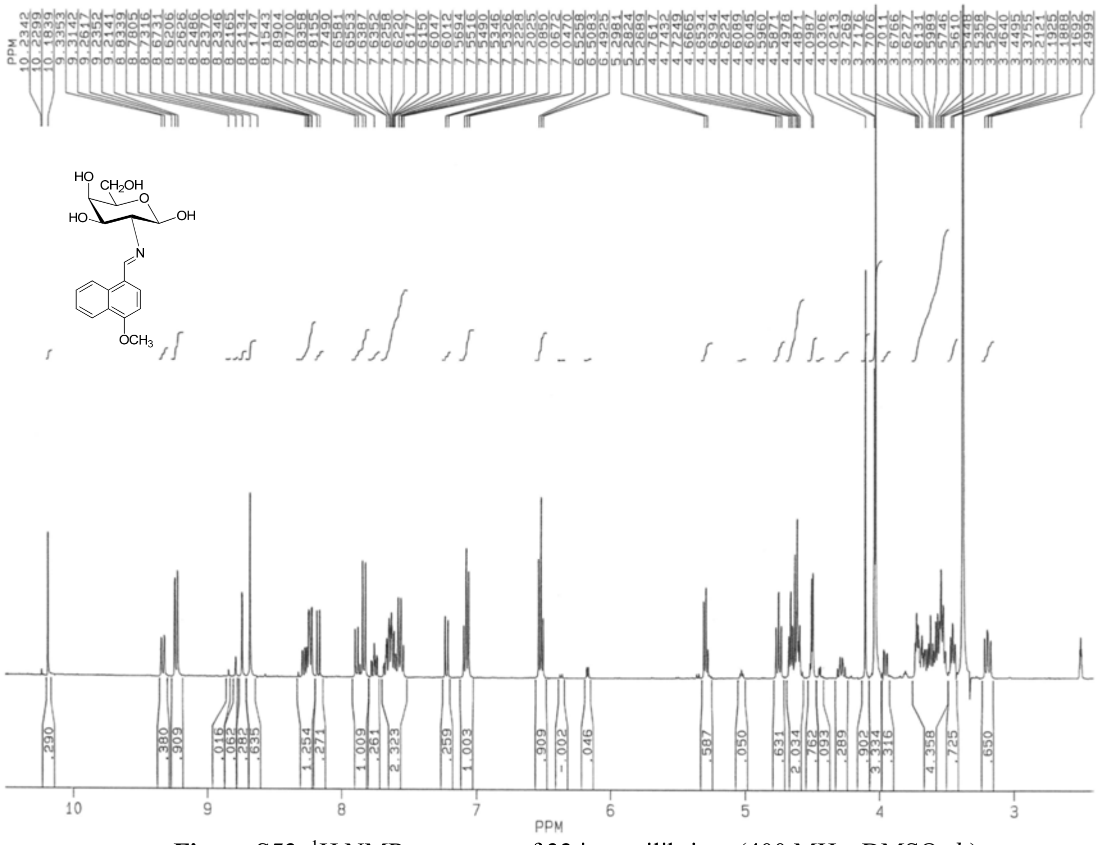

Figure S53. ^{1}H NMR spectrum of **33** in equilibrium (400 MHz, DMSO-d_6).

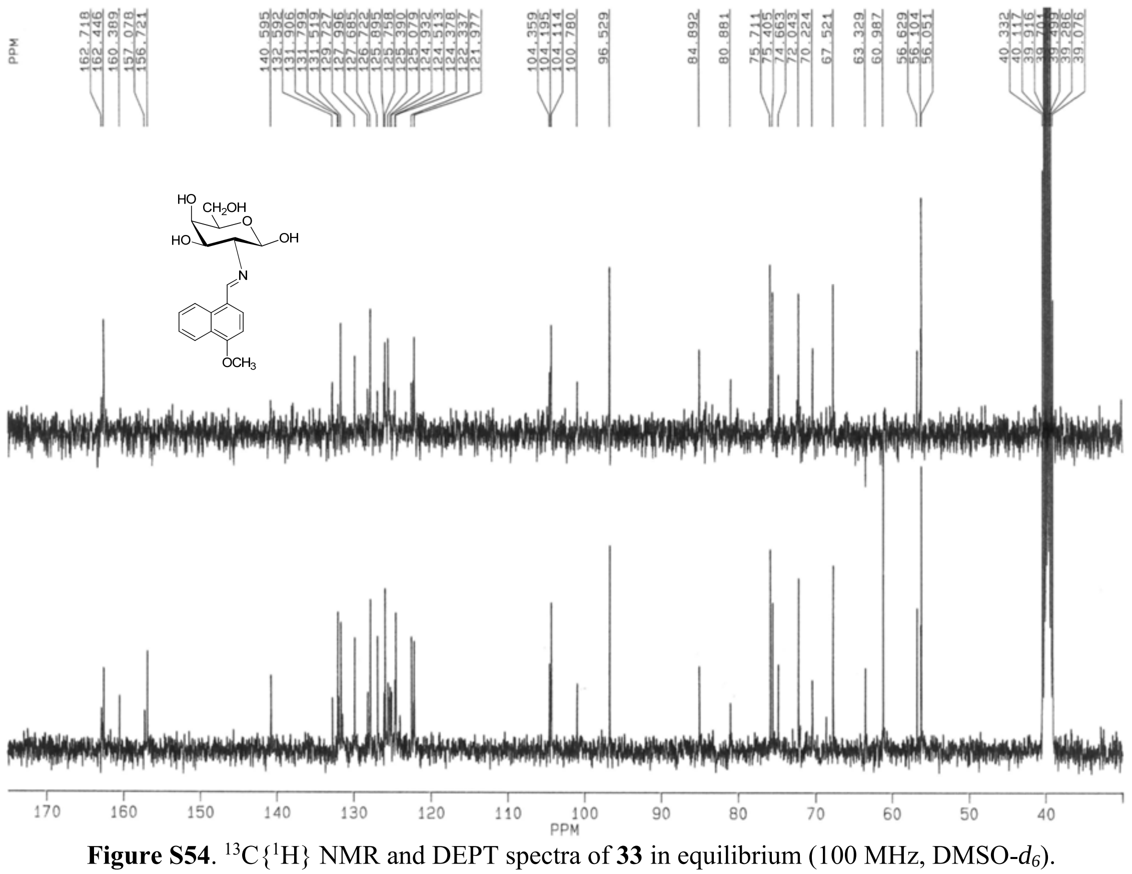

Figure S54. $^{13}C\{^{1}H\}$ NMR and DEPT spectra of **33** in equilibrium (100 MHz, DMSO-d_6).

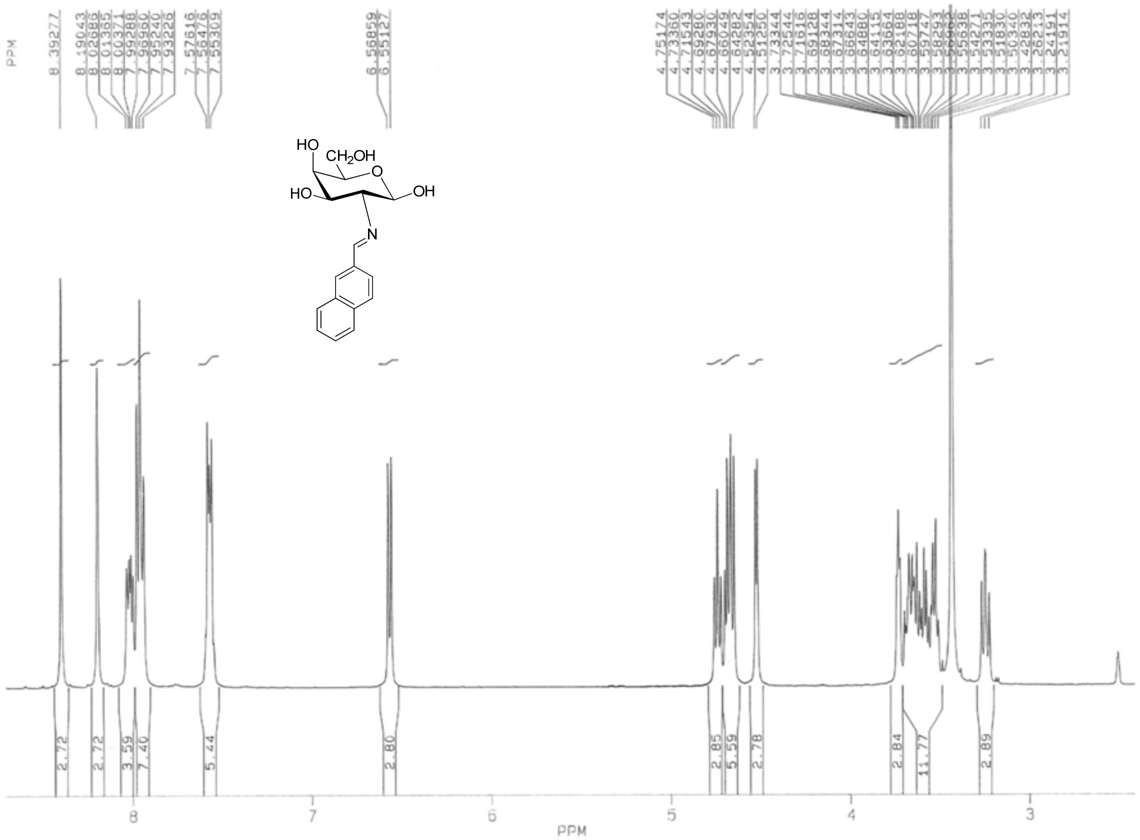

Figure S55. ^{1}H NMR spectrum of **34** (400 MHz, DMSO-d_6).

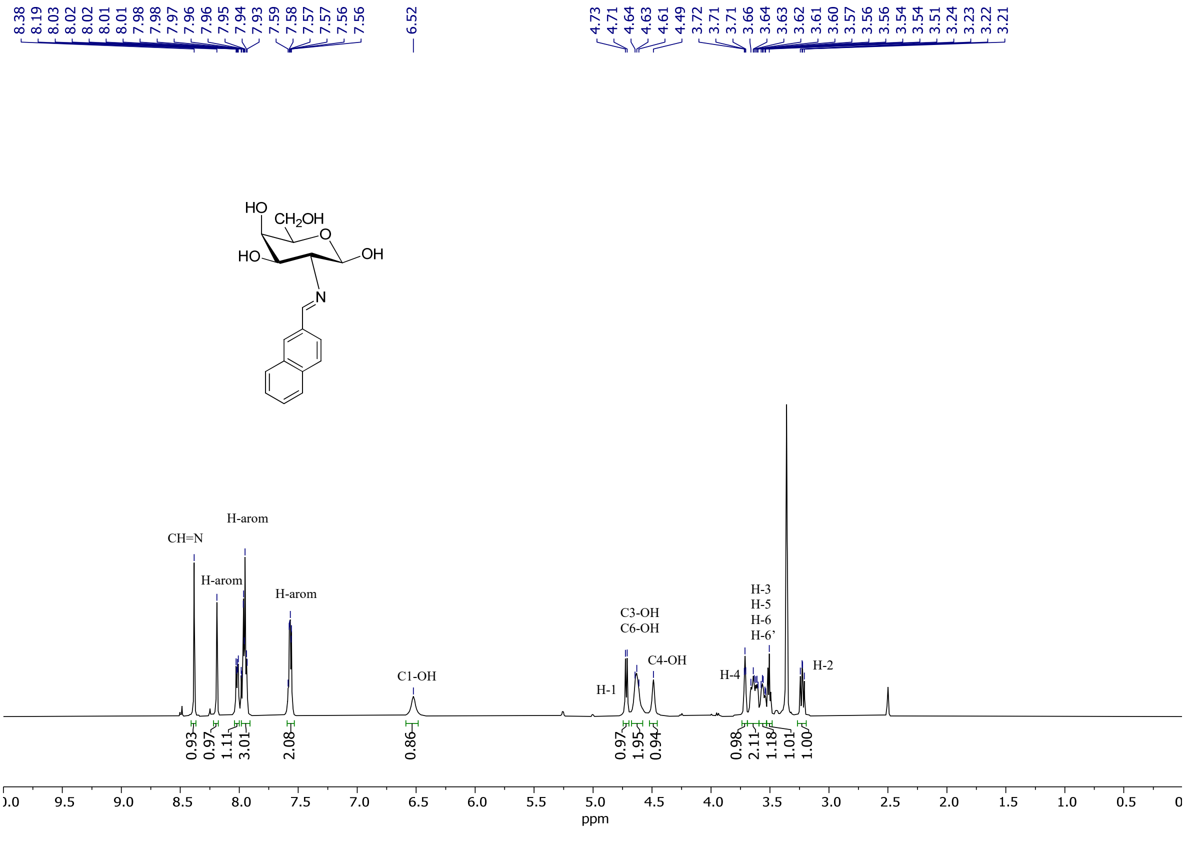

Figure S56. ^{1}H NMR spectrum of **34** (500 MHz, DMSO-d_6).

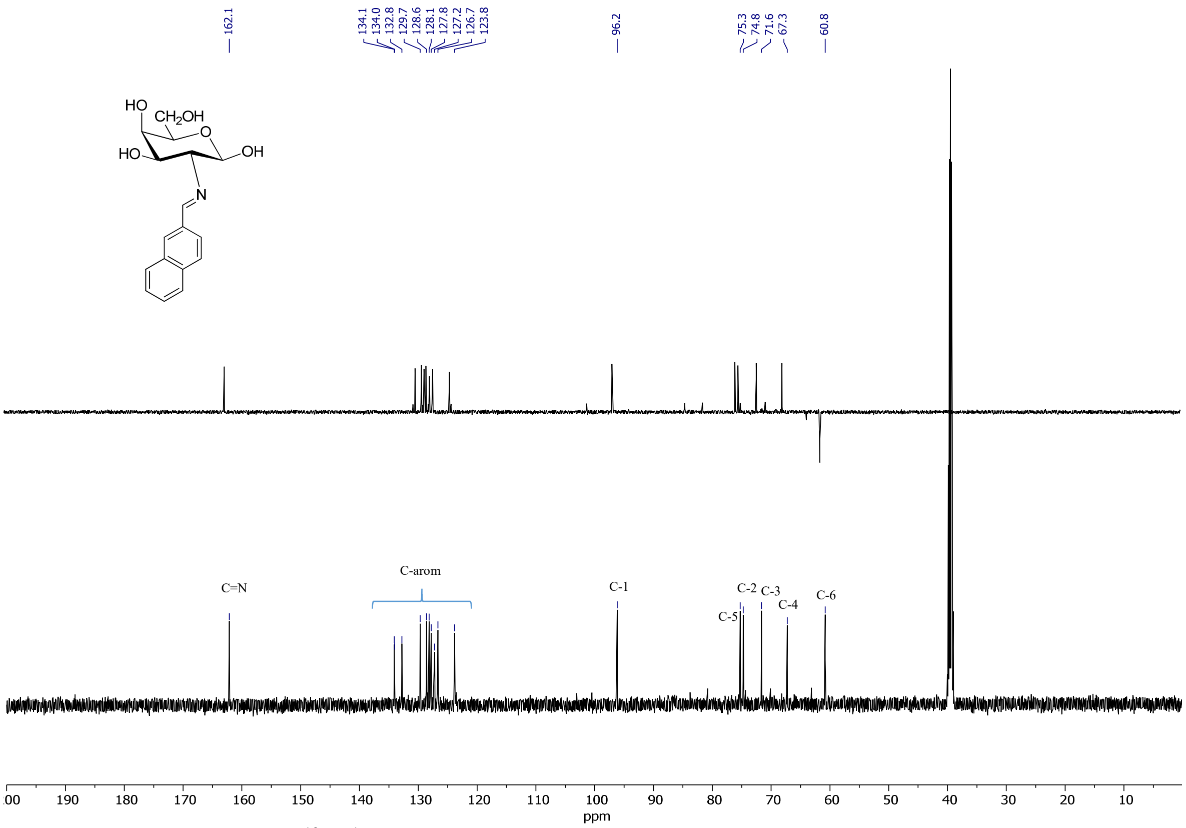

Figure S57. $^{13}C\{^{1}H\}$ NMR and DEPT spectra of **34** (125 MHz, DMSO-d_6).

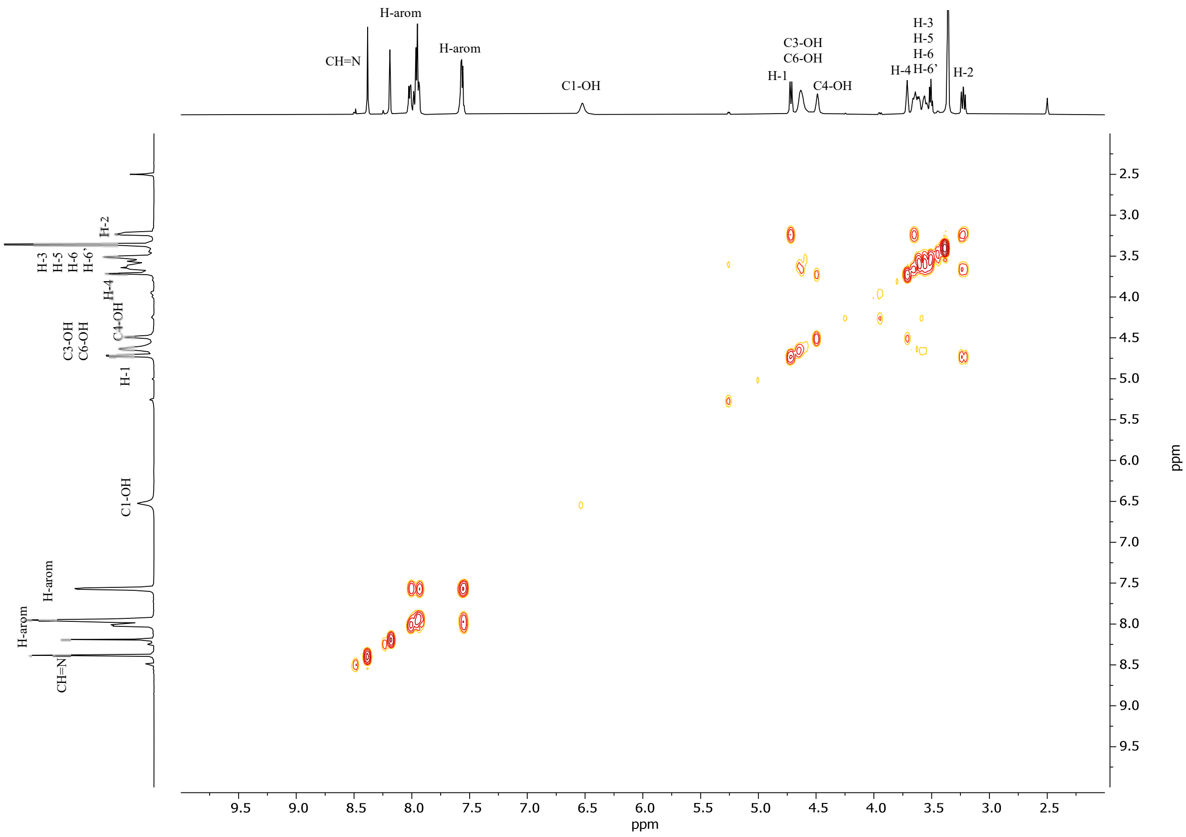

Figure S58. COSY spectrum of **34** (500 MHz, DMSO-d_6).

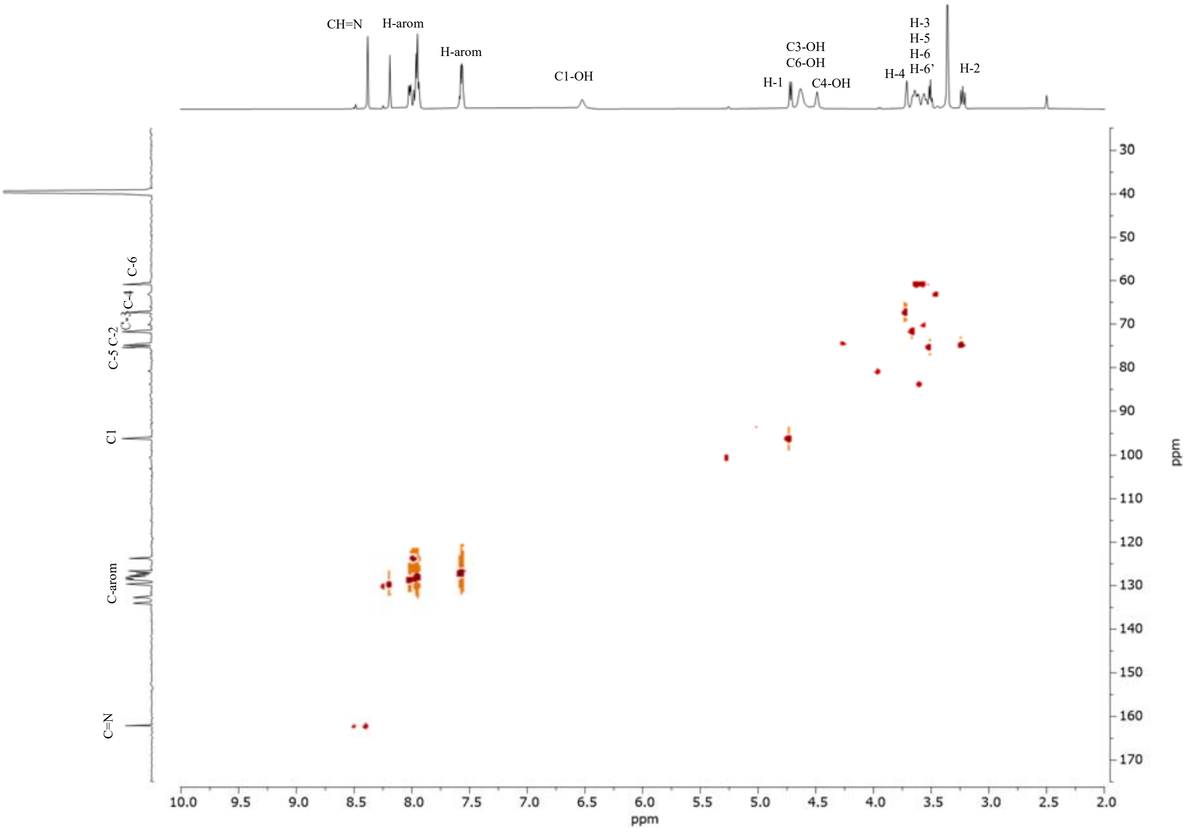

Figure S59. HSQC spectrum of **34** (500 MHz, DMSO-d_6).

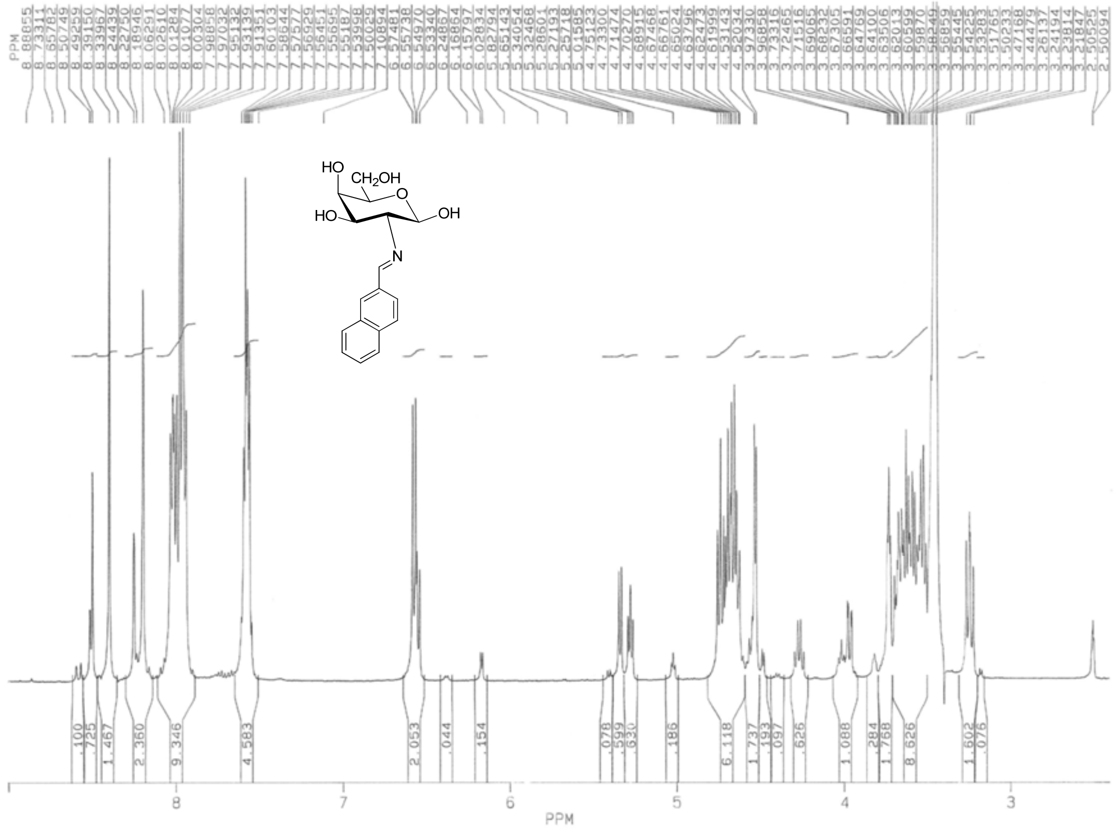

Figure S60. ^{1}H NMR spectrum of **34** in equilibrium (400 MHz, DMSO-d_6).

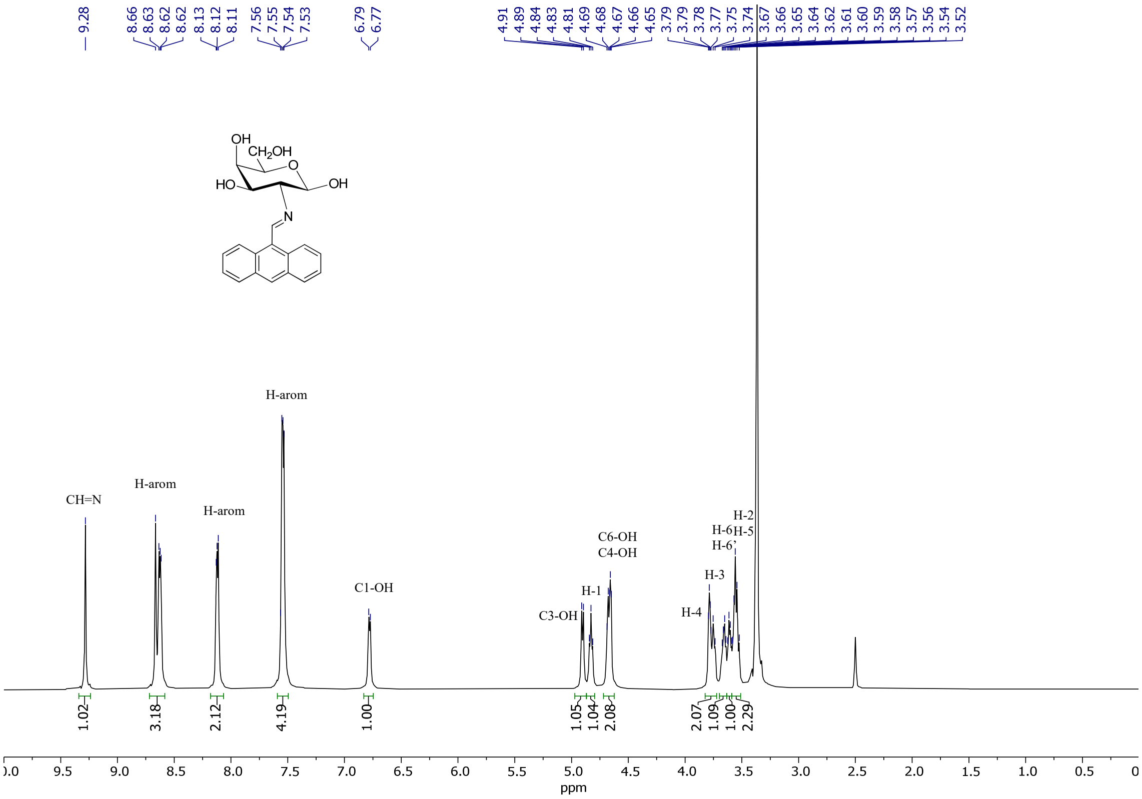

Figure S61. ^{1}H NMR spectrum of **35** (500 MHz, DMSO-d_6).

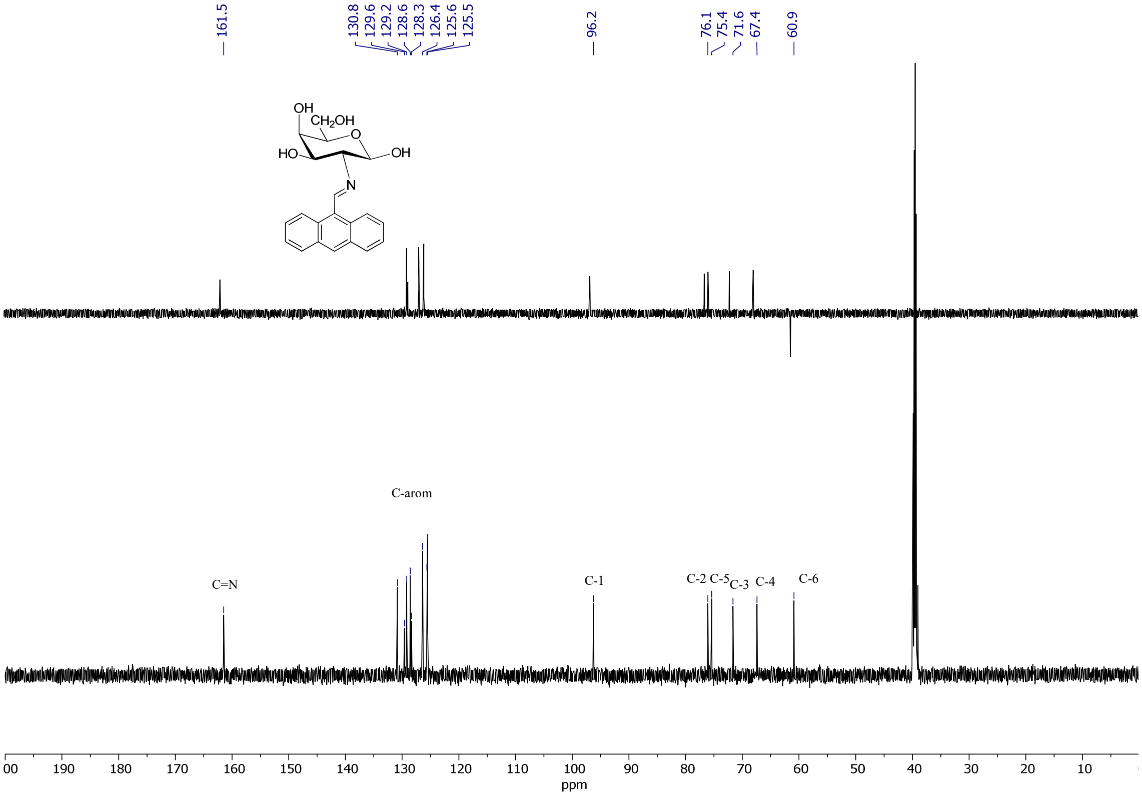

Figure S62. $^{13}C\{^{1}H\}$ NMR and DEPT spectra of **35** (100 MHz, DMSO-d_6).

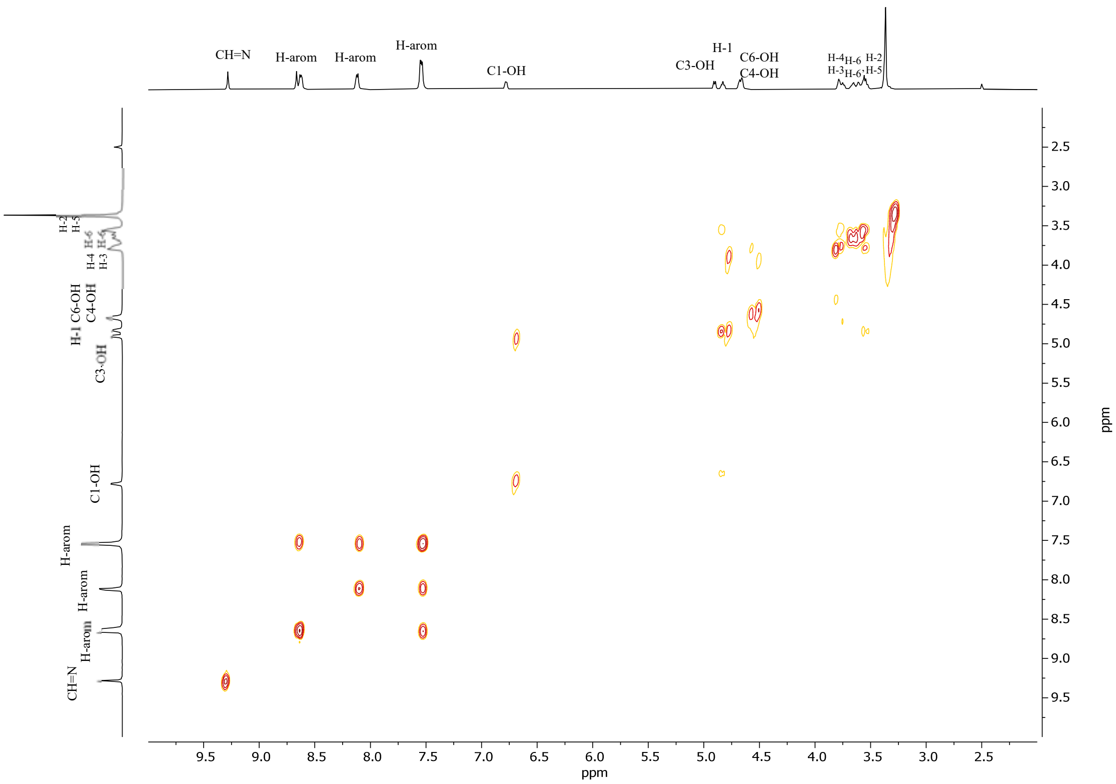

Figure S63. COSY spectrum of **35** in equilibrium (500 MHz, DMSO-d_6).

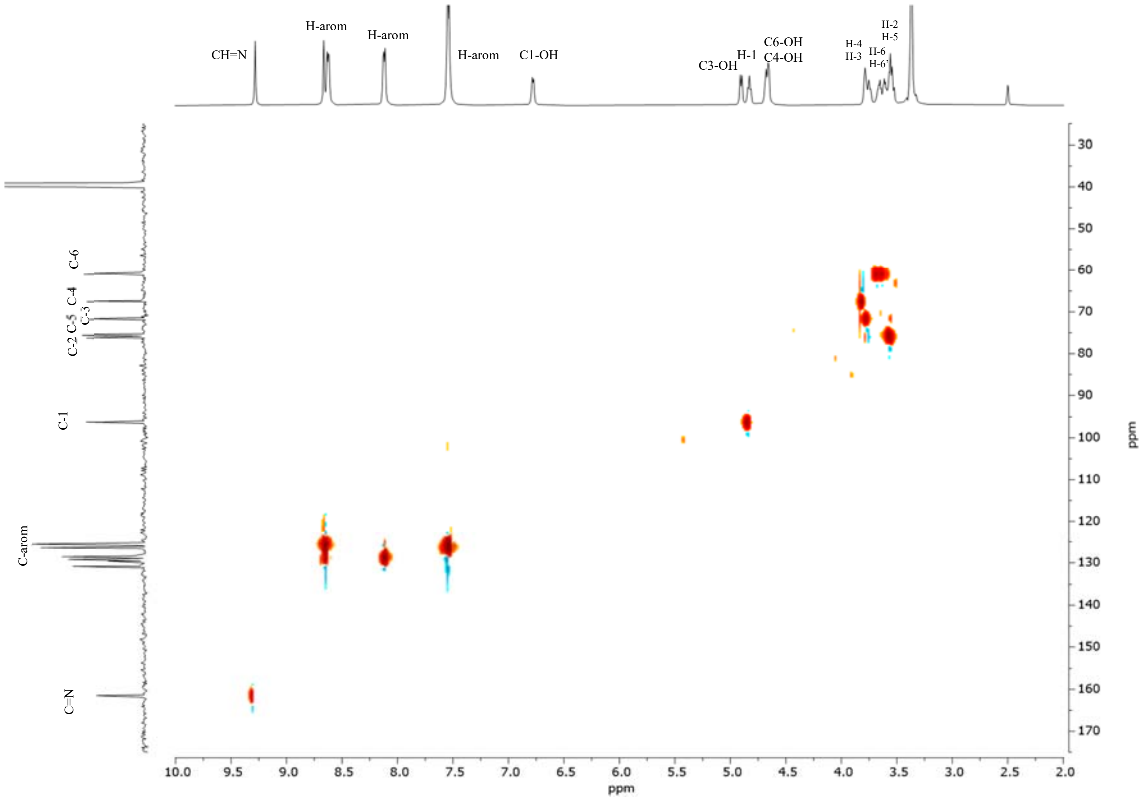

Figure S64. HSQC spectrum of **35** in equilibrium (500 MHz, DMSO-d_6).

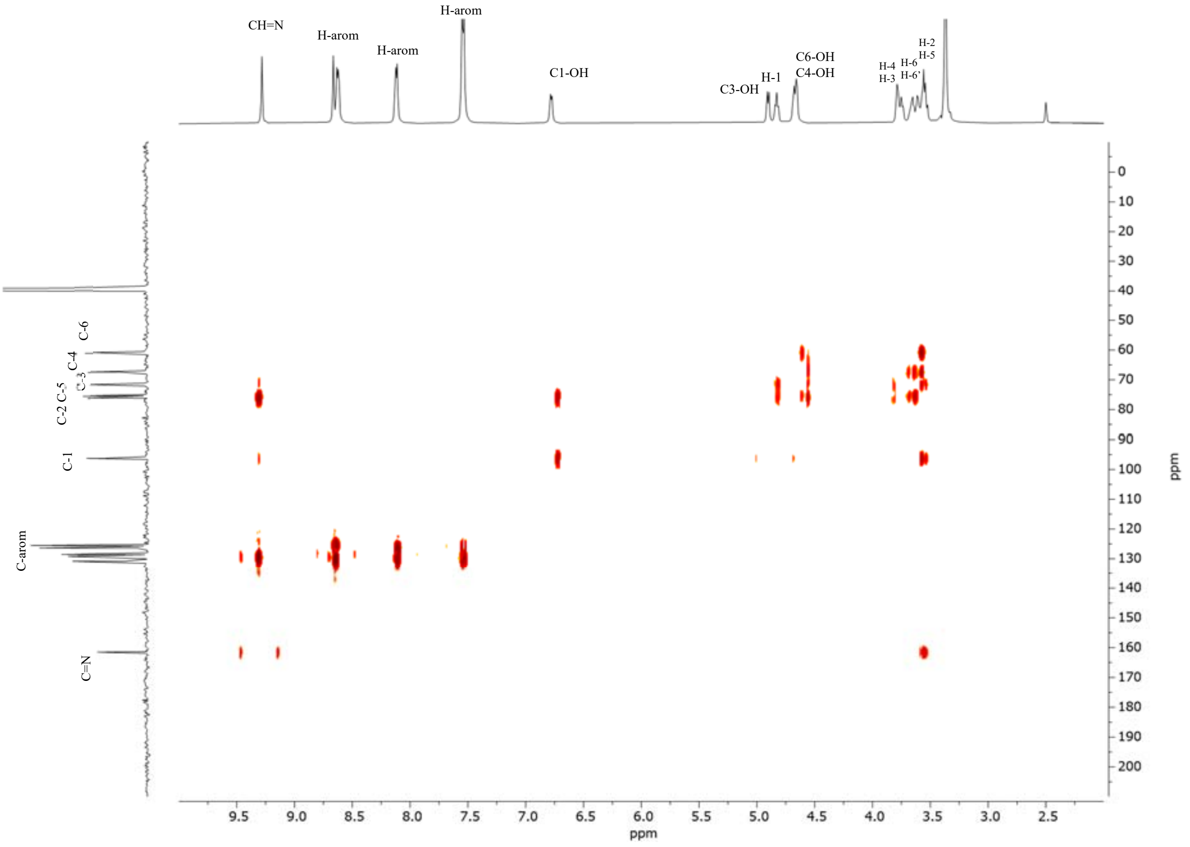

Figure S65. HMBC spectrum of **35** in equilibrium (500 MHz, DMSO-d_6).

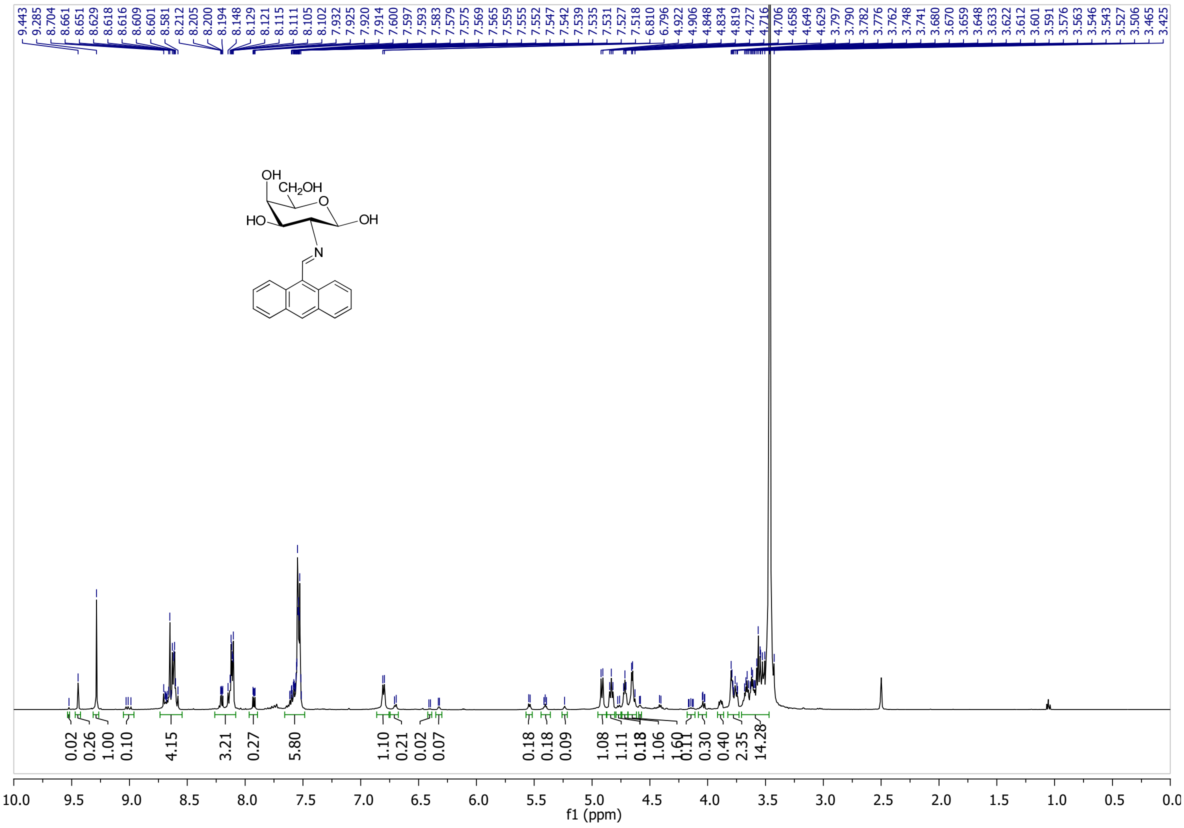

Figure S66. ^{1}H NMR spectrum of **35** in equilibrium (500 MHz, DMSO-d_6).

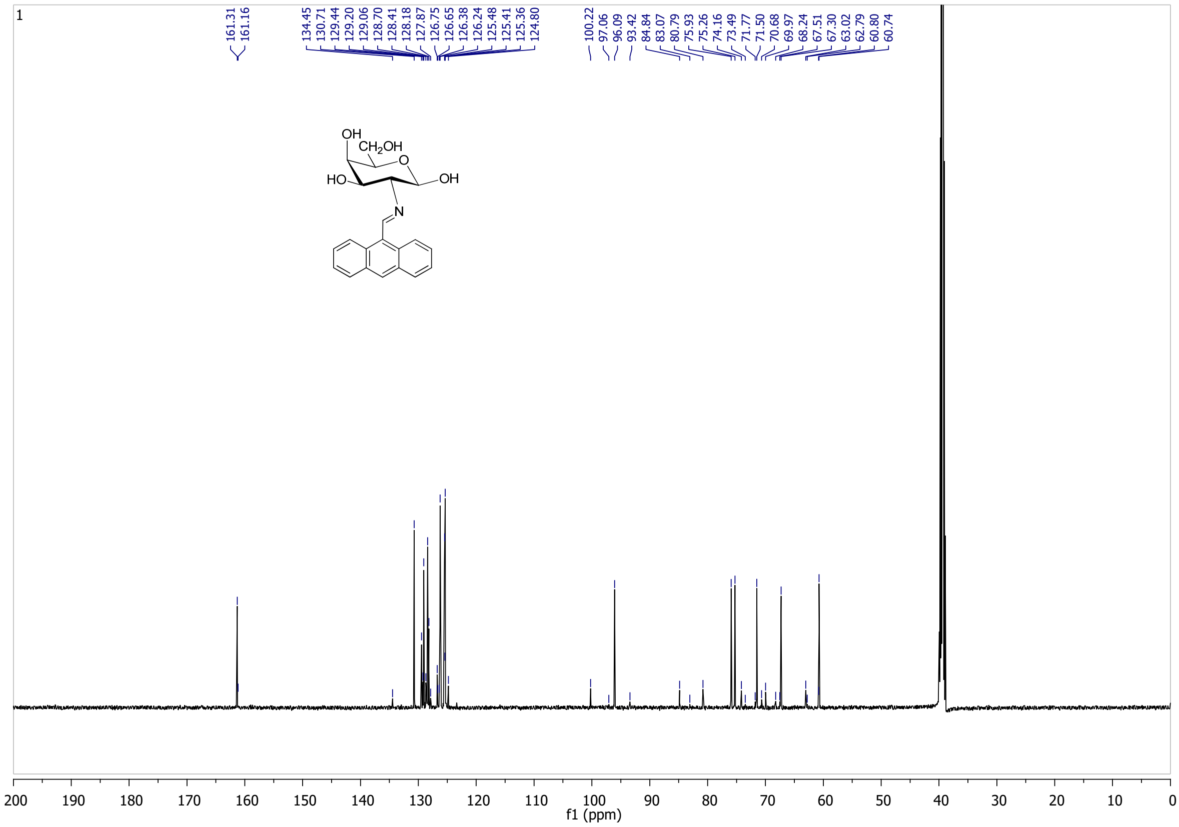

Figure S67. $^{13}C\{^1H\}$ NMR spectrum of **35** in equilibrium (125 MHz, DMSO-d_6).

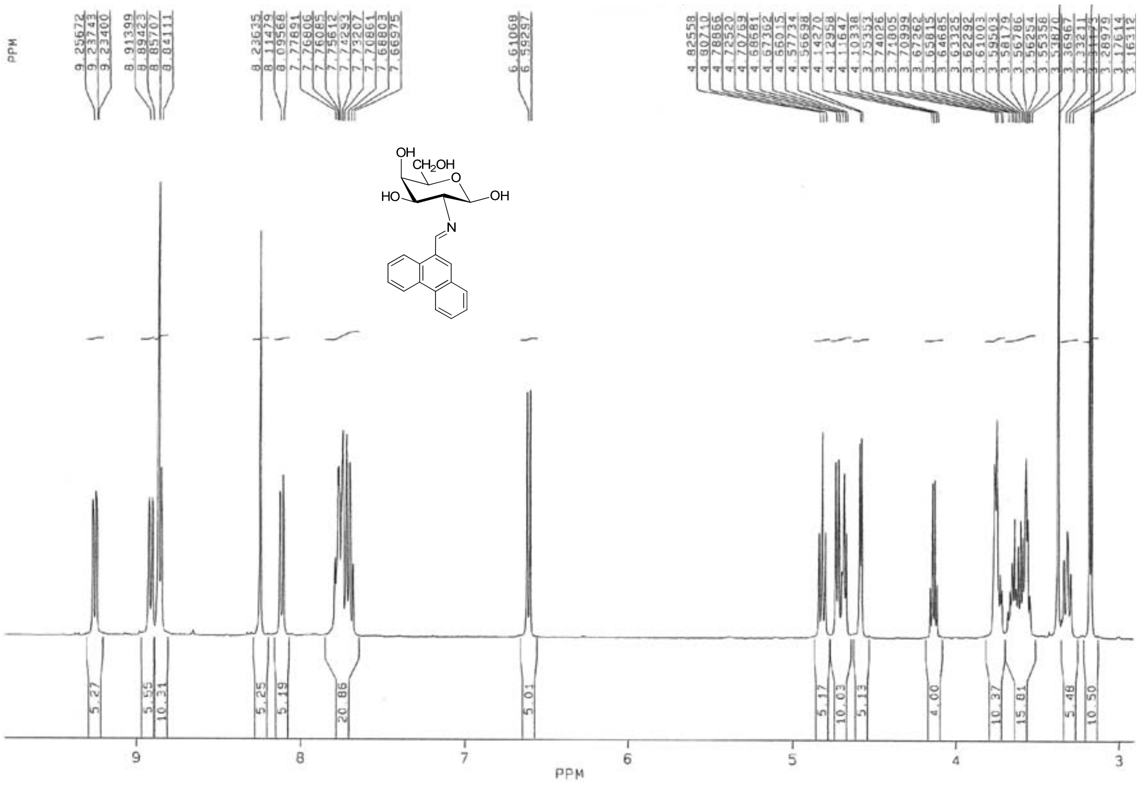

Figure S68. ^1H NMR spectrum of **36** (400 MHz, DMSO-d_6).

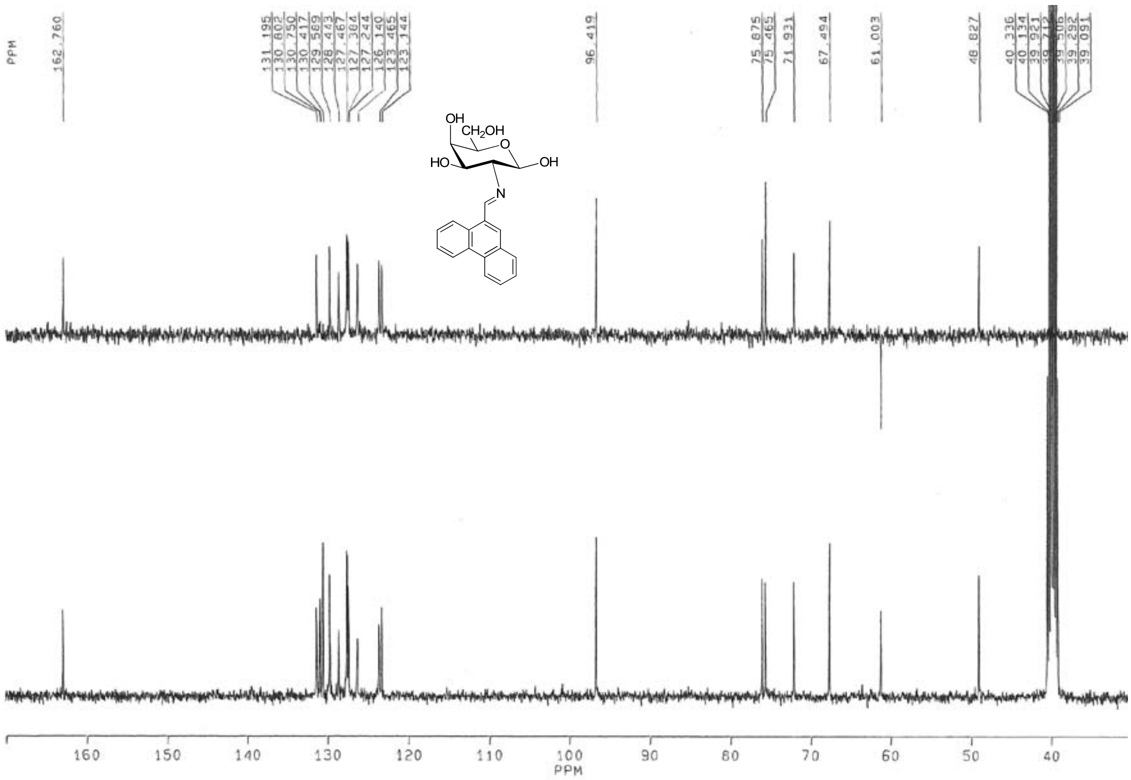

Figure S69. $^{13}C\{^1H\}$ NMR and DEPT spectra of **36** (100 MHz, DMSO-d_6).

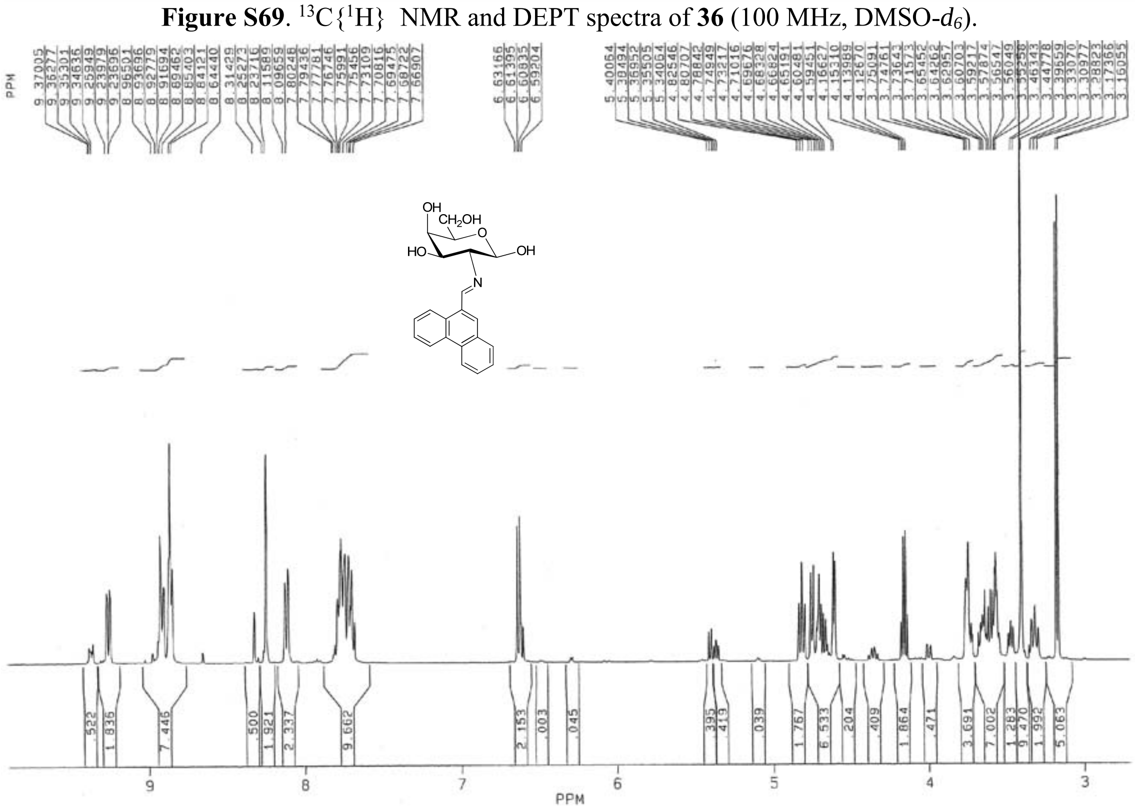

Figure S70. $^{13}C\{^1H\}$ NMR spectrum of **36** in equilibrium (400 MHz, DMSO-d_6).

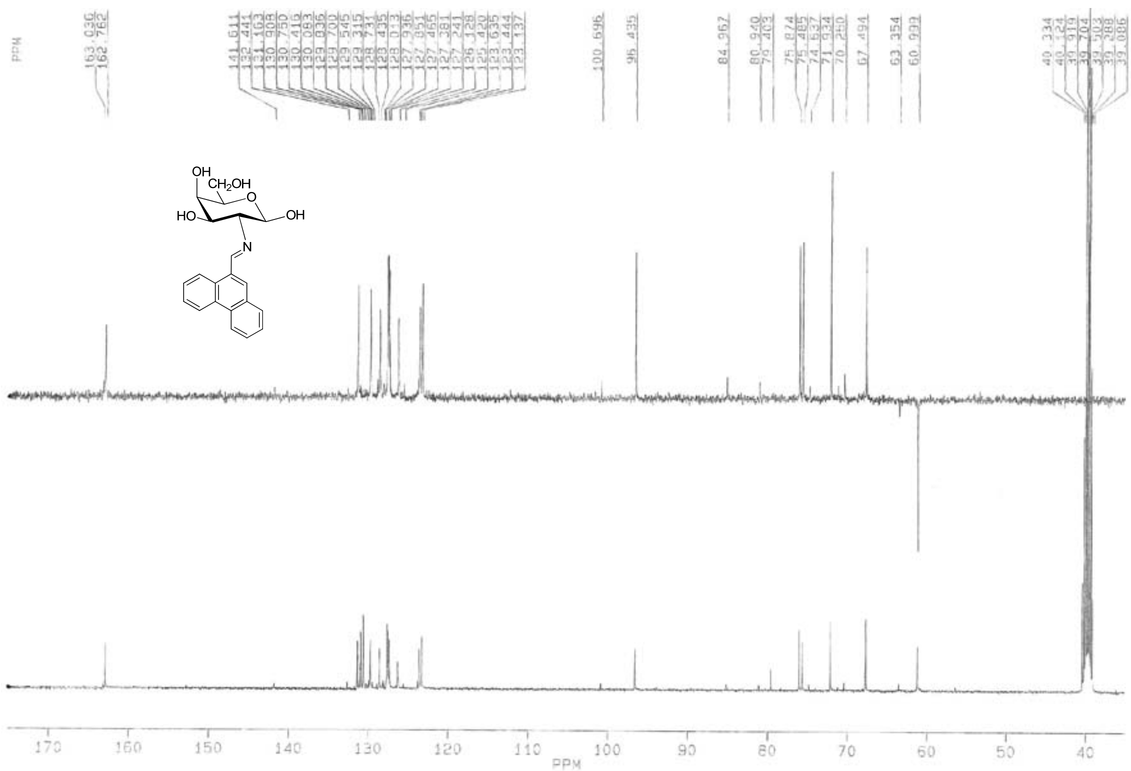

Figure S71. $^{13}C\{^{1}H\}$ NMR and DEPT spectra of **36** in equilibrium (100 MHz, DMSO-d_6).

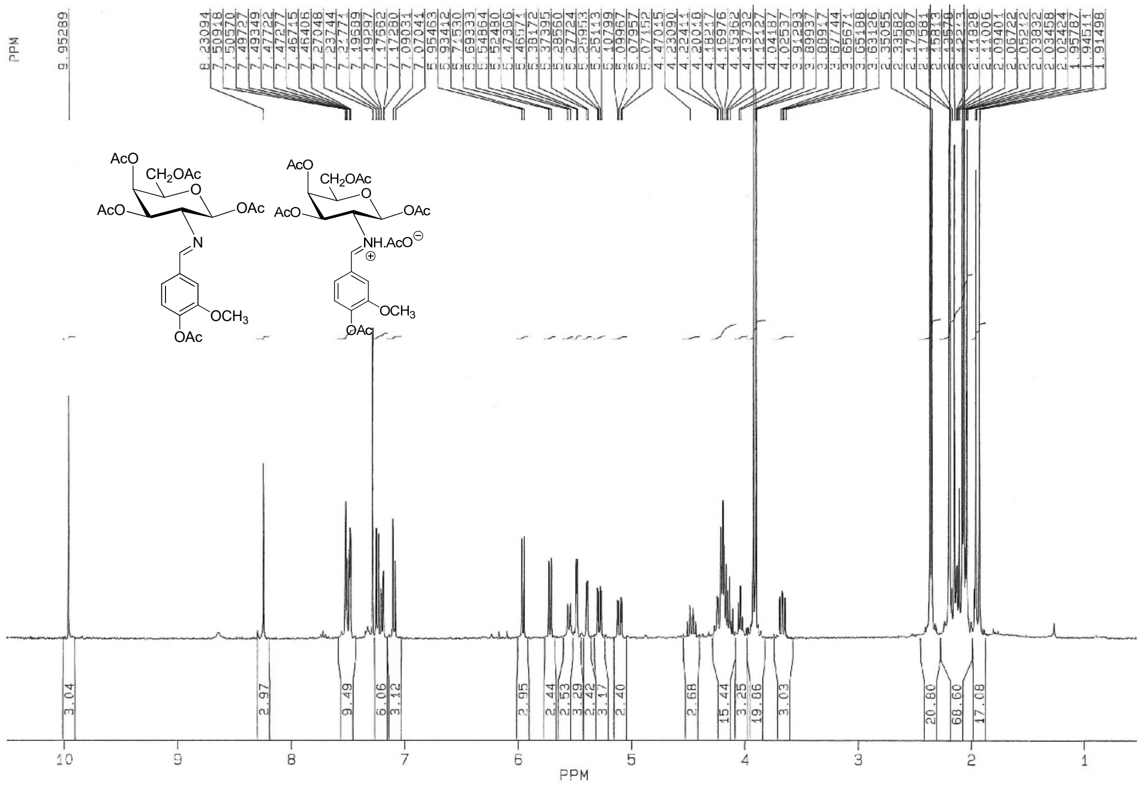

Figure S72. ^{1}H NMR spectrum of **37** and **41** (400 MHz, DMSO-d_6).

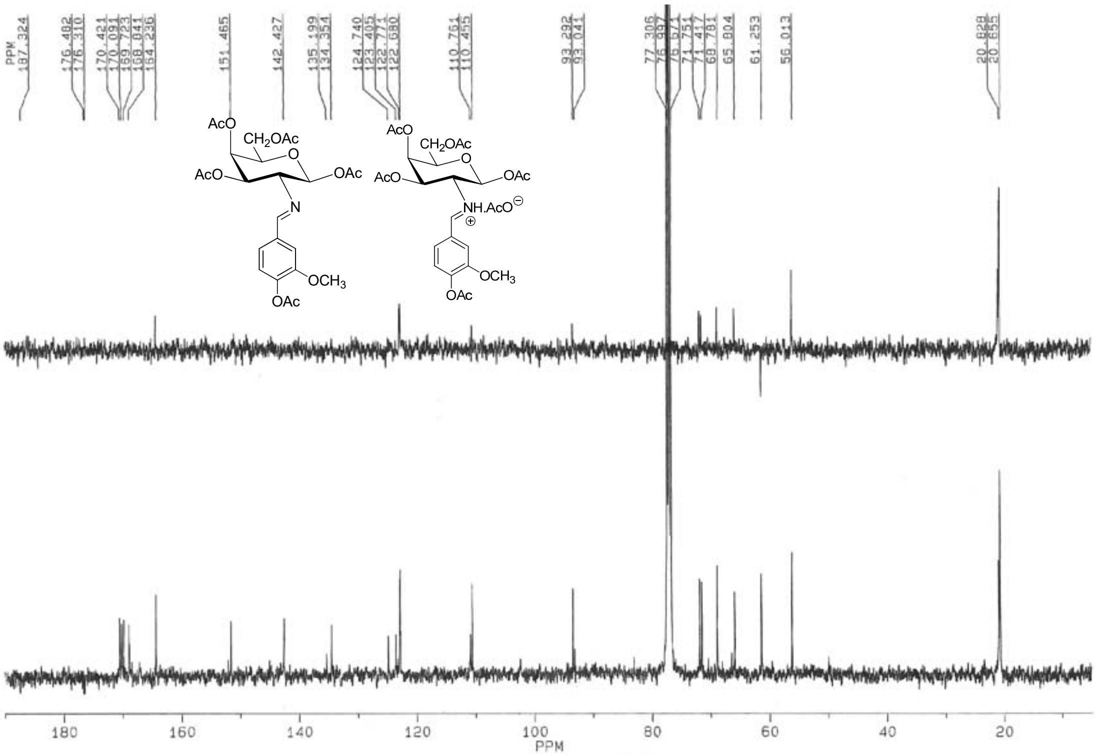

Figure S73. $^{13}C\{^{1}H\}$ NMR and DEPT spectra of **37** and **41** (100 MHz, DMSO-d_6).

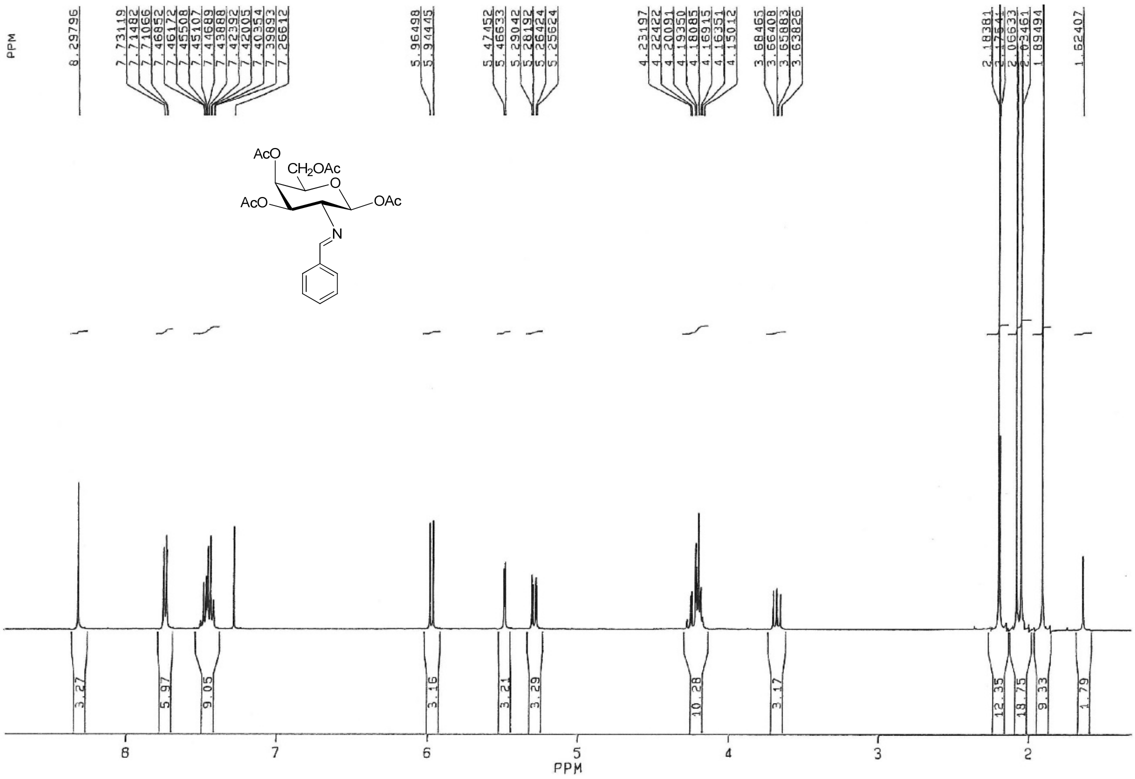

Figure S74. ^{1}H NMR spectrum of **38** (400 MHz, DMSO-d_6).

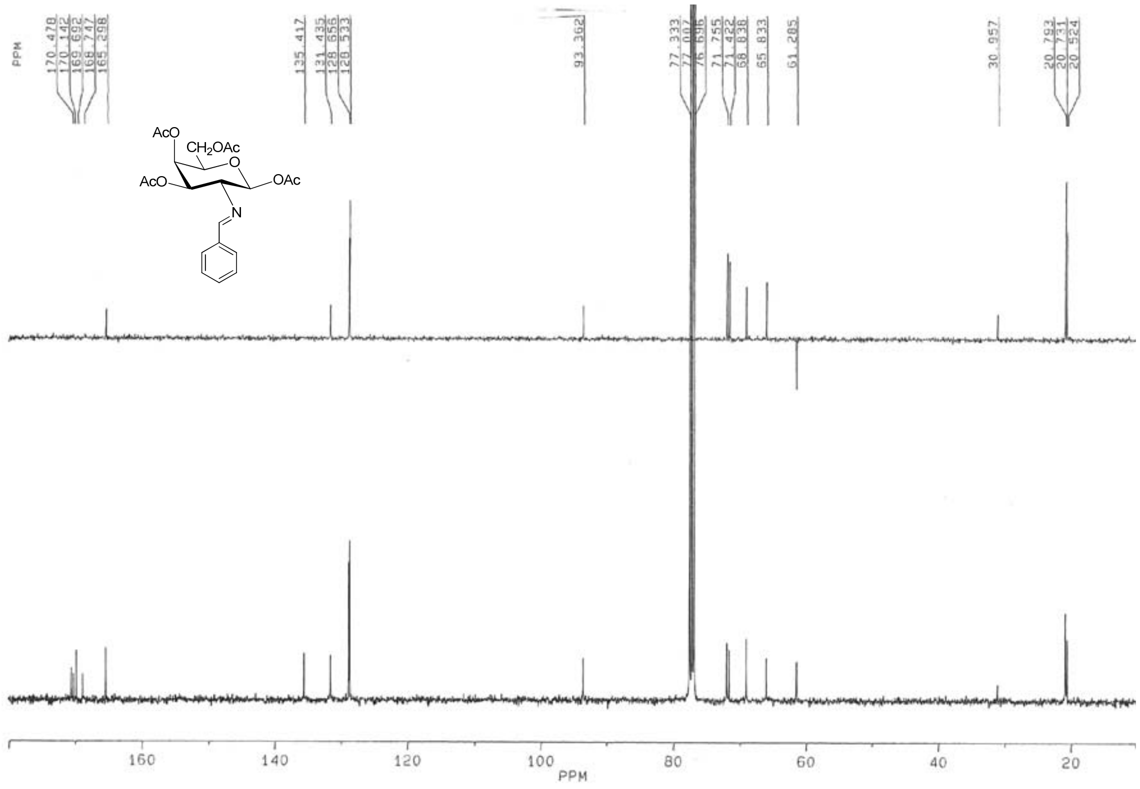

Figure S75. $^{13}C\{^{1}H\}$ NMR and DEPT spectra of **38** (100 MHz, DMSO-d_6).

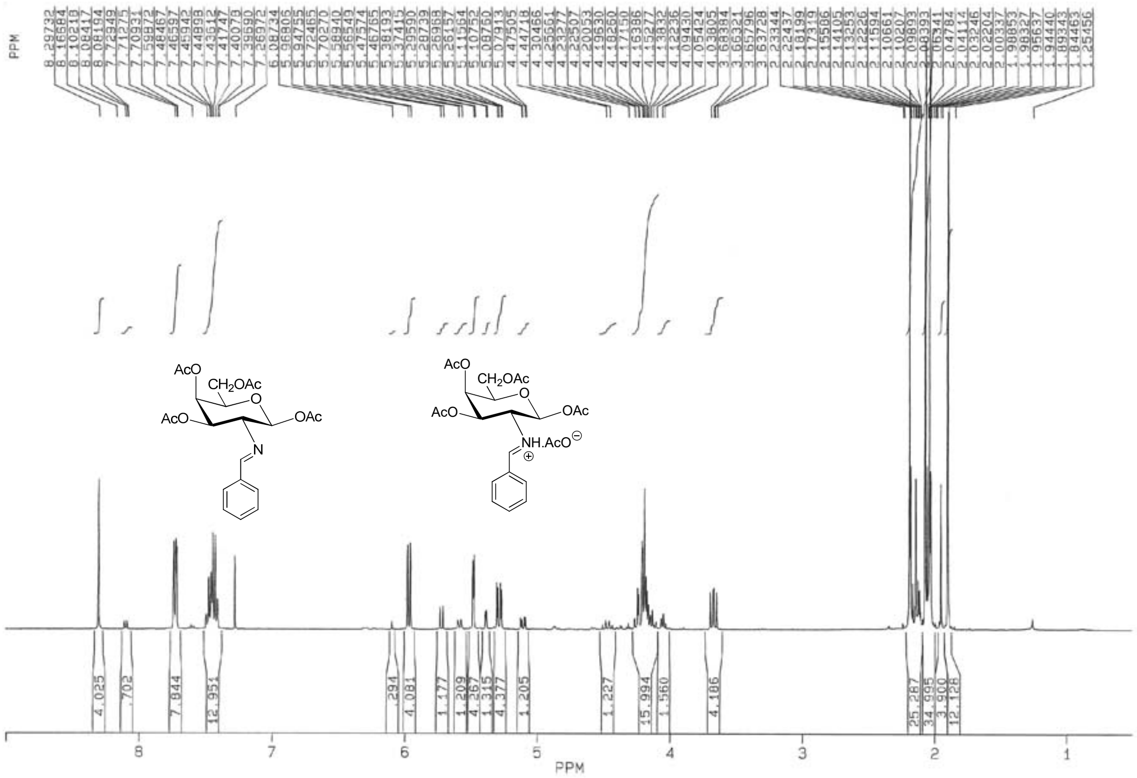

Figure S76. ^{1}H NMR spectrum of **38** and **40** (400 MHz, DMSO-d_6).

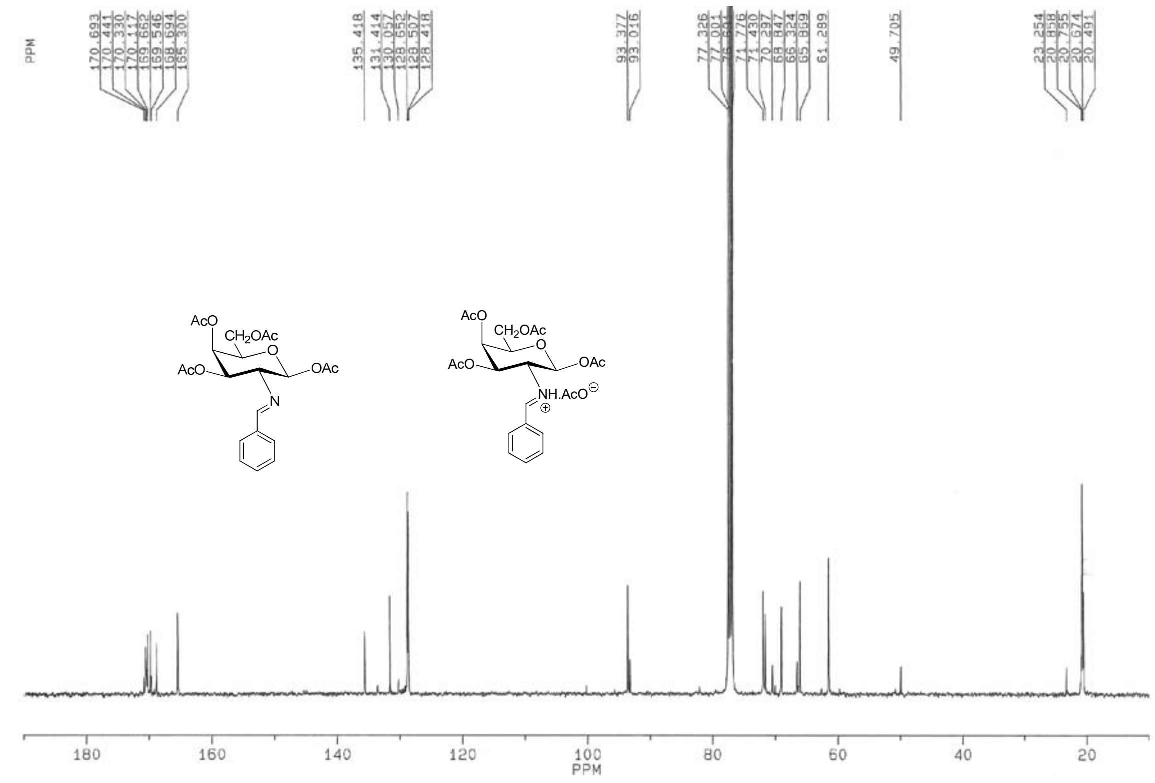

Figure S77. $^{13}C\{^1H\}$ NMR and DEPT spectra of **38** and **40** (100 MHz, DMSO-d_6).

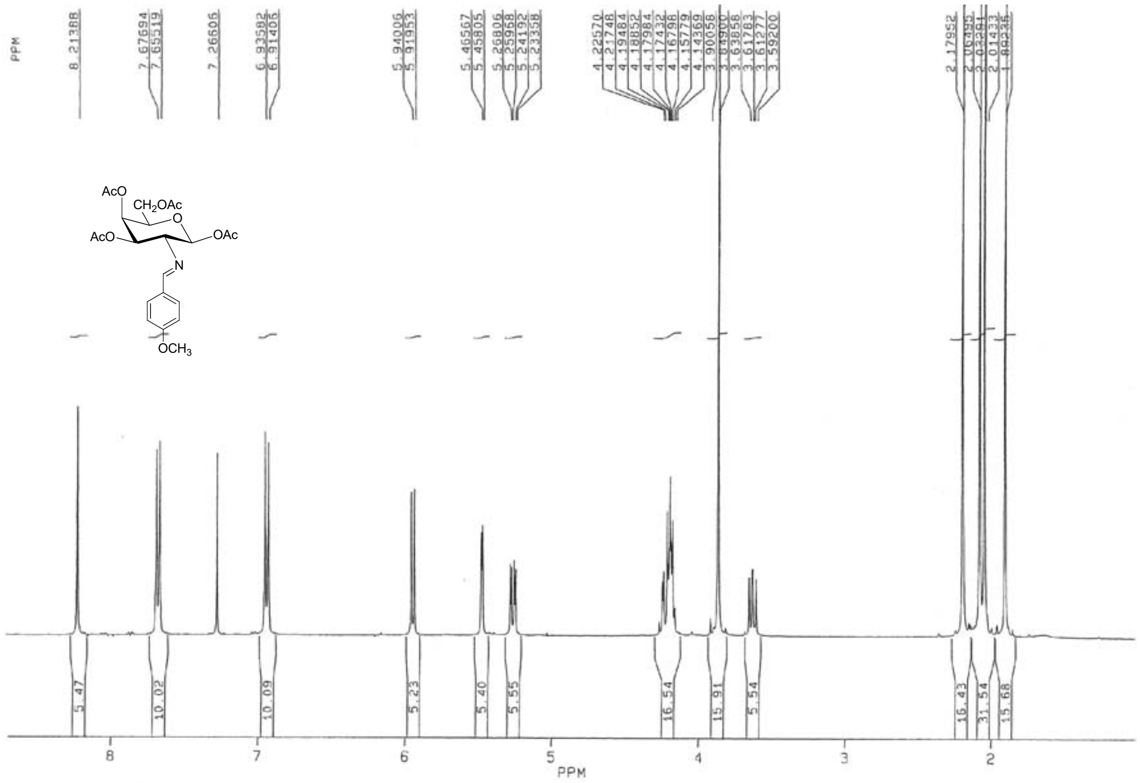

Figure S78. ^1H NMR spectrum of **39** (400 MHz, DMSO-d_6).

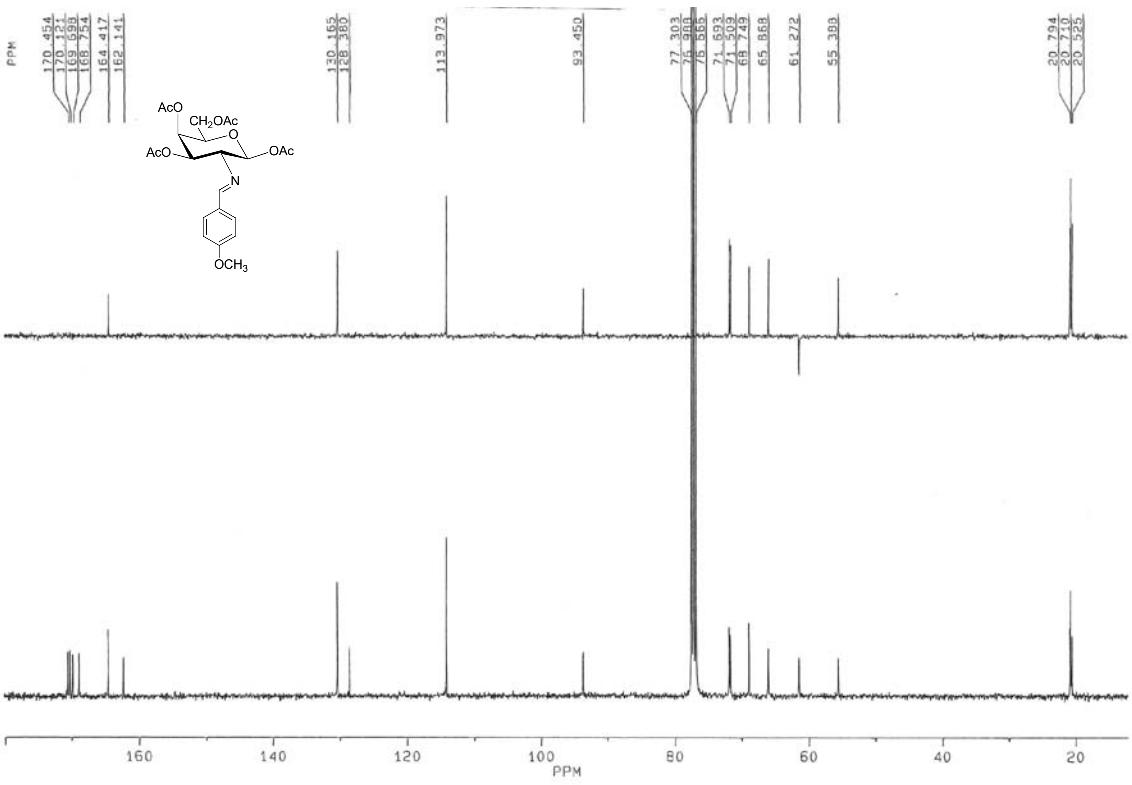

Figure S79. $^{13}C\{^{1}H\}$ NMR and DEPT spectra of **39** (100 MHz, DMSO-d_6).

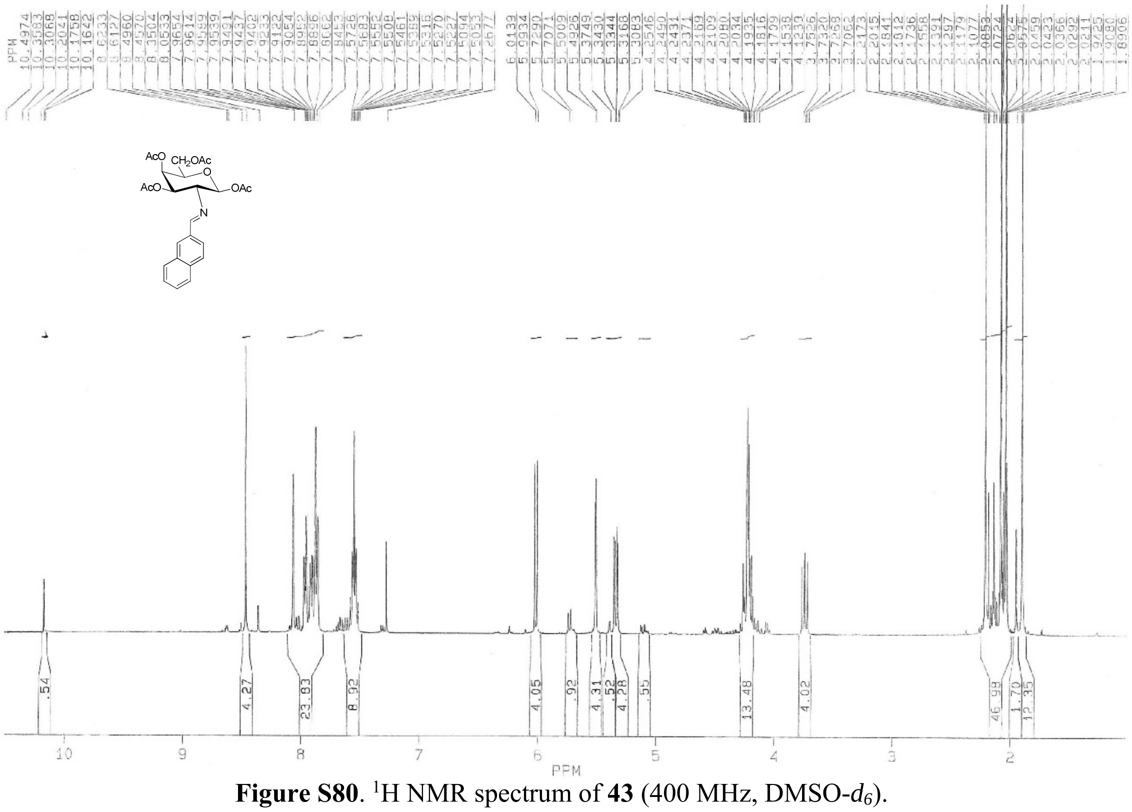

Figure S80. ^{1}H NMR spectrum of **43** (400 MHz, DMSO-d_6).

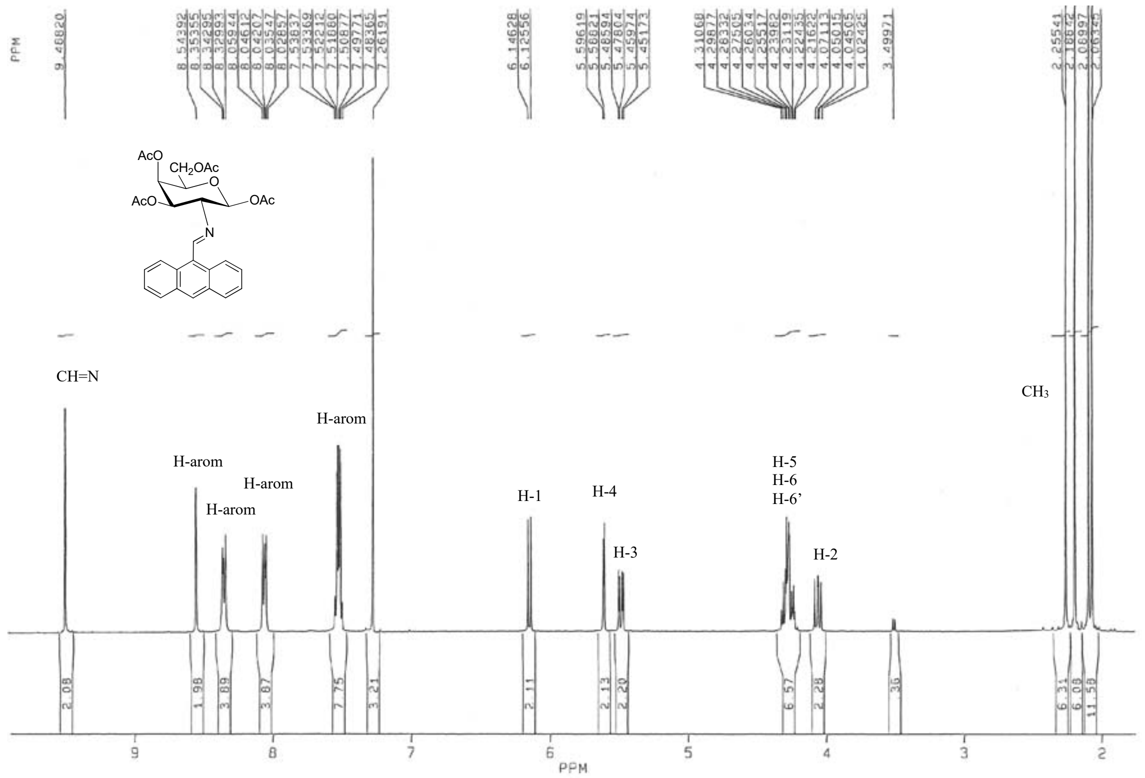

Figure S81. ^{1}H NMR spectrum of **44** (400 MHz, DMSO-d_6).

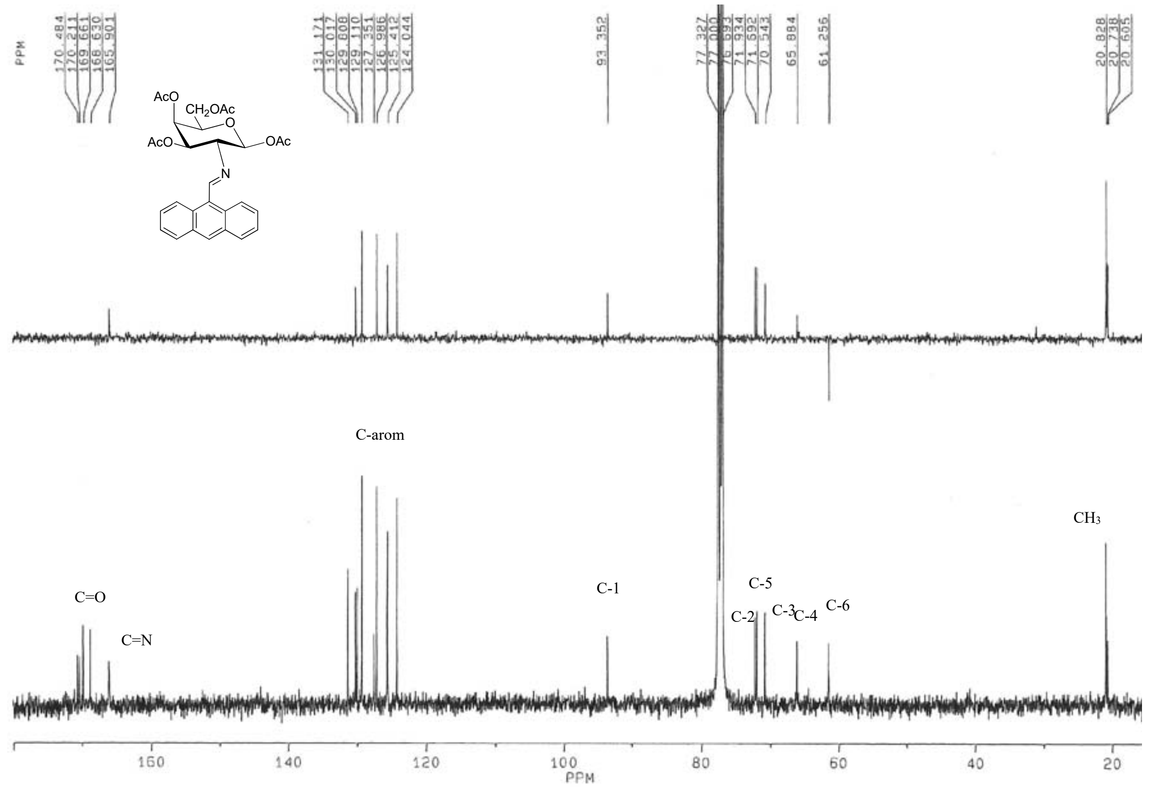

Figure S82. $^{13}C\{^{1}H\}$ NMR and DEPT spectra of **44** (100 MHz, DMSO-d_6).

Structure 29a (B3LYP, Gas Phase)

Energy (Hartrees): = -936.4752324
No imaginary frequencies

Standard orientation:

Center Number	Atomic Number	Atomic Type	Coordinates (Angstroms) X	Y	Z
1	6	0	1.103648	1.368831	0.474107
2	6	0	0.504381	0.062163	-0.072354
3	6	0	1.319236	-1.109178	0.495564
4	6	0	2.810587	-0.966087	0.193000
5	6	0	3.311412	0.435984	0.564123
6	1	0	1.050626	1.377754	1.579706
7	1	0	1.177767	-1.119702	1.587666
8	1	0	3.364126	-1.691172	0.807192
9	1	0	3.352462	0.483919	1.665439
10	1	0	0.634708	0.102184	-1.164108
11	8	0	2.458797	1.469504	0.067659
12	6	0	4.699619	0.720326	-0.039824
13	1	0	4.539088	1.050813	-1.073347
14	1	0	5.180369	1.536791	0.506307
15	8	0	5.561858	-0.400963	0.032961
16	1	0	5.220498	-1.029237	-0.621626
17	8	0	3.068338	-1.204727	-1.189086
18	1	0	2.507060	-1.959269	-1.427941
19	8	0	0.906736	-2.351010	-0.067001
20	1	0	-0.059279	-2.378037	-0.006699
21	8	0	0.406513	2.437236	-0.081454
22	1	0	0.856836	3.248226	0.192825
23	7	0	-0.880670	-0.106218	0.327854
24	6	0	-3.224205	0.041150	-0.243368
25	6	0	-3.688512	-0.345190	1.025553
26	6	0	-4.152881	0.316942	-1.257388
27	6	0	-5.053263	-0.453869	1.267270
28	1	0	-2.960676	-0.551655	1.803008
29	6	0	-5.521320	0.207791	-1.013351
30	1	0	-3.797162	0.619027	-2.239346
31	6	0	-5.973150	-0.178413	0.249006
32	1	0	-5.406411	-0.752016	2.250134
33	1	0	-6.232428	0.423688	-1.805040
34	1	0	-7.038544	-0.263818	0.442082
35	6	0	-1.786940	0.158204	-0.533250
36	1	0	-1.533953	0.493781	-1.550391

Structure 29a (B3LYP, DMSO)

Energy (Hartrees): = -936.4982273
No imaginary frequencies

Standard orientation:

Center Number	Atomic Number	Atomic Type	Coordinates (Angstroms) X	Y	Z
1	6	0	-1.169229	1.479662	-0.249352
2	6	0	-0.501574	0.141833	0.108535
3	6	0	-1.263036	-0.979898	-0.621437
4	6	0	-2.762034	-0.945273	-0.320505
5	6	0	-3.325001	0.469386	-0.496793
6	1	0	-1.128291	1.641719	-1.340889
7	1	0	-1.122531	-0.834592	-1.702522
8	1	0	-3.270030	-1.602162	-1.040911
9	1	0	-3.348126	0.677000	-1.578358
10	1	0	-0.608428	0.005756	1.195233
11	8	0	-2.529571	1.454462	0.169182
12	6	0	-4.733801	0.603245	0.105258
13	1	0	-4.620831	0.778209	1.182602
14	1	0	-5.238343	1.473683	-0.324115
15	8	0	-5.553058	-0.528792	-0.156743
16	1	0	-5.174104	-1.245011	0.377089
17	8	0	-3.024913	-1.391443	1.011758
18	1	0	-2.438605	-2.153732	1.146380
19	8	0	-0.807250	-2.273067	-0.228605
20	1	0	0.161625	-2.265106	-0.265132
21	8	0	-0.522957	2.499053	0.442478
22	1	0	-0.905031	3.338904	0.144096
23	7	0	0.882743	0.122183	-0.327904
24	6	0	3.226840	-0.040779	0.244365
25	6	0	3.694027	0.056846	-1.078374
26	6	0	4.153705	-0.176102	1.289652
27	6	0	5.059248	0.020234	-1.343793
28	1	0	2.974921	0.160168	-1.884679
29	6	0	5.522441	-0.212213	1.021597

30	1	0	3.795756	-0.251980	2.313297
31	6	0	5.977154	-0.114013	-0.294813
32	1	0	5.413364	0.096136	-2.367867
33	1	0	6.231633	-0.316536	1.837498
34	1	0	7.042353	-0.141694	-0.505800
35	6	0	1.790485	-0.004315	0.564428
36	1	0	1.542110	-0.089407	1.632240

Structure 29a (M06-2X/6-311G(d,p), Gas Phase)

Energy (Hartrees): = -936.3471293
No imaginary frequencies

Standard orientation:

Center Number	Atomic Number	Atomic Type	Coordinates (Angstroms) X	Y	Z
1	6	0	-1.043906	1.274654	-0.539499
2	6	0	-0.510624	-0.002338	0.106294
3	6	0	-1.349206	-1.174818	-0.387960
4	6	0	-2.828409	-0.952632	-0.118407
5	6	0	-3.262886	0.425161	-0.611431
6	1	0	-0.969849	1.206848	-1.638824
7	1	0	-1.193584	-1.269648	-1.471732
8	1	0	-3.406154	-1.708149	-0.664404
9	1	0	-3.278225	0.395621	-1.711548
10	1	0	-0.650951	0.123280	1.187959
11	8	0	-2.390534	1.453833	-0.170586
12	6	0	-4.640840	0.791373	-0.053906
13	1	0	-4.481165	1.160062	0.963282
14	1	0	-5.080274	1.592425	-0.649022
15	8	0	-5.531159	-0.300870	-0.084522
16	1	0	-5.255656	-0.887779	0.627786
17	8	0	-3.101814	-1.043164	1.269248
18	1	0	-2.591793	-1.793993	1.595228
19	8	0	-0.985541	-2.373138	0.269841
20	1	0	-0.027829	-2.453557	0.204318
21	8	0	-0.314157	2.343525	-0.044818
22	1	0	-0.715681	3.150826	-0.379073
23	7	0	0.871899	-0.250578	-0.255060
24	6	0	3.204752	0.078504	0.226159
25	6	0	3.651865	-0.588131	-0.917966
26	6	0	4.133259	0.602523	1.124854
27	6	0	5.010240	-0.727669	-1.152643
28	1	0	2.915921	-0.983022	-1.607889
29	6	0	5.496168	0.461754	0.888760
30	1	0	3.784344	1.122897	2.010859
31	6	0	5.934864	-0.203368	-0.249546
32	1	0	5.355101	-1.243094	-2.041066
33	1	0	6.213028	0.870471	1.590691
34	1	0	6.996341	-0.313925	-0.437034
35	6	0	1.765218	0.237064	0.500793
36	1	0	1.505812	0.810806	1.399564

Structure 29a (M06-2X/6-311G(d,p), DMSO)

Energy (Hartrees): = -936.3726836
No imaginary frequencies

Standard orientation:

Center Number	Atomic Number	Atomic Type	Coordinates (Angstroms) X	Y	Z
1	6	0	-1.163269	1.465922	-0.245497
2	6	0	-0.507704	0.136136	0.115834
3	6	0	-1.257158	-0.976848	-0.617432
4	6	0	-2.745663	-0.939244	-0.310448
5	6	0	-3.300741	0.468904	-0.500180
6	1	0	-1.118906	1.625529	-1.334732
7	1	0	-1.118903	-0.828727	-1.696076
8	1	0	-3.258335	-1.608656	-1.011424
9	1	0	-3.317240	0.677332	-1.579291
10	1	0	-0.613133	-0.000023	1.200820
11	8	0	-2.514088	1.445710	0.167808
12	6	0	-4.699801	0.592989	0.102522
13	1	0	-4.578899	0.733222	1.180875
14	1	0	-5.200064	1.472214	-0.305625
15	8	0	-5.510921	-0.529416	-0.184780
16	1	0	-5.162734	-1.252276	0.349928
17	8	0	-2.997990	-1.350241	1.025810
18	1	0	-2.432023	-2.116225	1.182692
19	8	0	-0.797692	-2.258391	-0.223423
20	1	0	0.161107	-2.277183	-0.326632

21	8	0	-0.513330	2.474768	0.444067
22	1	0	-0.878071	3.316369	0.146378
23	7	0	0.873262	0.120430	-0.318247
24	6	0	3.207394	-0.041336	0.247725
25	6	0	3.659035	0.060598	-1.072165
26	6	0	4.132193	-0.178858	1.284018
27	6	0	5.018243	0.025439	-1.346058
28	1	0	2.934063	0.166874	-1.870662
29	6	0	5.495745	-0.213864	1.007872
30	1	0	3.778305	-0.257616	2.307053
31	6	0	5.938987	-0.111530	-0.306389
32	1	0	5.366201	0.104871	-2.369479
33	1	0	6.209683	-0.320646	1.816232
34	1	0	7.000693	-0.137955	-0.523767
35	6	0	1.768103	-0.006022	0.571764
36	1	0	1.515403	-0.093268	1.636180

Structure 29b (B3LYP, Gas Phase)

Energy (Hartrees): = -936.4750172
No imaginary frequencies

Standard orientation:

Center Number	Atomic Number	Atomic Type	Coordinates (Angstroms) X	Y	Z
1	6	0	-1.170994	1.218516	-0.609686
2	6	0	-0.538307	-0.040295	0.007533
3	6	0	-1.296629	-1.268929	-0.516565
4	6	0	-2.799308	-1.151806	-0.254593
5	6	0	-3.325803	0.202098	-0.757283
6	1	0	-1.125732	-1.339581	-1.601897
7	1	0	-3.317361	-1.949578	-0.810789
8	1	0	-3.262316	0.170701	-1.861057
9	1	0	-0.693559	0.046230	1.093129
10	8	0	-2.543427	1.284445	-0.260187
11	6	0	-4.790345	0.466566	-0.387345
12	1	0	-5.111087	1.385952	-0.888808
13	1	0	-5.387055	-0.362313	-0.809858
14	8	0	-5.023699	0.649240	0.988305
15	1	0	-4.536602	-0.051136	1.450191
16	8	0	-3.059053	-1.285441	1.140023
17	1	0	-2.480706	-1.999106	1.451642
18	8	0	-0.863617	-2.461441	0.130781
19	1	0	0.104429	-2.456742	0.108941
20	7	0	0.860718	-0.173143	-0.355641
21	6	0	3.182808	0.093388	0.259588
22	6	0	3.692063	-0.315851	-0.984686
23	6	0	4.075368	0.441541	1.283774
24	6	0	5.065418	-0.375857	-1.192193
25	1	0	2.991789	-0.577578	-1.770760
26	6	0	5.452449	0.380799	1.074107
27	1	0	3.684613	0.762271	2.246306
28	6	0	5.949100	-0.028547	-0.163891
29	1	0	5.453531	-0.691527	-2.156218
30	1	0	6.135357	0.652688	1.873322
31	1	0	7.021327	-0.075849	-0.330391
32	6	0	1.735797	0.162394	0.513259
33	1	0	1.446087	0.528717	1.509750
34	1	0	-1.077043	1.186522	-1.712955
35	8	0	-0.536551	2.334282	-0.072928
36	1	0	-1.049625	3.109556	-0.341136

Structure 29b (B3LYP, DMSO)

Energy (Hartrees): = -936.4999106
No imaginary frequencies

Standard orientation:

Center Number	Atomic Number	Atomic Type	Coordinates (Angstroms) X	Y	Z
1	6	0	1.234991	-1.375554	-0.333428
2	6	0	0.535139	-0.055809	0.031226
3	6	0	1.236985	1.090602	-0.721358
4	6	0	2.745524	1.089106	-0.465974
5	6	0	3.331024	-0.308355	-0.711040
6	1	0	1.061919	0.952868	-1.798076
7	1	0	3.221004	1.789585	-1.168445
8	1	0	3.244264	-0.500324	-1.793878
9	1	0	0.665877	0.091535	1.113715
10	8	0	2.611705	-1.305096	0.018312
11	6	0	4.812352	-0.422022	-0.348832

12	1	0	5.168278	-1.416589	-0.639180
13	1	0	5.357162	0.311675	-0.966925
14	8	0	5.097735	-0.265354	1.031045
15	1	0	4.560089	0.487691	1.327054
16	8	0	3.011013	1.495794	0.880482
17	1	0	2.403293	2.230952	1.061902
18	8	0	0.763342	2.367488	-0.296807
19	1	0	-0.205646	2.332840	-0.287152
20	7	0	-0.860641	-0.087227	-0.366714
21	6	0	-3.192237	0.004976	0.269582
22	6	0	-3.692864	-0.128113	-1.037836
23	6	0	-4.093588	0.125147	1.338801
24	6	0	-5.065424	-0.141660	-1.264690
25	1	0	-2.993816	-0.219436	-1.862947
26	6	0	-5.469700	0.111351	1.109358
27	1	0	-3.710142	0.228345	2.350736
28	6	0	-5.957593	-0.022208	-0.191982
29	1	0	-5.445167	-0.245106	-2.277097
30	1	0	-6.158950	0.204415	1.943468
31	1	0	-7.028572	-0.033266	-0.372984
32	6	0	-1.746994	0.020753	0.549326
33	1	0	-1.471617	0.127679	1.608452
34	1	0	1.148536	-1.558086	-1.419670
35	8	0	0.651148	-2.401772	0.403430
36	1	0	1.062730	-3.231948	0.117418

Structure 29b (M06-2X/6-311G(d,p), Gas Phase)

Energy (Hartrees): = -936.3456104
No imaginary frequencies

Standard orientation:

Center Number	Atomic Number	Atomic Type	Coordinates (Angstroms) X	Y	Z
1	6	0	1.108691	-1.093034	-0.687530
2	6	0	0.547958	0.118408	0.054220
3	6	0	1.331543	1.353185	-0.374014
4	6	0	2.822777	1.153827	-0.150145
5	6	0	3.280107	-0.156917	-0.792188
6	1	0	1.144708	1.525454	-1.442930
7	1	0	3.369134	1.981771	-0.621624
8	1	0	3.193011	-0.020887	-1.883537
9	1	0	0.720894	-0.075127	1.120534
10	8	0	2.473546	-1.244763	-0.379716
11	6	0	4.731351	-0.506189	-0.475708
12	1	0	5.025401	-1.354240	-1.097720
13	1	0	5.349092	0.357508	-0.765279
14	8	0	4.945990	-0.885257	0.856389
15	1	0	4.477883	-0.251730	1.411201
16	8	0	3.098181	1.121421	1.239404
17	1	0	2.578433	1.827939	1.640556
18	8	0	0.948982	2.484672	0.383591
19	1	0	-0.013351	2.526811	0.363438
20	7	0	-0.852402	0.340727	-0.253646
21	6	0	-3.156459	-0.148059	0.230704
22	6	0	-3.660009	0.628083	-0.816824
23	6	0	-4.038903	-0.819786	1.075416
24	6	0	-5.028366	0.729227	-1.009122
25	1	0	-2.959102	1.137441	-1.466919
26	6	0	-5.411890	-0.718528	0.881193
27	1	0	-3.646240	-1.426018	1.885181
28	6	0	-5.906834	0.056785	-0.160207
29	1	0	-5.416772	1.331183	-1.821889
30	1	0	-6.092534	-1.243874	1.540006
31	1	0	-6.976328	0.137276	-0.314589
32	6	0	-1.705512	-0.267643	0.460638
33	1	0	-1.400181	-0.922112	1.286641
34	1	0	0.992273	-0.959937	-1.777927
35	8	0	0.437538	-2.219106	-0.238871
36	1	0	0.899875	-2.988432	-0.584601

Structure 29b (M06-2X/6-311G(d,p), DMSO)

Energy (Hartrees): = -936.3732019
No imaginary frequencies

Standard orientation:

Center Number	Atomic Number	Atomic Type	Coordinates (Angstroms) X	Y	Z
1	6	0	1.241524	-1.361057	-0.346640
2	6	0	0.545494	-0.052830	0.017143

3	6	0	1.230002	1.086158	-0.740548
4	6	0	2.726563	1.095257	-0.464202
5	6	0	3.316735	-0.292538	-0.708900
6	1	0	1.068049	0.937028	-1.815408
7	1	0	3.209739	1.803980	-1.147784
8	1	0	3.237378	-0.484334	-1.789754
9	1	0	0.674729	0.098033	1.097519
10	8	0	2.605094	-1.286880	0.012302
11	6	0	4.785878	-0.393444	-0.324861
12	1	0	5.167311	-1.362733	-0.653601
13	1	0	5.327686	0.386990	-0.877314
14	8	0	5.020195	-0.304976	1.064798
15	1	0	4.459725	0.410807	1.387403
16	8	0	2.965285	1.481183	0.884514
17	1	0	2.372651	2.219788	1.072232
18	8	0	0.736874	2.349126	-0.327567
19	1	0	-0.226318	2.325543	-0.375317
20	7	0	-0.847269	-0.095773	-0.375512
21	6	0	-3.166594	0.005710	0.269477
22	6	0	-3.666520	-0.149999	-1.027845
23	6	0	-4.053637	0.143153	1.338211
24	6	0	-5.036168	-0.169193	-1.246294
25	1	0	-2.971008	-0.254871	-1.852278
26	6	0	-5.427310	0.123991	1.117647
27	1	0	-3.662142	0.263496	2.343342
28	6	0	-5.918780	-0.032373	-0.174093
29	1	0	-5.421806	-0.290258	-2.252004
30	1	0	-6.111863	0.230174	1.951011
31	1	0	-6.988424	-0.047791	-0.349071
32	6	0	-1.716231	0.026384	0.540566
33	1	0	-1.429678	0.150744	1.592614
34	1	0	1.161496	-1.541544	-1.431069
35	8	0	0.653429	-2.380744	0.382607
36	1	0	1.050609	-3.211626	0.096856

Structure 29b (M06-2X/def2-TZVP, Gas Phase)

Energy (Hartrees): = -936.4622493
No imaginary frequencies

Standard orientation:

Center Number	Atomic Number	Atomic Type	Coordinates (Angstroms) X	Y	Z
1	6	0	1.119501	-1.125795	-0.653615
2	6	0	0.541926	0.100216	0.048743
3	6	0	1.321781	1.326613	-0.404594
4	6	0	2.812047	1.147477	-0.168134
5	6	0	3.278666	-0.172797	-0.777804
6	1	0	1.147390	1.465726	-1.479345
7	1	0	3.349469	1.963432	-0.667211
8	1	0	3.186619	-0.061120	-1.870397
9	1	0	0.697165	-0.061417	1.122733
10	8	0	2.483579	-1.256369	-0.342268
11	6	0	4.735038	-0.497899	-0.473725
12	1	0	5.027995	-1.362306	-1.071567
13	1	0	5.339725	0.359510	-0.800650
14	8	0	4.985418	-0.829250	0.864556
15	1	0	4.513706	-0.197458	1.420666
16	8	0	3.094854	1.164684	1.218068
17	1	0	2.572706	1.877158	1.607591
18	8	0	0.922827	2.479309	0.308911
19	1	0	-0.039816	2.529773	0.274418
20	7	0	-0.851756	0.290777	-0.289038
21	6	0	-3.162616	-0.114325	0.242214
22	6	0	-3.666577	0.517332	-0.894686
23	6	0	-4.042907	-0.673767	1.163633
24	6	0	-5.032544	0.587235	-1.099222
25	1	0	-2.969344	0.941213	-1.605623
26	6	0	-5.413423	-0.604173	0.957572
27	1	0	-3.648558	-1.166992	2.044691
28	6	0	-5.908368	0.026724	-0.173520
29	1	0	-5.422329	1.075644	-1.982977
30	1	0	-6.092952	-1.041182	1.677534
31	1	0	-6.976745	0.082081	-0.338500
32	6	0	-1.715030	-0.207286	0.489786
33	1	0	-1.419195	-0.747332	1.397227
34	1	0	1.002592	-1.026857	-1.746004
35	8	0	0.458644	-2.245363	-0.175504
36	1	0	0.926321	-3.026724	-0.489307

Structure 29b (M06-2X/ def2-TZVP, DMSO)

Energy (Hartrees): = -936.49032
No imaginary frequencies

Standard orientation:

Center Number	Atomic Number	Atomic Type	Coordinates (Angstroms) X	Y	Z
1	6	0	1.260088	-1.390713	-0.276624
2	6	0	0.539024	-0.088904	0.057570
3	6	0	1.200029	1.046242	-0.725129
4	6	0	2.698254	1.089740	-0.473201
5	6	0	3.301876	-0.291099	-0.706298
6	1	0	1.034745	0.871042	-1.794600
7	1	0	3.153782	1.787254	-1.184885
8	1	0	3.191784	-0.503880	-1.779635
9	1	0	0.659153	0.090992	1.133803
10	8	0	2.626829	-1.280418	0.050695
11	6	0	4.784638	-0.362850	-0.387462
12	1	0	5.160996	-1.341334	-0.690466
13	1	0	5.290234	0.394678	-0.999539
14	8	0	5.095871	-0.206628	0.980758
15	1	0	4.542237	0.510746	1.315697
16	8	0	2.965119	1.514839	0.856118
17	1	0	2.373254	2.254596	1.046780
18	8	0	0.682366	2.306254	-0.340932
19	1	0	-0.282005	2.271003	-0.388201
20	7	0	-0.848564	-0.164262	-0.337789
21	6	0	-3.175011	0.010761	0.261024
22	6	0	-3.665155	-0.193013	-1.030289
23	6	0	-4.068621	0.198995	1.312719
24	6	0	-5.030362	-0.209135	-1.259095
25	1	0	-2.967192	-0.335221	-1.845668
26	6	0	-5.438005	0.181675	1.082479
27	1	0	-3.684317	0.357462	2.314189
28	6	0	-5.919143	-0.022207	-0.203215
29	1	0	-5.408552	-0.365168	-2.261762
30	1	0	-6.127605	0.327735	1.904220
31	1	0	-6.986350	-0.034665	-0.386931
32	6	0	-1.730237	0.034975	0.548502
33	1	0	-1.458063	0.235016	1.591565
34	1	0	1.162938	-1.608880	-1.351295
35	8	0	0.710210	-2.402357	0.493443
36	1	0	1.097504	-3.241870	0.214709

Structure 29c (B3LYP, Gas Phase)

Energy (Hartrees): = -936.4753559
No imaginary frequencies

Standard orientation:

Center Number	Atomic Number	Atomic Type	Coordinates (Angstroms) X	Y	Z
1	6	0	-4.241814	0.568658	0.982648
2	6	0	-3.231472	0.027151	0.175488
3	6	0	-3.588117	-0.716014	-0.962155
4	6	0	-4.927821	-0.911151	-1.278711
5	6	0	-5.930105	-0.367904	-0.467013
6	6	0	-5.585176	0.372651	0.664002
7	6	0	-1.822207	0.244562	0.539585
8	7	0	-0.851810	-0.232782	-0.135581
9	6	0	0.494342	0.074400	0.293123
10	6	0	1.091121	1.159902	-0.646829
11	6	0	2.617844	1.272760	-0.482215
12	6	0	3.265586	-0.107123	-0.584793
13	8	0	2.724526	-0.918084	0.472531
14	6	0	1.332387	-1.211987	0.274210
15	8	0	0.482846	2.413060	-0.409872
16	8	0	0.910058	-2.016226	1.323843
17	6	0	4.780997	-0.128747	-0.409071
18	8	0	5.282382	-1.451531	-0.465665
19	8	0	2.914150	1.890935	0.774973
20	1	0	-1.655751	0.848850	1.444472
21	1	0	0.531685	0.461716	1.326073
22	1	0	0.973448	2.791278	0.338511
23	1	0	0.855909	0.878837	-1.680213
24	1	0	2.852854	1.186149	1.439539
25	1	0	3.008702	1.947124	-1.251799
26	1	0	4.770444	-1.952172	0.185569
27	1	0	5.248914	0.430448	-1.225418
28	1	0	5.045519	0.371535	0.533420
29	1	0	3.017827	-0.560588	-1.557877
30	1	0	1.346443	-2.875026	1.241790
31	1	0	1.221113	-1.712058	-0.703199
32	1	0	-3.969730	1.145932	1.862942
33	1	0	-6.359976	0.796629	1.295844

34	1	0	-6.975622	-0.522063	-0.717842
35	1	0	-5.197069	-1.486733	-2.159654
36	1	0	-2.797129	-1.128547	-1.579179

Structure 29c (B3LYP, DMSO)

Energy (Hartrees): = -936.501303
No imaginary frequencies

Standard orientation:

Center Number	Atomic Number	Atomic Type	Coordinates (Angstroms) X	Y	Z
1	6	0	-4.226873	0.477753	1.062318
2	6	0	-3.232530	0.019357	0.184264
3	6	0	-3.614591	-0.619034	-1.009037
4	6	0	-4.961537	-0.794308	-1.311000
5	6	0	-5.946826	-0.334776	-0.428606
6	6	0	-5.577138	0.301679	0.757898
7	6	0	-1.816859	0.215306	0.541747
8	7	0	-0.855110	-0.195730	-0.190295
9	6	0	0.499410	0.074154	0.249139
10	6	0	1.122255	1.168179	-0.660843
11	6	0	2.644323	1.279412	-0.453460
12	6	0	3.291019	-0.100489	-0.553150
13	8	0	2.702618	-0.941936	0.455946
14	6	0	1.322368	-1.221585	0.184658
15	8	0	0.506674	2.424778	-0.429564
16	8	0	0.855028	-2.087423	1.165357
17	6	0	4.794047	-0.127838	-0.308155
18	8	0	5.306317	-1.451135	-0.402890
19	8	0	2.909184	1.901726	0.811679
20	1	0	-1.639687	0.738387	1.492158
21	1	0	0.536189	0.427998	1.293110
22	1	0	0.957651	2.777719	0.356264
23	1	0	0.923641	0.899113	-1.704316
24	1	0	2.805009	1.208565	1.484522
25	1	0	3.056876	1.949242	-1.214336
26	1	0	4.751462	-1.985564	0.185736
27	1	0	5.296127	0.471751	-1.073773
28	1	0	5.018256	0.319379	0.670711
29	1	0	3.085578	-0.521333	-1.548913
30	1	0	1.289549	-2.944446	1.033818
31	1	0	1.248544	-1.664525	-0.822217
32	1	0	-3.935780	0.972900	1.985350
33	1	0	-6.338173	0.660495	1.444694
34	1	0	-6.997365	-0.473350	-0.667602
35	1	0	-5.248606	-1.289492	-2.234288
36	1	0	-2.844474	-0.971670	-1.687720

Structure 29c (M06-2X/6-311G(d,p), Gas Phase)

Energy (Hartrees): = -936.3453331
No imaginary frequencies

Standard orientation:

Center Number	Atomic Number	Atomic Type	Coordinates (Angstroms) X	Y	Z
1	6	0	-4.204286	0.683886	0.894853
2	6	0	-3.206720	0.034601	0.169352
3	6	0	-3.561319	-0.824930	-0.873526
4	6	0	-4.897204	-1.027574	-1.181170
5	6	0	-5.891328	-0.375265	-0.452587
6	6	0	-5.544457	0.480862	0.585251
7	6	0	-1.792809	0.264956	0.519042
8	7	0	-0.839549	-0.292955	-0.097440
9	6	0	0.504140	0.046122	0.304604
10	6	0	1.055400	1.116792	-0.659099
11	6	0	2.568262	1.269764	-0.490108
12	6	0	3.240768	-0.091586	-0.592619
13	8	0	2.733930	-0.902501	0.466653
14	6	0	1.356196	-1.218623	0.288624
15	8	0	0.412685	2.347545	-0.440316
16	8	0	0.956112	-2.009735	1.347105
17	6	0	4.748660	-0.063996	-0.424487
18	8	0	5.277038	-1.368936	-0.465569
19	8	0	2.837835	1.871335	0.769250
20	1	0	-1.613093	0.956272	1.353092
21	1	0	0.544636	0.446993	1.329671
22	1	0	0.855070	2.739452	0.322873
23	1	0	0.825428	0.810312	-1.683978
24	1	0	2.825489	1.161552	1.423186

25	1	0	2.948183	1.955913	-1.251079
26	1	0	4.793544	-1.872741	0.196163
27	1	0	5.196174	0.495833	-1.247879
28	1	0	4.999092	0.446602	0.512848
29	1	0	2.997864	-0.559515	-1.557529
30	1	0	1.393228	-2.862410	1.274824
31	1	0	1.238178	-1.729458	-0.679758
32	1	0	-3.927254	1.350989	1.704613
33	1	0	-6.315299	0.989072	1.151891
34	1	0	-6.934856	-0.536149	-0.696179
35	1	0	-5.170346	-1.693535	-1.990966
36	1	0	-2.771812	-1.320219	-1.425470

Structure 29c (M06-2X/6-311G(d,p), DMSO)

Energy (Hartrees): = -936.3744594
No imaginary frequencies

Standard orientation:

Center Number	Atomic Number	Atomic Type	Coordinates (Angstroms) X	Y	Z
1	6	0	-4.199396	0.505479	1.046239
2	6	0	-3.210607	0.025488	0.186127
3	6	0	-3.581934	-0.641188	-0.986333
4	6	0	-4.923478	-0.823697	-1.288006
5	6	0	-5.908080	-0.342475	-0.424200
6	6	0	-5.545033	0.322083	0.742488
7	6	0	-1.791357	0.227925	0.540298
8	7	0	-0.844200	-0.205011	-0.180290
9	6	0	0.508191	0.073683	0.248228
10	6	0	1.110993	1.150800	-0.674289
11	6	0	2.619833	1.274967	-0.450188
12	6	0	3.268535	-0.096804	-0.556180
13	8	0	2.694179	-0.934569	0.449171
14	6	0	1.325114	-1.213819	0.183571
15	8	0	0.479135	2.392751	-0.460897
16	8	0	0.857834	-2.069394	1.163682
17	6	0	4.763594	-0.101582	-0.311052
18	8	0	5.279062	-1.416773	-0.387831
19	8	0	2.859544	1.862408	0.824956
20	1	0	-1.605982	0.774783	1.473003
21	1	0	0.545883	0.433023	1.288389
22	1	0	0.867388	2.743675	0.351373
23	1	0	0.922830	0.864320	-1.712817
24	1	0	2.787807	1.154317	1.478916
25	1	0	3.038732	1.956423	-1.193748
26	1	0	4.745027	-1.945402	0.215741
27	1	0	5.257518	0.491606	-1.082839
28	1	0	4.978564	0.350730	0.664249
29	1	0	3.062860	-0.522622	-1.547442
30	1	0	1.275624	-2.928859	1.033683
31	1	0	1.246023	-1.658477	-0.819540
32	1	0	-3.909899	1.024356	1.954624
33	1	0	-6.307035	0.698003	1.415316
34	1	0	-6.955577	-0.487658	-0.662440
35	1	0	-5.206872	-1.342770	-2.196419
36	1	0	-2.809767	-1.010902	-1.650894

Structure 29c (M06-2X/6-311G(d,p), H_2O)

Energy (Hartrees): = -936.3852129
No imaginary frequencies

Standard orientation:

Center Number	Atomic Number	Atomic Type	Coordinates (Angstroms) X	Y	Z
1	6	0	-4.160454	0.631243	0.983360
2	6	0	-3.207840	0.018738	0.167147
3	6	0	-3.628367	-0.757141	-0.918335
4	6	0	-4.981849	-0.916018	-1.177103
5	6	0	-5.929428	-0.301862	-0.357398
6	6	0	-5.517744	0.472258	0.722248
7	6	0	-1.780534	0.209250	0.484171
8	7	0	-0.842771	-0.314574	-0.195801
9	6	0	0.508753	0.001420	0.221150
10	6	0	1.087412	1.083616	-0.705311
11	6	0	2.583880	1.279170	-0.452812
12	6	0	3.288652	-0.066952	-0.527454
13	8	0	2.721444	-0.933230	0.458989
14	6	0	1.367929	-1.256079	0.169343
15	8	0	0.392860	2.304820	-0.537262

16	8	0	0.918005	-2.132585	1.156420
17	6	0	4.772951	0.000682	-0.240615
18	8	0	5.373655	-1.277401	-0.383266
19	8	0	2.793044	1.912501	0.804908
20	1	0	-1.572751	0.836129	1.358921
21	1	0	0.542790	0.376592	1.254537
22	1	0	0.679837	2.660198	0.314725
23	1	0	0.945236	0.776072	-1.744428
24	1	0	2.735660	1.236386	1.493158
25	1	0	2.985046	1.954776	-1.211065
26	1	0	4.898221	-1.873142	0.206987
27	1	0	5.248520	0.667554	-0.961238
28	1	0	4.935767	0.397571	0.766779
29	1	0	3.132198	-0.499947	-1.524312
30	1	0	1.296237	-3.002168	0.980939
31	1	0	1.319992	-1.716517	-0.826136
32	1	0	-3.832881	1.232771	1.825107
33	1	0	-6.251425	0.949624	1.360778
34	1	0	-6.985967	-0.428908	-0.562508
35	1	0	-5.302406	-1.518389	-2.018987
36	1	0	-2.886909	-1.229379	-1.551310

Structure 29c (M06-2X/def2-TZVP, Gas Phase)

Energy (Hartrees): = -936.461986
No imaginary frequencies

Standard orientation:

Center Number	Atomic Number	Atomic Type	Coordinates (Angstroms) X	Y	Z
1	6	0	-4.203968	0.645833	0.925194
2	6	0	-3.209384	0.029865	0.172489
3	6	0	-3.566297	-0.788400	-0.899027
4	6	0	-4.900539	-0.983035	-1.207078
5	6	0	-5.891411	-0.363828	-0.450780
6	6	0	-5.542746	0.451299	0.615356
7	6	0	-1.797807	0.253795	0.526657
8	7	0	-0.841304	-0.266959	-0.110909
9	6	0	0.501256	0.055273	0.297104
10	6	0	1.072304	1.119313	-0.657438
11	6	0	2.582090	1.265026	-0.474725
12	6	0	3.243044	-0.100231	-0.572635
13	8	0	2.717681	-0.920785	0.463809
14	6	0	1.341444	-1.216476	0.274074
15	8	0	0.430866	2.353211	-0.460032
16	8	0	0.925904	-2.018064	1.319416
17	6	0	4.746923	-0.076532	-0.389536
18	8	0	5.286486	-1.374593	-0.473699
19	8	0	2.855556	1.882382	0.773189
20	1	0	-1.621934	0.907062	1.390604
21	1	0	0.541399	0.451941	1.323165
22	1	0	0.869859	2.773767	0.291306
23	1	0	0.860591	0.807853	-1.684195
24	1	0	2.820131	1.195338	1.451712
25	1	0	2.969809	1.937956	-1.242588
26	1	0	4.825438	-1.914091	0.177448
27	1	0	5.199468	0.514124	-1.186929
28	1	0	4.991515	0.397047	0.566849
29	1	0	3.012566	-0.550288	-1.548435
30	1	0	1.342843	-2.881938	1.243102
31	1	0	1.223281	-1.717204	-0.698164
32	1	0	-3.925049	1.281152	1.758100
33	1	0	-6.311249	0.934516	1.204480
34	1	0	-6.934541	-0.518863	-0.695056
35	1	0	-5.175347	-1.617709	-2.039748
36	1	0	-2.781037	-1.260558	-1.474689

Structure 29c (M06-2X/def2-TZVP, DMSO)

Energy (Hartrees): = -936.4912703
No imaginary frequencies

Standard orientation:

Center Number	Atomic Number	Atomic Type	Coordinates (Angstroms) X	Y	Z
1	6	0	-4.194411	0.458794	1.071719
2	6	0	-3.213106	0.015253	0.188394
3	6	0	-3.592324	-0.599582	-1.006263
4	6	0	-4.933385	-0.766646	-1.306335
5	6	0	-5.910134	-0.322023	-0.418698
6	6	0	-5.539756	0.291277	0.769954

7	6	0	-1.795635	0.207121	0.545430
8	7	0	-0.846035	-0.182717	-0.191605
9	6	0	0.505400	0.077584	0.241362
10	6	0	1.125835	1.152685	-0.666500
11	6	0	2.630509	1.271854	-0.428142
12	6	0	3.271343	-0.101726	-0.535905
13	8	0	2.678660	-0.956442	0.437725
14	6	0	1.312348	-1.214567	0.159541
15	8	0	0.494088	2.396450	-0.470163
16	8	0	0.833124	-2.087743	1.119082
17	6	0	4.760291	-0.106715	-0.270021
18	8	0	5.298456	-1.408042	-0.405088
19	8	0	2.873411	1.871414	0.837781
20	1	0	-1.611882	0.710231	1.501807
21	1	0	0.543739	0.425374	1.284758
22	1	0	0.872237	2.770845	0.337920
23	1	0	0.956236	0.865262	-1.707368
24	1	0	2.772951	1.187100	1.514797
25	1	0	3.055873	1.943630	-1.175808
26	1	0	4.791067	-1.984468	0.179565
27	1	0	5.258104	0.529854	-1.002495
28	1	0	4.960339	0.295125	0.728565
29	1	0	3.083925	-0.503680	-1.540106
30	1	0	1.225449	-2.957480	0.969492
31	1	0	1.234356	-1.642317	-0.849804
32	1	0	-3.897878	0.937599	1.998456
33	1	0	-6.296260	0.640074	1.461716
34	1	0	-6.958305	-0.455010	-0.656600
35	1	0	-5.223629	-1.245255	-2.233501
36	1	0	-2.827845	-0.942803	-1.692068

Structure 29.1H$_2$O (M06-2X/6-311G(d,p), Gas Phase)

Energy (Hartrees): = -1012.7767086
No imaginary frequencies

Standard orientation:

Center Number	Atomic Number	Atomic Type	Coordinates (Angstroms) X	Y	Z
1	6	0	4.067469	-1.323839	0.499562
2	6	0	3.148547	-0.315189	0.208078
3	6	0	3.601152	0.910981	-0.290524
4	6	0	4.957076	1.109178	-0.501749
5	6	0	5.869414	0.093268	-0.222607
6	6	0	5.424747	-1.124374	0.278774
7	6	0	1.719126	-0.574439	0.448228
8	7	0	0.794204	0.179378	0.018320
9	6	0	-0.572933	-0.192730	0.311468
10	6	0	-1.100274	-1.169461	-0.759819
11	6	0	-2.607332	-1.385054	-0.582372
12	6	0	-3.324157	-0.044563	-0.509417
13	8	0	-2.790736	0.682121	0.599947
14	6	0	-1.442832	1.067299	0.368695
15	8	0	-0.420320	-2.397370	-0.687394
16	8	0	-1.047160	1.947937	1.360573
17	6	0	-4.820815	-0.130750	-0.276717
18	8	0	-5.382223	1.156815	-0.204883
19	8	0	-2.826159	-2.144761	0.599977
20	1	0	1.488679	-1.476879	1.027418
21	1	0	-0.649782	-0.699114	1.289161
22	1	0	-0.863872	-2.902248	0.006116
23	1	0	-0.897301	-0.742425	-1.746870
24	1	0	-2.866027	-1.516981	1.331058
25	1	0	-2.983269	-1.986029	-1.414113
26	1	0	-4.859876	1.645540	0.439390
27	1	0	-5.290559	-0.648120	-1.115425
28	1	0	-5.015743	-0.710912	0.633560
29	1	0	-3.141980	0.525646	-1.430856
30	1	0	-0.965096	1.466119	2.189875
31	1	0	-1.374232	1.624547	-0.572172
32	1	0	3.713016	-2.272085	0.890549
33	1	0	6.132222	-1.914702	0.498117
34	1	0	6.927309	0.254972	-0.392542
35	1	0	5.306494	2.061079	-0.882991
36	1	0	2.893855	1.706991	-0.493166
37	1	0	0.860653	2.002015	-0.786043
38	8	0	0.729684	2.959105	-0.850566
39	1	0	0.406286	3.174209	0.029860

Structure 29.1H$_2$O (M06-2X/6-311G(d,p), DMSO)

Energy (Hartrees): = -1012.8096903
No imaginary frequencies

Standard orientation:

Center Number	Atomic Number	Atomic Type	Coordinates (Angstroms) X	Y	Z
1	6	0	4.039016	-1.306894	0.593180
2	6	0	3.141850	-0.301386	0.227268
3	6	0	3.626881	0.885593	-0.332877
4	6	0	4.989753	1.048912	-0.536957
5	6	0	5.879553	0.032989	-0.188181
6	6	0	5.403769	-1.144203	0.379651
7	6	0	1.703636	-0.530337	0.458111
8	7	0	0.791472	0.191589	-0.050473
9	6	0	-0.578756	-0.173539	0.257345
10	6	0	-1.122900	-1.139027	-0.813551
11	6	0	-2.619761	-1.378709	-0.589155
12	6	0	-3.350382	-0.048167	-0.484658
13	8	0	-2.786774	0.702245	0.596694
14	6	0	-1.449036	1.084144	0.319792
15	8	0	-0.421192	-2.359837	-0.790537
16	8	0	-1.024281	1.978984	1.289938
17	6	0	-4.830888	-0.168672	-0.187779
18	8	0	-5.426506	1.110835	-0.096979
19	8	0	-2.796851	-2.166696	0.583427
20	1	0	1.459479	-1.380544	1.104198
21	1	0	-0.639591	-0.671855	1.238057
22	1	0	-0.776657	-2.851483	-0.038195
23	1	0	-0.968986	-0.692164	-1.799739
24	1	0	-2.760768	-1.568523	1.341501
25	1	0	-3.017193	-1.963025	-1.421971
26	1	0	-4.894252	1.606891	0.535081
27	1	0	-5.320974	-0.704179	-1.002869
28	1	0	-4.976098	-0.738656	0.737332
29	1	0	-3.213085	0.511391	-1.419444
30	1	0	-0.878807	1.494829	2.113515
31	1	0	-1.415366	1.623252	-0.633830
32	1	0	3.660855	-2.220989	1.039706
33	1	0	6.092777	-1.932525	0.659111
34	1	0	6.942840	0.165451	-0.352605
35	1	0	5.363726	1.972201	-0.964265
36	1	0	2.938762	1.684778	-0.583223
37	1	0	0.877502	2.017909	-0.814948
38	8	0	0.781944	2.983962	-0.835313
39	1	0	0.378408	3.145947	0.024913

Structure 29.1H_2O (M06-2X/6-311G(d,p), H_2O)

Energy (Hartrees): = -1012.8233544
No imaginary frequencies

Standard orientation:

Center Number	Atomic Number	Atomic Type	Coordinates (Angstroms) X	Y	Z
1	6	0	4.033500	-1.276457	0.661461
2	6	0	3.144310	-0.295673	0.215869
3	6	0	3.641946	0.849511	-0.415527
4	6	0	5.008194	0.997271	-0.606403
5	6	0	5.890063	0.007556	-0.172046
6	6	0	5.402248	-1.128813	0.464198
7	6	0	1.705158	-0.513158	0.443230
8	7	0	0.792441	0.221139	-0.053126
9	6	0	-0.577163	-0.156519	0.254589
10	6	0	-1.120726	-1.106007	-0.827650
11	6	0	-2.611208	-1.371469	-0.599553
12	6	0	-3.356409	-0.049874	-0.477812
13	8	0	-2.795699	0.702600	0.602845
14	6	0	-1.457556	1.091359	0.335589
15	8	0	-0.398919	-2.321576	-0.843115
16	8	0	-1.048737	1.971778	1.336397
17	6	0	-4.830546	-0.205349	-0.171421
18	8	0	-5.466006	1.061690	-0.097041
19	8	0	-2.785894	-2.183153	0.556516
20	1	0	1.456846	-1.367228	1.081863
21	1	0	-0.634686	-0.668789	1.226688
22	1	0	-0.672172	-2.809751	-0.054113
23	1	0	-0.985982	-0.641775	-1.808030
24	1	0	-2.700113	-1.615386	1.333630
25	1	0	-3.002989	-1.943615	-1.442965
26	1	0	-4.984237	1.572151	0.564372
27	1	0	-5.309801	-0.765170	-0.975672
28	1	0	-4.953309	-0.758219	0.765318
29	1	0	-3.234306	0.517666	-1.409778
30	1	0	-0.897093	1.467411	2.146569

31	1	0	-1.425443	1.651700	-0.604919
32	1	0	3.645044	-2.158669	1.159636
33	1	0	6.084916	-1.896034	0.809267
34	1	0	6.956046	0.128413	-0.325866
35	1	0	5.390573	1.887566	-1.091274
36	1	0	2.962032	1.628459	-0.738262
37	1	0	0.876112	2.038844	-0.740859
38	8	0	0.778220	3.004885	-0.827222
39	1	0	0.386891	3.240394	0.022106

Structure 29.5H_2O (M06-2X/6-311G(d,p), Gas Phase)

Energy (Hartrees): = -1318.5172021
No imaginary frequencies

Standard orientation:

Center Number	Atomic Number	Atomic Type	Coordinates (Angstroms) X	Y	Z
1	6	0	4.232415	0.232540	0.707738
2	6	0	3.663922	-0.405959	-0.399495
3	6	0	4.489243	-1.072819	-1.305816
4	6	0	5.866164	-1.098164	-1.121322
5	6	0	6.425543	-0.458721	-0.021863
6	6	0	5.606431	0.203821	0.890408
7	6	0	2.211884	-0.411303	-0.645280
8	7	0	1.371866	0.246690	0.039004
9	6	0	-0.033993	0.083099	-0.282090
10	6	0	-0.643619	-0.970530	0.666190
11	8	0	-2.042715	-1.021356	0.491311
12	6	0	-2.750669	0.188286	0.707292
13	6	0	-2.270673	1.226388	-0.292451
14	6	0	-0.758978	1.424515	-0.194218
15	8	0	-0.169422	-2.239976	0.366274
16	6	0	-4.226971	-0.154397	0.544641
17	8	0	-4.635193	-1.150529	1.438323
18	8	0	-2.586079	0.799486	-1.627605
19	8	0	-0.300457	2.188850	-1.300761
20	8	0	-0.724462	1.320666	3.047874
21	8	0	1.713065	2.137789	2.061280
22	8	0	-1.976958	-2.129459	-1.970374
23	8	0	-3.358163	-3.445203	0.279151
24	1	0	1.879733	-1.036628	-1.482996
25	1	0	-0.529854	1.909491	0.758815
26	1	0	-0.839062	2.990019	-1.374676
27	1	0	-1.872033	1.156956	-2.177341
28	1	0	-2.769678	2.181583	-0.092491
29	1	0	-2.550404	0.559161	1.719136
30	1	0	-4.814477	0.745833	0.749595
31	1	0	-4.407597	-0.444992	-0.499036
32	1	0	-4.298635	-1.996730	1.099286
33	1	0	-0.525124	-2.455468	-0.510452
34	1	0	-0.390487	-0.737556	1.703712
35	1	0	-0.159752	-0.306264	-1.302988
36	1	0	3.599265	0.742904	1.423417
37	1	0	6.043501	0.695073	1.751326
38	1	0	7.498293	-0.478812	0.129060
39	1	0	6.498760	-1.617887	-1.830395
40	1	0	4.046400	-1.574039	-2.160236
41	1	0	1.660800	1.488818	1.322988
42	1	0	1.871271	2.988521	1.646061
43	1	0	-2.296991	-1.244464	-1.729174
44	1	0	-2.095023	-2.215919	-2.918845
45	1	0	0.162400	1.689611	2.893263
46	1	0	-0.706408	0.958744	3.935644
47	1	0	-2.589204	-3.222543	0.816068
48	1	0	-3.080987	-3.120661	-0.588668
49	8	0	-2.602406	3.617953	-2.117530
50	1	0	-3.202896	2.866855	-2.184493
51	1	0	-3.136738	4.405267	-2.235554

Structure 29.5H_2O (M06-2X/6-311G(d,p), DMSO)

Energy (Hartrees): = -1318.5602845
No imaginary frequencies

Standard orientation:

Center Number	Atomic Number	Atomic Type	Coordinates (Angstroms) X	Y	Z
1	6	0	4.129452	0.045995	0.859467
2	6	0	3.621308	-0.607277	-0.269386
3	6	0	4.498173	-1.260341	-1.138242

4	6	0	5.867211	-1.255691	-0.892995
5	6	0	6.366491	-0.600978	0.227615
6	6	0	5.495285	0.047623	1.102955
7	6	0	2.181995	-0.639375	-0.584148
8	7	0	1.303181	-0.005512	0.075724
9	6	0	-0.081600	-0.138732	-0.338430
10	6	0	-0.860962	-0.853079	0.778103
11	8	0	-2.249815	-0.824297	0.525774
12	6	0	-2.846870	0.447884	0.305905
13	6	0	-2.173916	1.117116	-0.880001
14	6	0	-0.671399	1.238871	-0.635539
15	8	0	-0.516102	-2.195890	0.857246
16	6	0	-4.324270	0.171952	0.064670
17	8	0	-4.903354	-0.565612	1.116324
18	8	0	-2.390103	0.314766	-2.041579
19	8	0	-0.031701	1.723354	-1.800141
20	8	0	-1.190268	1.934630	2.863240
21	8	0	1.463672	1.982008	2.035360
22	8	0	-2.072623	-2.565845	-1.570515
23	8	0	-3.692781	-3.172455	0.713054
24	1	0	1.901202	-1.252893	-1.447465
25	1	0	-0.503903	1.902020	0.222409
26	1	0	-0.215943	2.677868	-1.838032
27	1	0	-1.740261	0.615820	-2.692314
28	1	0	-2.600388	2.116793	-1.028143
29	1	0	-2.705845	1.076064	1.193694
30	1	0	-4.849290	1.127860	-0.009554
31	1	0	-4.436207	-0.351563	-0.892434
32	1	0	-4.561038	-1.470448	1.036815
33	1	0	-0.792767	-2.581912	0.008670
34	1	0	-0.639763	-0.389542	1.745874
35	1	0	-0.162503	-0.750156	-1.246817
36	1	0	3.456939	0.546605	1.546354
37	1	0	5.885227	0.552233	1.979281
38	1	0	7.432555	-0.596484	0.423706
39	1	0	6.541083	-1.762168	-1.573932
40	1	0	4.101394	-1.769751	-2.010427
41	1	0	1.480869	1.283058	1.342525
42	1	0	1.781685	2.779404	1.598850
43	1	0	-2.254773	-1.615128	-1.677403
44	1	0	-1.971990	-2.922679	-2.459690
45	1	0	-0.244903	2.000159	2.638991
46	1	0	-1.237293	1.214973	3.499735
47	1	0	-2.965825	-2.802335	1.228486
48	1	0	-3.342125	-3.061551	-0.185095
49	8	0	-0.815965	4.359597	-1.570735
50	1	0	-1.701631	4.567437	-1.889306
51	1	0	-0.789867	4.699041	-0.668912

Structure 29.5H$_2$O (M06-2X/6-311G(d,p), H$_2$O)

Energy (Hartrees): = -1318.5843831
No imaginary frequencies

Standard orientation:

Center Number	Atomic Number	Atomic Type	Coordinates (Angstroms) X	Y	Z
1	6	0	4.177755	0.193065	0.802235
2	6	0	3.667037	-0.543876	-0.272561
3	6	0	4.541196	-1.261231	-1.091810
4	6	0	5.909739	-1.247885	-0.844163
5	6	0	6.411349	-0.515322	0.226120
6	6	0	5.543092	0.204420	1.047477
7	6	0	2.228202	-0.597587	-0.581124
8	7	0	1.339795	0.005905	0.098111
9	6	0	-0.041777	-0.140939	-0.327586
10	6	0	-0.831225	-0.806283	0.808302
11	8	0	-2.219393	-0.794277	0.558094
12	6	0	-2.820356	0.455643	0.241764
13	6	0	-2.125342	1.082148	-0.953625
14	6	0	-0.631037	1.222888	-0.673658
15	8	0	-0.482126	-2.156462	0.935943
16	6	0	-4.283991	0.144240	-0.029758
17	8	0	-4.874845	-0.557979	1.050380
18	8	0	-2.321302	0.241419	-2.089739
19	8	0	0.038858	1.693845	-1.830230
20	8	0	-1.394014	2.054799	2.981492
21	8	0	1.287185	1.826123	2.248035
22	8	0	-2.047939	-2.619630	-1.505818
23	8	0	-3.687227	-3.167146	0.729493
24	1	0	1.954125	-1.202926	-1.451716
25	1	0	-0.491152	1.909042	0.172156
26	1	0	-0.225461	2.627596	-1.935291
27	1	0	-1.648790	0.494914	-2.737000

28	1	0	-2.550603	2.074999	-1.141718
29	1	0	-2.727676	1.134498	1.097284
30	1	0	-4.826879	1.082159	-0.162318
31	1	0	-4.363400	-0.438905	-0.952923
32	1	0	-4.525349	-1.464569	1.008665
33	1	0	-0.778074	-2.579019	0.110043
34	1	0	-0.607424	-0.313406	1.761748
35	1	0	-0.117047	-0.784662	-1.213320
36	1	0	3.506591	0.753877	1.442199
37	1	0	5.935292	0.775173	1.880793
38	1	0	7.476861	-0.503003	0.423258
39	1	0	6.580448	-1.809380	-1.483388
40	1	0	4.141745	-1.830868	-1.924409
41	1	0	1.365934	1.164962	1.525036
42	1	0	1.462540	2.668061	1.812642
43	1	0	-2.182938	-1.671825	-1.682859
44	1	0	-2.006776	-3.041689	-2.370597
45	1	0	-0.465067	1.914279	2.718911
46	1	0	-1.732869	1.168672	3.143443
47	1	0	-3.004396	-2.869036	1.342982
48	1	0	-3.255690	-3.004340	-0.128766
49	8	0	-1.011469	4.248428	-1.724572
50	1	0	-1.892433	4.144753	-2.102493
51	1	0	-1.169750	4.270681	-0.773436

Structure 29.5H_2O (M06-2X/def2-TZVP, Gas Phase)

Energy (Hartrees): = -1318.6729408
No imaginary frequencies

Standard orientation:

Center Number	Atomic Number	Atomic Type	Coordinates (Angstroms) X	Y	Z
1	6	0	4.236894	0.075833	0.769334
2	6	0	3.675885	-0.416412	-0.410020
3	6	0	4.507060	-0.946204	-1.394084
4	6	0	5.882275	-0.972165	-1.216957
5	6	0	6.434252	-0.474019	-0.046317
6	6	0	5.609117	0.046027	0.945507
7	6	0	2.226407	-0.407500	-0.655280
8	7	0	1.382027	0.188927	0.073163
9	6	0	-0.019533	0.054162	-0.263497
10	6	0	-0.666610	-0.979902	0.676061
11	8	0	-2.058921	-1.011425	0.470424
12	6	0	-2.746232	0.209440	0.670829
13	6	0	-2.229602	1.242369	-0.314579
14	6	0	-0.719508	1.406032	-0.180115
15	8	0	-0.198324	-2.257282	0.406823
16	6	0	-4.224838	-0.091923	0.479732
17	8	0	-4.695425	-1.043414	1.391371
18	8	0	-2.538129	0.835561	-1.653319
19	8	0	-0.214904	2.180881	-1.255457
20	8	0	-0.774760	1.389086	3.125824
21	8	0	1.752869	2.009540	2.174245
22	8	0	-2.056948	-2.179718	-2.008909
23	8	0	-3.494382	-3.448977	0.287237
24	1	0	1.898488	-0.963348	-1.541325
25	1	0	-0.509459	1.871594	0.786601
26	1	0	-0.730352	2.997144	-1.335776
27	1	0	-1.809388	1.166143	-2.201467
28	1	0	-2.710370	2.204883	-0.107102
29	1	0	-2.562256	0.577565	1.686523
30	1	0	-4.788329	0.831861	0.635994
31	1	0	-4.389909	-0.414252	-0.555441
32	1	0	-4.405549	-1.918633	1.083537
33	1	0	-0.525991	-2.495632	-0.475324
34	1	0	-0.435582	-0.742145	1.718377
35	1	0	-0.141243	-0.333628	-1.285126
36	1	0	3.597329	0.470500	1.547655
37	1	0	6.039533	0.425409	1.863210
38	1	0	7.506659	-0.495806	0.098930
39	1	0	6.520539	-1.382658	-1.988260
40	1	0	4.070271	-1.338495	-2.305295
41	1	0	1.691356	1.388190	1.410443
42	1	0	1.966938	2.869860	1.804375
43	1	0	-2.318622	-1.278953	-1.752728
44	1	0	-2.221297	-2.262854	-2.951897
45	1	0	0.143698	1.679594	2.980039
46	1	0	-0.853755	1.183250	4.059896
47	1	0	-2.735270	-3.272134	0.855765
48	1	0	-3.181999	-3.140239	-0.576086
49	8	0	-2.529436	3.676566	-2.140464
50	1	0	-3.130474	2.925546	-2.219944

51	1	0	-2.985689	4.443770	-2.493586

Structure 29.5H$_2$O (M06-2X/def2-TZVP, DMSO)

Energy (Hartrees): = -1318.7161264
No imaginary frequencies

Standard orientation:

Center Number	Atomic Number	Atomic Type	Coordinates (Angstroms) X	Y	Z
1	6	0	4.046840	-0.494372	0.993095
2	6	0	3.601503	-0.651720	-0.320700
3	6	0	4.529502	-0.861271	-1.339000
4	6	0	5.888169	-0.892389	-1.055428
5	6	0	6.324820	-0.726744	0.251581
6	6	0	5.401838	-0.532449	1.275518
7	6	0	2.172849	-0.614609	-0.671112
8	7	0	1.272817	-0.203271	0.118404
9	6	0	-0.100488	-0.212561	-0.341503
10	6	0	-0.963753	-0.912476	0.716050
11	8	0	-2.335016	-0.776522	0.432612
12	6	0	-2.821512	0.545318	0.268017
13	6	0	-2.077713	1.223429	-0.867138
14	6	0	-0.577356	1.218156	-0.589170
15	8	0	-0.707781	-2.276278	0.759462
16	6	0	-4.309560	0.420510	-0.008541
17	8	0	-4.987706	-0.275814	1.011729
18	8	0	-2.336158	0.512001	-2.074526
19	8	0	0.123067	1.706647	-1.713286
20	8	0	-1.234252	2.062820	3.061101
21	8	0	1.391281	1.292276	2.495610
22	8	0	-2.336249	-2.439501	-1.774514
23	8	0	-4.055902	-3.010084	0.492296
24	1	0	1.920781	-0.965621	-1.676890
25	1	0	-0.381287	1.829296	0.300235
26	1	0	0.118694	2.680525	-1.676943
27	1	0	-1.667281	0.805479	-2.710678
28	1	0	-2.424663	2.258950	-0.959380
29	1	0	-2.652468	1.119869	1.187281
30	1	0	-4.736586	1.423453	-0.074790
31	1	0	-4.454374	-0.072127	-0.975681
32	1	0	-4.761769	-1.214870	0.909649
33	1	0	-0.972596	-2.630070	-0.106544
34	1	0	-0.740543	-0.503236	1.709494
35	1	0	-0.198925	-0.768673	-1.282456
36	1	0	3.329546	-0.362252	1.792979
37	1	0	5.743428	-0.416778	2.296498
38	1	0	7.383766	-0.755457	0.476804
39	1	0	6.603990	-1.049183	-1.852375
40	1	0	4.179995	-0.995078	-2.356508
41	1	0	1.436529	0.761168	1.665997
42	1	0	2.064095	1.977775	2.413554
43	1	0	-2.386741	-1.468451	-1.842934
44	1	0	-2.313695	-2.778900	-2.676926
45	1	0	-0.306884	1.815823	2.887880
46	1	0	-1.578054	1.368481	3.632633
47	1	0	-3.341466	-2.790447	1.104093
48	1	0	-3.632391	-2.851253	-0.368833
49	8	0	0.043374	4.478780	-1.477008
50	1	0	-0.827388	4.893221	-1.516461
51	1	0	0.478480	4.863699	-0.706333

Structure 29.5H$_2$O (M06-2X/def2-TZVP, H$_2$O)

Energy (Hartrees): = -1318.7384629
No imaginary frequencies

Standard orientation:

Center Number	Atomic Number	Atomic Type	Coordinates (Angstroms) X	Y	Z
1	6	0	4.199206	0.123845	0.805005
2	6	0	3.665780	-0.623370	-0.247172
3	6	0	4.518513	-1.366320	-1.061574
4	6	0	5.887847	-1.364796	-0.833940
5	6	0	6.411533	-0.620551	0.213716
6	6	0	5.564770	0.122477	1.032328
7	6	0	2.225651	-0.666352	-0.538813
8	7	0	1.350198	-0.009234	0.102290
9	6	0	-0.032501	-0.163614	-0.305979
10	6	0	-0.838192	-0.700377	0.881257
11	8	0	-2.217051	-0.724492	0.599879

12	6	0	-2.797336	0.504257	0.190332
13	6	0	-2.092978	1.034753	-1.044723
14	6	0	-0.599498	1.172195	-0.769928
15	8	0	-0.475505	-2.016971	1.183124
16	6	0	-4.264933	0.217767	-0.066138
17	8	0	-4.903337	-0.326378	1.074992
18	8	0	-2.308977	0.127653	-2.119982
19	8	0	0.085233	1.549056	-1.949972
20	8	0	-1.282204	2.320886	3.071609
21	8	0	1.343965	1.851373	2.251056
22	8	0	-2.201984	-2.736084	-1.381267
23	8	0	-4.134412	-3.055999	0.679171
24	1	0	1.936429	-1.317362	-1.369762
25	1	0	-0.457682	1.923113	0.017309
26	1	0	-0.119844	2.491603	-2.107509
27	1	0	-1.652576	0.332289	-2.801630
28	1	0	-2.502152	2.019888	-1.295482
29	1	0	-2.696397	1.245828	0.992316
30	1	0	-4.766161	1.151219	-0.326882
31	1	0	-4.356724	-0.471336	-0.910161
32	1	0	-4.677706	-1.274236	1.088236
33	1	0	-0.679927	-2.551422	0.397803
34	1	0	-0.641284	-0.095592	1.774021
35	1	0	-0.126301	-0.885434	-1.127270
36	1	0	3.544203	0.701360	1.444643
37	1	0	5.975169	0.701247	1.849945
38	1	0	7.478580	-0.617954	0.396779
39	1	0	6.542279	-1.945849	-1.470891
40	1	0	4.100402	-1.946425	-1.876212
41	1	0	1.408706	1.208994	1.507604
42	1	0	1.710858	2.673987	1.905676
43	1	0	-2.233835	-1.780808	-1.571187
44	1	0	-2.315165	-3.174659	-2.233007
45	1	0	-0.382636	2.095931	2.762394
46	1	0	-1.751562	1.480301	3.114328
47	1	0	-3.588563	-3.175591	1.465862
48	1	0	-3.490105	-2.929146	-0.044748
49	8	0	-0.699813	4.220648	-1.964353
50	1	0	-1.578604	4.227297	-2.363690
51	1	0	-0.865278	4.280368	-1.014804

Structure 38β (B3LYP, Gas Phase)

Energy (Hartrees): = -1547.1349088
No imaginary frequencies

Standard orientation:

Center Number	Atomic Number	Atomic Type	Coordinates (Angstroms) X	Y	Z
1	6	0	0.055453	1.640088	-0.598050
2	6	0	0.546704	0.286491	-0.059136
3	6	0	-0.172491	-0.814040	-0.853153
4	6	0	-1.699885	-0.641669	-0.828401
5	6	0	-2.041633	0.784488	-1.279962
6	1	0	0.387327	1.789951	-1.633426
7	1	0	0.160254	-0.785695	-1.895278
8	1	0	-2.153093	-1.373511	-1.501773
9	1	0	-1.715136	0.845095	-2.334426
10	1	0	0.257193	0.216671	1.000775
11	8	0	-1.348201	1.753967	-0.503770
12	6	0	-3.521176	1.171805	-1.286700
13	1	0	-3.637305	2.074858	-1.890157
14	1	0	-4.126414	0.364052	-1.704785
15	8	0	-4.004344	1.525127	0.018038
16	8	0	-2.202915	-0.865111	0.504087
17	8	0	0.197644	-2.078464	-0.289384
18	8	0	0.594783	2.656509	0.224152
19	7	0	1.973816	0.181094	-0.275769
20	6	0	4.175079	-0.225921	0.628893
21	6	0	4.843027	-0.084010	-0.599188
22	6	0	4.917588	-0.510932	1.783781
23	6	0	6.224742	-0.225199	-0.662925
24	1	0	4.257512	0.134462	-1.485820
25	6	0	6.303218	-0.652282	1.718115
26	1	0	4.403556	-0.622344	2.735502
27	6	0	6.958413	-0.509583	0.494491
28	1	0	6.735791	-0.115210	-1.614889
29	1	0	6.869403	-0.873307	2.617982
30	6	0	2.714051	-0.083894	0.728037
31	1	0	2.292622	-0.223310	1.736245
32	6	0	-4.872649	0.690783	0.631640
33	6	0	-2.664631	-2.117086	0.783970
34	6	0	0.793999	3.881297	-0.357459
35	6	0	0.249373	-3.133872	-1.156202

36	8	0	0.577076	4.116933	-1.520769
37	8	0	-5.270395	-0.354460	0.162386
38	8	0	-2.646385	-3.037444	-0.001748
39	8	0	0.087163	-3.024633	-2.349383
40	6	0	1.319521	4.857394	0.663073
41	6	0	0.496780	-4.418483	-0.412984
42	6	0	-3.230570	-2.184027	2.178771
43	6	0	-5.261966	1.246703	1.979578
44	1	0	1.531261	5.809747	0.178742
45	1	0	0.576853	4.998504	1.454067
46	1	0	2.224863	4.460905	1.130973
47	1	0	-0.454731	-4.740831	0.022349
48	1	0	0.848993	-5.179435	-1.109010
49	1	0	1.213169	-4.274203	0.398457
50	1	0	-3.175832	-3.211059	2.540604
51	1	0	-2.706677	-1.507880	2.856817
52	1	0	-4.282464	-1.886234	2.127540
53	1	0	-6.059764	0.641136	2.408405
54	1	0	-4.390399	1.235408	2.641355
55	1	0	-5.584332	2.286588	1.884129
56	1	0	8.037561	-0.619696	0.440002

Structure 38β (B3LYP, $CHCl_3$)

Energy (Hartrees): = -1547.1670084
No imaginary frequencies

Standard orientation:

Center Number	Atomic Number	Atomic Type	Coordinates (Angstroms) X	Y	Z
1	6	0	0.061251	1.651136	-0.607049
2	6	0	0.548095	0.290283	-0.083447
3	6	0	-0.189294	-0.801299	-0.874631
4	6	0	-1.714195	-0.615405	-0.834050
5	6	0	-2.052380	0.815619	-1.269802
6	1	0	0.377823	1.805619	-1.645657
7	1	0	0.134387	-0.777387	-1.918679
8	1	0	-2.188682	-1.332655	-1.506693
9	1	0	-1.739754	0.887406	-2.325275
10	1	0	0.271089	0.217621	0.978937
11	8	0	-1.339586	1.774233	-0.487215
12	6	0	-3.525562	1.210485	-1.253997
13	1	0	-3.637970	2.148969	-1.801893
14	1	0	-4.131353	0.435684	-1.727889
15	8	0	-4.010749	1.480010	0.074078
16	8	0	-2.191209	-0.847131	0.509135
17	8	0	0.179810	-2.067264	-0.302914
18	8	0	0.622620	2.656120	0.219244
19	7	0	1.974842	0.171116	-0.311069
20	6	0	4.177857	-0.219411	0.607067
21	6	0	4.856747	-0.130724	-0.620973
22	6	0	4.909489	-0.471213	1.777916
23	6	0	6.237841	-0.289545	-0.669443
24	1	0	4.286445	0.061024	-1.524051
25	6	0	6.294259	-0.630206	1.727238
26	1	0	4.387026	-0.541995	2.728784
27	6	0	6.959971	-0.540330	0.503536
28	1	0	6.756629	-0.220278	-1.621240
29	1	0	6.851605	-0.824633	2.638893
30	6	0	2.717775	-0.064921	0.700429
31	1	0	2.298712	-0.169470	1.711959
32	6	0	-4.922718	0.641445	0.612901
33	6	0	-2.671234	-2.087362	0.792634
34	6	0	0.811344	3.891642	-0.337182
35	6	0	0.263168	-3.130924	-1.147925
36	8	0	0.556025	4.150638	-1.491257
37	8	0	-5.348601	-0.352809	0.058879
38	8	0	-2.733894	-2.990397	-0.015395
39	8	0	0.076748	-3.050575	-2.343325
40	6	0	1.370774	4.843039	0.681941
41	6	0	0.598457	-4.385849	-0.392095
42	6	0	-3.141098	-2.177154	2.219322
43	6	0	-5.329988	1.118801	1.981535
44	1	0	1.571264	5.806696	0.214152
45	1	0	0.652780	4.970532	1.498483
46	1	0	2.290081	4.435919	1.113225
47	1	0	-0.282490	-4.700451	0.177289
48	1	0	0.872219	-5.174395	-1.093195
49	1	0	1.411321	-4.207160	0.316298
50	1	0	-3.002662	-3.197676	2.580457
51	1	0	-2.616601	-1.470490	2.864397
52	1	0	-4.212217	-1.950535	2.244899
53	1	0	-6.115182	0.476050	2.379399
54	1	0	-4.464207	1.105249	2.651038

55	1	0	-5.685058	2.152208	1.929595
56	1	0	8.038225	-0.664917	0.460864

Structure 38β (M06-2X/6-311G(d,p), Gas Phase)

Energy (Hartrees): = -1546.9160046
No imaginary frequencies

Standard orientation:

Center Number	Atomic Number	Atomic Type	Coordinates (Angstroms) X	Y	Z
1	6	0	0.070600	1.688099	-0.552734
2	6	0	0.547957	0.327551	-0.054530
3	6	0	-0.168097	-0.741914	-0.875316
4	6	0	-1.685425	-0.552673	-0.826761
5	6	0	-2.004160	0.875463	-1.262767
6	1	0	0.393504	1.852749	-1.588295
7	1	0	0.161452	-0.682718	-1.916689
8	1	0	-2.170674	-1.276970	-1.484588
9	1	0	-1.653639	0.961092	-2.305144
10	1	0	0.263604	0.227656	1.001907
11	8	0	-1.322568	1.809599	-0.450897
12	6	0	-3.471717	1.269810	-1.283276
13	1	0	-3.555970	2.237902	-1.775325
14	1	0	-4.057765	0.519894	-1.816761
15	8	0	-3.997464	1.468877	0.025982
16	8	0	-2.142421	-0.749684	0.515791
17	8	0	0.199992	-2.004765	-0.338786
18	8	0	0.627683	2.668924	0.280692
19	7	0	1.969409	0.220989	-0.282058
20	6	0	4.155199	-0.296518	0.573467
21	6	0	4.806013	-0.084259	-0.644889
22	6	0	4.895759	-0.669826	1.694209
23	6	0	6.179371	-0.245493	-0.733910
24	1	0	4.213569	0.203243	-1.505006
25	6	0	6.273860	-0.830726	1.603968
26	1	0	4.389209	-0.835042	2.639685
27	6	0	6.915866	-0.618671	0.389989
28	1	0	6.681888	-0.081954	-1.679706
29	1	0	6.844500	-1.120520	2.478073
30	6	0	2.694644	-0.132977	0.691888
31	1	0	2.267056	-0.338988	1.682936
32	6	0	-4.715594	0.478050	0.574455
33	6	0	-2.542089	-2.000747	0.841729
34	6	0	0.700654	3.925566	-0.238559
35	6	0	0.056336	-3.061988	-1.181215
36	8	0	0.362155	4.198384	-1.350623
37	8	0	-4.970619	-0.557401	0.021770
38	8	0	-2.472496	-2.937729	0.098394
39	8	0	-0.267429	-2.938487	-2.326682
40	6	0	1.260991	4.874787	0.779681
41	6	0	0.336358	-4.353281	-0.475235
42	6	0	-3.105472	-2.047900	2.232695
43	6	0	-5.130915	0.850914	1.969773
44	1	0	1.364911	5.860518	0.334750
45	1	0	0.588626	4.914470	1.637830
46	1	0	2.225800	4.506258	1.129597
47	1	0	-0.562184	-4.613326	0.088142
48	1	0	0.539696	-5.126986	-1.211270
49	1	0	1.167751	-4.241642	0.218995
50	1	0	-2.933231	-3.035812	2.654112
51	1	0	-2.672014	-1.273604	2.862936
52	1	0	-4.182329	-1.886354	2.148283
53	1	0	-5.835855	0.117227	2.352962
54	1	0	-4.237300	0.881624	2.596619
55	1	0	-5.571607	1.847455	1.973008
56	1	0	7.989634	-0.744421	0.316252

Structure 38β (M06-2X/6-311G(d,p), $CHCl_3$)

Energy (Hartrees): = -1546.9508892
No imaginary frequencies

Standard orientation:

Center Number	Atomic Number	Atomic Type	Coordinates (Angstroms) X	Y	Z
1	6	0	0.101301	1.702310	-0.540042
2	6	0	0.565052	0.329376	-0.061564
3	6	0	-0.175711	-0.726428	-0.879384
4	6	0	-1.687757	-0.506920	-0.826464
5	6	0	-1.988244	0.927746	-1.247956

6	1	0	0.413962	1.875081	-1.576631
7	1	0	0.155314	-0.682724	-1.920218
8	1	0	-2.195488	-1.213175	-1.485405
9	1	0	-1.637631	1.025629	-2.287163
10	1	0	0.295593	0.224200	0.997621
11	8	0	-1.288835	1.841117	-0.418258
12	6	0	-3.449145	1.336101	-1.265601
13	1	0	-3.518758	2.332874	-1.699759
14	1	0	-4.026434	0.630444	-1.864123
15	8	0	-4.012945	1.456747	0.042374
16	8	0	-2.135272	-0.700216	0.520323
17	8	0	0.166628	-1.992814	-0.323262
18	8	0	0.684477	2.662261	0.304356
19	7	0	1.984652	0.203440	-0.304145
20	6	0	4.172849	-0.333668	0.537695
21	6	0	4.822997	-0.140526	-0.685392
22	6	0	4.914232	-0.699596	1.661861
23	6	0	6.195704	-0.313058	-0.776515
24	1	0	4.237433	0.141098	-1.552618
25	6	0	6.291558	-0.871303	1.569255
26	1	0	4.408023	-0.849835	2.610076
27	6	0	6.932742	-0.677715	0.350700
28	1	0	6.696796	-0.164218	-1.725976
29	1	0	6.862589	-1.154370	2.445786
30	6	0	2.714340	-0.155362	0.667134
31	1	0	2.294328	-0.350370	1.662365
32	6	0	-4.746974	0.438324	0.513171
33	6	0	-2.585868	-1.928674	0.846079
34	6	0	0.764714	3.929936	-0.179815
35	6	0	0.006625	-3.067364	-1.130946
36	8	0	0.388320	4.235125	-1.275133
37	8	0	-4.927321	-0.590451	-0.085724
38	8	0	-2.573220	-2.864399	0.091904
39	8	0	-0.324574	-2.973450	-2.280971
40	6	0	1.379466	4.841843	0.834718
41	6	0	0.285082	-4.340502	-0.396002
42	6	0	-3.077017	-1.984937	2.261475
43	6	0	-5.321795	0.775688	1.856871
44	1	0	1.470446	5.842169	0.418954
45	1	0	0.752500	4.859939	1.728189
46	1	0	2.359826	4.458423	1.122458
47	1	0	-0.593592	-4.572358	0.210810
48	1	0	0.452153	-5.143345	-1.110545
49	1	0	1.143324	-4.223718	0.264990
50	1	0	-4.033987	-2.506591	2.274476
51	1	0	-2.364232	-2.571334	2.846171
52	1	0	-3.171753	-0.994365	2.700982
53	1	0	-5.781758	-0.107056	2.294982
54	1	0	-4.543633	1.173009	2.509482
55	1	0	-6.076339	1.555402	1.727936
56	1	0	8.005800	-0.811884	0.275631

Structure 38β-a (B3LYP, Gas Phase)

Energy (Hartrees): = -1547.13862
No imaginary frequencies

Standard orientation:

Center Number	Atomic Number	Atomic Type	Coordinates (Angstroms) X	Y	Z
1	6	0	0.504523	-1.168271	-0.231228
2	6	0	-0.484118	-0.043052	0.122473
3	6	0	-0.067582	1.216933	-0.655697
4	6	0	1.420233	1.556422	-0.480199
5	6	0	2.251975	0.310371	-0.793671
6	1	0	0.390169	-1.477471	-1.278837
7	1	0	-0.259643	1.065457	-1.722207
8	1	0	1.680662	2.390101	-1.137174
9	1	0	2.089676	0.058526	-1.855011
10	1	0	-0.391823	0.154741	1.201374
11	8	0	1.830336	-0.773181	0.028021
12	6	0	3.748597	0.476413	-0.568016
13	1	0	4.125478	1.332853	-1.134153
14	1	0	3.970651	0.595817	0.492892
15	8	0	4.400059	-0.695075	-1.077473
16	8	0	1.683548	1.933537	0.888728
17	8	0	-0.881297	2.303682	-0.190925
18	8	0	0.232424	-2.265042	0.620985
19	7	0	-1.818766	-0.433536	-0.279724
20	6	0	-4.130951	-0.793946	0.316049
21	6	0	-4.534364	-1.176191	-0.974497
22	6	0	-5.077901	-0.766235	1.349944
23	6	0	-5.858611	-1.522614	-1.218250
24	1	0	-3.792974	-1.192367	-1.766126

25	6	0	-6.405568	-1.114046	1.103789
26	1	0	-4.769024	-0.470678	2.349770
27	6	0	-6.797393	-1.492783	-0.180689
28	1	0	-6.165037	-1.816749	-2.217787
29	1	0	-7.131505	-1.089636	1.910979
30	6	0	-2.737169	-0.422697	0.605123
31	1	0	-2.531355	-0.131191	1.647330
32	6	0	4.860080	-1.599422	-0.164055
33	6	0	1.709042	3.259192	1.187881
34	6	0	0.741010	-3.475870	0.234860
35	6	0	-1.155128	3.285394	-1.101448
36	8	0	1.341754	-3.653023	-0.798126
37	8	0	4.966569	-1.373644	1.018554
38	8	0	1.553292	4.143902	0.375532
39	8	0	-0.817420	3.230978	-2.261129
40	6	0	0.442578	-4.521034	1.278010
41	6	0	-1.886415	4.426478	-0.447229
42	6	0	1.962715	3.456622	2.661339
43	6	0	5.181164	-2.904105	-0.845393
44	1	0	0.750212	-5.499088	0.910171
45	1	0	0.988034	-4.283612	2.196635
46	1	0	-0.623474	-4.522849	1.520056
47	1	0	-1.150808	5.034158	0.090063
48	1	0	-2.364784	5.037674	-1.212114
49	1	0	-2.621658	4.063205	0.273827
50	1	0	2.063166	4.520421	2.872904
51	1	0	1.129596	3.040724	3.235945
52	1	0	2.866433	2.921971	2.965756
53	1	0	5.805853	-3.514695	-0.193776
54	1	0	4.236882	-3.425456	-1.038916
55	1	0	5.673089	-2.735565	-1.805889
56	1	0	-7.830882	-1.763996	-0.375563

Structure 38β-a (B3LYP, $CHCl_3$)

Energy (Hartrees): = -1547.1699376
No imaginary frequencies

Standard orientation:

Center Number	Atomic Number	Atomic Type	Coordinates (Angstroms) X	Y	Z
1	6	0	0.497680	-1.188315	-0.228053
2	6	0	-0.483772	-0.054853	0.116806
3	6	0	-0.057509	1.196013	-0.670948
4	6	0	1.429675	1.528674	-0.485251
5	6	0	2.262699	0.277969	-0.778551
6	1	0	0.388628	-1.495856	-1.275463
7	1	0	-0.242244	1.036278	-1.736824
8	1	0	1.708681	2.348791	-1.149993
9	1	0	2.117300	0.023504	-1.840223
10	1	0	-0.395880	0.152053	1.193585
11	8	0	1.827692	-0.806212	0.042726
12	6	0	3.750337	0.464745	-0.524161
13	1	0	4.120893	1.330332	-1.079501
14	1	0	3.955600	0.587204	0.539341
15	8	0	4.443716	-0.690882	-1.029619
16	8	0	1.677006	1.920063	0.883582
17	8	0	-0.876101	2.286326	-0.213198
18	8	0	0.207605	-2.281416	0.625661
19	7	0	-1.822175	-0.443715	-0.283142
20	6	0	-4.142294	-0.757452	0.317293
21	6	0	-4.547156	-1.213279	-0.949654
22	6	0	-5.094891	-0.645326	1.341652
23	6	0	-5.877069	-1.549491	-1.179979
24	1	0	-3.807020	-1.297235	-1.738612
25	6	0	-6.428347	-0.981570	1.108305
26	1	0	-4.784736	-0.294042	2.322678
27	6	0	-6.821114	-1.433971	-0.152559
28	1	0	-6.183574	-1.901535	-2.160767
29	1	0	-7.158262	-0.891545	1.907471
30	6	0	-2.744784	-0.392557	0.598544
31	1	0	-2.540305	-0.069435	1.629975
32	6	0	4.971269	-1.561174	-0.130649
33	6	0	1.741709	3.247076	1.167371
34	6	0	0.622252	-3.517270	0.210781
35	6	0	-1.163558	3.266095	-1.113492
36	8	0	1.182169	-3.712950	-0.844379
37	8	0	5.042330	-1.348223	1.061097
38	8	0	1.621901	4.122994	0.335979
39	8	0	-0.797907	3.238052	-2.269346
40	6	0	0.280436	-4.557174	1.239545
41	6	0	-1.961957	4.365010	-0.470582
42	6	0	1.989268	3.459327	2.635713
43	6	0	5.431572	-2.814676	-0.823435

44	1	0	0.569682	-5.542960	0.875904
45	1	0	0.808483	-4.337890	2.173128
46	1	0	-0.791907	-4.535982	1.455390
47	1	0	-1.290351	4.971999	0.146268
48	1	0	-2.404274	4.995798	-1.241865
49	1	0	-2.738616	3.955523	0.179674
50	1	0	2.065981	4.526093	2.844818
51	1	0	1.169404	3.024651	3.215967
52	1	0	2.909583	2.951079	2.939034
53	1	0	6.058557	-3.400840	-0.151322
54	1	0	4.549574	-3.403116	-1.100338
55	1	0	5.974825	-2.578382	-1.741873
56	1	0	-7.858773	-1.697314	-0.336681

Structure 38β-a (M06-2X/6-311G(d,p), Gas Phase)

Energy (Hartrees): = -1546.9167682
No imaginary frequencies

Standard orientation:

Center Number	Atomic Number	Atomic Type	Coordinates (Angstroms) X	Y	Z
1	6	0	0.562621	-1.154982	-0.281922
2	6	0	-0.455202	-0.079071	0.085886
3	6	0	-0.107239	1.185294	-0.699414
4	6	0	1.357385	1.585051	-0.517413
5	6	0	2.238083	0.383917	-0.832501
6	1	0	0.457223	-1.453486	-1.333030
7	1	0	-0.294138	1.015923	-1.763759
8	1	0	1.588809	2.436466	-1.161140
9	1	0	2.104224	0.124805	-1.893858
10	1	0	-0.356164	0.123967	1.161074
11	8	0	1.863121	-0.712146	-0.023361
12	6	0	3.714065	0.612742	-0.574179
13	1	0	4.078170	1.459739	-1.156726
14	1	0	3.890039	0.770523	0.489455
15	8	0	4.407637	-0.544587	-1.025620
16	8	0	1.589160	1.936483	0.851582
17	8	0	-0.962890	2.219408	-0.228338
18	8	0	0.340748	-2.261400	0.554846
19	7	0	-1.774538	-0.520520	-0.298906
20	6	0	-4.073528	-0.888947	0.301146
21	6	0	-4.452521	-1.332932	-0.968549
22	6	0	-5.021675	-0.827043	1.321179
23	6	0	-5.764826	-1.708226	-1.207120
24	1	0	-3.701740	-1.374219	-1.748327
25	6	0	-6.338232	-1.203550	1.080672
26	1	0	-4.724982	-0.482333	2.306526
27	6	0	-6.709794	-1.644168	-0.183721
28	1	0	-6.058133	-2.051386	-2.191950
29	1	0	-7.070900	-1.151829	1.876873
30	6	0	-2.684246	-0.482197	0.579563
31	1	0	-2.481398	-0.138286	1.603359
32	6	0	4.708149	-1.485756	-0.092410
33	6	0	1.455448	3.232080	1.202978
34	6	0	1.003984	-3.397449	0.214995
35	6	0	-1.139308	3.268418	-1.075656
36	8	0	1.650934	-3.505083	-0.785722
37	8	0	4.662234	-1.291908	1.086606
38	8	0	1.188704	4.110572	0.431189
39	8	0	-0.687883	3.289303	-2.183579
40	6	0	0.813530	-4.451358	1.265071
41	6	0	-1.931189	4.357632	-0.419496
42	6	0	1.685886	3.400995	2.677201
43	6	0	5.080255	-2.776800	-0.759691
44	1	0	1.180776	-5.403435	0.891592
45	1	0	1.378647	-4.157975	2.151988
46	1	0	-0.237884	-4.518753	1.542715
47	1	0	-1.246016	4.906543	0.230240
48	1	0	-2.323163	5.026393	-1.181313
49	1	0	-2.732749	3.939330	0.187332
50	1	0	1.707377	4.459907	2.919913
51	1	0	0.874635	2.909302	3.217142
52	1	0	2.617905	2.916239	2.967114
53	1	0	5.606178	-3.411872	-0.051370
54	1	0	4.151183	-3.261653	-1.071115
55	1	0	5.685089	-2.589823	-1.645823
56	1	0	-7.735221	-1.937495	-0.375096

Structure 38β-a (M06-2X/6-311G(d,p), $CHCl_3$)

Energy (Hartrees): = -1546.9503357
No imaginary frequencies

Standard orientation:

Center Number	Atomic Number	Atomic Type	Coordinates (Angstroms) X	Y	Z
1	6	0	0.563205	-1.164570	-0.296268
2	6	0	-0.445766	-0.080085	0.070399
3	6	0	-0.089047	1.180786	-0.717551
4	6	0	1.378629	1.568380	-0.535302
5	6	0	2.251936	0.359850	-0.840960
6	1	0	0.461442	-1.455957	-1.348732
7	1	0	-0.280657	1.014543	-1.780913
8	1	0	1.627864	2.407770	-1.186425
9	1	0	2.116424	0.098537	-1.900536
10	1	0	-0.347991	0.124836	1.144772
11	8	0	1.867453	-0.733938	-0.026844
12	6	0	3.725038	0.595113	-0.579495
13	1	0	4.092106	1.419714	-1.190556
14	1	0	3.903866	0.794478	0.476253
15	8	0	4.426200	-0.579390	-0.989017
16	8	0	1.603737	1.930595	0.832531
17	8	0	-0.937928	2.218820	-0.233910
18	8	0	0.320712	-2.271717	0.535950
19	7	0	-1.771226	-0.514248	-0.310037
20	6	0	-4.071920	-0.862079	0.304007
21	6	0	-4.463570	-1.336062	-0.952147
22	6	0	-5.016231	-0.754849	1.325593
23	6	0	-5.783323	-1.697273	-1.176063
24	1	0	-3.722246	-1.415398	-1.738453
25	6	0	-6.340220	-1.116448	1.099242
26	1	0	-4.708514	-0.387975	2.299602
27	6	0	-6.723995	-1.587028	-0.151626
28	1	0	-6.085775	-2.063853	-2.150080
29	1	0	-7.069700	-1.030045	1.895971
30	6	0	-2.677494	-0.464731	0.574048
31	1	0	-2.468867	-0.118438	1.594706
32	6	0	4.722995	-1.493031	-0.037669
33	6	0	1.500044	3.232601	1.169322
34	6	0	0.929071	-3.432991	0.180797
35	6	0	-1.129533	3.276320	-1.058288
36	8	0	1.589815	-3.545023	-0.813532
37	8	0	4.652456	-1.276916	1.140396
38	8	0	1.264439	4.106250	0.377647
39	8	0	-0.681682	3.322934	-2.170817
40	6	0	0.658731	-4.502043	1.191290
41	6	0	-1.941197	4.338078	-0.386335
42	6	0	1.719633	3.417723	2.639073
43	6	0	5.136546	-2.789110	-0.665181
44	1	0	1.021297	-5.456836	0.818544
45	1	0	1.177579	-4.243875	2.117318
46	1	0	-0.409239	-4.550505	1.405893
47	1	0	-1.297977	4.840111	0.340361
48	1	0	-2.286479	5.056535	-1.125946
49	1	0	-2.783043	3.894470	0.145189
50	1	0	1.734198	4.478822	2.875173
51	1	0	0.909816	2.925022	3.181525
52	1	0	2.656077	2.944637	2.937416
53	1	0	5.604468	-3.426179	0.081760
54	1	0	4.235068	-3.274520	-1.049039
55	1	0	5.811181	-2.611427	-1.502516
56	1	0	-7.755114	-1.868954	-0.331576

Structure 38β-b (B3LYP, Gas Phase)

Energy (Hartrees): = -1547.1387738
No imaginary frequencies

Standard orientation:

Center Number	Atomic Number	Atomic Type	Coordinates (Angstroms) X	Y	Z
1	6	0	0.104242	1.706900	0.083991
2	6	0	0.634842	0.281392	0.321305
3	6	0	-0.326847	-0.701542	-0.363279
4	6	0	-1.783113	-0.471433	0.058597
5	6	0	-2.141848	1.002414	-0.152874
6	1	0	0.177895	1.981341	-0.975471
7	1	0	-0.259718	-0.583620	-1.449012
8	1	0	-2.441067	-1.123107	-0.518583
9	1	0	-2.105958	1.208578	-1.233573
10	1	0	0.626398	0.095060	1.406279
11	8	0	-1.224549	1.844173	0.542528
12	6	0	-3.520831	1.388844	0.381094
13	1	0	-3.539606	1.267614	1.464140

14	1	0	-3.735130	2.423758	0.110171
15	8	0	-4.543995	0.515875	-0.128522
16	8	0	-1.940560	-0.771162	1.463645
17	8	0	0.088662	-2.031491	-0.017071
18	8	0	0.888379	2.590813	0.860905
19	7	0	1.949945	0.162434	-0.271848
20	6	0	4.286472	-0.392072	-0.016975
21	6	0	4.625046	-0.128144	-1.355006
22	6	0	5.283855	-0.821122	0.870404
23	6	0	5.935897	-0.291679	-1.788742
24	1	0	3.844502	0.202242	-2.031946
25	6	0	6.597893	-0.984724	0.433763
26	1	0	5.025065	-1.026773	1.906325
27	6	0	6.925420	-0.720139	-0.896541
28	1	0	6.191916	-0.086451	-2.824034
29	1	0	7.363216	-1.317584	1.128504
30	6	0	2.908749	-0.228269	0.472472
31	1	0	2.753419	-0.467046	1.536753
32	6	0	-5.054757	0.824530	-1.352943
33	6	0	-2.409326	-2.001324	1.798797
34	6	0	1.042824	3.867554	0.384382
35	6	0	-0.182985	-3.006638	-0.933916
36	8	0	0.589714	4.253689	-0.664416
37	8	0	-4.676915	1.762720	-2.017018
38	8	0	-2.709045	-2.860063	0.999435
39	8	0	-0.667688	-2.785080	-2.019320
40	6	0	1.855753	4.684066	1.355574
41	6	0	0.170231	-4.362939	-0.384997
42	6	0	-2.498232	-2.135953	3.299077
43	6	0	-6.133125	-0.156365	-1.738591
44	1	0	2.012048	5.682013	0.948100
45	1	0	1.330498	4.749633	2.313048
46	1	0	2.817039	4.197154	1.542188
47	1	0	-0.628161	-4.667614	0.299578
48	1	0	0.234904	-5.080382	-1.202615
49	1	0	1.106888	-4.330053	0.175583
50	1	0	-2.937668	-3.100299	3.551086
51	1	0	-1.498073	-2.058370	3.735959
52	1	0	-3.100915	-1.325642	3.718038
53	1	0	-6.526714	0.106115	-2.719662
54	1	0	-5.725644	-1.171312	-1.757286
55	1	0	-6.935757	-0.141346	-0.995659
56	1	0	7.948031	-0.846869	-1.239857

Structure 38β-b (B3LYP, $CHCl_3$)

Energy (Hartrees): = -1547.16968
No imaginary frequencies

Standard orientation:

Center Number	Atomic Number	Atomic Type	Coordinates (Angstroms) X	Y	Z
1	6	0	0.100204	1.706721	0.042416
2	6	0	0.639454	0.286791	0.289227
3	6	0	-0.318119	-0.706624	-0.387284
4	6	0	-1.775723	-0.483746	0.034027
5	6	0	-2.147022	0.986881	-0.176078
6	1	0	0.166436	1.974342	-1.018804
7	1	0	-0.251171	-0.598994	-1.473258
8	1	0	-2.432725	-1.133318	-0.546009
9	1	0	-2.119612	1.189209	-1.256678
10	1	0	0.634325	0.108369	1.374785
11	8	0	-1.226494	1.838965	0.509311
12	6	0	-3.519916	1.366519	0.378651
13	1	0	-3.523761	1.254823	1.463259
14	1	0	-3.751045	2.398453	0.109589
15	8	0	-4.544540	0.480320	-0.107317
16	8	0	-1.925769	-0.790082	1.437978
17	8	0	0.110229	-2.030572	-0.020908
18	8	0	0.883782	2.598943	0.814980
19	7	0	1.958878	0.169473	-0.300261
20	6	0	4.301548	-0.355545	-0.010431
21	6	0	4.664292	-0.103723	-1.345318
22	6	0	5.284278	-0.776467	0.898779
23	6	0	5.982939	-0.271522	-1.755180
24	1	0	3.901238	0.221851	-2.044735
25	6	0	6.605697	-0.945009	0.485643
26	1	0	5.006816	-0.971655	1.931728
27	6	0	6.956706	-0.692969	-0.841783
28	1	0	6.256918	-0.075012	-2.787751
29	1	0	7.358598	-1.272260	1.196648
30	6	0	2.917380	-0.193740	0.461565
31	1	0	2.755822	-0.410918	1.527905
32	6	0	-5.107287	0.782057	-1.306769

33	6	0	-2.433646	-2.003879	1.773986
34	6	0	0.984774	3.890111	0.372093
35	6	0	-0.102758	-3.025012	-0.925153
36	8	0	0.475652	4.286684	-0.650937
37	8	0	-4.775876	1.737058	-1.977718
38	8	0	-2.781454	-2.840524	0.966843
39	8	0	-0.587913	-2.839216	-2.020789
40	6	0	1.803983	4.707482	1.330308
41	6	0	0.320046	-4.353732	-0.364266
42	6	0	-2.499340	-2.150886	3.269844
43	6	0	-6.176670	-0.215391	-1.659153
44	1	0	1.937742	5.713367	0.932926
45	1	0	1.294721	4.757720	2.298197
46	1	0	2.776823	4.234770	1.493959
47	1	0	-0.424320	-4.675315	0.372132
48	1	0	0.370800	-5.091100	-1.165561
49	1	0	1.284095	-4.274743	0.144015
50	1	0	-2.952908	-3.108264	3.525646
51	1	0	-1.490584	-2.095436	3.691221
52	1	0	-3.081730	-1.333391	3.704907
53	1	0	-6.600155	0.029169	-2.633030
54	1	0	-5.756533	-1.225478	-1.677563
55	1	0	-6.963100	-0.201883	-0.897978
56	1	0	7.985289	-0.823108	-1.166016

Structure 38β-b (M06-2X/6-311G(d,p), Gas Phase)

Energy (Hartrees): = -1546.918867
No imaginary frequencies

Standard orientation:

Center Number	Atomic Number	Atomic Type	Coordinates (Angstroms) X	Y	Z
1	6	0	0.306767	1.798008	-0.121188
2	6	0	0.681896	0.363153	0.246419
3	6	0	-0.314880	-0.555677	-0.450092
4	6	0	-1.752356	-0.159670	-0.141857
5	6	0	-1.974766	1.324062	-0.403208
6	1	0	0.423294	1.965241	-1.198825
7	1	0	-0.157061	-0.510995	-1.531325
8	1	0	-2.420436	-0.774143	-0.745751
9	1	0	-1.910410	1.502920	-1.483197
10	1	0	0.587133	0.251056	1.335380
11	8	0	-1.002892	2.095308	0.284191
12	6	0	-3.322065	1.796561	0.150942
13	1	0	-3.182086	2.043901	1.200493
14	1	0	-3.676216	2.666372	-0.402623
15	8	0	-4.315177	0.768072	0.137304
16	8	0	-2.012137	-0.382034	1.249242
17	8	0	-0.086438	-1.884801	0.009417
18	8	0	1.158610	2.655253	0.590915
19	7	0	2.008361	0.085592	-0.249758
20	6	0	4.236922	-0.711693	0.163334
21	6	0	4.665676	-0.522967	-1.153175
22	6	0	5.125870	-1.214015	1.112683
23	6	0	5.968370	-0.834106	-1.508735
24	1	0	3.960317	-0.131920	-1.876333
25	6	0	6.433040	-1.525729	0.754808
26	1	0	4.790765	-1.359896	2.134568
27	6	0	6.854137	-1.335738	-0.555584
28	1	0	6.299496	-0.688089	-2.529866
29	1	0	7.120085	-1.916233	1.495826
30	6	0	2.856054	-0.383419	0.563338
31	1	0	2.603444	-0.568966	1.616585
32	6	0	-4.735134	0.367121	-1.083004
33	6	0	-2.727140	-1.471297	1.577523
34	6	0	1.304399	3.911719	0.085853
35	6	0	-0.561148	-2.873428	-0.792884
36	8	0	0.782650	4.278798	-0.923008
37	8	0	-4.334725	0.863904	-2.098333
38	8	0	-3.049415	-2.323499	0.793524
39	8	0	-1.030963	-2.665894	-1.873711
40	6	0	2.200943	4.725848	0.972188
41	6	0	-0.445998	-4.208529	-0.121610
42	6	0	-3.087428	-1.449219	3.034273
43	6	0	-5.694077	-0.780606	-0.971465
44	1	0	2.346008	5.708200	0.531202
45	1	0	1.743189	4.816718	1.958233
46	1	0	3.155962	4.213792	1.093998
47	1	0	-1.289359	-4.292583	0.567972
48	1	0	-0.512600	-4.994031	-0.870010
49	1	0	0.481080	-4.279721	0.444737
50	1	0	-3.477527	-2.419725	3.328833

51	1	0	-2.220514	-1.176508	3.634905
52	1	0	-3.853308	-0.683953	3.179136
53	1	0	-6.153220	-0.961705	-1.939510
54	1	0	-5.130750	-1.662032	-0.653111
55	1	0	-6.448352	-0.571165	-0.213506
56	1	0	7.871951	-1.578798	-0.837199

Structure 38β-b (M06-2X/6-311G(d,p), $CHCl_3$)

Energy (Hartrees): = -1546.9516513
No imaginary frequencies

Standard orientation:

Center Number	Atomic Number	Atomic Type	Coordinates (Angstroms) X	Y	Z
1	6	0	0.227992	1.774781	-0.077661
2	6	0	0.657860	0.346025	0.246735
3	6	0	-0.328144	-0.594041	-0.439848
4	6	0	-1.770123	-0.260752	-0.072042
5	6	0	-2.040561	1.219228	-0.309278
6	1	0	0.310549	1.973292	-1.152680
7	1	0	-0.212004	-0.515313	-1.524030
8	1	0	-2.448273	-0.878805	-0.661154
9	1	0	-1.992805	1.409644	-1.388022
10	1	0	0.600789	0.209379	1.335174
11	8	0	-1.080153	2.015376	0.369947
12	6	0	-3.386959	1.654793	0.262420
13	1	0	-3.284257	1.772996	1.339084
14	1	0	-3.703175	2.595618	-0.189259
15	8	0	-4.394343	0.655329	0.086910
16	8	0	-1.973769	-0.515244	1.322314
17	8	0	-0.011999	-1.920285	-0.020020
18	8	0	1.070495	2.640874	0.639308
19	7	0	1.982166	0.122368	-0.287960
20	6	0	4.252682	-0.584217	0.078060
21	6	0	4.658474	-0.373557	-1.243431
22	6	0	5.173930	-1.053397	1.015390
23	6	0	5.968900	-0.630659	-1.616586
24	1	0	3.935391	-0.007350	-1.962606
25	6	0	6.488355	-1.311061	0.639633
26	1	0	4.857319	-1.215515	2.040960
27	6	0	6.886146	-1.099761	-0.675767
28	1	0	6.280419	-0.466811	-2.641679
29	1	0	7.199223	-1.677060	1.371224
30	6	0	2.867155	-0.314486	0.506167
31	1	0	2.646724	-0.512988	1.562941
32	6	0	-4.871313	0.489387	-1.162570
33	6	0	-2.532565	-1.690590	1.670658
34	6	0	1.139667	3.925024	0.197265
35	6	0	-0.438464	-2.922366	-0.824449
36	8	0	0.537546	4.317562	-0.760446
37	8	0	-4.482316	1.136885	-2.097795
38	8	0	-2.834570	-2.544002	0.878917
39	8	0	-0.987256	-2.725826	-1.873769
40	6	0	2.062970	4.727973	1.058544
41	6	0	-0.143356	-4.258876	-0.220043
42	6	0	-2.719826	-1.768270	3.154738
43	6	0	-5.913737	-0.584944	-1.202338
44	1	0	2.083270	5.757325	0.709922
45	1	0	1.724788	4.683919	2.095011
46	1	0	3.063603	4.293836	1.013093
47	1	0	-0.851317	-4.423259	0.595818
48	1	0	-0.270125	-5.033870	-0.972331
49	1	0	0.866442	-4.277837	0.189545
50	1	0	-3.174921	-2.719918	3.417572
51	1	0	-1.751582	-1.662430	3.647684
52	1	0	-3.354594	-0.942622	3.481369
53	1	0	-6.282335	-0.700111	-2.218454
54	1	0	-5.479059	-1.522165	-0.849098
55	1	0	-6.732632	-0.322686	-0.530291
56	1	0	7.909811	-1.300601	-0.970590

Structure 56a (B3LYP, Gas Phase)

Energy (Hartrees): = -936.4649509
No imaginary frequencies

Standard orientation:

Center Number	Atomic Number	Atomic Type	Coordinates (Angstroms) X	Y	Z
1	6	0	2.540728	0.398080	-0.131343

2	8	0	2.144629	1.442044	0.766045
3	6	0	0.834425	1.868811	0.427301
4	6	0	0.182213	0.724121	-0.381182
5	6	0	1.241388	-0.397821	-0.301724
6	1	0	2.849953	0.813572	-1.102089
7	1	0	0.313341	2.063990	1.372641
8	1	0	0.115054	1.052064	-1.430141
9	1	0	1.056062	-0.986792	0.608858
10	7	0	-1.091887	0.305083	0.159465
11	6	0	-3.455440	-0.071512	-0.181861
12	6	0	-3.699873	-0.531663	1.123234
13	6	0	-4.517244	-0.010435	-1.095873
14	6	0	-4.980719	-0.920957	1.498800
15	1	0	-2.871039	-0.572334	1.821948
16	6	0	-5.801034	-0.402003	-0.717857
17	1	0	-4.332668	0.346966	-2.106077
18	6	0	-6.034515	-0.857903	0.579983
19	1	0	-5.163648	-1.274404	2.509380
20	1	0	-6.616369	-0.350650	-1.433236
21	1	0	-7.033644	-1.162709	0.877436
22	6	0	-2.110743	0.345914	-0.609614
23	1	0	-2.034342	0.704064	-1.649222
24	8	0	1.293970	-1.225166	-1.451348
25	1	0	0.491666	-1.763742	-1.461220
26	6	0	3.715341	-0.350504	0.484140
27	1	0	3.427724	-0.650389	1.506234
28	6	0	4.096685	-1.608106	-0.300670
29	1	0	3.290975	-2.351130	-0.278072
30	1	0	4.286620	-1.340734	-1.349074
31	8	0	4.823634	0.533195	0.517406
32	1	0	5.587387	-0.035992	0.700114
33	8	0	5.284203	-2.102388	0.329097
34	1	0	5.713665	-2.720125	-0.274139
35	8	0	0.847201	3.012695	-0.394701
36	1	0	1.346102	3.693587	0.078408

Structure 56a (B3LYP, DMSO)

Energy (Hartrees): = -936.4909487
No imaginary frequencies

Standard orientation:

Center Number	Atomic Number	Atomic Type	Coordinates (Angstroms) X	Y	Z
1	6	0	2.546676	0.390002	-0.163842
2	8	0	2.180630	1.488679	0.683404
3	6	0	0.844331	1.890086	0.392099
4	6	0	0.187926	0.730831	-0.384607
5	6	0	1.237684	-0.399498	-0.288093
6	1	0	2.853907	0.749993	-1.157672
7	1	0	0.356519	2.074596	1.356931
8	1	0	0.113799	1.027987	-1.441414
9	1	0	1.064918	-0.966898	0.637512
10	7	0	-1.090013	0.340700	0.174468
11	6	0	-3.444338	-0.093510	-0.176069
12	6	0	-3.715324	-0.467535	1.152368
13	6	0	-4.487984	-0.098566	-1.114732
14	6	0	-5.003277	-0.837201	1.527342
15	1	0	-2.907668	-0.462666	1.877482
16	6	0	-5.778531	-0.470233	-0.737072
17	1	0	-4.281643	0.189920	-2.142316
18	6	0	-6.038230	-0.839810	0.584020
19	1	0	-5.205826	-1.124315	2.555211
20	1	0	-6.578680	-0.470984	-1.471486
21	1	0	-7.042357	-1.129091	0.880626
22	6	0	-2.097003	0.306524	-0.613221
23	1	0	-2.008764	0.580949	-1.674321
24	8	0	1.263417	-1.253401	-1.419060
25	1	0	0.495463	-1.840922	-1.358001
26	6	0	3.713692	-0.346799	0.480210
27	1	0	3.420312	-0.609361	1.510457
28	6	0	4.092763	-1.631529	-0.257668
29	1	0	3.289589	-2.374566	-0.199934
30	1	0	4.283817	-1.410957	-1.315782
31	8	0	4.839249	0.526223	0.495670
32	1	0	5.595338	-0.069032	0.631208
33	8	0	5.284656	-2.105745	0.384802
34	1	0	5.743324	-2.689624	-0.235140
35	8	0	0.798104	3.040504	-0.417760
36	1	0	1.159293	3.773413	0.104513

Structure 56a (M06-2X/6-311G(d,p), Gas Phase)

Energy (Hartrees): = -936.3323844
No imaginary frequencies

Standard orientation:

Center Number	Atomic Number	Atomic Type	Coordinates (Angstroms) X	Y	Z
1	6	0	2.524821	0.403146	-0.117925
2	8	0	2.158728	1.457394	0.763311
3	6	0	0.873038	1.910109	0.406478
4	6	0	0.195990	0.763686	-0.363823
5	6	0	1.219524	-0.373273	-0.248748
6	1	0	2.822129	0.797235	-1.099406
7	1	0	0.350112	2.157446	1.334036
8	1	0	0.138204	1.057724	-1.420976
9	1	0	1.031770	-0.926297	0.681824
10	7	0	-1.084710	0.383994	0.180951
11	6	0	-3.421214	-0.067866	-0.187475
12	6	0	-3.684317	-0.412182	1.141320
13	6	0	-4.449838	-0.113402	-1.127420
14	6	0	-4.961391	-0.797223	1.516939
15	1	0	-2.873407	-0.367149	1.858050
16	6	0	-5.730346	-0.501611	-0.749711
17	1	0	-4.244721	0.156579	-2.158479
18	6	0	-5.986172	-0.843595	0.572537
19	1	0	-5.164548	-1.061501	2.547921
20	1	0	-6.525572	-0.536685	-1.484393
21	1	0	-6.983182	-1.145440	0.870597
22	6	0	-2.070273	0.341385	-0.614108
23	1	0	-1.964792	0.613564	-1.674465
24	8	0	1.241195	-1.230998	-1.366155
25	1	0	0.405894	-1.705800	-1.391104
26	6	0	3.687189	-0.355402	0.481785
27	1	0	3.446343	-0.573048	1.532688
28	6	0	3.954456	-1.667414	-0.242228
29	1	0	3.121077	-2.363949	-0.113729
30	1	0	4.087561	-1.469770	-1.312201
31	8	0	4.824813	0.472181	0.394345
32	1	0	5.576661	-0.097914	0.588707
33	8	0	5.150342	-2.175210	0.339047
34	1	0	5.474360	-2.898088	-0.200155
35	8	0	0.938781	3.011160	-0.456677
36	1	0	1.475432	3.680775	-0.022264

Structure 56a (M06-2X/6-311G(d,p), DMSO)

Energy (Hartrees): = -936.3610782
No imaginary frequencies

Standard orientation:

Center Number	Atomic Number	Atomic Type	Coordinates (Angstroms) X	Y	Z
1	6	0	2.533907	0.392163	-0.171447
2	8	0	2.233258	1.535250	0.624631
3	6	0	0.900480	1.947192	0.376566
4	6	0	0.209624	0.788230	-0.350123
5	6	0	1.210325	-0.363295	-0.200827
6	1	0	2.809391	0.687452	-1.193494
7	1	0	0.444041	2.157270	1.348022
8	1	0	0.152694	1.040094	-1.417551
9	1	0	1.051645	-0.862863	0.764147
10	7	0	-1.081312	0.454244	0.205998
11	6	0	-3.397959	-0.085156	-0.181942
12	6	0	-3.700850	-0.327180	1.162162
13	6	0	-4.395806	-0.223279	-1.148372
14	6	0	-4.985095	-0.702450	1.528245
15	1	0	-2.922071	-0.218539	1.908059
16	6	0	-5.683417	-0.600199	-0.779786
17	1	0	-4.158289	-0.034573	-2.190546
18	6	0	-5.978746	-0.839838	0.558094
19	1	0	-5.216432	-0.890393	2.570440
20	1	0	-6.453339	-0.707416	-1.535082
21	1	0	-6.980957	-1.134064	0.848097
22	6	0	-2.041972	0.307945	-0.610207
23	1	0	-1.910242	0.466360	-1.688225
24	8	0	1.172218	-1.279165	-1.269825
25	1	0	0.366503	-1.801324	-1.184081
26	6	0	3.691391	-0.350518	0.458167
27	1	0	3.465482	-0.492011	1.524834
28	6	0	3.918097	-1.713154	-0.177771
29	1	0	3.079126	-2.382855	0.029734

30	1	0	4.026651	-1.601857	-1.262569
31	8	0	4.855555	0.441307	0.304168
32	1	0	5.592271	-0.160611	0.465723
33	8	0	5.123091	-2.204651	0.403348
34	1	0	5.429280	-2.950014	-0.121625
35	8	0	0.850748	3.071418	-0.452225
36	1	0	1.234596	3.809597	0.034806

Structure 56b (B3LYP, Gas Phase)

Energy (Hartrees): = -936.4702766
No imaginary frequencies

Standard orientation:

Center Number	Atomic Number	Atomic Type	Coordinates (Angstroms) X	Y	Z
1	6	0	-2.620722	0.645217	0.234727
2	8	0	-2.296838	1.130993	-1.070035
3	6	0	-0.982715	1.686052	-1.005700
4	6	0	-0.262520	1.025587	0.196101
5	6	0	-1.304885	0.025135	0.732139
6	1	0	-2.877459	1.474626	0.910659
7	1	0	-0.500629	1.449155	-1.961268
8	1	0	-0.103482	1.795121	0.965938
9	1	0	-1.134420	-0.940265	0.232293
10	7	0	0.963677	0.369734	-0.209856
11	6	0	3.322058	0.001515	0.157438
12	6	0	3.469114	-0.922506	-0.890712
13	6	0	4.440479	0.338117	0.933034
14	6	0	4.709940	-1.492732	-1.152324
15	1	0	2.596079	-1.175596	-1.482755
16	6	0	5.684465	-0.234666	0.669858
17	1	0	4.330869	1.051543	1.746160
18	6	0	5.821225	-1.150906	-0.373322
19	1	0	4.816625	-2.206408	-1.964276
20	1	0	6.544001	0.033026	1.277229
21	1	0	6.788876	-1.598705	-0.580546
22	6	0	2.021385	0.622802	0.457215
23	1	0	2.016495	1.328550	1.302627
24	8	0	-1.224167	-0.097079	2.133688
25	1	0	-2.004874	-0.615393	2.387156
26	6	0	-3.811328	-0.309501	0.158209
27	1	0	-4.732317	0.267712	-0.017401
28	6	0	-3.712717	-1.356579	-0.948574
29	1	0	-3.762315	-0.871017	-1.929940
30	1	0	-2.763559	-1.903515	-0.875766
31	8	0	-3.880907	-0.956569	1.431014
32	1	0	-4.469462	-1.716162	1.296793
33	8	0	-4.823026	-2.234490	-0.729399
34	1	0	-4.698905	-3.027059	-1.264778
35	8	0	-1.017195	3.072831	-0.774063
36	1	0	-1.496880	3.475551	-1.511304

Structure 56b (B3LYP, DMSO)

Energy (Hartrees): = -936.4936868
No imaginary frequencies

Standard orientation:

Center Number	Atomic Number	Atomic Type	Coordinates (Angstroms) X	Y	Z
1	6	0	-2.609242	0.628644	0.330905
2	8	0	-2.306760	1.315036	-0.886613
3	6	0	-0.929158	1.708029	-0.846195
4	6	0	-0.252697	0.879076	0.272849
5	6	0	-1.325968	-0.159704	0.630815
6	1	0	-2.767793	1.342674	1.153973
7	1	0	-0.514245	1.482565	-1.835472
8	1	0	-0.110632	1.536826	1.143477
9	1	0	-1.235751	-1.004242	-0.068314
10	7	0	0.987390	0.278099	-0.177836
11	6	0	3.361930	-0.038909	0.157990
12	6	0	3.551588	-0.864035	-0.964826
13	6	0	4.462461	0.270565	0.972527
14	6	0	4.815414	-1.364746	-1.261659
15	1	0	2.700421	-1.102022	-1.594771
16	6	0	5.728662	-0.233796	0.674391
17	1	0	4.319801	0.907605	1.841761
18	6	0	5.907429	-1.051557	-0.443046
19	1	0	4.954354	-2.000107	-2.131679
20	1	0	6.573226	0.011153	1.311813

21	1	0	6.892560	-1.444189	-0.678158
22	6	0	2.042473	0.515659	0.503309
23	1	0	2.021531	1.158067	1.395365
24	8	0	-1.219319	-0.590327	1.973025
25	1	0	-2.054616	-1.059728	2.140585
26	6	0	-3.880619	-0.205186	0.176698
27	1	0	-4.748632	0.469367	0.121246
28	6	0	-3.918724	-1.109558	-1.051486
29	1	0	-3.966832	-0.507065	-1.966144
30	1	0	-3.025444	-1.743933	-1.098776
31	8	0	-3.969754	-1.005928	1.364000
32	1	0	-4.579709	-1.725604	1.128180
33	8	0	-5.097623	-1.911496	-0.890494
34	1	0	-5.001118	-2.698279	-1.444955
35	8	0	-0.777786	3.068412	-0.535054
36	1	0	-1.120196	3.577752	-1.285825

Structure 56b (M06-2X/6-311G(d,p), Gas Phase)

Energy (Hartrees): = -936.3387615
No imaginary frequencies

Standard orientation:

Center Number	Atomic Number	Atomic Type	Coordinates (Angstroms) X	Y	Z
1	6	0	-2.603587	0.596355	0.301386
2	8	0	-2.348707	1.147242	-0.982733
3	6	0	-1.089849	1.796045	-0.914977
4	6	0	-0.290571	1.110921	0.206882
5	6	0	-1.246286	0.028674	0.718168
6	1	0	-2.878824	1.380975	1.018241
7	1	0	-0.619762	1.677214	-1.893728
8	1	0	-0.139254	1.835261	1.016972
9	1	0	-1.044977	-0.899330	0.167822
10	7	0	0.947929	0.537180	-0.270827
11	6	0	3.255234	0.012321	0.156362
12	6	0	3.451005	-0.636265	-1.065954
13	6	0	4.309064	0.098455	1.064812
14	6	0	4.685551	-1.187380	-1.369433
15	1	0	2.620217	-0.694276	-1.758510
16	6	0	5.547038	-0.456857	0.760588
17	1	0	4.155651	0.599968	2.014873
18	6	0	5.736076	-1.099892	-0.456579
19	1	0	4.835138	-1.687922	-2.318775
20	1	0	6.361147	-0.387458	1.471930
21	1	0	6.699881	-1.533258	-0.696262
22	6	0	1.948974	0.605513	0.501201
23	1	0	1.890891	1.110168	1.475279
24	8	0	-1.093062	-0.150462	2.099473
25	1	0	-1.790366	-0.763167	2.361648
26	6	0	-3.717750	-0.434186	0.211092
27	1	0	-4.693830	0.066431	0.184707
28	6	0	-3.617979	-1.318600	-1.019751
29	1	0	-3.800469	-0.723341	-1.917647
30	1	0	-2.622633	-1.771584	-1.083441
31	8	0	-3.615946	-1.227368	1.385281
32	1	0	-4.170737	-2.000850	1.232204
33	8	0	-4.615582	-2.317043	-0.831181
34	1	0	-4.533977	-2.976664	-1.521898
35	8	0	-1.241015	3.142879	-0.568281
36	1	0	-1.790611	3.554974	-1.240881

Structure 56b (M06-2X/6-311G(d,p), DMSO)

Energy (Hartrees): = -936.3643742
No imaginary frequencies

Standard orientation:

Center Number	Atomic Number	Atomic Type	Coordinates (Angstroms) X	Y	Z
1	6	0	-2.598268	0.627551	0.338823
2	8	0	-2.308257	1.265201	-0.897014
3	6	0	-0.950338	1.681606	-0.868771
4	6	0	-0.262175	0.888776	0.257088
5	6	0	-1.314785	-0.144139	0.648602
6	1	0	-2.753693	1.367802	1.135743
7	1	0	-0.530050	1.454825	-1.851978
8	1	0	-0.115626	1.564861	1.109901
9	1	0	-1.227761	-1.009239	-0.020959
10	7	0	0.975245	0.284718	-0.185770
11	6	0	3.338911	-0.031419	0.153718

12	6	0	3.511545	-0.895348	-0.932626
13	6	0	4.439877	0.315864	0.938738
14	6	0	4.769899	-1.399813	-1.226342
15	1	0	2.652440	-1.161909	-1.537423
16	6	0	5.701448	-0.190582	0.642944
17	1	0	4.303418	0.986746	1.780940
18	6	0	5.867466	-1.048050	-0.439331
19	1	0	4.900295	-2.069877	-2.068266
20	1	0	6.552628	0.083575	1.255405
21	1	0	6.849443	-1.443880	-0.672115
22	6	0	2.017308	0.531157	0.492985
23	1	0	1.991414	1.185188	1.373379
24	8	0	-1.180875	-0.520914	1.995158
25	1	0	-1.986000	-1.013562	2.200140
26	6	0	-3.848047	-0.226625	0.206932
27	1	0	-4.735113	0.418616	0.188443
28	6	0	-3.874913	-1.095232	-1.037063
29	1	0	-3.937261	-0.469619	-1.931080
30	1	0	-2.975035	-1.716201	-1.093259
31	8	0	-3.878823	-1.050857	1.369316
32	1	0	-4.509577	-1.755630	1.174291
33	8	0	-5.034843	-1.910742	-0.888428
34	1	0	-4.993815	-2.618508	-1.538383
35	8	0	-0.834500	3.040673	-0.572050
36	1	0	-1.201616	3.534434	-1.314412

Structure 56c (B3LYP, Gas Phase)

Energy (Hartrees): = -936.4710438
No imaginary frequencies

Standard orientation:

Center Number	Atomic Number	Atomic Type	Coordinates (Angstroms) X	Y	Z
1	6	0	2.568902	0.114705	-0.014062
2	8	0	2.231671	1.413627	0.511856
3	6	0	0.911484	1.771517	0.100227
4	6	0	0.241352	0.474574	-0.398590
5	6	0	1.230708	-0.623844	0.048343
6	1	0	2.911191	0.191402	-1.054423
7	1	0	0.408670	2.186129	0.982467
8	1	0	0.245105	0.502633	-1.499636
9	1	0	1.009207	-0.883557	1.094759
10	7	0	-1.078875	0.278323	0.156212
11	6	0	-3.439955	-0.082055	-0.217356
12	6	0	-3.771733	-0.166261	1.145594
13	6	0	-4.452766	-0.216082	-1.178077
14	6	0	-5.090101	-0.379560	1.532165
15	1	0	-2.979728	-0.060142	1.879272
16	6	0	-5.774437	-0.430057	-0.788639
17	1	0	-4.200285	-0.150384	-2.233617
18	6	0	-6.094806	-0.512094	0.566877
19	1	0	-5.340658	-0.443176	2.587048
20	1	0	-6.551466	-0.531953	-1.540245
21	1	0	-7.123500	-0.678525	0.872930
22	6	0	-2.054553	0.143796	-0.657581
23	1	0	-1.905867	0.194250	-1.748228
24	8	0	1.238930	-1.770226	-0.778383
25	1	0	0.415151	-2.251156	-0.623556
26	6	0	3.698294	-0.443492	0.851741
27	1	0	3.283646	-0.729439	1.832835
28	6	0	4.387020	-1.651379	0.211957
29	1	0	5.080840	-2.084280	0.949858
30	1	0	3.645175	-2.408916	-0.054849
31	8	0	4.717582	0.542426	1.014057
32	1	0	4.261115	1.387345	1.139334
33	8	0	5.062725	-1.277399	-0.975634
34	1	0	5.557150	-0.477822	-0.737685
35	8	0	0.918610	2.689513	-0.961185
36	1	0	1.323790	3.507198	-0.641020

Structure 56c (B3LYP, DMSO)

Energy (Hartrees): = -936.4947884
No imaginary frequencies

Standard orientation:

Center Number	Atomic Number	Atomic Type	Coordinates (Angstroms) X	Y	Z
1	6	0	2.571323	0.124988	-0.029382
2	8	0	2.256232	1.444749	0.450751

3	6	0	0.925282	1.803710	0.058946
4	6	0	0.247001	0.504258	-0.416779
5	6	0	1.225502	-0.599184	0.045146
6	1	0	2.919043	0.164917	-1.070818
7	1	0	0.440723	2.223701	0.948138
8	1	0	0.243091	0.508152	-1.517376
9	1	0	1.004944	-0.851401	1.092318
10	7	0	-1.075553	0.333436	0.146533
11	6	0	-3.427864	-0.098792	-0.209309
12	6	0	-3.772385	-0.095733	1.154122
13	6	0	-4.430559	-0.312332	-1.168127
14	6	0	-5.092107	-0.301055	1.543844
15	1	0	-2.995391	0.068564	1.893910
16	6	0	-5.753272	-0.518649	-0.775289
17	1	0	-4.167632	-0.315545	-2.222920
18	6	0	-6.086087	-0.513198	0.580684
19	1	0	-5.351082	-0.297415	2.598766
20	1	0	-6.521723	-0.682536	-1.525049
21	1	0	-7.115291	-0.673521	0.888826
22	6	0	-2.043850	0.118140	-0.660903
23	1	0	-1.891665	0.087123	-1.749450
24	8	0	1.220371	-1.754225	-0.774069
25	1	0	0.410500	-2.247799	-0.575653
26	6	0	3.688731	-0.416667	0.862092
27	1	0	3.271417	-0.630656	1.858353
28	6	0	4.340750	-1.681351	0.307650
29	1	0	5.031497	-2.078723	1.067511
30	1	0	3.580310	-2.440716	0.107642
31	8	0	4.740064	0.550427	0.959388
32	1	0	4.306555	1.414058	1.039825
33	8	0	5.022108	-1.419149	-0.913900
34	1	0	5.515368	-0.600009	-0.746663
35	8	0	0.917134	2.723771	-0.999035
36	1	0	1.246146	3.568372	-0.654337

Structure 56c (M06-2X/6-311G(d,p), Gas Phase)

Energy (Hartrees): = -936.3380508
No imaginary frequencies

Standard orientation:

Center Number	Atomic Number	Atomic Type	Coordinates (Angstroms) X	Y	Z
1	6	0	2.555396	0.128305	0.000782
2	8	0	2.236180	1.436471	0.485280
3	6	0	0.925941	1.780983	0.066786
4	6	0	0.253742	0.473099	-0.371942
5	6	0	1.224622	-0.598293	0.135110
6	1	0	2.862321	0.163452	-1.051527
7	1	0	0.424170	2.237342	0.924479
8	1	0	0.269942	0.443723	-1.470678
9	1	0	1.019602	-0.788247	1.197616
10	7	0	-1.071237	0.309394	0.172534
11	6	0	-3.412204	-0.100603	-0.217716
12	6	0	-3.762118	-0.085307	1.135365
13	6	0	-4.398539	-0.304254	-1.182132
14	6	0	-5.083167	-0.269476	1.510873
15	1	0	-2.983050	0.074894	1.870808
16	6	0	-5.723497	-0.488938	-0.804167
17	1	0	-4.125694	-0.314172	-2.232600
18	6	0	-6.066194	-0.470866	0.542316
19	1	0	-5.353395	-0.256125	2.560119
20	1	0	-6.485628	-0.644336	-1.558155
21	1	0	-7.098101	-0.613329	0.840306
22	6	0	-2.016624	0.103738	-0.646195
23	1	0	-1.839894	0.075737	-1.730814
24	8	0	1.205316	-1.785281	-0.616221
25	1	0	0.362319	-2.221044	-0.463039
26	6	0	3.696453	-0.403490	0.848652
27	1	0	3.317611	-0.612051	1.859513
28	6	0	4.313320	-1.659691	0.249684
29	1	0	5.045159	-2.059724	0.962262
30	1	0	3.540416	-2.408368	0.070910
31	8	0	4.740521	0.555520	0.900355
32	1	0	4.326434	1.414639	1.032448
33	8	0	4.917722	-1.367224	-0.990569
34	1	0	5.463951	-0.589137	-0.833829
35	8	0	0.943588	2.640308	-1.032457
36	1	0	1.363088	3.461390	-0.761149

Structure 56c (M06-2X/6-311G(d,p), DMSO)

Energy (Hartrees): = -936.3646636

No imaginary frequencies

Standard orientation:

Center Number	Atomic Number	Atomic Type	Coordinates (Angstroms) X	Y	Z
1	6	0	2.553664	0.135107	-0.024841
2	8	0	2.290405	1.485018	0.365480
3	6	0	0.974354	1.850439	-0.032034
4	6	0	0.265825	0.548137	-0.416380
5	6	0	1.197024	-0.535721	0.140998
6	1	0	2.868907	0.088454	-1.074654
7	1	0	0.504934	2.338855	0.826223
8	1	0	0.278234	0.461908	-1.511430
9	1	0	0.988217	-0.681207	1.209125
10	7	0	-1.066543	0.450452	0.130835
11	6	0	-3.384020	-0.112015	-0.207699
12	6	0	-3.771641	0.147955	1.111183
13	6	0	-4.333301	-0.557097	-1.128997
14	6	0	-5.091168	-0.036508	1.496665
15	1	0	-3.030749	0.495688	1.821733
16	6	0	-5.656137	-0.743742	-0.740436
17	1	0	-4.030634	-0.758297	-2.151754
18	6	0	-6.035834	-0.483634	0.572080
19	1	0	-5.388176	0.167818	2.518948
20	1	0	-6.387491	-1.093036	-1.460003
21	1	0	-7.065910	-0.627433	0.877586
22	6	0	-1.989525	0.071397	-0.653389
23	1	0	-1.795508	-0.140704	-1.712510
24	8	0	1.132150	-1.749208	-0.567347
25	1	0	0.291704	-2.171036	-0.354962
26	6	0	3.665096	-0.377963	0.872257
27	1	0	3.278478	-0.458530	1.896828
28	6	0	4.204773	-1.724383	0.423771
29	1	0	4.927111	-2.076291	1.170530
30	1	0	3.393120	-2.449398	0.352860
31	8	0	4.768138	0.519942	0.824153
32	1	0	4.405371	1.411946	0.871231
33	8	0	4.808322	-1.626492	-0.853916
34	1	0	5.385889	-0.855318	-0.804903
35	8	0	0.987445	2.691114	-1.143545
36	1	0	1.355955	3.538597	-0.868366

Structure 56c (M06-2X/de2-TZVP, Gas Phase)

Energy (Hartrees): = -936.4552457
No imaginary frequencies

Standard orientation:

Center Number	Atomic Number	Atomic Type	Coordinates (Angstroms) X	Y	Z
1	6	0	2.560051	0.144236	-0.013636
2	8	0	2.239217	1.450189	0.469432
3	6	0	0.921413	1.790126	0.080645
4	6	0	0.254680	0.486918	-0.372537
5	6	0	1.230815	-0.584384	0.118926
6	1	0	2.865869	0.180817	-1.065566
7	1	0	0.428570	2.227265	0.952240
8	1	0	0.268066	0.470426	-1.470592
9	1	0	1.033012	-0.784965	1.179429
10	7	0	-1.066465	0.309669	0.168477
11	6	0	-3.407209	-0.099126	-0.213555
12	6	0	-3.755752	-0.104002	1.137223
13	6	0	-4.392026	-0.294772	-1.177221
14	6	0	-5.072770	-0.300498	1.510653
15	1	0	-2.979551	0.050012	1.875420
16	6	0	-5.713271	-0.492030	-0.801775
17	1	0	-4.119821	-0.289098	-2.226704
18	6	0	-6.053941	-0.494496	0.542347
19	1	0	-5.341802	-0.303000	2.559170
20	1	0	-6.474191	-0.641825	-1.556481
21	1	0	-7.083583	-0.647432	0.839241
22	6	0	-2.016262	0.115969	-0.643940
23	1	0	-1.845350	0.106635	-1.728595
24	8	0	1.204477	-1.764839	-0.640418
25	1	0	0.367765	-2.213852	-0.483001
26	6	0	3.697667	-0.387025	0.836715
27	1	0	3.318563	-0.568953	1.851471
28	6	0	4.292894	-1.668693	0.278447
29	1	0	5.020466	-2.053143	1.001876
30	1	0	3.510456	-2.413049	0.133146
31	8	0	4.756003	0.554552	0.873010
32	1	0	4.367888	1.426624	1.006759

33	8	0	4.898066	-1.443411	-0.973878
34	1	0	5.474493	-0.677790	-0.865600
35	8	0	0.911932	2.675941	-0.997993
36	1	0	1.299109	3.510855	-0.716532

Structure 56c (M06-2X/de2-TZVP, DMSO)

Energy (Hartrees): = -936.482
No imaginary frequencies

Standard orientation:

Center Number	Atomic Number	Atomic Type	Coordinates (Angstroms) X	Y	Z
1	6	0	2.581400	0.159200	-0.084115
2	8	0	2.310846	1.518552	0.266327
3	6	0	0.930768	1.819344	0.100876
4	6	0	0.271378	0.521467	-0.353611
5	6	0	1.249503	-0.541810	0.144082
6	1	0	2.862389	0.086838	-1.141171
7	1	0	0.546600	2.156991	1.067158
8	1	0	0.285715	0.500322	-1.451794
9	1	0	1.097150	-0.703118	1.218647
10	7	0	-1.053439	0.350277	0.182179
11	6	0	-3.381703	-0.125310	-0.206590
12	6	0	-3.755069	-0.080715	1.137853
13	6	0	-4.347792	-0.372053	-1.179416
14	6	0	-5.077144	-0.278355	1.497185
15	1	0	-3.001322	0.108936	1.891852
16	6	0	-5.673800	-0.570401	-0.817991
17	1	0	-4.054797	-0.405152	-2.222938
18	6	0	-6.039075	-0.523164	0.520028
19	1	0	-5.364134	-0.243675	2.540879
20	1	0	-6.419832	-0.758697	-1.579807
21	1	0	-7.072486	-0.677290	0.805153
22	6	0	-1.988629	0.091370	-0.631837
23	1	0	-1.804616	0.023098	-1.710413
24	8	0	1.175766	-1.750645	-0.569052
25	1	0	0.345697	-2.187723	-0.340660
26	6	0	3.727634	-0.307588	0.792512
27	1	0	3.379944	-0.326632	1.832895
28	6	0	4.244086	-1.683789	0.423343
29	1	0	4.988469	-1.983913	1.169303
30	1	0	3.427862	-2.405685	0.439412
31	8	0	4.830164	0.579530	0.658292
32	1	0	4.487222	1.480474	0.708666
33	8	0	4.805141	-1.702460	-0.875926
34	1	0	5.403938	-0.946453	-0.925517
35	8	0	0.732019	2.796003	-0.870354
36	1	0	0.985435	3.652726	-0.505134

Structure 56d ($^1T_2$) (B3LYP, Gas Phase)

Energy (Hartrees): = -936.4798897
No imaginary frequencies

Standard orientation:

Center Number	Atomic Number	Atomic Type	Coordinates (Angstroms) X	Y	Z
1	6	0	2.573030	0.035843	0.283039
2	8	0	2.292797	-0.287809	-1.108337
3	6	0	1.626251	-1.518869	-1.179870
4	6	0	0.781381	-1.605059	0.100627
5	6	0	1.794733	-1.014101	1.127581
6	1	0	3.645718	-0.096439	0.470653
7	1	0	1.047016	-1.522826	-2.109690
8	1	0	0.531593	-2.642193	0.358548
9	1	0	1.278328	-0.546671	1.968234
10	7	0	-0.378606	-0.748275	-0.034485
11	6	0	-2.789799	-0.486273	0.043320
12	6	0	-2.801995	0.916338	-0.055682
13	6	0	-4.008939	-1.181155	0.073350
14	6	0	-4.011087	1.598715	-0.133053
15	1	0	-1.864412	1.460746	-0.053200
16	6	0	-5.218867	-0.494736	-0.008739
17	1	0	-4.004012	-2.265078	0.157474
18	6	0	-5.221070	0.896656	-0.113607
19	1	0	-4.013751	2.682131	-0.204395
20	1	0	-6.155914	-1.042704	0.011395
21	1	0	-6.162027	1.435420	-0.174494
22	6	0	-1.540941	-1.254968	0.122664
23	1	0	-1.661056	-2.329741	0.327962

24	8	0	2.632623	-2.019399	1.660394
25	1	0	2.988843	-2.505179	0.898129
26	6	0	2.207185	1.496568	0.549121
27	1	0	2.580244	1.745938	1.552878
28	6	0	2.847736	2.470960	-0.448371
29	1	0	3.940499	2.448713	-0.362257
30	1	0	2.580218	2.165862	-1.470856
31	8	0	0.800469	1.733161	0.567553
32	1	0	0.348665	1.018815	0.072174
33	8	0	2.425220	3.793446	-0.169435
34	1	0	1.468625	3.714094	-0.026980
35	8	0	2.533403	-2.617544	-1.139358
36	1	0	3.199862	-2.457319	-1.822985

Structure 56d ($^{1}T_{2}$) (B3LYP, DMSO)

Energy (Hartrees): = -936.503427
No imaginary frequencies

Standard orientation:

Center Number	Atomic Number	Atomic Type	Coordinates (Angstroms) X	Y	Z
1	6	0	2.572680	0.060840	0.271765
2	8	0	2.274898	-0.235129	-1.121644
3	6	0	1.637643	-1.486050	-1.207643
4	6	0	0.812151	-1.605551	0.082328
5	6	0	1.835381	-1.026642	1.103047
6	1	0	3.651885	-0.042531	0.434883
7	1	0	1.049908	-1.492223	-2.131157
8	1	0	0.562361	-2.645792	0.319993
9	1	0	1.332218	-0.599111	1.972463
10	7	0	-0.352897	-0.746256	-0.024705
11	6	0	-2.767126	-0.510321	0.026215
12	6	0	-2.802820	0.858541	-0.297266
13	6	0	-3.973843	-1.187411	0.267691
14	6	0	-4.019891	1.528604	-0.370872
15	1	0	-1.877778	1.389980	-0.494381
16	6	0	-5.192247	-0.513086	0.193830
17	1	0	-3.950877	-2.245515	0.514935
18	6	0	-5.217103	0.845891	-0.124920
19	1	0	-4.039409	2.584990	-0.622232
20	1	0	-6.118853	-1.046634	0.383525
21	1	0	-6.164561	1.373587	-0.184720
22	6	0	-1.511898	-1.269194	0.117624
23	1	0	-1.623691	-2.342688	0.319535
24	8	0	2.711836	-2.032297	1.584584
25	1	0	3.066112	-2.469402	0.790912
26	6	0	2.179262	1.503019	0.594390
27	1	0	2.603012	1.730367	1.583094
28	6	0	2.746132	2.516680	-0.404418
29	1	0	3.841761	2.516848	-0.376969
30	1	0	2.430334	2.242650	-1.421503
31	8	0	0.769065	1.713668	0.690798
32	1	0	0.301410	0.982168	0.226822
33	8	0	2.306143	3.827671	-0.067340
34	1	0	1.358177	3.713719	0.112071
35	8	0	2.570531	-2.562597	-1.192448
36	1	0	3.198561	-2.409054	-1.916570

Structure 56d ($^{1}T_{2}$) (M06-2X/6-311G(d,p), Gas Phase)

Energy (Hartrees): = -936.3484487
No imaginary frequencies

Standard orientation:

Center Number	Atomic Number	Atomic Type	Coordinates (Angstroms) X	Y	Z
1	6	0	2.513047	0.025924	0.262275
2	8	0	2.199259	-0.286607	-1.109660
3	6	0	1.611687	-1.550643	-1.160460
4	6	0	0.762157	-1.643153	0.106344
5	6	0	1.731750	-0.997260	1.126791
6	1	0	3.584112	-0.123800	0.435561
7	1	0	1.046748	-1.614470	-2.092781
8	1	0	0.531706	-2.678064	0.380401
9	1	0	1.180652	-0.499542	1.924570
10	7	0	-0.402345	-0.804976	-0.057651
11	6	0	-2.784946	-0.506732	0.055152
12	6	0	-2.741694	0.889679	0.108922
13	6	0	-4.013702	-1.157099	-0.056676
14	6	0	-3.917590	1.619419	0.030339

15	1	0	-1.788248	1.387662	0.241481
16	6	0	-5.190201	-0.422481	-0.143164
17	1	0	-4.045644	-2.241481	-0.082908
18	6	0	-5.141531	0.966041	-0.102001
19	1	0	-3.883687	2.700944	0.079821
20	1	0	-6.141624	-0.931359	-0.238494
21	1	0	-6.057817	1.541207	-0.162428
22	6	0	-1.550260	-1.306100	0.129606
23	1	0	-1.675251	-2.372742	0.361910
24	8	0	2.568021	-1.953115	1.729272
25	1	0	2.973502	-2.458053	1.014194
26	6	0	2.167642	1.484679	0.513474
27	1	0	2.475583	1.736033	1.534841
28	6	0	2.883266	2.424100	-0.450507
29	1	0	3.965447	2.366479	-0.306999
30	1	0	2.648752	2.124321	-1.479137
31	8	0	0.773165	1.734721	0.430875
32	1	0	0.358101	1.037720	-0.102623
33	8	0	2.492464	3.754675	-0.198054
34	1	0	1.531510	3.726201	-0.131291
35	8	0	2.581410	-2.573466	-1.067390
36	1	0	3.220274	-2.434146	-1.773480

Structure 56d ($^{1}T_{2}$) (M06-2X/6-311G(d,p), DMSO)

Energy (Hartrees): = -936.374405
No imaginary frequencies

Standard orientation:

Center Number	Atomic Number	Atomic Type	Coordinates (Angstroms) X	Y	Z
1	6	0	2.532594	0.030762	0.255254
2	8	0	2.190378	-0.219127	-1.122863
3	6	0	1.614274	-1.490155	-1.217130
4	6	0	0.784626	-1.631046	0.058689
5	6	0	1.776984	-1.037395	1.086660
6	1	0	3.609012	-0.110217	0.394681
7	1	0	1.038482	-1.528005	-2.143218
8	1	0	0.538906	-2.671112	0.289789
9	1	0	1.248827	-0.588522	1.927588
10	7	0	-0.376858	-0.774146	-0.061497
11	6	0	-2.770155	-0.506440	0.018087
12	6	0	-2.770355	0.848951	-0.330484
13	6	0	-3.980195	-1.146769	0.292359
14	6	0	-3.966285	1.548121	-0.397683
15	1	0	-1.834995	1.347316	-0.556829
16	6	0	-5.178623	-0.443515	0.225666
17	1	0	-3.977538	-2.198485	0.559593
18	6	0	-5.172137	0.904057	-0.118346
19	1	0	-3.963146	2.597241	-0.669315
20	1	0	-6.113917	-0.946636	0.440919
21	1	0	-6.104262	1.454554	-0.172493
22	6	0	-1.523985	-1.288069	0.107891
23	1	0	-1.642948	-2.353703	0.335701
24	8	0	2.635613	-2.023203	1.614430
25	1	0	3.038186	-2.465040	0.855804
26	6	0	2.182745	1.468560	0.601383
27	1	0	2.584248	1.672302	1.600650
28	6	0	2.796466	2.462960	-0.373732
29	1	0	3.887402	2.411066	-0.338494
30	1	0	2.465382	2.220937	-1.390126
31	8	0	0.781720	1.706383	0.657047
32	1	0	0.317873	0.999333	0.172458
33	8	0	2.416748	3.780834	-0.017648
34	1	0	1.464265	3.730725	0.127882
35	8	0	2.592639	-2.506799	-1.172901
36	1	0	3.189730	-2.377565	-1.920319

Structure 56d ($^{1}T_{2}$) (M06-2X/def2-TZVP, Gas Phase)

Energy (Hartrees): = -936.4631177
No imaginary frequencies

Standard orientation:

Center Number	Atomic Number	Atomic Type	Coordinates (Angstroms) X	Y	Z
1	6	0	2.535176	-0.027013	0.260296
2	8	0	2.208095	-0.301948	-1.113283
3	6	0	1.557944	-1.530265	-1.192964
4	6	0	0.721505	-1.620355	0.081723
5	6	0	1.720184	-1.035319	1.105372

6	1	0	3.599248	-0.219609	0.425842
7	1	0	0.976273	-1.537914	-2.116047
8	1	0	0.461060	-2.652106	0.333808
9	1	0	1.195646	-0.535238	1.918076
10	7	0	-0.422639	-0.751874	-0.052863
11	6	0	-2.811994	-0.443190	0.038240
12	6	0	-2.787127	0.939704	-0.146701
13	6	0	-4.035016	-1.098635	0.152673
14	6	0	-3.972988	1.647340	-0.221860
15	1	0	-1.836866	1.451173	-0.222757
16	6	0	-5.223805	-0.388023	0.074216
17	1	0	-4.052544	-2.172016	0.302775
18	6	0	-5.192519	0.985527	-0.113744
19	1	0	-3.951007	2.720060	-0.361755
20	1	0	-6.170440	-0.904493	0.161817
21	1	0	-6.117320	1.544611	-0.173108
22	6	0	-1.577148	-1.235257	0.123428
23	1	0	-1.710999	-2.299449	0.354904
24	8	0	2.519337	-2.034959	1.686158
25	1	0	2.914154	-2.547093	0.968466
26	6	0	2.249528	1.434437	0.555814
27	1	0	2.595107	1.637751	1.575749
28	6	0	2.987934	2.367563	-0.392615
29	1	0	4.067338	2.243766	-0.283351
30	1	0	2.709722	2.124091	-1.423382
31	8	0	0.869279	1.748838	0.512789
32	1	0	0.383384	1.045498	0.048315
33	8	0	2.685869	3.709176	-0.085591
34	1	0	1.726502	3.751362	0.009161
35	8	0	2.471983	-2.605682	-1.152583
36	1	0	3.102659	-2.489751	-1.871650

Structure 56d ($^1T_2$) (M06-2X/def2-TZVP, DMSO)

Energy (Hartrees): = -936.4895352
No imaginary frequencies

Standard orientation:

Center Number	Atomic Number	Atomic Type	Coordinates (Angstroms) X	Y	Z
1	6	0	2.516075	0.001717	0.258882
2	8	0	2.192108	-0.256353	-1.118415
3	6	0	1.576352	-1.506563	-1.216837
4	6	0	0.748896	-1.633906	0.060316
5	6	0	1.743931	-1.052620	1.086129
6	1	0	3.588324	-0.148637	0.415859
7	1	0	0.993092	-1.520086	-2.138525
8	1	0	0.489981	-2.669857	0.290314
9	1	0	1.220776	-0.597623	1.926359
10	7	0	-0.401566	-0.766173	-0.052592
11	6	0	-2.790681	-0.474079	0.018746
12	6	0	-2.787945	0.860094	-0.392498
13	6	0	-3.996242	-1.087049	0.352857
14	6	0	-3.975834	1.567739	-0.457690
15	1	0	-1.855506	1.334258	-0.672263
16	6	0	-5.186673	-0.375135	0.290558
17	1	0	-3.995929	-2.125415	0.664933
18	6	0	-5.176763	0.952466	-0.113452
19	1	0	-3.970583	2.601585	-0.779699
20	1	0	-6.119321	-0.857492	0.554581
21	1	0	-6.103744	1.510050	-0.165154
22	6	0	-1.553593	-1.265020	0.108462
23	1	0	-1.683514	-2.329309	0.331693
24	8	0	2.587551	-2.047088	1.622370
25	1	0	2.992985	-2.507974	0.874866
26	6	0	2.186753	1.444662	0.597136
27	1	0	2.512882	1.611746	1.630836
28	6	0	2.931938	2.417740	-0.299467
29	1	0	4.009291	2.309214	-0.160709
30	1	0	2.689274	2.207716	-1.346540
31	8	0	0.802339	1.745971	0.521550
32	1	0	0.312987	0.987293	0.147336
33	8	0	2.593831	3.752382	0.033753
34	1	0	1.628980	3.777936	0.071450
35	8	0	2.521146	-2.553653	-1.194361
36	1	0	3.107904	-2.450542	-1.955213

Structure $56\cdot5H_2O$ (M06-2X/6-311G(d,p), Gas Phase)

Energy (Hartrees): = -1318.5338343
No imaginary frequencies

Standard orientation:

Center Number	Atomic Number	Atomic Type	Coordinates (Angstroms) X	Y	Z
1	6	0	1.057407	0.223796	0.444439
2	8	0	0.817691	-0.182845	-0.910593
3	6	0	0.292576	-1.488206	-0.909265
4	6	0	-0.601650	-1.536401	0.334549
5	6	0	0.330912	-0.823760	1.336542
6	1	0	2.131208	0.182183	0.656967
7	1	0	-0.231962	-1.619719	-1.858770
8	1	0	-0.820273	-2.563778	0.642088
9	1	0	-0.238728	-0.347162	2.135122
10	7	0	-1.778002	-0.721648	0.137676
11	6	0	-4.175580	-0.493500	0.097885
12	6	0	-4.181134	0.905131	0.105567
13	6	0	-5.378397	-1.189054	-0.026103
14	6	0	-5.379071	1.589731	-0.025670
15	1	0	-3.250230	1.445336	0.233725
16	6	0	-6.576955	-0.499627	-0.163142
17	1	0	-5.372465	-2.274094	-0.020824
18	6	0	-6.576646	0.890177	-0.164666
19	1	0	-5.382425	2.672727	-0.015459
20	1	0	-7.507573	-1.044160	-0.265327
21	1	0	-7.509975	1.430728	-0.267666
22	6	0	-2.923216	-1.256011	0.228709
23	1	0	-3.030349	-2.333060	0.414812
24	8	0	1.218385	-1.733792	1.953395
25	1	0	1.581539	-2.277821	1.236838
26	6	0	0.573273	1.662273	0.667165
27	1	0	0.586459	1.821475	1.755594
28	6	0	1.465310	2.727845	0.040487
29	1	0	1.175888	3.703561	0.446745
30	1	0	2.515021	2.537238	0.275907
31	8	0	-0.722354	1.885186	0.146029
32	1	0	-1.164358	1.027393	0.000731
33	8	0	1.318056	2.735063	-1.369413
34	1	0	0.384085	2.541685	-1.526334
35	8	0	1.277314	-2.458289	-0.703484
36	1	0	2.002211	-2.346736	-1.354699
37	8	0	4.587288	1.737717	1.421569
38	1	0	5.058397	1.189375	0.777143
39	8	0	3.422360	-1.908324	-2.203415
40	1	0	4.086559	-1.575733	-1.559185
41	1	0	3.897929	-2.345971	-2.911191
42	8	0	4.736376	-0.502404	-0.315242
43	1	0	4.160975	0.173666	-0.734764
44	1	0	4.356312	-0.741214	0.551480
45	8	0	3.841910	-0.757754	2.285998
46	1	0	2.923548	-0.995235	2.474512
47	1	0	3.940279	0.189875	2.447422
48	1	0	5.078193	2.558512	1.489898
49	8	0	3.269027	0.940966	-2.002226
50	1	0	2.835302	0.132154	-2.298168
51	1	0	2.551061	1.591766	-1.910253

Structure 56·5H_2O (M06-2X/6-311G(d,p), DMSO)

Energy (Hartrees): = -1318.5675577
No imaginary frequencies

Standard orientation:

Center Number	Atomic Number	Atomic Type	Coordinates (Angstroms) X	Y	Z
1	6	0	1.066341	0.255466	0.452949
2	8	0	0.823158	-0.187994	-0.892143
3	6	0	0.265432	-1.482538	-0.858274
4	6	0	-0.596529	-1.500034	0.407000
5	6	0	0.361720	-0.777535	1.373522
6	1	0	2.142795	0.233422	0.656481
7	1	0	-0.285808	-1.619934	-1.791229
8	1	0	-0.823414	-2.518226	0.733857
9	1	0	-0.174619	-0.294157	2.189716
10	7	0	-1.774981	-0.681855	0.215763
11	6	0	-4.173498	-0.490475	0.120529
12	6	0	-4.207801	0.908128	0.152472
13	6	0	-5.358869	-1.206824	-0.054655
14	6	0	-5.413874	1.575004	-0.004613
15	1	0	-3.292965	1.465499	0.319492
16	6	0	-6.565933	-0.535625	-0.217984
17	1	0	-5.329625	-2.291541	-0.067339
18	6	0	-6.593455	0.854823	-0.194557
19	1	0	-5.438880	2.658038	0.024855
20	1	0	-7.482704	-1.095564	-0.360289
21	1	0	-7.533680	1.379867	-0.318252

22	6	0	-2.913733	-1.238638	0.272872
23	1	0	-3.009634	-2.318109	0.436360
24	8	0	1.275698	-1.691047	1.962017
25	1	0	1.587990	-2.251654	1.233769
26	6	0	0.574128	1.695920	0.643895
27	1	0	0.596514	1.885522	1.725817
28	6	0	1.454956	2.742454	-0.021983
29	1	0	1.152675	3.733275	0.334254
30	1	0	2.505303	2.579936	0.230272
31	8	0	-0.725387	1.900214	0.117553
32	1	0	-1.188082	1.039070	0.059635
33	8	0	1.321268	2.682357	-1.438171
34	1	0	0.397991	2.435719	-1.590402
35	8	0	1.244246	-2.468227	-0.673402
36	1	0	1.954656	-2.352499	-1.338908
37	8	0	4.551546	1.768097	1.389309
38	1	0	5.136411	1.342097	0.749588
39	8	0	3.370555	-1.951337	-2.218168
40	1	0	4.047416	-1.658222	-1.573677
41	1	0	3.814543	-2.480399	-2.888233
42	8	0	4.755884	-0.553554	-0.330162
43	1	0	4.198173	0.134781	-0.747135
44	1	0	4.397177	-0.729691	0.559482
45	8	0	3.897580	-0.730512	2.318921
46	1	0	2.959537	-0.957321	2.409968
47	1	0	3.959730	0.235913	2.349039
48	1	0	4.967169	2.606284	1.616327
49	8	0	3.281154	0.890627	-2.068884
50	1	0	2.876444	0.047411	-2.309390
51	1	0	2.530833	1.493725	-1.916382

Structure 56·5H_2O (M06-2X/6-311G(d,p), water)

Energy (Hartrees): = -1318.585751
No imaginary frequencies

Standard orientation:

Center Number	Atomic Number	Atomic Type	Coordinates (Angstroms) X	Y	Z
1	6	0	1.143243	0.263393	0.333652
2	8	0	0.867282	-0.224868	-0.993733
3	6	0	0.263091	-1.497795	-0.910195
4	6	0	-0.571066	-1.450325	0.370072
5	6	0	0.414196	-0.711499	1.295249
6	1	0	2.219643	0.210256	0.530682
7	1	0	-0.308414	-1.643038	-1.828423
8	1	0	-0.799522	-2.452158	0.741240
9	1	0	-0.099571	-0.181627	2.096420
10	7	0	-1.751132	-0.632907	0.174325
11	6	0	-4.161147	-0.472282	0.148074
12	6	0	-4.221845	0.922759	0.053782
13	6	0	-5.341139	-1.218560	0.116154
14	6	0	-5.449249	1.554064	-0.083374
15	1	0	-3.309970	1.506797	0.100613
16	6	0	-6.570011	-0.583413	-0.026174
17	1	0	-5.289879	-2.299146	0.199612
18	6	0	-6.624110	0.802650	-0.127735
19	1	0	-5.494677	2.634305	-0.153605
20	1	0	-7.481568	-1.168143	-0.054529
21	1	0	-7.580264	1.300906	-0.237076
22	6	0	-2.886367	-1.193566	0.289497
23	1	0	-2.965665	-2.265179	0.502588
24	8	0	1.304205	-1.630476	1.912722
25	1	0	1.658846	-2.187794	1.200840
26	6	0	0.697268	1.721541	0.466405
27	1	0	0.812634	1.978205	1.527772
28	6	0	1.531112	2.711955	-0.331089
29	1	0	1.222755	3.724855	-0.052058
30	1	0	2.591338	2.596056	-0.107715
31	8	0	-0.645310	1.920749	0.043504
32	1	0	-1.127463	1.066424	0.037026
33	8	0	1.364322	2.527024	-1.734323
34	1	0	0.434930	2.296996	-1.868498
35	8	0	1.219155	-2.512124	-0.723020
36	1	0	1.932109	-2.398703	-1.387717
37	8	0	3.995957	2.026426	1.845827
38	1	0	4.405812	1.789445	1.004851
39	8	0	3.391674	-1.975080	-2.214078
40	1	0	3.956336	-1.795924	-1.436224
41	1	0	3.876598	-2.594517	-2.769105
42	8	0	4.669227	-0.757275	-0.106923
43	1	0	4.270796	0.018048	-0.537772
44	1	0	4.343886	-0.756404	0.812695
45	8	0	3.837703	-0.639558	2.570453

46	1	0	2.912191	-0.916077	2.461618
47	1	0	3.822566	0.330036	2.481722
48	1	0	4.655069	2.570843	2.290962
49	8	0	3.529845	0.851295	-2.090242
50	1	0	3.298959	-0.048624	-2.366292
51	1	0	2.684108	1.332072	-2.057307

Structure 56·5H_2O (M06-2X/def2-TZVP, Gas Phase)

Energy (Hartrees): = -1318.6860182
No imaginary frequencies

Standard orientation:

Center Number	Atomic Number	Atomic Type	Coordinates (Angstroms) X	Y	Z
1	6	0	1.059573	0.248874	0.471169
2	8	0	0.781629	-0.100328	-0.888855
3	6	0	0.234533	-1.390412	-0.928658
4	6	0	-0.632228	-1.473846	0.330905
5	6	0	0.322459	-0.806360	1.338318
6	1	0	2.135157	0.166916	0.660686
7	1	0	-0.316647	-1.476048	-1.867539
8	1	0	-0.851850	-2.508633	0.607085
9	1	0	-0.226949	-0.341581	2.156555
10	7	0	-1.811196	-0.656602	0.172994
11	6	0	-4.215506	-0.477100	0.078727
12	6	0	-4.256440	0.912157	-0.049851
13	6	0	-5.404311	-1.201920	0.080567
14	6	0	-5.473199	1.556983	-0.177815
15	1	0	-3.335256	1.480028	-0.044261
16	6	0	-6.623537	-0.553790	-0.050244
17	1	0	-5.371015	-2.280541	0.183879
18	6	0	-6.657726	0.826408	-0.179791
19	1	0	-5.502387	2.634296	-0.275602
20	1	0	-7.542920	-1.124214	-0.050036
21	1	0	-7.606966	1.336555	-0.281452
22	6	0	-2.948195	-1.207930	0.216199
23	1	0	-3.038916	-2.291492	0.362636
24	8	0	1.199696	-1.748611	1.918846
25	1	0	1.550913	-2.282690	1.187910
26	6	0	0.632369	1.688209	0.757180
27	1	0	0.729200	1.822749	1.844216
28	6	0	1.513720	2.738108	0.099314
29	1	0	1.248667	3.717691	0.509697
30	1	0	2.566425	2.538300	0.311603
31	8	0	-0.690206	1.951901	0.341358
32	1	0	-1.171258	1.112023	0.206499
33	8	0	1.340496	2.746098	-1.306334
34	1	0	0.407166	2.545721	-1.461802
35	8	0	1.208000	-2.384843	-0.795876
36	1	0	1.922527	-2.255823	-1.454802
37	8	0	4.795449	1.598398	1.485174
38	1	0	5.202461	1.058820	0.789899
39	8	0	3.408641	-1.894713	-2.336215
40	1	0	4.059516	-1.608254	-1.655416
41	1	0	3.865467	-2.465037	-2.958359
42	8	0	4.719767	-0.582584	-0.368765
43	1	0	4.165147	0.125069	-0.765044
44	1	0	4.345898	-0.838343	0.497161
45	8	0	3.903316	-0.933945	2.267720
46	1	0	2.968358	-1.106347	2.452643
47	1	0	4.080975	-0.003076	2.463427
48	1	0	5.382932	2.341862	1.639024
49	8	0	3.348214	1.012609	-2.042298
50	1	0	2.998773	0.227662	-2.482290
51	1	0	2.583870	1.602454	-1.901434

Structure 56·5H_2O (M06-2X/def2-TZVP, DMSO)

Energy (Hartrees): = -1318.7205675
No imaginary frequencies

Standard orientation:

Center Number	Atomic Number	Atomic Type	Coordinates (Angstroms) X	Y	Z
1	6	0	-0.993798	0.259568	-0.533413
2	8	0	-0.782079	0.077504	0.873085
3	6	0	-0.242412	-1.200021	1.102164
4	6	0	0.642744	-1.467542	-0.115889
5	6	0	-0.274851	-0.924134	-1.225020
6	1	0	-2.064051	0.196133	-0.757417

7	1	0	0.292677	-1.160613	2.052818
8	1	0	0.854325	-2.531041	-0.247232
9	1	0	0.292060	-0.601534	-2.097121
10	7	0	1.836593	-0.656729	-0.036700
11	6	0	4.243631	-0.521529	-0.031332
12	6	0	4.322882	0.842335	0.256827
13	6	0	5.411559	-1.240291	-0.278049
14	6	0	5.554350	1.473578	0.285768
15	1	0	3.420324	1.400670	0.471422
16	6	0	6.645421	-0.605273	-0.252871
17	1	0	5.347401	-2.300660	-0.494912
18	6	0	6.717167	0.752049	0.027860
19	1	0	5.612487	2.530623	0.513260
20	1	0	7.548407	-1.168667	-0.451297
21	1	0	7.678063	1.251034	0.051801
22	6	0	2.960813	-1.237403	-0.088596
23	1	0	3.027443	-2.325372	-0.191235
24	8	0	-1.182284	-1.920428	-1.667565
25	1	0	-1.521459	-2.349812	-0.865109
26	6	0	-0.504069	1.634862	-0.985725
27	1	0	-0.556683	1.628486	-2.082463
28	6	0	-1.373212	2.784740	-0.509469
29	1	0	-1.059828	3.698010	-1.024415
30	1	0	-2.421682	2.596144	-0.748563
31	8	0	0.809717	1.919726	-0.547564
32	1	0	1.274095	1.087462	-0.316612
33	8	0	-1.268744	2.968216	0.896007
34	1	0	-0.355202	2.742418	1.125499
35	8	0	-1.233717	-2.191014	1.104170
36	1	0	-1.927509	-1.955914	1.753886
37	8	0	-5.503093	0.913196	-1.941560
38	1	0	-5.626196	0.631671	-1.021724
39	8	0	-3.345729	-1.407326	2.666781
40	1	0	-4.051640	-1.249187	2.008888
41	1	0	-3.737455	-1.876604	3.412212
42	8	0	-4.749573	-0.383587	0.517987
43	1	0	-4.195056	0.371022	0.807543
44	1	0	-4.314410	-0.812576	-0.244929
45	8	0	-3.950014	-1.410027	-1.940176
46	1	0	-3.004233	-1.417269	-2.153067
47	1	0	-4.350326	-0.622407	-2.340930
48	1	0	-6.378414	1.127864	-2.281540
49	8	0	-3.279705	1.413695	1.964931
50	1	0	-2.909762	0.642005	2.415098
51	1	0	-2.509122	1.919950	1.640724

Structure 56·5H_2O (M06-2X/def2-TZVP, water)

Energy (Hartrees): = -1318.7368444
No imaginary frequencies

Standard orientation:

Center Number	Atomic Number	Atomic Type	Coordinates (Angstroms) X	Y	Z
1	6	0	-1.110801	0.280247	-0.389243
2	8	0	-0.816549	-0.134619	0.955024
3	6	0	-0.223322	-1.412360	0.934468
4	6	0	0.594267	-1.435471	-0.355093
5	6	0	-0.393028	-0.739476	-1.306371
6	1	0	-2.190259	0.213519	-0.567270
7	1	0	0.361881	-1.512925	1.849219
8	1	0	0.819125	-2.454041	-0.676499
9	1	0	0.117945	-0.247566	-2.131809
10	7	0	1.776830	-0.616400	-0.196238
11	6	0	4.184426	-0.472583	-0.116918
12	6	0	4.247416	0.887285	0.194726
13	6	0	5.363985	-1.193283	-0.292063
14	6	0	5.476123	1.511238	0.323769
15	1	0	3.333010	1.447890	0.341833
16	6	0	6.595361	-0.565886	-0.162757
17	1	0	5.311466	-2.249014	-0.531963
18	6	0	6.651751	0.786191	0.144908
19	1	0	5.521945	2.564945	0.567490
20	1	0	7.508319	-1.130544	-0.300860
21	1	0	7.610607	1.278063	0.249490
22	6	0	2.908780	-1.184882	-0.267635
23	1	0	2.984032	-2.260426	-0.455301
24	8	0	-1.290232	-1.675186	-1.882450
25	1	0	-1.655636	-2.205464	-1.154670
26	6	0	-0.677535	1.727027	-0.617002
27	1	0	-0.816993	1.914997	-1.689590
28	6	0	-1.514603	2.760120	0.112044
29	1	0	-1.191507	3.753666	-0.212030
30	1	0	-2.568029	2.641317	-0.140915

31	8	0	0.671658	1.957421	-0.244413
32	1	0	1.165483	1.107867	-0.201152
33	8	0	-1.405531	2.656897	1.526978
34	1	0	-0.479972	2.464716	1.732849
35	8	0	-1.190128	-2.426415	0.826980
36	1	0	-1.853559	-2.308960	1.539703
37	8	0	-4.381203	1.858622	-1.861993
38	1	0	-4.488778	1.673622	-0.919622
39	8	0	-3.292535	-1.894416	2.506087
40	1	0	-3.874046	-1.813209	1.725886
41	1	0	-3.691099	-2.562120	3.076278
42	8	0	-4.591331	-0.929822	0.236891
43	1	0	-4.223565	-0.106009	0.603179
44	1	0	-4.318652	-0.959821	-0.699398
45	8	0	-3.908587	-0.831701	-2.527990
46	1	0	-2.956009	-1.006442	-2.430421
47	1	0	-4.011909	0.134087	-2.445113
48	1	0	-5.257224	2.132910	-2.160023
49	8	0	-3.566085	0.980925	2.059952
50	1	0	-3.313638	0.143248	2.477426
51	1	0	-2.733486	1.477843	1.949438

Structure 56f (B3LYP, Gas Phase)

Energy (Hartrees): = -936.4693264
No imaginary frequencies

Standard orientation:

Center Number	Atomic Number	Atomic Type	Coordinates (Angstroms) X	Y	Z
1	6	0	-2.608279	0.608624	0.288692
2	8	0	-2.280237	1.293787	-0.922639
3	6	0	-0.910614	1.695862	-0.857667
4	6	0	-0.244309	0.851002	0.256444
5	6	0	-1.332441	-0.193706	0.564088
6	1	0	-2.753585	1.319303	1.117132
7	1	0	-0.478061	1.493966	-1.844873
8	1	0	-0.128203	1.499968	1.138910
9	1	0	-1.236945	-1.013393	-0.159149
10	7	0	0.997773	0.245783	-0.167605
11	6	0	3.379337	-0.016534	0.156438
12	6	0	3.554211	-0.917931	-0.907450
13	6	0	4.490225	0.358432	0.926030
14	6	0	4.815874	-1.429449	-1.189511
15	1	0	2.688116	-1.199371	-1.496940
16	6	0	5.754737	-0.155562	0.641708
17	1	0	4.359231	1.056779	1.749069
18	6	0	5.919088	-1.050377	-0.416180
19	1	0	4.945251	-2.125041	-2.013484
20	1	0	6.608645	0.141481	1.243075
21	1	0	6.902985	-1.452039	-0.640115
22	6	0	2.057902	0.541405	0.481304
23	1	0	2.036035	1.243096	1.330687
24	8	0	-1.347560	-0.692224	1.896595
25	1	0	-0.595257	-1.289538	2.004598
26	6	0	-3.901718	-0.191041	0.131720
27	1	0	-4.723233	0.510873	-0.053305
28	6	0	-3.918692	-1.220729	-1.001940
29	1	0	-3.813349	-0.721857	-1.969498
30	1	0	-3.074061	-1.922646	-0.892511
31	8	0	-4.213536	-0.842153	1.368893
32	1	0	-3.378316	-1.144670	1.763470
33	8	0	-5.153479	-1.913258	-0.998904
34	1	0	-5.318640	-2.121150	-0.065657
35	8	0	-0.774717	3.047675	-0.504471
36	1	0	-1.200718	3.573167	-1.196024

Structure 56f (B3LYP, DMSO)

Energy (Hartrees): = -936.4939365
No imaginary frequencies

Standard orientation:

Center Number	Atomic Number	Atomic Type	Coordinates (Angstroms) X	Y	Z
1	6	0	-2.608019	0.591677	0.335047
2	8	0	-2.312501	1.366578	-0.829692
3	6	0	-0.927357	1.743140	-0.790379
4	6	0	-0.250674	0.851510	0.277195
5	6	0	-1.325300	-0.219985	0.538080
6	1	0	-2.745295	1.241105	1.213730

7	1	0	-0.527819	1.559197	-1.794392
8	1	0	-0.131498	1.450248	1.192968
9	1	0	-1.235092	-0.997317	-0.230254
10	7	0	0.997066	0.284156	-0.188474
11	6	0	3.369648	-0.036431	0.158449
12	6	0	3.568823	-0.836901	-0.980629
13	6	0	4.465055	0.264628	0.983130
14	6	0	4.837148	-1.322444	-1.282894
15	1	0	2.721492	-1.069061	-1.617848
16	6	0	5.736066	-0.223301	0.678407
17	1	0	4.314694	0.882612	1.864723
18	6	0	5.924107	-1.017456	-0.454425
19	1	0	4.983618	-1.939941	-2.164432
20	1	0	6.576946	0.015366	1.322986
21	1	0	6.912775	-1.398365	-0.693878
22	6	0	2.045260	0.498905	0.512310
23	1	0	2.012392	1.105822	1.428359
24	8	0	-1.320184	-0.794555	1.841249
25	1	0	-0.597108	-1.439251	1.882286
26	6	0	-3.899963	-0.206724	0.151817
27	1	0	-4.732360	0.498863	0.043096
28	6	0	-3.938880	-1.152124	-1.050479
29	1	0	-3.870053	-0.585825	-1.983792
30	1	0	-3.091265	-1.854412	-1.018345
31	8	0	-4.168841	-0.952049	1.349177
32	1	0	-3.314225	-1.282913	1.678129
33	8	0	-5.173673	-1.860355	-1.063376
34	1	0	-5.287405	-2.161701	-0.147270
35	8	0	-0.756843	3.083732	-0.417989
36	1	0	-1.088401	3.632705	-1.145483

Structure 56f (M06-2X/6-311G(d,p), Gas Phase)

Energy (Hartrees): = -936.3359131
No imaginary frequencies

Standard orientation:

Center Number	Atomic Number	Atomic Type	Coordinates (Angstroms) X	Y	Z
1	6	0	-2.586779	0.598239	0.276522
2	8	0	-2.272632	1.264303	-0.937478
3	6	0	-0.924339	1.694386	-0.860909
4	6	0	-0.248931	0.851440	0.238479
5	6	0	-1.315544	-0.204704	0.531185
6	1	0	-2.714062	1.316263	1.098943
7	1	0	-0.478131	1.526782	-1.844433
8	1	0	-0.139654	1.488453	1.127737
9	1	0	-1.218933	-1.006376	-0.209177
10	7	0	0.996671	0.263853	-0.188494
11	6	0	3.363598	-0.011097	0.158433
12	6	0	3.538197	-0.893151	-0.911413
13	6	0	4.461066	0.353086	0.937970
14	6	0	4.796698	-1.400716	-1.191715
15	1	0	2.674448	-1.163346	-1.506884
16	6	0	5.723171	-0.156347	0.655359
17	1	0	4.324380	1.040594	1.766384
18	6	0	5.891043	-1.033773	-0.408858
19	1	0	4.930887	-2.085263	-2.020573
20	1	0	6.572640	0.130523	1.263302
21	1	0	6.873468	-1.433103	-0.631122
22	6	0	2.036138	0.542382	0.480805
23	1	0	1.997867	1.221791	1.344247
24	8	0	-1.311077	-0.719029	1.846169
25	1	0	-0.520573	-1.253187	1.964370
26	6	0	-3.873702	-0.196440	0.140442
27	1	0	-4.691427	0.496453	-0.077202
28	6	0	-3.864878	-1.253717	-0.953705
29	1	0	-3.693736	-0.791788	-1.927125
30	1	0	-3.062184	-1.981403	-0.766298
31	8	0	-4.192314	-0.806661	1.385115
32	1	0	-3.371732	-1.124320	1.782918
33	8	0	-5.117485	-1.900155	-0.991795
34	1	0	-5.332734	-2.102352	-0.074505
35	8	0	-0.834181	3.033268	-0.474968
36	1	0	-1.280127	3.561967	-1.143038

Structure 56f (M06-2X/6-311G(d,p), DMSO)

Energy (Hartrees): = -936.3631557
No imaginary frequencies

Standard orientation:

Center Number	Atomic Number	Atomic Type	Coordinates (Angstroms) X	Y	Z
1	6	0	-2.587078	0.584814	0.331061
2	8	0	-2.298094	1.342865	-0.834340
3	6	0	-0.923407	1.712288	-0.797836
4	6	0	-0.254433	0.820807	0.262698
5	6	0	-1.321418	-0.246939	0.507090
6	1	0	-2.694969	1.237993	1.208733
7	1	0	-0.520715	1.535026	-1.798402
8	1	0	-0.140721	1.410624	1.182491
9	1	0	-1.245409	-1.006646	-0.278580
10	7	0	0.995677	0.260552	-0.194991
11	6	0	3.360384	-0.037111	0.160514
12	6	0	3.557646	-0.862382	-0.951478
13	6	0	4.447871	0.304536	0.966659
14	6	0	4.827403	-1.334889	-1.248925
15	1	0	2.709081	-1.125217	-1.572501
16	6	0	5.721026	-0.168980	0.666260
17	1	0	4.291191	0.944875	1.828953
18	6	0	5.911388	-0.988752	-0.440844
19	1	0	4.977780	-1.975270	-2.110409
20	1	0	6.561938	0.100879	1.294487
21	1	0	6.902241	-1.359504	-0.677164
22	6	0	2.026908	0.489574	0.507923
23	1	0	1.980571	1.101988	1.416849
24	8	0	-1.303976	-0.836958	1.791009
25	1	0	-0.548891	-1.434994	1.843491
26	6	0	-3.885093	-0.187424	0.163710
27	1	0	-4.700862	0.527142	0.020247
28	6	0	-3.907556	-1.161250	-1.003442
29	1	0	-3.744763	-0.634460	-1.945062
30	1	0	-3.118062	-1.914629	-0.883955
31	8	0	-4.178098	-0.888788	1.369922
32	1	0	-3.348394	-1.263684	1.695629
33	8	0	-5.177330	-1.787129	-1.073018
34	1	0	-5.361769	-2.089921	-0.176167
35	8	0	-0.757108	3.042341	-0.415485
36	1	0	-1.092986	3.597533	-1.128755

Structure 56e (B3LYP, Gas Phase)

Energy (Hartrees): = -936.4677642
No imaginary frequencies

Standard orientation:

Center Number	Atomic Number	Atomic Type	Coordinates (Angstroms) X	Y	Z
1	6	0	2.535773	-0.167964	0.259670
2	8	0	2.208213	-0.387212	-1.131044
3	6	0	1.455644	-1.567292	-1.233466
4	6	0	0.574224	-1.617947	0.021904
5	6	0	1.580666	-1.079170	1.084696
6	1	0	3.562168	-0.513443	0.444904
7	1	0	0.904380	-1.519946	-2.178356
8	1	0	0.300058	-2.652382	0.277170
9	1	0	1.064174	-0.520192	1.865742
10	7	0	-0.560638	-0.745985	-0.144838
11	6	0	-2.929321	-0.364074	0.048385
12	6	0	-2.843503	1.002013	-0.270568
13	6	0	-4.187438	-0.940377	0.271415
14	6	0	-4.000270	1.767154	-0.370320
15	1	0	-1.859161	1.433158	-0.422042
16	6	0	-5.346110	-0.170933	0.168196
17	1	0	-4.255086	-1.995857	0.524364
18	6	0	-5.253944	1.183550	-0.153860
19	1	0	-3.930387	2.823467	-0.613983
20	1	0	-6.316981	-0.626241	0.340164
21	1	0	-6.154657	1.785638	-0.232432
22	6	0	-1.716708	-1.191799	0.153643
23	1	0	-1.870631	-2.225491	0.508028
24	8	0	2.276881	-2.139580	1.717170
25	1	0	2.630806	-2.689460	0.999538
26	6	0	2.492567	1.320025	0.595188
27	1	0	2.992568	1.431062	1.574340
28	6	0	3.263410	2.161719	-0.420101
29	1	0	4.320575	1.858629	-0.451299
30	1	0	2.822056	2.008556	-1.411470
31	8	0	1.155695	1.778143	0.675791
32	1	0	1.226825	2.745023	0.692206
33	8	0	3.125925	3.520237	0.009593
34	1	0	3.302263	4.096810	-0.743111
35	8	0	2.289924	-2.724591	-1.177299
36	1	0	2.982461	-2.602845	-1.842266

Structure 56e (B3LYP, DMSO)

Energy (Hartrees): = -936.4919863
No imaginary frequencies

Standard orientation:

Center Number	Atomic Number	Atomic Type	Coordinates (Angstroms) X	Y	Z
1	6	0	2.530304	-0.293061	0.204424
2	8	0	2.094668	-0.439372	-1.164095
3	6	0	1.278648	-1.583010	-1.232734
4	6	0	0.433305	-1.566050	0.049209
5	6	0	1.493785	-1.063728	1.075680
6	1	0	3.500868	-0.791616	0.331392
7	1	0	0.706516	-1.519267	-2.164182
8	1	0	0.107569	-2.578421	0.324315
9	1	0	1.035030	-0.419118	1.826944
10	7	0	-0.670185	-0.641954	-0.095091
11	6	0	-3.045922	-0.232003	0.057844
12	6	0	-2.964553	1.136207	-0.257039
13	6	0	-4.305966	-0.810269	0.275786
14	6	0	-4.121875	1.902419	-0.355367
15	1	0	-1.988031	1.582593	-0.416140
16	6	0	-5.465548	-0.040780	0.174076
17	1	0	-4.371906	-1.867108	0.521798
18	6	0	-5.375565	1.316205	-0.142052
19	1	0	-4.052015	2.959085	-0.597510
20	1	0	-6.435950	-0.499072	0.341412
21	1	0	-6.276861	1.917365	-0.220760
22	6	0	-1.843644	-1.076560	0.160681
23	1	0	-2.023266	-2.117161	0.470528
24	8	0	2.095234	-2.146423	1.774565
25	1	0	2.439679	-2.740769	1.087934
26	6	0	2.743825	1.173154	0.557344
27	1	0	3.227504	1.177994	1.550307
28	6	0	3.682446	1.875263	-0.419670
29	1	0	4.667762	1.388101	-0.423531
30	1	0	3.262948	1.822748	-1.430857
31	8	0	1.507402	1.870902	0.613226
32	1	0	1.761591	2.808103	0.600580
33	8	0	3.777461	3.234501	0.025702
34	1	0	4.035914	3.777217	-0.732158
35	8	0	2.045056	-2.781827	-1.174882
36	1	0	2.699936	-2.735962	-1.889672

Structure 56e (M06-2X/6-311G(d,p), Gas Phase)

Energy (Hartrees): = -936.337028
No imaginary frequencies

Standard orientation:

Center Number	Atomic Number	Atomic Type	Coordinates (Angstroms) X	Y	Z
1	6	0	2.496636	-0.073385	0.255839
2	8	0	2.184263	-0.294129	-1.126635
3	6	0	1.558770	-1.536289	-1.238561
4	6	0	0.673532	-1.656344	-0.001373
5	6	0	1.621134	-1.059451	1.068782
6	1	0	3.546517	-0.335550	0.431973
7	1	0	1.017846	-1.550213	-2.186297
8	1	0	0.445657	-2.700869	0.244237
9	1	0	1.058897	-0.545361	1.846476
10	7	0	-0.489212	-0.831845	-0.189626
11	6	0	-2.837110	-0.461870	0.055819
12	6	0	-2.742455	0.892911	-0.275634
13	6	0	-4.087174	-1.035385	0.279215
14	6	0	-3.893682	1.655816	-0.390524
15	1	0	-1.754218	1.314586	-0.423717
16	6	0	-5.241133	-0.268453	0.158464
17	1	0	-4.155251	-2.085643	0.544679
18	6	0	-5.144322	1.076584	-0.176857
19	1	0	-3.822792	2.707253	-0.642955
20	1	0	-6.211964	-0.718803	0.327624
21	1	0	-6.042034	1.677009	-0.266441
22	6	0	-1.614111	-1.278995	0.173128
23	1	0	-1.736678	-2.288606	0.593604
24	8	0	2.390148	-2.062777	1.692244
25	1	0	2.796887	-2.575529	0.983457
26	6	0	2.313631	1.391045	0.610034
27	1	0	2.827007	1.556949	1.569487

28	6	0	2.942116	2.301838	-0.428632
29	1	0	4.016003	2.096885	-0.517082
30	1	0	2.453047	2.117293	-1.388927
31	8	0	0.942857	1.693849	0.740645
32	1	0	0.894007	2.654661	0.793027
33	8	0	2.704789	3.628566	0.028255
34	1	0	2.852516	4.236341	-0.697940
35	8	0	2.502651	-2.589316	-1.157663
36	1	0	3.166390	-2.435169	-1.836780

Structure 56e (M06-2X/6-311G(d,p), DMSO)

Energy (Hartrees): = -936.3636545
No imaginary frequencies

Standard orientation:

Center Number	Atomic Number	Atomic Type	Coordinates (Angstroms) X	Y	Z
1	6	0	2.510908	-0.178870	0.233647
2	8	0	2.146704	-0.327700	-1.145729
3	6	0	1.456645	-1.536729	-1.273298
4	6	0	0.567359	-1.619647	-0.035866
5	6	0	1.548751	-1.088496	1.037753
6	1	0	3.530358	-0.553943	0.379081
7	1	0	0.919268	-1.515861	-2.222936
8	1	0	0.282109	-2.650773	0.199072
9	1	0	1.021632	-0.536214	1.814120
10	7	0	-0.568237	-0.745801	-0.216139
11	6	0	-2.921427	-0.346579	0.053214
12	6	0	-2.887602	0.925714	-0.527360
13	6	0	-4.133971	-0.858714	0.517137
14	6	0	-4.052537	1.669972	-0.641223
15	1	0	-1.940984	1.316374	-0.882581
16	6	0	-5.302092	-0.110555	0.403163
17	1	0	-4.158722	-1.846093	0.967308
18	6	0	-5.262626	1.153136	-0.176199
19	1	0	-4.021921	2.655483	-1.091575
20	1	0	-6.240525	-0.513340	0.766488
21	1	0	-6.171630	1.737005	-0.265370
22	6	0	-1.696133	-1.161590	0.182318
23	1	0	-1.825746	-2.150010	0.642861
24	8	0	2.235945	-2.149699	1.671621
25	1	0	2.642634	-2.663826	0.962375
26	6	0	2.497552	1.282213	0.633555
27	1	0	2.992699	1.347198	1.614187
28	6	0	3.273689	2.140856	-0.345972
29	1	0	4.315893	1.805614	-0.400388
30	1	0	2.813403	2.059630	-1.334485
31	8	0	1.167030	1.750985	0.733332
32	1	0	1.239998	2.712179	0.771785
33	8	0	3.183867	3.474172	0.148209
34	1	0	3.423204	4.076887	-0.561842
35	8	0	2.338270	-2.640049	-1.196050
36	1	0	2.990362	-2.545630	-1.901395

Structure 56e (M06-2X/def2-TZVP, Gas Phase)

Energy (Hartrees): = -936.4528598
No imaginary frequencies

Standard orientation:

Center Number	Atomic Number	Atomic Type	Coordinates (Angstroms) X	Y	Z
1	6	0	2.499426	-0.157546	0.257000
2	8	0	2.188117	-0.346793	-1.126048
3	6	0	1.488961	-1.545778	-1.258790
4	6	0	0.587937	-1.630267	-0.031925
5	6	0	1.537959	-1.070691	1.052455
6	1	0	3.521827	-0.502852	0.446675
7	1	0	0.956174	-1.514089	-2.209931
8	1	0	0.326407	-2.666286	0.209496
9	1	0	0.982586	-0.509667	1.800854
10	7	0	-0.554978	-0.780758	-0.224729
11	6	0	-2.897838	-0.368849	0.051174
12	6	0	-2.826915	0.942345	-0.419875
13	6	0	-4.129020	-0.904777	0.413254
14	6	0	-3.980304	1.697408	-0.532761
15	1	0	-1.855432	1.342963	-0.682098
16	6	0	-5.285688	-0.145752	0.299190
17	1	0	-4.180226	-1.922099	0.784792
18	6	0	-5.211467	1.155002	-0.175249

19	1	0	-3.925316	2.715355	-0.896876
20	1	0	-6.241222	-0.568268	0.581624
21	1	0	-6.111437	1.750354	-0.262795
22	6	0	-1.680399	-1.188955	0.172592
23	1	0	-1.809539	-2.179351	0.631762
24	8	0	2.221632	-2.101455	1.728000
25	1	0	2.639444	-2.657300	1.057718
26	6	0	2.429522	1.311835	0.619395
27	1	0	2.884124	1.416902	1.615268
28	6	0	3.219167	2.165073	-0.353326
29	1	0	4.267690	1.848463	-0.368296
30	1	0	2.790278	2.048778	-1.351280
31	8	0	1.086627	1.733268	0.655548
32	1	0	1.102964	2.695459	0.720369
33	8	0	3.096413	3.504353	0.106658
34	1	0	3.356871	4.108326	-0.592013
35	8	0	2.362537	-2.656705	-1.188457
36	1	0	3.028994	-2.557013	-1.876573

Structure 56e (M06-2X/def2-TZVP, DMSO)

Energy (Hartrees): = -936.4801267
No imaginary frequencies

Standard orientation:

Center Number	Atomic Number	Atomic Type	Coordinates (Angstroms) X	Y	Z
1	6	0	2.498083	-0.313740	0.196690
2	8	0	2.071688	-0.408228	-1.163032
3	6	0	1.269117	-1.548145	-1.268884
4	6	0	0.406349	-1.552186	-0.010365
5	6	0	1.419038	-1.034825	1.038070
6	1	0	3.439397	-0.861210	0.320459
7	1	0	0.712389	-1.477134	-2.204417
8	1	0	0.083593	-2.564361	0.251735
9	1	0	0.933283	-0.354040	1.734109
10	7	0	-0.701066	-0.642302	-0.175282
11	6	0	-3.056757	-0.209221	0.046525
12	6	0	-3.003705	1.084599	-0.475108
13	6	0	-4.277981	-0.727021	0.471360
14	6	0	-4.157775	1.843615	-0.568295
15	1	0	-2.052802	1.483396	-0.806433
16	6	0	-5.435181	0.035625	0.379250
17	1	0	-4.317467	-1.732806	0.874696
18	6	0	-5.375963	1.320887	-0.140814
19	1	0	-4.112479	2.846784	-0.973737
20	1	0	-6.380512	-0.372876	0.713977
21	1	0	-6.276727	1.917843	-0.213219
22	6	0	-1.850562	-1.048949	0.159109
23	1	0	-2.011381	-2.058559	0.556139
24	8	0	1.966971	-2.082304	1.811630
25	1	0	2.376179	-2.709509	1.199971
26	6	0	2.764940	1.123851	0.584704
27	1	0	3.205682	1.095892	1.591670
28	6	0	3.758883	1.781019	-0.349759
29	1	0	4.702844	1.226716	-0.342711
30	1	0	3.354726	1.788592	-1.365308
31	8	0	1.558692	1.859727	0.608129
32	1	0	1.812720	2.789973	0.662828
33	8	0	3.942670	3.108894	0.128744
34	1	0	4.348295	3.636904	-0.566950
35	8	0	2.043596	-2.726029	-1.214327
36	1	0	2.669885	-2.709015	-1.949884

Structure 69a (B3LYP, Gas Phase)

Energy (Hartrees): = -936.4770251
No imaginary frequencies

Standard orientation:

Center Number	Atomic Number	Atomic Type	Coordinates (Angstroms) X	Y	Z
1	6	0	-1.158543	1.402701	0.631456
2	6	0	-0.495157	0.040060	0.301691
3	6	0	-1.244229	-0.604347	-0.876980
4	6	0	-2.738117	-0.716053	-0.574185
5	6	0	-3.308291	0.627817	-0.108903
6	1	0	-0.721285	1.809678	1.554538
7	1	0	-1.109817	0.030317	-1.762177
8	1	0	-3.260350	-0.995822	-1.500151

9	1	0	-3.346003	1.288784	-0.983740
10	1	0	-0.598718	-0.613918	1.180004
11	8	0	-2.513528	1.240454	0.917564
12	6	0	-4.707140	0.465118	0.513694
13	1	0	-4.566937	0.162629	1.558803
14	1	0	-5.226706	1.427046	0.507117
15	8	0	-5.514477	-0.455355	-0.201536
16	1	0	-5.144457	-1.328389	-0.001405
17	8	0	-2.972468	-1.702704	0.431681
18	1	0	-2.417214	-2.459083	0.187243
19	8	0	-0.786785	-1.931453	-1.130960
20	1	0	0.168271	-1.891878	-1.275749
21	8	0	-0.989628	2.305479	-0.442785
22	1	0	-0.089231	2.157097	-0.774432
23	7	0	0.887090	0.271338	-0.074682
24	6	0	3.242853	-0.172858	0.285177
25	6	0	3.699776	0.651453	-0.757438
26	6	0	4.177261	-0.861434	1.073204
27	6	0	5.062432	0.781019	-1.000124
28	1	0	2.971270	1.179170	-1.363527
29	6	0	5.543368	-0.729599	0.829040
30	1	0	3.828162	-1.499840	1.881067
31	6	0	5.987520	0.091648	-0.207880
32	1	0	5.409359	1.419430	-1.807140
33	1	0	6.258566	-1.265436	1.445569
34	1	0	7.051151	0.195871	-0.400608
35	6	0	1.810259	-0.333412	0.572296
36	1	0	1.567450	-1.019072	1.399368

Structure 69a (B3LYP, DMSO)

Energy (Hartrees): = -936.4993477
No imaginary frequencies

Standard orientation:

Center Number	Atomic Number	Atomic Type	Coordinates (Angstroms) X	Y	Z
1	6	0	1.152998	1.377563	-0.667769
2	6	0	0.492991	0.015402	-0.340366
3	6	0	1.236235	-0.645141	0.834000
4	6	0	2.737303	-0.729214	0.559540
5	6	0	3.294988	0.630205	0.133787
6	1	0	0.736846	1.765162	-1.608387
7	1	0	1.080782	-0.035950	1.733815
8	1	0	3.240017	-1.019286	1.492858
9	1	0	3.288753	1.277896	1.018890
10	1	0	0.581844	-0.631157	-1.224197
11	8	0	2.523456	1.240262	-0.914442
12	6	0	4.717645	0.512826	-0.439617
13	1	0	4.632800	0.215460	-1.492473
14	1	0	5.210793	1.488755	-0.406266
15	8	0	5.531864	-0.392368	0.294668
16	1	0	5.170418	-1.272140	0.103520
17	8	0	3.015410	-1.691593	-0.460869
18	1	0	2.455062	-2.456651	-0.253095
19	8	0	0.798134	-1.985504	1.052752
20	1	0	-0.168003	-1.974360	1.130772
21	8	0	0.938979	2.301258	0.384227
22	1	0	0.056497	2.090824	0.736607
23	7	0	-0.885980	0.262219	0.044876
24	6	0	-3.246743	-0.172316	-0.273987
25	6	0	-3.686185	0.663094	0.768640
26	6	0	-4.195197	-0.860530	-1.047156
27	6	0	-5.045949	0.804330	1.026121
28	1	0	-2.953060	1.192516	1.368629
29	6	0	-5.558174	-0.716365	-0.787844
30	1	0	-3.857862	-1.507213	-1.853132
31	6	0	-5.985306	0.115815	0.248707
32	1	0	-5.378805	1.450092	1.833538
33	1	0	-6.284340	-1.251697	-1.392350
34	1	0	-7.046067	0.228741	0.453419
35	6	0	-1.819871	-0.349652	-0.582058
36	1	0	-1.594963	-1.049074	-1.398963

Structure 69a (M06-2X/6-311G(d,p), Gas Phase)

Energy (Hartrees): = -936.3492278
No imaginary frequencies

Standard orientation:

Center Number	Atomic Number	Atomic Type	Coordinates (Angstroms) X	Y	Z

1	6	0	1.198791	1.414455	-0.673315
2	6	0	0.501074	0.082107	-0.349194
3	6	0	1.197184	-0.557444	0.853688
4	6	0	2.684535	-0.713099	0.579553
5	6	0	3.292050	0.605279	0.112581
6	1	0	0.781262	1.842863	-1.590249
7	1	0	1.062820	0.090403	1.726419
8	1	0	3.183477	-1.005991	1.510777
9	1	0	3.324258	1.283582	0.971702
10	1	0	0.606529	-0.590233	-1.210274
11	8	0	2.544192	1.206397	-0.940228
12	6	0	4.688003	0.382181	-0.472590
13	1	0	4.552459	0.024688	-1.497326
14	1	0	5.229026	1.327955	-0.503262
15	8	0	5.447563	-0.521030	0.301365
16	1	0	5.099425	-1.396350	0.103682
17	8	0	2.904448	-1.698648	-0.417837
18	1	0	2.343186	-2.447534	-0.186771
19	8	0	0.694935	-1.859803	1.103015
20	1	0	-0.237225	-1.788859	1.326795
21	8	0	1.060986	2.308168	0.403239
22	1	0	0.149037	2.228483	0.707486
23	7	0	-0.880276	0.346319	-0.004794
24	6	0	-3.218274	-0.181717	-0.277829
25	6	0	-3.665836	0.745271	0.667726
26	6	0	-4.143541	-0.967870	-0.963709
27	6	0	-5.021880	0.880020	0.917739
28	1	0	-2.935394	1.347751	1.193379
29	6	0	-5.503949	-0.831614	-0.712389
30	1	0	-3.794100	-1.686771	-1.697812
31	6	0	-5.942857	0.092191	0.228307
32	1	0	-5.368277	1.599136	1.650262
33	1	0	-6.218269	-1.443497	-1.249333
34	1	0	-7.002477	0.201143	0.426332
35	6	0	-1.782440	-0.345961	-0.566342
36	1	0	-1.527959	-1.114451	-1.310050

Structure 69a (M06-2X/6-311G(d,p), DMSO)

Energy (Hartrees): =-936.3739895
No imaginary frequencies

Standard orientation:

Center Number	Atomic Number	Atomic Type	Coordinates (Angstroms) X	Y	Z
1	6	0	1.180301	1.384310	-0.695398
2	6	0	0.497646	0.046176	-0.370113
3	6	0	1.200612	-0.602695	0.823879
4	6	0	2.693535	-0.724098	0.564888
5	6	0	3.278067	0.613410	0.130902
6	1	0	0.771669	1.790360	-1.626114
7	1	0	1.048924	0.021123	1.711721
8	1	0	3.181438	-1.023926	1.499732
9	1	0	3.272945	1.279143	0.999923
10	1	0	0.587591	-0.616331	-1.239260
11	8	0	2.540142	1.208534	-0.935206
12	6	0	4.693027	0.442548	-0.421687
13	1	0	4.604051	0.089065	-1.453281
14	1	0	5.203446	1.406639	-0.431385
15	8	0	5.471387	-0.441821	0.361161
16	1	0	5.125785	-1.324537	0.185787
17	8	0	2.955598	-1.685034	-0.448567
18	1	0	2.398688	-2.447782	-0.249470
19	8	0	0.726336	-1.920672	1.046009
20	1	0	-0.222743	-1.879993	1.211429
21	8	0	1.004186	2.298504	0.360453
22	1	0	0.113993	2.144674	0.705065
23	7	0	-0.880947	0.314831	-0.011147
24	6	0	-3.224263	-0.180584	-0.269683
25	6	0	-3.655379	0.743707	0.687863
26	6	0	-4.162996	-0.951736	-0.956880
27	6	0	-5.009517	0.891208	0.948539
28	1	0	-2.921112	1.337593	1.219684
29	6	0	-5.521085	-0.802070	-0.694515
30	1	0	-3.822902	-1.668357	-1.697746
31	6	0	-5.944339	0.118998	0.257779
32	1	0	-5.343127	1.607296	1.690676
33	1	0	-6.246048	-1.402264	-1.231656
34	1	0	-7.001833	0.237817	0.464519
35	6	0	-1.793375	-0.363391	-0.575993
36	1	0	-1.558728	-1.125127	-1.329873

Structure 69b (B3LYP, Gas Phase)

Energy (Hartrees): = -936.4769492
No imaginary frequencies

Standard orientation:

Center Number	Atomic Number	Atomic Type	Coordinates (Angstroms) X	Y	Z
1	6	0	1.229075	1.303503	-0.589904
2	6	0	0.534635	-0.024261	-0.186042
3	6	0	1.231172	-0.593678	1.061614
4	6	0	2.734664	-0.731095	0.814886
5	6	0	3.323961	0.595987	0.317644
6	1	0	1.067721	0.095275	1.900287
7	1	0	3.222661	-0.986798	1.768650
8	1	0	3.258357	1.304885	1.155513
9	1	0	0.659162	-0.740091	-1.011446
10	8	0	2.594279	1.114465	-0.800280
11	6	0	4.795474	0.498040	-0.097893
12	1	0	5.150100	1.506319	-0.335204
13	1	0	5.359981	0.151596	0.787265
14	8	0	5.040367	-0.298868	-1.233317
15	1	0	4.527566	-1.113728	-1.117942
16	8	0	2.971654	-1.762587	-0.143123
17	1	0	2.415192	-2.510116	0.123176
18	8	0	0.757854	-1.900929	1.380762
19	1	0	-0.204495	-1.854710	1.462126
20	7	0	-0.857205	0.248170	0.122557
21	6	0	-3.204374	-0.171664	-0.314789
22	6	0	-3.687608	0.701773	0.674651
23	6	0	-4.118681	-0.874166	-1.114085
24	6	0	-5.056292	0.865482	0.854504
25	1	0	-2.974554	1.239699	1.290089
26	6	0	-5.490826	-0.707522	-0.933371
27	1	0	-3.749062	-1.550549	-1.880844
28	6	0	-5.961143	0.162194	0.051216
29	1	0	-5.423755	1.541471	1.620790
30	1	0	-6.190364	-1.254126	-1.558340
31	1	0	-7.029583	0.293266	0.194646
32	6	0	-1.764711	-0.370462	-0.533706
33	1	0	-1.501430	-1.098787	-1.316926
34	1	0	0.836964	1.641607	-1.559401
35	8	0	1.020435	2.286490	0.405865
36	1	0	0.107182	2.164258	0.712051

Structure 69b (B3LYP, DMSO)

Energy (Hartrees): = -936.5013284
No imaginary frequencies

Standard orientation:

Center Number	Atomic Number	Atomic Type	Coordinates (Angstroms) X	Y	Z
1	6	0	1.220673	1.282016	-0.622090
2	6	0	0.531538	-0.046682	-0.221103
3	6	0	1.225026	-0.632612	1.021781
4	6	0	2.733767	-0.738397	0.798984
5	6	0	3.306060	0.606826	0.341638
6	1	0	1.041090	0.032291	1.875703
7	1	0	3.211525	-1.006435	1.752773
8	1	0	3.197751	1.301109	1.185530
9	1	0	0.641409	-0.754883	-1.053547
10	8	0	2.599715	1.121933	-0.798549
11	6	0	4.790835	0.550263	-0.016484
12	1	0	5.133900	1.564826	-0.246618
13	1	0	5.330847	0.221256	0.887663
14	8	0	5.101812	-0.257594	-1.139575
15	1	0	4.581301	-1.070678	-1.031643
16	8	0	3.011259	-1.742938	-0.183404
17	1	0	2.451675	-2.502829	0.043133
18	8	0	0.773371	-1.955778	1.306843
19	1	0	-0.195680	-1.942069	1.330593
20	7	0	-0.857359	0.241455	0.094845
21	6	0	-3.210296	-0.176291	-0.299683
22	6	0	-3.679487	0.732851	0.665396
23	6	0	-4.136209	-0.899715	-1.068025
24	6	0	-5.046404	0.911173	0.852208
25	1	0	-2.963588	1.289831	1.261245
26	6	0	-5.506339	-0.718338	-0.879653
27	1	0	-3.775264	-1.603249	-1.813847
28	6	0	-5.963116	0.186831	0.080330

29	1	0	-5.402890	1.614138	1.599492
30	1	0	-6.214908	-1.281669	-1.479570
31	1	0	-7.029568	0.329142	0.229562
32	6	0	-1.775021	-0.394240	-0.532500
33	1	0	-1.528071	-1.147169	-1.293634
34	1	0	0.846129	1.601054	-1.604830
35	8	0	0.970506	2.282545	0.349961
36	1	0	0.076388	2.097695	0.687078

Structure 69b (M06-2X/6-311G(d,p), Gas Phase)

Energy (Hartrees): = -936.3478136
No imaginary frequencies

Standard orientation:

Center Number	Atomic Number	Atomic Type	Coordinates (Angstroms) X	Y	Z
1	6	0	1.273574	1.309005	-0.629277
2	6	0	0.543369	0.015929	-0.223908
3	6	0	1.182666	-0.533266	1.052642
4	6	0	2.676533	-0.724601	0.835962
5	6	0	3.309804	0.568680	0.326611
6	1	0	1.024677	0.179835	1.868573
7	1	0	3.141033	-0.983940	1.796502
8	1	0	3.243611	1.299294	1.142830
9	1	0	0.671917	-0.725072	-1.022833
10	8	0	2.628198	1.073023	-0.814164
11	6	0	4.777192	0.409365	-0.054118
12	1	0	5.184373	1.400423	-0.263526
13	1	0	5.303528	0.005402	0.824638
14	8	0	4.993508	-0.370807	-1.200025
15	1	0	4.441164	-1.155258	-1.117576
16	8	0	2.890032	-1.768705	-0.101430
17	1	0	2.329635	-2.504763	0.169103
18	8	0	0.658638	-1.808976	1.381327
19	1	0	-0.284984	-1.718760	1.541732
20	7	0	-0.846699	0.322924	0.041853
21	6	0	-3.179542	-0.184451	-0.305657
22	6	0	-3.652478	0.810085	0.555032
23	6	0	-4.087247	-0.998841	-0.981989
24	6	0	-5.015701	0.983105	0.731361
25	1	0	-2.935900	1.434310	1.074364
26	6	0	-5.454872	-0.824103	-0.804665
27	1	0	-3.719226	-1.769418	-1.651641
28	6	0	-5.919017	0.166762	0.051872
29	1	0	-5.380685	1.754249	1.398935
30	1	0	-6.155207	-1.458057	-1.334397
31	1	0	-6.984367	0.305809	0.192103
32	6	0	-1.735632	-0.390303	-0.514965
33	1	0	-1.461854	-1.209662	-1.194564
34	1	0	0.903015	1.660771	-1.597025
35	8	0	1.095098	2.292763	0.361152
36	1	0	0.170469	2.241781	0.631525

Structure 69b (M06-2X/6-311G(d,p), DMSO)

Energy (Hartrees): = -936.3749237
No imaginary frequencies

Standard orientation:

Center Number	Atomic Number	Atomic Type	Coordinates (Angstroms) X	Y	Z
1	6	0	1.259353	1.291823	-0.645885
2	6	0	0.540416	-0.006720	-0.244696
3	6	0	1.182973	-0.570622	1.024514
4	6	0	2.680875	-0.733806	0.819348
5	6	0	3.295187	0.578790	0.344326
6	1	0	1.011263	0.120642	1.856691
7	1	0	3.142765	-1.003800	1.776727
8	1	0	3.197529	1.293377	1.169716
9	1	0	0.655885	-0.736699	-1.054573
10	8	0	2.626486	1.084812	-0.809362
11	6	0	4.772993	0.460516	0.004423
12	1	0	5.164796	1.459725	-0.198963
13	1	0	5.285666	0.074065	0.896669
14	8	0	5.043526	-0.330237	-1.133805
15	1	0	4.478030	-1.108272	-1.063314
16	8	0	2.927137	-1.753501	-0.143268
17	1	0	2.356897	-2.498161	0.084916
18	8	0	0.678763	-1.861161	1.326400
19	1	0	-0.278043	-1.799366	1.430163

20	7	0	-0.847776	0.306346	0.030685
21	6	0	-3.184189	-0.184619	-0.296651
22	6	0	-3.646654	0.810589	0.571008
23	6	0	-4.100744	-0.991432	-0.972606
24	6	0	-5.009195	0.992570	0.754290
25	1	0	-2.929935	1.431758	1.095521
26	6	0	-5.467382	-0.806657	-0.788475
27	1	0	-3.737506	-1.762778	-1.644212
28	6	0	-5.921770	0.184861	0.074539
29	1	0	-5.365993	1.763046	1.427961
30	1	0	-6.174721	-1.434724	-1.317195
31	1	0	-6.985974	0.330792	0.220768
32	6	0	-1.743559	-0.405566	-0.519549
33	1	0	-1.484638	-1.227768	-1.198171
34	1	0	0.895998	1.627906	-1.622016
35	8	0	1.050114	2.289473	0.326679
36	1	0	0.144383	2.172372	0.643849

Structure 69b (M06-2X/def2-TZVP, Gas Phase)

Energy (Hartrees): = -936.4637428
No imaginary frequencies

Standard orientation:

Center Number	Atomic Number	Atomic Type	Coordinates (Angstroms) X	Y	Z
1	6	0	1.260759	1.301059	-0.625990
2	6	0	0.538853	0.004932	-0.219975
3	6	0	1.191134	-0.554588	1.042160
4	6	0	2.682731	-0.739596	0.817770
5	6	0	3.300790	0.565383	0.327063
6	1	0	1.041622	0.151285	1.865053
7	1	0	3.150965	-1.001529	1.774530
8	1	0	3.224151	1.281355	1.154634
9	1	0	0.661387	-0.725319	-1.028630
10	8	0	2.616970	1.078575	-0.804284
11	6	0	4.772485	0.438614	-0.037139
12	1	0	5.161884	1.437472	-0.238702
13	1	0	5.297012	0.043817	0.844674
14	8	0	5.028802	-0.333498	-1.179903
15	1	0	4.486183	-1.129182	-1.129199
16	8	0	2.908513	-1.773040	-0.125209
17	1	0	2.355956	-2.521834	0.129817
18	8	0	0.666429	-1.827972	1.371535
19	1	0	-0.280257	-1.744272	1.525878
20	7	0	-0.847935	0.301046	0.057769
21	6	0	-3.182846	-0.176689	-0.306339
22	6	0	-3.655432	0.782834	0.589464
23	6	0	-4.089447	-0.961012	-1.013357
24	6	0	-5.016110	0.950594	0.769368
25	1	0	-2.941776	1.385484	1.135664
26	6	0	-5.454786	-0.791685	-0.832901
27	1	0	-3.720653	-1.705306	-1.710008
28	6	0	-5.918023	0.164254	0.058538
29	1	0	-5.381411	1.695044	1.464835
30	1	0	-6.153936	-1.403382	-1.387656
31	1	0	-6.982339	0.299467	0.202200
32	6	0	-1.742036	-0.380665	-0.523012
33	1	0	-1.474764	-1.173252	-1.234542
34	1	0	0.890862	1.642964	-1.596989
35	8	0	1.069210	2.295015	0.350566
36	1	0	0.152847	2.226615	0.648510

Structure 69b (M06-2X/def2-TZVP, DMSO)

Energy (Hartrees): = -936.4907441
No imaginary frequencies

Standard orientation:

Center Number	Atomic Number	Atomic Type	Coordinates (Angstroms) X	Y	Z
1	6	0	1.249398	1.285480	-0.645609
2	6	0	0.536334	-0.014814	-0.244085
3	6	0	1.190023	-0.590331	1.010580
4	6	0	2.685771	-0.749251	0.801074
5	6	0	3.286616	0.573982	0.346389
6	1	0	1.027750	0.094668	1.849144
7	1	0	3.148522	-1.021880	1.756128
8	1	0	3.178308	1.272826	1.183222
9	1	0	0.644388	-0.733792	-1.063701
10	8	0	2.617901	1.091068	-0.798374

11	6	0	4.767751	0.485648	0.025601
12	1	0	5.144154	1.491090	-0.170478
13	1	0	5.275769	0.106824	0.921604
14	8	0	5.077199	-0.297448	-1.108036
15	1	0	4.526621	-1.089906	-1.063548
16	8	0	2.946878	-1.758394	-0.165766
17	1	0	2.388057	-2.517154	0.048256
18	8	0	0.680103	-1.876643	1.313418
19	1	0	-0.280770	-1.819875	1.394853
20	7	0	-0.848258	0.289228	0.043583
21	6	0	-3.186872	-0.176866	-0.295617
22	6	0	-3.650215	0.798166	0.589769
23	6	0	-4.101101	-0.968712	-0.986326
24	6	0	-5.010306	0.974236	0.775900
25	1	0	-2.937071	1.410355	1.127650
26	6	0	-5.465482	-0.790607	-0.799318
27	1	0	-3.736408	-1.724630	-1.672807
28	6	0	-5.920260	0.180758	0.081496
29	1	0	-5.368347	1.729969	1.463856
30	1	0	-6.170884	-1.408364	-1.340606
31	1	0	-6.983644	0.322414	0.230425
32	6	0	-1.749593	-0.395618	-0.525281
33	1	0	-1.496556	-1.195063	-1.231140
34	1	0	0.890227	1.610890	-1.626262
35	8	0	1.023404	2.293917	0.311179
36	1	0	0.131427	2.154609	0.661505

Structure 69c (B3LYP, Gas Phase)

Energy (Hartrees): = -936.4800208
No imaginary frequencies

Standard orientation:

Center Number	Atomic Number	Atomic Type	Coordinates (Angstroms) X	Y	Z
1	6	0	4.245064	0.958330	-0.663460
2	6	0	3.229494	0.096024	-0.224372
3	6	0	3.577901	-1.069008	0.479625
4	6	0	4.913265	-1.360490	0.733629
5	6	0	5.919624	-0.494775	0.290850
6	6	0	5.583716	0.665419	-0.407643
7	6	0	1.830216	0.437901	-0.515896
8	7	0	0.840598	-0.294971	-0.176106
9	6	0	-0.502964	0.162990	-0.471976
10	6	0	-1.054781	0.955688	0.747603
11	6	0	-2.579659	1.137358	0.645965
12	6	0	-3.257138	-0.208469	0.404875
13	8	0	-2.744146	-0.742686	-0.837132
14	6	0	-1.370860	-1.080813	-0.788888
15	8	0	-0.415241	2.213161	0.847703
16	8	0	-1.158588	-2.082907	0.169003
17	6	0	-4.771025	-0.155581	0.232842
18	8	0	-5.302872	-1.445044	-0.002047
19	8	0	-2.870800	2.074456	-0.399388
20	1	0	1.677178	1.384423	-1.052063
21	1	0	-0.531219	0.837781	-1.342114
22	1	0	-0.911597	2.793342	0.246513
23	1	0	-0.816261	0.396900	1.658545
24	1	0	-2.880115	1.563562	-1.223957
25	1	0	-2.943093	1.595459	1.572381
26	1	0	-4.751840	-1.835404	-0.696138
27	1	0	-5.227004	0.222540	1.153913
28	1	0	-5.023999	0.546997	-0.575439
29	1	0	-3.012466	-0.901537	1.217191
30	1	0	-0.227402	-1.980117	0.436245
31	1	0	-1.147360	-1.447447	-1.800984
32	1	0	3.979775	1.862667	-1.205364
33	1	0	6.361737	1.340249	-0.751464
34	1	0	6.961605	-0.725408	0.492233
35	1	0	5.175342	-2.262319	1.278939
36	1	0	2.788586	-1.730187	0.820273

Structure 69c (B3LYP, DMSO)

Energy (Hartrees): = -936.5027116
No imaginary frequencies

Standard orientation:

Center Number	Atomic Number	Atomic Type	Coordinates (Angstroms) X	Y	Z

1	6	0	4.248135	0.881359	-0.809175
2	6	0	3.236028	0.089392	-0.243735
3	6	0	3.592025	-0.989889	0.584612
4	6	0	4.931949	-1.267040	0.836631
5	6	0	5.934811	-0.472606	0.267611
6	6	0	5.591020	0.601693	-0.555277
7	6	0	1.833136	0.419007	-0.538526
8	7	0	0.844463	-0.257342	-0.092090
9	6	0	-0.500990	0.179847	-0.415610
10	6	0	-1.092970	0.951268	0.795448
11	6	0	-2.614775	1.126889	0.649543
12	6	0	-3.274653	-0.221881	0.378196
13	8	0	-2.714738	-0.773758	-0.835331
14	6	0	-1.337240	-1.079607	-0.750080
15	8	0	-0.462529	2.213283	0.942236
16	8	0	-1.126425	-2.086448	0.211974
17	6	0	-4.778183	-0.165302	0.146528
18	8	0	-5.309062	-1.459827	-0.108491
19	8	0	-2.889048	2.084423	-0.383533
20	1	0	1.679907	1.297668	-1.178064
21	1	0	-0.522336	0.847793	-1.289701
22	1	0	-0.905539	2.788409	0.295467
23	1	0	-0.887559	0.382084	1.707841
24	1	0	-2.813134	1.613315	-1.229504
25	1	0	-3.010510	1.558898	1.574350
26	1	0	-4.753070	-1.832406	-0.809881
27	1	0	-5.268680	0.216122	1.047706
28	1	0	-5.001351	0.528340	-0.676929
29	1	0	-3.065633	-0.900021	1.212637
30	1	0	-0.215389	-1.941704	0.528046
31	1	0	-1.075531	-1.441072	-1.753796
32	1	0	3.976160	1.717574	-1.448311
33	1	0	6.366164	1.220399	-0.997698
34	1	0	6.979671	-0.691961	0.467636
35	1	0	5.199870	-2.101848	1.477823
36	1	0	2.810356	-1.600365	1.025160

Structure 69c (M06-2X/6-311G(d,p), Gas Phase)

Energy (Hartrees): = -936.3506388
No imaginary frequencies

Standard orientation:

Center Number	Atomic Number	Atomic Type	Coordinates (Angstroms) X	Y	Z
1	6	0	4.207000	0.999712	-0.598029
2	6	0	3.207437	0.103368	-0.222011
3	6	0	3.557477	-1.095849	0.404020
4	6	0	4.889711	-1.389458	0.645997
5	6	0	5.885155	-0.489887	0.266874
6	6	0	5.543620	0.704829	-0.354983
7	6	0	1.800561	0.441978	-0.497517
8	7	0	0.837306	-0.326079	-0.201002
9	6	0	-0.509037	0.131189	-0.473100
10	6	0	-1.031464	0.916572	0.748015
11	6	0	-2.540521	1.137657	0.629726
12	6	0	-3.243441	-0.190930	0.403457
13	8	0	-2.740067	-0.751789	-0.817612
14	6	0	-1.381288	-1.104846	-0.753084
15	8	0	-0.360199	2.148958	0.854758
16	8	0	-1.200951	-2.060646	0.249707
17	6	0	-4.745034	-0.090102	0.216072
18	8	0	-5.302047	-1.362287	-0.013106
19	8	0	-2.790310	2.045610	-0.437623
20	1	0	1.626884	1.412983	-0.976680
21	1	0	-0.547468	0.798484	-1.345557
22	1	0	-0.810631	2.743784	0.241934
23	1	0	-0.812163	0.344686	1.652958
24	1	0	-2.845942	1.519894	-1.244490
25	1	0	-2.903447	1.620434	1.540796
26	1	0	-4.761930	-1.772917	-0.695971
27	1	0	-5.196644	0.308184	1.127094
28	1	0	-4.966782	0.602280	-0.605539
29	1	0	-3.026619	-0.879402	1.225050
30	1	0	-0.265696	-2.014006	0.490533
31	1	0	-1.148442	-1.520153	-1.738944
32	1	0	3.934179	1.932038	-1.081440
33	1	0	6.315463	1.405440	-0.649275
34	1	0	6.926023	-0.722020	0.458831
35	1	0	5.158273	-2.319148	1.132929
36	1	0	2.771330	-1.782162	0.693162

Structure 69c (M06-2X/6-311G(d,p), DMSO)

Energy (Hartrees): = -936.3760965
No imaginary frequencies

Standard orientation:

Center Number	Atomic Number	Atomic Type	Coordinates (Angstroms) X	Y	Z
1	6	0	4.209627	0.958531	-0.697347
2	6	0	3.210951	0.098417	-0.237587
3	6	0	3.565473	-1.053464	0.473102
4	6	0	4.901572	-1.336626	0.715160
5	6	0	5.896076	-0.473825	0.252882
6	6	0	5.549481	0.673856	-0.452379
7	6	0	1.801553	0.429838	-0.518376
8	7	0	0.838633	-0.304915	-0.142900
9	6	0	-0.508783	0.140440	-0.433787
10	6	0	-1.056811	0.907632	0.786350
11	6	0	-2.562656	1.131314	0.638054
12	6	0	-3.255103	-0.197464	0.384077
13	8	0	-2.723122	-0.768976	-0.820027
14	6	0	-1.361891	-1.102530	-0.734865
15	8	0	-0.385307	2.138375	0.935293
16	8	0	-1.184985	-2.075793	0.258052
17	6	0	-4.748532	-0.089593	0.159099
18	8	0	-5.310304	-1.365226	-0.085686
19	8	0	-2.800259	2.060866	-0.414636
20	1	0	1.630546	1.356836	-1.077356
21	1	0	-0.539310	0.804960	-1.307836
22	1	0	-0.763146	2.723736	0.265533
23	1	0	-0.866533	0.323934	1.690535
24	1	0	-2.770517	1.568985	-1.245713
25	1	0	-2.948972	1.588622	1.551839
26	1	0	-4.775371	-1.758233	-0.784464
27	1	0	-5.220522	0.311682	1.058074
28	1	0	-4.950069	0.595898	-0.672632
29	1	0	-3.062342	-0.875953	1.219770
30	1	0	-0.266513	-1.989455	0.550813
31	1	0	-1.102492	-1.502357	-1.719808
32	1	0	3.932149	1.851520	-1.248431
33	1	0	6.319557	1.345978	-0.812561
34	1	0	6.939137	-0.697947	0.445245
35	1	0	5.173118	-2.229399	1.266656
36	1	0	2.786935	-1.717514	0.830373

Structure 69c (M06-2X/6-311G(d,p), H_2O)

Energy (Hartrees): = -936.3848318
No imaginary frequencies

Standard orientation:

Center Number	Atomic Number	Atomic Type	Coordinates (Angstroms) X	Y	Z
1	6	0	4.181646	0.975022	-0.705965
2	6	0	3.216138	0.082688	-0.235645
3	6	0	3.617269	-1.064541	0.457479
4	6	0	4.965382	-1.310554	0.672330
5	6	0	5.925955	-0.415081	0.200343
6	6	0	5.533240	0.728267	-0.487371
7	6	0	1.796878	0.383181	-0.493798
8	7	0	0.844875	-0.359823	-0.096386
9	6	0	-0.503734	0.086780	-0.388096
10	6	0	-1.062890	0.860677	0.816930
11	6	0	-2.555173	1.133820	0.622733
12	6	0	-3.282496	-0.173425	0.352598
13	8	0	-2.724684	-0.797893	-0.814304
14	6	0	-1.367787	-1.144071	-0.690383
15	8	0	-0.355618	2.071618	1.003159
16	8	0	-1.239344	-2.113873	0.324955
17	6	0	-4.758022	-0.001394	0.063363
18	8	0	-5.391301	-1.260852	-0.106726
19	8	0	-2.748383	2.078858	-0.424953
20	1	0	1.604667	1.300566	-1.061144
21	1	0	-0.532752	0.747227	-1.264536
22	1	0	-0.632099	2.658278	0.286052
23	1	0	-0.926091	0.267098	1.723811
24	1	0	-2.671373	1.613018	-1.267788
25	1	0	-2.951773	1.593840	1.530287
26	1	0	-4.927777	-1.707248	-0.824716
27	1	0	-5.236220	0.491812	0.910928
28	1	0	-4.889547	0.622764	-0.826472
29	1	0	-3.158951	-0.837877	1.212769

30	1	0	-0.328976	-2.055773	0.647024
31	1	0	-1.089484	-1.567620	-1.658603
32	1	0	3.868064	1.863598	-1.244182
33	1	0	6.276413	1.425485	-0.855618
34	1	0	6.978068	-0.610867	0.371215
35	1	0	5.272428	-2.200429	1.209078
36	1	0	2.867488	-1.756184	0.822431

Structure 69c (M06-2X/def2-TZVP, Gas Phase)

Energy (Hartrees): = -936.465848
No imaginary frequencies

Standard orientation:

Center Number	Atomic Number	Atomic Type	Coordinates (Angstroms) X	Y	Z
1	6	0	4.208139	0.977247	-0.639367
2	6	0	3.210450	0.099023	-0.227488
3	6	0	3.561461	-1.074410	0.439596
4	6	0	4.891883	-1.360462	0.685401
5	6	0	5.885310	-0.478770	0.269967
6	6	0	5.543213	0.690552	-0.392209
7	6	0	1.805764	0.432533	-0.508930
8	7	0	0.839177	-0.312488	-0.179928
9	6	0	-0.505060	0.134518	-0.459387
10	6	0	-1.043816	0.912414	0.755762
11	6	0	-2.550126	1.132596	0.626642
12	6	0	-3.243280	-0.196004	0.382259
13	8	0	-2.726523	-0.759692	-0.825623
14	6	0	-1.368610	-1.100686	-0.758109
15	8	0	-0.372342	2.141295	0.884749
16	8	0	-1.185169	-2.077013	0.224115
17	6	0	-4.741056	-0.091782	0.186389
18	8	0	-5.314893	-1.363321	-0.000159
19	8	0	-2.803857	2.063181	-0.416751
20	1	0	1.637002	1.382742	-1.028346
21	1	0	-0.540507	0.804382	-1.329095
22	1	0	-0.822985	2.762148	0.296712
23	1	0	-0.840316	0.333772	1.659599
24	1	0	-2.839555	1.571582	-1.247100
25	1	0	-2.920549	1.592615	1.545579
26	1	0	-4.791092	-1.816655	-0.670259
27	1	0	-5.192729	0.341253	1.080253
28	1	0	-4.958528	0.570266	-0.659048
29	1	0	-3.037246	-0.877552	1.212455
30	1	0	-0.257544	-2.018430	0.495456
31	1	0	-1.126331	-1.498080	-1.748546
32	1	0	3.934339	1.890371	-1.155330
33	1	0	6.313435	1.378406	-0.715656
34	1	0	6.925584	-0.705346	0.465581
35	1	0	5.161148	-2.271142	1.204459
36	1	0	2.778283	-1.749124	0.758983

Structure 69c (M06-2X/def2-TZVP, DMSO)

Energy (Hartrees): = -936.4913882
No imaginary frequencies

Standard orientation:

Center Number	Atomic Number	Atomic Type	Coordinates (Angstroms) X	Y	Z
1	6	0	4.208763	0.940000	-0.728420
2	6	0	3.215831	0.093569	-0.240657
3	6	0	3.576096	-1.036597	0.496051
4	6	0	4.911509	-1.311210	0.735827
5	6	0	5.900118	-0.461290	0.245819
6	6	0	5.548201	0.664678	-0.485516
7	6	0	1.807832	0.419730	-0.522059
8	7	0	0.842331	-0.284823	-0.107285
9	6	0	-0.503381	0.146478	-0.409456
10	6	0	-1.077589	0.907069	0.798070
11	6	0	-2.579008	1.127475	0.624919
12	6	0	-3.258315	-0.203581	0.356035
13	8	0	-2.702127	-0.785970	-0.825900
14	6	0	-1.341551	-1.100297	-0.727980
15	8	0	-0.410442	2.135581	0.973766
16	8	0	-1.161208	-2.087399	0.251310
17	6	0	-4.745693	-0.092414	0.109926
18	8	0	-5.331759	-1.368405	-0.065425
19	8	0	-2.813045	2.072202	-0.411891

20	1	0	1.639143	1.319786	-1.122902
21	1	0	-0.530576	0.810001	-1.283541
22	1	0	-0.772808	2.742359	0.312696
23	1	0	-0.910300	0.318809	1.703285
24	1	0	-2.753669	1.611390	-1.260353
25	1	0	-2.980809	1.567304	1.539751
26	1	0	-4.834461	-1.813613	-0.762527
27	1	0	-5.220779	0.366535	0.978018
28	1	0	-4.933536	0.543656	-0.761434
29	1	0	-3.084642	-0.868755	1.206536
30	1	0	-0.252014	-1.989965	0.573129
31	1	0	-1.065077	-1.486750	-1.713132
32	1	0	3.926413	1.816973	-1.300216
33	1	0	6.313929	1.328011	-0.867484
34	1	0	6.943630	-0.679385	0.436818
35	1	0	5.187825	-2.188889	1.306837
36	1	0	2.803187	-1.693444	0.875270

Structure 69.1H$_2$O (M06-2X/6-311G(d,p), Gas Phase)

Energy (Hartrees): = -1012.783814
No imaginary frequencies

Standard orientation:

Center Number	Atomic Number	Atomic Type	Coordinates (Angstroms) X	Y	Z
1	6	0	-4.004455	-1.442824	-0.274477
2	6	0	-3.142686	-0.345022	-0.288600
3	6	0	-3.669781	0.945234	-0.170981
4	6	0	-5.037475	1.121496	-0.022269
5	6	0	-5.890208	0.020148	0.011509
6	6	0	-5.372731	-1.263596	-0.115666
7	6	0	-1.699236	-0.596797	-0.446046
8	7	0	-0.796137	0.252130	-0.169898
9	6	0	0.582462	-0.160687	-0.377339
10	6	0	1.110456	-0.944745	0.839467
11	6	0	2.598455	-1.262670	0.633980
12	6	0	3.378442	-0.000155	0.304209
13	8	0	2.798368	0.591933	-0.867841
14	6	0	1.486170	1.058509	-0.641557
15	8	0	0.387492	-2.140133	1.018433
16	8	0	1.515100	1.936054	0.436955
17	6	0	4.839195	-0.227212	-0.035493
18	8	0	5.472706	0.996671	-0.323388
19	8	0	2.715630	-2.231470	-0.404432
20	1	0	-1.433548	-1.592942	-0.816216
21	1	0	0.647723	-0.820023	-1.254074
22	1	0	0.791477	-2.777367	0.415222
23	1	0	0.977726	-0.334646	1.735444
24	1	0	2.749209	-1.746419	-1.237254
25	1	0	2.988991	-1.733155	1.539964
26	1	0	4.895905	1.456952	-0.941668
27	1	0	5.347507	-0.668610	0.824428
28	1	0	4.920268	-0.928741	-0.875228
29	1	0	3.310349	0.710905	1.131351
30	1	0	0.713166	2.488561	0.443452
31	1	0	1.182086	1.560721	-1.565599
32	1	0	-3.594688	-2.442229	-0.376547
33	1	0	-6.032776	-2.122156	-0.096045
34	1	0	-6.957510	0.165744	0.128713
35	1	0	-5.443389	2.122500	0.060974
36	1	0	-3.012089	1.805230	-0.214434
37	1	0	-1.117141	1.952513	0.502278
38	8	0	-1.028904	2.922783	0.612443
39	1	0	-1.315115	3.121472	1.506734

Structure 69.1H$_2$O (M06-2X/6-311G(d,p), DMSO)

Energy (Hartrees): = -1012.8127452
No imaginary frequencies

Standard orientation:

Center Number	Atomic Number	Atomic Type	Coordinates (Angstroms) X	Y	Z
1	6	0	-4.152465	-1.248305	-0.739980
2	6	0	-3.194379	-0.297988	-0.382249
3	6	0	-3.606309	0.939223	0.126297
4	6	0	-4.957589	1.210335	0.281757
5	6	0	-5.909942	0.252903	-0.068082
6	6	0	-5.507151	-0.976008	-0.579620
7	6	0	-1.770468	-0.633551	-0.562634

8	7	0	-0.828445	0.110673	-0.151691
9	6	0	0.534742	-0.337564	-0.361824
10	6	0	1.146145	-0.760764	0.989990
11	6	0	2.640373	-1.046200	0.829821
12	6	0	3.312811	0.146387	0.170491
13	8	0	2.721341	0.331561	-1.119062
14	6	0	1.366270	0.760427	-1.058063
15	8	0	0.476742	-1.894797	1.492962
16	8	0	1.271659	2.020636	-0.488962
17	6	0	4.798351	-0.024688	-0.070624
18	8	0	5.331419	1.114948	-0.717652
19	8	0	2.814165	-2.232396	0.061141
20	1	0	-1.564613	-1.578361	-1.078637
21	1	0	0.558859	-1.213177	-1.025108
22	1	0	0.825198	-2.644634	0.992821
23	1	0	1.016127	0.039814	1.722478
24	1	0	2.752745	-1.976876	-0.868826
25	1	0	3.075537	-1.239922	1.812926
26	1	0	4.750513	1.284597	-1.468017
27	1	0	5.310534	-0.130585	0.887764
28	1	0	4.975088	-0.932666	-0.659679
29	1	0	3.150973	1.043505	0.777739
30	1	0	0.829707	2.013354	0.376240
31	1	0	1.061234	0.831523	-2.104058
32	1	0	-3.830038	-2.204056	-1.140501
33	1	0	-6.245054	-1.720159	-0.855440
34	1	0	-6.964871	0.469425	0.055575
35	1	0	-5.274140	2.170526	0.672167
36	1	0	-2.868806	1.688941	0.389203
37	1	0	-0.846686	1.434409	1.183039
38	8	0	-0.454274	2.173924	1.688874
39	1	0	-0.403939	1.873393	2.602061

Structure 69.1H_2O (M06-2X/6-311G(d,p), H_2O)

Energy (Hartrees): = -1012.823532
No imaginary frequencies

Standard orientation:

Center Number	Atomic Number	Atomic Type	Coordinates (Angstroms) X	Y	Z
1	6	0	-3.977086	-1.427329	-0.524723
2	6	0	-3.143273	-0.332201	-0.281998
3	6	0	-3.710378	0.892708	0.087754
4	6	0	-5.086953	1.009661	0.215144
5	6	0	-5.912164	-0.089942	-0.019879
6	6	0	-5.355845	-1.309316	-0.390693
7	6	0	-1.693318	-0.537740	-0.436987
8	7	0	-0.800640	0.322717	-0.144067
9	6	0	0.575909	-0.117597	-0.331943
10	6	0	1.109279	-0.818115	0.928704
11	6	0	2.571009	-1.227601	0.707677
12	6	0	3.392458	-0.029010	0.258078
13	8	0	2.803144	0.559235	-0.912798
14	6	0	1.500993	1.053415	-0.695846
15	8	0	0.332030	-1.952138	1.258665
16	8	0	1.574721	2.029273	0.314925
17	6	0	4.817084	-0.373561	-0.119401
18	8	0	5.564152	0.799955	-0.404648
19	8	0	2.638575	-2.306076	-0.221178
20	1	0	-1.410913	-1.517910	-0.834327
21	1	0	0.626850	-0.832081	-1.163452
22	1	0	0.537510	-2.623463	0.593740
23	1	0	1.052569	-0.132394	1.776206
24	1	0	2.571612	-1.945480	-1.114649
25	1	0	2.972377	-1.611121	1.648098
26	1	0	5.096155	1.261477	-1.110069
27	1	0	5.301817	-0.871181	0.721938
28	1	0	4.819857	-1.053199	-0.977579
29	1	0	3.408862	0.710164	1.063878
30	1	0	0.753161	2.554187	0.315418
31	1	0	1.184946	1.498256	-1.642866
32	1	0	-3.536525	-2.374584	-0.818076
33	1	0	-5.993012	-2.164997	-0.579306
34	1	0	-6.986611	0.008116	0.082833
35	1	0	-5.520915	1.961544	0.497190
36	1	0	-3.082173	1.757279	0.260728
37	1	0	-1.071917	2.105816	0.357972
38	8	0	-0.977103	3.074541	0.481574
39	1	0	-1.129612	3.212003	1.423147

Structure 69.5H_2O (M06-2X/6-311G(d,p), Gas Phase)

Energy (Hartrees): = -1318.522736
No imaginary frequencies

Standard orientation:

Center Number	Atomic Number	Atomic Type	Coordinates (Angstroms) X	Y	Z
1	6	0	4.514995	0.454801	-1.284781
2	6	0	3.535354	-0.337047	-0.686555
3	6	0	3.915638	-1.454391	0.062982
4	6	0	5.256702	-1.766252	0.216046
5	6	0	6.231762	-0.966212	-0.378260
6	6	0	5.860694	0.142356	-1.129442
7	6	0	2.119989	0.036920	-0.854067
8	7	0	1.179121	-0.560089	-0.241128
9	6	0	-0.165727	-0.055046	-0.468849
10	6	0	-0.619730	0.789057	0.720675
11	6	0	-2.089732	1.165311	0.522992
12	6	0	-2.879813	-0.139795	0.478521
13	8	0	-2.447111	-0.892150	-0.658493
14	6	0	-1.105804	-1.257904	-0.637580
15	8	0	0.222354	1.926358	0.819281
16	8	0	-0.923271	-2.183158	0.420064
17	6	0	-4.397032	0.076299	0.343459
18	8	0	-5.019986	-0.916898	-0.436673
19	8	0	-2.262914	1.933625	-0.648140
20	1	0	1.909676	0.882130	-1.521825
21	1	0	-0.224293	0.566050	-1.366644
22	1	0	0.050571	2.312704	1.697166
23	1	0	-0.531804	0.196874	1.640285
24	1	0	-2.713148	1.407007	-1.337113
25	1	0	-2.413236	1.761087	1.386233
26	1	0	-4.709934	-1.775998	-0.104947
27	1	0	-4.824031	0.125544	1.352268
28	1	0	-4.590195	1.030448	-0.149016
29	1	0	-2.669907	-0.723956	1.379009
30	1	0	0.024005	-2.200514	0.609547
31	1	0	-0.920156	-1.745323	-1.598154
32	1	0	4.217389	1.319306	-1.869024
33	1	0	6.617389	0.762882	-1.593591
34	1	0	7.280016	-1.211359	-0.256249
35	1	0	5.547873	-2.634292	0.795344
36	1	0	3.146675	-2.077775	0.504141
37	1	0	-3.771493	-3.544072	1.615057
38	8	0	-3.678054	-3.047014	0.801153
39	1	0	-2.727365	-2.948383	0.647585
40	1	0	0.707515	1.334170	3.467298
41	8	0	-0.042299	1.953442	3.504443
42	1	0	0.135549	2.551449	4.232439
43	1	0	1.846121	1.068042	1.698775
44	8	0	2.070710	0.530163	2.472003
45	1	0	2.107226	-0.358131	2.104839
46	8	0	0.364817	2.816873	-1.854116
47	1	0	-0.594084	2.781582	-1.942425
48	1	0	0.456242	2.772545	-0.892735
49	8	0	-3.785148	0.554536	-2.580008
50	1	0	-4.064988	-0.238787	-2.095730
51	1	0	-4.602016	0.972589	-2.861592

Structure 69.5H_2O (M06-2X/6-311G(d,p), DMSO)

Energy (Hartrees): = -1318.5594491
No imaginary frequencies

Standard orientation:

Center Number	Atomic Number	Atomic Type	Coordinates (Angstroms) X	Y	Z
1	6	0	-4.540663	-0.263987	-1.415727
2	6	0	-3.556723	0.394627	-0.676537
3	6	0	-3.926534	1.381013	0.244406
4	6	0	-5.265047	1.698036	0.420455
5	6	0	-6.245203	1.033551	-0.318108
6	6	0	-5.883025	0.053631	-1.235938
7	6	0	-2.147027	0.019917	-0.886295
8	7	0	-1.197116	0.519204	-0.206781
9	6	0	0.147709	0.049577	-0.487400
10	6	0	0.622233	-0.868259	0.639536
11	6	0	2.111248	-1.169341	0.459149
12	6	0	2.843412	0.166438	0.508842
13	8	0	2.414945	0.947037	-0.613338
14	6	0	1.061179	1.283235	-0.590924

15	8	0	-0.182550	-2.036560	0.630464
16	8	0	0.851784	2.158675	0.499882
17	6	0	4.370283	0.023210	0.435009
18	8	0	4.979276	1.108286	-0.236023
19	8	0	2.361445	-1.846107	-0.764215
20	1	0	-1.960732	-0.733063	-1.661047
21	1	0	0.203541	-0.506246	-1.429058
22	1	0	-0.060242	-2.459116	1.502888
23	1	0	0.500659	-0.351343	1.600001
24	1	0	2.816160	-1.249668	-1.390578
25	1	0	2.446808	-1.806154	1.285485
26	1	0	4.578729	1.918269	0.127643
27	1	0	4.748001	-0.080922	1.458205
28	1	0	4.643505	-0.881017	-0.111594
29	1	0	2.571024	0.691675	1.427961
30	1	0	-0.084168	2.102928	0.741265
31	1	0	0.871257	1.805269	-1.532055
32	1	0	-4.248390	-1.027519	-2.129549
33	1	0	-6.643842	-0.461943	-1.810083
34	1	0	-7.290423	1.283382	-0.176228
35	1	0	-5.550236	2.464923	1.131208
36	1	0	-3.159005	1.900598	0.807154
37	1	0	3.608394	3.216507	1.980412
38	8	0	3.529281	3.156581	1.022670
39	1	0	2.598010	2.941676	0.859589
40	1	0	-0.814788	-1.777840	3.318574
41	8	0	-0.074810	-2.408716	3.305278
42	1	0	-0.365116	-3.166386	3.823401
43	1	0	-1.865357	-1.205324	1.550171
44	8	0	-2.121671	-0.786662	2.384221
45	1	0	-2.040303	0.152704	2.180124
46	8	0	0.019704	-2.909087	-2.050702
47	1	0	0.910525	-2.656816	-1.765358
48	1	0	-0.441614	-2.841163	-1.206279
49	8	0	3.957790	-0.311484	-2.554985
50	1	0	4.166894	0.437402	-1.973637
51	1	0	4.807919	-0.730825	-2.725967

Structure 69.5H$_2$O (M06-2X/6-311G(d,p), H$_2$O)

Energy (Hartrees): = -1318.5837843
No imaginary frequencies

Standard orientation:

Center Number	Atomic Number	Atomic Type	Coordinates (Angstroms) X	Y	Z
1	6	0	-4.475605	-0.758667	-1.015943
2	6	0	-3.483401	0.152834	-0.651038
3	6	0	-3.848421	1.418134	-0.178149
4	6	0	-5.188011	1.763318	-0.081738
5	6	0	-6.175530	0.850010	-0.453043
6	6	0	-5.819048	-0.410297	-0.920645
7	6	0	-2.073958	-0.264207	-0.752887
8	7	0	-1.100605	0.522964	-0.526502
9	6	0	0.230687	-0.059124	-0.572107
10	6	0	0.665456	-0.418101	0.851420
11	6	0	2.154538	-0.786006	0.939476
12	6	0	2.969853	0.304250	0.256352
13	8	0	2.523088	0.514618	-1.096420
14	6	0	1.192544	0.950118	-1.203137
15	8	0	-0.152467	-1.494969	1.283122
16	8	0	1.089793	2.220174	-0.596301
17	6	0	4.449036	-0.003974	0.178952
18	8	0	5.174656	1.037770	-0.454356
19	8	0	2.431056	-2.084780	0.449693
20	1	0	-1.906812	-1.310931	-1.033068
21	1	0	0.258803	-0.962852	-1.187445
22	1	0	-0.010735	-1.573162	2.246949
23	1	0	0.500510	0.455313	1.495593
24	1	0	2.363364	-2.094728	-0.520240
25	1	0	2.427212	-0.801093	1.998648
26	1	0	4.720391	1.880663	-0.277522
27	1	0	4.811079	-0.180061	1.199094
28	1	0	4.604357	-0.916005	-0.402500
29	1	0	2.826265	1.232971	0.816931
30	1	0	0.169277	2.342798	-0.324102
31	1	0	0.998393	1.029681	-2.275523
32	1	0	-4.188015	-1.741512	-1.374512
33	1	0	-6.584620	-1.121024	-1.207641
34	1	0	-7.221350	1.122930	-0.374136
35	1	0	-5.468527	2.742860	0.286849
36	1	0	-3.077798	2.120867	0.114758
37	1	0	3.651633	3.633363	0.762909
38	8	0	3.681847	3.415543	-0.175280

39	1	0	2.780499	3.115168	-0.374845
40	1	0	-0.618712	-0.240776	3.669317
41	8	0	0.121361	-0.816632	3.927449
42	1	0	-0.249683	-1.403099	4.595495
43	1	0	-1.810269	-0.378794	1.947830
44	8	0	-2.052672	0.360813	2.524180
45	1	0	-1.835067	1.133743	1.988673
46	8	0	0.004046	-3.418492	-0.972565
47	1	0	0.726685	-3.068988	-1.512514
48	1	0	0.067223	-2.912145	-0.148446
49	8	0	2.257499	-2.055058	-2.367612
50	1	0	2.521726	-1.127787	-2.259389
51	1	0	3.033880	-2.499765	-2.726007

Structure 69.5H$_2$O (M06-2X/def2-TZVP, Gas Phase)

Energy (Hartrees): = -1318.6770995
No imaginary frequencies

Standard orientation:

Center Number	Atomic Number	Atomic Type	Coordinates (Angstroms) X	Y	Z
1	6	0	-4.450518	-0.177897	-1.362896
2	6	0	-3.456324	0.519639	-0.683196
3	6	0	-3.813579	1.528107	0.213717
4	6	0	-5.147960	1.826501	0.426133
5	6	0	-6.137744	1.124326	-0.256492
6	6	0	-5.788600	0.123970	-1.150720
7	6	0	-2.049131	0.146185	-0.905328
8	7	0	-1.096707	0.677812	-0.260366
9	6	0	0.235170	0.160507	-0.496246
10	6	0	0.613938	-0.797124	0.633914
11	6	0	2.081077	-1.198437	0.479572
12	6	0	2.904463	0.081622	0.555751
13	8	0	2.542267	0.922982	-0.537373
14	6	0	1.216585	1.336754	-0.546186
15	8	0	-0.259867	-1.908471	0.575879
16	8	0	1.014264	2.208480	0.548230
17	6	0	4.415865	-0.173390	0.473354
18	8	0	5.109957	0.867653	-0.171694
19	8	0	2.286674	-1.902848	-0.722307
20	1	0	-1.862949	-0.645772	-1.640139
21	1	0	0.304856	-0.388863	-1.439805
22	1	0	-0.186544	-2.386598	1.423676
23	1	0	0.489565	-0.289222	1.598257
24	1	0	2.759662	-1.347929	-1.370481
25	1	0	2.351488	-1.854590	1.315792
26	1	0	4.806191	1.707572	0.212428
27	1	0	4.786840	-0.331002	1.491533
28	1	0	4.611111	-1.081022	-0.097997
29	1	0	2.674739	0.599534	1.491469
30	1	0	0.072871	2.173707	0.772115
31	1	0	1.090139	1.885079	-1.482856
32	1	0	-4.168661	-0.964239	-2.053558
33	1	0	-6.557219	-0.423290	-1.680429
34	1	0	-7.180588	1.360039	-0.087736
35	1	0	-5.423084	2.611160	1.119152
36	1	0	-3.030838	2.067431	0.732424
37	1	0	3.911781	3.812627	1.555641
38	8	0	3.746991	2.912223	1.268219
39	1	0	2.808754	2.859379	1.026431
40	1	0	-1.264824	-1.977446	3.215643
41	8	0	-0.416859	-2.454049	3.246794
42	1	0	-0.565407	-3.257116	3.751072
43	1	0	-2.018021	-1.229390	1.316935
44	8	0	-2.550066	-1.151564	2.124317
45	1	0	-3.145892	-0.410602	1.979415
46	8	0	-0.450686	-2.653624	-2.163940
47	1	0	0.463597	-2.719737	-2.456277
48	1	0	-0.368159	-2.623593	-1.198361
49	8	0	3.930618	-0.395460	-2.555857
50	1	0	4.208637	0.328643	-1.970624
51	1	0	4.743108	-0.786593	-2.887545

Structure 69.5H$_2$O (M06-2X/def2-TZVP, DMSO)

Energy (Hartrees): = -1318.7141926
No imaginary frequencies

Standard orientation:

Center Number	Atomic Number	Atomic Type	Coordinates (Angstroms) X	Y	Z

1	6	0	-4.505715	0.067042	-1.539056
2	6	0	-3.523962	0.548076	-0.676234
3	6	0	-3.890749	1.346664	0.409567
4	6	0	-5.223177	1.652096	0.625995
5	6	0	-6.201302	1.165288	-0.238083
6	6	0	-5.842312	0.374268	-1.320496
7	6	0	-2.120313	0.185133	-0.927758
8	7	0	-1.176625	0.533444	-0.157881
9	6	0	0.168494	0.104103	-0.473841
10	6	0	0.632953	-0.914641	0.564106
11	6	0	2.127500	-1.185161	0.397517
12	6	0	2.841825	0.147866	0.574883
13	8	0	2.430274	1.016622	-0.480971
14	6	0	1.076497	1.344880	-0.460101
15	8	0	-0.164693	-2.074580	0.418339
16	8	0	0.835625	2.142388	0.680405
17	6	0	4.367759	0.016495	0.530221
18	8	0	4.997810	1.162091	-0.003807
19	8	0	2.419544	-1.763676	-0.864421
20	1	0	-1.933125	-0.419809	-1.821646
21	1	0	0.237206	-0.354271	-1.466875
22	1	0	-0.086010	-2.595235	1.241283
23	1	0	0.486343	-0.492045	1.566124
24	1	0	2.871023	-1.116387	-1.439510
25	1	0	2.451734	-1.881429	1.177880
26	1	0	4.583573	1.943183	0.406447
27	1	0	4.713726	-0.192900	1.547303
28	1	0	4.662742	-0.826364	-0.095440
29	1	0	2.543684	0.582751	1.532191
30	1	0	-0.090356	2.018416	0.939796
31	1	0	0.903003	1.932324	-1.364668
32	1	0	-4.217480	-0.550986	-2.381927
33	1	0	-6.600499	-0.004484	-1.994152
34	1	0	-7.242968	1.405206	-0.064089
35	1	0	-5.505222	2.270957	1.468585
36	1	0	-3.124354	1.727553	1.074352
37	1	0	3.572182	3.404250	2.210042
38	8	0	3.496600	3.193787	1.272642
39	1	0	2.572420	2.934167	1.127739
40	1	0	-1.154497	-2.415220	3.031924
41	8	0	-0.329498	-2.931924	3.020979
42	1	0	-0.550029	-3.810666	3.349136
43	1	0	-1.929950	-1.342144	1.292599
44	8	0	-2.424753	-1.353971	2.126899
45	1	0	-2.699608	-0.440171	2.268387
46	8	0	0.247838	-3.010918	-2.290135
47	1	0	1.078672	-2.667649	-1.924308
48	1	0	-0.339700	-2.892241	-1.533913
49	8	0	4.047019	-0.040466	-2.531583
50	1	0	4.239199	0.636747	-1.861460
51	1	0	4.903542	-0.415145	-2.767722

Structure 69.5H₂O (M06-2X/def2-TZVP, H₂O)

Energy (Hartrees): = -1318.738728
No imaginary frequencies

Standard orientation:

Center Number	Atomic Number	Atomic Type	Coordinates (Angstroms) X	Y	Z
1	6	0	-4.423226	-0.267825	-1.228770
2	6	0	-3.424635	0.465084	-0.591155
3	6	0	-3.775502	1.547365	0.219056
4	6	0	-5.107505	1.886040	0.383765
5	6	0	-6.101114	1.150716	-0.258044
6	6	0	-5.758718	0.074967	-1.065274
7	6	0	-2.025897	0.053410	-0.786326
8	7	0	-1.038616	0.676720	-0.288666
9	6	0	0.280785	0.121652	-0.516826
10	6	0	0.734013	-0.640443	0.728059
11	6	0	2.219448	-1.017945	0.672960
12	6	0	3.021712	0.233913	0.353781
13	8	0	2.573561	0.817202	-0.876613
14	6	0	1.245511	1.262241	-0.853003
15	8	0	-0.087512	-1.790578	0.838188
16	8	0	1.138681	2.327439	0.066183
17	6	0	4.503207	-0.023561	0.212623
18	8	0	5.231507	1.168669	-0.031706
19	8	0	2.486890	-2.085823	-0.213299
20	1	0	-1.878650	-0.842680	-1.398970
21	1	0	0.288783	-0.566037	-1.368519
22	1	0	0.027463	-2.152403	1.739810
23	1	0	0.591721	0.003647	1.603961

24	1	0	2.345653	-1.806852	-1.135875
25	1	0	2.511483	-1.370592	1.665149
26	1	0	4.758611	1.923713	0.364199
27	1	0	4.849026	-0.513437	1.129041
28	1	0	4.686726	-0.698126	-0.625700
29	1	0	2.864157	0.950823	1.166204
30	1	0	0.247150	2.314391	0.445350
31	1	0	1.046131	1.627325	-1.862825
32	1	0	-4.146852	-1.110185	-1.852558
33	1	0	-6.529924	-0.497412	-1.564435
34	1	0	-7.141744	1.418422	-0.125214
35	1	0	-5.377199	2.724920	1.012591
36	1	0	-2.999860	2.115407	0.716627
37	1	0	3.725431	3.697553	1.652560
38	8	0	3.718797	3.451850	0.720086
39	1	0	2.810057	3.154935	0.542020
40	1	0	-0.969004	-1.696282	3.504863
41	8	0	-0.107268	-2.136811	3.598661
42	1	0	-0.307082	-3.014950	3.943401
43	1	0	-1.859192	-1.139200	1.619149
44	8	0	-2.445082	-1.026681	2.385342
45	1	0	-2.845449	-0.155786	2.271791
46	8	0	-0.054146	-3.174004	-1.757710
47	1	0	0.598522	-2.658151	-2.253128
48	1	0	-0.029459	-2.793201	-0.863724
49	8	0	2.102437	-1.368590	-2.951554
50	1	0	2.365056	-0.472438	-2.692870
51	1	0	2.847377	-1.715500	-3.457940

Structure 82a (B3LYP, Gas Phase)

Energy (Hartrees): = -936.4729931
No imaginary frequencies

Standard orientation:

Center Number	Atomic Number	Atomic Type	Coordinates (Angstroms) X	Y	Z
1	6	0	-2.574590	-0.950934	-0.082207
2	8	0	-2.394743	-0.694830	1.319164
3	6	0	-1.113831	-0.097979	1.541220
4	6	0	-0.230063	-0.820812	0.486805
5	6	0	-1.172727	-0.907838	-0.729466
6	1	0	-2.987381	-1.960688	-0.193465
7	1	0	-0.812999	-0.350057	2.561823
8	1	0	-0.074899	-1.851437	0.850637
9	1	0	-1.052679	-0.001257	-1.333605
10	7	0	1.002394	-0.144985	0.175691
11	6	0	3.417481	-0.172043	0.106846
12	6	0	3.540293	1.136302	-0.390877
13	6	0	4.575746	-0.928181	0.336978
14	6	0	4.797640	1.669288	-0.652313
15	1	0	2.636456	1.712292	-0.558522
16	6	0	5.835913	-0.392207	0.073615
17	1	0	4.485009	-1.940280	0.724288
18	6	0	5.948688	0.907255	-0.421888
19	1	0	4.886121	2.682012	-1.034849
20	1	0	6.726675	-0.986266	0.255348
21	1	0	6.929019	1.327687	-0.626710
22	6	0	2.099167	-0.761549	0.390730
23	1	0	2.117190	-1.782843	0.808107
24	8	0	-0.994358	-2.069963	-1.526907
25	1	0	-0.170823	-1.956387	-2.019943
26	6	0	-3.549512	0.050569	-0.706966
27	1	0	-3.751242	-0.274667	-1.739967
28	6	0	-4.866035	0.134600	0.058542
29	1	0	-5.415296	-0.813900	-0.025043
30	1	0	-4.646226	0.326174	1.114686
31	8	0	-2.942237	1.345251	-0.731683
32	1	0	-3.681905	1.970006	-0.811713
33	8	0	-5.599070	1.215604	-0.529527
34	1	0	-6.260729	1.516905	0.104420
35	8	0	-1.139772	1.287360	1.465408
36	1	0	-1.630631	1.531412	0.656908

Structure 82a (B3LYP, DMSO)

Energy (Hartrees): = -936.4977694
No imaginary frequencies

Standard orientation:

Center Number	Atomic Number	Atomic Type	Coordinates (Angstroms) X	Y	Z
1	6	0	-2.578629	-0.945576	-0.094094
2	8	0	-2.393837	-0.726721	1.319161
3	6	0	-1.116132	-0.133126	1.547786
4	6	0	-0.229499	-0.825606	0.480153
5	6	0	-1.173667	-0.909815	-0.737133
6	1	0	-3.012245	-1.943135	-0.224988
7	1	0	-0.806550	-0.402838	2.561853
8	1	0	-0.057035	-1.856184	0.829915
9	1	0	-1.048879	-0.008803	-1.348989
10	7	0	0.999011	-0.128551	0.181800
11	6	0	3.416818	-0.164192	0.086823
12	6	0	3.554487	1.143168	-0.412795
13	6	0	4.569966	-0.925244	0.334864
14	6	0	4.818322	1.672284	-0.655439
15	1	0	2.662511	1.730668	-0.605682
16	6	0	5.836532	-0.393115	0.091333
17	1	0	4.468468	-1.936580	0.720578
18	6	0	5.963044	0.906052	-0.404203
19	1	0	4.916014	2.683027	-1.041261
20	1	0	6.721849	-0.990701	0.288115
21	1	0	6.948131	1.322511	-0.594196
22	6	0	2.097475	-0.758329	0.360550
23	1	0	2.116396	-1.791442	0.738304
24	8	0	-0.994694	-2.075904	-1.531409
25	1	0	-0.164143	-1.964523	-2.018233
26	6	0	-3.538997	0.078761	-0.699686
27	1	0	-3.702674	-0.203560	-1.751070
28	6	0	-4.882325	0.127219	0.017834
29	1	0	-5.421384	-0.819462	-0.122597
30	1	0	-4.715946	0.282024	1.089579
31	8	0	-2.939229	1.382263	-0.654263
32	1	0	-3.689448	2.001074	-0.696622
33	8	0	-5.600771	1.228222	-0.555006
34	1	0	-6.254876	1.526957	0.092306
35	8	0	-1.156035	1.259157	1.496082
36	1	0	-1.701104	1.499824	0.717910

Structure 82a (M06-2X/6-311G(d,p), Gas Phase)

Energy (Hartrees): = -936.3409675
No imaginary frequencies

Standard orientation:

Center Number	Atomic Number	Atomic Type	Coordinates (Angstroms) X	Y	Z
1	6	0	-2.528871	-0.916622	-0.012895
2	8	0	-2.390347	-0.538923	1.356269
3	6	0	-1.120323	0.058556	1.566223
4	6	0	-0.223502	-0.724098	0.585383
5	6	0	-1.131665	-0.823760	-0.640281
6	1	0	-2.866370	-1.956868	-0.056013
7	1	0	-0.843727	-0.111431	2.606451
8	1	0	-0.096839	-1.741902	0.986162
9	1	0	-1.033913	0.095167	-1.227396
10	7	0	1.023036	-0.085589	0.270345
11	6	0	3.419132	-0.191556	0.118109
12	6	0	3.552035	1.118826	-0.349663
13	6	0	4.554609	-0.983113	0.282021
14	6	0	4.807160	1.622070	-0.651824
15	1	0	2.656977	1.719112	-0.460466
16	6	0	5.813465	-0.477044	-0.022258
17	1	0	4.450506	-1.999067	0.649438
18	6	0	5.939955	0.825367	-0.490097
19	1	0	4.908627	2.638433	-1.013157
20	1	0	6.692560	-1.096869	0.106365
21	1	0	6.919847	1.222623	-0.727043
22	6	0	2.090112	-0.746119	0.438311
23	1	0	2.079884	-1.773066	0.833778
24	8	0	-0.902516	-1.960583	-1.443303
25	1	0	-0.081535	-1.819062	-1.922209
26	6	0	-3.542680	-0.027105	-0.715505
27	1	0	-3.688412	-0.411941	-1.734197
28	6	0	-4.871710	-0.003563	0.013960
29	1	0	-5.335333	-0.996154	-0.020164
30	1	0	-4.688810	0.282870	1.053569
31	8	0	-3.019189	1.292216	-0.775204
32	1	0	-3.772196	1.873089	-0.937096
33	8	0	-5.667574	0.964754	-0.660296
34	1	0	-6.413942	1.196148	-0.104819
35	8	0	-1.139672	1.427343	1.370597

36	1	0	-1.640491	1.608553	0.560976

Structure 82a (M06-2X/6-311G(d,p), DMSO)

Energy (Hartrees): = -936.3683686
No imaginary frequencies

Standard orientation:

Center Number	Atomic Number	Atomic Type	Coordinates (Angstroms) X	Y	Z
1	6	0	-2.526380	-0.909512	-0.027915
2	8	0	-2.392731	-0.576395	1.359398
3	6	0	-1.127667	0.018392	1.588179
4	6	0	-0.221581	-0.725172	0.591820
5	6	0	-1.123426	-0.798603	-0.641953
6	1	0	-2.872122	-1.944369	-0.103049
7	1	0	-0.848757	-0.175350	2.624118
8	1	0	-0.082603	-1.749154	0.966819
9	1	0	-1.024607	0.131628	-1.211547
10	7	0	1.024592	-0.064795	0.303908
11	6	0	3.418563	-0.188282	0.094556
12	6	0	3.567069	1.124154	-0.366572
13	6	0	4.548767	-0.988472	0.266552
14	6	0	4.829732	1.623847	-0.648840
15	1	0	2.684493	1.738920	-0.499813
16	6	0	5.815244	-0.485736	-0.015829
17	1	0	4.431990	-2.006538	0.624326
18	6	0	5.956655	0.820106	-0.473605
19	1	0	4.940412	2.640608	-1.007780
20	1	0	6.689013	-1.112031	0.121611
21	1	0	6.942246	1.213579	-0.694850
22	6	0	2.088023	-0.749049	0.402865
23	1	0	2.075059	-1.797863	0.726378
24	8	0	-0.880436	-1.914594	-1.470490
25	1	0	-0.051714	-1.755713	-1.936461
26	6	0	-3.533442	0.000791	-0.711198
27	1	0	-3.635431	-0.334414	-1.752162
28	6	0	-4.889657	-0.032552	-0.037910
29	1	0	-5.329404	-1.031283	-0.132041
30	1	0	-4.769600	0.215048	1.020757
31	8	0	-3.030462	1.334594	-0.688832
32	1	0	-3.794623	1.910006	-0.824843
33	8	0	-5.679226	0.948583	-0.704236
34	1	0	-6.441353	1.151432	-0.153338
35	8	0	-1.164687	1.397830	1.421734
36	1	0	-1.716212	1.579426	0.643012

Structure 82a* (B3LYP, Gas Phase)

Energy (Hartrees): = -936.4672858
No imaginary frequencies

Standard orientation:

Center Number	Atomic Number	Atomic Type	Coordinates (Angstroms) X	Y	Z
1	6	0	2.549396	0.821297	0.191737
2	8	0	2.425836	0.121319	1.438486
3	6	0	1.095574	-0.319758	1.659560
4	6	0	0.229439	0.623266	0.794187
5	6	0	1.146240	0.842723	-0.431054
6	1	0	2.852156	1.856657	0.405962
7	1	0	0.918292	-0.206375	2.736447
8	1	0	0.109039	1.591321	1.304041
9	1	0	1.037187	-0.004707	-1.115251
10	7	0	-1.023937	0.015097	0.421065
11	6	0	-3.415577	0.203732	0.087060
12	6	0	-3.592081	-1.105147	-0.393212
13	6	0	-4.531340	1.046585	0.199776
14	6	0	-4.858568	-1.555033	-0.748845
15	1	0	-2.725183	-1.751182	-0.479856
16	6	0	-5.800629	0.594056	-0.157477
17	1	0	-4.399723	2.060286	0.570297
18	6	0	-5.966048	-0.707543	-0.632173
19	1	0	-4.987684	-2.567951	-1.118559
20	1	0	-6.657482	1.254610	-0.065484
21	1	0	-6.953628	-1.062943	-0.911154
22	6	0	-2.092069	0.714226	0.473103
23	1	0	-2.070999	1.759018	0.819927
24	8	0	0.930072	2.075580	-1.097558

25	1	0	0.237568	1.937866	-1.755666
26	6	0	3.613233	0.197522	-0.712962
27	1	0	3.809602	0.924555	-1.520894
28	6	0	4.917691	-0.057095	0.036902
29	1	0	5.348177	0.889548	0.395330
30	1	0	4.703430	-0.698558	0.898735
31	8	0	3.130882	-1.019868	-1.254183
32	1	0	3.921322	-1.483294	-1.571340
33	8	0	5.793035	-0.704942	-0.894212
34	1	0	6.462567	-1.186165	-0.393950
35	8	0	0.925132	-1.661274	1.300020
36	1	0	0.151697	-1.697886	0.713926

Structure 82a* (B3LYP, DMSO)

Energy (Hartrees): = -936.4922007
No imaginary frequencies

Standard orientation:

Center Number	Atomic Number	Atomic Type	Coordinates (Angstroms) X	Y	Z
1	6	0	2.535432	0.621864	0.413144
2	8	0	2.399134	-0.484191	1.323525
3	6	0	1.038945	-0.867158	1.478223
4	6	0	0.216553	0.308017	0.905361
5	6	0	1.151983	0.814945	-0.215045
6	1	0	2.782766	1.526154	0.989628
7	1	0	0.878608	-1.019226	2.553289
8	1	0	0.120537	1.101712	1.659440
9	1	0	1.048418	0.162988	-1.089538
10	7	0	-1.055286	-0.145091	0.390622
11	6	0	-3.436585	0.210429	0.118780
12	6	0	-3.670373	-0.940483	-0.655091
13	6	0	-4.516175	1.047487	0.444189
14	6	0	-4.957314	-1.243385	-1.088216
15	1	0	-2.837432	-1.587876	-0.909705
16	6	0	-5.805505	0.741927	0.008583
17	1	0	-4.338514	1.938449	1.041094
18	6	0	-6.027948	-0.403553	-0.758074
19	1	0	-5.130868	-2.134408	-1.684620
20	1	0	-6.633664	1.395524	0.266158
21	1	0	-7.031113	-0.644136	-1.098073
22	6	0	-2.096461	0.566528	0.607404
23	1	0	-2.038679	1.497009	1.188059
24	8	0	0.964506	2.175014	-0.564751
25	1	0	0.200399	2.219749	-1.158180
26	6	0	3.665582	0.400838	-0.587357
27	1	0	3.796813	1.358382	-1.120818
28	6	0	4.983956	0.057066	0.097687
29	1	0	5.295202	0.873620	0.764617
30	1	0	4.853950	-0.852958	0.694137
31	8	0	3.324477	-0.628845	-1.506669
32	1	0	4.170601	-0.878260	-1.912421
33	8	0	5.938792	-0.149346	-0.951474
34	1	0	6.647768	-0.706448	-0.601268
35	8	0	0.766760	-2.066223	0.797833
36	1	0	-0.039687	-1.893048	0.278597

Structure 82a* (M06-2X/6-311G(d,p), Gas Phase)

Energy (Hartrees): = -936.3351683
No imaginary frequencies

Standard orientation:

Center Number	Atomic Number	Atomic Type	Coordinates (Angstroms) X	Y	Z
1	6	0	-2.503360	-0.777180	0.312192
2	8	0	-2.443421	0.107782	1.430702
3	6	0	-1.147424	0.635311	1.589772
4	6	0	-0.236972	-0.411642	0.934870
5	6	0	-1.092957	-0.817844	-0.271969
6	1	0	-2.759107	-1.780153	0.675694
7	1	0	-0.990033	0.747231	2.665527
8	1	0	-0.140025	-1.288572	1.588803
9	1	0	-0.999701	-0.059205	-1.052799
10	7	0	1.028995	0.128454	0.515796
11	6	0	3.384493	-0.198671	0.123029
12	6	0	3.593953	1.068691	-0.427927
13	6	0	4.456663	-1.082283	0.242375
14	6	0	4.860063	1.440664	-0.850400
15	1	0	2.752444	1.744774	-0.515171

16	6	0	5.726581	-0.708391	-0.182331
17	1	0	4.294129	-2.066200	0.670754
18	6	0	5.928589	0.553182	-0.728818
19	1	0	5.019278	2.423738	-1.277191
20	1	0	6.554916	-1.399694	-0.086431
21	1	0	6.916960	0.847928	-1.060601
22	6	0	2.048624	-0.621769	0.579050
23	1	0	1.979788	-1.640656	0.984011
24	8	0	-0.794022	-2.106405	-0.759406
25	1	0	-0.171338	-2.019553	-1.483578
26	6	0	-3.557966	-0.341192	-0.691759
27	1	0	-3.718206	-1.179888	-1.386667
28	6	0	-4.870846	-0.019286	-0.003504
29	1	0	-5.261418	-0.908489	0.505760
30	1	0	-4.687078	0.772943	0.727098
31	8	0	-3.097518	0.791221	-1.393457
32	1	0	-3.878912	1.190487	-1.790664
33	8	0	-5.752549	0.416970	-1.032864
34	1	0	-6.495163	0.871064	-0.631516
35	8	0	-1.031715	1.873056	0.960196
36	1	0	-0.202343	1.866562	0.467555

Structure 82a* (M06-2X, DMSO)

Energy (Hartrees): = -936.3625345
No imaginary frequencies

Standard orientation:

Center Number	Atomic Number	Atomic Type	Coordinates (Angstroms) X	Y	Z
1	6	0	2.503056	0.621822	0.426490
2	8	0	2.409952	-0.490204	1.321583
3	6	0	1.077818	-0.939291	1.440644
4	6	0	0.222953	0.229651	0.931902
5	6	0	1.117832	0.769548	-0.189952
6	1	0	2.723968	1.526523	1.007273
7	1	0	0.913446	-1.170112	2.496088
8	1	0	0.138007	0.999893	1.708283
9	1	0	1.031757	0.119713	-1.066102
10	7	0	-1.053943	-0.208616	0.426500
11	6	0	-3.411043	0.206893	0.133517
12	6	0	-3.652457	-0.929731	-0.645317
13	6	0	-4.469134	1.058247	0.457725
14	6	0	-4.937034	-1.206566	-1.089101
15	1	0	-2.827388	-1.586511	-0.895752
16	6	0	-5.756978	0.778808	0.012373
17	1	0	-4.276915	1.940157	1.060572
18	6	0	-5.991194	-0.353055	-0.760979
19	1	0	-5.123041	-2.088037	-1.691834
20	1	0	-6.574897	1.442259	0.267682
21	1	0	-6.993690	-0.573462	-1.109553
22	6	0	-2.063120	0.532636	0.633919
23	1	0	-1.981777	1.462244	1.210172
24	8	0	0.876393	2.115728	-0.521704
25	1	0	0.110249	2.145836	-1.105016
26	6	0	3.614857	0.434969	-0.587010
27	1	0	3.723184	1.391423	-1.120603
28	6	0	4.933422	0.109815	0.085352
29	1	0	5.221468	0.919671	0.765317
30	1	0	4.822401	-0.819770	0.651105
31	8	0	3.274212	-0.594248	-1.495585
32	1	0	4.102260	-0.839815	-1.924678
33	8	0	5.881321	-0.046081	-0.966908
34	1	0	6.656542	-0.491118	-0.612334
35	8	0	0.872434	-2.091951	0.676306
36	1	0	0.063666	-1.947013	0.166957

Structure 82b (B3LYP, Gas Phase)

Energy (Hartrees): = -936.4712532
No imaginary frequencies

Standard orientation:

Center Number	Atomic Number	Atomic Type	Coordinates (Angstroms) X	Y	Z
1	6	0	2.659732	0.547661	-0.604924
2	8	0	2.347257	1.560222	0.338461
3	6	0	1.075260	2.127823	-0.040860
4	6	0	0.322567	0.993423	-0.828700
5	6	0	1.324177	-0.175096	-0.844741
6	1	0	2.977888	0.977574	-1.570275

7	1	0	1.272609	2.993664	-0.686454
8	1	0	0.140146	1.300980	-1.867645
9	1	0	1.100992	-0.825877	0.013616
10	7	0	-0.899482	0.637763	-0.133182
11	6	0	-3.203347	-0.101800	-0.236955
12	6	0	-3.380126	-0.212418	1.152646
13	6	0	-4.279237	-0.395355	-1.087729
14	6	0	-4.608567	-0.603458	1.673167
15	1	0	-2.543007	0.009647	1.805592
16	6	0	-5.510633	-0.787301	-0.564611
17	1	0	-4.145885	-0.314740	-2.163655
18	6	0	-5.677193	-0.891276	0.816719
19	1	0	-4.738203	-0.686508	2.748246
20	1	0	-6.336908	-1.011216	-1.232529
21	1	0	-6.635107	-1.196782	1.227336
22	6	0	-1.921616	0.313212	-0.826027
23	1	0	-1.888958	0.326410	-1.925434
24	8	0	1.261450	-0.887000	-2.059817
25	1	0	2.013523	-1.500845	-2.027236
26	6	0	3.790672	-0.334917	-0.076784
27	1	0	4.745287	0.206685	-0.157469
28	6	0	3.627197	-0.755886	1.381411
29	1	0	3.715003	0.119120	2.034885
30	1	0	2.642344	-1.214876	1.541568
31	8	0	3.819749	-1.492994	-0.916211
32	1	0	4.355652	-2.141893	-0.433350
33	8	0	4.673542	-1.706655	1.611946
34	1	0	4.516390	-2.138188	2.460039
35	8	0	0.397743	2.579585	1.071027
36	1	0	-0.206642	1.859566	1.326119

Structure 82b (B3LYP, DMSO)

Energy (Hartrees): = -936.4942975
No imaginary frequencies

Standard orientation:

Center Number	Atomic Number	Atomic Type	Coordinates (Angstroms) X	Y	Z
1	6	0	2.654389	0.619638	-0.529110
2	8	0	2.304995	1.544991	0.493911
3	6	0	1.018217	2.096214	0.141989
4	6	0	0.305977	1.005437	-0.738278
5	6	0	1.344339	-0.127368	-0.822818
6	1	0	2.949572	1.145053	-1.452966
7	1	0	1.187944	3.014189	-0.434626
8	1	0	0.122266	1.393194	-1.748686
9	1	0	1.139112	-0.841505	-0.012425
10	7	0	-0.911353	0.566984	-0.080993
11	6	0	-3.237355	-0.085776	-0.246380
12	6	0	-3.396756	-0.402624	1.114600
13	6	0	-4.334064	-0.216917	-1.112920
14	6	0	-4.629577	-0.834930	1.592937
15	1	0	-2.547965	-0.308620	1.784318
16	6	0	-5.569154	-0.650821	-0.631234
17	1	0	-4.213029	0.024419	-2.165880
18	6	0	-5.719038	-0.959567	0.722123
19	1	0	-4.745764	-1.077932	2.645175
20	1	0	-6.411273	-0.747762	-1.310022
21	1	0	-6.679647	-1.298079	1.099575
22	6	0	-1.955172	0.380659	-0.795200
23	1	0	-1.944336	0.564893	-1.878140
24	8	0	1.324233	-0.768738	-2.083490
25	1	0	2.119451	-1.328848	-2.077598
26	6	0	3.828030	-0.254288	-0.088165
27	1	0	4.746184	0.352713	-0.084215
28	6	0	3.681360	-0.877924	1.296058
29	1	0	3.698742	-0.098271	2.066513
30	1	0	2.736211	-1.428132	1.379262
31	8	0	3.942605	-1.290617	-1.073612
32	1	0	4.463770	-1.986619	-0.638719
33	8	0	4.796463	-1.771135	1.427029
34	1	0	4.589208	-2.404719	2.128087
35	8	0	0.316544	2.439448	1.282012
36	1	0	-0.256873	1.675147	1.478880

Structure 82b (M06-2X/6-311G(d,p), Gas Phase)

Energy (Hartrees): = -936.3389692
No imaginary frequencies

Standard orientation:

Center Number	Atomic Number	Atomic Type	Coordinates (Angstroms) X	Y	Z
1	6	0	-2.650615	0.579320	0.558267
2	8	0	-2.339502	1.486070	-0.475586
3	6	0	-1.115741	2.129202	-0.107709
4	6	0	-0.338252	1.081723	0.753053
5	6	0	-1.313222	-0.091452	0.877010
6	1	0	-2.983990	1.103093	1.467535
7	1	0	-1.365326	3.015291	0.484392
8	1	0	-0.147635	1.471050	1.759842
9	1	0	-1.077172	-0.825079	0.095775
10	7	0	0.878600	0.677068	0.081259
11	6	0	3.160497	-0.080722	0.242973
12	6	0	3.324186	-0.286660	-1.129539
13	6	0	4.229470	-0.319045	1.105881
14	6	0	4.543158	-0.720149	-1.625705
15	1	0	2.484326	-0.102731	-1.788237
16	6	0	5.452561	-0.752978	0.607474
17	1	0	4.100287	-0.162066	2.171822
18	6	0	5.609772	-0.953491	-0.758438
19	1	0	4.667723	-0.879449	-2.690119
20	1	0	6.279050	-0.934820	1.283578
21	1	0	6.561351	-1.293359	-1.149809
22	6	0	1.879352	0.387142	0.801113
23	1	0	1.835488	0.472043	1.895074
24	8	0	-1.242888	-0.665405	2.154758
25	1	0	-1.957098	-1.314099	2.185488
26	6	0	-3.739218	-0.379632	0.104079
27	1	0	-4.720406	0.106309	0.168739
28	6	0	-3.552039	-0.860835	-1.323821
29	1	0	-3.688983	-0.026844	-2.015784
30	1	0	-2.546994	-1.276761	-1.454517
31	8	0	-3.689349	-1.483808	0.997901
32	1	0	-4.220922	-2.178530	0.592651
33	8	0	-4.540844	-1.870685	-1.498774
34	1	0	-4.409501	-2.293745	-2.348816
35	8	0	-0.442594	2.553260	-1.224086
36	1	0	0.141558	1.830235	-1.487960

Structure 82b (M06-2X/6-311G(d,p), DMSO)

Energy (Hartrees): = -936.3643529
No imaginary frequencies

Standard orientation:

Center Number	Atomic Number	Atomic Type	Coordinates (Angstroms) X	Y	Z
1	6	0	2.641944	0.634374	-0.528758
2	8	0	2.310250	1.493572	0.543975
3	6	0	1.028857	2.057894	0.252132
4	6	0	0.309019	1.026027	-0.675222
5	6	0	1.334385	-0.097918	-0.829009
6	1	0	2.917350	1.209890	-1.425223
7	1	0	1.181604	3.008193	-0.266718
8	1	0	0.123140	1.467418	-1.660688
9	1	0	1.147016	-0.850072	-0.052614
10	7	0	-0.903043	0.542684	-0.048684
11	6	0	-3.227006	-0.057672	-0.245385
12	6	0	-3.355467	-0.512191	1.071365
13	6	0	-4.336678	-0.075472	-1.091936
14	6	0	-4.579756	-0.976618	1.529107
15	1	0	-2.489695	-0.498591	1.723402
16	6	0	-5.564674	-0.539484	-0.630687
17	1	0	-4.233455	0.277443	-2.113120
18	6	0	-5.686464	-0.990745	0.679210
19	1	0	-4.676301	-1.330596	2.548968
20	1	0	-6.423070	-0.549447	-1.292122
21	1	0	-6.641456	-1.354588	1.040923
22	6	0	-1.943344	0.447211	-0.766950
23	1	0	-1.939184	0.742151	-1.823347
24	8	0	1.279069	-0.667549	-2.113041
25	1	0	2.049404	-1.248729	-2.161948
26	6	0	3.806022	-0.264381	-0.144312
27	1	0	4.739624	0.311073	-0.172424
28	6	0	3.677771	-0.879087	1.236771
29	1	0	3.723792	-0.097459	1.999141
30	1	0	2.730890	-1.420430	1.332114
31	8	0	3.846810	-1.293263	-1.129603
32	1	0	4.405284	-1.989172	-0.760691
33	8	0	4.778897	-1.778394	1.341980
34	1	0	4.640515	-2.338178	2.111644
35	8	0	0.357006	2.332596	1.421793
36	1	0	-0.138069	1.534981	1.654702

Structure 82c (B3LYP, Gas Phase)

Energy (Hartrees): = -936.479231
No imaginary frequencies

Standard orientation:

Center Number	Atomic Number	Atomic Type	Coordinates (Angstroms) X	Y	Z
1	6	0	2.588472	0.905039	0.023283
2	8	0	2.435888	0.503598	1.406146
3	6	0	1.140691	0.018919	1.626367
4	6	0	0.247587	0.827799	0.666518
5	6	0	1.168276	0.940606	-0.577152
6	1	0	3.008676	1.917455	0.016983
7	1	0	0.916515	0.168280	2.688116
8	1	0	0.097712	1.840862	1.068461
9	1	0	1.017360	0.058892	-1.209562
10	7	0	-0.984015	0.132778	0.378918
11	6	0	-3.385914	0.194274	0.058536
12	6	0	-3.522286	-1.167646	-0.260919
13	6	0	-4.529720	1.006185	0.095903
14	6	0	-4.777617	-1.699844	-0.531801
15	1	0	-2.634534	-1.789908	-0.292745
16	6	0	-5.787602	0.470927	-0.176190
17	1	0	-4.429086	2.060445	0.341804
18	6	0	-5.913146	-0.882930	-0.489676
19	1	0	-4.876289	-2.752929	-0.777534
20	1	0	-6.666454	1.107529	-0.143370
21	1	0	-6.891833	-1.302874	-0.702016
22	6	0	-2.078558	0.793485	0.356999
23	1	0	-2.091820	1.873393	0.569919
24	8	0	0.986009	2.137594	-1.313570
25	1	0	0.276413	1.990436	-1.951437
26	6	0	3.534234	-0.050797	-0.708845
27	1	0	3.646799	0.314152	-1.738403
28	6	0	4.924889	-0.134383	-0.069748
29	1	0	5.429455	0.837647	-0.118701
30	1	0	4.814734	-0.405154	0.991606
31	8	0	2.990909	-1.366120	-0.818680
32	1	0	2.542750	-1.576976	0.024198
33	8	0	5.727343	-1.066942	-0.770498
34	1	0	5.143856	-1.828399	-0.916040
35	8	0	1.074640	-1.366945	1.313209
36	1	0	0.209710	-1.501563	0.883773

Structure 82c (B3LYP, DMSO)

Energy (Hartrees): = = -936.5028296
No imaginary frequencies

Standard orientation:

Center Number	Atomic Number	Atomic Type	Coordinates (Angstroms) X	Y	Z
1	6	0	2.594993	0.903513	0.020638
2	8	0	2.443984	0.495256	1.404468
3	6	0	1.139631	0.031043	1.627467
4	6	0	0.251689	0.851063	0.675808
5	6	0	1.171559	0.984269	-0.567493
6	1	0	3.047584	1.901276	0.017981
7	1	0	0.918746	0.179290	2.689355
8	1	0	0.091469	1.852963	1.097094
9	1	0	0.991833	0.130811	-1.231089
10	7	0	-0.974222	0.145395	0.378369
11	6	0	-3.374902	0.188517	0.055391
12	6	0	-3.509077	-1.181051	-0.235433
13	6	0	-4.519162	1.002474	0.063997
14	6	0	-4.762709	-1.718775	-0.508447
15	1	0	-2.625911	-1.811597	-0.243962
16	6	0	-5.774798	0.461111	-0.210991
17	1	0	-4.418445	2.061340	0.287928
18	6	0	-5.898471	-0.899937	-0.496917
19	1	0	-4.859459	-2.777343	-0.731477
20	1	0	-6.653523	1.099129	-0.201851
21	1	0	-6.875224	-1.324157	-0.710900
22	6	0	-2.073165	0.801183	0.355099
23	1	0	-2.096222	1.878604	0.565708
24	8	0	1.017490	2.207021	-1.269439
25	1	0	0.215149	2.133258	-1.807621
26	6	0	3.515104	-0.068698	-0.720861
27	1	0	3.609653	0.286656	-1.755466
28	6	0	4.915372	-0.149753	-0.106958

29	1	0	5.432522	0.811605	-0.203028
30	1	0	4.829593	-0.384121	0.965125
31	8	0	2.967283	-1.387843	-0.798253
32	1	0	2.472503	-1.553954	0.030142
33	8	0	5.692554	-1.128263	-0.786544
34	1	0	5.084509	-1.878796	-0.890302
35	8	0	1.054621	-1.359172	1.312196
36	1	0	0.190943	-1.468473	0.866548

Structure 82c (M06-2X/6-311G(d,p), Gas Phase)

Energy (Hartrees): = -936.3463456
No imaginary frequencies

Standard orientation:

Center Number	Atomic Number	Atomic Type	Coordinates (Angstroms) X	Y	Z
1	6	0	-2.528022	-0.848951	0.181943
2	8	0	-2.447766	-0.167647	1.444344
3	6	0	-1.168794	0.364159	1.626026
4	6	0	-0.244815	-0.587269	0.863098
5	6	0	-1.102481	-0.876536	-0.379300
6	1	0	-2.862089	-1.875055	0.364652
7	1	0	-0.985812	0.418630	2.700606
8	1	0	-0.131534	-1.526070	1.420298
9	1	0	-0.977991	-0.061207	-1.098024
10	7	0	1.008720	0.027629	0.512513
11	6	0	3.372749	-0.202295	0.092430
12	6	0	3.565238	1.129953	-0.283634
13	6	0	4.456614	-1.079958	0.097064
14	6	0	4.826864	1.572212	-0.647294
15	1	0	2.715085	1.800727	-0.284445
16	6	0	5.721671	-0.635682	-0.269475
17	1	0	4.306365	-2.114222	0.389162
18	6	0	5.906827	0.690347	-0.641234
19	1	0	4.974302	2.605363	-0.937604
20	1	0	6.559835	-1.321571	-0.264739
21	1	0	6.891567	1.039653	-0.927564
22	6	0	2.044668	-0.703366	0.484751
23	1	0	1.996073	-1.766050	0.758599
24	8	0	-0.822875	-2.126186	-0.965879
25	1	0	-0.204946	-1.994372	-1.687351
26	6	0	-3.514082	-0.129938	-0.726305
27	1	0	-3.558845	-0.667355	-1.680145
28	6	0	-4.914275	-0.065024	-0.131537
29	1	0	-5.321401	-1.070883	-0.001088
30	1	0	-4.855321	0.417222	0.852715
31	8	0	-3.077028	1.184355	-1.032035
32	1	0	-2.691567	1.570398	-0.230214
33	8	0	-5.777097	0.631533	-1.001455
34	1	0	-5.291014	1.419854	-1.267444
35	8	0	-1.116279	1.653470	1.057172
36	1	0	-0.228347	1.761434	0.688636

Structure 82c (M06-2X/6-311G(d,p), DMSO)

Energy (Hartrees): = -936.3723751
No imaginary frequencies
Standard orientation:

Center Number	Atomic Number	Atomic Type	Coordinates (Angstroms) X	Y	Z
1	6	0	2.543318	0.859515	0.109869
2	8	0	2.435215	0.281110	1.423787
3	6	0	1.142895	-0.212946	1.625458
4	6	0	0.242363	0.688316	0.779784
5	6	0	1.123397	0.886971	-0.465457
6	1	0	2.913259	1.883561	0.215380
7	1	0	0.937746	-0.183056	2.696394
8	1	0	0.115391	1.657357	1.277203
9	1	0	0.977748	0.040314	-1.144752
10	7	0	-1.004770	0.042180	0.454529
11	6	0	-3.378168	0.204451	0.073096
12	6	0	-3.543103	-1.135224	-0.294694
13	6	0	-4.487426	1.051310	0.109664
14	6	0	-4.803384	-1.616150	-0.616916
15	1	0	-2.678613	-1.788277	-0.324914
16	6	0	-5.750699	0.567345	-0.214548
17	1	0	-4.355249	2.090403	0.393596
18	6	0	-5.909302	-0.766038	-0.576940
19	1	0	-4.929088	-2.654493	-0.900982
20	1	0	-6.608324	1.229181	-0.184871

21	1	0	-6.892565	-1.145339	-0.830567
22	6	0	-2.056602	0.752336	0.427930
23	1	0	-2.032632	1.820233	0.675756
24	8	0	0.902175	2.107301	-1.132356
25	1	0	0.097617	2.016609	-1.655013
26	6	0	3.520064	0.046433	-0.725994
27	1	0	3.561818	0.487439	-1.727974
28	6	0	4.919441	0.043964	-0.129405
29	1	0	5.336407	1.053795	-0.136060
30	1	0	4.864240	-0.305843	0.909430
31	8	0	3.085414	-1.297299	-0.889077
32	1	0	2.618469	-1.563891	-0.080987
33	8	0	5.776495	-0.780487	-0.897533
34	1	0	5.275372	-1.591410	-1.044881
35	8	0	1.081890	-1.548283	1.159004
36	1	0	0.205517	-1.662291	0.759863

Structure 82c (M06-2X/def2-TZVP, Gas Phase)

Energy (Hartrees): = -936.4623483
No imaginary frequencies

Standard orientation:

Center Number	Atomic Number	Atomic Type	Coordinates (Angstroms) X	Y	Z
1	6	0	2.530754	0.822275	0.189288
2	8	0	2.427524	0.127661	1.438318
3	6	0	1.139008	-0.382595	1.612069
4	6	0	0.237201	0.577002	0.834968
5	6	0	1.115584	0.860877	-0.391529
6	1	0	2.863286	1.845395	0.388112
7	1	0	0.945046	-0.419611	2.685108
8	1	0	0.125183	1.513070	1.395394
9	1	0	0.991153	0.050811	-1.114729
10	7	0	-1.016584	-0.020422	0.469333
11	6	0	-3.386401	0.203940	0.094610
12	6	0	-3.577539	-1.108475	-0.337803
13	6	0	-4.472749	1.072161	0.152790
14	6	0	-4.839161	-1.539786	-0.704280
15	1	0	-2.726358	-1.775039	-0.380813
16	6	0	-5.738265	0.639289	-0.216401
17	1	0	-4.323515	2.091759	0.489559
18	6	0	-5.921487	-0.666776	-0.644811
19	1	0	-4.984992	-2.558458	-1.039301
20	1	0	-6.578411	1.319543	-0.169605
21	1	0	-6.907046	-1.007993	-0.934026
22	6	0	-2.058826	0.696607	0.490918
23	1	0	-2.016199	1.744205	0.814930
24	8	0	0.859909	2.112287	-0.983163
25	1	0	0.228503	2.000927	-1.698447
26	6	0	3.533052	0.120438	-0.711360
27	1	0	3.565003	0.652636	-1.667967
28	6	0	4.931434	0.102539	-0.114579
29	1	0	5.304573	1.121216	0.008814
30	1	0	4.891325	-0.374628	0.871820
31	8	0	3.129722	-1.204481	-1.005589
32	1	0	2.729960	-1.591281	-0.210101
33	8	0	5.822779	-0.569929	-0.973319
34	1	0	5.376951	-1.381919	-1.243015
35	8	0	1.069403	-1.678498	1.067321
36	1	0	0.201305	-1.768967	0.647100

Structure 82c (M06-2X/def2-TZVP, DMSO)

Energy (Hartrees): = -936.4883004
No imaginary frequencies

Standard orientation:

Center Number	Atomic Number	Atomic Type	Coordinates (Angstroms) X	Y	Z
1	6	0	2.538478	0.819847	0.157597
2	8	0	2.418772	0.169551	1.432274
3	6	0	1.119989	-0.315035	1.613260
4	6	0	0.234463	0.625598	0.798524
5	6	0	1.125614	0.869660	-0.428298
6	1	0	2.898923	1.839042	0.322597
7	1	0	0.912527	-0.314116	2.683834
8	1	0	0.114475	1.571848	1.337715
9	1	0	0.983088	0.051896	-1.141035
10	7	0	-1.015858	0.012267	0.439839
11	6	0	-3.388808	0.205001	0.081575

12	6	0	-3.569465	-1.110504	-0.349855
13	6	0	-4.485836	1.060104	0.157486
14	6	0	-4.832170	-1.558451	-0.697160
15	1	0	-2.715054	-1.773120	-0.409976
16	6	0	-5.751984	0.609817	-0.191953
17	1	0	-4.341569	2.081199	0.492442
18	6	0	-5.925606	-0.699322	-0.618834
19	1	0	-4.969788	-2.579093	-1.031778
20	1	0	-6.600307	1.279895	-0.131713
21	1	0	-6.911631	-1.053248	-0.893218
22	6	0	-2.064904	0.721790	0.462907
23	1	0	-2.034743	1.770981	0.776439
24	8	0	0.908098	2.111653	-1.053878
25	1	0	0.134824	2.034963	-1.625997
26	6	0	3.533531	0.068885	-0.711013
27	1	0	3.544154	0.540467	-1.699386
28	6	0	4.936942	0.113974	-0.132955
29	1	0	5.297213	1.143751	-0.100911
30	1	0	4.921398	-0.281756	0.889337
31	8	0	3.149856	-1.283842	-0.903959
32	1	0	2.671693	-1.583478	-0.113438
33	8	0	5.832606	-0.624976	-0.941876
34	1	0	5.396649	-1.468595	-1.119176
35	8	0	1.041307	-1.634325	1.113600
36	1	0	0.182595	-1.719176	0.668417

Structure 82d ($^1T_2$) (B3LYP, Gas Phase)

Energy (Hartrees): = - 936.4729727
No imaginary frequencies

Standard orientation:

Center Number	Atomic Number	Atomic Type	Coordinates (Angstroms) X	Y	Z
1	6	0	-2.683010	-0.226678	-0.258540
2	8	0	-2.312628	-0.533188	1.092675
3	6	0	-1.527875	-1.726209	1.134141
4	6	0	-0.769516	-1.716670	-0.221854
5	6	0	-1.859786	-1.186908	-1.169147
6	1	0	-3.753044	-0.449082	-0.394738
7	1	0	-2.169521	-2.612957	1.199474
8	1	0	-0.450956	-2.724368	-0.517150
9	1	0	-1.431726	-0.657651	-2.029056
10	7	0	0.329407	-0.774830	-0.129258
11	6	0	2.719664	-0.357152	-0.161052
12	6	0	2.637337	1.045481	-0.101293
13	6	0	3.982050	-0.970600	-0.147377
14	6	0	3.796905	1.808364	-0.019010
15	1	0	1.666017	1.526380	-0.137602
16	6	0	5.142091	-0.203431	-0.060320
17	1	0	4.049757	-2.054326	-0.199713
18	6	0	5.050487	1.187315	0.005388
19	1	0	3.727097	2.891066	0.022272
20	1	0	6.113268	-0.688395	-0.045529
21	1	0	5.952448	1.788615	0.070246
22	6	0	1.526307	-1.208859	-0.243546
23	1	0	1.719586	-2.278194	-0.416009
24	8	0	-2.610778	-2.326205	-1.573375
25	1	0	-3.344870	-2.023556	-2.123675
26	6	0	-2.464104	1.262493	-0.540164
27	1	0	-2.926828	1.484909	-1.513431
28	6	0	-3.110232	2.175659	0.508538
29	1	0	-4.199648	2.053918	0.509345
30	1	0	-2.736729	1.895825	1.504163
31	8	0	-1.086063	1.617233	-0.657990
32	1	0	-0.542213	0.938831	-0.208206
33	8	0	-2.834028	3.531291	0.203844
34	1	0	-1.885439	3.543910	0.001012
35	8	0	-0.728532	-1.733748	2.262574
36	1	0	-0.158732	-0.951006	2.206995

Structure 82d ($^1T_2$) (B3LYP, DMSO)

Energy (Hartrees): = -936.4985746
No imaginary frequencies

Standard orientation:

Center Number	Atomic Number	Atomic Type	Coordinates (Angstroms) X	Y	Z

1	6	0	-2.685617	-0.205637	-0.283745
2	8	0	-2.366280	-0.474304	1.093952
3	6	0	-1.572508	-1.661649	1.199668
4	6	0	-0.799446	-1.721836	-0.142079
5	6	0	-1.872578	-1.228479	-1.127726
6	1	0	-3.757649	-0.390057	-0.443611
7	1	0	-2.215589	-2.542960	1.309274
8	1	0	-0.477376	-2.741374	-0.383507
9	1	0	-1.431220	-0.762178	-2.016286
10	7	0	0.305661	-0.781681	-0.068106
11	6	0	2.698682	-0.383136	-0.139496
12	6	0	2.645279	0.983733	0.190012
13	6	0	3.945371	-0.979207	-0.391015
14	6	0	3.816007	1.731730	0.260326
15	1	0	1.687954	1.451564	0.394321
16	6	0	5.117165	-0.226657	-0.320341
17	1	0	3.989369	-2.035603	-0.642348
18	6	0	5.053925	1.129591	0.004775
19	1	0	3.768404	2.785986	0.516600
20	1	0	6.075800	-0.697258	-0.517046
21	1	0	5.965125	1.717925	0.062023
22	6	0	1.497201	-1.223162	-0.225468
23	1	0	1.678004	-2.284379	-0.440880
24	8	0	-2.648278	-2.371834	-1.475568
25	1	0	-3.373196	-2.068800	-2.042743
26	6	0	-2.414335	1.262904	-0.623180
27	1	0	-2.866991	1.448825	-1.607895
28	6	0	-3.046494	2.232182	0.380336
29	1	0	-4.139327	2.152682	0.361124
30	1	0	-2.703250	1.978691	1.393825
31	8	0	-1.027014	1.590525	-0.738631
32	1	0	-0.492461	0.897484	-0.290956
33	8	0	-2.704852	3.572312	0.043128
34	1	0	-1.753384	3.525025	-0.147927
35	8	0	-0.784647	-1.608346	2.337332
36	1	0	-0.141186	-0.892886	2.198668

Structure 82d ($^1T_2$) (M06-2X/6-311G(d,p), Gas Phase)

Energy (Hartrees): = -936.3416159
No imaginary frequencies

Standard orientation:

Center Number	Atomic Number	Atomic Type	Coordinates (Angstroms) X	Y	Z
1	6	0	-2.634847	-0.218431	-0.233855
2	8	0	-2.206947	-0.526492	1.088831
3	6	0	-1.499034	-1.756067	1.084284
4	6	0	-0.756879	-1.732614	-0.269070
5	6	0	-1.835850	-1.150541	-1.183557
6	1	0	-3.703158	-0.455157	-0.333429
7	1	0	-2.189562	-2.603462	1.122292
8	1	0	-0.455412	-2.732256	-0.597289
9	1	0	-1.401376	-0.596501	-2.021356
10	7	0	0.345392	-0.804305	-0.154069
11	6	0	2.709836	-0.365939	-0.177912
12	6	0	2.584704	1.024656	-0.254621
13	6	0	3.972325	-0.939560	-0.026936
14	6	0	3.712730	1.824643	-0.162785
15	1	0	1.606107	1.464057	-0.410647
16	6	0	5.100739	-0.134557	0.072166
17	1	0	4.068013	-2.019489	0.020296
18	6	0	4.970488	1.247464	0.005861
19	1	0	3.615373	2.901490	-0.228950
20	1	0	6.078186	-0.584354	0.197167
21	1	0	5.849054	1.877714	0.075939
22	6	0	1.529497	-1.241780	-0.265076
23	1	0	1.726478	-2.308252	-0.437635
24	8	0	-2.608391	-2.252234	-1.621536
25	1	0	-3.328971	-1.924961	-2.165164
26	6	0	-2.437695	1.267561	-0.491559
27	1	0	-2.873482	1.506038	-1.469900
28	6	0	-3.120705	2.132881	0.560093
29	1	0	-4.202398	1.976423	0.541795
30	1	0	-2.743540	1.849662	1.549756
31	8	0	-1.067870	1.633592	-0.554351
32	1	0	-0.540083	0.960446	-0.094882
33	8	0	-2.884831	3.494848	0.282120
34	1	0	-1.937942	3.558528	0.115965
35	8	0	-0.689523	-1.838239	2.193039
36	1	0	-0.096392	-1.077898	2.167506

Structure 82d ($^1T_2$) (M06-2X/6-311G(d,p), DMSO)

Energy (Hartrees): = -936.369498
No imaginary frequencies

Standard orientation:

Center Number	Atomic Number	Atomic Type	Coordinates (Angstroms) X	Y	Z
1	6	0	-2.648493	-0.203072	-0.251764
2	8	0	-2.260217	-0.461383	1.099102
3	6	0	-1.530542	-1.679741	1.165702
4	6	0	-0.786052	-1.734901	-0.182702
5	6	0	-1.867971	-1.212936	-1.129859
6	1	0	-3.722310	-0.398904	-0.363862
7	1	0	-2.210105	-2.531164	1.261588
8	1	0	-0.474591	-2.749433	-0.446428
9	1	0	-1.442925	-0.738790	-2.019486
10	7	0	0.316828	-0.799481	-0.109509
11	6	0	2.689663	-0.383028	-0.151614
12	6	0	2.592474	0.986993	0.116580
13	6	0	3.946398	-0.961798	-0.338080
14	6	0	3.740175	1.761448	0.193965
15	1	0	1.619529	1.439372	0.270571
16	6	0	5.096571	-0.183344	-0.259045
17	1	0	4.017675	-2.024859	-0.544019
18	6	0	4.993719	1.177989	0.005856
19	1	0	3.662629	2.822079	0.402632
20	1	0	6.069339	-0.638702	-0.403818
21	1	0	5.888340	1.787145	0.066577
22	6	0	1.498260	-1.244307	-0.242157
23	1	0	1.687464	-2.306428	-0.436083
24	8	0	-2.662990	-2.335252	-1.468684
25	1	0	-3.402912	-2.023921	-2.001663
26	6	0	-2.402616	1.259026	-0.592427
27	1	0	-2.851217	1.442841	-1.576246
28	6	0	-3.051495	2.196518	0.415655
29	1	0	-4.137809	2.078058	0.405771
30	1	0	-2.679333	1.955928	1.418422
31	8	0	-1.025241	1.600244	-0.685487
32	1	0	-0.489664	0.894144	-0.279021
33	8	0	-2.762634	3.541817	0.077321
34	1	0	-1.813509	3.552092	-0.095463
35	8	0	-0.723324	-1.679568	2.282280
36	1	0	-0.083693	-0.963035	2.170165

Structure 82d ($^{1}T_{2}$) (M06-2X/ def2-TZVP, Gas Phase)

Energy (Hartrees): = -936.4579439
No imaginary frequencies

Standard orientation:

Center Number	Atomic Number	Atomic Type	Coordinates (Angstroms) X	Y	Z
1	6	0	-2.640585	-0.253196	-0.207118
2	8	0	-2.189819	-0.542911	1.109049
3	6	0	-1.435801	-1.741067	1.105838
4	6	0	-0.730979	-1.722149	-0.266657
5	6	0	-1.848785	-1.185141	-1.158327
6	1	0	-3.706177	-0.504806	-0.286573
7	1	0	-2.091427	-2.612830	1.177974
8	1	0	-0.417681	-2.721959	-0.578307
9	1	0	-1.454535	-0.639796	-2.019961
10	7	0	0.355985	-0.775507	-0.203005
11	6	0	2.723948	-0.332044	-0.180955
12	6	0	2.608500	1.058614	-0.187964
13	6	0	3.984173	-0.917050	-0.092011
14	6	0	3.742203	1.845295	-0.095186
15	1	0	1.630651	1.513745	-0.279353
16	6	0	5.119776	-0.126500	0.005591
17	1	0	4.072176	-1.997447	-0.093231
18	6	0	4.998382	1.254823	0.004809
19	1	0	3.650584	2.923463	-0.104335
20	1	0	6.095860	-0.587328	0.081198
21	1	0	5.882401	1.875345	0.076639
22	6	0	1.544222	-1.204464	-0.265435
23	1	0	1.748073	-2.275693	-0.385241
24	8	0	-2.607222	-2.309028	-1.560306
25	1	0	-3.369771	-2.011802	-2.065186
26	6	0	-2.480050	1.229692	-0.497911
27	1	0	-2.938982	1.424834	-1.475755
28	6	0	-3.184220	2.093576	0.536650
29	1	0	-4.257223	1.890496	0.536284

30	1	0	-2.784408	1.858798	1.528565
31	8	0	-1.127964	1.641962	-0.578333
32	1	0	-0.545123	0.940153	-0.238401
33	8	0	-3.015671	3.457757	0.224570
34	1	0	-2.073635	3.579131	0.055909
35	8	0	-0.598146	-1.776494	2.196091
36	1	0	-0.026793	-0.998362	2.154300

Structure 82d ($^1T_2$) (M06-2X/ def2-TZVP, DMSO)

Energy (Hartrees): = -936.4864596
No imaginary frequencies

Standard orientation:

Center Number	Atomic Number	Atomic Type	Coordinates (Angstroms) X	Y	Z
1	6	0	-2.616372	-0.230421	-0.281390
2	8	0	-2.294844	-0.478381	1.085476
3	6	0	-1.534290	-1.671576	1.199180
4	6	0	-0.749022	-1.745136	-0.121905
5	6	0	-1.795479	-1.241662	-1.113837
6	1	0	-3.681547	-0.430743	-0.447001
7	1	0	-2.194851	-2.536432	1.300186
8	1	0	-0.426476	-2.762418	-0.354885
9	1	0	-1.336939	-0.770647	-1.987109
10	7	0	0.347679	-0.807592	-0.040369
11	6	0	2.712180	-0.367090	-0.130014
12	6	0	2.632515	0.945064	0.339991
13	6	0	3.943845	-0.882945	-0.526910
14	6	0	3.772075	1.728250	0.402440
15	1	0	1.679501	1.340191	0.669577
16	6	0	5.084964	-0.094271	-0.469462
17	1	0	4.001912	-1.904829	-0.884199
18	6	0	4.999046	1.210825	-0.005197
19	1	0	3.709178	2.743972	0.772086
20	1	0	6.038588	-0.498134	-0.785149
21	1	0	5.888183	1.827219	0.043392
22	6	0	1.527516	-1.233246	-0.216217
23	1	0	1.718655	-2.285064	-0.453149
24	8	0	-2.568819	-2.365922	-1.490702
25	1	0	-3.292643	-2.063168	-2.052715
26	6	0	-2.369154	1.228848	-0.627857
27	1	0	-2.683309	1.363863	-1.670753
28	6	0	-3.198172	2.155314	0.243999
29	1	0	-4.262418	1.981993	0.074139
30	1	0	-2.972543	1.959074	1.297642
31	8	0	-1.013121	1.629631	-0.516443
32	1	0	-0.462684	0.883193	-0.208265
33	8	0	-2.932064	3.508200	-0.080631
34	1	0	-1.969741	3.590431	-0.094573
35	8	0	-0.759300	-1.627166	2.338235
36	1	0	-0.099342	-0.927986	2.218951

Structure 82e (B3LYP, Gas Phase)

Energy (Hartrees): = -936.468613

No imaginary frequencies

Standard orientation:

Center Number	Atomic Number	Atomic Type	Coordinates (Angstroms) X	Y	Z
1	6	0	2.616395	0.623328	0.713798
2	8	0	2.389558	-0.357374	1.736642
3	6	0	1.042793	-0.782705	1.729393
4	6	0	0.267405	0.349238	1.011293
5	6	0	1.302127	0.707325	-0.074132
6	1	0	2.781157	1.603086	1.186030
7	1	0	0.746375	-0.923362	2.776666
8	1	0	0.149889	1.213116	1.684044
9	1	0	1.255072	-0.064159	-0.850043
10	7	0	-0.978933	-0.121476	0.459150
11	6	0	-3.347294	0.216901	0.076182
12	6	0	-3.536897	-0.958399	-0.671162
13	6	0	-4.444727	1.055295	0.324305
14	6	0	-4.799220	-1.283305	-1.154129
15	1	0	-2.684233	-1.601113	-0.861610
16	6	0	-5.709855	0.727534	-0.160363
17	1	0	-4.302601	1.965398	0.901882
18	6	0	-5.888503	-0.442176	-0.899830
19	1	0	-4.939634	-2.192851	-1.730394

20	1	0	-6.553062	1.381889	0.038311
21	1	0	-6.872976	-0.700075	-1.278737
22	6	0	-2.030052	0.592121	0.606523
23	1	0	-1.996110	1.545601	1.156176
24	8	0	1.182983	2.002358	-0.648361
25	1	0	0.434657	1.990028	-1.260536
26	6	0	3.869215	0.304126	-0.106132
27	1	0	4.723203	0.260399	0.579587
28	6	0	3.850667	-1.008989	-0.892649
29	1	0	3.749207	-1.858562	-0.212762
30	1	0	2.989394	-1.029196	-1.581882
31	8	0	4.140936	1.392318	-1.001948
32	1	0	3.291901	1.748229	-1.308617
33	8	0	5.066755	-1.153121	-1.605187
34	1	0	5.220144	-0.287177	-2.014134
35	8	0	0.925369	-1.990154	1.015806
36	1	0	0.063001	-1.954901	0.567757

Structure 82e (B3LYP, DMSO)

Energy (Hartrees): = -936.494355
No imaginary frequencies

Standard orientation:

Center Number	Atomic Number	Atomic Type	Coordinates (Angstroms) X	Y	Z
1	6	0	-2.617385	-0.354951	0.852101
2	8	0	-2.414302	0.912944	1.493180
3	6	0	-1.033496	1.264705	1.484394
4	6	0	-0.275739	-0.030755	1.102590
5	6	0	-1.298225	-0.663949	0.138065
6	1	0	-2.769260	-1.130987	1.617420
7	1	0	-0.791906	1.627437	2.490921
8	1	0	-0.166635	-0.679790	1.982563
9	1	0	-1.231214	-0.140930	-0.822237
10	7	0	0.976376	0.275116	0.450774
11	6	0	3.327425	-0.201786	0.125709
12	6	0	3.536346	0.776548	-0.863045
13	6	0	4.405398	-1.002489	0.536106
14	6	0	4.798054	0.946645	-1.423641
15	1	0	2.703295	1.393961	-1.183135
16	6	0	5.669419	-0.830305	-0.027791
17	1	0	4.246997	-1.760000	1.299334
18	6	0	5.867617	0.144317	-1.007817
19	1	0	4.952404	1.703778	-2.186897
20	1	0	6.496780	-1.454488	0.296460
21	1	0	6.851029	0.280010	-1.448641
22	6	0	2.012395	-0.416164	0.746099
23	1	0	1.967073	-1.219274	1.493987
24	8	0	-1.191924	-2.069458	-0.054066
25	1	0	-0.451951	-2.231909	-0.659562
26	6	0	-3.869151	-0.343234	-0.029626
27	1	0	-4.739267	-0.143836	0.607170
28	6	0	-3.884371	0.685405	-1.161835
29	1	0	-3.860424	1.699829	-0.754651
30	1	0	-3.002712	0.560468	-1.809192
31	8	0	-4.075489	-1.655100	-0.577815
32	1	0	-3.200181	-2.031494	-0.772640
33	8	0	-5.082749	0.548157	-1.918364
34	1	0	-5.158256	-0.406676	-2.078198
35	8	0	-0.801088	2.281536	0.540826
36	1	0	0.014309	2.019540	0.073937

Structure 82e (M06-2X/6-311G(d,p), Gas Phase)

Energy (Hartrees): = -936.3364153
No imaginary frequencies
Standard orientation:

Center Number	Atomic Number	Atomic Type	Coordinates (Angstroms) X	Y	Z
1	6	0	-2.594628	-0.658261	0.717240
2	8	0	-2.438085	0.339891	1.724684
3	6	0	-1.137613	0.858424	1.694359
4	6	0	-0.288432	-0.268743	1.088192
5	6	0	-1.251005	-0.727917	-0.011976
6	1	0	-2.756297	-1.631185	1.197248
7	1	0	-0.867281	1.123542	2.719211
8	1	0	-0.170382	-1.086291	1.812274
9	1	0	-1.200479	-0.005634	-0.831110
10	7	0	0.959913	0.202415	0.548595
11	6	0	3.296878	-0.194801	0.102011

12	6	0	3.501080	1.009103	-0.578061
13	6	0	4.360103	-1.082354	0.266701
14	6	0	4.754814	1.316080	-1.081265
15	1	0	2.667221	1.689266	-0.699655
16	6	0	5.616997	-0.774097	-0.240246
17	1	0	4.200726	-2.017163	0.794336
18	6	0	5.814448	0.425246	-0.913595
19	1	0	4.912300	2.250983	-1.605241
20	1	0	6.438566	-1.467482	-0.109347
21	1	0	6.793395	0.669213	-1.308518
22	6	0	1.977507	-0.548178	0.651912
23	1	0	1.918717	-1.515767	1.169984
24	8	0	-1.039439	-2.040018	-0.490147
25	1	0	-0.258355	-2.034786	-1.051334
26	6	0	-3.796582	-0.363920	-0.167168
27	1	0	-4.695391	-0.365031	0.455801
28	6	0	-3.753325	0.970087	-0.898667
29	1	0	-3.703248	1.792749	-0.185374
30	1	0	-2.864178	1.024337	-1.541874
31	8	0	-3.962575	-1.419858	-1.110746
32	1	0	-3.096847	-1.784132	-1.327648
33	8	0	-4.928190	1.117338	-1.666629
34	1	0	-5.038412	0.283875	-2.136581
35	8	0	-1.105014	1.985350	0.860503
36	1	0	-0.214469	2.030955	0.489729

Structure 82e (M06-2X/6-311G(d,p), DMSO)

Energy (Hartrees): = -936.3646359
No imaginary frequencies

Standard orientation:

Center Number	Atomic Number	Atomic Type	Coordinates (Angstroms) X	Y	Z
1	6	0	2.604375	0.607856	0.751218
2	8	0	2.447906	-0.461275	1.685406
3	6	0	1.123421	-0.934841	1.669242
4	6	0	0.294145	0.228999	1.111277
5	6	0	1.259478	0.739459	0.035176
6	1	0	2.779971	1.541471	1.298868
7	1	0	0.864059	-1.210255	2.693860
8	1	0	0.170296	1.005708	1.875598
9	1	0	1.193729	0.075531	-0.832114
10	7	0	-0.952854	-0.221145	0.544758
11	6	0	-3.286120	0.193871	0.101256
12	6	0	-3.504564	-1.000363	-0.593752
13	6	0	-4.338057	1.096023	0.270735
14	6	0	-4.761144	-1.283419	-1.107882
15	1	0	-2.685767	-1.698574	-0.722746
16	6	0	-5.597234	0.811226	-0.247277
17	1	0	-4.164138	2.021889	0.809631
18	6	0	-5.809382	-0.378335	-0.935851
19	1	0	-4.929270	-2.210518	-1.643593
20	1	0	-6.409259	1.516194	-0.113268
21	1	0	-6.789942	-0.603340	-1.339657
22	6	0	-1.967935	0.531912	0.666872
23	1	0	-1.912587	1.482665	1.210571
24	8	0	1.072908	2.082699	-0.359730
25	1	0	0.278343	2.128240	-0.905757
26	6	0	3.800249	0.362881	-0.157326
27	1	0	4.705131	0.328629	0.456598
28	6	0	3.739632	-0.926791	-0.961407
29	1	0	3.705161	-1.787097	-0.292498
30	1	0	2.842621	-0.945481	-1.592924
31	8	0	3.965059	1.471855	-1.039973
32	1	0	3.089477	1.785399	-1.300095
33	8	0	4.905269	-1.040063	-1.761008
34	1	0	4.996361	-0.187033	-2.201605
35	8	0	1.035880	-2.052591	0.824429
36	1	0	0.178424	-1.991285	0.379145

Structure 82·5H_2O (M06-2X/6-311G(d,p), Gas Phase)

Energy (Hartrees): = -1318.5257258
No imaginary frequencies

Standard orientation:

Center Number	Atomic Number	Atomic Type	Coordinates (Angstroms) X	Y	Z
1	6	0	-1.595197	1.364410	-0.448584
2	8	0	-1.807044	0.499897	-1.577963

3	6	0	-0.679486	-0.289644	-1.839392
4	6	0	0.488690	0.457933	-1.191763
5	6	0	-0.191111	1.046064	0.054561
6	1	0	-1.630373	2.402939	-0.798563
7	1	0	-0.599828	-0.390911	-2.923696
8	1	0	0.805950	1.287010	-1.838576
9	1	0	-0.255573	0.272339	0.824389
10	7	0	1.571464	-0.413319	-0.810757
11	6	0	3.915416	-0.632050	-0.290297
12	6	0	3.843744	-1.967803	0.114515
13	6	0	5.122367	0.060867	-0.177062
14	6	0	4.970178	-2.599711	0.617282
15	1	0	2.899089	-2.490377	0.026989
16	6	0	6.249646	-0.573398	0.333355
17	1	0	5.168849	1.099255	-0.492006
18	6	0	6.173445	-1.903864	0.728648
19	1	0	4.915075	-3.636230	0.927471
20	1	0	7.183901	-0.032348	0.420590
21	1	0	7.050481	-2.401076	1.125268
22	6	0	2.735639	0.090980	-0.796715
23	1	0	2.917781	1.119126	-1.129982
24	8	0	0.454483	2.193640	0.556394
25	1	0	1.096421	1.918242	1.236688
26	6	0	-2.679311	1.132862	0.604384
27	6	0	-4.059963	1.236226	-0.033674
28	1	0	-4.228643	2.251706	-0.401888
29	1	0	-4.087363	0.551389	-0.889615
30	8	0	-2.524870	-0.107563	1.248667
31	1	0	-2.767145	-0.815813	0.619958
32	8	0	-5.102304	0.929230	0.874198
33	1	0	-4.816391	0.199127	1.458118
34	8	0	-0.840021	-1.564154	-1.266956
35	1	0	-0.012238	-1.789895	-0.819956
36	8	0	-3.434209	-2.154382	-0.315178
37	8	0	2.819365	1.715536	1.862419
38	1	0	3.301796	0.884424	1.846250
39	1	0	3.208371	2.263325	1.163572
40	1	0	-4.255588	-1.893886	-0.758030
41	8	0	-5.037029	-1.631699	2.009848
42	1	0	-5.598723	-1.949292	1.292788
43	1	0	-4.173065	-1.996506	1.790734
44	8	0	-6.038285	-1.177434	-0.602664
45	1	0	-5.967058	-0.325884	-0.127153
46	1	0	-6.775046	-1.100210	-1.210632
47	8	0	2.805118	3.275856	-0.447949
48	1	0	1.857387	3.118293	-0.292296
49	1	0	2.907063	4.220569	-0.579225
50	1	0	-2.706577	-2.141537	-0.950690
51	1	0	-2.564775	1.900817	1.376707

Structure 82·5H_2O (M06-2X/6-311G(d,p), DMSO)

Energy (Hartrees): = -1318.5600509
No imaginary frequencies

Standard orientation:

Center Number	Atomic Number	Atomic Type	Coordinates (Angstroms) X	Y	Z
1	6	0	-1.608156	1.338926	-0.381252
2	8	0	-1.801537	0.417534	-1.470176
3	6	0	-0.640770	-0.328566	-1.724430
4	6	0	0.500898	0.460929	-1.079466
5	6	0	-0.205975	1.058066	0.147215
6	1	0	-1.652177	2.360035	-0.777044
7	1	0	-0.547958	-0.437342	-2.806025
8	1	0	0.808940	1.276123	-1.745908
9	1	0	-0.256503	0.298274	0.933005
10	7	0	1.590717	-0.394580	-0.677978
11	6	0	3.948862	-0.650923	-0.260802
12	6	0	3.867289	-1.980569	0.166845
13	6	0	5.173812	0.018403	-0.209887
14	6	0	5.000958	-2.628311	0.633623
15	1	0	2.913844	-2.494367	0.124713
16	6	0	6.308370	-0.631778	0.265971
17	1	0	5.233419	1.049701	-0.545176
18	6	0	6.222122	-1.954716	0.686118
19	1	0	4.938202	-3.659767	0.960577
20	1	0	7.255694	-0.107225	0.306535
21	1	0	7.104984	-2.464375	1.054675
22	6	0	2.764631	0.085249	-0.738517
23	1	0	2.951551	1.091369	-1.130526
24	8	0	0.391259	2.236766	0.636866
25	1	0	1.144246	1.993745	1.207620
26	6	0	-2.709882	1.145514	0.656882

27	6	0	-4.076478	1.264970	-0.007917
28	1	0	-4.243663	2.292297	-0.339010
29	1	0	-4.089903	0.614292	-0.889933
30	8	0	-2.596117	-0.095043	1.322515
31	1	0	-2.765195	-0.801909	0.673473
32	8	0	-5.136808	0.915074	0.871126
33	1	0	-4.862807	0.130117	1.379897
34	8	0	-0.766659	-1.607745	-1.142211
35	1	0	0.060231	-1.797031	-0.672903
36	8	0	-3.395347	-2.186352	-0.330182
37	8	0	2.912091	1.990138	1.727865
38	1	0	3.450142	1.191151	1.713951
39	1	0	3.166211	2.494347	0.936300
40	1	0	-4.150413	-1.845931	-0.834406
41	8	0	-5.152438	-1.785707	1.845803
42	1	0	-5.771589	-1.941660	1.120894
43	1	0	-4.321050	-2.106340	1.472460
44	8	0	-5.979538	-1.169581	-0.769225
45	1	0	-5.977286	-0.305995	-0.320407
46	1	0	-6.601438	-1.100980	-1.500686
47	8	0	2.603290	3.352563	-0.655356
48	1	0	1.665250	3.219756	-0.444983
49	1	0	2.698268	4.275108	-0.912560
50	1	0	-2.590167	-2.105109	-0.863359
51	1	0	-2.595508	1.919425	1.422188

Structure 82·5H_2O (M06-2X/6-311G(d,p), Water)

Energy (Hartrees): = -1318.5828702
No imaginary frequencies

Standard orientation:

Center Number	Atomic Number	Atomic Type	Coordinates (Angstroms) X	Y	Z
1	6	0	-1.551730	1.225054	-0.335261
2	8	0	-1.723364	0.313460	-1.437642
3	6	0	-0.624372	-0.572744	-1.533532
4	6	0	0.532398	0.219160	-0.917883
5	6	0	-0.162343	0.943567	0.237977
6	1	0	-1.580076	2.246501	-0.727129
7	1	0	-0.468461	-0.803771	-2.585725
8	1	0	0.824587	0.985016	-1.648894
9	1	0	-0.235115	0.277008	1.103045
10	7	0	1.654163	-0.575504	-0.476201
11	6	0	4.060312	-0.676063	-0.221543
12	6	0	4.116571	-1.971261	0.303038
13	6	0	5.231772	0.079848	-0.318208
14	6	0	5.329674	-2.497500	0.722812
15	1	0	3.209694	-2.559605	0.369814
16	6	0	6.446249	-0.447587	0.108306
17	1	0	5.181688	1.085106	-0.725742
18	6	0	6.495459	-1.736564	0.628399
19	1	0	5.371703	-3.503410	1.123407
20	1	0	7.350315	0.145075	0.035888
21	1	0	7.440080	-2.151336	0.960363
22	6	0	2.800102	-0.056496	-0.669634
23	1	0	2.914047	0.906452	-1.178556
24	8	0	0.460137	2.155546	0.612037
25	1	0	1.262779	1.957509	1.130524
26	6	0	-2.674891	1.027649	0.676050
27	6	0	-4.024415	1.310822	0.035451
28	1	0	-4.070051	2.354814	-0.279314
29	1	0	-4.133966	0.675214	-0.851065
30	8	0	-2.649694	-0.281346	1.226017
31	1	0	-3.038905	-0.895524	0.571326
32	8	0	-5.102750	1.089116	0.935360
33	1	0	-5.007192	0.196186	1.314265
34	8	0	-0.902489	-1.803243	-0.914219
35	1	0	-1.051282	-1.660527	0.031261
36	8	0	-3.810776	-2.066652	-0.539434
37	8	0	3.063949	2.137922	1.601245
38	1	0	3.573928	1.320436	1.613151
39	1	0	3.217082	2.517997	0.718560
40	1	0	-4.603476	-1.602557	-0.864449
41	8	0	-5.460299	-1.622051	1.774173
42	1	0	-6.082715	-1.641600	1.033294
43	1	0	-4.675374	-2.043990	1.401998
44	8	0	-6.304363	-0.820396	-0.819715
45	1	0	-6.198459	0.042666	-0.390443
46	1	0	-6.803141	-0.652587	-1.625998
47	8	0	2.613965	3.245073	-0.961017
48	1	0	1.730240	2.974132	-0.663141
49	1	0	2.588065	4.208149	-0.954381
50	1	0	-3.111699	-1.938243	-1.193782

51	1	0	-2.506686	1.722655	1.504873

Structure 82·5H_2O (M06-2X/def2-TZVP, Gas Phase)

Energy (Hartrees): = -1318.680665
No imaginary frequencies

Standard orientation:

Center Number	Atomic Number	Atomic Type	Coordinates (Angstroms) X	Y	Z
1	6	0	-1.616263	1.267172	-0.542261
2	8	0	-1.776882	0.359476	-1.639576
3	6	0	-0.614946	-0.388487	-1.871179
4	6	0	0.506443	0.397768	-1.190137
5	6	0	-0.232458	0.978424	0.022937
6	1	0	-1.641092	2.291869	-0.930940
7	1	0	-0.499143	-0.480839	-2.952525
8	1	0	0.817881	1.229008	-1.835678
9	1	0	-0.311540	0.208935	0.794425
10	7	0	1.604077	-0.430168	-0.771219
11	6	0	3.953017	-0.601863	-0.262934
12	6	0	3.897187	-1.905707	0.230467
13	6	0	5.157707	0.096977	-0.226188
14	6	0	5.035658	-2.499048	0.745658
15	1	0	2.954812	-2.436799	0.202182
16	6	0	6.297601	-0.497696	0.296732
17	1	0	5.193938	1.111773	-0.608174
18	6	0	6.236738	-1.796370	0.780170
19	1	0	4.991512	-3.511681	1.124851
20	1	0	7.230177	0.050304	0.324366
21	1	0	7.124252	-2.263661	1.186862
22	6	0	2.762126	0.080815	-0.791940
23	1	0	2.930537	1.091478	-1.180308
24	8	0	0.367234	2.140386	0.543065
25	1	0	1.021261	1.885907	1.220792
26	6	0	-2.746183	1.084481	0.467366
27	6	0	-4.090541	1.171680	-0.241070
28	1	0	-4.180112	2.140223	-0.738596
29	1	0	-4.122060	0.394118	-1.012622
30	8	0	-2.620609	-0.123673	1.179503
31	1	0	-2.816278	-0.878955	0.581500
32	8	0	-5.195476	1.043639	0.632316
33	1	0	-4.988963	0.422517	1.357634
34	8	0	-0.749397	-1.671758	-1.316961
35	1	0	0.058312	-1.868010	-0.819656
36	8	0	-3.367982	-2.288153	-0.283176
37	8	0	2.771037	1.769675	1.895673
38	1	0	3.269708	0.946258	1.890707
39	1	0	3.134620	2.314814	1.179543
40	1	0	-4.214866	-2.094666	-0.712569
41	8	0	-5.025851	-1.191456	2.348271
42	1	0	-5.244522	-1.787647	1.620256
43	1	0	-4.060847	-1.171566	2.377144
44	8	0	-6.006338	-1.365497	-0.471505
45	1	0	-6.003945	-0.439251	-0.161118
46	1	0	-6.883732	-1.553676	-0.810766
47	8	0	2.676525	3.332394	-0.459934
48	1	0	1.735827	3.131831	-0.303977
49	1	0	2.744970	4.281937	-0.586041
50	1	0	-2.658973	-2.261185	-0.940946
51	1	0	-2.664885	1.888992	1.205028

Structure 82·5H_2O (M06-2X/def2-TZVP, DMSO)

Energy (Hartrees): = -1318.7146508
No imaginary frequencies

Standard orientation:

Center Number	Atomic Number	Atomic Type	Coordinates (Angstroms) X	Y	Z
1	6	0	-1.633214	1.172438	-0.604782
2	8	0	-1.778742	0.159367	-1.608905
3	6	0	-0.568552	-0.516266	-1.850998
4	6	0	0.512048	0.328774	-1.173228
5	6	0	-0.264163	0.923469	0.008365
6	1	0	-1.639654	2.157084	-1.086457
7	1	0	-0.445989	-0.599730	-2.931358
8	1	0	0.810324	1.144419	-1.842111
9	1	0	-0.344151	0.171510	0.798402
10	7	0	1.622565	-0.461621	-0.714396
11	6	0	3.974090	-0.597720	-0.210474

12	6	0	3.947935	-1.918133	0.242549
13	6	0	5.160197	0.131187	-0.143127
14	6	0	5.096997	-2.496929	0.752362
15	1	0	3.024820	-2.481659	0.186983
16	6	0	6.310370	-0.449306	0.375558
17	1	0	5.177224	1.155847	-0.499348
18	6	0	6.278970	-1.763013	0.821846
19	1	0	5.076895	-3.522580	1.098902
20	1	0	7.228658	0.121854	0.427195
21	1	0	7.175126	-2.219277	1.223763
22	6	0	2.774122	0.066185	-0.742926
23	1	0	2.929796	1.068934	-1.156127
24	8	0	0.294343	2.111098	0.513007
25	1	0	1.019926	1.891187	1.126867
26	6	0	-2.797963	1.100146	0.374255
27	6	0	-4.112119	1.158249	-0.387713
28	1	0	-4.161400	2.084829	-0.962779
29	1	0	-4.142893	0.321216	-1.094138
30	8	0	-2.738851	-0.053404	1.191954
31	1	0	-2.822717	-0.857549	0.632201
32	8	0	-5.250627	1.123636	0.461458
33	1	0	-5.061196	0.574995	1.241490
34	8	0	-0.633885	-1.811487	-1.306556
35	1	0	0.148024	-1.936351	-0.744980
36	8	0	-3.205789	-2.368384	-0.165225
37	8	0	2.771252	2.036036	1.808870
38	1	0	3.380943	1.289214	1.842909
39	1	0	3.030698	2.568235	1.036233
40	1	0	-4.052544	-2.164272	-0.590443
41	8	0	-5.122584	-1.015493	2.443605
42	1	0	-5.386991	-1.552490	1.682158
43	1	0	-4.169468	-0.884913	2.336176
44	8	0	-5.906989	-1.503841	-0.324398
45	1	0	-6.005012	-0.538358	-0.251931
46	1	0	-6.701069	-1.836674	-0.758295
47	8	0	2.520826	3.462703	-0.575961
48	1	0	1.587599	3.216086	-0.465055
49	1	0	2.538794	4.409542	-0.755267
50	1	0	-2.490958	-2.266266	-0.813215
51	1	0	-2.726682	1.960185	1.046084

Structure 82·5H_2O (M06-2X/def2-TZVP, Water)

Energy (Hartrees): = -1318.7347904
No imaginary frequencies

Standard orientation:

Center Number	Atomic Number	Atomic Type	Coordinates (Angstroms) X	Y	Z
1	6	0	-1.560583	1.004852	-0.451783
2	8	0	-1.715715	-0.083785	-1.375490
3	6	0	-0.528283	-0.854055	-1.469002
4	6	0	0.567236	0.075816	-0.952837
5	6	0	-0.177190	0.824449	0.155334
6	1	0	-1.580312	1.946026	-1.011235
7	1	0	-0.415624	-1.135830	-2.514595
8	1	0	0.810436	0.798597	-1.740159
9	1	0	-0.234380	0.195169	1.048133
10	7	0	1.725827	-0.621339	-0.456767
11	6	0	4.129546	-0.628575	-0.198507
12	6	0	4.240492	-1.913122	0.336239
13	6	0	5.260877	0.182125	-0.279694
14	6	0	5.466659	-2.371417	0.787567
15	1	0	3.367295	-2.550504	0.385009
16	6	0	6.488060	-0.275942	0.180658
17	1	0	5.169079	1.177322	-0.701590
18	6	0	6.591022	-1.553449	0.714013
19	1	0	5.551999	-3.370287	1.196180
20	1	0	7.361121	0.361187	0.121720
21	1	0	7.546774	-1.916568	1.070274
22	6	0	2.853138	-0.079764	-0.679965
23	1	0	2.930665	0.850893	-1.250335
24	8	0	0.382846	2.077907	0.474030
25	1	0	1.173653	1.950941	1.031741
26	6	0	-2.693289	1.005489	0.563614
27	6	0	-4.016667	1.324425	-0.111969
28	1	0	-3.980342	2.337963	-0.512629
29	1	0	-4.180186	0.634086	-0.946610
30	8	0	-2.763154	-0.217934	1.274634
31	1	0	-3.077645	-0.912888	0.666844
32	8	0	-5.105040	1.267656	0.796857
33	1	0	-5.059974	0.426067	1.286509
34	8	0	-0.641747	-2.035779	-0.717748
35	1	0	-0.054752	-1.984544	0.049744

36	8	0	-3.882122	-2.014459	-0.697116
37	8	0	2.968608	2.259026	1.592197
38	1	0	3.518459	1.472959	1.696832
39	1	0	3.141785	2.580036	0.689807
40	1	0	-4.755353	-1.597293	-0.810650
41	8	0	-5.674897	-1.331128	1.971081
42	1	0	-6.109489	-1.441693	1.109691
43	1	0	-4.853199	-1.831731	1.897879
44	8	0	-6.471795	-0.797930	-0.691122
45	1	0	-6.328690	0.124464	-0.425080
46	1	0	-7.143898	-0.782822	-1.382139
47	8	0	2.534903	3.270480	-1.067084
48	1	0	1.669836	2.907255	-0.811857
49	1	0	2.419307	4.227962	-1.050643
50	1	0	-3.273707	-1.494634	-1.244193
51	1	0	-2.474839	1.779807	1.304805

Structure 97a (B3LYP, Gas Phase)

Energy (Hartrees): = -936.4575812
No imaginary frequencies

Standard orientation:

Center Number	Atomic Number	Atomic Type	Coordinates (Angstroms) X	Y	Z
1	6	0	-0.586789	1.750324	-0.972210
2	6	0	-0.476526	0.384958	-0.267158
3	6	0	-1.408818	-0.684988	-0.901572
4	6	0	-2.788164	-0.825212	-0.233908
5	6	0	-3.715314	0.388384	-0.278010
6	1	0	-1.222540	1.773779	-1.880891
7	1	0	-1.552583	-0.454047	-1.971350
8	1	0	-3.310328	-1.617027	-0.791281
9	1	0	-4.092799	0.461080	-1.309640
10	1	0	-0.769251	0.544546	0.776541
11	8	0	-2.988559	1.573934	0.062352
12	6	0	-4.893283	0.212029	0.704408
13	1	0	-4.515667	0.462661	1.703994
14	1	0	-5.689717	0.923138	0.454146
15	8	0	-5.466233	-1.081082	0.661584
16	1	0	-4.814893	-1.657506	1.090192
17	8	0	-2.614439	-1.243353	1.120310
18	1	0	-1.934105	-1.935803	1.064912
19	8	0	-0.828555	-1.976901	-0.756022
20	1	0	0.134595	-1.807659	-0.749254
21	8	0	0.020568	2.729913	-0.607111
22	7	0	0.902012	-0.106044	-0.354273
23	6	0	3.233699	0.119475	0.240621
24	6	0	3.687174	-0.937255	-0.567409
25	6	0	4.157176	0.819320	1.031826
26	6	0	5.032986	-1.285487	-0.574282
27	1	0	2.972088	-1.467593	-1.187032
28	6	0	5.506461	0.469305	1.023814
29	1	0	3.811158	1.641455	1.653065
30	6	0	5.945939	-0.584353	0.221652
31	1	0	5.376905	-2.102067	-1.202239
32	1	0	6.212490	1.017596	1.640090
33	1	0	6.996809	-0.858419	0.211977
34	6	0	1.819343	0.517358	0.284320
35	1	0	1.592814	1.399460	0.893684
36	1	0	-3.574627	2.335474	-0.038462

Structure 97a (B3LYP, DMSO)

Energy (Hartrees): = -936.4809734
No imaginary frequencies

Standard orientation:

Center Number	Atomic Number	Atomic Type	Coordinates (Angstroms) X	Y	Z
1	6	0	-0.618663	1.752181	-0.930578
2	6	0	-0.461136	0.382365	-0.244394
3	6	0	-1.386432	-0.696367	-0.861264
4	6	0	-2.767195	-0.833708	-0.203032
5	6	0	-3.698473	0.371475	-0.302314
6	1	0	-1.191988	1.758463	-1.877934
7	1	0	-1.525451	-0.481394	-1.932784
8	1	0	-3.273721	-1.644422	-0.747011
9	1	0	-4.017290	0.437830	-1.351539
10	1	0	-0.697425	0.521889	0.816498

11	8	0	-3.006349	1.568931	0.076323
12	6	0	-4.927838	0.201099	0.613957
13	1	0	-4.619489	0.467616	1.632917
14	1	0	-5.710127	0.903674	0.306655
15	8	0	-5.496422	-1.101581	0.563886
16	1	0	-4.853500	-1.672554	1.013110
17	8	0	-2.617678	-1.212625	1.169163
18	1	0	-1.942779	-1.911998	1.153227
19	8	0	-0.796863	-1.989230	-0.695305
20	1	0	0.163349	-1.833732	-0.768709
21	8	0	-0.080327	2.755675	-0.514865
22	7	0	0.926713	-0.049382	-0.431783
23	6	0	3.237336	0.077048	0.271265
24	6	0	3.748496	-0.704533	-0.780745
25	6	0	4.116177	0.565333	1.251546
26	6	0	5.108290	-0.991438	-0.842529
27	1	0	3.071494	-1.075754	-1.543391
28	6	0	5.479226	0.276606	1.187247
29	1	0	3.724068	1.172335	2.063507
30	6	0	5.977067	-0.502154	0.140764
31	1	0	5.496835	-1.594277	-1.658214
32	1	0	6.150723	0.658965	1.950279
33	1	0	7.038363	-0.727228	0.087576
34	6	0	1.808078	0.403173	0.379715
35	1	0	1.536968	1.066448	1.210000
36	1	0	-3.545502	2.323972	-0.203144

Structure 97a (M06-2X/6-311G(d,p), Gas Phase)

Energy (Hartrees): = -936.3248633
No imaginary frequencies

Standard orientation:

Center Number	Atomic Number	Atomic Type	Coordinates (Angstroms) X	Y	Z
1	6	0	-0.577029	1.761734	-1.012060
2	6	0	-0.481687	0.392851	-0.314235
3	6	0	-1.429753	-0.643502	-0.959234
4	6	0	-2.776683	-0.796563	-0.248449
5	6	0	-3.679177	0.425293	-0.208768
6	1	0	-1.386466	1.871840	-1.756303
7	1	0	-1.608717	-0.374852	-2.011420
8	1	0	-3.326126	-1.565417	-0.806450
9	1	0	-4.075354	0.572516	-1.222577
10	1	0	-0.774781	0.554938	0.728930
11	8	0	-2.933217	1.565941	0.197314
12	6	0	-4.825700	0.193445	0.785112
13	1	0	-4.411213	0.351137	1.785812
14	1	0	-5.614533	0.931356	0.614520
15	8	0	-5.410264	-1.080009	0.654671
16	1	0	-4.785080	-1.696803	1.050925
17	8	0	-2.560217	-1.246965	1.079005
18	1	0	-1.894586	-1.942717	1.003589
19	8	0	-0.865208	-1.939170	-0.881313
20	1	0	0.092774	-1.813996	-0.943884
21	8	0	0.187864	2.652217	-0.785716
22	7	0	0.886102	-0.086001	-0.415535
23	6	0	3.180834	0.095773	0.283266
24	6	0	3.674723	-0.786128	-0.681980
25	6	0	4.052391	0.646353	1.222363
26	6	0	5.020619	-1.115016	-0.697348
27	1	0	2.988768	-1.194556	-1.414105
28	6	0	5.402264	0.314778	1.207438
29	1	0	3.668490	1.338042	1.965057
30	6	0	5.886305	-0.566581	0.248293
31	1	0	5.402188	-1.797435	-1.447395
32	1	0	6.074683	0.744207	1.940134
33	1	0	6.938577	-0.824979	0.232458
34	6	0	1.753958	0.458702	0.332325
35	1	0	1.479582	1.229884	1.060614
36	1	0	-3.528971	2.315895	0.276812

Structure 97a (M06-2X/6-311G(d,p), DMSO)

Energy (Hartrees): = -936.3510177
No imaginary frequencies

Standard orientation:

Center Number	Atomic Number	Atomic Type	Coordinates (Angstroms) X	Y	Z
1	6	0	-0.630542	1.766646	-0.915390

2	6	0	-0.469012	0.383690	-0.266430
3	6	0	-1.395769	-0.660635	-0.914870
4	6	0	-2.759291	-0.807892	-0.240341
5	6	0	-3.650451	0.421043	-0.237867
6	1	0	-1.347148	1.838518	-1.752160
7	1	0	-1.546735	-0.405684	-1.972850
8	1	0	-3.294455	-1.575009	-0.814111
9	1	0	-3.969596	0.594491	-1.272720
10	1	0	-0.704972	0.496864	0.797082
11	8	0	-2.926067	1.546540	0.249399
12	6	0	-4.865735	0.191866	0.670256
13	1	0	-4.530272	0.339913	1.701468
14	1	0	-5.633178	0.936993	0.448141
15	8	0	-5.454300	-1.082241	0.497003
16	1	0	-4.844456	-1.710341	0.901329
17	8	0	-2.592477	-1.254268	1.098910
18	1	0	-1.939822	-1.964778	1.054240
19	8	0	-0.825893	-1.958600	-0.804072
20	1	0	0.123488	-1.852263	-0.951119
21	8	0	0.027269	2.710538	-0.574179
22	7	0	0.914238	-0.021221	-0.469182
23	6	0	3.196249	0.050884	0.294542
24	6	0	3.734373	-0.553599	-0.846510
25	6	0	4.036269	0.388853	1.357174
26	6	0	5.094100	-0.818415	-0.915567
27	1	0	3.078888	-0.805726	-1.672100
28	6	0	5.399620	0.120849	1.287055
29	1	0	3.615629	0.860917	2.239283
30	6	0	5.928791	-0.483182	0.151458
31	1	0	5.509756	-1.283740	-1.801814
32	1	0	6.045892	0.383843	2.116292
33	1	0	6.991017	-0.691393	0.093191
34	6	0	1.755871	0.344410	0.407662
35	1	0	1.449894	0.893855	1.305115
36	1	0	-3.478687	2.329237	0.141482

Structure 97a (M06-2X/def2-TZVP, Gas Phase)

Energy (Hartrees): = -936.4386358
No imaginary frequencies

Standard orientation:

Center Number	Atomic Number	Atomic Type	Coordinates (Angstroms) X	Y	Z
1	6	0	-0.584667	1.701835	-1.013604
2	6	0	-0.462410	0.358518	-0.277118
3	6	0	-1.408825	-0.703273	-0.869852
4	6	0	-2.770756	-0.810778	-0.183809
5	6	0	-3.658310	0.418734	-0.236749
6	1	0	-1.344723	1.752769	-1.813457
7	1	0	-1.571192	-0.489382	-1.936050
8	1	0	-3.310865	-1.597346	-0.724860
9	1	0	-3.988023	0.537389	-1.276917
10	1	0	-0.728553	0.552731	0.766784
11	8	0	-2.926777	1.561854	0.179894
12	6	0	-4.871201	0.238261	0.680196
13	1	0	-4.524627	0.369768	1.708658
14	1	0	-5.609615	1.014014	0.462172
15	8	0	-5.511752	-1.001226	0.501648
16	1	0	-4.964873	-1.664696	0.937495
17	8	0	-2.604823	-1.198356	1.167054
18	1	0	-1.947437	-1.906707	1.161307
19	8	0	-0.853004	-1.994810	-0.721637
20	1	0	0.106310	-1.891378	-0.808970
21	8	0	0.119991	2.634451	-0.766687
22	7	0	0.907837	-0.102288	-0.401087
23	6	0	3.208206	0.094064	0.270624
24	6	0	3.701390	-0.785218	-0.693536
25	6	0	4.083442	0.663711	1.190664
26	6	0	5.049356	-1.092305	-0.726254
27	1	0	3.013751	-1.211612	-1.412184
28	6	0	5.435802	0.355025	1.158046
29	1	0	3.699461	1.353781	1.933133
30	6	0	5.918722	-0.523984	0.200322
31	1	0	5.430174	-1.774352	-1.475425
32	1	0	6.111032	0.800342	1.876930
33	1	0	6.973425	-0.765966	0.170924
34	6	0	1.779689	0.436869	0.341194
35	1	0	1.506084	1.192250	1.085100
36	1	0	-3.516898	2.320370	0.230037

Structure 97a (M06-2X/def2-TZVP, DMSO)

Energy (Hartrees): = -936.4656824
No imaginary frequencies

Standard orientation:

Center Number	Atomic Number	Atomic Type	Coordinates (Angstroms) X	Y	Z
1	6	0	-0.684952	1.638439	-0.955941
2	6	0	-0.437872	0.337730	-0.184683
3	6	0	-1.353372	-0.790338	-0.680252
4	6	0	-2.731423	-0.843404	-0.022930
5	6	0	-3.596158	0.386442	-0.216158
6	1	0	-1.420518	1.593966	-1.777523
7	1	0	-1.487459	-0.688561	-1.764948
8	1	0	-3.254521	-1.671268	-0.519142
9	1	0	-3.739019	0.517929	-1.295753
10	1	0	-0.624703	0.550719	0.872717
11	8	0	-2.940368	1.516840	0.343085
12	6	0	-4.958027	0.229077	0.455487
13	1	0	-4.812228	0.175067	1.537216
14	1	0	-5.556657	1.116142	0.236197
15	8	0	-5.684218	-0.884085	-0.027082
16	1	0	-5.329415	-1.679136	0.386917
17	8	0	-2.609872	-1.111002	1.360848
18	1	0	-1.972320	-1.832813	1.441872
19	8	0	-0.779553	-2.053951	-0.381637
20	1	0	0.168085	-1.984965	-0.564023
21	8	0	-0.075069	2.645214	-0.724099
22	7	0	0.942722	-0.046814	-0.422138
23	6	0	3.251340	0.077137	0.246052
24	6	0	3.741193	-0.700316	-0.805015
25	6	0	4.137090	0.592534	1.189923
26	6	0	5.097780	-0.957244	-0.903214
27	1	0	3.050664	-1.099176	-1.537676
28	6	0	5.498160	0.337279	1.088370
29	1	0	3.753281	1.196182	2.004750
30	6	0	5.978855	-0.438401	0.042946
31	1	0	5.474549	-1.563087	-1.717881
32	1	0	6.181646	0.742070	1.824088
33	1	0	7.039555	-0.641482	-0.038304
34	6	0	1.816007	0.367984	0.395667
35	1	0	1.541487	0.963585	1.272429
36	1	0	-3.450562	2.306884	0.123599

Structure 97c (B3LYP, Gas Phase)

Energy (Hartrees): = -936.4582713
No imaginary frequencies

Standard orientation:

Center Number	Atomic Number	Atomic Type	Coordinates (Angstroms) X	Y	Z
1	6	0	-4.455616	1.029682	0.282385
2	6	0	-3.426719	0.095133	0.096948
3	6	0	-3.730549	-1.160663	-0.455565
4	6	0	-5.038842	-1.469314	-0.810081
5	6	0	-6.060147	-0.531349	-0.620379
6	6	0	-5.767042	0.718727	-0.074279
7	6	0	-2.055055	0.448823	0.489844
8	7	0	-1.073723	-0.360761	0.384083
9	6	0	0.241418	0.109647	0.762309
10	6	0	0.977703	0.649821	-0.524884
11	6	0	2.488439	0.966510	-0.350222
12	6	0	3.434138	-0.159551	-0.812025
13	8	0	3.162779	-1.457184	-0.260965
14	6	0	0.955883	-1.021038	1.473514
15	8	0	0.283378	1.815890	-0.943545
16	8	0	1.917774	-0.852140	2.208949
17	6	0	4.912249	0.176145	-0.599513
18	8	0	5.737457	-0.835061	-1.147743
19	8	0	2.778421	1.520054	0.929795
20	1	0	-1.925704	1.465927	0.888688
21	1	0	0.221398	0.959912	1.464728
22	1	0	0.559593	2.530918	-0.350172
23	1	0	0.836009	-0.085611	-1.321421
24	1	0	2.626330	0.831744	1.603462
25	1	0	2.676841	1.786148	-1.054904
26	1	0	5.318959	-1.673411	-0.899638
27	1	0	5.166609	1.116971	-1.098909
28	1	0	5.098038	0.320032	0.477446
29	1	0	3.267909	-0.291127	-1.887809

30	1	0	0.533421	-2.028229	1.305838
31	1	0	-4.223326	2.003192	0.707158
32	1	0	-6.557026	1.448965	0.072829
33	1	0	-7.080732	-0.776199	-0.899729
34	1	0	-5.268312	-2.441075	-1.237251
35	1	0	-2.925745	-1.873908	-0.597316
36	1	0	3.298153	-1.416817	0.698168

Structure 97c (B3LYP, DMSO)

Energy (Hartrees): = -936.4824734
No imaginary frequencies

Standard orientation:

Center Number	Atomic Number	Atomic Type	Coordinates (Angstroms) X	Y	Z
1	6	0	-4.489340	0.981001	0.448581
2	6	0	-3.453784	0.096332	0.109063
3	6	0	-3.762228	-1.087599	-0.584202
4	6	0	-5.079890	-1.377181	-0.923947
5	6	0	-6.107273	-0.490386	-0.578969
6	6	0	-5.810001	0.688850	0.106848
7	6	0	-2.074524	0.432566	0.496438
8	7	0	-1.084005	-0.342988	0.272632
9	6	0	0.232084	0.111598	0.679485
10	6	0	1.023738	0.582905	-0.596660
11	6	0	2.514524	0.956705	-0.344350
12	6	0	3.503462	-0.143137	-0.777198
13	8	0	3.243859	-1.446958	-0.227446
14	6	0	0.899118	-1.021725	1.430808
15	8	0	0.320155	1.680248	-1.164315
16	8	0	1.826939	-0.860197	2.213056
17	6	0	4.961372	0.230845	-0.511104
18	8	0	5.839503	-0.770468	-1.010713
19	8	0	2.749330	1.487570	0.959679
20	1	0	-1.950592	1.399555	1.003595
21	1	0	0.201187	0.976584	1.361361
22	1	0	0.430332	2.433902	-0.562122
23	1	0	0.966527	-0.216620	-1.340399
24	1	0	2.557395	0.787582	1.611148
25	1	0	2.711916	1.790811	-1.027293
26	1	0	5.442184	-1.612469	-0.736955
27	1	0	5.211261	1.169656	-1.016496
28	1	0	5.106997	0.388136	0.569235
29	1	0	3.373650	-0.275929	-1.857112
30	1	0	0.476665	-2.027076	1.255631
31	1	0	-4.253996	1.897887	0.983055
32	1	0	-6.604374	1.379031	0.375235
33	1	0	-7.134806	-0.719989	-0.846014
34	1	0	-5.311247	-2.293442	-1.459506
35	1	0	-2.959865	-1.768765	-0.848816
36	1	0	3.342962	-1.394140	0.736180

Structure 97c (M06-2X/6-311G(d,p), Gas Phase)

Energy (Hartrees): = -936.323411
9No imaginary frequencies

Standard orientation:

Center Number	Atomic Number	Atomic Type	Coordinates (Angstroms) X	Y	Z
1	6	0	-4.415003	1.032897	0.277200
2	6	0	-3.397405	0.097379	0.095062
3	6	0	-3.699703	-1.156794	-0.441886
4	6	0	-5.005655	-1.465064	-0.787232
5	6	0	-6.020539	-0.526760	-0.600911
6	6	0	-5.724954	0.722403	-0.069369
7	6	0	-2.019813	0.448606	0.480654
8	7	0	-1.055875	-0.366324	0.381384
9	6	0	0.258784	0.102503	0.753562
10	6	0	0.965917	0.649615	-0.525697
11	6	0	2.464251	0.970480	-0.358290
12	6	0	3.400221	-0.153045	-0.812347
13	8	0	3.089284	-1.447522	-0.301637
14	6	0	0.984318	-1.038977	1.427938
15	8	0	0.265444	1.814715	-0.913206
16	8	0	1.911397	-0.873789	2.186125
17	6	0	4.862052	0.161270	-0.535766
18	8	0	5.697683	-0.799901	-1.139476
19	8	0	2.753085	1.520989	0.914934
20	1	0	-1.876562	1.466312	0.866428

21	1	0	0.236201	0.937927	1.469345
22	1	0	0.536536	2.517006	-0.311060
23	1	0	0.823315	-0.076356	-1.328517
24	1	0	2.628497	0.840077	1.591003
25	1	0	2.653205	1.788128	-1.061287
26	1	0	5.309613	-1.659333	-0.946908
27	1	0	5.126373	1.136397	-0.949848
28	1	0	5.019342	0.208799	0.551115
29	1	0	3.272653	-0.254131	-1.894377
30	1	0	0.598209	-2.045818	1.199605
31	1	0	-4.178160	2.007305	0.691974
32	1	0	-6.511289	1.453339	0.074665
33	1	0	-7.040518	-0.772100	-0.872464
34	1	0	-5.238429	-2.437364	-1.204641
35	1	0	-2.896399	-1.870118	-0.579798
36	1	0	3.278575	-1.458806	0.643620

Structure 97c (M06-2X/6-311G(d,p), DMSO)

Energy (Hartrees): = -936.3505772
No imaginary frequencies

Standard orientation:

Center Number	Atomic Number	Atomic Type	Coordinates (Angstroms) X	Y	Z
1	6	0	-4.443925	0.991674	0.400274
2	6	0	-3.413261	0.097911	0.105337
3	6	0	-3.707480	-1.107819	-0.539876
4	6	0	-5.017458	-1.411955	-0.879399
5	6	0	-6.045068	-0.516983	-0.578550
6	6	0	-5.757743	0.684430	0.060476
7	6	0	-2.033041	0.449955	0.488821
8	7	0	-1.059308	-0.344512	0.323993
9	6	0	0.255385	0.130579	0.700552
10	6	0	0.997665	0.580488	-0.593665
11	6	0	2.482513	0.952384	-0.389799
12	6	0	3.441440	-0.172906	-0.783244
13	8	0	3.151122	-1.435640	-0.181669
14	6	0	0.948946	-0.985148	1.449287
15	8	0	0.280475	1.673119	-1.135486
16	8	0	1.825897	-0.789651	2.260778
17	6	0	4.894080	0.188735	-0.526769
18	8	0	5.759184	-0.803685	-1.047894
19	8	0	2.740092	1.527217	0.883153
20	1	0	-1.901713	1.443673	0.933753
21	1	0	0.219404	1.003426	1.368297
22	1	0	0.391059	2.419519	-0.532459
23	1	0	0.919039	-0.222505	-1.329730
24	1	0	2.575210	0.863402	1.567227
25	1	0	2.677877	1.754526	-1.106910
26	1	0	5.408937	-1.650595	-0.749232
27	1	0	5.138447	1.130794	-1.022096
28	1	0	5.051501	0.321317	0.552156
29	1	0	3.309633	-0.339682	-1.855982
30	1	0	0.583227	-2.002928	1.238411
31	1	0	-4.212397	1.927336	0.899213
32	1	0	-6.554619	1.381161	0.292862
33	1	0	-7.067892	-0.758255	-0.844989
34	1	0	-5.242406	-2.346157	-1.380893
35	1	0	-2.901143	-1.794611	-0.769521
36	1	0	3.354530	-1.377774	0.760220

Structure 98a (B3LYP, Gas Phase)

Energy (Hartrees): = -936.4681333
No imaginary frequencies
Standard orientation:

Center Number	Atomic Number	Atomic Type	Coordinates (Angstroms) X	Y	Z
1	6	0	0.663082	1.918907	0.163037
2	6	0	0.449767	0.396405	0.109628
3	6	0	1.305085	-0.339551	1.160858
4	6	0	2.717155	-0.715170	0.685923
5	6	0	3.590862	0.430783	0.151312
6	1	0	1.381698	0.296684	2.051668
7	1	0	3.231711	-1.135861	1.565991
8	1	0	3.676445	1.183662	0.943504
9	1	0	0.709074	0.060663	-0.901773
10	8	0	3.018633	1.071066	-0.983712
11	6	0	4.990502	-0.077058	-0.240112

12	1	0	5.651112	0.776890	-0.414719
13	1	0	5.422611	-0.686200	0.567469
14	8	0	4.928490	-0.791325	-1.471256
15	1	0	4.317031	-1.532386	-1.316726
16	8	0	2.616850	-1.718110	-0.331028
17	1	0	1.929713	-2.326709	-0.011990
18	8	0	0.704083	-1.594622	1.491063
19	1	0	-0.251294	-1.421126	1.531153
20	7	0	-0.965757	0.164451	0.407501
21	6	0	-3.247236	0.022500	-0.385905
22	6	0	-3.822295	-0.111479	0.889710
23	6	0	-4.074361	-0.013571	-1.518525
24	6	0	-5.195229	-0.284673	1.021516
25	1	0	-3.176389	-0.071391	1.760247
26	6	0	-5.450900	-0.187082	-1.384011
27	1	0	-3.633311	0.093764	-2.506473
28	6	0	-6.012539	-0.323675	-0.113971
29	1	0	-5.634779	-0.386356	2.009272
30	1	0	-6.082936	-0.214697	-2.266346
31	1	0	-7.084894	-0.457184	-0.006079
32	6	0	-1.799612	0.198683	-0.562633
33	1	0	-1.466293	0.353257	-1.600966
34	1	0	0.244978	2.460112	-0.712431
35	8	0	1.169352	2.510044	1.086677
36	1	0	3.340861	0.549180	-1.739229

Structure 98a (B3LYP, DMSO)

Energy (Hartrees): = -936.4900948
No imaginary frequencies

Standard orientation:

Center Number	Atomic Number	Atomic Type	Coordinates (Angstroms) X	Y	Z
1	6	0	-0.692117	1.878176	0.322806
2	6	0	-0.460377	0.392632	0.019304
3	6	0	-1.298976	-0.103031	-1.177120
4	6	0	-2.712081	-0.582124	-0.817423
5	6	0	-3.632356	0.442276	-0.139581
6	1	0	-1.369009	0.706750	-1.914878
7	1	0	-3.193227	-0.860963	-1.767919
8	1	0	-3.752418	1.285879	-0.828068
9	1	0	-0.691652	-0.183757	0.921882
10	8	0	-3.088774	0.960104	1.075038
11	6	0	-5.012697	-0.160740	0.162642
12	1	0	-5.702737	0.636661	0.453618
13	1	0	-5.419588	-0.654166	-0.731205
14	8	0	-4.937312	-1.055360	1.274966
15	1	0	-4.294298	-1.736812	1.010214
16	8	0	-2.616364	-1.739734	0.026123
17	1	0	-1.922135	-2.289750	-0.373807
18	8	0	-0.678848	-1.255320	-1.760805
19	1	0	0.277234	-1.075696	-1.736562
20	7	0	0.960207	0.283477	-0.336743
21	6	0	3.240543	-0.083103	0.378127
22	6	0	3.817788	0.156375	-0.882156
23	6	0	4.067465	-0.438533	1.455716
24	6	0	5.192708	0.036871	-1.055196
25	1	0	3.178323	0.436764	-1.712960
26	6	0	5.445991	-0.557301	1.279445
27	1	0	3.623349	-0.621528	2.430756
28	6	0	6.010093	-0.320165	0.024411
29	1	0	5.633179	0.223163	-2.030371
30	1	0	6.077684	-0.833163	2.118565
31	1	0	7.083417	-0.410984	-0.115296
32	6	0	1.792645	0.025854	0.603165
33	1	0	1.460233	-0.137876	1.637211
34	1	0	-0.471875	2.166052	1.369637
35	8	0	-0.987023	2.708368	-0.511074
36	1	0	-3.323163	0.297505	1.747571

Structure 98a (M06-2X/6-311G(d,p), Gas Phase)

Energy (Hartrees): = -936.3356629
No imaginary frequencies

Standard orientation:

Center Number	Atomic Number	Atomic Type	Coordinates (Angstroms) X	Y	Z
1	6	0	-0.685689	1.832177	-0.242781
2	6	0	-0.441890	0.325587	-0.105528

3	6	0	-1.283005	-0.469135	-1.106471
4	6	0	-2.705328	-0.757705	-0.627543
5	6	0	-3.520398	0.459523	-0.188141
6	1	0	-1.322165	0.088636	-2.047736
7	1	0	-3.226725	-1.230485	-1.471773
8	1	0	-3.527871	1.179496	-1.011456
9	1	0	-0.680671	0.030337	0.922016
10	8	0	-2.939777	1.101896	0.931886
11	6	0	-4.954229	0.060121	0.173294
12	1	0	-5.556420	0.961191	0.296584
13	1	0	-5.393863	-0.549074	-0.625790
14	8	0	-4.981991	-0.609851	1.420895
15	1	0	-4.420318	-1.390195	1.329375
16	8	0	-2.655542	-1.665467	0.467850
17	1	0	-2.003305	-2.334840	0.226532
18	8	0	-0.717461	-1.756783	-1.309038
19	1	0	0.227812	-1.627435	-1.446930
20	7	0	0.970574	0.121872	-0.409290
21	6	0	3.245360	0.038318	0.379319
22	6	0	3.809848	-0.085312	-0.893562
23	6	0	4.069595	0.023798	1.504182
24	6	0	5.180941	-0.228737	-1.030901
25	1	0	3.156757	-0.061023	-1.757430
26	6	0	5.444924	-0.120318	1.364807
27	1	0	3.630113	0.125002	2.491340
28	6	0	6.000461	-0.247410	0.097377
29	1	0	5.617936	-0.323619	-2.017716
30	1	0	6.080441	-0.131572	2.241881
31	1	0	7.072466	-0.358209	-0.015230
32	6	0	1.790538	0.181850	0.556684
33	1	0	1.447486	0.333567	1.590445
34	1	0	-0.272001	2.435653	0.588508
35	8	0	-1.211689	2.342375	-1.188241
36	1	0	-3.278778	0.625556	1.701175

Structure 98a (M06-2X/6-311G(d,p), DMSO)

Energy (Hartrees): = -936.3601486
No imaginary frequencies

Standard orientation:

Center Number	Atomic Number	Atomic Type	Coordinates (Angstroms) X	Y	Z
1	6	0	-0.702137	1.813987	0.212967
2	6	0	-0.443230	0.322852	0.002814
3	6	0	-1.276206	-0.248323	-1.146072
4	6	0	-2.701581	-0.627405	-0.752170
5	6	0	-3.532488	0.491770	-0.127624
6	1	0	-1.310667	0.486280	-1.957338
7	1	0	-3.208722	-0.948179	-1.671461
8	1	0	-3.525457	1.344730	-0.811139
9	1	0	-0.649840	-0.204123	0.939583
10	8	0	-2.979115	0.931474	1.103881
11	6	0	-4.975107	0.046813	0.107668
12	1	0	-5.573735	0.913021	0.393758
13	1	0	-5.391414	-0.374700	-0.814693
14	8	0	-5.056376	-0.872557	1.188498
15	1	0	-4.476077	-1.611525	0.964319
16	8	0	-2.665251	-1.708520	0.178113
17	1	0	-2.005456	-2.329928	-0.156338
18	8	0	-0.703006	-1.468819	-1.601904
19	1	0	0.246135	-1.317551	-1.692253
20	7	0	0.971145	0.235749	-0.357718
21	6	0	3.248689	-0.069163	0.364913
22	6	0	3.816542	0.175997	-0.890129
23	6	0	4.072499	-0.391655	1.444714
24	6	0	5.191072	0.096210	-1.055847
25	1	0	3.172123	0.431238	-1.723363
26	6	0	5.451010	-0.471614	1.276292
27	1	0	3.627414	-0.577795	2.417001
28	6	0	6.010530	-0.227174	0.026641
29	1	0	5.630272	0.286539	-2.028380
30	1	0	6.085407	-0.723134	2.118131
31	1	0	7.084489	-0.286651	-0.108113
32	6	0	1.793426	0.006499	0.583376
33	1	0	1.455903	-0.155541	1.614606
34	1	0	-0.403460	2.192543	1.207126
35	8	0	-1.104162	2.551419	-0.645966
36	1	0	-3.209749	0.247799	1.746348

Structure 98a (M06-2X/def2-TZVP, Gas Phase)

Energy (Hartrees): = -936.4487738

No imaginary frequencies

Standard orientation:

Center Number	Atomic Number	Atomic Type	Coordinates (Angstroms) X	Y	Z
1	6	0	-0.689498	1.839243	0.152259
2	6	0	-0.437138	0.341318	-0.030253
3	6	0	-1.280032	-0.236441	-1.166173
4	6	0	-2.699931	-0.622042	-0.759450
5	6	0	-3.517283	0.480645	-0.091520
6	1	0	-1.330064	0.502995	-1.970926
7	1	0	-3.218464	-0.902026	-1.686098
8	1	0	-3.519784	1.346183	-0.759377
9	1	0	-0.664006	-0.159726	0.916426
10	8	0	-2.949245	0.895733	1.135072
11	6	0	-4.955607	0.028972	0.159213
12	1	0	-5.558087	0.892718	0.439976
13	1	0	-5.377566	-0.408796	-0.751860
14	8	0	-5.022684	-0.870153	1.248817
15	1	0	-4.461412	-1.627600	1.034010
16	8	0	-2.663327	-1.739819	0.116015
17	1	0	-2.008336	-2.350862	-0.246961
18	8	0	-0.707356	-1.446433	-1.637559
19	1	0	0.237619	-1.290264	-1.756621
20	7	0	0.972900	0.223587	-0.372686
21	6	0	3.247045	-0.054946	0.370988
22	6	0	3.824115	0.168988	-0.879384
23	6	0	4.059009	-0.370074	1.456641
24	6	0	5.194758	0.070707	-1.034553
25	1	0	3.182876	0.425574	-1.712592
26	6	0	5.433733	-0.469410	1.299594
27	1	0	3.608823	-0.538098	2.428505
28	6	0	6.001583	-0.249339	0.053683
29	1	0	5.642051	0.245912	-2.004460
30	1	0	6.059107	-0.716962	2.147227
31	1	0	7.074068	-0.323700	-0.072938
32	6	0	1.793669	0.036769	0.572431
33	1	0	1.449712	-0.079621	1.609107
34	1	0	-0.320084	2.247155	1.111362
35	8	0	-1.175212	2.545509	-0.680958
36	1	0	-3.310706	0.306988	1.812063

Structure 98a (M06-2X/ def2-TZVP, DMSO)

Energy (Hartrees): = -936.4736068
No imaginary frequencies

Standard orientation:

Center Number	Atomic Number	Atomic Type	Coordinates (Angstroms) X	Y	Z
1	6	0	-0.690466	1.792701	0.166770
2	6	0	-0.433304	0.297144	0.012217
3	6	0	-1.271676	-0.323889	-1.103099
4	6	0	-2.704587	-0.665772	-0.707402
5	6	0	-3.527080	0.494905	-0.156735
6	1	0	-1.298851	0.369417	-1.949049
7	1	0	-3.202546	-1.014151	-1.620593
8	1	0	-3.488638	1.308045	-0.885585
9	1	0	-0.627900	-0.190853	0.971601
10	8	0	-2.995012	0.993896	1.059302
11	6	0	-4.983894	0.097080	0.054152
12	1	0	-5.569908	0.988946	0.277564
13	1	0	-5.379783	-0.356394	-0.860432
14	8	0	-5.131972	-0.769570	1.168766
15	1	0	-4.559319	-1.533008	1.011982
16	8	0	-2.708541	-1.706860	0.263706
17	1	0	-2.066290	-2.367204	-0.029942
18	8	0	-0.707200	-1.566338	-1.501987
19	1	0	0.244445	-1.433656	-1.607872
20	7	0	0.977175	0.206768	-0.353288
21	6	0	3.262081	-0.060028	0.358876
22	6	0	3.826766	0.159017	-0.899332
23	6	0	4.087873	-0.350232	1.442653
24	6	0	5.199005	0.085288	-1.063883
25	1	0	3.182853	0.390471	-1.738599
26	6	0	5.464140	-0.424333	1.275926
27	1	0	3.644910	-0.516413	2.418279
28	6	0	6.019982	-0.206171	0.023016
29	1	0	5.635963	0.255971	-2.039948
30	1	0	6.100193	-0.651734	2.122059
31	1	0	7.093247	-0.261035	-0.111117

32	6	0	1.810003	0.010793	0.582494
33	1	0	1.481476	-0.124738	1.619071
34	1	0	-0.433050	2.200265	1.159143
35	8	0	-1.043277	2.508675	-0.730670
36	1	0	-3.262060	0.371249	1.749608

Structure 98c (B3LYP, Gas Phase)

Energy (Hartrees): = -936.4588195
No imaginary frequencies

Standard orientation:

Center Number	Atomic Number	Atomic Type	Coordinates (Angstroms) X	Y	Z
1	6	0	4.227538	0.727345	-1.195260
2	6	0	3.350583	0.068366	-0.321844
3	6	0	3.876371	-0.661425	0.757662
4	6	0	5.251447	-0.726960	0.952110
5	6	0	6.120158	-0.067750	0.074876
6	6	0	5.606361	0.659781	-0.999150
7	6	0	1.900695	0.153193	-0.553823
8	7	0	1.045179	-0.436738	0.185639
9	6	0	-0.364907	-0.283615	-0.143649
10	6	0	-1.095522	0.444493	1.014013
11	6	0	-2.537546	0.906924	0.685880
12	6	0	-3.499080	-0.151303	0.137669
13	8	0	-3.131617	-0.360245	-1.231751
14	6	0	-0.855696	-1.723526	-0.355282
15	8	0	-0.373103	1.598099	1.408487
16	8	0	-1.403564	-2.392783	0.491638
17	6	0	-4.953585	0.312616	0.190482
18	8	0	-5.694999	-0.632885	-0.584296
19	8	0	-2.482641	2.015004	-0.220761
20	1	0	1.594452	0.769813	-1.415480
21	1	0	-0.505453	0.273227	-1.085992
22	1	0	-0.644309	2.289549	0.782360
23	1	0	-1.118028	-0.234887	1.870886
24	1	0	-2.532158	1.614200	-1.105003
25	1	0	-2.941557	1.303533	1.625104
26	1	0	-6.557723	-0.255582	-0.793585
27	1	0	-5.301681	0.339362	1.232757
28	1	0	-5.027795	1.323386	-0.231549
29	1	0	-3.391911	-1.086019	0.701397
30	1	0	-0.593427	-2.151408	-1.346221
31	1	0	3.824000	1.294507	-2.030740
32	1	0	6.277250	1.173364	-1.681398
33	1	0	7.193740	-0.121431	0.230814
34	1	0	5.652193	-1.291169	1.789193
35	1	0	3.186699	-1.163210	1.427776
36	1	0	-3.904057	-0.786432	-1.638422

Structure 98c (B3LYP, DMSO)

Energy (Hartrees): = -936.4848741
No imaginary frequencies

Standard orientation:

Center Number	Atomic Number	Atomic Type	Coordinates (Angstroms) X	Y	Z
1	6	0	-4.238869	-0.503352	-1.307436
2	6	0	-3.354077	-0.023203	-0.329114
3	6	0	-3.874901	0.513771	0.861802
4	6	0	-5.250343	0.565481	1.064422
5	6	0	-6.125642	0.083666	0.083345
6	6	0	-5.617875	-0.450141	-1.102645
7	6	0	-1.905494	-0.098708	-0.579545
8	7	0	-1.034805	0.303261	0.266116
9	6	0	0.374095	0.194064	-0.111143
10	6	0	1.142031	-0.596071	0.977079
11	6	0	2.593262	-0.982594	0.602907
12	6	0	3.505664	0.162678	0.162900
13	8	0	3.097167	0.527602	-1.161947
14	6	0	0.826027	1.655279	-0.237825
15	8	0	0.453052	-1.802786	1.284862
16	8	0	1.211302	2.330256	0.695246
17	6	0	4.977800	-0.238942	0.130969
18	8	0	5.665963	0.835542	-0.521321
19	8	0	2.561823	-2.000411	-0.406776
20	1	0	-1.616134	-0.533844	-1.547002
21	1	0	0.492387	-0.293588	-1.090010

22	1	0	0.713475	-2.418460	0.578747
23	1	0	1.155566	0.006592	1.889951
24	1	0	2.571312	-1.518285	-1.251653
25	1	0	3.021931	-1.448644	1.497022
26	1	0	6.484189	0.482966	-0.898469
27	1	0	5.350146	-0.390067	1.153071
28	1	0	5.093411	-1.176318	-0.426085
29	1	0	3.380319	1.014676	0.844074
30	1	0	0.676113	2.096316	-1.243009
31	1	0	-3.839632	-0.919074	-2.229095
32	1	0	-6.293760	-0.824442	-1.865926
33	1	0	-7.198858	0.125286	0.245770
34	1	0	-5.645632	0.981093	1.986860
35	1	0	-3.189474	0.885534	1.616680
36	1	0	3.863739	0.994236	-1.538544

Structure 98c (M06-2X/6-311G(d,p), Gas Phase)

Energy (Hartrees): = -936.327083
No imaginary frequencies

Standard orientation:

Center Number	Atomic Number	Atomic Type	Coordinates (Angstroms) X	Y	Z
1	6	0	-4.149805	-1.040255	-0.875897
2	6	0	-3.296085	-0.109043	-0.286818
3	6	0	-3.828871	0.918516	0.496076
4	6	0	-5.199042	1.007578	0.681825
5	6	0	-6.049373	0.074268	0.089843
6	6	0	-5.524579	-0.949846	-0.688949
7	6	0	-1.841447	-0.222970	-0.496777
8	7	0	-1.019509	0.607923	-0.013872
9	6	0	0.393583	0.370761	-0.250393
10	6	0	1.012881	-0.348775	0.966367
11	6	0	2.430588	-0.889160	0.703860
12	6	0	3.450719	0.111896	0.176217
13	8	0	3.128607	0.324812	-1.194777
14	6	0	0.970880	1.769167	-0.460195
15	8	0	0.217119	-1.446442	1.349817
16	8	0	1.516620	2.403418	0.398507
17	6	0	4.865188	-0.434872	0.272040
18	8	0	5.675454	0.464453	-0.471388
19	8	0	2.346191	-1.993544	-0.189538
20	1	0	-1.511182	-1.080292	-1.101008
21	1	0	0.556434	-0.228325	-1.158702
22	1	0	0.471022	-2.170099	0.763785
23	1	0	1.031362	0.353948	1.801276
24	1	0	2.443940	-1.620652	-1.075334
25	1	0	2.784368	-1.293291	1.657374
26	1	0	6.546479	0.081980	-0.590928
27	1	0	5.173111	-0.476294	1.322818
28	1	0	4.892435	-1.443179	-0.155461
29	1	0	3.382105	1.053661	0.729455
30	1	0	0.775706	2.204757	-1.458482
31	1	0	-3.733871	-1.839413	-1.481058
32	1	0	-6.183819	-1.676828	-1.147755
33	1	0	-7.120218	0.146413	0.239283
34	1	0	-5.610727	1.803190	1.291204
35	1	0	-3.147301	1.628853	0.948077
36	1	0	3.906974	0.734933	-1.591602

Structure 98c (M06-2X/6-311G(d,p), DMSO)

Energy (Hartrees): = -936.3557393
No imaginary frequencies

Standard orientation:

Center Number	Atomic Number	Atomic Type	Coordinates (Angstroms) X	Y	Z
1	6	0	-4.187846	-0.642818	-1.240579
2	6	0	-3.317855	-0.045929	-0.326594
3	6	0	-3.838120	0.618003	0.789256
4	6	0	-5.210075	0.679669	0.983544
5	6	0	-6.075499	0.080679	0.067146
6	6	0	-5.563933	-0.579868	-1.044708
7	6	0	-1.863942	-0.136800	-0.562550
8	7	0	-1.014092	0.381540	0.222396
9	6	0	0.393344	0.226709	-0.122814
10	6	0	1.120132	-0.503503	1.019274
11	6	0	2.545385	-0.949939	0.657392
12	6	0	3.465841	0.150027	0.147859

13	8	0	3.042179	0.452169	-1.179499
14	6	0	0.877582	1.663849	-0.311528
15	8	0	0.396677	-1.657624	1.399813
16	8	0	1.299630	2.345455	0.584543
17	6	0	4.915933	-0.295132	0.117344
18	8	0	5.620121	0.730403	-0.574775
19	8	0	2.472176	-2.003608	-0.297284
20	1	0	-1.561141	-0.691091	-1.459755
21	1	0	0.513817	-0.316908	-1.069028
22	1	0	0.600137	-2.320810	0.727344
23	1	0	1.153344	0.158523	1.886644
24	1	0	2.483792	-1.577518	-1.164932
25	1	0	2.978357	-1.380105	1.564824
26	1	0	6.480848	0.388551	-0.834776
27	1	0	5.288279	-0.415731	1.139833
28	1	0	4.995487	-1.250485	-0.411766
29	1	0	3.372584	1.036718	0.784776
30	1	0	0.722004	2.076964	-1.323680
31	1	0	-3.781152	-1.156210	-2.106051
32	1	0	-6.234765	-1.044247	-1.758129
33	1	0	-7.147035	0.129810	0.223501
34	1	0	-5.610775	1.192434	1.850350
35	1	0	-3.156036	1.077593	1.495111
36	1	0	3.788955	0.897695	-1.601681

Structure 99a (B3LYP, Gas Phase)

Energy (Hartrees): = -936.4613851
No imaginary frequencies

Standard orientation:

Center Number	Atomic Number	Atomic Type	Coordinates (Angstroms) X	Y	Z
1	6	0	2.693988	0.446787	-0.406289
2	8	0	2.885221	1.852344	-0.297683
3	6	0	0.138350	2.298336	0.687170
4	6	0	0.197068	0.975592	-0.102235
5	6	0	1.408325	0.091681	0.346273
6	1	0	2.552752	0.157832	-1.461317
7	1	0	1.128685	2.745713	0.885596
8	1	0	0.354615	1.272298	-1.152977
9	1	0	1.575122	0.253013	1.426203
10	7	0	-1.012457	0.192974	0.050882
11	6	0	-3.403299	-0.029169	-0.223461
12	6	0	-3.515296	-1.225344	0.505127
13	6	0	-4.549682	0.518104	-0.818267
14	6	0	-4.747007	-1.858575	0.626320
15	1	0	-2.628377	-1.637505	0.974046
16	6	0	-5.784228	-0.117897	-0.696566
17	1	0	-4.468640	1.447455	-1.376440
18	6	0	-5.884311	-1.307771	0.025062
19	1	0	-4.826775	-2.781810	1.192679
20	1	0	-6.665135	0.314394	-1.161443
21	1	0	-6.845059	-1.804620	0.123543
22	6	0	-2.118080	0.667355	-0.381474
23	1	0	-2.167776	1.625157	-0.913133
24	8	0	1.125177	-1.268828	0.087749
25	1	0	0.157509	-1.339735	0.200260
26	6	0	3.949841	-0.259864	0.124403
27	1	0	4.018033	-0.090780	1.211731
28	6	0	4.027335	-1.758533	-0.145330
29	1	0	3.270972	-2.295636	0.434389
30	1	0	3.849880	-1.948643	-1.213084
31	8	0	5.040659	0.389970	-0.531957
32	1	0	5.812930	-0.173522	-0.369907
33	8	0	5.359884	-2.137038	0.228806
34	1	0	5.524776	-3.030688	-0.094217
35	8	0	-0.891607	2.824011	1.042567
36	1	0	3.810981	2.008371	-0.542373

Structure 99a (B3LYP, DMSO)

Energy (Hartrees): = -936.482661
No imaginary frequencies

Standard orientation:

Center Number	Atomic Number	Atomic Type	Coordinates (Angstroms) X	Y	Z
1	6	0	2.675932	0.426129	-0.440079

2	8	0	2.876558	1.838136	-0.400695
3	6	0	0.165647	2.308431	0.740804
4	6	0	0.194220	1.000676	-0.057919
5	6	0	1.412776	0.110432	0.367317
6	1	0	2.501844	0.094604	-1.476536
7	1	0	1.153286	2.791861	0.846296
8	1	0	0.320563	1.304693	-1.110027
9	1	0	1.618501	0.288527	1.436465
10	7	0	-1.019595	0.229832	0.134866
11	6	0	-3.391997	-0.035371	-0.249987
12	6	0	-3.550869	-1.143911	0.600943
13	6	0	-4.500005	0.440231	-0.969166
14	6	0	-4.791638	-1.761344	0.721951
15	1	0	-2.697278	-1.507518	1.163937
16	6	0	-5.743151	-0.180353	-0.846370
17	1	0	-4.380708	1.298550	-1.625285
18	6	0	-5.890685	-1.282007	-0.001413
19	1	0	-4.907966	-2.616122	1.381878
20	1	0	-6.593659	0.194739	-1.407845
21	1	0	-6.857955	-1.766264	0.097289
22	6	0	-2.097694	0.643383	-0.414576
23	1	0	-2.111472	1.529344	-1.061780
24	8	0	1.097162	-1.254920	0.149785
25	1	0	0.136617	-1.308765	0.319043
26	6	0	3.947096	-0.256240	0.084689
27	1	0	4.057693	-0.016126	1.154962
28	6	0	4.010206	-1.768972	-0.088620
29	1	0	3.279135	-2.265662	0.556429
30	1	0	3.793369	-2.034841	-1.131527
31	8	0	5.020989	0.339055	-0.653353
32	1	0	5.780610	-0.248277	-0.506589
33	8	0	5.353661	-2.137852	0.261570
34	1	0	5.531822	-3.006654	-0.124616
35	8	0	-0.837982	2.800061	1.211917
36	1	0	3.790642	1.976742	-0.695401

Structure 99a (M06-2X/6-311G(d,p), Gas Phase)

Energy (Hartrees): -936.3279096
No imaginary frequencies

Standard orientation:

Center Number	Atomic Number	Atomic Type	Coordinates (Angstroms) X	Y	Z
1	6	0	2.673207	0.443252	-0.430708
2	8	0	2.807426	1.848066	-0.323425
3	6	0	0.163234	2.206421	0.782826
4	6	0	0.206901	0.933477	-0.080615
5	6	0	1.406036	0.034035	0.305782
6	1	0	2.549664	0.149033	-1.483063
7	1	0	1.150622	2.604196	1.067364
8	1	0	0.342848	1.277900	-1.117054
9	1	0	1.569687	0.116795	1.392709
10	7	0	-1.008687	0.166123	0.069973
11	6	0	-3.388295	-0.014855	-0.223156
12	6	0	-3.510044	-1.199975	0.506932
13	6	0	-4.516839	0.541094	-0.824228
14	6	0	-4.743667	-1.819885	0.623510
15	1	0	-2.627863	-1.613630	0.979785
16	6	0	-5.754419	-0.081380	-0.707242
17	1	0	-4.423255	1.466701	-1.382704
18	6	0	-5.867954	-1.262528	0.015507
19	1	0	-4.835208	-2.738256	1.190965
20	1	0	-6.627358	0.355958	-1.176234
21	1	0	-6.831665	-1.748678	0.110960
22	6	0	-2.087917	0.662074	-0.372678
23	1	0	-2.109043	1.619451	-0.905315
24	8	0	1.146815	-1.304265	-0.048228
25	1	0	0.200701	-1.434561	0.106491
26	6	0	3.938659	-0.210475	0.106746
27	1	0	4.037848	0.037338	1.173576
28	6	0	4.005326	-1.717459	-0.061164
29	1	0	3.276713	-2.209385	0.585683
30	1	0	3.789988	-1.976845	-1.104142
31	8	0	4.995512	0.395640	-0.622409
32	1	0	5.782717	-0.131775	-0.447069
33	8	0	5.343637	-2.064396	0.286816
34	1	0	5.486851	-2.989730	0.082343
35	8	0	-0.858153	2.739186	1.108354
36	1	0	3.700303	2.056005	-0.622030

Structure 99a (M06-2X/6-311G(d,p), DMSO)

Energy (Hartrees): = -936.3516839
No imaginary frequencies

Standard orientation:

Center Number	Atomic Number	Atomic Type	Coordinates (Angstroms) X	Y	Z
1	6	0	2.662955	0.428173	-0.467161
2	8	0	2.795103	1.839289	-0.409238
3	6	0	0.188945	2.196522	0.819286
4	6	0	0.203652	0.942386	-0.058139
5	6	0	1.408244	0.035224	0.301568
6	1	0	2.525129	0.102061	-1.507719
7	1	0	1.179653	2.640379	1.004959
8	1	0	0.312332	1.298219	-1.093481
9	1	0	1.595680	0.112530	1.383778
10	7	0	-1.014985	0.182774	0.121100
11	6	0	-3.384661	-0.019041	-0.241106
12	6	0	-3.528198	-1.186535	0.516145
13	6	0	-4.500487	0.534798	-0.870877
14	6	0	-4.771652	-1.789470	0.633731
15	1	0	-2.660163	-1.610471	1.007714
16	6	0	-5.747751	-0.070165	-0.750361
17	1	0	-4.387235	1.442757	-1.454754
18	6	0	-5.883716	-1.232625	0.000725
19	1	0	-4.879218	-2.695474	1.218911
20	1	0	-6.610160	0.365834	-1.240854
21	1	0	-6.854165	-1.706198	0.096883
22	6	0	-2.076703	0.645067	-0.395209
23	1	0	-2.075456	1.558018	-1.001449
24	8	0	1.125225	-1.302783	-0.049906
25	1	0	0.196133	-1.440993	0.181442
26	6	0	3.939115	-0.198768	0.076781
27	1	0	4.053486	0.098001	1.129153
28	6	0	4.010179	-1.710215	-0.020759
29	1	0	3.285638	-2.174444	0.651235
30	1	0	3.800204	-2.024946	-1.049203
31	8	0	4.987884	0.375101	-0.696204
32	1	0	5.772444	-0.156672	-0.515841
33	8	0	5.348046	-2.043549	0.347233
34	1	0	5.507495	-2.960617	0.105656
35	8	0	-0.813452	2.679701	1.270972
36	1	0	3.681113	2.038436	-0.734747

Structure 99a (M06-2X/ def2-TZVP, Gas Phase)

Energy (Hartrees): = -936.4426871
No imaginary frequencies

Standard orientation:

Center Number	Atomic Number	Atomic Type	Coordinates (Angstroms) X	Y	Z
1	6	0	2.668440	0.441804	-0.435439
2	8	0	2.796326	1.846969	-0.339919
3	6	0	0.156750	2.193416	0.809394
4	6	0	0.206852	0.928126	-0.058686
5	6	0	1.411765	0.034223	0.318929
6	1	0	2.535126	0.141202	-1.483823
7	1	0	1.138679	2.588459	1.112508
8	1	0	0.340191	1.283036	-1.090888
9	1	0	1.591619	0.127478	1.401130
10	7	0	-1.006713	0.163156	0.085962
11	6	0	-3.381651	-0.023112	-0.231113
12	6	0	-3.518020	-1.195864	0.511067
13	6	0	-4.499565	0.532040	-0.846200
14	6	0	-4.754830	-1.803614	0.626022
15	1	0	-2.644898	-1.612207	0.995986
16	6	0	-5.740755	-0.077812	-0.730831
17	1	0	-4.394274	1.449070	-1.414590
18	6	0	-5.868514	-1.246282	0.004493
19	1	0	-4.857497	-2.713328	1.203468
20	1	0	-6.605834	0.360420	-1.210968
21	1	0	-6.835687	-1.723143	0.099535
22	6	0	-2.079614	0.645520	-0.378176
23	1	0	-2.092073	1.590399	-0.931274
24	8	0	1.147134	-1.306476	-0.014851
25	1	0	0.197509	-1.434832	0.129637
26	6	0	3.943369	-0.200637	0.091122
27	1	0	4.056092	0.061873	1.152125
28	6	0	4.006960	-1.708032	-0.054247
29	1	0	3.285919	-2.185973	0.609174

30	1	0	3.778158	-1.987256	-1.087723
31	8	0	4.989379	0.397555	-0.657321
32	1	0	5.787532	-0.117720	-0.489256
33	8	0	5.344098	-2.061236	0.285010
34	1	0	5.484475	-2.992431	0.100693
35	8	0	-0.865791	2.727878	1.125677
36	1	0	3.685670	2.066679	-0.644249

Structure 99a (M06-2X/ def2-TZVP, DMSO)

Energy (Hartrees): = -936.4669643
No imaginary frequencies

Standard orientation:

Center Number	Atomic Number	Atomic Type	Coordinates (Angstroms) X	Y	Z
1	6	0	2.656233	0.425261	-0.477066
2	8	0	2.787037	1.836091	-0.436755
3	6	0	0.191866	2.195437	0.840818
4	6	0	0.203531	0.945555	-0.034596
5	6	0	1.412937	0.040084	0.313473
6	1	0	2.505329	0.089581	-1.511767
7	1	0	1.179078	2.647402	1.018562
8	1	0	0.307710	1.306542	-1.067771
9	1	0	1.619228	0.126735	1.390178
10	7	0	-1.013810	0.190445	0.143965
11	6	0	-3.376546	-0.027661	-0.247365
12	6	0	-3.530393	-1.191758	0.507737
13	6	0	-4.484220	0.531191	-0.880677
14	6	0	-4.775900	-1.784681	0.621412
15	1	0	-2.668690	-1.623883	1.001239
16	6	0	-5.734037	-0.062965	-0.763737
17	1	0	-4.361899	1.436743	-1.464606
18	6	0	-5.880213	-1.221157	-0.013491
19	1	0	-4.891724	-2.689365	1.205303
20	1	0	-6.590830	0.378631	-1.256847
21	1	0	-6.853290	-1.687399	0.080321
22	6	0	-2.068747	0.631418	-0.397373
23	1	0	-2.060014	1.529580	-1.023738
24	8	0	1.118498	-1.299169	-0.017091
25	1	0	0.185326	-1.431326	0.208415
26	6	0	3.941273	-0.191938	0.054986
27	1	0	4.076011	0.130409	1.096368
28	6	0	4.002722	-1.703518	-0.002531
29	1	0	3.284211	-2.141397	0.691133
30	1	0	3.777805	-2.049546	-1.016295
31	8	0	4.976851	0.360614	-0.748811
32	1	0	5.775229	-0.151487	-0.566058
33	8	0	5.338202	-2.045259	0.360730
34	1	0	5.486324	-2.974743	0.157197
35	8	0	-0.807161	2.674751	1.303762
36	1	0	3.668419	2.042562	-0.773611

Structure 99c (B3LYP, Gas Phase)

Energy (Hartrees): = -936.4583711
No imaginary frequencies

Standard orientation:

Center Number	Atomic Number	Atomic Type	Coordinates (Angstroms) X	Y	Z
1	6	0	-2.838721	0.352773	-0.075774
2	8	0	-3.241518	1.665151	-0.520795
3	6	0	-0.290119	2.327740	0.036815
4	6	0	-0.297705	0.806775	0.258196
5	6	0	-1.405846	0.066846	-0.557928
6	1	0	-2.878972	0.403530	1.016762
7	1	0	-0.541701	0.667727	1.326575
8	1	0	-1.333886	0.406079	-1.611158
9	7	0	0.963565	0.174841	-0.076727
10	6	0	3.347690	-0.033566	0.248553
11	6	0	3.550800	-0.928199	-0.815858
12	6	0	4.439044	0.338421	1.047389
13	6	0	4.818413	-1.440639	-1.066488
14	1	0	2.704957	-1.202948	-1.436753
15	6	0	5.709567	-0.176757	0.795477
16	1	0	4.287088	1.035222	1.867815
17	6	0	5.900563	-1.067621	-0.261219
18	1	0	4.969332	-2.130233	-1.891725
19	1	0	6.547541	0.116925	1.420272

20	1	0	6.889587	-1.469263	-0.461191
21	6	0	2.022119	0.524743	0.549770
22	1	0	1.999151	1.259210	1.363965
23	8	0	-1.157142	-1.318832	-0.485387
24	1	0	-0.187908	-1.395386	-0.562096
25	6	0	-3.891709	-0.689258	-0.526991
26	1	0	-3.673601	-1.014592	-1.557343
27	6	0	-3.957380	-1.911480	0.398063
28	1	0	-4.662188	-2.633502	-0.042834
29	1	0	-2.978592	-2.382644	0.481270
30	8	0	-5.188767	-0.094938	-0.460150
31	1	0	-5.046556	0.863764	-0.523738
32	8	0	-4.366669	-1.521065	1.699113
33	1	0	-5.157277	-0.978723	1.549807
34	8	0	0.713349	2.983850	-0.128042
35	1	0	-3.103429	1.704508	-1.479941
36	1	0	-1.292819	2.794451	0.059457

Structure 99c (B3LYP, DMSO)

Energy (Hartrees): = -936.3504885
No imaginary frequencies

Standard orientation:

Center Number	Atomic Number	Atomic Type	Coordinates (Angstroms) X	Y	Z
1	6	0	-2.783447	0.349087	-0.159094
2	8	0	-3.005879	1.582152	-0.847612
3	6	0	-0.378794	2.124165	0.259917
4	6	0	-0.282576	0.608246	0.367188
5	6	0	-1.359478	-0.122100	-0.455633
6	1	0	-2.905606	0.582815	0.902719
7	1	0	-0.471191	0.376160	1.426456
8	1	0	-1.155769	0.076080	-1.519925
9	7	0	1.000130	0.088576	-0.070520
10	6	0	3.377320	-0.057828	0.271265
11	6	0	3.624861	-0.800645	-0.888057
12	6	0	4.438060	0.282178	1.112742
13	6	0	4.917817	-1.197457	-1.194758
14	1	0	2.796925	-1.056487	-1.539077
15	6	0	5.734630	-0.116571	0.803572
16	1	0	4.241667	0.859762	2.010518
17	6	0	5.974582	-0.856362	-0.349285
18	1	0	5.108866	-1.771029	-2.094432
19	1	0	6.554956	0.149632	1.459745
20	1	0	6.984053	-1.166729	-0.593845
21	6	0	2.016300	0.383668	0.628151
22	1	0	1.938805	0.980518	1.545229
23	8	0	-1.246086	-1.506691	-0.204895
24	1	0	-0.318698	-1.729022	-0.361979
25	6	0	-3.869984	-0.663581	-0.556534
26	1	0	-3.596911	-1.138377	-1.508284
27	6	0	-4.114065	-1.729300	0.505353
28	1	0	-4.843162	-2.446223	0.107650
29	1	0	-3.194295	-2.258025	0.742095
30	8	0	-5.113233	0.018304	-0.683026
31	1	0	-4.907061	0.921255	-0.953941
32	8	0	-4.594264	-1.135224	1.698660
33	1	0	-5.298440	-0.539255	1.415616
34	8	0	0.504946	2.830054	-0.146093
35	1	0	-2.740295	1.460812	-1.769734
36	1	0	-1.329976	2.548318	0.619585

Structure 99c (M06-2X/6-311G(d,p), Gas Phase)

Energy (Hartrees): = -936.3229165
No imaginary frequencies

Standard orientation:

Center Number	Atomic Number	Atomic Type	Coordinates (Angstroms) X	Y	Z
1	6	0	-2.801556	0.346605	-0.195190
2	8	0	-3.083434	1.547494	-0.919788
3	6	0	-0.360057	2.170225	0.156335
4	6	0	-0.299381	0.651061	0.326900
5	6	0	-1.367019	-0.091129	-0.500829
6	1	0	-2.917523	0.614843	0.859310
7	1	0	-0.533814	0.451315	1.384672
8	1	0	-1.168329	0.123438	-1.565108
9	7	0	0.978867	0.076582	-0.037512
10	6	0	3.363639	-0.016287	0.249951

11	6	0	3.575722	-0.904797	-0.807562
12	6	0	4.444641	0.405280	1.023192
13	6	0	4.853320	-1.366540	-1.079031
14	1	0	2.727107	-1.213049	-1.405925
15	6	0	5.725955	-0.060077	0.751536
16	1	0	4.279369	1.102139	1.838257
17	6	0	5.930159	-0.946426	-0.298863
18	1	0	5.016840	-2.054266	-1.899929
19	1	0	6.562260	0.268999	1.356177
20	1	0	6.928059	-1.309425	-0.514344
21	6	0	2.015664	0.486483	0.566350
22	1	0	1.966076	1.232218	1.368277
23	8	0	-1.227189	-1.470404	-0.268220
24	1	0	-0.284037	-1.655775	-0.365210
25	6	0	-3.861276	-0.719663	-0.516054
26	1	0	-3.592071	-1.243318	-1.443774
27	6	0	-4.033734	-1.724542	0.621065
28	1	0	-4.752248	-2.485589	0.293223
29	1	0	-3.088530	-2.204606	0.859455
30	8	0	-5.126776	-0.090855	-0.648396
31	1	0	-4.965273	0.814339	-0.936555
32	8	0	-4.490168	-1.062466	1.782110
33	1	0	-5.263714	-0.561614	1.500117
34	8	0	0.590478	2.848535	-0.111910
35	1	0	-2.828659	1.410414	-1.838856
36	1	0	-1.357329	2.614278	0.315631

Structure 99c (M06-2X/6-311G(d,p), DMSO)

Energy (Hartrees): = -936.3504885
No imaginary frequencies

Standard orientation:

Center Number	Atomic Number	Atomic Type	Coordinates (Angstroms) X	Y	Z
1	6	0	-2.783447	0.349087	-0.159094
2	8	0	-3.005879	1.582152	-0.847612
3	6	0	-0.378794	2.124165	0.259917
4	6	0	-0.282576	0.608246	0.367188
5	6	0	-1.359478	-0.122100	-0.455633
6	1	0	-2.905606	0.582815	0.902719
7	1	0	-0.471191	0.376160	1.426456
8	1	0	-1.155769	0.076080	-1.519925
9	7	0	1.000130	0.088576	-0.070520
10	6	0	3.377320	-0.057828	0.271265
11	6	0	3.624861	-0.800645	-0.888057
12	6	0	4.438060	0.282178	1.112742
13	6	0	4.917817	-1.197457	-1.194758
14	1	0	2.796925	-1.056487	-1.539077
15	6	0	5.734630	-0.116571	0.803572
16	1	0	4.241667	0.859762	2.010518
17	6	0	5.974582	-0.856362	-0.349285
18	1	0	5.108866	-1.771029	-2.094432
19	1	0	6.554956	0.149632	1.459745
20	1	0	6.984053	-1.166729	-0.593845
21	6	0	2.016300	0.383668	0.628151
22	1	0	1.938805	0.980518	1.545229
23	8	0	-1.246086	-1.506691	-0.204895
24	1	0	-0.318698	-1.729022	-0.361979
25	6	0	-3.869984	-0.663581	-0.556534
26	1	0	-3.596911	-1.138377	-1.508284
27	6	0	-4.114065	-1.729300	0.505353
28	1	0	-4.843162	-2.446223	0.107650
29	1	0	-3.194295	-2.258025	0.742095
30	8	0	-5.113233	0.018304	-0.683026
31	1	0	-4.907061	0.921255	-0.953941
32	8	0	-4.594264	-1.135224	1.698660
33	1	0	-5.298440	-0.539255	1.415616
34	8	0	0.504946	2.830054	-0.146093
35	1	0	-2.740295	1.460812	-1.769734
36	1	0	-1.329976	2.548318	0.619585

Structure 100a (B3LYP, Gas Phase)

Energy (Hartrees): = -936.4613549
No imaginary frequencies

Standard orientation:

Center Number	Atomic Number	Atomic Type	Coordinates (Angstroms) X	Y	Z
1	6	0	2.515353	-0.132470	-0.569934
2	8	0	2.885003	1.190777	-0.942378

3	6	0	0.469641	2.236524	-0.377742
4	6	0	0.134190	0.747304	-0.572481
5	6	0	1.169729	-0.135876	0.168293
6	1	0	2.384296	-0.705766	-1.496209
7	1	0	0.531417	2.831589	-1.312273
8	1	0	0.158367	0.516559	-1.651177
9	1	0	1.294661	0.280517	1.176901
10	7	0	-1.175107	0.494414	0.009816
11	6	0	-3.487016	-0.135618	-0.298847
12	6	0	-3.843077	0.029156	1.050788
13	6	0	-4.453456	-0.577778	-1.213874
14	6	0	-5.139722	-0.248020	1.469085
15	1	0	-3.089150	0.379454	1.747817
16	6	0	-5.752814	-0.856332	-0.791990
17	1	0	-4.181754	-0.703790	-2.259122
18	6	0	-6.097388	-0.691976	0.550087
19	1	0	-5.410275	-0.116880	2.512679
20	1	0	-6.493750	-1.199447	-1.507785
21	1	0	-7.109249	-0.906497	0.881359
22	6	0	-2.123488	0.147422	-0.772502
23	1	0	-1.966556	0.038664	-1.858043
24	8	0	0.742782	-1.488295	0.218244
25	1	0	-0.088240	-1.496799	0.714021
26	6	0	3.630230	-0.768858	0.270104
27	1	0	3.258768	-1.703364	0.712055
28	6	0	4.878353	-1.057252	-0.560038
29	1	0	4.684121	-1.882055	-1.260630
30	1	0	5.139911	-0.159761	-1.135793
31	8	0	3.948225	0.177571	1.289545
32	1	0	4.835982	-0.064772	1.596926
33	8	0	5.908749	-1.396377	0.372167
34	1	0	6.762913	-1.313439	-0.068210
35	8	0	0.618541	2.746072	0.709017
36	1	0	3.284667	1.569553	-0.139696

Structure 100a (B3LYP, DMSO)

Energy (Hartrees): = -936.4867941
No imaginary frequencies

Standard orientation:

Center Number	Atomic Number	Atomic Type	Coordinates (Angstroms) X	Y	Z
1	6	0	2.533718	-0.095881	-0.590763
2	8	0	2.902843	1.260775	-0.832757
3	6	0	0.493367	2.238449	-0.340407
4	6	0	0.133879	0.767147	-0.584143
5	6	0	1.166223	-0.163659	0.104894
6	1	0	2.442408	-0.590294	-1.564954
7	1	0	0.662448	2.841158	-1.253216
8	1	0	0.137277	0.575760	-1.668097
9	1	0	1.263619	0.170845	1.147311
10	7	0	-1.171249	0.509174	0.015625
11	6	0	-3.484158	-0.125833	-0.287767
12	6	0	-3.827988	-0.037306	1.073116
13	6	0	-4.462501	-0.509878	-1.218137
14	6	0	-5.123606	-0.328299	1.488097
15	1	0	-3.070313	0.261121	1.790564
16	6	0	-5.761034	-0.801555	-0.799805
17	1	0	-4.199524	-0.578155	-2.270671
18	6	0	-6.093422	-0.711107	0.553216
19	1	0	-5.382855	-0.257877	2.540574
20	1	0	-6.510883	-1.098271	-1.527162
21	1	0	-7.103969	-0.937378	0.881066
22	6	0	-2.125113	0.171835	-0.767659
23	1	0	-1.978175	0.085170	-1.853730
24	8	0	0.744150	-1.518590	0.046065
25	1	0	-0.098168	-1.568017	0.523566
26	6	0	3.627830	-0.786712	0.233571
27	1	0	3.251265	-1.753537	0.593695
28	6	0	4.907512	-1.007573	-0.567251
29	1	0	4.750118	-1.783317	-1.328788
30	1	0	5.191757	-0.075975	-1.071775
31	8	0	3.907675	0.079166	1.339063
32	1	0	4.818544	-0.141666	1.598620
33	8	0	5.908755	-1.402239	0.379334
34	1	0	6.775468	-1.196999	0.001587
35	8	0	0.539386	2.740425	0.764601
36	1	0	3.318997	1.549150	-0.000194

Structure 100a (M06-2X/6-311G(d,p), Gas Phase)

Energy (Hartrees): = -936.3273144

No imaginary frequencies

Standard orientation:

Center Number	Atomic Number	Atomic Type	Coordinates (Angstroms) X	Y	Z
1	6	0	-2.471609	0.161765	-0.575385
2	8	0	-2.875057	-1.119332	-1.017337
3	6	0	-0.519654	-2.265736	-0.463563
4	6	0	-0.146070	-0.788589	-0.620759
5	6	0	-1.136396	0.090725	0.160964
6	1	0	-2.323072	0.782063	-1.465449
7	1	0	-0.523765	-2.863090	-1.394069
8	1	0	-0.174006	-0.513971	-1.686395
9	1	0	-1.275545	-0.355641	1.152383
10	7	0	1.171722	-0.604256	-0.045179
11	6	0	3.439913	0.154164	-0.296021
12	6	0	3.821221	-0.197902	1.002128
13	6	0	4.362782	0.763607	-1.144782
14	6	0	5.110136	0.063302	1.438578
15	1	0	3.092047	-0.676896	1.644595
16	6	0	5.655064	1.025883	-0.705023
17	1	0	4.065209	1.035530	-2.152374
18	6	0	6.028713	0.676226	0.587071
19	1	0	5.405465	-0.212208	2.443904
20	1	0	6.367751	1.500331	-1.368591
21	1	0	7.035440	0.878381	0.933043
22	6	0	2.072857	-0.105774	-0.783102
23	1	0	1.876576	0.165939	-1.830086
24	8	0	-0.670718	1.420301	0.250267
25	1	0	0.130556	1.411729	0.782371
26	6	0	-3.550048	0.779611	0.306835
27	1	0	-3.152855	1.687153	0.775826
28	6	0	-4.799458	1.115223	-0.486835
29	1	0	-4.595356	1.950628	-1.166414
30	1	0	-5.094663	0.235148	-1.068313
31	8	0	-3.857718	-0.198271	1.286182
32	1	0	-4.706697	0.055365	1.664947
33	8	0	-5.792062	1.458235	0.471503
34	1	0	-6.649649	1.472453	0.043735
35	8	0	-0.760455	-2.760584	0.599668
36	1	0	-3.236114	-1.560942	-0.235756

Structure 100a (M06-2X/6-311G(d,p), DMSO)

Energy (Hartrees): =-936.3552633
No imaginary frequencies

Standard orientation:

Center Number	Atomic Number	Atomic Type	Coordinates (Angstroms) X	Y	Z
1	6	0	2.495192	-0.126941	-0.588345
2	8	0	2.881843	1.193667	-0.929234
3	6	0	0.518440	2.250982	-0.403079
4	6	0	0.145458	0.783764	-0.607518
5	6	0	1.148272	-0.121135	0.130291
6	1	0	2.375913	-0.685884	-1.521442
7	1	0	0.635294	2.852848	-1.320134
8	1	0	0.155060	0.547960	-1.680066
9	1	0	1.267802	0.269949	1.148295
10	7	0	-1.165511	0.575025	-0.013495
11	6	0	-3.446223	-0.145303	-0.286682
12	6	0	-3.808452	0.072047	1.047024
13	6	0	-4.393336	-0.635730	-1.187089
14	6	0	-5.101591	-0.200428	1.468059
15	1	0	-3.068752	0.455964	1.740108
16	6	0	-5.689902	-0.908934	-0.763151
17	1	0	-4.109489	-0.802816	-2.221267
18	6	0	-6.044550	-0.691864	0.564123
19	1	0	-5.379531	-0.030668	2.501910
20	1	0	-6.420280	-1.291180	-1.466796
21	1	0	-7.054026	-0.904316	0.897228
22	6	0	-2.081070	0.131701	-0.772220
23	1	0	-1.903355	-0.071858	-1.835455
24	8	0	0.700684	-1.460339	0.148722
25	1	0	-0.123412	-1.484036	0.648615
26	6	0	3.573743	-0.781885	0.266682
27	1	0	3.187565	-1.723549	0.672703
28	6	0	4.839014	-1.050981	-0.524750
29	1	0	4.657369	-1.839662	-1.262565
30	1	0	5.146272	-0.136310	-1.042609
31	8	0	3.855981	0.132929	1.319345
32	1	0	4.735168	-0.100969	1.642232

33	8	0	5.820158	-1.448259	0.427934
34	1	0	6.688100	-1.378228	0.019173
35	8	0	0.636805	2.742577	0.688118
36	1	0	3.275134	1.561226	-0.124627

Structure 100a (M06-2X/ def2-TZVP, Gas Phase)

Energy (Hartrees): = -936.4426021
No imaginary frequencies

Standard orientation:

Center Number	Atomic Number	Atomic Type	Coordinates (Angstroms) X	Y	Z
1	6	0	2.481109	-0.135390	-0.562046
2	8	0	2.879345	1.151903	-0.984505
3	6	0	0.509906	2.267663	-0.470410
4	6	0	0.144486	0.789050	-0.611871
5	6	0	1.144387	-0.078126	0.170484
6	1	0	2.337177	-0.740794	-1.462286
7	1	0	0.589356	2.835575	-1.414274
8	1	0	0.176970	0.508319	-1.674518
9	1	0	1.274767	0.368561	1.161979
10	7	0	-1.169719	0.595731	-0.040320
11	6	0	-3.438279	-0.157463	-0.294146
12	6	0	-3.829563	0.194073	0.998247
13	6	0	-4.350440	-0.774001	-1.144930
14	6	0	-5.116301	-0.076498	1.427021
15	1	0	-3.110854	0.679960	1.645623
16	6	0	-5.640528	-1.046246	-0.713069
17	1	0	-4.044968	-1.044574	-2.149279
18	6	0	-6.023335	-0.698028	0.573631
19	1	0	-5.419338	0.198551	2.428985
20	1	0	-6.344706	-1.527372	-1.379150
21	1	0	-7.029109	-0.908009	0.914352
22	6	0	-2.073253	0.107328	-0.777268
23	1	0	-1.879206	-0.153177	-1.826374
24	8	0	0.690273	-1.409764	0.262511
25	1	0	-0.136345	-1.409508	0.757399
26	6	0	3.560888	-0.770404	0.304013
27	1	0	3.164686	-1.688266	0.751038
28	6	0	4.803057	-1.096373	-0.501025
29	1	0	4.580692	-1.896059	-1.214904
30	1	0	5.123831	-0.203731	-1.046512
31	8	0	3.872813	0.177027	1.309056
32	1	0	4.713718	-0.090560	1.698763
33	8	0	5.789856	-1.505622	0.434736
34	1	0	6.650556	-1.521609	0.010656
35	8	0	0.674148	2.796321	0.590257
36	1	0	3.256525	1.590151	-0.207486

Structure 100a (M06-2X/ def2-TZVP, DMSO)

Energy (Hartrees): = -936.4710445
No imaginary frequencies

Standard orientation:

Center Number	Atomic Number	Atomic Type	Coordinates (Angstroms) X	Y	Z
1	6	0	2.502158	-0.083969	-0.565629
2	8	0	2.894909	1.242669	-0.865463
3	6	0	0.507058	2.274629	-0.412266
4	6	0	0.144104	0.804519	-0.592776
5	6	0	1.154721	-0.088175	0.149498
6	1	0	2.379867	-0.609913	-1.516522
7	1	0	0.677969	2.846364	-1.338345
8	1	0	0.160999	0.558493	-1.661765
9	1	0	1.268036	0.303491	1.167063
10	7	0	-1.165175	0.588362	-0.006655
11	6	0	-3.437473	-0.155572	-0.284274
12	6	0	-3.817134	0.067662	1.040550
13	6	0	-4.367004	-0.663777	-1.188839
14	6	0	-5.108859	-0.217353	1.448411
15	1	0	-3.093161	0.465307	1.740842
16	6	0	-5.662448	-0.949542	-0.778625
17	1	0	-4.069125	-0.835492	-2.217215
18	6	0	-6.033783	-0.726786	0.539943
19	1	0	-5.400671	-0.042818	2.476613
20	1	0	-6.379405	-1.345390	-1.486901
21	1	0	-7.043191	-0.949368	0.863252
22	6	0	-2.074120	0.131409	-0.761070
23	1	0	-1.888875	-0.077219	-1.820914

24	8	0	0.713508	-1.427650	0.172754
25	1	0	-0.136749	-1.453926	0.629915
26	6	0	3.576247	-0.782358	0.256770
27	1	0	3.186702	-1.742884	0.609116
28	6	0	4.835295	-1.022067	-0.548284
29	1	0	4.627593	-1.731133	-1.355132
30	1	0	5.185266	-0.080587	-0.981641
31	8	0	3.860804	0.065792	1.361241
32	1	0	4.727584	-0.197883	1.697476
33	8	0	5.799225	-1.543422	0.358787
34	1	0	6.669193	-1.500714	-0.052473
35	8	0	0.564987	2.806125	0.664414
36	1	0	3.304173	1.595315	-0.061110

Structure 100c (B3LYP, Gas Phase)

Energy (Hartrees): = -936.4560112
No imaginary frequencies

Standard orientation:

Center Number	Atomic Number	Atomic Type	Coordinates (Angstroms) X	Y	Z
1	6	0	2.531648	-0.274487	-0.815763
2	8	0	2.617453	1.113004	-1.165849
3	6	0	0.480629	1.820636	0.635160
4	6	0	0.163081	0.620262	-0.263567
5	6	0	1.177066	-0.532313	-0.113968
6	1	0	2.529739	-0.895603	-1.722341
7	1	0	0.161678	1.001057	-1.298156
8	1	0	1.349165	-0.677212	0.960264
9	7	0	-1.151939	0.132992	0.142384
10	6	0	-3.544897	0.111659	-0.208117
11	6	0	-3.835840	-0.655464	0.933116
12	6	0	-4.592323	0.514494	-1.049735
13	6	0	-5.148881	-1.011950	1.218015
14	1	0	-3.019335	-0.956094	1.580930
15	6	0	-5.908404	0.156254	-0.761890
16	1	0	-4.370887	1.109367	-1.932465
17	6	0	-6.187875	-0.607683	0.371859
18	1	0	-5.368652	-1.603937	2.101440
19	1	0	-6.712683	0.472067	-1.419277
20	1	0	-7.212389	-0.887305	0.598968
21	6	0	-2.168053	0.503136	-0.539782
22	1	0	-2.060062	1.139393	-1.433139
23	8	0	0.660727	-1.723011	-0.696136
24	1	0	-0.171017	-1.908310	-0.235357
25	6	0	3.703391	-0.698346	0.079638
26	1	0	3.568985	-1.760744	0.324270
27	6	0	5.079187	-0.534724	-0.579001
28	1	0	5.183933	-1.217902	-1.430304
29	1	0	5.185772	0.498758	-0.958373
30	8	0	3.715648	0.010271	1.318192
31	1	0	3.228369	0.844915	1.206678
32	8	0	6.105306	-0.835620	0.342481
33	1	0	5.824173	-0.405007	1.166238
34	8	0	1.448198	1.935825	1.352942
35	1	0	3.372037	1.228126	-1.756033
36	1	0	-0.296282	2.614885	0.601145

Structure 100c (B3LYP, DMSO)

Energy (Hartrees): = -936.4852705
No imaginary frequencies

Standard orientation:

Center Number	Atomic Number	Atomic Type	Coordinates (Angstroms) X	Y	Z
1	6	0	2.551423	-0.023949	-0.568337
2	8	0	2.910251	1.367716	-0.626947
3	6	0	0.431823	2.295045	-0.153131
4	6	0	0.141521	0.827342	-0.493498
5	6	0	1.202775	-0.106660	0.148930
6	1	0	2.428650	-0.435553	-1.577892
7	1	0	0.160602	0.708237	-1.587333
8	1	0	1.327274	0.202619	1.196224
9	7	0	-1.156499	0.472182	0.074363
10	6	0	-3.473812	-0.109832	-0.301477
11	6	0	-3.799706	-0.262007	1.058187
12	6	0	-4.467175	-0.316569	-1.271603
13	6	0	-5.092153	-0.614788	1.432868
14	1	0	-3.031165	-0.101599	1.807447

15	6	0	-5.762777	-0.669410	-0.893482
16	1	0	-4.218133	-0.199379	-2.323191
17	6	0	-6.076881	-0.819588	0.458603
18	1	0	-5.337222	-0.731365	2.484614
19	1	0	-6.524429	-0.826692	-1.651484
20	1	0	-7.084896	-1.094872	0.755263
21	6	0	-2.119271	0.259484	-0.741257
22	1	0	-1.983043	0.342249	-1.828885
23	8	0	0.789670	-1.462273	0.067988
24	1	0	-0.067631	-1.511335	0.519174
25	6	0	3.654786	-0.794304	0.175170
26	1	0	3.265143	-1.765846	0.492938
27	6	0	4.907900	-1.013454	-0.676245
28	1	0	4.693666	-1.703247	-1.499814
29	1	0	5.230698	-0.055178	-1.119409
30	8	0	4.021715	-0.092247	1.369168
31	1	0	4.046262	0.845204	1.108043
32	8	0	5.946796	-1.581997	0.108211
33	1	0	5.908997	-1.089916	0.945052
34	8	0	0.411095	2.735430	0.977325
35	1	0	3.425691	1.518989	-1.432699
36	1	0	0.628094	2.951525	-1.022907

Structure 100c (M06-2X/6-311G(d,p), Gas Phase)

Energy (Hartrees): = -936.3229813
No imaginary frequencies

Standard orientation:

Center Number	Atomic Number	Atomic Type	Coordinates (Angstroms) X	Y	Z
1	6	0	2.480119	-0.388385	-0.714262
2	8	0	2.642788	0.900677	-1.299711
3	6	0	0.524495	2.014509	0.152332
4	6	0	0.159993	0.643503	-0.415481
5	6	0	1.143767	-0.436121	0.042392
6	1	0	2.420480	-1.158172	-1.492388
7	1	0	0.153059	0.725020	-1.512571
8	1	0	1.322320	-0.288334	1.112833
9	7	0	-1.153544	0.326424	0.118327
10	6	0	-3.511273	0.020590	-0.253319
11	6	0	-3.812465	-0.201927	1.093528
12	6	0	-4.526754	-0.037662	-1.207092
13	6	0	-5.115025	-0.482364	1.473354
14	1	0	-3.011800	-0.145807	1.820912
15	6	0	-5.833050	-0.319805	-0.824429
16	1	0	-4.290956	0.136771	-2.251838
17	6	0	-6.126811	-0.542438	0.515414
18	1	0	-5.348322	-0.653469	2.517245
19	1	0	-6.618295	-0.365630	-1.568931
20	1	0	-7.144125	-0.761602	0.816839
21	6	0	-2.134276	0.315906	-0.684433
22	1	0	-2.000856	0.520483	-1.756300
23	8	0	0.622253	-1.728580	-0.197838
24	1	0	-0.165838	-1.822752	0.346338
25	6	0	3.634232	-0.698145	0.232429
26	1	0	3.433700	-1.662574	0.712104
27	6	0	4.986109	-0.777795	-0.465785
28	1	0	5.008883	-1.618769	-1.163972
29	1	0	5.159447	0.152225	-1.029576
30	8	0	3.721950	0.271810	1.263650
31	1	0	3.298907	1.087589	0.960443
32	8	0	6.009129	-0.977476	0.477947
33	1	0	5.814663	-0.363737	1.196041
34	8	0	1.462762	2.233825	0.864042
35	1	0	3.345576	0.853205	-1.951092
36	1	0	-0.187467	2.819336	-0.116985

Structure 100c (M06-2X/6-311G(d,p), DMSO)

Energy (Hartrees): = -936.3533126
No imaginary frequencies

Standard orientation:

Center Number	Atomic Number	Atomic Type	Coordinates (Angstroms) X	Y	Z
1	6	0	2.498315	-0.127226	-0.585822
2	8	0	2.873375	1.225837	-0.844560
3	6	0	0.525764	2.287136	-0.359513
4	6	0	0.156063	0.822546	-0.595514
5	6	0	1.157993	-0.093085	0.134783

6	1	0	2.367077	-0.682329	-1.521252
7	1	0	0.172809	0.605415	-1.671950
8	1	0	1.291480	0.298518	1.150355
9	7	0	-1.154693	0.594628	-0.009642
10	6	0	-3.426096	-0.146916	-0.294621
11	6	0	-3.793646	0.063334	1.038750
12	6	0	-4.365049	-0.647822	-1.197771
13	6	0	-5.083905	-0.226508	1.457014
14	1	0	-3.060148	0.455620	1.733748
15	6	0	-5.658658	-0.939022	-0.776450
16	1	0	-4.077264	-0.809300	-2.231738
17	6	0	-6.018618	-0.728795	0.550471
18	1	0	-5.366039	-0.061926	2.490558
19	1	0	-6.382769	-1.329582	-1.482026
20	1	0	-7.025824	-0.955263	0.881173
21	6	0	-2.062928	0.148743	-0.775285
22	1	0	-1.880257	-0.042993	-1.839894
23	8	0	0.699172	-1.427480	0.161315
24	1	0	-0.118600	-1.441263	0.672009
25	6	0	3.575216	-0.792826	0.268402
26	1	0	3.185972	-1.728790	0.675014
27	6	0	4.847027	-1.074581	-0.517482
28	1	0	4.656271	-1.821730	-1.290946
29	1	0	5.188496	-0.150604	-1.007766
30	8	0	3.890765	0.036548	1.381470
31	1	0	3.945048	0.936613	1.031727
32	8	0	5.851841	-1.584637	0.336898
33	1	0	5.832175	-1.017701	1.117267
34	8	0	0.577285	2.770012	0.739445
35	1	0	3.395146	1.258951	-1.653835
36	1	0	0.710910	2.891859	-1.263275

Structure 101α (B3LYP, Gas Phase)

Energy (Hartrees): =-1547.1361896
No imaginary frequencies

Standard orientation:

Center Number	Atomic Number	Atomic Type	Coordinates (Angstroms) X	Y	Z
1	6	0	0.003098	1.827165	-0.373004
2	6	0	-0.473718	0.359370	-0.334502
3	6	0	0.197606	-0.339661	0.853136
4	6	0	1.727848	-0.196085	0.782195
5	6	0	2.090206	1.287140	0.651532
6	1	0	-0.381730	2.347430	-1.249625
7	1	0	-0.155881	0.098619	1.788991
8	1	0	2.170255	-0.609451	1.692105
9	1	0	1.783146	1.749795	1.601904
10	1	0	-0.114175	-0.113968	-1.262114
11	8	0	1.396864	1.921871	-0.428009
12	6	0	3.572960	1.619239	0.487633
13	1	0	3.714360	2.681974	0.698182
14	1	0	4.176946	1.021448	1.174246
15	8	0	4.033580	1.435406	-0.859805
16	8	0	2.238566	-0.930078	-0.350559
17	8	0	-0.169237	-1.725665	0.818919
18	8	0	-0.480011	2.493146	0.807660
19	7	0	-1.914595	0.310440	-0.204971
20	6	0	-4.022063	-0.562304	-1.002546
21	6	0	-4.803049	0.077706	-0.025629
22	6	0	-4.651468	-1.363377	-1.966128
23	6	0	-6.183852	-0.084120	-0.018937
24	1	0	-4.304100	0.693983	0.714380
25	6	0	-6.036381	-1.524579	-1.958282
26	1	0	-4.049986	-1.860137	-2.723625
27	6	0	-6.804195	-0.885065	-0.984544
28	1	0	-6.782831	0.412810	0.738565
29	1	0	-6.514607	-2.146504	-2.709022
30	6	0	-2.559330	-0.411451	-1.036281
31	1	0	-2.046346	-0.970214	-1.835863
32	6	0	4.909824	0.437468	-1.110517
33	6	0	2.697976	-2.191012	-0.108411
34	6	0	-1.238532	3.617114	0.643429
35	6	0	-0.251839	-2.359740	2.026084
36	8	0	-1.514874	4.096878	-0.429994
37	8	0	5.332600	-0.332957	-0.274485
38	8	0	2.675401	-2.722981	0.978433
39	8	0	-0.117436	-1.795348	3.087038
40	6	0	-1.676823	4.147047	1.984967
41	6	0	-0.494619	-3.832487	1.834253
42	6	0	3.268375	-2.807513	-1.359340
43	6	0	5.270426	0.418699	-2.575782
44	1	0	-2.240259	5.068744	1.845259

45	1	0	-2.298862	3.400402	2.487387
46	1	0	-0.807011	4.329541	2.622224
47	1	0	0.458898	-4.296066	1.561108
48	1	0	-0.851911	-4.267340	2.767352
49	1	0	-1.206439	-4.012957	1.025874
50	1	0	3.204741	-3.893542	-1.286762
51	1	0	2.753709	-2.450318	-2.253152
52	1	0	4.323052	-2.521814	-1.422364
53	1	0	6.088087	-0.281892	-2.742371
54	1	0	4.396207	0.112334	-3.158681
55	1	0	5.551785	1.419273	-2.913047
56	1	0	-7.883205	-1.008726	-0.975702

Structure 101α (B3LYP, $CHCl_3$)

Energy (Hartrees): = -1547.1673388
No imaginary frequencies

Standard orientation:

Center	Atomic	Atomic	Coordinates (Angstroms)		
Number	Number	Type	X	Y	Z
1	6	0	-0.038725	1.789296	-0.423610
2	6	0	-0.533867	0.330817	-0.328182
3	6	0	0.165179	-0.360271	0.848981
4	6	0	1.693261	-0.225431	0.738018
5	6	0	2.054337	1.254955	0.594534
6	1	0	-0.424531	2.274938	-1.319460
7	1	0	-0.163511	0.080256	1.792684
8	1	0	2.167180	-0.637063	1.631231
9	1	0	1.741496	1.730087	1.535770
10	1	0	-0.211704	-0.161907	-1.258216
11	8	0	1.356853	1.865439	-0.502700
12	6	0	3.531064	1.597642	0.444119
13	1	0	3.647648	2.677740	0.559444
14	1	0	4.113834	1.084267	1.211492
15	8	0	4.053165	1.294597	-0.862437
16	8	0	2.151965	-0.961744	-0.416568
17	8	0	-0.213914	-1.748003	0.820380
18	8	0	-0.509171	2.501905	0.735127
19	7	0	-1.972873	0.297511	-0.154082
20	6	0	-4.116323	-0.500350	-0.938230
21	6	0	-4.867547	0.106235	0.083830
22	6	0	-4.778320	-1.251649	-1.921357
23	6	0	-6.250534	-0.039059	0.115619
24	1	0	-4.350418	0.684801	0.842249
25	6	0	-6.165230	-1.396287	-1.887732
26	1	0	-4.200536	-1.722585	-2.712793
27	6	0	-6.902955	-0.790293	-0.869404
28	1	0	-6.825413	0.431874	0.907824
29	1	0	-6.668214	-1.979547	-2.653405
30	6	0	-2.652040	-0.371348	-1.004569
31	1	0	-2.170982	-0.895708	-1.844135
32	6	0	4.939665	0.280865	-0.980315
33	6	0	2.618536	-2.222491	-0.209106
34	6	0	-0.899249	3.798434	0.566837
35	6	0	-0.256287	-2.403207	2.012253
36	8	0	-0.895243	4.366491	-0.502970
37	8	0	5.289805	-0.429757	-0.059223
38	8	0	2.708120	-2.740159	0.884123
39	8	0	-0.042693	-1.865663	3.078137
40	6	0	-1.332201	4.392642	1.878040
41	6	0	-0.577171	-3.857369	1.810732
42	6	0	2.976626	-2.877620	-1.514944
43	6	0	5.442629	0.183886	-2.396712
44	1	0	-1.657304	5.421705	1.726400
45	1	0	-2.148917	3.800646	2.302244
46	1	0	-0.503381	4.366538	2.592684
47	1	0	0.318108	-4.362448	1.432466
48	1	0	-0.864008	-4.305395	2.762259
49	1	0	-1.373712	-3.984768	1.073726
50	1	0	3.472076	-3.829263	-1.322957
51	1	0	2.063512	-3.051476	-2.094271
52	1	0	3.626431	-2.230445	-2.108628
53	1	0	6.044836	-0.716901	-2.515242
54	1	0	4.607365	0.180631	-3.102266
55	1	0	6.057183	1.061729	-2.623715
56	1	0	-7.983096	-0.901525	-0.840589

Structure 101α (M06-2X/6-311G(d,p), Gas Phase)

Energy (Hartrees): = -1546.9188599
No imaginary frequencies

Standard orientation:

Center Number	Atomic Number	Atomic Type	Coordinates (Angstroms) X	Y	Z
1	6	0	0.026515	1.902323	-0.458660
2	6	0	-0.401152	0.426271	-0.442925
3	6	0	0.245931	-0.253685	0.758948
4	6	0	1.762136	-0.052748	0.727377
5	6	0	2.051922	1.442660	0.669189
6	1	0	-0.344859	2.411533	-1.345176
7	1	0	-0.152829	0.168686	1.683554
8	1	0	2.212998	-0.493224	1.619313
9	1	0	1.660426	1.866306	1.604099
10	1	0	-0.025943	-0.051376	-1.357252
11	8	0	1.404152	2.056903	-0.436821
12	6	0	3.515681	1.840872	0.601336
13	1	0	3.583881	2.918956	0.742327
14	1	0	4.084670	1.321322	1.373728
15	8	0	4.082930	1.593011	-0.682198
16	8	0	2.297470	-0.692040	-0.438816
17	8	0	-0.076656	-1.637060	0.687437
18	8	0	-0.523985	2.521642	0.706289
19	7	0	-1.840531	0.375315	-0.327379
20	6	0	-3.922970	-0.669579	-0.883782
21	6	0	-4.705934	0.393260	-0.424809
22	6	0	-4.535972	-1.845898	-1.312089
23	6	0	-6.085706	0.267531	-0.385876
24	1	0	-4.211996	1.312121	-0.129665
25	6	0	-5.919567	-1.973276	-1.263457
26	1	0	-3.925317	-2.664956	-1.678381
27	6	0	-6.694100	-0.916986	-0.799122
28	1	0	-6.693695	1.095154	-0.040635
29	1	0	-6.391574	-2.891713	-1.590875
30	6	0	-2.453972	-0.552772	-0.930114
31	1	0	-1.922441	-1.314560	-1.516488
32	6	0	4.848853	0.503114	-0.835899
33	6	0	2.727046	-1.966565	-0.286211
34	6	0	-1.699065	3.185848	0.566908
35	6	0	0.028595	-2.325124	1.855303
36	8	0	-2.185407	3.467118	-0.488542
37	8	0	5.117938	-0.264854	0.047599
38	8	0	2.628597	-2.584400	0.735546
39	8	0	0.279477	-1.796032	2.898812
40	6	0	-2.290520	3.484200	1.913441
41	6	0	-0.187487	-3.791223	1.634450
42	6	0	3.359213	-2.486541	-1.545190
43	6	0	5.295700	0.383247	-2.265653
44	1	0	-3.091687	4.210569	1.805221
45	1	0	-2.689240	2.548520	2.312936
46	1	0	-1.525051	3.845632	2.599059
47	1	0	0.746988	-4.196395	1.240298
48	1	0	-0.419180	-4.267792	2.583491
49	1	0	-0.979735	-3.959361	0.906213
50	1	0	4.430109	-2.283105	-1.477087
51	1	0	3.211818	-3.563019	-1.597465
52	1	0	2.952742	-1.995040	-2.427141
53	1	0	6.036220	-0.407527	-2.356097
54	1	0	4.421453	0.155461	-2.879045
55	1	0	5.702060	1.333780	-2.610258
56	1	0	-7.773119	-1.011071	-0.766464

Structure 101α (M06-2X/6-311G(d,p), $CHCl_3$)

Energy (Hartrees): = -1546.9522251
No imaginary frequencies

Standard orientation:

Center Number	Atomic Number	Atomic Type	Coordinates (Angstroms) X	Y	Z
1	6	0	-0.000201	1.881268	-0.511763
2	6	0	-0.431599	0.409648	-0.429385
3	6	0	0.243151	-0.238581	0.775218
4	6	0	1.755840	-0.030352	0.717640
5	6	0	2.042446	1.461139	0.609612
6	1	0	-0.378070	2.346464	-1.419449
7	1	0	-0.146657	0.192852	1.699280
8	1	0	2.228517	-0.439361	1.612325
9	1	0	1.660605	1.918824	1.531199
10	1	0	-0.084146	-0.093260	-1.341089
11	8	0	1.379572	2.030862	-0.517032
12	6	0	3.503297	1.857023	0.523421
13	1	0	3.569611	2.942406	0.590780
14	1	0	4.059008	1.400423	1.343087

15	8	0	4.098973	1.520815	-0.731622
16	8	0	2.269749	-0.696999	-0.441896
17	8	0	-0.067701	-1.629361	0.724264
18	8	0	-0.525946	2.566220	0.628861
19	7	0	-1.867443	0.348586	-0.266109
20	6	0	-3.965126	-0.654342	-0.876350
21	6	0	-4.745904	0.220929	-0.114696
22	6	0	-4.576080	-1.704056	-1.563438
23	6	0	-6.118310	0.038111	-0.039158
24	1	0	-4.261980	1.041944	0.401710
25	6	0	-5.952210	-1.889513	-1.482067
26	1	0	-3.967967	-2.377026	-2.159726
27	6	0	-6.723255	-1.019028	-0.719657
28	1	0	-6.722881	0.719049	0.548687
29	1	0	-6.422049	-2.709101	-2.013098
30	6	0	-2.502206	-0.491551	-0.970442
31	1	0	-1.992528	-1.148193	-1.687328
32	6	0	4.872546	0.427383	-0.796528
33	6	0	2.746695	-1.947225	-0.270360
34	6	0	-1.673394	3.267466	0.476373
35	6	0	0.042049	-2.313427	1.886879
36	8	0	-2.194279	3.465043	-0.585911
37	8	0	5.066675	-0.313311	0.132437
38	8	0	2.720633	-2.532451	0.778820
39	8	0	0.300515	-1.782512	2.932084
40	6	0	-2.180306	3.738598	1.804415
41	6	0	-0.183925	-3.776818	1.673415
42	6	0	3.280812	-2.515847	-1.550426
43	6	0	5.470251	0.281886	-2.164349
44	1	0	-3.021175	4.411952	1.657780
45	1	0	-2.496010	2.866427	2.381983
46	1	0	-1.381485	4.236206	2.355773
47	1	0	0.722326	-4.188136	1.222064
48	1	0	-0.363754	-4.261563	2.630218
49	1	0	-1.017480	-3.941938	0.991121
50	1	0	4.232301	-3.006023	-1.345217
51	1	0	2.577631	-3.273447	-1.905089
52	1	0	3.397583	-1.752351	-2.316462
53	1	0	5.949361	-0.690191	-2.257031
54	1	0	4.700747	0.411014	-2.926136
55	1	0	6.213888	1.069868	-2.305890
56	1	0	-7.796250	-1.159666	-0.656894

Structure 101α-a (B3LYP, Gas Phase)

Energy (Hartrees): = -1547.1389641
No imaginary frequencies

Standard orientation:

Center Number	Atomic Number	Atomic Type	Coordinates (Angstroms) X	Y	Z
1	6	0	-0.472181	-1.319442	-0.386687
2	6	0	0.462945	-0.089587	-0.390789
3	6	0	-0.007481	0.890497	0.690032
4	6	0	-1.495199	1.227556	0.516572
5	6	0	-2.318005	-0.061724	0.452302
6	1	0	-0.232690	-2.001836	-1.201749
7	1	0	0.143510	0.454886	1.680127
8	1	0	-1.825521	1.860566	1.343792
9	1	0	-2.256313	-0.547211	1.435188
10	1	0	0.336837	0.393944	-1.372596
11	8	0	-1.812076	-0.952987	-0.549766
12	6	0	-3.778390	0.194532	0.116496
13	1	0	-4.188749	0.952310	0.792232
14	1	0	-3.888441	0.523205	-0.916654
15	8	0	-4.515495	-1.021514	0.319625
16	8	0	-1.688235	1.943134	-0.726167
17	8	0	0.778994	2.087228	0.578334
18	8	0	-0.302553	-2.011745	0.862034
19	7	0	1.828728	-0.500060	-0.142577
20	6	0	4.140320	-0.484725	-0.846544
21	6	0	4.613083	-1.218314	0.254559
22	6	0	5.046178	-0.068441	-1.832639
23	6	0	5.964621	-1.526614	0.359932
24	1	0	3.902232	-1.532848	1.010950
25	6	0	6.401319	-0.378573	-1.725649
26	1	0	4.683531	0.499523	-2.686005
27	6	0	6.861967	-1.108254	-0.629228
28	1	0	6.324961	-2.094110	1.212938
29	1	0	7.095008	-0.052764	-2.494870
30	6	0	2.716383	-0.144192	-0.987256
31	1	0	2.459416	0.446573	-1.881719
32	6	0	-5.233548	-1.500591	-0.732902
33	6	0	-1.753303	3.300336	-0.674161

34	6	0	-0.054808	-3.354388	0.819305
35	6	0	0.960419	2.798693	1.729970
36	8	0	0.031536	-3.994593	-0.201211
37	8	0	-5.362547	-0.924291	-1.788079
38	8	0	-1.694237	3.945836	0.348760
39	8	0	0.565228	2.428723	2.811369
40	6	0	0.102517	-3.897148	2.216812
41	6	0	1.673806	4.093517	1.446849
42	6	0	-1.910962	3.869444	-2.061782
43	6	0	-5.830865	-2.842028	-0.387273
44	1	0	0.271549	-4.972299	2.172151
45	1	0	0.946263	-3.404920	2.709198
46	1	0	-0.791956	-3.680642	2.807791
47	1	0	0.944481	4.796722	1.031367
48	1	0	2.073189	4.499184	2.376009
49	1	0	2.469806	3.953269	0.712439
50	1	0	-2.070297	4.945066	-1.996723
51	1	0	-1.008189	3.664502	-2.645413
52	1	0	-2.748006	3.391440	-2.577330
53	1	0	-6.531240	-3.138810	-1.167073
54	1	0	-5.031151	-3.585488	-0.311539
55	1	0	-6.334098	-2.799864	0.582023
56	1	0	7.916953	-1.351622	-0.543206

Structure 101α-a (B3LYP, $CHCl_3$)

Energy (Hartrees): = -1547.169996
No imaginary frequencies

Standard orientation:

Center Number	Atomic Number	Atomic Type	Coordinates (Angstroms) X	Y	Z
1	6	0	-0.448539	-1.274372	-0.355551
2	6	0	0.497722	-0.054705	-0.356088
3	6	0	0.021474	0.951558	0.697905
4	6	0	-1.460690	1.299635	0.502664
5	6	0	-2.292841	0.016899	0.449475
6	1	0	-0.209056	-1.953255	-1.173737
7	1	0	0.159004	0.540475	1.700136
8	1	0	-1.802005	1.941814	1.317070
9	1	0	-2.244714	-0.451255	1.440856
10	1	0	0.393620	0.406777	-1.349754
11	8	0	-1.784095	-0.895764	-0.536665
12	6	0	-3.748243	0.276598	0.097439
13	1	0	-4.164097	1.036781	0.765022
14	1	0	-3.850810	0.597870	-0.938666
15	8	0	-4.495257	-0.936135	0.309180
16	8	0	-1.626099	1.999755	-0.752389
17	8	0	0.827764	2.136333	0.561132
18	8	0	-0.285358	-1.966687	0.894699
19	7	0	1.859027	-0.469354	-0.077789
20	6	0	4.150600	-0.626333	-0.833295
21	6	0	4.644837	-1.197944	0.352470
22	6	0	5.031731	-0.394550	-1.900812
23	6	0	5.991259	-1.529943	0.460763
24	1	0	3.960384	-1.372673	1.176075
25	6	0	6.381085	-0.730110	-1.790941
26	1	0	4.653387	0.048786	-2.818631
27	6	0	6.862779	-1.297642	-0.610053
28	1	0	6.367320	-1.970036	1.379863
29	1	0	7.054388	-0.548246	-2.623377
30	6	0	2.733639	-0.260078	-0.985264
31	1	0	2.470015	0.212188	-1.943740
32	6	0	-5.106923	-1.503647	-0.761073
33	6	0	-1.720707	3.355106	-0.718665
34	6	0	-0.419177	-3.324738	0.887047
35	6	0	1.059046	2.861302	1.688984
36	8	0	-0.640995	-3.970423	-0.113334
37	8	0	-5.144154	-1.005551	-1.866419
38	8	0	-1.723768	4.007085	0.304796
39	8	0	0.644508	2.543610	2.783634
40	6	0	-0.243150	-3.877783	2.273433
41	6	0	1.866330	4.090631	1.378356
42	6	0	-1.815434	3.912010	-2.112419
43	6	0	-5.717900	-2.824252	-0.376814
44	1	0	-0.321924	-4.964460	2.249514
45	1	0	0.732164	-3.581224	2.670783
46	1	0	-1.007607	-3.464748	2.939204
47	1	0	1.220764	4.819452	0.876417
48	1	0	2.242342	4.525306	2.304615
49	1	0	2.693847	3.854365	0.704827
50	1	0	-2.029587	4.979764	-2.067407
51	1	0	-0.865047	3.751626	-2.632771
52	1	0	-2.592779	3.394045	-2.680710

53	1	0	-6.357698	-3.182537	-1.183228
54	1	0	-4.918459	-3.551290	-0.197811
55	1	0	-6.291734	-2.728641	0.548937
56	1	0	7.913363	-1.559046	-0.521409

Structure 101α-a (M06-2X/6-311G(d,p), Gas Phase)

Energy (Hartrees): = -1546.9170187
No imaginary frequencies

Standard orientation:

Center Number	Atomic Number	Atomic Type	Coordinates (Angstroms) X	Y	Z
1	6	0	-0.671593	-1.266098	-0.402180
2	6	0	0.411144	-0.183472	-0.414287
3	6	0	0.115605	0.817981	0.699983
4	6	0	-1.307493	1.363348	0.565636
5	6	0	-2.285522	0.201148	0.511716
6	1	0	-0.557939	-1.961735	-1.231845
7	1	0	0.226699	0.335550	1.673240
8	1	0	-1.527570	2.029120	1.403075
9	1	0	-2.249222	-0.315671	1.478577
10	1	0	0.323439	0.342414	-1.374940
11	8	0	-1.939719	-0.707932	-0.525778
12	6	0	-3.718829	0.609458	0.233613
13	1	0	-4.085875	1.277991	1.013379
14	1	0	-3.799726	1.079939	-0.745512
15	8	0	-4.500813	-0.579154	0.274793
16	8	0	-1.425640	2.085383	-0.666981
17	8	0	1.056787	1.878887	0.576809
18	8	0	-0.556069	-1.974278	0.828828
19	7	0	1.714847	-0.774084	-0.219737
20	6	0	4.053429	-0.790126	-0.779281
21	6	0	4.377336	-1.813234	0.115674
22	6	0	5.054223	-0.216311	-1.561902
23	6	0	5.687463	-2.252167	0.220009
24	1	0	3.585382	-2.245064	0.715434
25	6	0	6.368815	-0.656064	-1.454901
26	1	0	4.800783	0.578580	-2.256000
27	6	0	6.685347	-1.674510	-0.564373
28	1	0	5.937493	-3.046745	0.912755
29	1	0	7.142590	-0.206130	-2.064947
30	6	0	2.667426	-0.304956	-0.909033
31	1	0	2.509206	0.496936	-1.644056
32	6	0	-4.805560	-1.147197	-0.923842
33	6	0	-1.202533	3.415867	-0.644327
34	6	0	-1.168668	-3.182768	0.884796
35	6	0	1.233774	2.638229	1.690996
36	8	0	-1.752618	-3.669177	-0.039529
37	8	0	-4.740657	-0.567815	-1.967043
38	8	0	-0.937616	4.032576	0.349784
39	8	0	0.707184	2.387594	2.735818
40	6	0	-1.006447	-3.792703	2.247415
41	6	0	2.131398	3.803766	1.407444
42	6	0	-1.331641	3.994353	-2.024249
43	6	0	-5.203687	-2.580309	-0.732584
44	1	0	-1.394181	-4.807585	2.235731
45	1	0	0.045529	-3.781176	2.532550
46	1	0	-1.555449	-3.192678	2.975459
47	1	0	1.532522	4.553800	0.886297
48	1	0	2.492786	4.214865	2.346531
49	1	0	2.959421	3.505814	0.765761
50	1	0	-1.325423	5.079097	-1.961180
51	1	0	-0.488214	3.653532	-2.627812
52	1	0	-2.245353	3.636110	-2.497528
53	1	0	-5.711128	-2.939048	-1.624224
54	1	0	-4.285655	-3.153464	-0.574684
55	1	0	-5.833127	-2.689083	0.149667
56	1	0	7.708460	-2.020871	-0.479117

Structure 101α-a (M06-2X/6-311G(d,p), $CHCl_3$)

Energy (Hartrees): = -1546.9506847
No imaginary frequencies

Standard orientation:

Center Number	Atomic Number	Atomic Type	Coordinates (Angstroms) X	Y	Z
1	6	0	-0.578917	-1.285585	-0.358390
2	6	0	0.450636	-0.152942	-0.370773
3	6	0	0.088472	0.853281	0.717782

4	6	0	-1.355734	1.326787	0.551745
5	6	0	-2.286724	0.125790	0.476923
6	1	0	-0.403718	-1.989344	-1.170037
7	1	0	0.210629	0.398964	1.702942
8	1	0	-1.635794	1.974842	1.383584
9	1	0	-2.276953	-0.371096	1.454000
10	1	0	0.361651	0.342869	-1.346474
11	8	0	-1.870887	-0.791961	-0.530235
12	6	0	-3.710444	0.513254	0.137906
13	1	0	-4.093926	1.220711	0.874060
14	1	0	-3.765979	0.940749	-0.861821
15	8	0	-4.517722	-0.662644	0.217390
16	8	0	-1.471781	2.047500	-0.682873
17	8	0	0.982021	1.956516	0.579165
18	8	0	-0.463023	-1.958097	0.892110
19	7	0	1.777627	-0.676577	-0.133616
20	6	0	4.087484	-0.768174	-0.804947
21	6	0	4.492652	-1.574719	0.263388
22	6	0	5.024071	-0.362499	-1.756173
23	6	0	5.817951	-1.967378	0.372349
24	1	0	3.756692	-1.883358	0.996391
25	6	0	6.353341	-0.757241	-1.645575
26	1	0	4.708510	0.263998	-2.584501
27	6	0	6.750646	-1.559405	-0.581546
28	1	0	6.129248	-2.591999	1.201612
29	1	0	7.076196	-0.437768	-2.387027
30	6	0	2.685874	-0.331304	-0.947360
31	1	0	2.473889	0.317559	-1.807514
32	6	0	-4.871910	-1.259329	-0.941918
33	6	0	-1.333434	3.389209	-0.648013
34	6	0	-0.963821	-3.216254	0.959081
35	6	0	1.137732	2.746197	1.668108
36	8	0	-1.452229	-3.776794	0.018288
37	8	0	-4.706602	-0.768225	-2.024198
38	8	0	-1.158205	4.011726	0.365329
39	8	0	0.613740	2.511246	2.722178
40	6	0	-0.818019	-3.771202	2.341924
41	6	0	2.016150	3.917290	1.360026
42	6	0	-1.422919	3.969058	-2.025739
43	6	0	-5.487427	-2.599391	-0.676413
44	1	0	-1.162785	-4.802007	2.359825
45	1	0	0.225896	-3.711404	2.653018
46	1	0	-1.408863	-3.166781	3.033450
47	1	0	1.444303	4.610651	0.738692
48	1	0	2.304241	4.410321	2.285330
49	1	0	2.896161	3.598083	0.801493
50	1	0	-1.477905	5.053053	-1.961666
51	1	0	-0.531500	3.677433	-2.586375
52	1	0	-2.292626	3.568742	-2.547422
53	1	0	-5.952979	-2.974806	-1.584473
54	1	0	-4.694317	-3.282068	-0.362415
55	1	0	-6.215905	-2.530217	0.131734
56	1	0	7.785971	-1.867890	-0.492634

Structure 101α-b (B3LYP, Gas Phase)

Energy (Hartrees): =-1547.1404217
No imaginary frequencies

Standard orientation:

Center Number	Atomic Number	Atomic Type	Coordinates (Angstroms) X	Y	Z
1	6	0	-0.031822	1.559246	-1.061046
2	6	0	-0.635481	0.182016	-0.706147
3	6	0	0.248800	-0.487813	0.352640
4	6	0	1.711080	-0.542141	-0.109618
5	6	0	2.178841	0.861482	-0.498682
6	1	0	-0.573532	2.030122	-1.881100
7	1	0	0.195013	0.068840	1.290723
8	1	0	2.337152	-0.951449	0.685069
9	1	0	2.192912	1.477464	0.408252
10	1	0	-0.590287	-0.424586	-1.624756
11	8	0	1.303080	1.450126	-1.470284
12	6	0	3.562547	0.900925	-1.144017
13	1	0	3.561428	0.323439	-2.068483
14	1	0	3.835009	1.938162	-1.345310
15	8	0	4.546458	0.281772	-0.295913
16	8	0	1.820812	-1.392983	-1.274980
17	8	0	-0.249515	-1.818318	0.565351
18	8	0	-0.115454	2.404331	0.096375
19	7	0	-1.985922	0.342583	-0.210583
20	6	0	-4.315270	-0.284047	-0.347536
21	6	0	-4.727901	0.529550	0.721000
22	6	0	-5.266927	-1.059049	-1.025731

23	6	0	-6.065795	0.562712	1.097466
24	1	0	-3.981842	1.123431	1.237825
25	6	0	-6.608369	-1.024672	-0.647054
26	1	0	-4.951086	-1.689533	-1.853457
27	6	0	-7.009369	-0.213656	0.415138
28	1	0	-6.379294	1.193402	1.924189
29	1	0	-7.337956	-1.627943	-1.178924
30	6	0	-2.907078	-0.341471	-0.768471
31	1	0	-2.693543	-1.023965	-1.607369
32	6	0	5.087579	1.062845	0.678922
33	6	0	2.200853	-2.683224	-1.082878
34	6	0	-0.699926	3.631406	-0.048166
35	6	0	-0.029693	-2.354791	1.801311
36	8	0	-1.162980	4.041049	-1.085662
37	8	0	4.771100	2.216372	0.864335
38	8	0	2.464410	-3.165056	-0.003803
39	8	0	0.476366	-1.743613	2.713985
40	6	0	-0.679974	4.371616	1.264265
41	6	0	-0.468437	-3.794272	1.839060
42	6	0	2.242748	-3.417920	-2.400303
43	6	0	6.110457	0.288382	1.471155
44	1	0	-1.156574	5.343242	1.140620
45	1	0	-1.204626	3.788102	2.026132
46	1	0	0.351835	4.500575	1.603970
47	1	0	0.299919	-4.393495	1.339636
48	1	0	-0.561036	-4.119598	2.874915
49	1	0	-1.410728	-3.933344	1.304717
50	1	0	2.628019	-4.424422	-2.241843
51	1	0	1.235318	-3.469964	-2.824297
52	1	0	2.870903	-2.881322	-3.116313
53	1	0	6.558803	0.941109	2.219007
54	1	0	5.632255	-0.565459	1.960371
55	1	0	6.881574	-0.108254	0.804913
56	1	0	-8.053570	-0.184793	0.712525

Structure 101α-b (B3LYP, $CHCl_3$)

Energy (Hartrees): = -1547.1703758
No imaginary frequencies

Standard orientation:

Center Number	Atomic Number	Atomic Type	Coordinates (Angstroms) X	Y	Z
1	6	0	-0.076783	1.565838	-0.999791
2	6	0	-0.672553	0.182962	-0.660709
3	6	0	0.219862	-0.507218	0.377890
4	6	0	1.680852	-0.546935	-0.089968
5	6	0	2.144732	0.861804	-0.463643
6	1	0	-0.620899	2.035721	-1.818753
7	1	0	0.167487	0.021256	1.332113
8	1	0	2.313584	-0.956918	0.698280
9	1	0	2.170536	1.460332	0.454019
10	1	0	-0.633614	-0.402248	-1.592082
11	8	0	1.257529	1.469618	-1.416892
12	6	0	3.517743	0.907332	-1.132724
13	1	0	3.492890	0.367616	-2.079815
14	1	0	3.805794	1.945907	-1.304627
15	8	0	4.510556	0.238820	-0.332395
16	8	0	1.786688	-1.387407	-1.262139
17	8	0	-0.278999	-1.846691	0.551147
18	8	0	-0.173995	2.396355	0.168869
19	7	0	-2.023239	0.327884	-0.153782
20	6	0	-4.365728	-0.231022	-0.362573
21	6	0	-4.779031	0.504239	0.762211
22	6	0	-5.321639	-0.936314	-1.109603
23	6	0	-6.121292	0.529951	1.125932
24	1	0	-4.035084	1.046243	1.336852
25	6	0	-6.667127	-0.909769	-0.742968
26	1	0	-5.004105	-1.505617	-1.979747
27	6	0	-7.068597	-0.176389	0.374924
28	1	0	-6.434498	1.100097	1.995768
29	1	0	-7.399540	-1.458712	-1.327525
30	6	0	-2.955225	-0.284247	-0.777310
31	1	0	-2.750878	-0.893936	-1.670481
32	6	0	5.085175	0.956860	0.667340
33	6	0	2.221708	-2.662723	-1.090806
34	6	0	-0.472403	3.716257	-0.011848
35	6	0	-0.083350	-2.428657	1.764841
36	8	0	-0.682469	4.212755	-1.096346
37	8	0	4.783667	2.104448	0.922365
38	8	0	2.538670	-3.131803	-0.017510
39	8	0	0.441489	-1.863236	2.700540
40	6	0	-0.511196	4.431041	1.309544
41	6	0	-0.580937	-3.846925	1.761610

42	6	0	2.248922	-3.391702	-2.406591
43	6	0	6.131301	0.141539	1.376580
44	1	0	-0.747551	5.483317	1.152694
45	1	0	-1.264023	3.970961	1.957103
46	1	0	0.455926	4.337144	1.813135
47	1	0	0.134817	-4.465839	1.209709
48	1	0	-0.654060	-4.213352	2.785677
49	1	0	-1.548191	-3.921397	1.258890
50	1	0	2.666452	-4.388608	-2.266300
51	1	0	1.231731	-3.471246	-2.803586
52	1	0	2.843776	-2.835801	-3.137046
53	1	0	6.503567	0.693035	2.239768
54	1	0	5.713961	-0.818402	1.693577
55	1	0	6.958719	-0.069621	0.691117
56	1	0	-8.115825	-0.153504	0.662596

Structure 101α-b (M06-2X/6-311G(d,p), Gas Phase)

Energy (Hartrees): = -1546.9213962
No imaginary frequencies

Standard orientation:

Center Number	Atomic Number	Atomic Type	Coordinates (Angstroms) X	Y	Z
1	6	0	-0.198080	1.799992	-1.031704
2	6	0	-0.594537	0.334456	-0.783928
3	6	0	0.307684	-0.229702	0.305381
4	6	0	1.773113	-0.019558	-0.049998
5	6	0	2.051610	1.439991	-0.370846
6	1	0	-0.773437	2.227622	-1.850002
7	1	0	0.092721	0.257301	1.258467
8	1	0	2.390378	-0.361252	0.781278
9	1	0	1.957662	2.021708	0.549308
10	1	0	-0.419294	-0.228642	-1.710441
11	8	0	1.144582	1.933135	-1.355092
12	6	0	3.436738	1.620553	-0.998786
13	1	0	3.349008	1.435423	-2.066694
14	1	0	3.808968	2.629026	-0.816816
15	8	0	4.382816	0.657718	-0.527373
16	8	0	2.080123	-0.782576	-1.224294
17	8	0	0.048795	-1.628670	0.404723
18	8	0	-0.462041	2.532349	0.164192
19	7	0	-1.971931	0.298972	-0.351279
20	6	0	-4.130002	-0.744774	-0.419593
21	6	0	-4.789021	0.317753	0.205095
22	6	0	-4.826930	-1.917037	-0.708118
23	6	0	-6.126785	0.195345	0.546080
24	1	0	-4.238808	1.232722	0.391491
25	6	0	-6.166561	-2.040855	-0.357897
26	1	0	-4.315364	-2.735795	-1.204222
27	6	0	-6.816006	-0.984814	0.270431
28	1	0	-6.639770	1.021813	1.023329
29	1	0	-6.702717	-2.955861	-0.578489
30	6	0	-2.706759	-0.634452	-0.787092
31	1	0	-2.318642	-1.408193	-1.463902
32	6	0	4.748914	0.769626	0.768670
33	6	0	2.757354	-1.931025	-1.054641
34	6	0	-1.632661	3.214734	0.236859
35	6	0	0.434472	-2.217416	1.566863
36	8	0	-2.366357	3.380258	-0.692678
37	8	0	4.342751	1.644087	1.481534
38	8	0	3.020146	-2.402186	0.019556
39	8	0	0.852772	-1.599871	2.502419
40	6	0	-1.864932	3.698021	1.638439
41	6	0	0.299852	-3.708148	1.483721
42	6	0	3.154688	-2.512057	-2.380201
43	6	0	5.661419	-0.352690	1.165080
44	1	0	-2.677251	4.419890	1.642840
45	1	0	-2.133535	2.833290	2.249751
46	1	0	-0.953490	4.130533	2.049477
47	1	0	1.196666	-4.081103	0.983079
48	1	0	0.250567	-4.121342	2.487965
49	1	0	-0.574086	-3.989497	0.898592
50	1	0	3.557240	-3.510806	-2.234717
51	1	0	2.298542	-2.530705	-3.053942
52	1	0	3.915849	-1.864821	-2.820790
53	1	0	6.060805	-0.161447	2.157205
54	1	0	5.079613	-1.278149	1.164879
55	1	0	6.463418	-0.460219	0.435074
56	1	0	-7.861667	-1.075892	0.539442

Structure 101α-b (M06-2X/6-311G(d,p), $CHCl_3$)

Energy (Hartrees): = -1546.9527966
No imaginary frequencies

Standard orientation:

Center Number	Atomic Number	Atomic Type	Coordinates (Angstroms) X	Y	Z
1	6	0	-0.139166	1.685279	-1.105616
2	6	0	-0.586102	0.253405	-0.775016
3	6	0	0.324852	-0.310720	0.308935
4	6	0	1.792091	-0.175515	-0.084351
5	6	0	2.105054	1.263955	-0.461603
6	1	0	-0.708283	2.085575	-1.941860
7	1	0	0.153481	0.215460	1.250200
8	1	0	2.423758	-0.500570	0.742787
9	1	0	2.031636	1.879587	0.438324
10	1	0	-0.471946	-0.350560	-1.684723
11	8	0	1.202459	1.746627	-1.456250
12	6	0	3.483473	1.401476	-1.101349
13	1	0	3.433437	1.033173	-2.123842
14	1	0	3.800699	2.444692	-1.094049
15	8	0	4.457034	0.581760	-0.449990
16	8	0	2.047873	-0.992577	-1.233424
17	8	0	-0.010734	-1.690786	0.455835
18	8	0	-0.347970	2.501134	0.047996
19	7	0	-1.947780	0.287506	-0.289698
20	6	0	-4.185046	-0.585389	-0.367697
21	6	0	-4.716982	0.374626	0.499234
22	6	0	-5.003373	-1.607453	-0.849468
23	6	0	-6.047902	0.303081	0.880883
24	1	0	-4.076216	1.172919	0.855073
25	6	0	-6.337402	-1.680606	-0.462021
26	1	0	-4.591451	-2.348028	-1.527504
27	6	0	-6.859928	-0.725692	0.403323
28	1	0	-6.458539	1.050044	1.550291
29	1	0	-6.967690	-2.478844	-0.835942
30	6	0	-2.772456	-0.534356	-0.788846
31	1	0	-2.476101	-1.252809	-1.564332
32	6	0	4.881010	0.991796	0.761966
33	6	0	2.583426	-2.211765	-1.026949
34	6	0	-1.463893	3.265822	0.092082
35	6	0	0.345386	-2.280122	1.621221
36	8	0	-2.220420	3.388397	-0.830560
37	8	0	4.476846	1.994983	1.286737
38	8	0	2.832867	-2.652159	0.063967
39	8	0	0.843788	-1.676799	2.531224
40	6	0	-1.608046	3.906409	1.437439
41	6	0	0.046447	-3.746306	1.591380
42	6	0	2.811568	-2.917625	-2.328220
43	6	0	5.882014	0.035653	1.333068
44	1	0	-2.416713	4.632859	1.412179
45	1	0	-1.832010	3.124941	2.167615
46	1	0	-0.671948	4.381932	1.731639
47	1	0	0.766914	-4.223272	0.922506
48	1	0	0.147592	-4.160579	2.591597
49	1	0	-0.955797	-3.921543	1.199616
50	1	0	3.283924	-3.879535	-2.145156
51	1	0	1.853437	-3.058912	-2.832321
52	1	0	3.441684	-2.301426	-2.971641
53	1	0	6.246760	0.411765	2.285623
54	1	0	5.406108	-0.937798	1.470877
55	1	0	6.707555	-0.093758	0.631755
56	1	0	-7.899913	-0.778197	0.704510

Structure 29.3DMSO (M06-2X/def2-TZVP, Gas Phase)

Energy (Hartrees): =-1515.9232295
No imaginary frequencies

Standard orientation:

Center Number	Atomic Number	Atomic Type	Coordinates (Angstroms) X	Y	Z
1	6	0	4.103761	-3.061731	0.969795
2	6	0	3.447980	-2.367029	-0.041688
3	6	0	3.845748	-2.545506	-1.366595
4	6	0	4.885364	-3.406888	-1.667036
5	6	0	5.538738	-4.098502	-0.650852
6	6	0	5.147628	-3.925505	0.668164
7	6	0	2.343445	-1.457681	0.307435
8	7	0	1.713093	-0.780601	-0.549078
9	6	0	0.662898	0.092249	-0.090763
10	6	0	-0.598129	-0.141362	-0.914153
11	6	0	-1.674878	0.883856	-0.559781
12	6	0	-1.100389	2.286667	-0.689943
13	8	0	0.079096	2.423634	0.085894
14	6	0	1.112311	1.549940	-0.265544
15	8	0	-1.043977	-1.457721	-0.669971
16	8	0	2.204226	1.863432	0.538708
17	6	0	-2.052819	3.371812	-0.225493
18	8	0	-1.568335	4.653898	-0.529519
19	8	0	-2.149077	0.711847	0.755833
20	1	0	2.096509	-1.401141	1.377147
21	1	0	0.422967	-0.050332	0.973714
22	1	0	-1.871484	-1.595321	-1.157995
23	1	0	-0.333872	-0.020654	-1.973348
24	1	0	-2.481397	-0.191420	0.868361
25	1	0	-2.494950	0.778816	-1.283816
26	1	0	-0.835944	4.867209	0.073429
27	1	0	-3.005719	3.246535	-0.745477
28	1	0	-2.229040	3.241760	0.845393
29	1	0	-0.854269	2.465884	-1.747579
30	1	0	1.879478	1.905328	1.447640
31	1	0	1.430478	1.711648	-1.304272
32	1	0	3.794618	-2.921298	1.999466
33	1	0	5.654188	-4.460989	1.460424
34	1	0	6.352168	-4.771142	-0.890494
35	1	0	5.192187	-3.543816	-2.695900
36	1	0	3.324501	-1.999106	-2.141470
37	8	0	-3.252563	-1.985435	1.512477
38	6	0	-2.268836	-2.695136	1.517672
39	6	0	-2.223927	-3.989224	0.755528
40	1	0	-1.449311	-3.906582	-0.009588
41	1	0	-1.944169	-4.809481	1.419311
42	1	0	-3.184395	-4.191171	0.288545
43	6	0	-1.036733	-2.328819	2.296569
44	1	0	-0.951000	-2.996045	3.158131
45	1	0	-0.156460	-2.469638	1.669093
46	1	0	-1.101777	-1.298147	2.638734
47	8	0	-3.740426	-1.640235	-1.524085
48	6	0	-4.751264	-1.609131	-0.856072
49	6	0	-5.593303	-2.839288	-0.655047
50	1	0	-5.231460	-3.652173	-1.279638
51	1	0	-5.530976	-3.119462	0.398809
52	1	0	-6.640221	-2.627898	-0.877858
53	6	0	-5.239063	-0.351918	-0.194196
54	1	0	-5.423956	-0.549545	0.862283
55	1	0	-4.512876	0.450867	-0.292391
56	1	0	-6.186577	-0.052995	-0.648660
57	8	0	0.776298	5.280147	0.998274
58	6	0	1.802225	5.047404	0.401420
59	6	0	1.827976	4.756165	-1.075168
60	1	0	2.450697	3.880889	-1.264719
61	1	0	0.819706	4.604690	-1.453579
62	1	0	2.289507	5.602931	-1.589904
63	6	0	3.134679	5.059391	1.102766
64	1	0	3.574886	4.062926	1.032092
65	1	0	3.814145	5.751303	0.601233
66	1	0	3.009757	5.346647	2.143435

Structure 29.3DMSO (M06-2X/def2-TZVP, DMSO)

Energy (Hartrees): = -=-1515.9625366
No imaginary frequencies

Standard orientation:

Center Number	Atomic Number	Atomic Type	Coordinates (Angstroms) X	Y	Z
1	6	0	-0.991993	-4.938387	0.927365
2	6	0	-0.520556	-4.078970	-0.062459
3	6	0	-0.363499	-4.554220	-1.365834
4	6	0	-0.675449	-5.868801	-1.668136
5	6	0	-1.147549	-6.722952	-0.674357
6	6	0	-1.305284	-6.256963	0.623435
7	6	0	-0.203412	-2.686078	0.301023
8	7	0	0.211823	-1.833360	-0.534112
9	6	0	0.490540	-0.499514	-0.055405
10	6	0	-0.325659	0.513723	-0.851508
11	6	0	0.071226	1.941247	-0.479027
12	6	0	1.578038	2.092358	-0.626768
13	8	0	2.261436	1.119036	0.149120
14	6	0	1.984312	-0.202659	-0.237010
15	8	0	-1.690058	0.272523	-0.590507
16	8	0	2.782289	-1.044946	0.525610
17	6	0	2.088858	3.447142	-0.185538
18	8	0	3.440687	3.644644	-0.532752
19	8	0	-0.296218	2.267664	0.843632
20	1	0	-0.353542	-2.427256	1.355866
21	1	0	0.254245	-0.384022	1.012479
22	1	0	-2.231064	0.945222	-1.041566
23	1	0	-0.103411	0.369017	-1.917180
24	1	0	-1.232652	2.052274	0.986090
25	1	0	-0.406826	2.625666	-1.193717
26	1	0	3.992405	3.015916	-0.034075
27	1	0	1.505864	4.222325	-0.687559
28	1	0	1.934426	3.550086	0.892733
29	1	0	1.830033	1.950201	-1.687655
30	1	0	2.498077	-0.973930	1.448269
31	1	0	2.271124	-0.361233	-1.284430
32	1	0	-1.112645	-4.567875	1.939257
33	1	0	-1.671868	-6.918294	1.398302
34	1	0	-1.390828	-7.750369	-0.914877
35	1	0	-0.552337	-6.233657	-2.680205
36	1	0	0.004066	-3.884878	-2.133324
37	8	0	-3.134127	1.926809	1.604351
38	6	0	-3.384672	0.737051	1.643062
39	6	0	-4.606863	0.168170	0.988566
40	1	0	-4.286553	-0.394474	0.107561
41	1	0	-5.102512	-0.536580	1.658612
42	1	0	-5.292858	0.956069	0.686846
43	6	0	-2.499578	-0.235882	2.361992
44	1	0	-3.000367	-0.527751	3.290141
45	1	0	-2.365617	-1.136525	1.762610
46	1	0	-1.537242	0.213924	2.598292
47	8	0	-3.543927	2.129998	-1.543615
48	6	0	-3.971418	3.121020	-0.982339
49	6	0	-5.443005	3.361324	-0.834442
50	1	0	-6.019215	2.598687	-1.353058
51	1	0	-5.692801	3.360693	0.229538
52	1	0	-5.692589	4.353434	-1.217649
53	6	0	-3.065657	4.189947	-0.452009
54	1	0	-3.464654	4.619806	0.465637
55	1	0	-2.061010	3.808603	-0.286329
56	1	0	-3.025515	4.987767	-1.200431
57	8	0	5.235399	1.909204	0.833032
58	6	0	5.452524	0.829907	0.318662
59	6	0	5.129193	0.554575	-1.120089
60	1	0	4.729443	-0.452305	-1.238856
61	1	0	4.436478	1.295418	-1.514185
62	1	0	6.065431	0.601516	-1.684988
63	6	0	6.105271	-0.283418	1.081615
64	1	0	5.399207	-1.112864	1.160050
65	1	0	6.972160	-0.649736	0.527281
66	1	0	6.404327	0.047447	2.073544

Structure 29.3DMSO (M06-2X/def2-TZVP, H_2O)

Energy (Hartrees): =-1515.9699676
No imaginary frequencies

Standard orientation:

Center Number	Atomic Number	Atomic Type	Coordinates (Angstroms) X	Y	Z
1	6	0	1.266913	4.822595	0.936511
2	6	0	0.723808	4.028053	-0.071297
3	6	0	0.576508	4.555111	-1.355761
4	6	0	0.967685	5.856384	-1.620966
5	6	0	1.510589	6.644992	-0.609364
6	6	0	1.660026	6.127239	0.669618
7	6	0	0.328056	2.651912	0.266294
8	7	0	-0.150667	1.829798	-0.574009
9	6	0	-0.484761	0.513441	-0.072033
10	6	0	0.254356	-0.555486	-0.869732
11	6	0	-0.191205	-1.957143	-0.457492
12	6	0	-1.706769	-2.041653	-0.539885
13	8	0	-2.299577	-1.024173	0.260380
14	6	0	-1.991166	0.277097	-0.192068
15	8	0	1.639036	-0.372475	-0.652808
16	8	0	-2.730097	1.179652	0.570293
17	6	0	-2.261208	-3.366111	-0.064128
18	8	0	-3.633516	-3.510660	-0.385070
19	8	0	0.211856	-2.273361	0.863284
20	1	0	0.476179	2.368270	1.313803
21	1	0	-0.225065	0.406032	0.990377
22	1	0	2.139408	-1.085666	-1.089445
23	1	0	0.011349	-0.428475	-1.931461
24	1	0	1.169679	-2.132889	0.949706
25	1	0	0.233668	-2.675196	-1.169060
26	1	0	-4.143781	-2.816759	0.072336
27	1	0	-1.724310	-4.173693	-0.563487
28	1	0	-2.103477	-3.460250	1.013603
29	1	0	-1.999575	-1.890074	-1.587614
30	1	0	-2.348889	1.220811	1.459547
31	1	0	-2.321224	0.391763	-1.230322
32	1	0	1.380537	4.411390	1.933120
33	1	0	2.081532	6.737184	1.458090
34	1	0	1.815087	7.661964	-0.821488
35	1	0	0.851124	6.261334	-2.618067
36	1	0	0.154889	3.939604	-2.139616
37	8	0	3.092563	-2.061521	1.572352
38	6	0	3.373145	-0.874436	1.624293
39	6	0	4.613511	-0.335499	0.985195
40	1	0	4.313690	0.222105	0.093130
41	1	0	5.106978	0.370242	1.654610
42	1	0	5.291319	-1.137348	0.702691
43	6	0	2.502183	0.108013	2.342001
44	1	0	2.987815	0.350062	3.292091
45	1	0	2.423922	1.033991	1.771280
46	1	0	1.517843	-0.313117	2.536373
47	8	0	3.544747	-2.222275	-1.565177
48	6	0	3.907144	-3.251611	-1.017605
49	6	0	5.356211	-3.551933	-0.804213
50	1	0	5.986793	-2.804190	-1.278765
51	1	0	5.547299	-3.575962	0.271792
52	1	0	5.583884	-4.547314	-1.190652
53	6	0	2.931123	-4.289176	-0.562542
54	1	0	3.254931	-4.738073	0.375698
55	1	0	1.931804	-3.871939	-0.466022
56	1	0	2.918462	-5.080668	-1.317956
57	8	0	-5.317339	-1.585033	0.848157
58	6	0	-5.577697	-0.562276	0.234822
59	6	0	-5.154992	-0.362159	-1.186851
60	1	0	-4.789291	0.654667	-1.333958
61	1	0	-4.405139	-1.091981	-1.484834
62	1	0	-6.043250	-0.477830	-1.814659
63	6	0	-6.352811	0.547450	0.870497
64	1	0	-5.674972	1.392072	1.019806
65	1	0	-7.142640	0.885857	0.198429
66	1	0	-6.767189	0.235432	1.825920

Structure 69.3DMSO (M06-2X/def2-TZVP, Gas Phase)

Energy (Hartrees): =-1515.9236375
No imaginary frequencies

Standard orientation:

Center Number	Atomic Number	Atomic Type	Coordinates (Angstroms) X	Y	Z
1	6	0	-4.864036	-1.683821	0.799629
2	6	0	-3.755641	-2.010707	0.024039
3	6	0	-3.903739	-2.894148	-1.044614
4	6	0	-5.143040	-3.439497	-1.326508
5	6	0	-6.247799	-3.108073	-0.547102
6	6	0	-6.107937	-2.229117	0.515967
7	6	0	-2.453209	-1.409798	0.354669
8	7	0	-1.396339	-1.639609	-0.295400
9	6	0	-0.181750	-0.953837	0.091111
10	6	0	-0.015433	0.303303	-0.757791
11	6	0	1.372443	0.910989	-0.555652
12	6	0	2.440823	-0.143893	-0.791896
13	8	0	2.221249	-1.277458	0.050160
14	6	0	0.999203	-1.909273	-0.138098
15	8	0	-1.038180	1.203499	-0.380718
16	8	0	0.944184	-2.460396	-1.428885
17	6	0	3.836382	0.382725	-0.512858
18	8	0	4.832774	-0.400227	-1.122382
19	8	0	1.533524	1.437896	0.743073
20	1	0	-2.452454	-0.733301	1.218016
21	1	0	-0.183335	-0.652681	1.145278
22	1	0	-0.967012	1.991748	-0.941942
23	1	0	-0.118635	0.025841	-1.813463
24	1	0	0.930527	2.184225	0.864798
25	1	0	1.502238	1.697785	-1.309829
26	1	0	5.156345	-1.045010	-0.475380
27	1	0	3.904609	1.393573	-0.926251
28	1	0	3.976440	0.452435	0.568682
29	1	0	2.394537	-0.465282	-1.837386
30	1	0	0.006585	-2.579997	-1.635632
31	1	0	0.963591	-2.709095	0.611426
32	1	0	-4.748390	-0.996289	1.630013
33	1	0	-6.965255	-1.968709	1.122898
34	1	0	-7.216075	-3.537031	-0.771095
35	1	0	-5.254305	-4.126372	-2.155480
36	1	0	-3.036107	-3.140869	-1.642245
37	8	0	-0.293295	3.751079	1.435164
38	6	0	-1.265070	3.099078	1.753792
39	6	0	-2.614314	3.343818	1.136903
40	1	0	-3.098871	2.394767	0.906909
41	1	0	-3.240365	3.878771	1.855816
42	1	0	-2.512232	3.938673	0.231552
43	6	0	-1.176397	2.015802	2.789832
44	1	0	-1.953352	2.147094	3.545324
45	1	0	-1.355119	1.064032	2.286796
46	1	0	-0.191246	2.005376	3.249137
47	8	0	-0.321471	3.638447	-1.631389
48	6	0	0.145183	4.651188	-1.156294
49	6	0	1.572715	4.738122	-0.696879
50	1	0	2.009698	3.748396	-0.585629
51	1	0	2.142603	5.303858	-1.438926
52	1	0	1.627126	5.274610	0.249894
53	6	0	-0.679540	5.900807	-1.017265
54	1	0	-0.135863	6.765328	-1.401575
55	1	0	-1.629329	5.789623	-1.534671
56	1	0	-0.851989	6.068335	0.048405
57	8	0	5.245189	-2.258683	0.932951
58	6	0	4.381858	-3.101211	0.825837
59	6	0	4.000266	-3.692476	-0.502019
60	1	0	2.929795	-3.887164	-0.558338
61	1	0	4.295858	-3.030783	-1.313032
62	1	0	4.522484	-4.648926	-0.602745
63	6	0	3.653676	-3.611484	2.039608
64	1	0	2.695902	-3.086132	2.081313
65	1	0	3.448163	-4.679447	1.964484
66	1	0	4.218144	-3.384363	2.940418

Structure 69.3DMSO (M06-2X/def2-TZVP, DMSO)

Energy (Hartrees): =-1515.9615081
No imaginary frequencies

Standard orientation:

Center Number	Atomic Number	Atomic Type	Coordinates (Angstroms) X	Y	Z
1	6	0	-4.677057	-2.039586	0.924545
2	6	0	-3.636140	-2.133308	0.003478
3	6	0	-3.841484	-2.812639	-1.198753
4	6	0	-5.070420	-3.389592	-1.468894
5	6	0	-6.106424	-3.295528	-0.542678
6	6	0	-5.909257	-2.619898	0.653449
7	6	0	-2.344209	-1.504484	0.329287
8	7	0	-1.337786	-1.565075	-0.433244
9	6	0	-0.122487	-0.886888	-0.033018
10	6	0	0.030049	0.396727	-0.843309
11	6	0	1.416067	1.007185	-0.639606
12	6	0	2.476120	-0.040807	-0.936671
13	8	0	2.283240	-1.202014	-0.131112
14	6	0	1.056456	-1.835637	-0.302940
15	8	0	-0.992651	1.279631	-0.431837
16	8	0	0.980319	-2.368075	-1.603789
17	6	0	3.878445	0.464726	-0.672747
18	8	0	4.869872	-0.405254	-1.174857
19	8	0	1.599796	1.489711	0.674068
20	1	0	-2.303440	-0.972335	1.285926
21	1	0	-0.121188	-0.626495	1.031467
22	1	0	-0.894076	2.113728	-0.920885
23	1	0	-0.075371	0.154879	-1.908157
24	1	0	0.915771	2.148545	0.871562
25	1	0	1.534558	1.820393	-1.368143
26	1	0	5.001594	-1.113938	-0.524289
27	1	0	3.996014	1.427064	-1.178010
28	1	0	4.004540	0.629850	0.400765
29	1	0	2.396033	-0.317322	-1.993049
30	1	0	0.037181	-2.448645	-1.810593
31	1	0	1.041077	-2.645236	0.434395
32	1	0	-4.515936	-1.509482	1.856645
33	1	0	-6.713150	-2.543492	1.374836
34	1	0	-7.066440	-3.748990	-0.756825
35	1	0	-5.226754	-3.914106	-2.403335
36	1	0	-3.032490	-2.881003	-1.915478
37	8	0	-0.269097	3.604358	1.531182
38	6	0	-1.261968	2.987299	1.869839
39	6	0	-2.622067	3.356238	1.358263
40	1	0	-3.266929	2.480739	1.286553
41	1	0	-3.074367	4.043778	2.080031
42	1	0	-2.546532	3.859381	0.395862
43	6	0	-1.190253	1.851052	2.841996
44	1	0	-1.916956	2.005836	3.643044
45	1	0	-1.475662	0.934219	2.320740
46	1	0	-0.188471	1.748147	3.253753
47	8	0	-0.563307	3.793157	-1.620784
48	6	0	-0.103119	4.783028	-1.083074
49	6	0	1.345211	4.881622	-0.712341
50	1	0	1.790480	3.894705	-0.600722
51	1	0	1.857819	5.412389	-1.520972
52	1	0	1.474413	5.463487	0.199214
53	6	0	-0.950251	5.986744	-0.805376
54	1	0	-0.443556	6.889539	-1.153013
55	1	0	-1.927256	5.892980	-1.274529
56	1	0	-1.066021	6.079282	0.277785
57	8	0	5.135550	-2.232275	0.958645
58	6	0	4.292189	-3.107990	0.971921
59	6	0	3.907445	-3.853125	-0.270663
60	1	0	2.842796	-4.086012	-0.277830
61	1	0	4.181161	-3.291488	-1.161233
62	1	0	4.451390	-4.803330	-0.263465
63	6	0	3.612613	-3.519519	2.241957
64	1	0	2.567572	-3.200542	2.189556
65	1	0	3.610183	-4.607064	2.335747
66	1	0	4.089983	-3.061572	3.105233

Structure 69.3DMSO (M06-2X/def2-TZVP, H_2O)

Energy (Hartrees): =-1515.9663177
No imaginary frequencies

Standard orientation:

Center Number	Atomic Number	Atomic Type	Coordinates (Angstroms) X	Y	Z
1	6	0	-4.567157	-2.102095	0.857528
2	6	0	-3.501385	-2.316983	-0.014035
3	6	0	-3.676282	-3.149558	-1.120717
4	6	0	-4.901673	-3.753135	-1.346727
5	6	0	-5.964967	-3.528932	-0.475411
6	6	0	-5.797042	-2.703235	0.627093
7	6	0	-2.226547	-1.643270	0.276219
8	7	0	-1.216030	-1.685531	-0.489322
9	6	0	-0.042611	-0.935710	-0.085396
10	6	0	0.037971	0.365721	-0.877895
11	6	0	1.372264	1.068440	-0.634549
12	6	0	2.502908	0.103640	-0.942321
13	8	0	2.379994	-1.078338	-0.145752
14	6	0	1.198511	-1.798953	-0.343366
15	8	0	-1.054816	1.170598	-0.477017
16	8	0	1.195130	-2.320998	-1.654418
17	6	0	3.871168	0.688740	-0.669035
18	8	0	4.907645	-0.116193	-1.207292
19	8	0	1.503322	1.515894	0.703986
20	1	0	-2.201087	-1.081047	1.215366
21	1	0	-0.062295	-0.690231	0.982044
22	1	0	-0.991212	2.030169	-0.927116
23	1	0	-0.027695	0.138192	-1.947936
24	1	0	0.843270	2.206930	0.875760
25	1	0	1.445186	1.915567	-1.326293
26	1	0	5.102296	-0.821608	-0.567382
27	1	0	3.930135	1.668515	-1.146814
28	1	0	4.010700	0.820972	0.406757
29	1	0	2.444111	-0.165037	-2.001310
30	1	0	0.268932	-2.450917	-1.908118
31	1	0	1.233805	-2.615741	0.382662
32	1	0	-4.426512	-1.456244	1.716907
33	1	0	-6.620598	-2.528088	1.307244
34	1	0	-6.921852	-4.001797	-0.657559
35	1	0	-5.033019	-4.401428	-2.203773
36	1	0	-2.849232	-3.321394	-1.797460
37	8	0	-0.482271	3.542470	1.535864
38	6	0	-1.411195	2.830406	1.883521
39	6	0	-2.800886	3.059574	1.379337
40	1	0	-3.311551	2.110212	1.217194
41	1	0	-3.351724	3.598231	2.156284
42	1	0	-2.793121	3.654839	0.468262
43	6	0	-1.215496	1.710117	2.854388
44	1	0	-1.941137	1.801704	3.665425
45	1	0	-1.423315	0.766941	2.343604
46	1	0	-0.201750	1.705703	3.248176
47	8	0	-0.838600	3.765974	-1.632517
48	6	0	-0.466892	4.791078	-1.082082
49	6	0	0.963916	5.001128	-0.699334
50	1	0	1.474205	4.049196	-0.565560
51	1	0	1.443394	5.550809	-1.515119
52	1	0	1.040337	5.610535	0.200062
53	6	0	-1.416302	5.909548	-0.796920
54	1	0	-0.989881	6.853334	-1.142274
55	1	0	-2.383772	5.732767	-1.260879
56	1	0	-1.532411	5.988308	0.287631
57	8	0	5.261082	-1.880035	0.963017
58	6	0	4.471161	-2.811032	0.999438
59	6	0	4.163005	-3.622962	-0.218188
60	1	0	3.113932	-3.918013	-0.233933
61	1	0	4.429611	-3.081751	-1.123759
62	1	0	4.757872	-4.539835	-0.159643
63	6	0	3.787582	-3.200550	2.269580
64	1	0	2.732164	-2.927110	2.177117
65	1	0	3.826750	-4.282614	2.403315
66	1	0	4.225504	-2.688183	3.122656
